# Supplementary material for: Looking through glass: Knowledge discovery from materials science literature using natural language processing
Source: Patterns (N Y). 2021 Jun 24;2(7):100290. doi: 10.1016/j.patter.2021.100290 (PMC8276010; doi:10.1016/j.patter.2021.100290)

Patterns, Volume *2*

Supplemental information

Looking through glass: Knowledge discovery from materials science literature using natural language processing

Vineeth Venugopal, Sourav Sahoo, Mohd Zaki, Manish Agarwal, Nitya Nand Gosvami, and N. M. Anoop Krishnan

Supplemental Items

Figure S1. Cluster plot of 50000 abstracts with 15 topics. From the initial corpus of 600000 abstracts, 50000 were randomly selected and put through an LDA algorithm which identified 15 topics as being the most predominant among them. The optimum number of topics were identified using the coherence plot shown below – which plateaus at 15. The abstracts were clustered and labelled according to their LDA topic number.

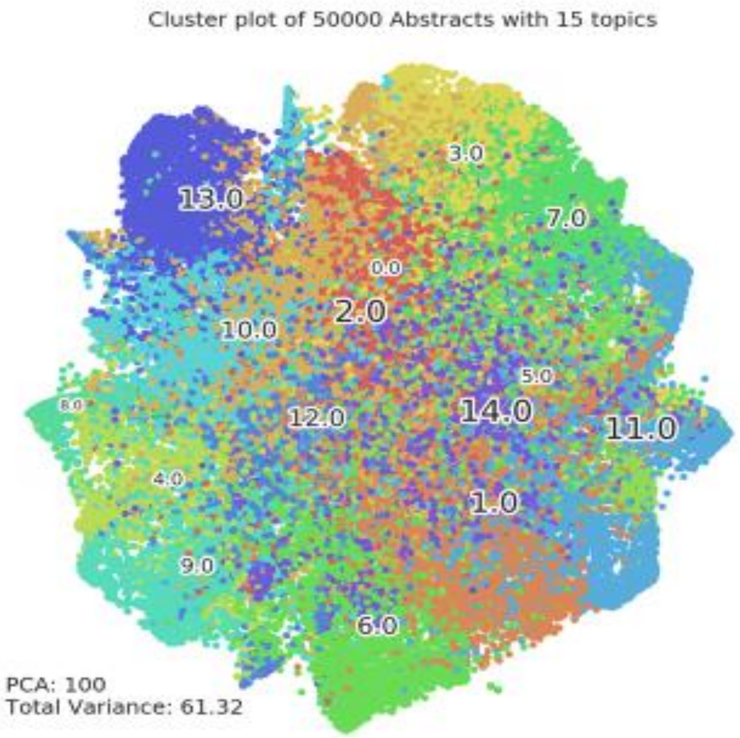

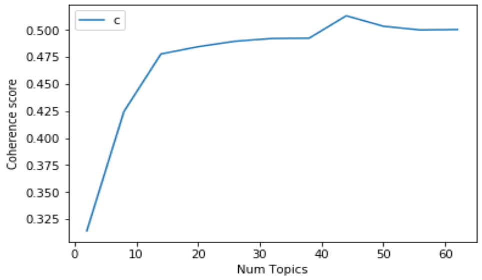

Table S1. Keywords corresponding to the topic number. The word distribution of the 15 topics showing the top ten words with the highest probability in each topic. It is seen that many topics contain words that are not directly relevant to the material science literature on oxide glasses. These abstracts were removed from the database and further classified using supervised learning.

Topic No	Keywords
0	['cells', 'protein', 'cell', 'activity', 'binding', 'structure', 'proteins', 'dna', 'human', 'enzyme']
1	['water', 'properties', 'concentration', 'ph', 'content', 'showed', 'results', 'temperature', 'polymer', 'different']
2	['glass', 'composite', 'fiber', 'fibers', 'properties', 'mechanical', 'materials', 'material', 'strength', 'results']
3	['based', 'use', 'new', 'paper', 'structure', 'methods', 'study', 'research', 'data', 'systems']
4	['temperature', 'phase', 'glass', 'transition', 'magnetic', 'fe', 'structure', 'properties', 'dielectric', 'samples']
5	['soil', 'study', 'patients', 'treatment', 'species', 'group', 'results', 'fluoride', 'significantly', 'rare']
6	['particles', 'size', 'particle', 'nanoparticles', 'ag', 'powder', 'surface', 'nm', 'silver', 'diameter']
7	['mantle', 'ma', 'pb', 'high', 'sr', 'rocks', 'ree', 'ratios', 'samples', 'composition']
8	['model', 'results', 'data', 'experimental', 'flow', 'method', 'structure', 'parameters', 'time', 'transition']
9	['la', 'ce', 'et', 'da', 'ra', 'des', 'en', 'ta', 'sa', 'une']
10	['surface', 'reaction', 'adsorption', 'high', 'fe', 'tio', 'carbon', 'oxidation', 'metal', 'prepared']
11	['water', 'data', 'area', 'zone', 'high', 'basin', 'region', 'formation', 'sediments', 'changes']
12	['films', 'optical', 'nm', 'film', 'emission', 'absorption', 'properties', 'glass', 'doped', 'energy']
13	['properties', 'mechanical', 'composites', 'strength', 'al', 'high', 'microstructure', 'temperature', 'tensile', 'alloy']
14	['structure', 'complexes', 'ii', 'compounds', 'complex', 'crystal', 'molecular', 'spectra', 'metal', 'structures']

Three thousand and sixty randomly selected abstracts were manually labelled as 'glass' and 'not glass' from among these articles. They were then subjected to 5-fold cross validation. Three machine learning models – logistic regression, naïve bayes, and random forest were trained on this data for classification. The results of the models on the training set are shown below.

Table S2. Model Performance on Training Set.

Method	Precision, %	Accuracy, %	Recall, %	Confusion Matrix
Logistic Regression	93	88	73	[1205, 40<br>195, 518]
Naïve Bayes	98	87	67	[1220, 8<br>238, 492]
Random Forest	100	100	100	[1253, 0<br>0, 705]

Naïve Bayes and Random Forest were found to perform the best in terms of precision and accuracy. However, logistic regression was found to give higher recall than random forest and also performed well on the test and validation set as shown below.

Table S3. Model Performance on Validation Set.

Method	Precision, %	Accuracy, %	Recall, %	Confusion Matrix
Logistic Regression	93	86	69	[295, 9<br>58, 128]
Naïve Bayes	99	88	66	[320, 1<br>58, 111]
Random Forest	97	84	63	[292, 4<br>72, 122]

Table S4. Model Performance on Test Set.

Method	Precision, %	Accuracy, %	Recall, %	Confusion Matrix
Logistic Regression	92	86	67	[374, 13<br>74, 151]
Naïve Bayes	98	83	55	[384, 3<br>102, 123]
Random Forest	95	84	60	[380, 7<br>91, 134]

We note that the precision of the model is very high, which suggests that number of false positives ("non-glass" classified as "glass") is minimal. However, we note that the recall on the validation set is relatively low for all the models, which suggests that the false negatives ("glass" classified as "non-glass") is very high. Low recall, thus, leads to the missing of several papers belonging to the "glass" topic. In our case, thus, the model with the highest recall on validation set was given preference to include as many glass-related papers as possible. This does mean that some non-glass articles are included in the final corpus but their effect on the final results was found to be minimal.

Figure S2. The distribution of chemicals in the database as identified by ChemDataExtractor.

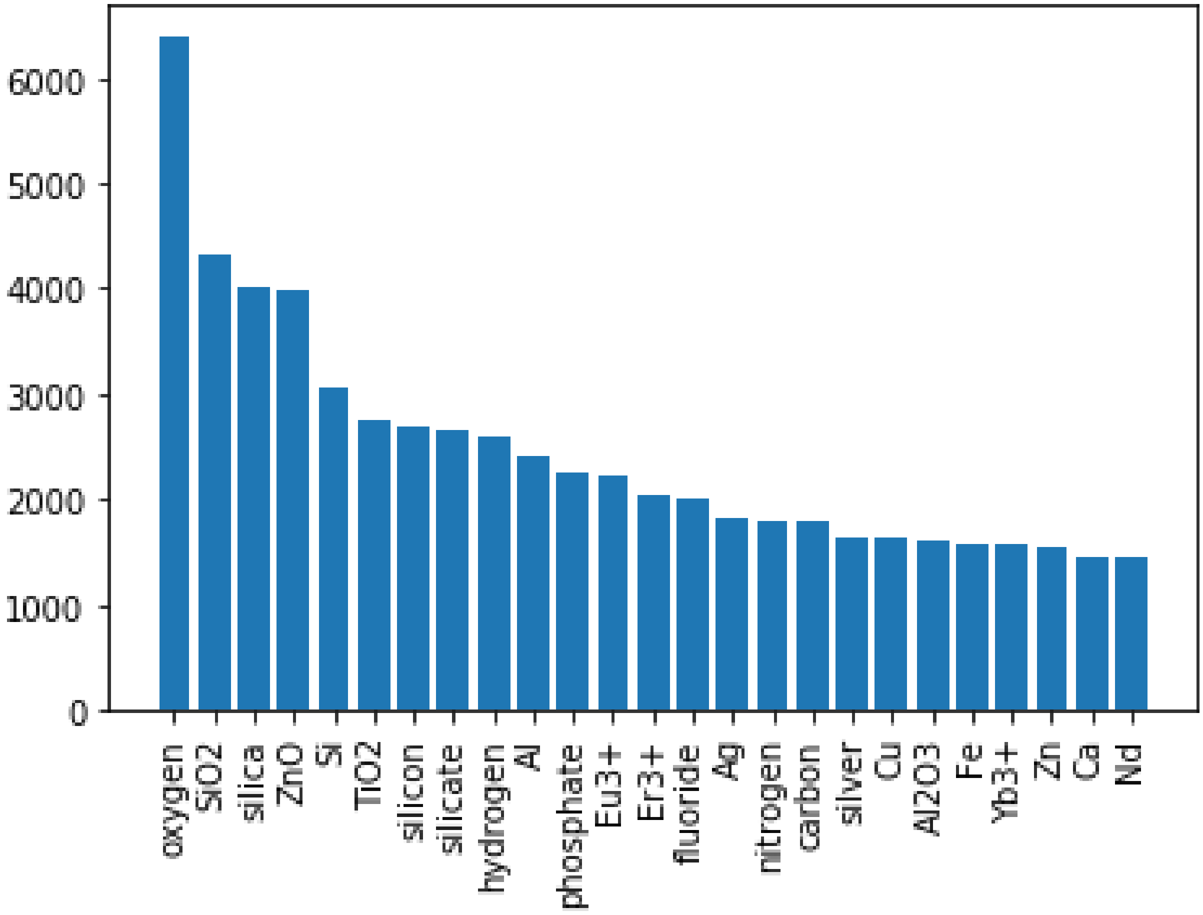

Figure S3. LDA topic modeling for topic number 5. Each topic from the LDA plot can be further subjected to another round of LDA topic classification – as shown here for topic no 5 –thin films on glasses. The optimum number of topics was found to be 7 through the coherence plot. 12866 abstracts are contained in Topic 5, which is clustered separately and labelled in the inset diagram shown alongside.

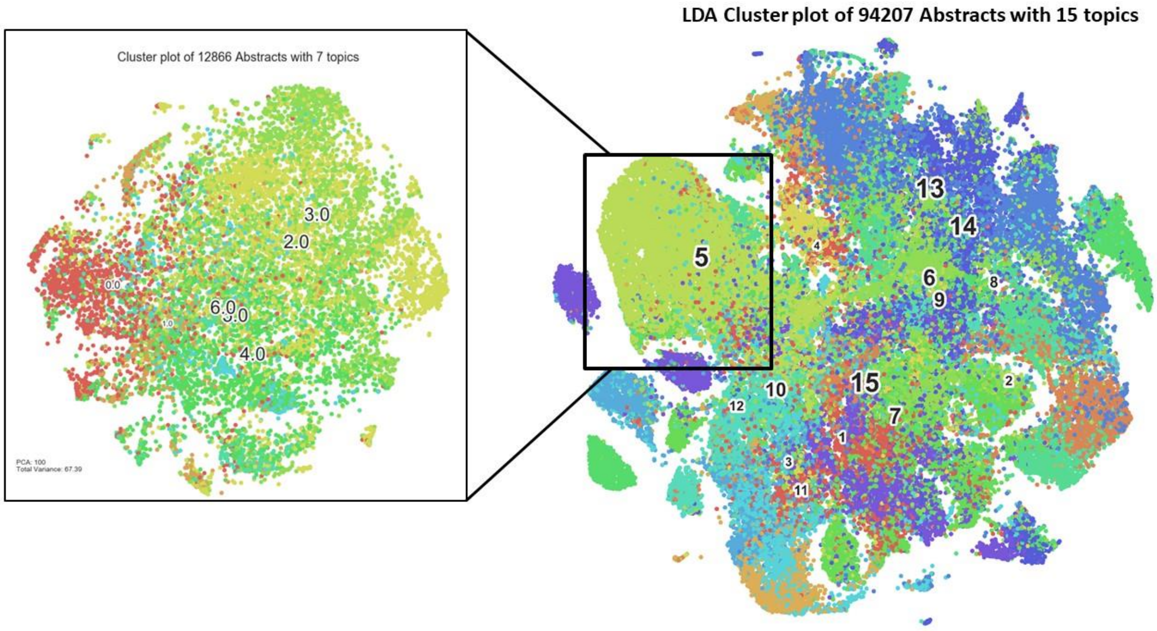

The words with the highest probability distribution in each topic is listed separately in the Table S5 at the bottom.

Table S5. List of keywords associated with each topic.

Topic No	Keywords
0	['glass', 'layer', 'tio', 'film', 'light', 'ito', 'oxide', 'nm', 'solar', 'ag']
1	['films', 'surface', 'glass', 'nm', 'film', 'ions', 'irradiation', 'emission', 'gas', 'luminescence']
2	['films', 'optical', 'band', 'ray', 'gap', 'deposited', 'ev', 'film', 'properties', 'energy']
3	['films', 'zno', 'doped', 'optical', 'properties', 'temperature', 'electrical', 'deposited', 'doping', 'film']
4	['films', 'si', 'deposition', 'deposited', 'glass', 'film', 'silicon', 'substrate', 'sio', 'temperature']
5	['laser', 'growth', 'grown', 'cu', 'temperature', 'pulsed', 'emission', 'substrate', 'ga', 'layer']
6	['films', 'doped', 'dielectric', 'ti', 'film', 'properties', 'mg', 'si', 'fe', 'doping']

Supplemental Experimental Procedures

Section 1. HYPER PARAMETERS for t-SNE, TFIDF and LDA

1. t-SNE
    a. n_components = 2
    b. metric = 'precomputed'
    c. perplexity = 40
    d. n_iter = 300
    e. random_state = 92
2. TFIDF
    a. min_df = 100
    b. max_df = 0.9
    c. ngram_range = (1,3)
3. LDA
    a. num_topics = 15
    b. passes = 10
    c. chunksize = 1000

Demonstration of NLP algorithms to other topics in Material Science

The techniques developed in the paper can easily be applied to any field of knowledge. We demonstrate this here with two specific applications within material science: magnesium alloys and metallic glasses. Magnesium alloys are a class of metals that contain magnesium as the main alloying agent. They find many uses in structural applications such as in aeronautics. Metallic glasses are alloys of metals that resemble glasses in their atomic structure. They find applications where high strength is desirable, for example in making shaving blades. Both these topics are active areas of research. The NLP tools developed in this paper can greatly assist researchers and other members of the community to selectively query, visualize, and interact with the application space.

Elemental Map for Metallic Glasses

A corpus of around 30,000 articles on Metallic glasses were extracted from Elsevier. The abstracts of these articles were extracted and used to create an LDA plot as explained in the Methodology section. Chemical elements were extracted from these documents and individual markers for each element were created. By superimposing these markers on the LDA plot, the application subspace of each element can be visualized with respect to the total application space of Metallic glasses. Only the maps for copper and Iron are shown here. It is seen that these metals are areas where there are both used and certain applications where one metal is preferred over the other. The extraction and visualization of this information is currently impossible through any other means.

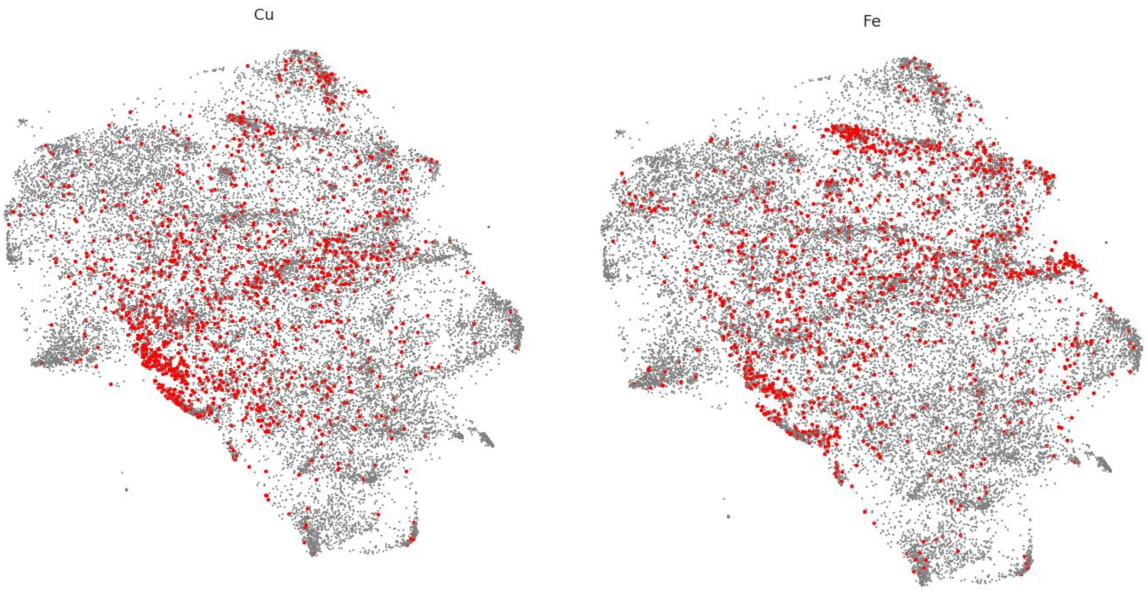

Figure S4. Elemental Map for Metallic Glasses. The Elemental maps for metallic glasses plotted here for copper and Iron. The graphs clearly demonstrate that they share common applications as well as regions where one is preferred over the other.

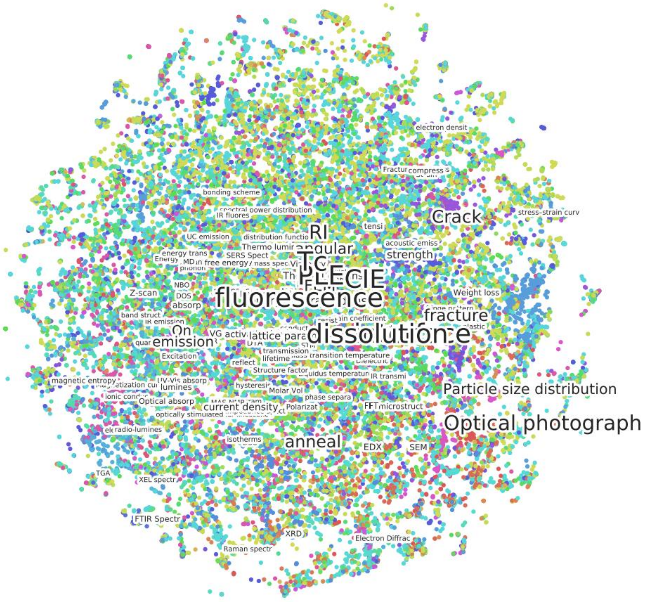

Figure S5. Caption Cluster Plot for Magnesium alloys. A corpus of around 40,000 articles on Magnesium alloys were extracted from Elsevier. The abstracts of these articles were extracted and used to create a CC plot as explained in the Methodology section.

Section 2. Elemental Maps (CCP and LDA plots for elements)

Figure S6. Caption Cluster plot for Hydrogen.

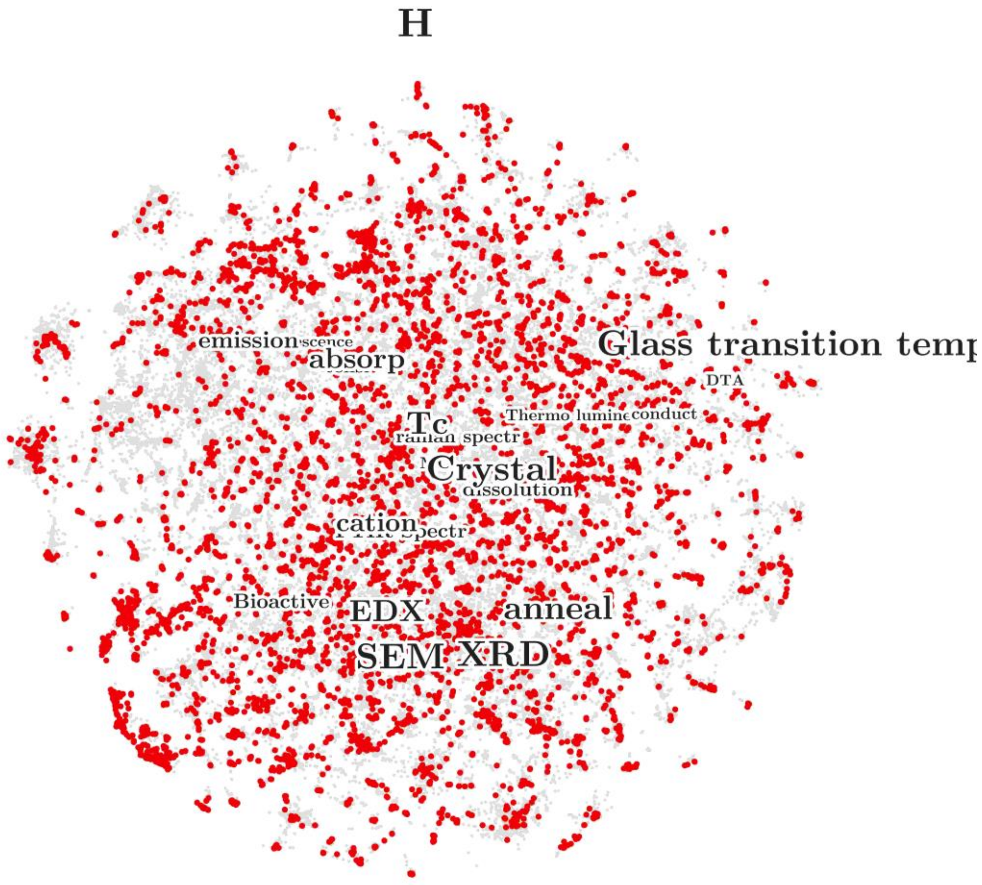

Figure S7. Latent Dirichlet Allocation plot for Hydrogen.

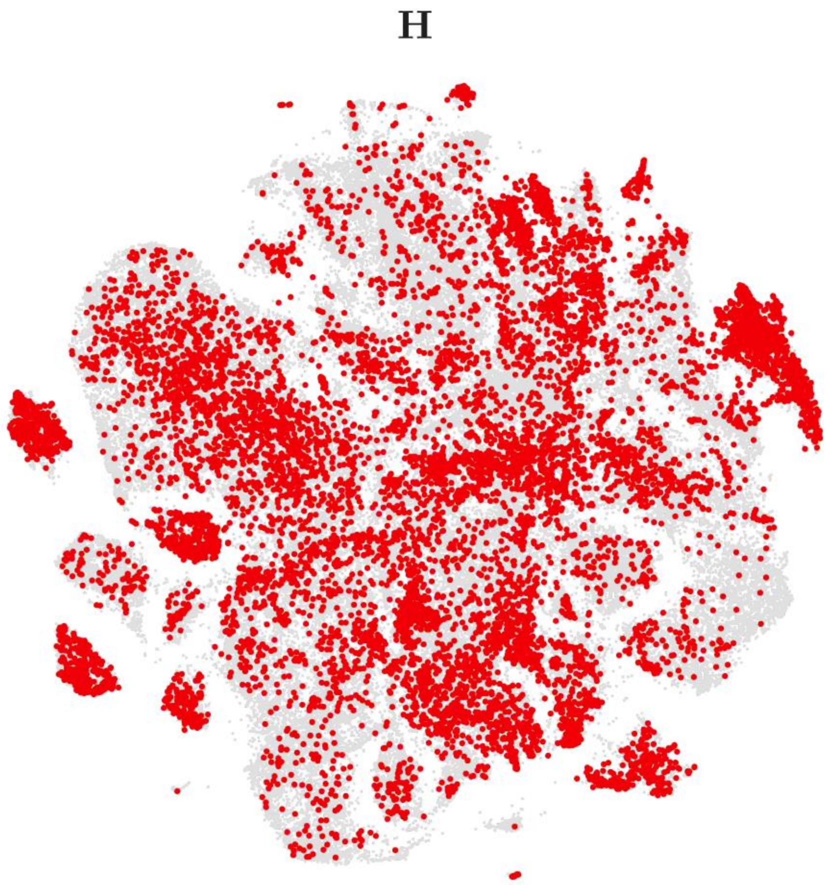

Figure S8. Caption Cluster plot for Helium.

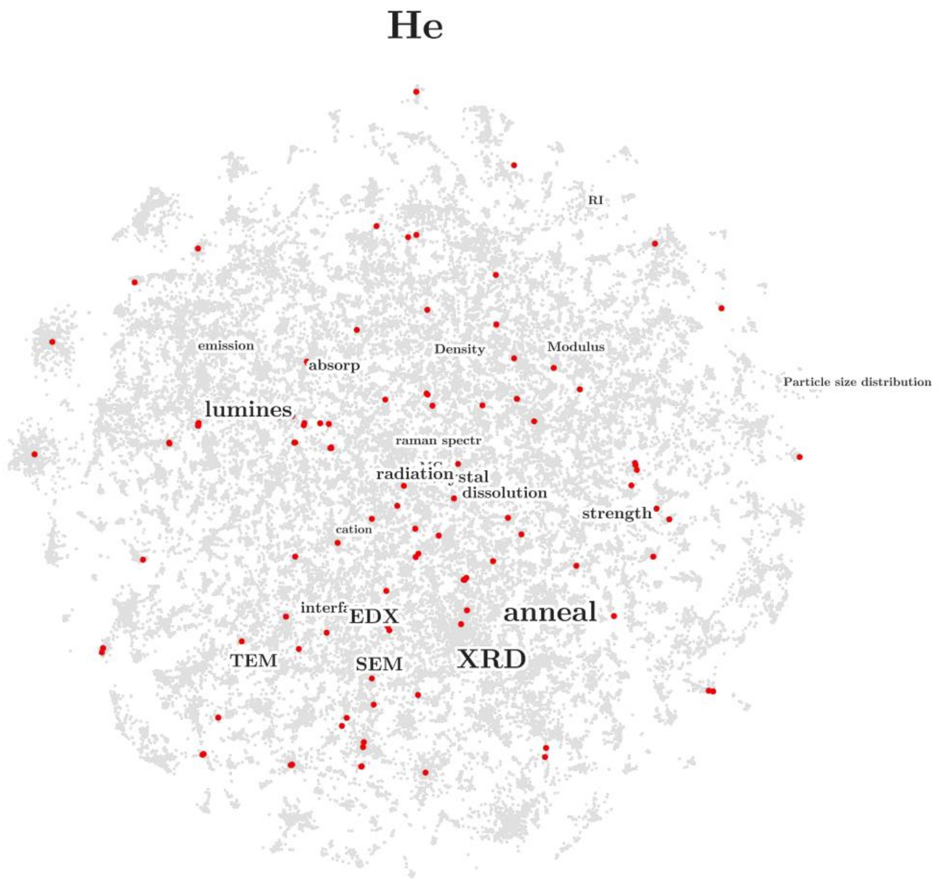

Figure S9. Latent Dirichlet Allocation plot for Helium.

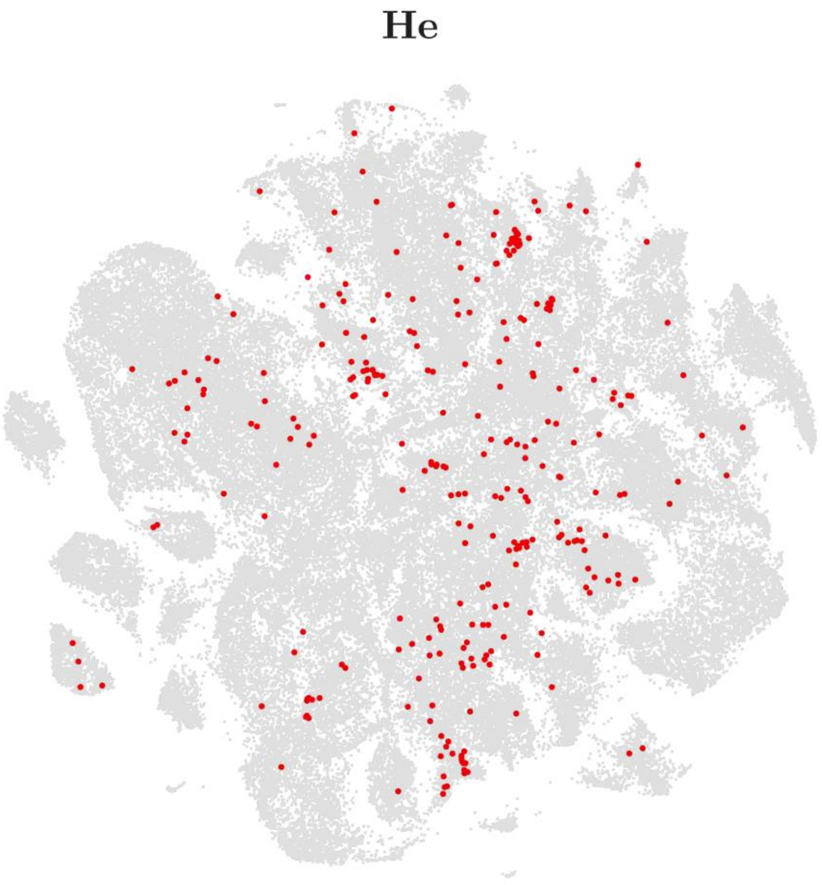

Figure S10. Caption Cluster plot for Lithium.

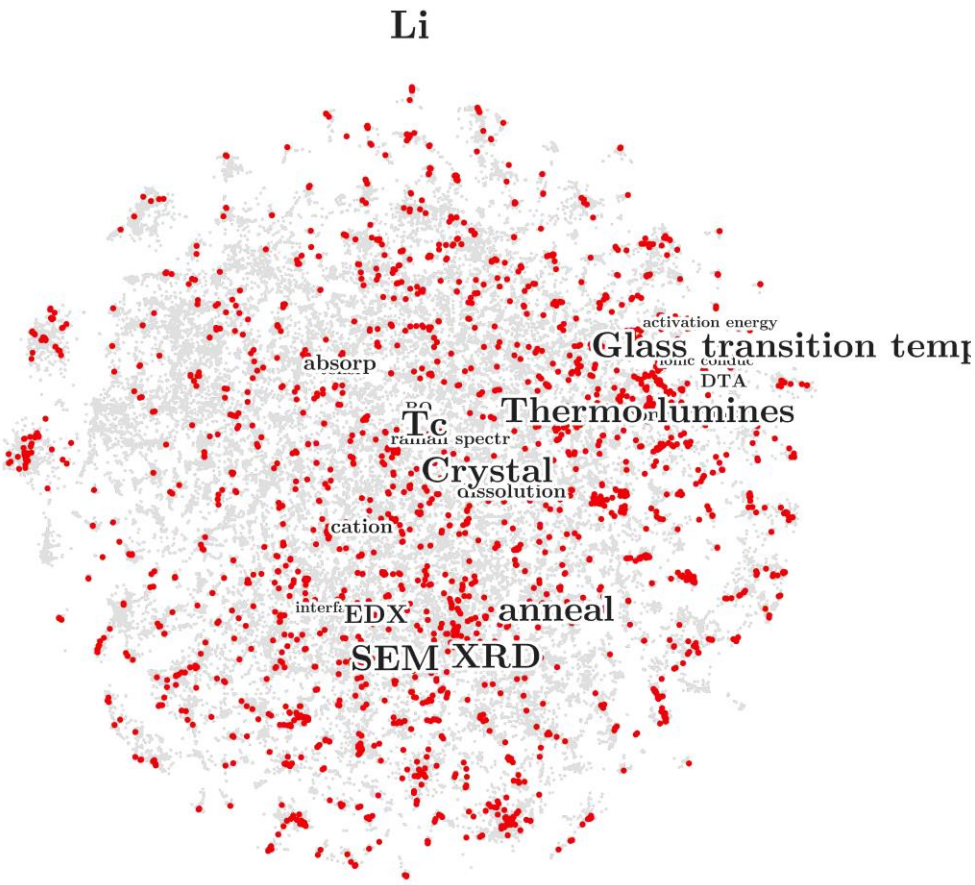

Figure S11. Latent Dirichlet Allocation plot for Lithium.

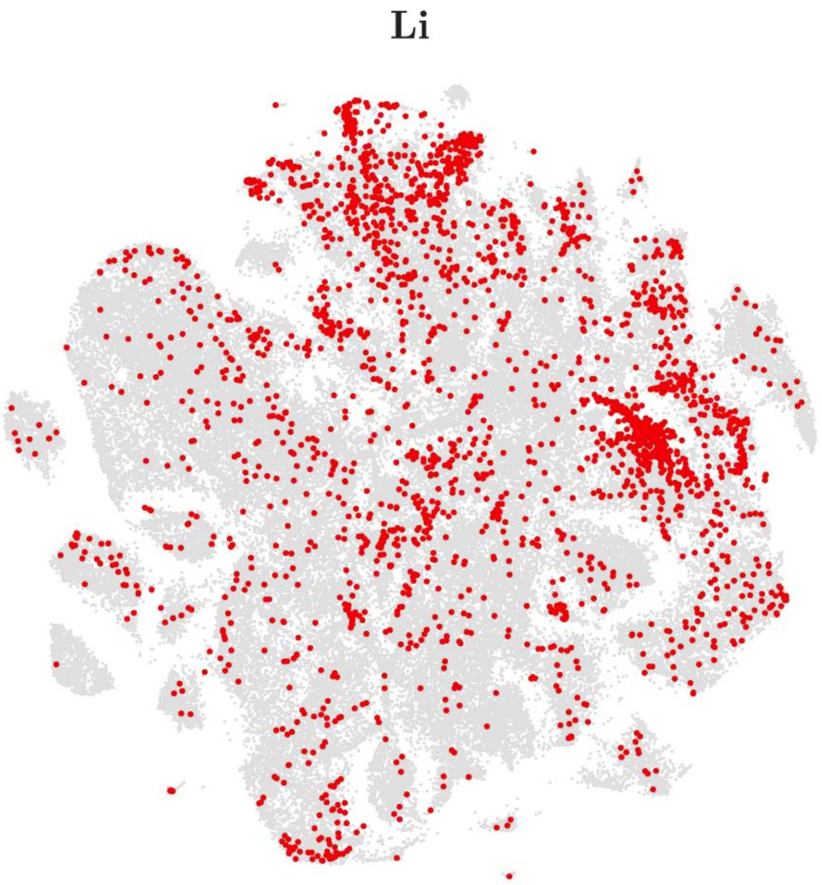

Figure S12. Caption Cluster plot for Beryllium.

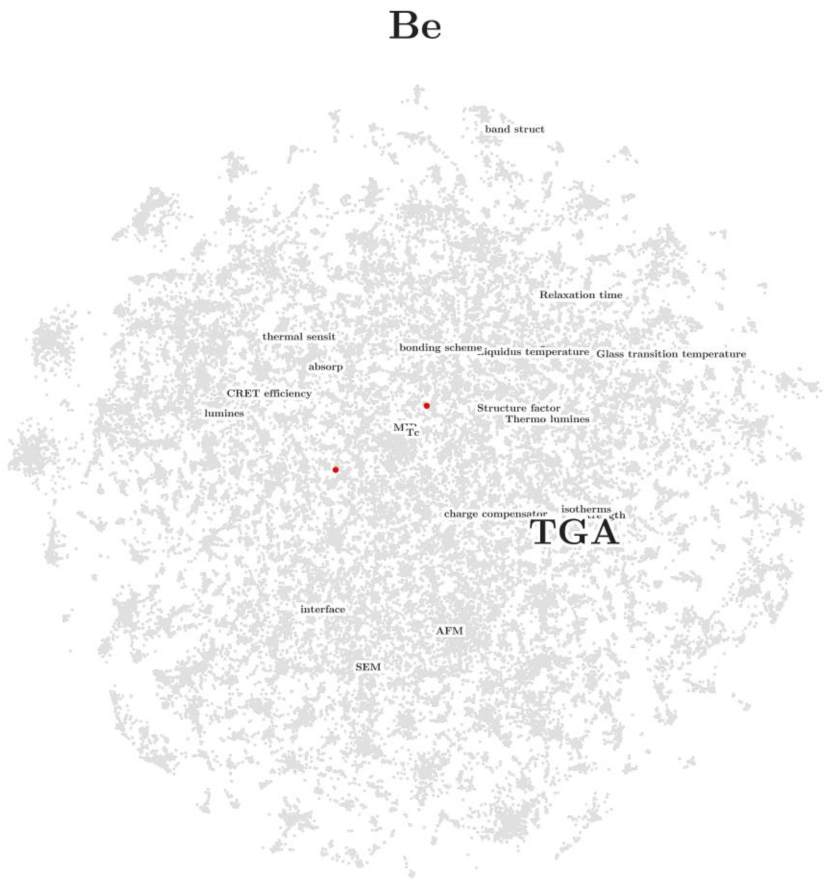

Figure S13. Latent Dirichlet Allocation plot for Beryllium.

Be

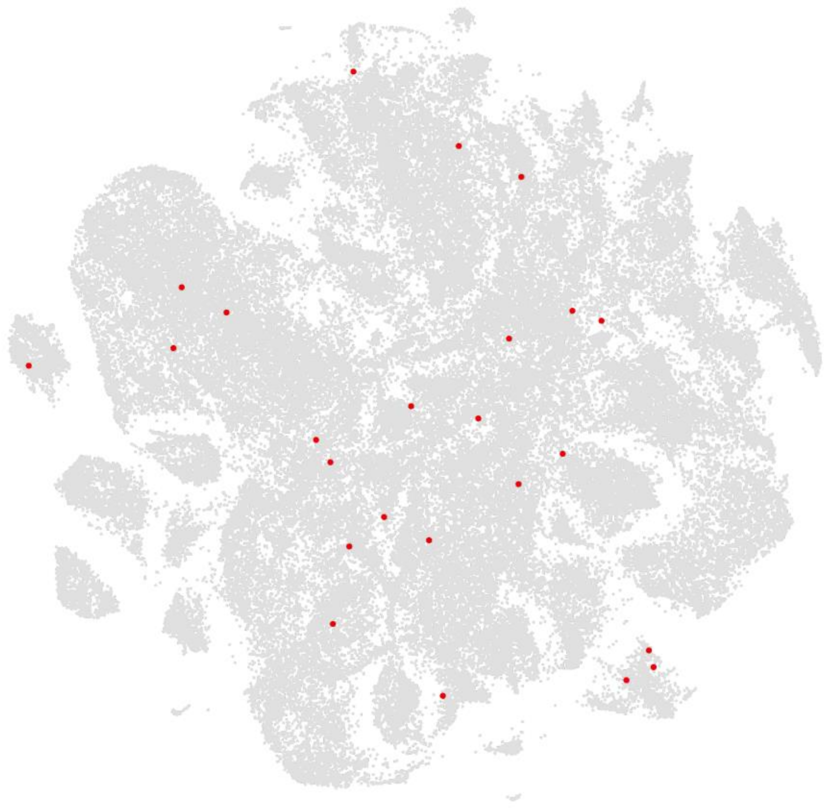

Figure S14. Caption Cluster plot for Boron.

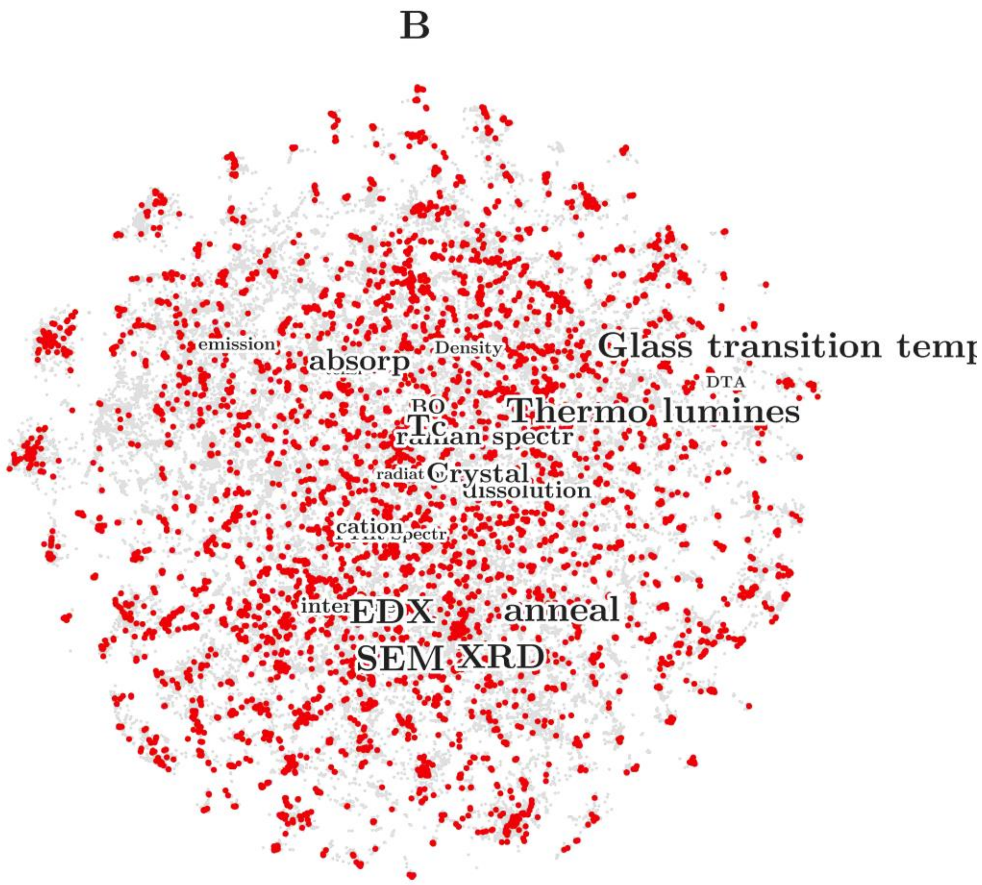

Figure S15. Latent Dirichlet Allocation plot for Boron.

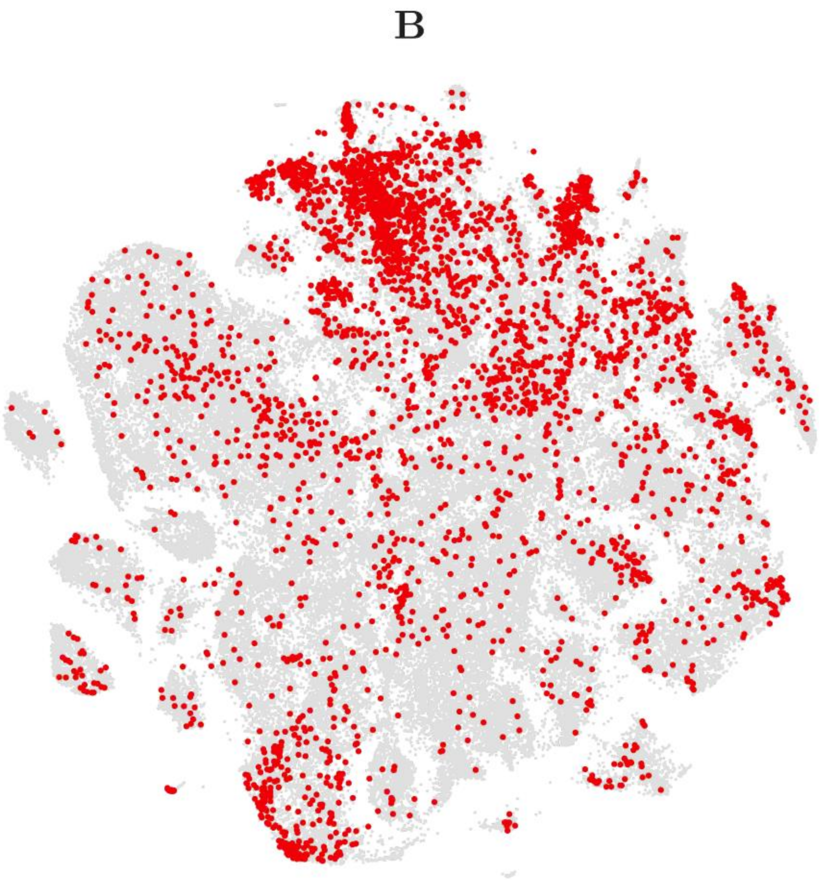

Figure S16. Caption Cluster plot for Carbon.

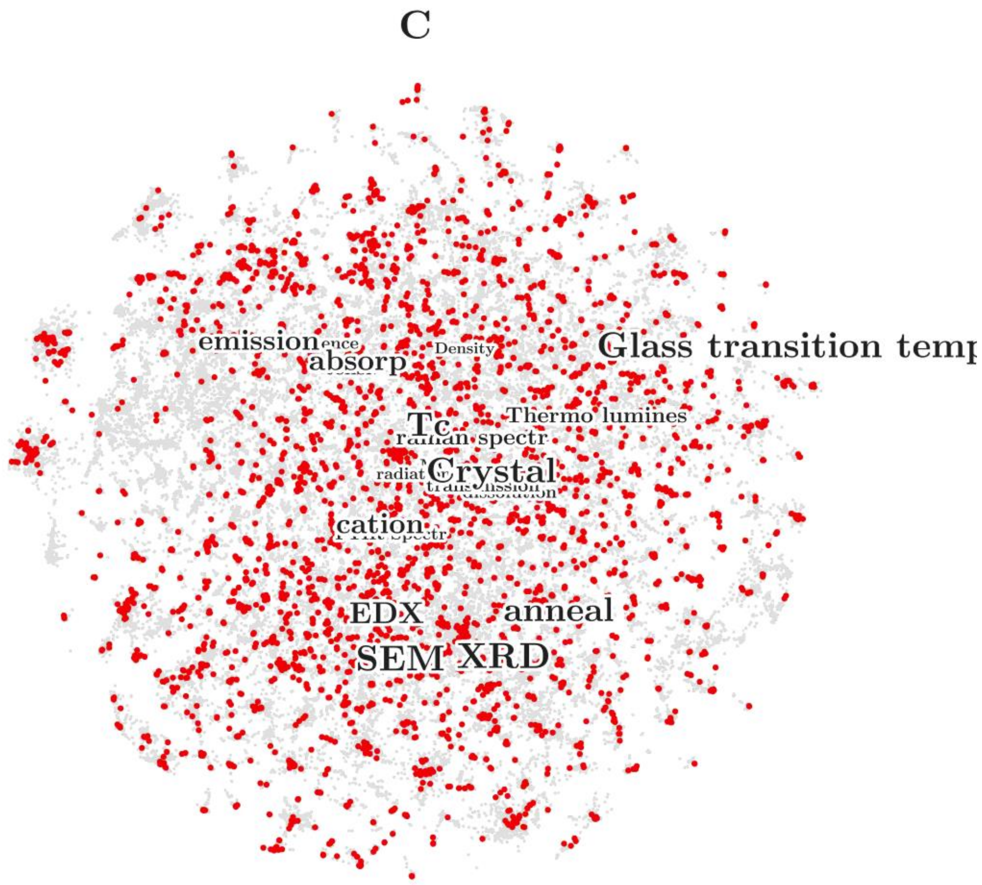

Figure S17. Latent Dirichlet Allocation plot for Carbon.

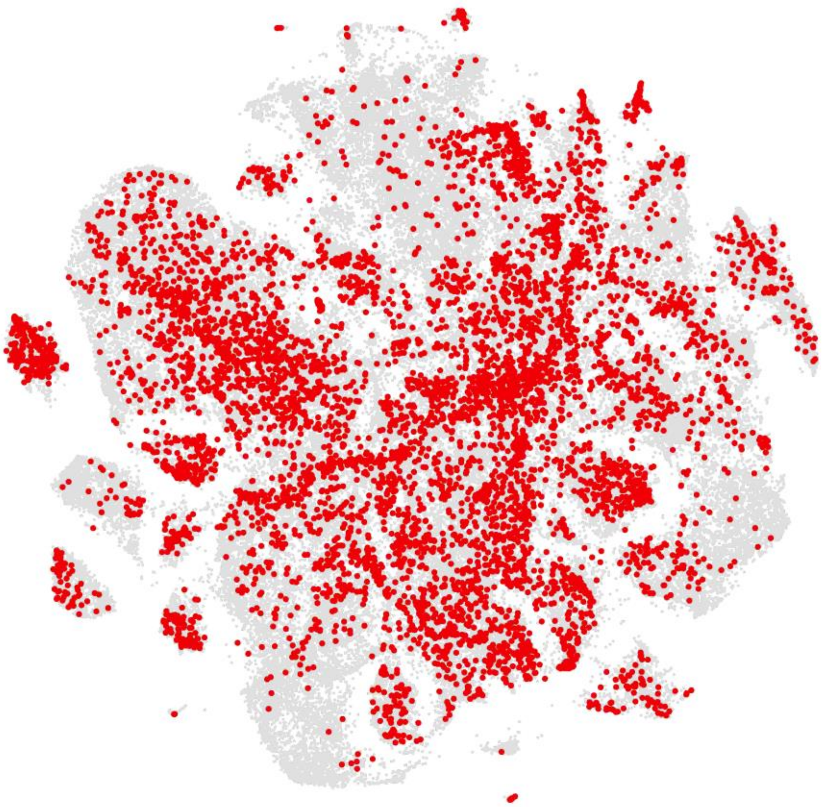

Figure S18. Caption Cluster plot for Nitrogen.

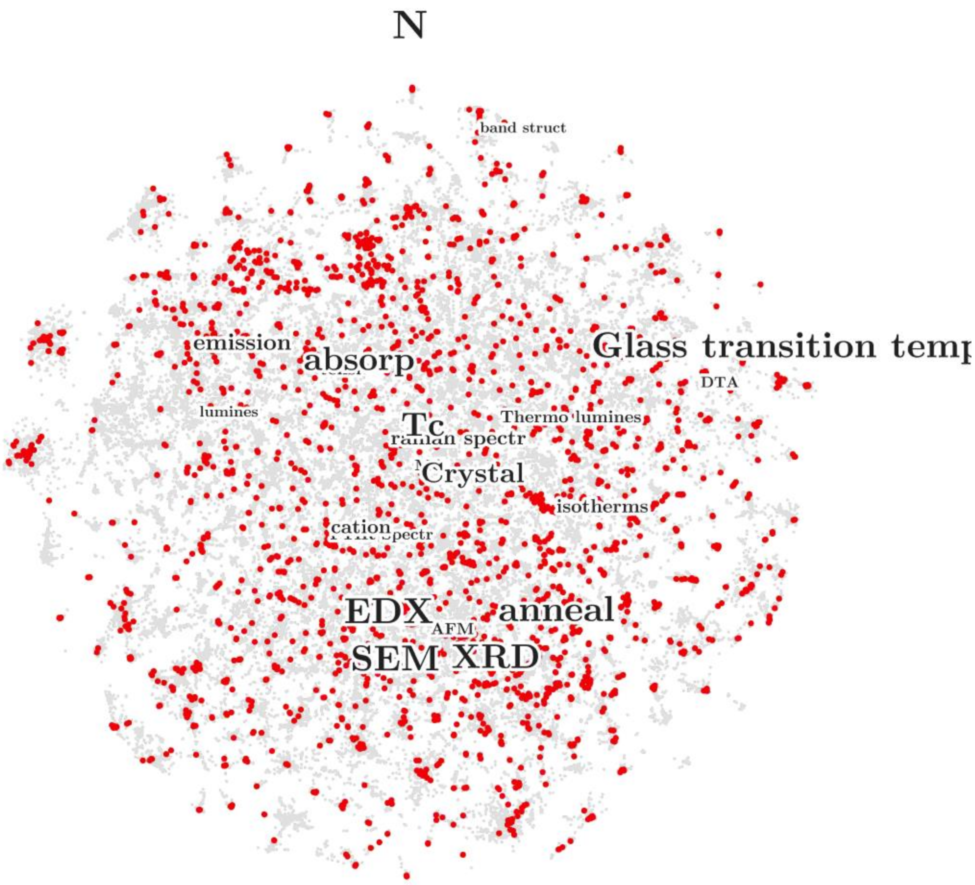

Figure S19. Latent Dirichlet Allocation plot for Nitrogen.

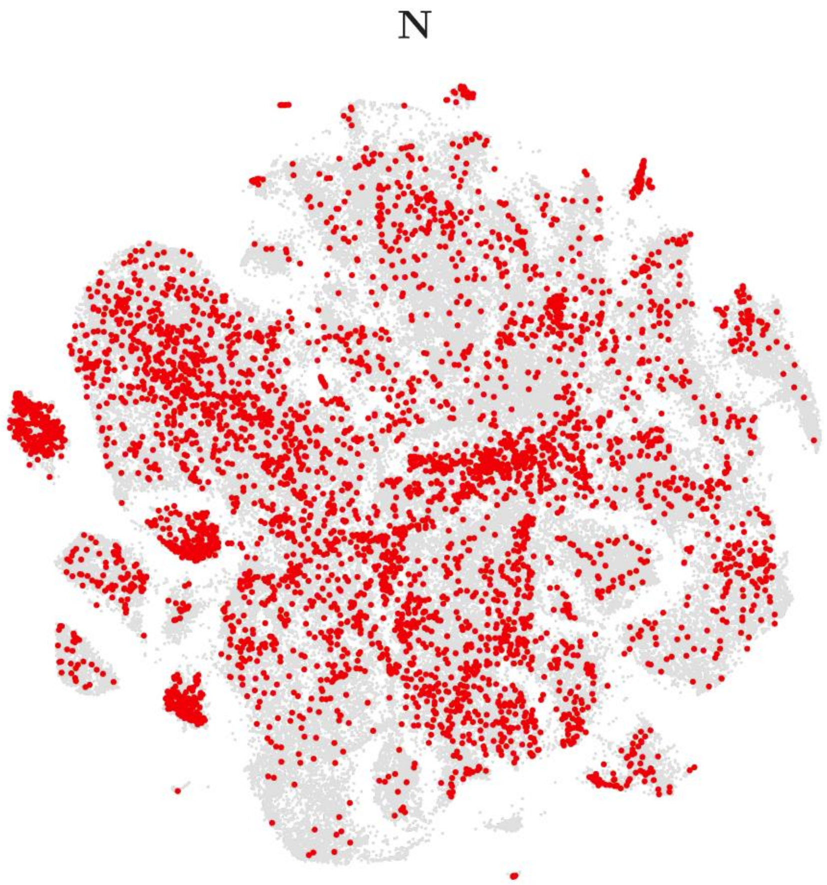

Figure S20. Caption Cluster plot for Oxygen.

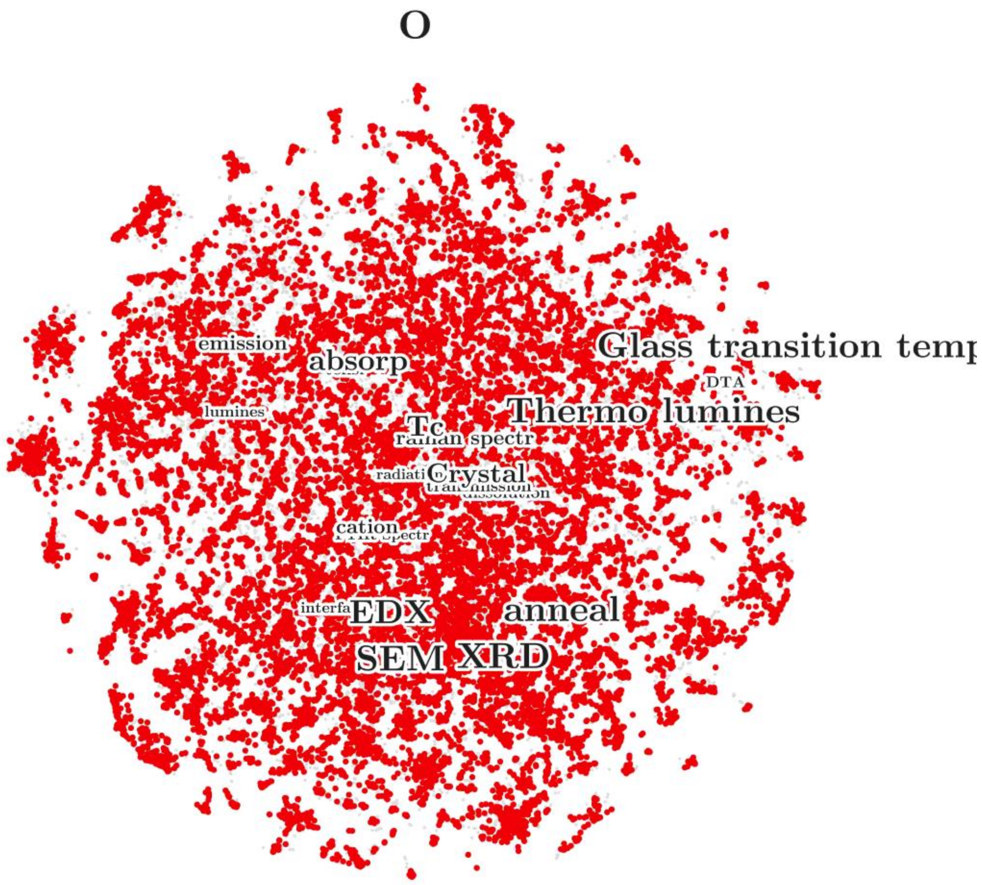

Figure S21. Latent Dirichlet Allocation plot for Oxygen.

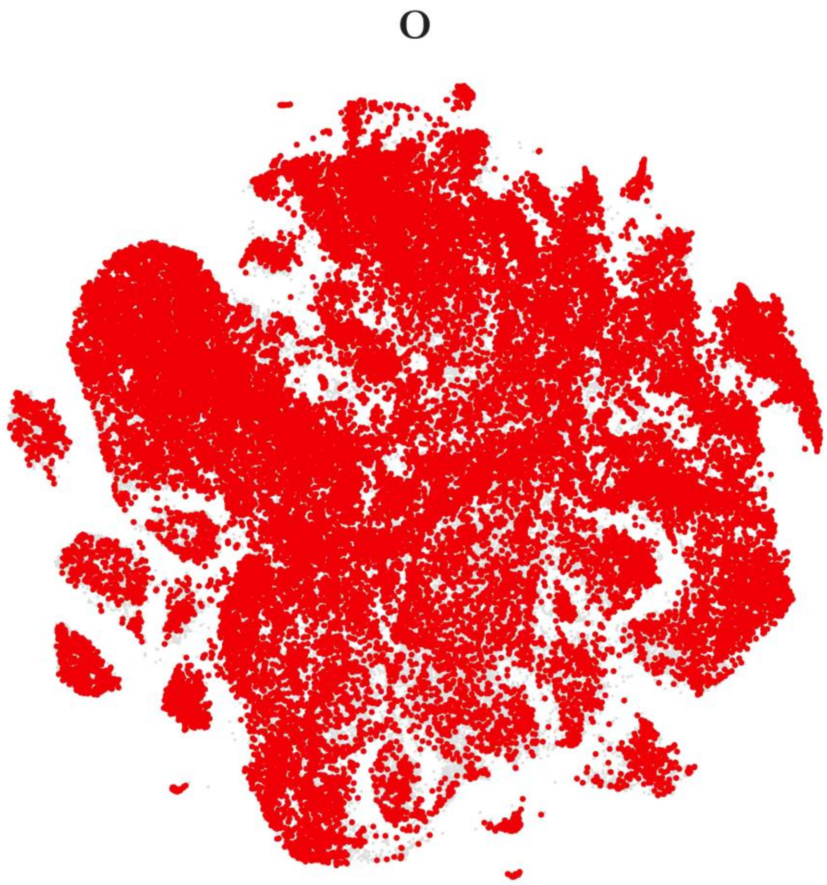

Figure S22. Caption Cluster plot for Fluorine.

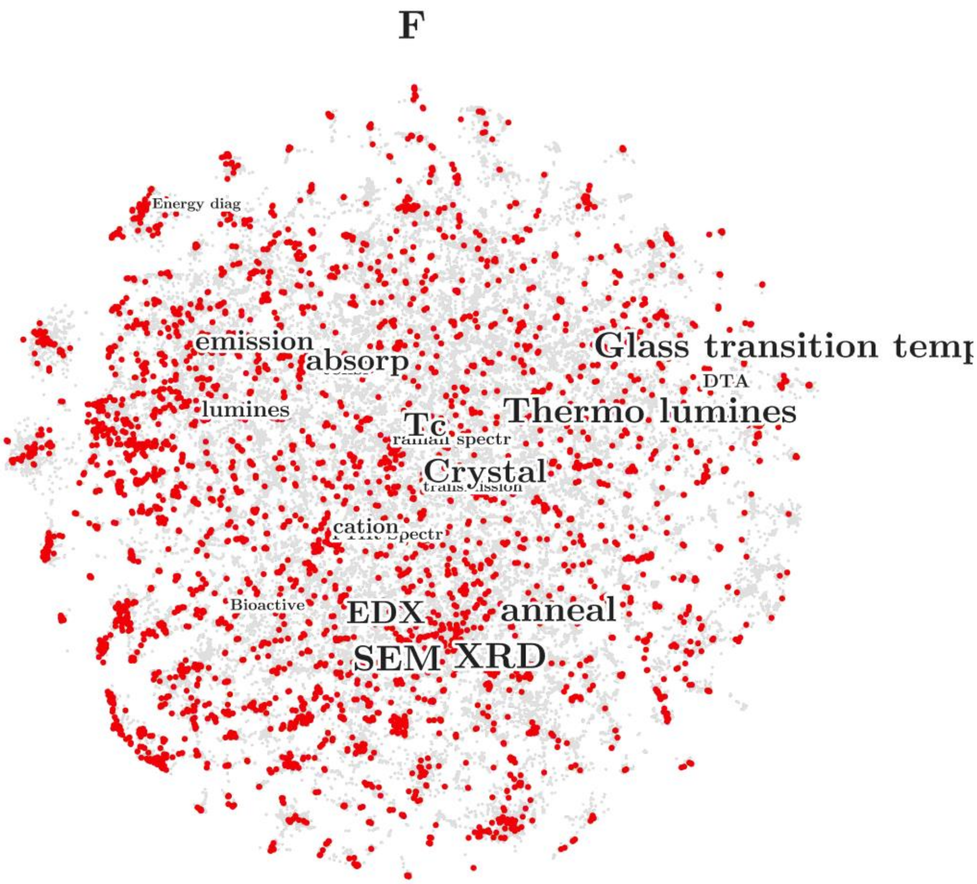

Figure S23. Latent Dirichlet Allocation plot for Fluorine.

F

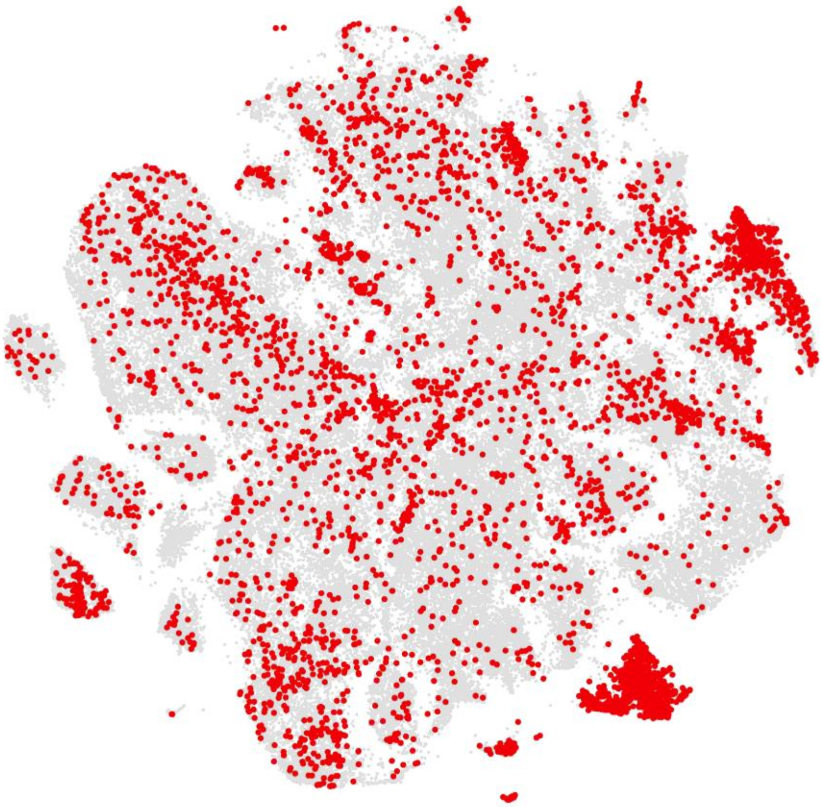

Figure S24. Caption Cluster plot for Neon.

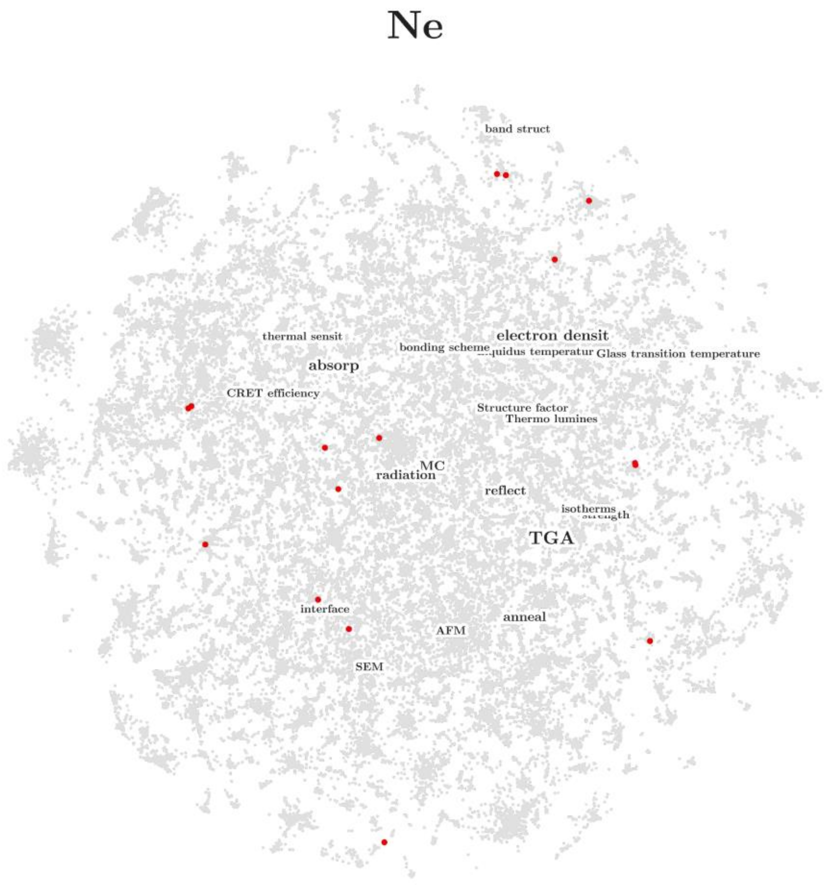

Figure S25. Latent Dirichlet Allocation plot for Neon.

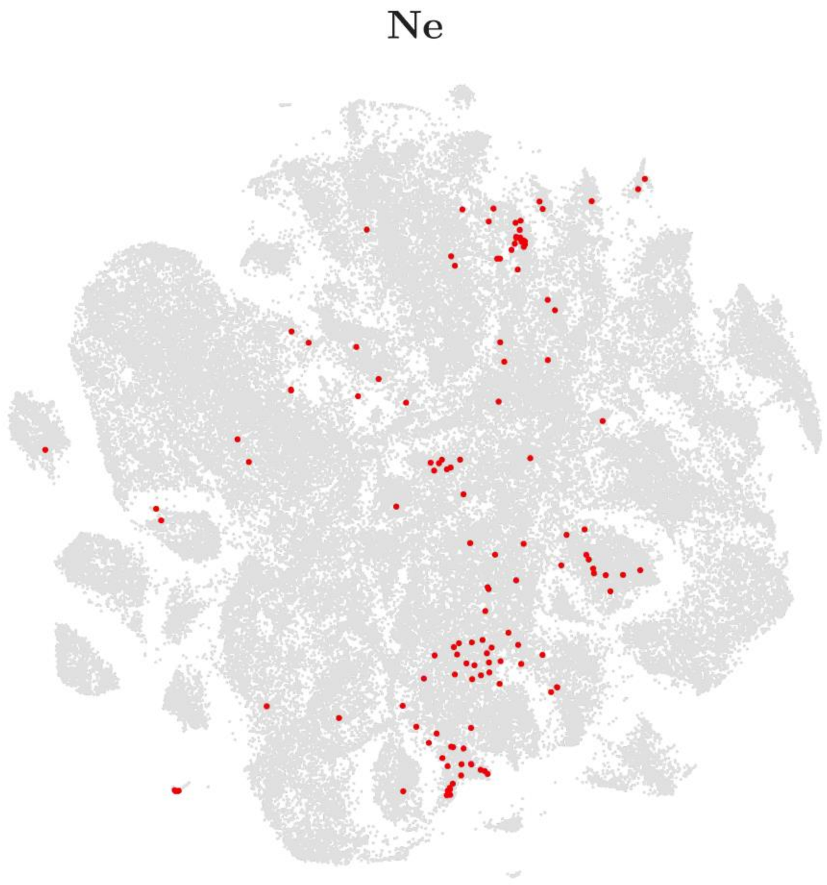

Figure S26. Caption Cluster plot for Sodium.

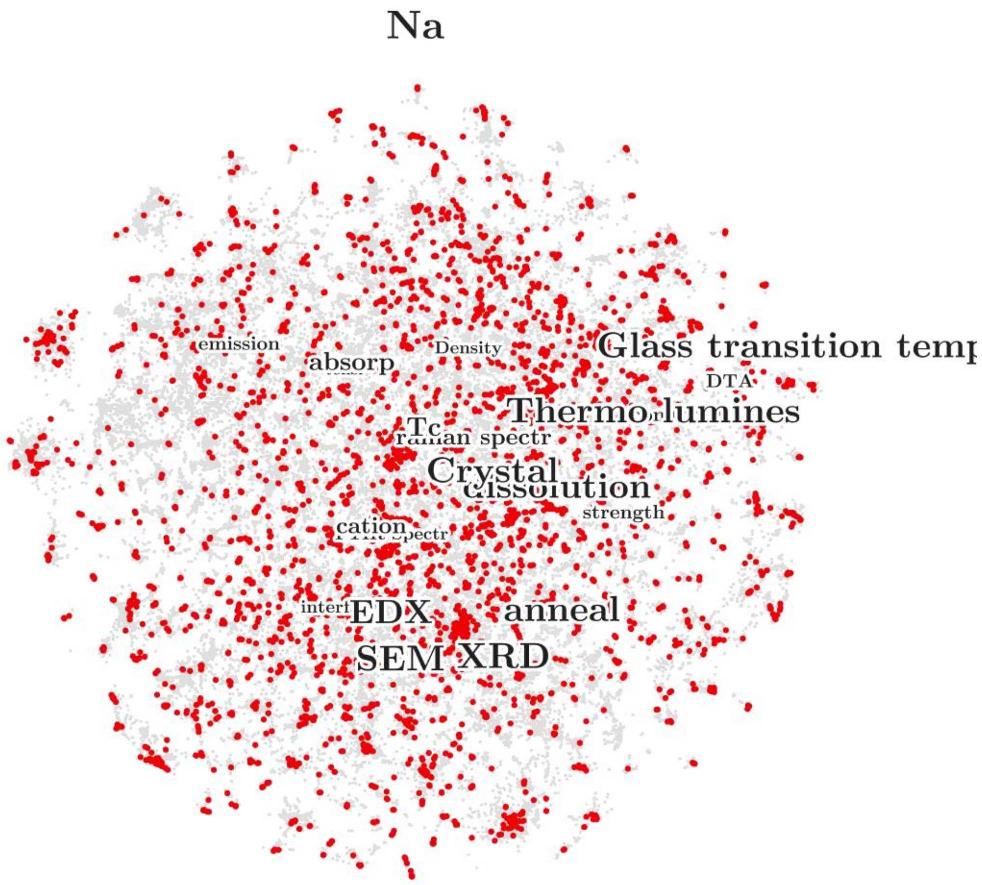

Figure S27. Latent Dirichlet Allocation plot for Sodium.

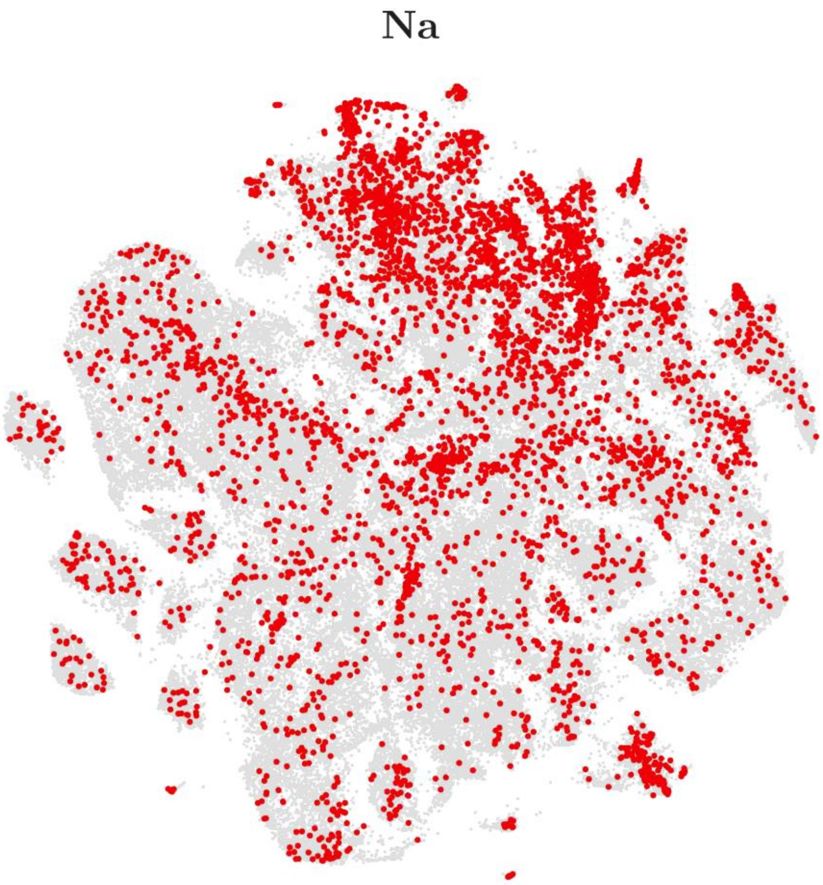

Figure S28. Caption Cluster plot for Magnesium.

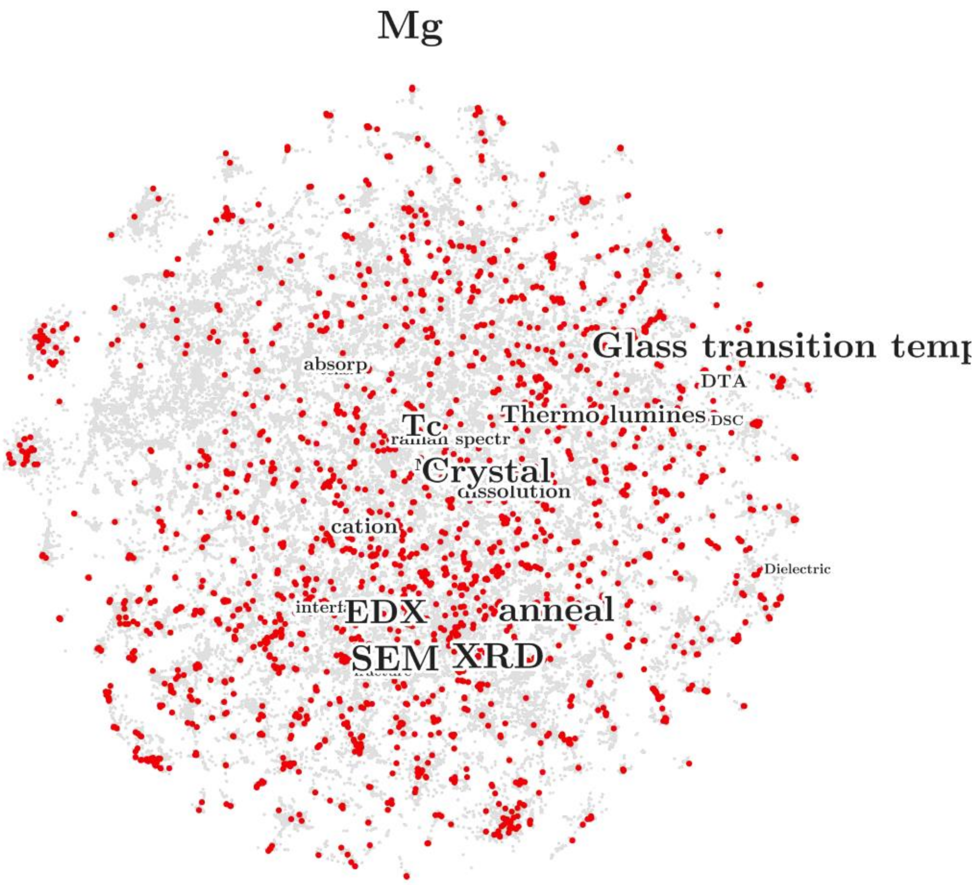

Figure S29. Latent Dirichlet Allocation plot for Magnesium.

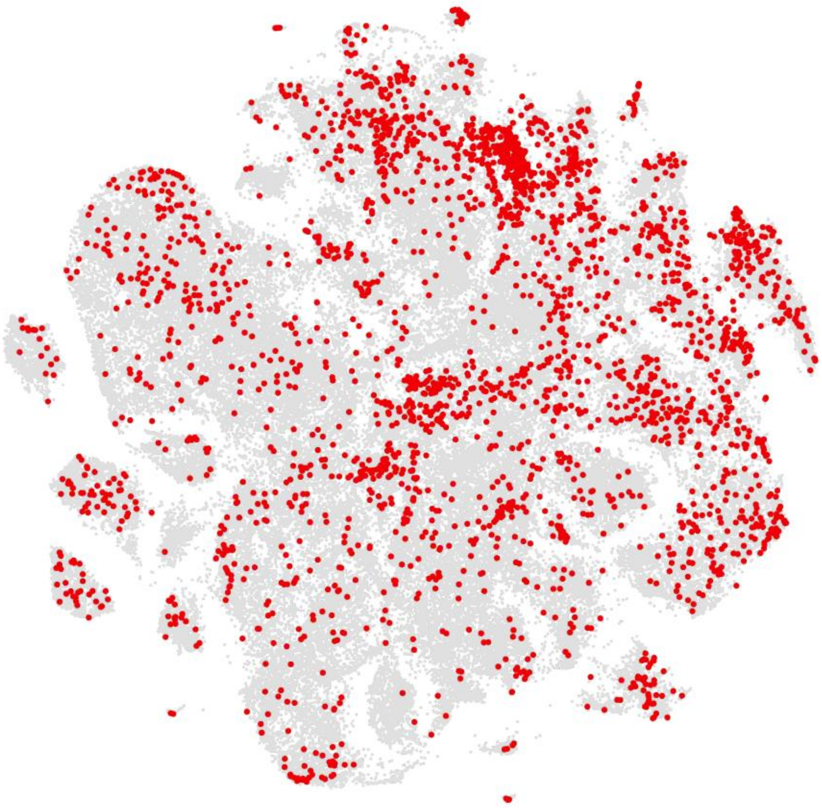

Figure S30. Caption Cluster plot for Aluminium.

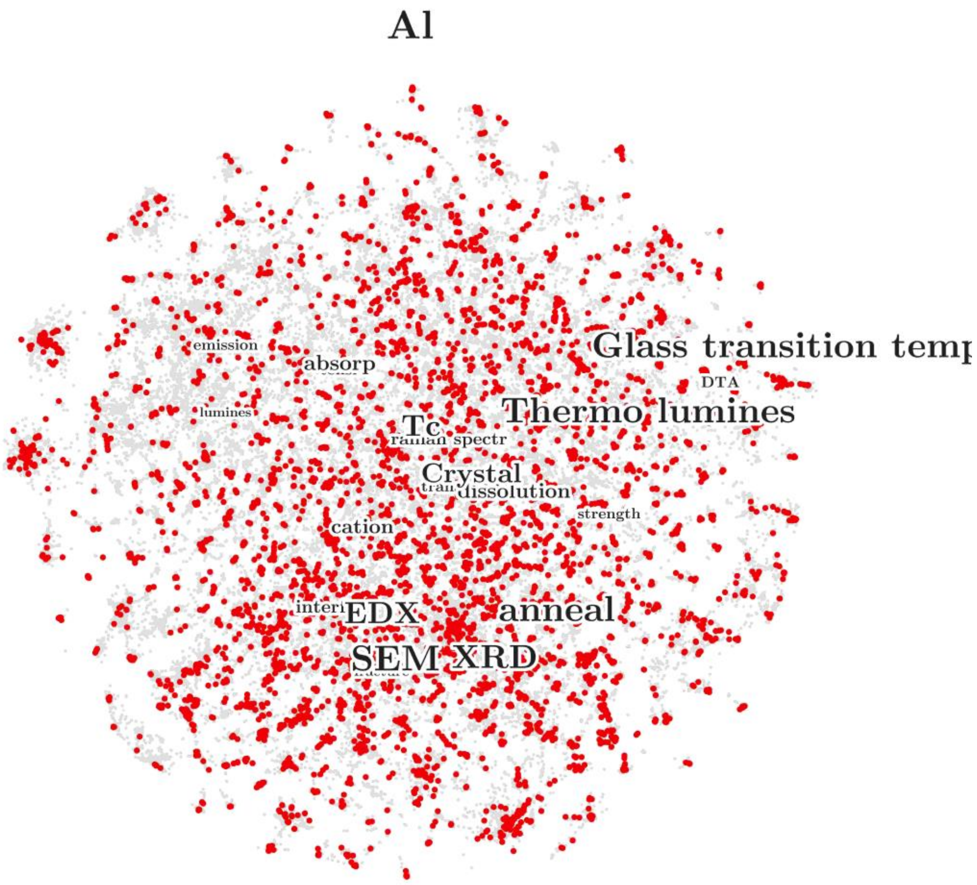

Figure S31. Latent Dirichlet Allocation plot for Aluminium.

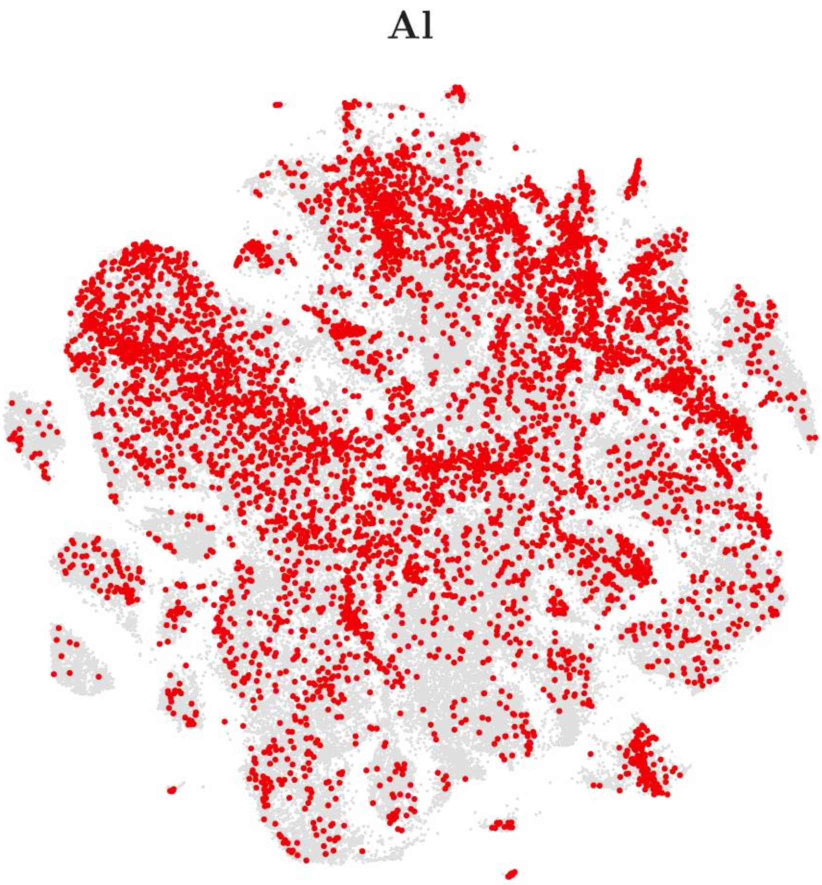

Figure S32. Caption Cluster plot for Silicon.

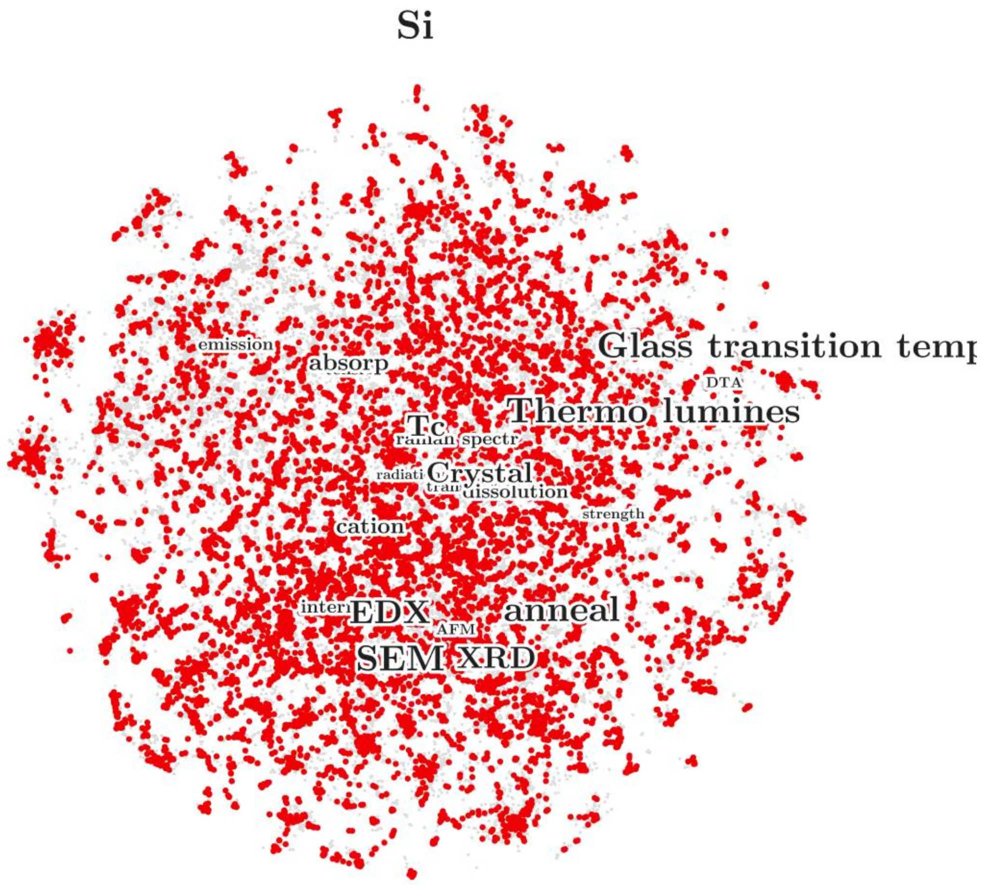

Figure S33. Latent Dirichlet Allocation plot for Silicon.

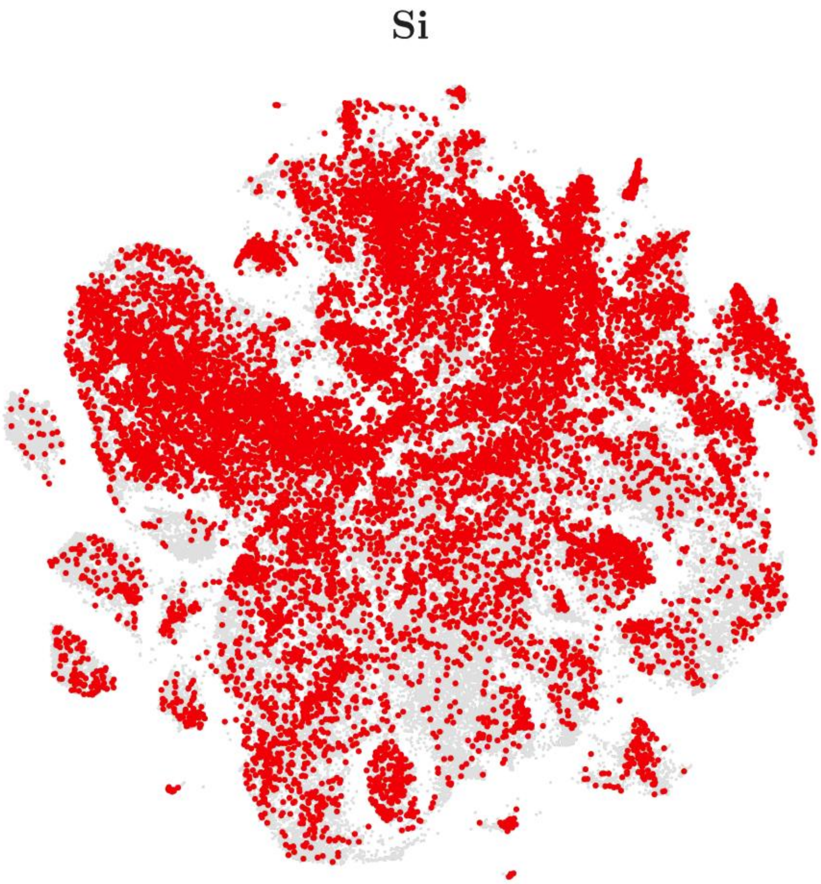

Figure S34. Caption Cluster plot for Phosphorus.

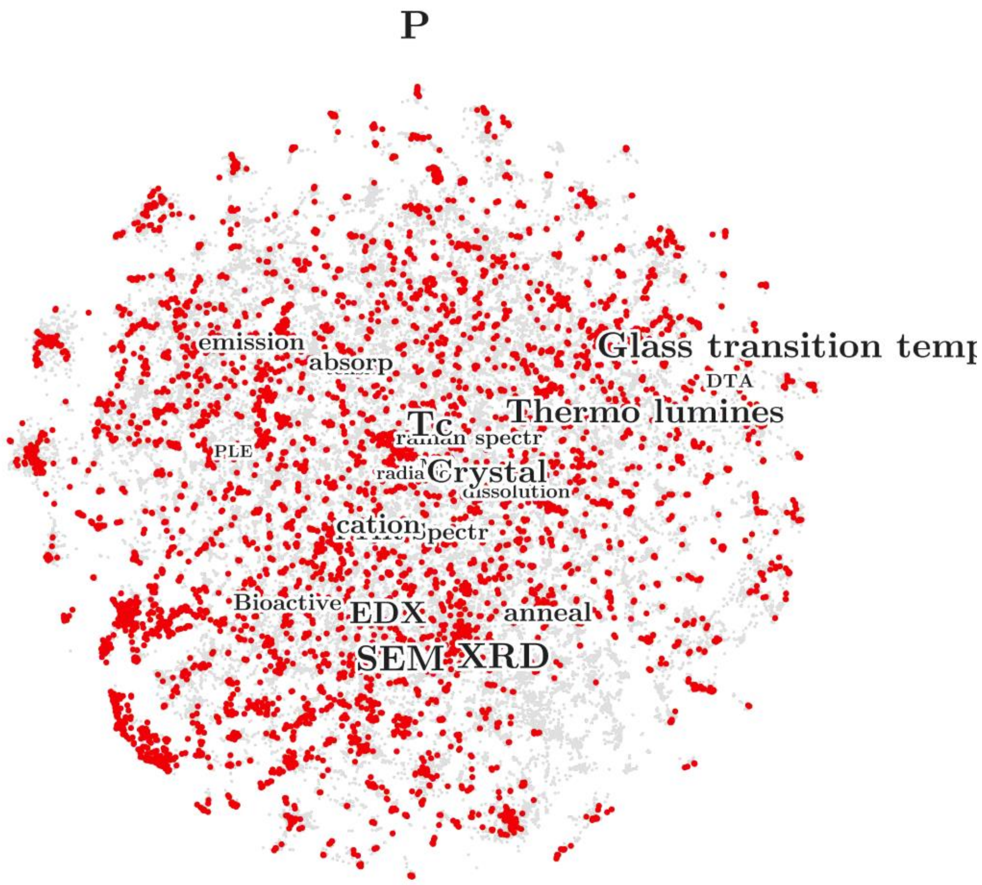

Figure S35. Latent Dirichlet Allocation plot for Phosphorus.

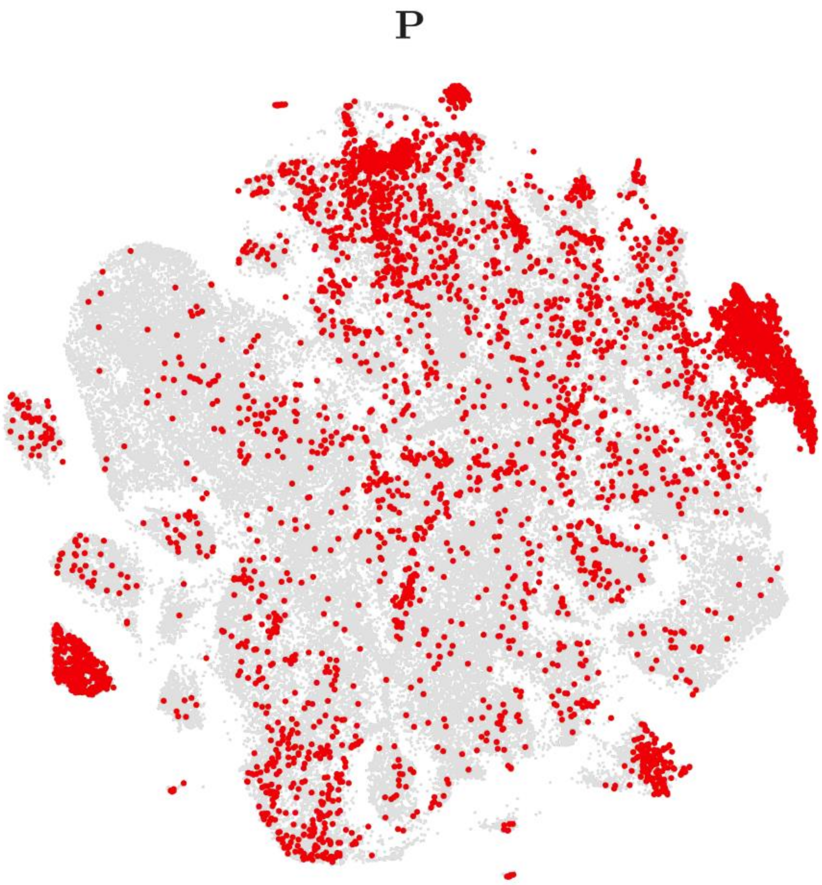

Figure S36. Caption Cluster plot for Sulfur.

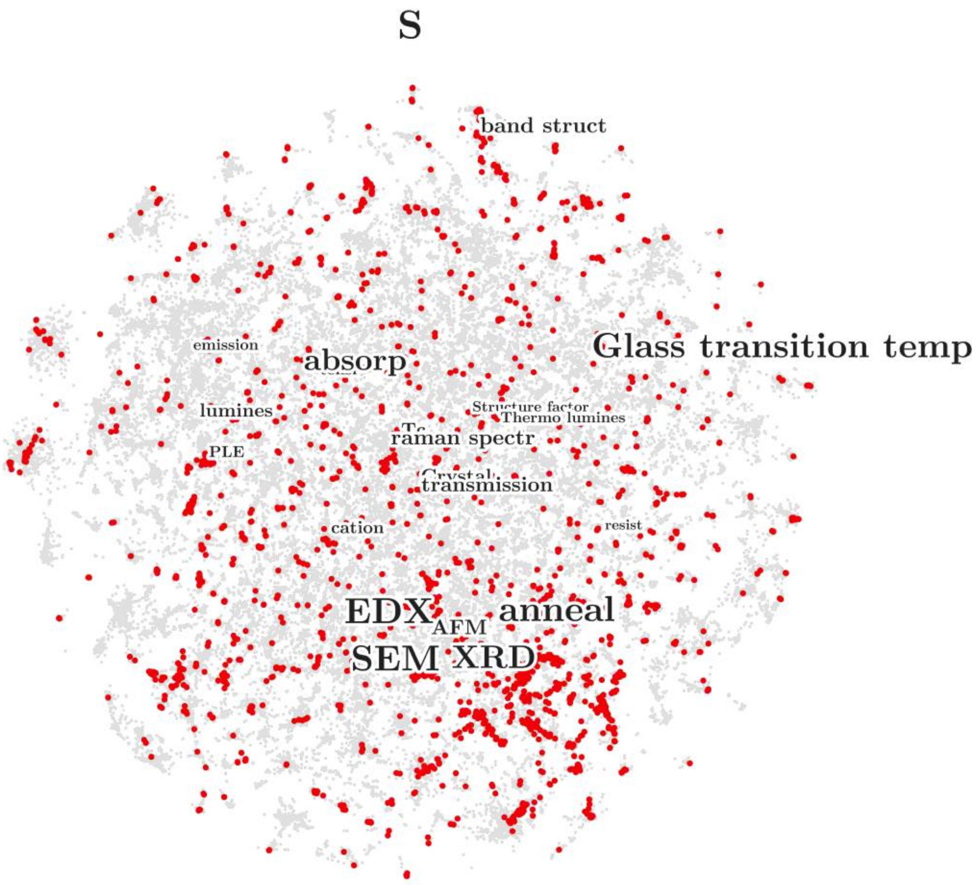

Figure S37. Latent Dirichlet Allocation plot for Sulfur.

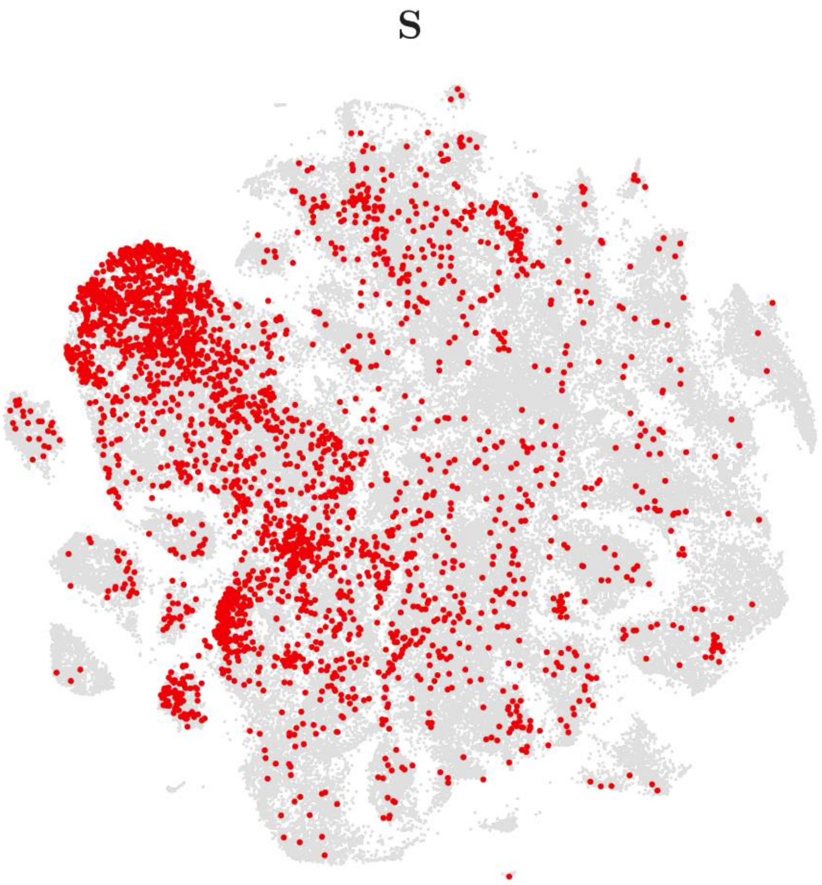

Figure S38. Caption Cluster plot for Chlorine.

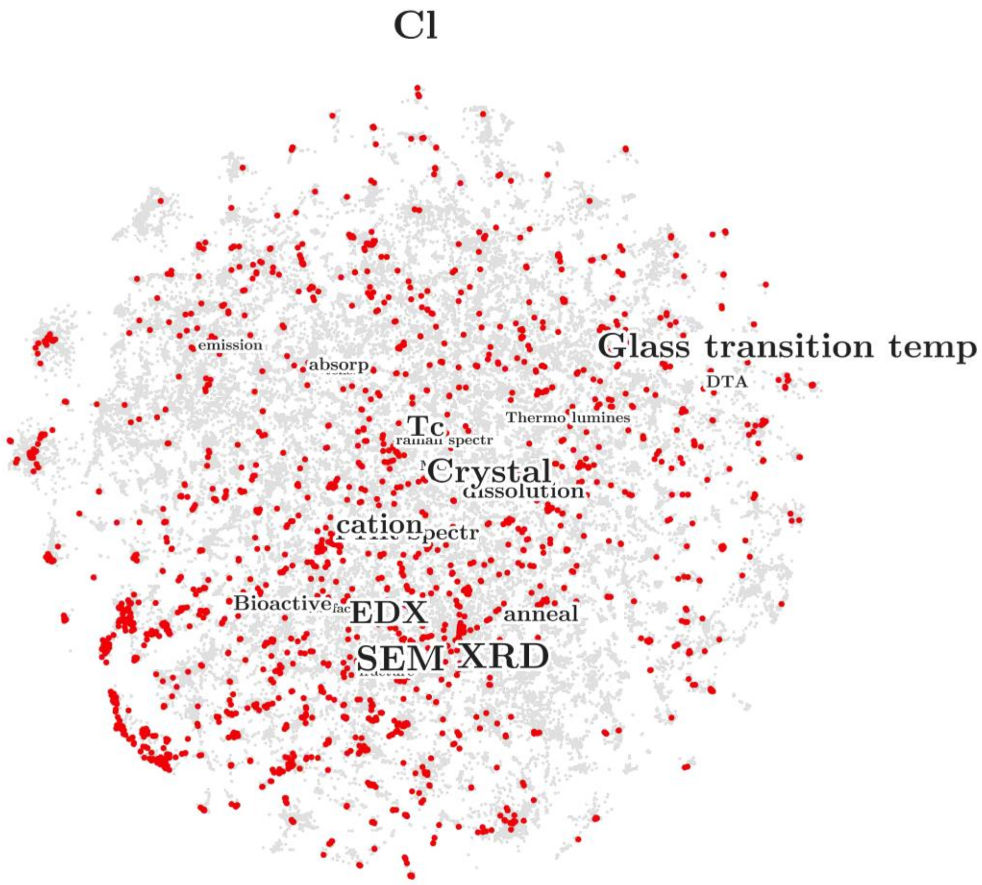

Figure S39. Latent Dirichlet Allocation plot for Chlorine.

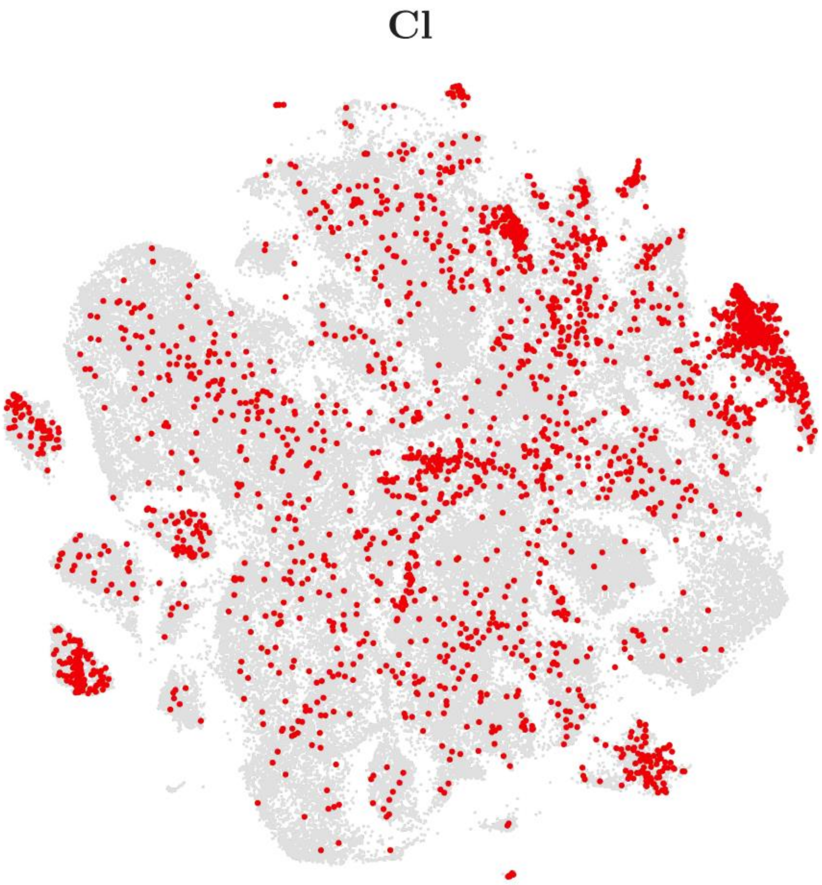

Figure S40. Caption Cluster plot for Argon.

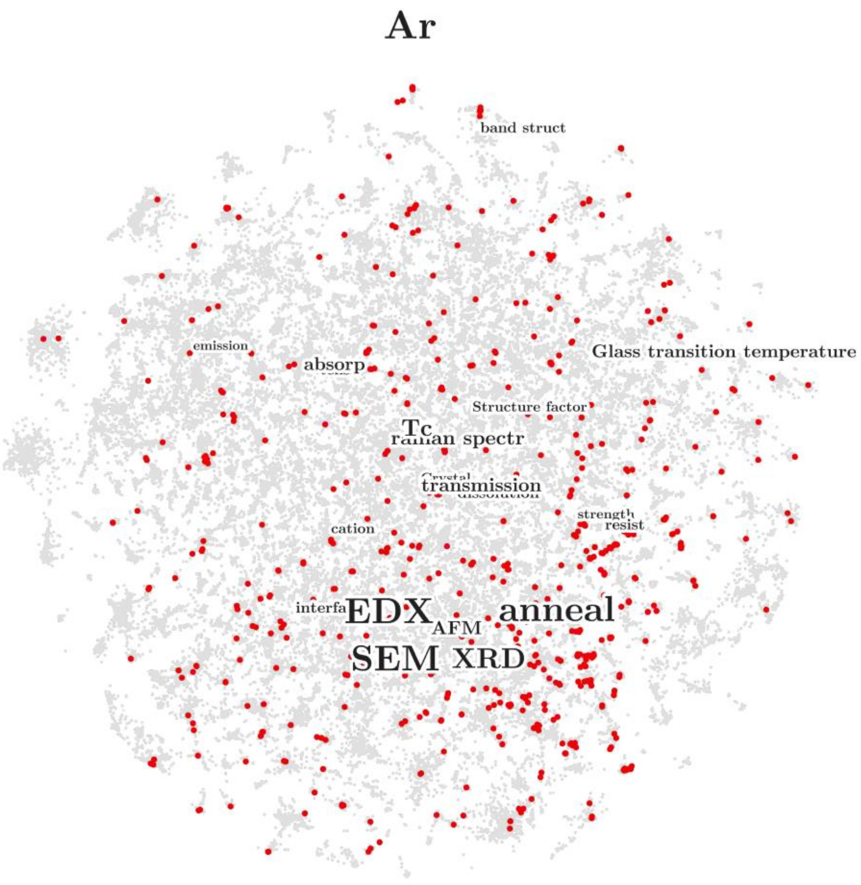

Figure S41. Latent Dirichlet Allocation plot for Argon.

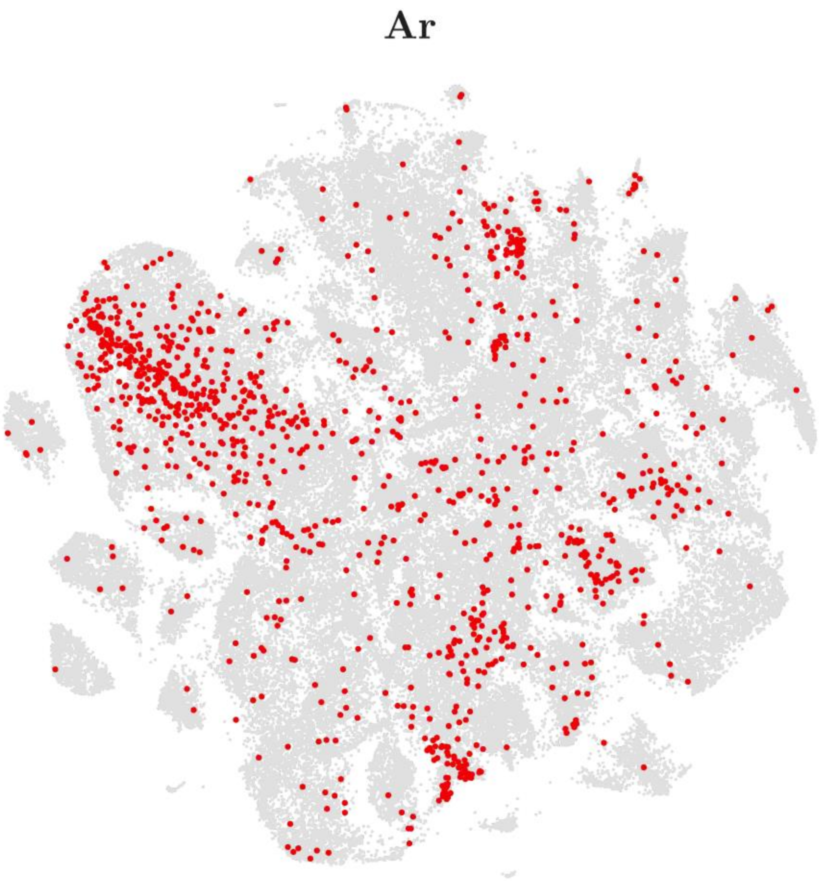

Figure S42. Caption Cluster plot for Potassium.

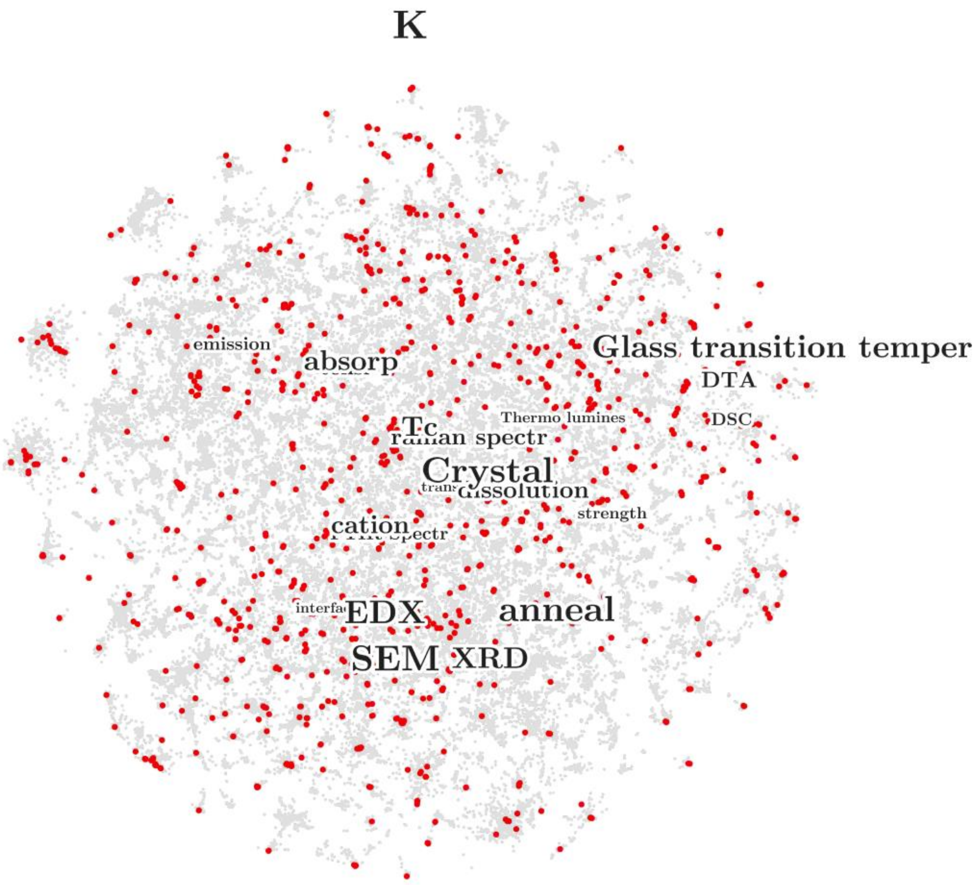

Figure S43. Latent Dirichlet Allocation plot for Potassium.

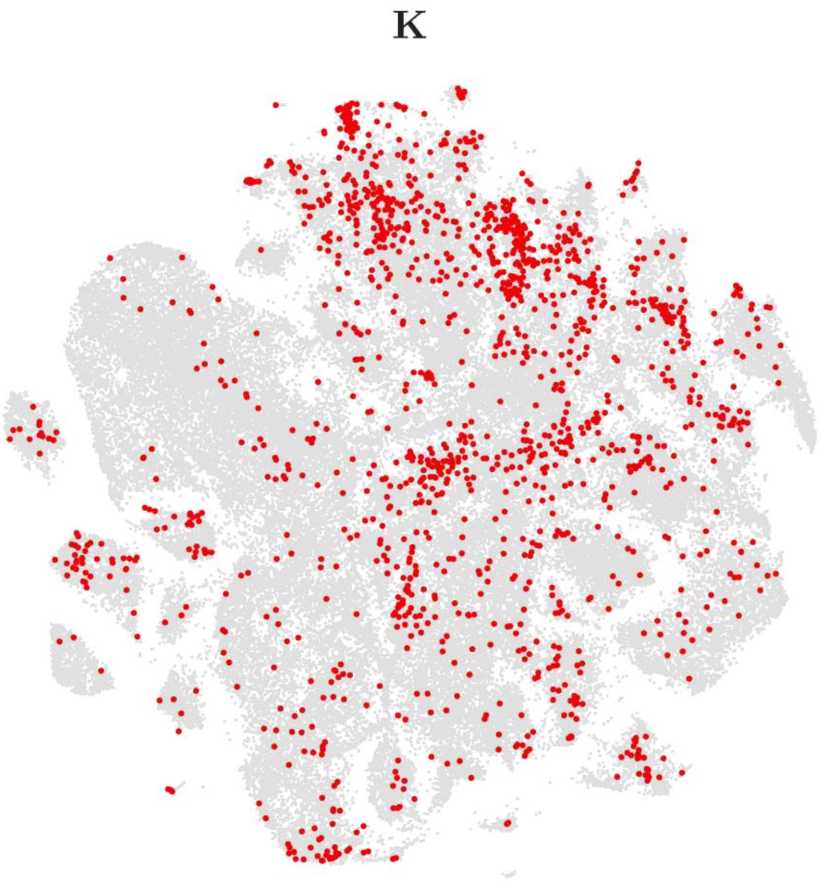

Figure S44. Caption Cluster plot for Calcium.

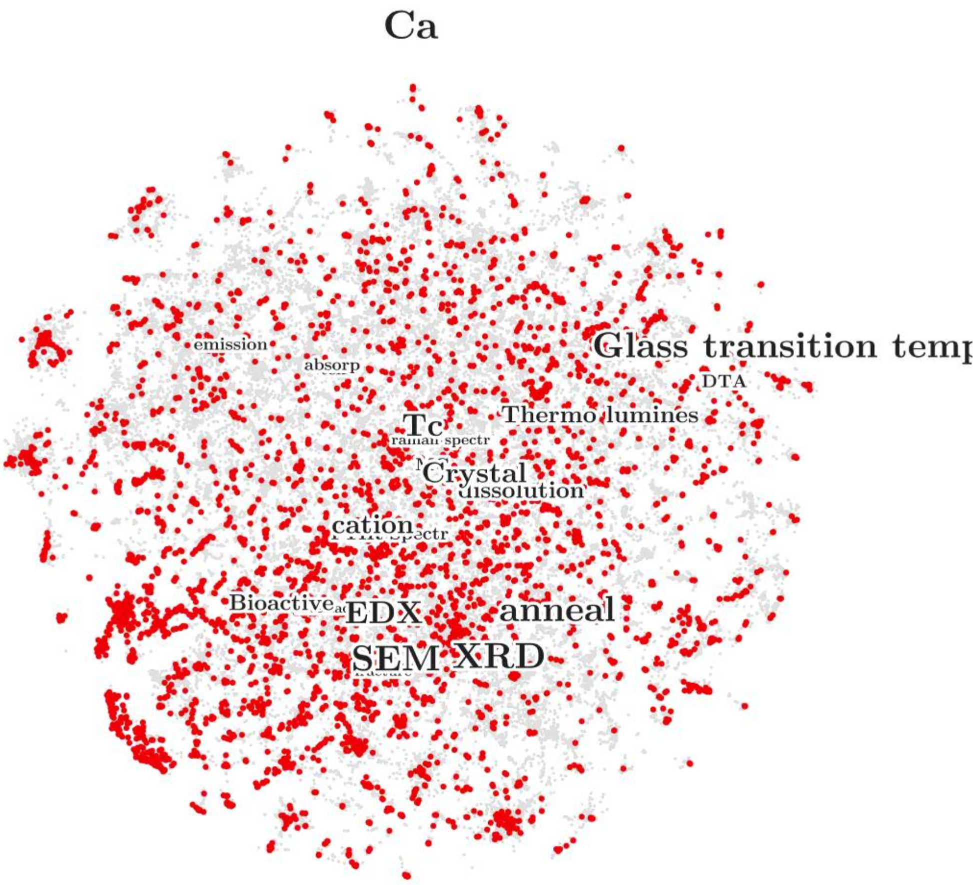

Figure S45. Latent Dirichlet Allocation plot for Calcium.

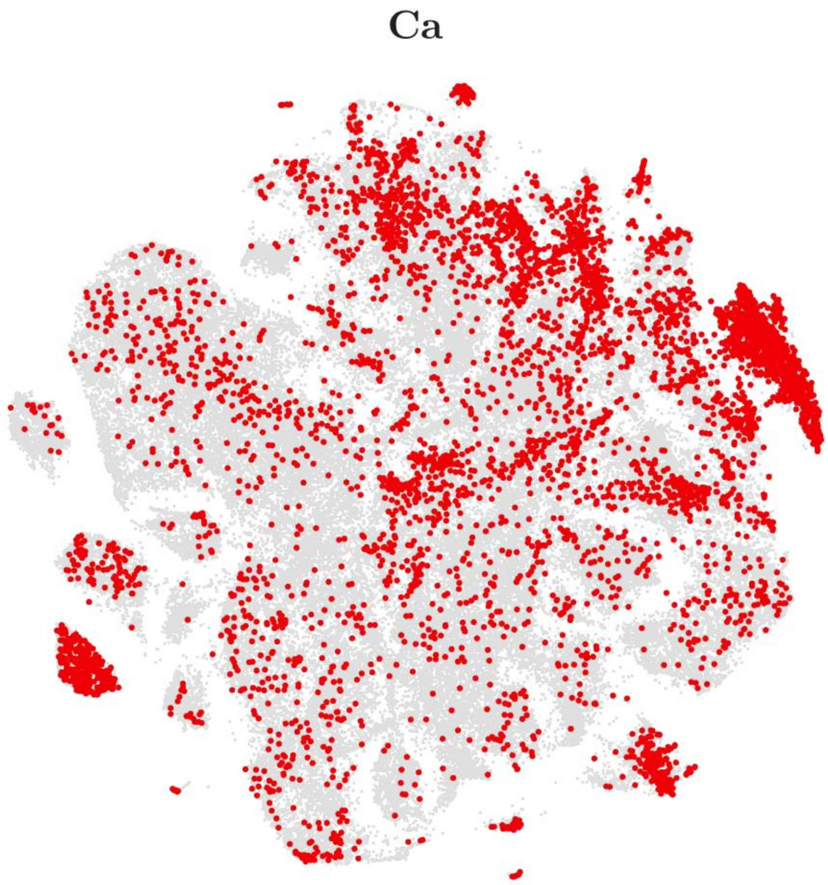

Figure S46. Caption Cluster plot for Scandium.

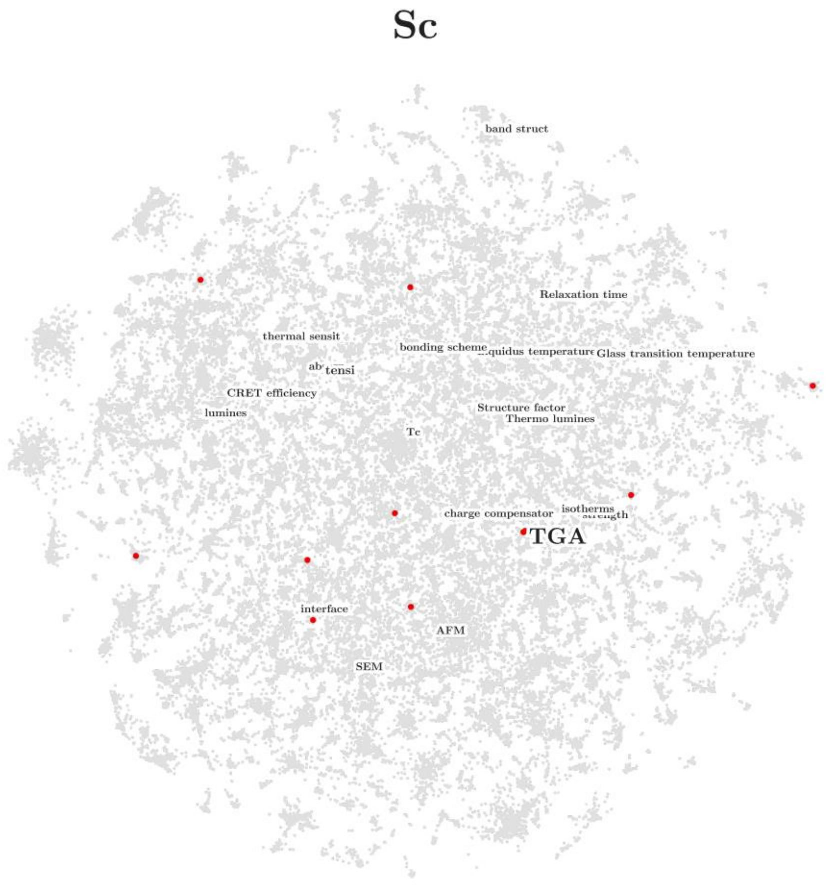

Figure S47. Latent Dirichlet Allocation plot for Scandium.

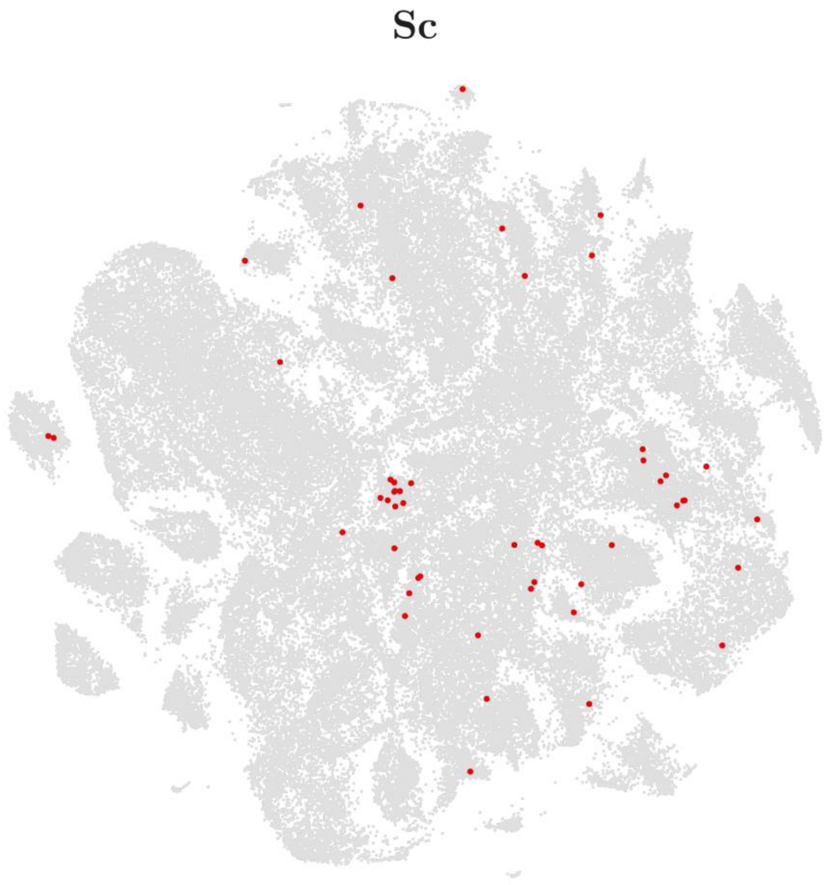

Figure S48. Caption Cluster plot for Titanium.

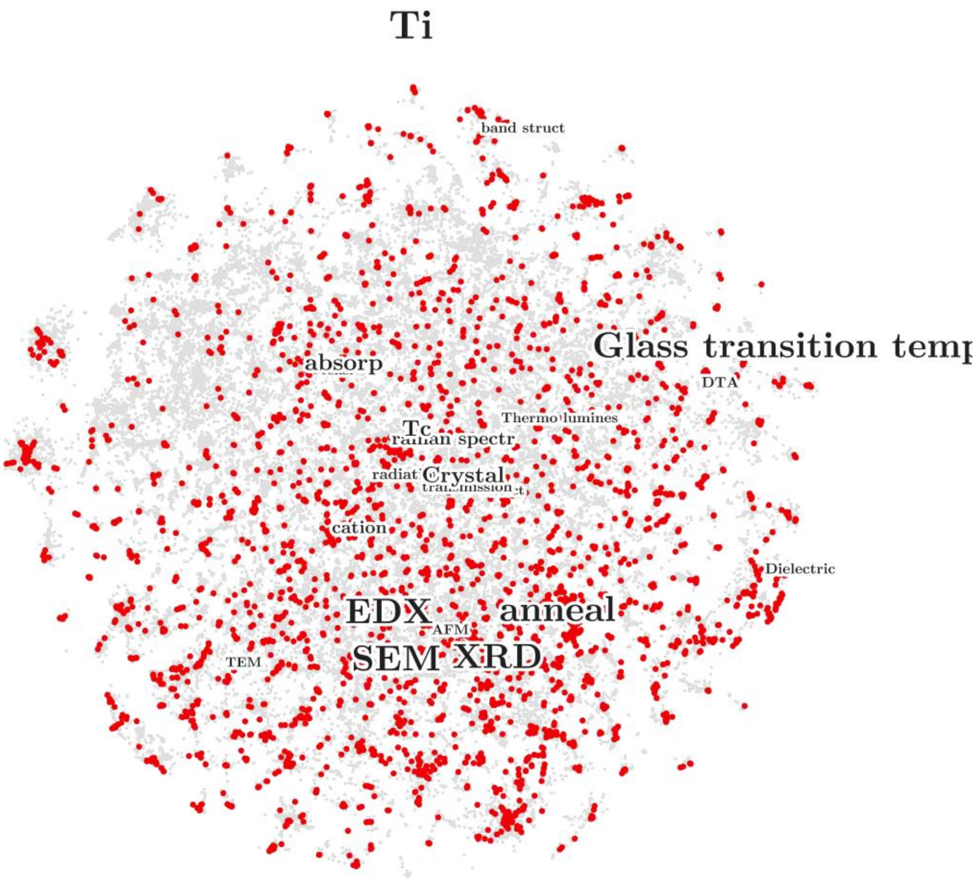

Figure S49. Latent Dirichlet Allocation plot for Titanium.

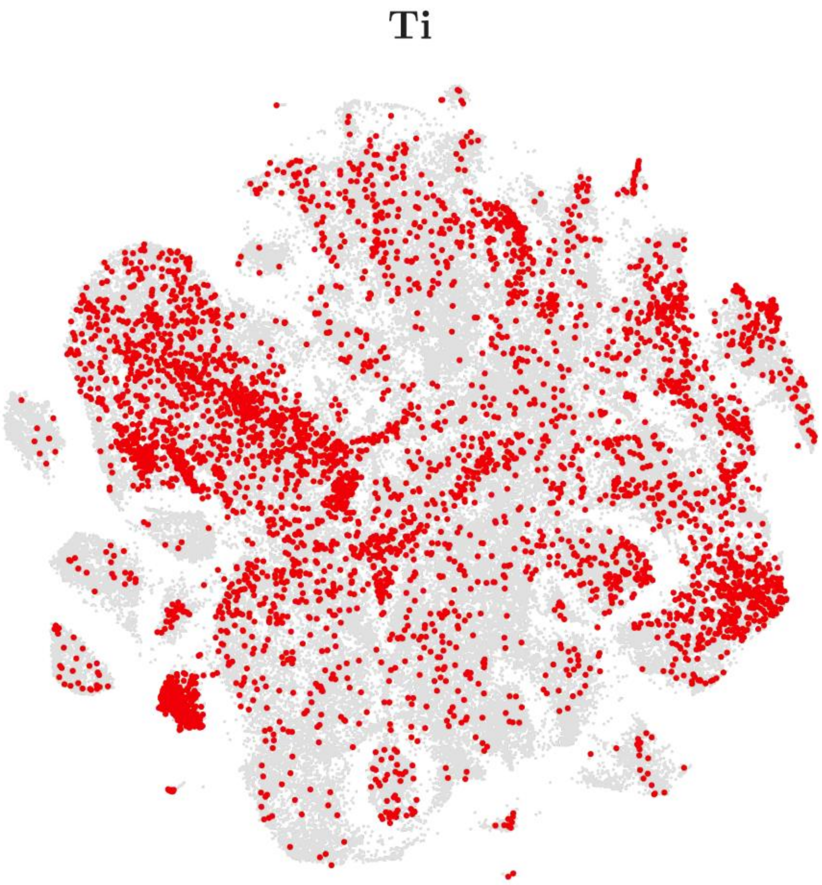

Figure S50. Caption Cluster plot for Vanadium.

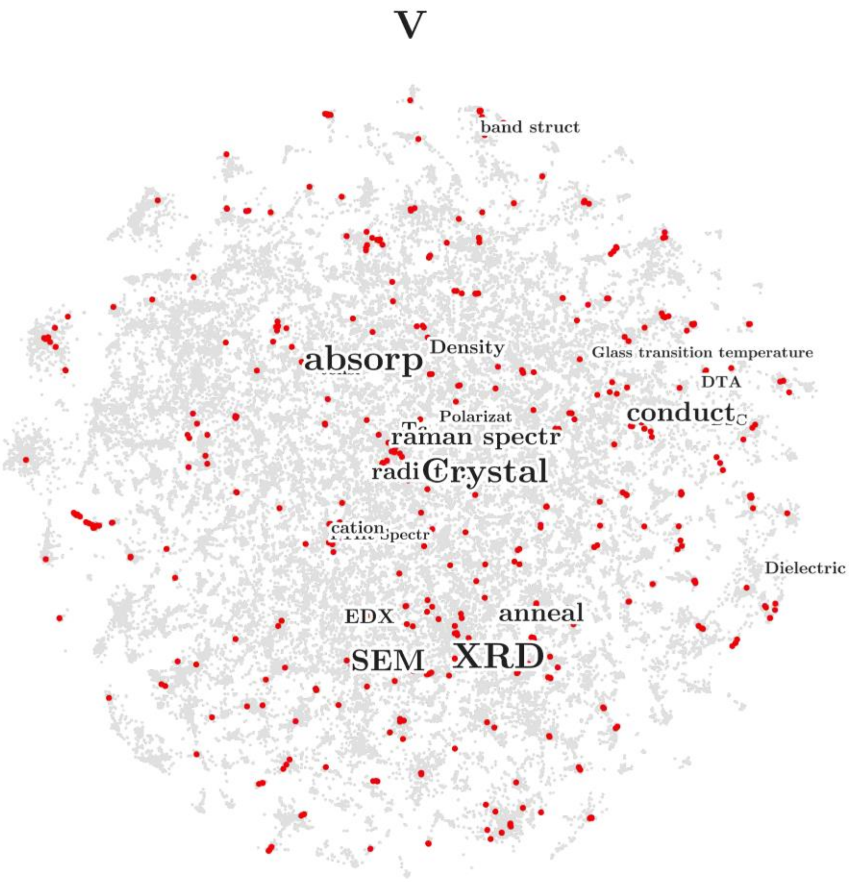

Figure S51. Latent Dirichlet Allocation plot for Vanadium.

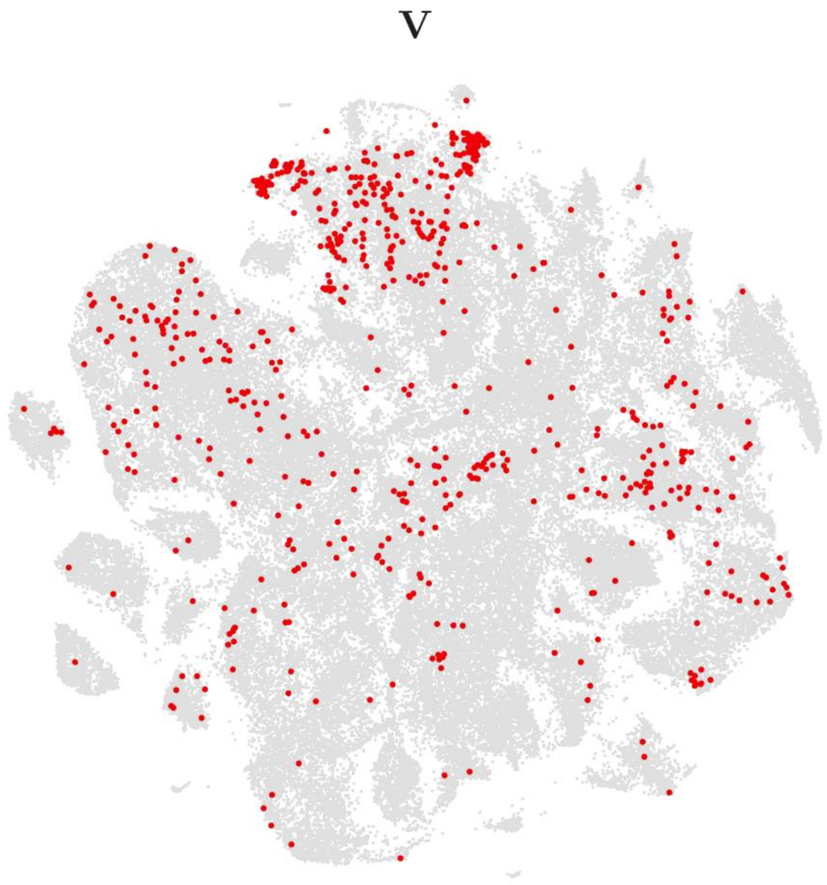

Figure S52. Caption Cluster plot for Chromium.

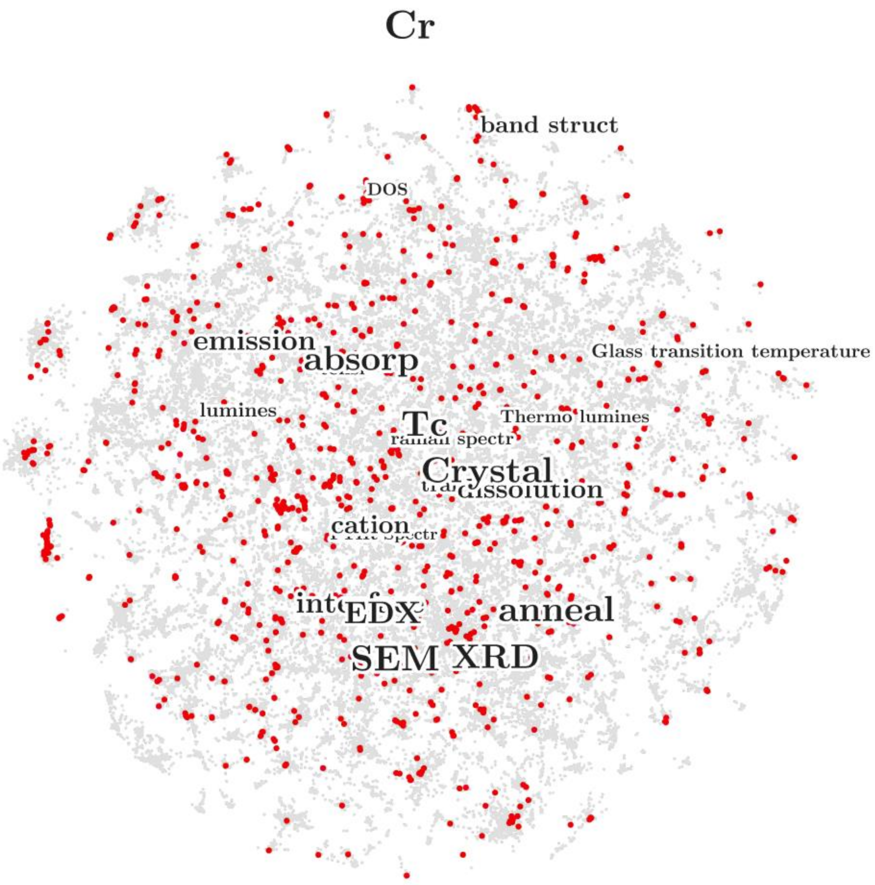

Figure S53. Latent Dirichlet Allocation plot for Chromium.

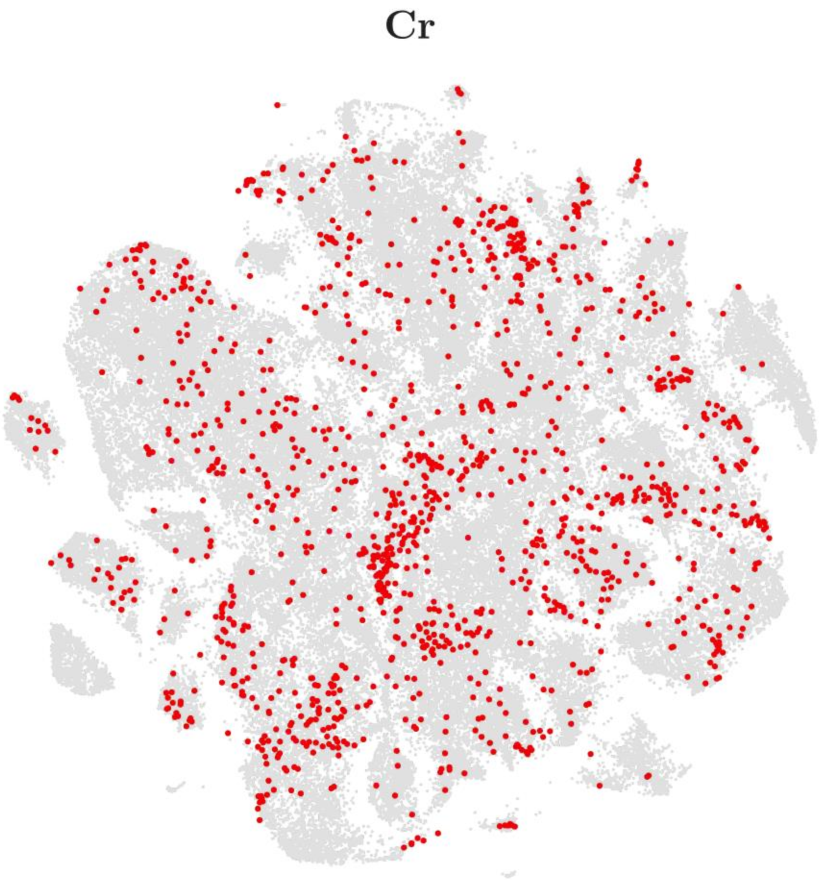

Figure S54. Caption Cluster plot for Manganese.

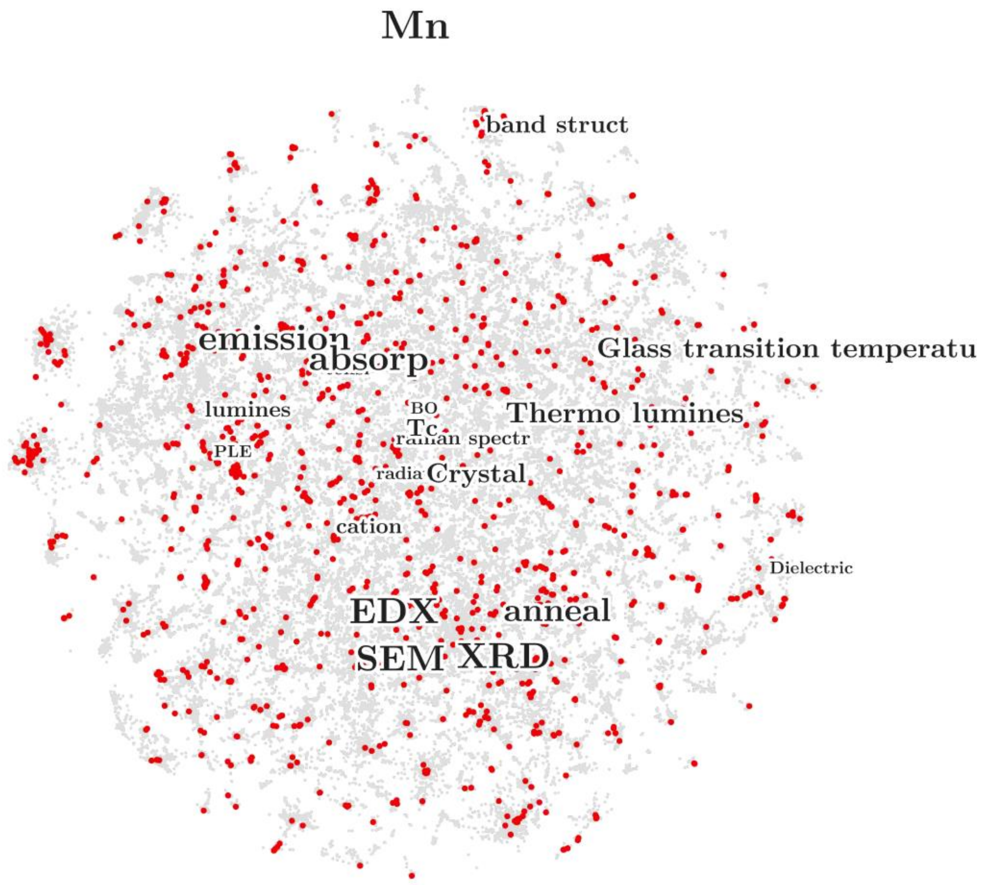

Figure S55. Latent Dirichlet Allocation plot for Manganese.

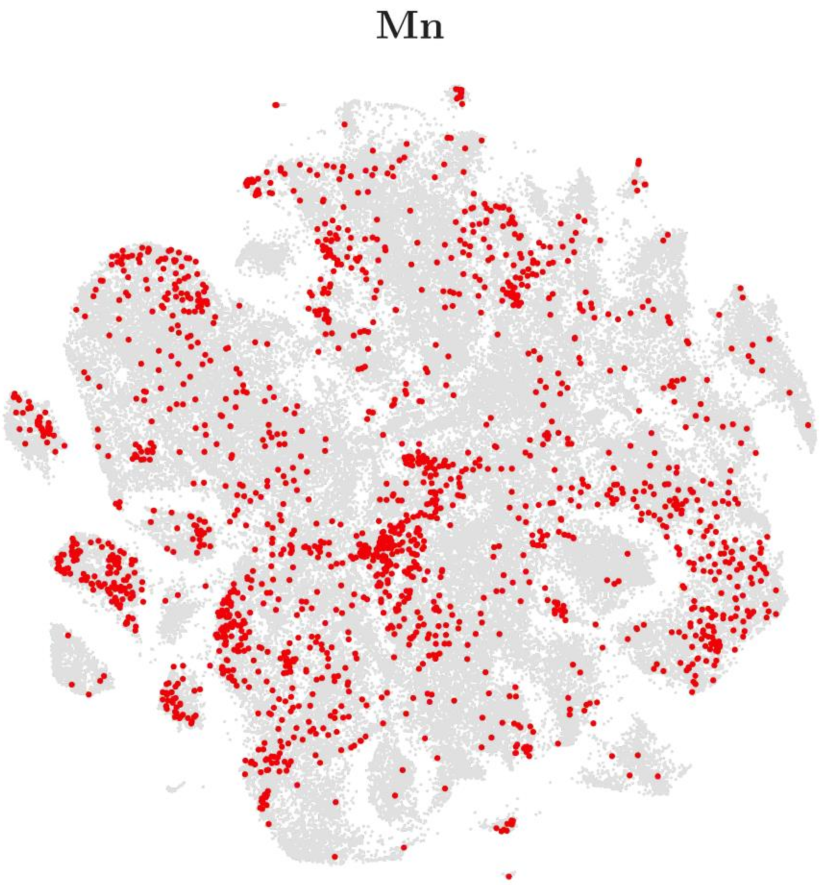

Figure S56. Caption Cluster plot for Iron.

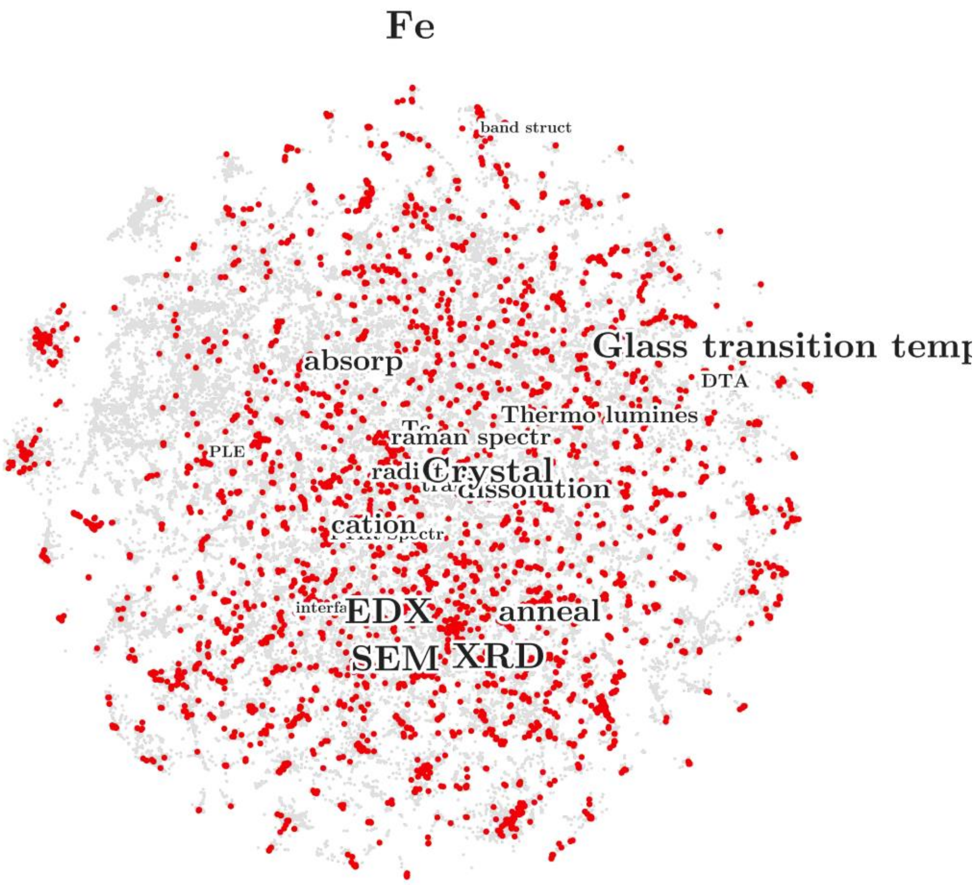

Figure S57. Latent Dirichlet Allocation plot for Iron.

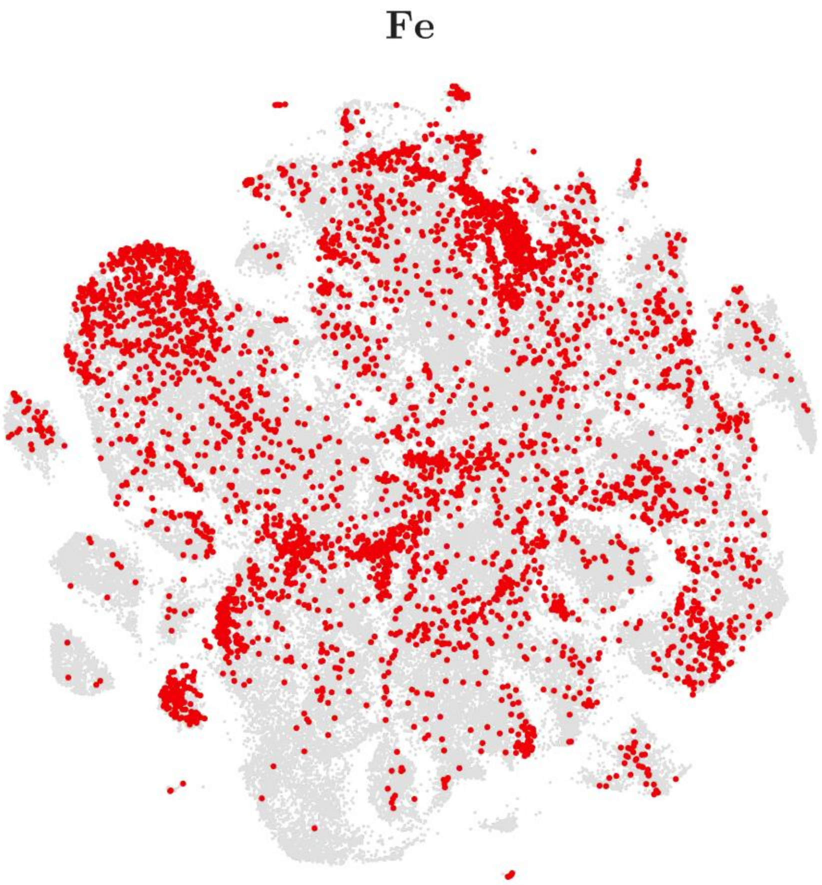

Figure S58. Caption Cluster plot for Cobalt.

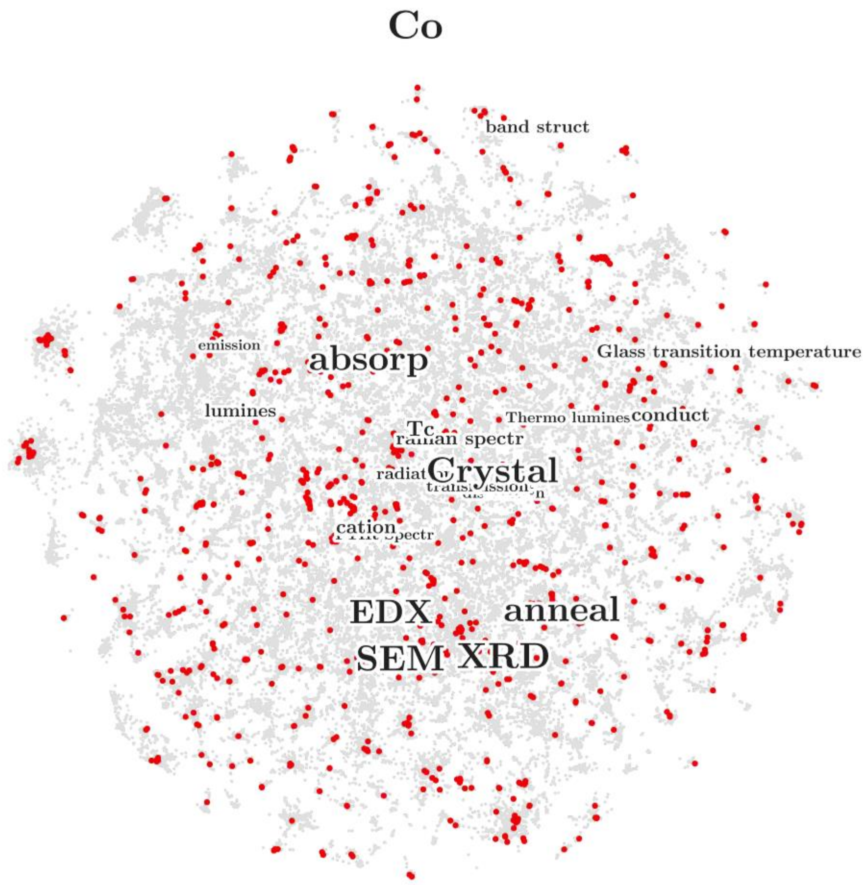

Figure S59. Latent Dirichlet Allocation plot for Cobalt.

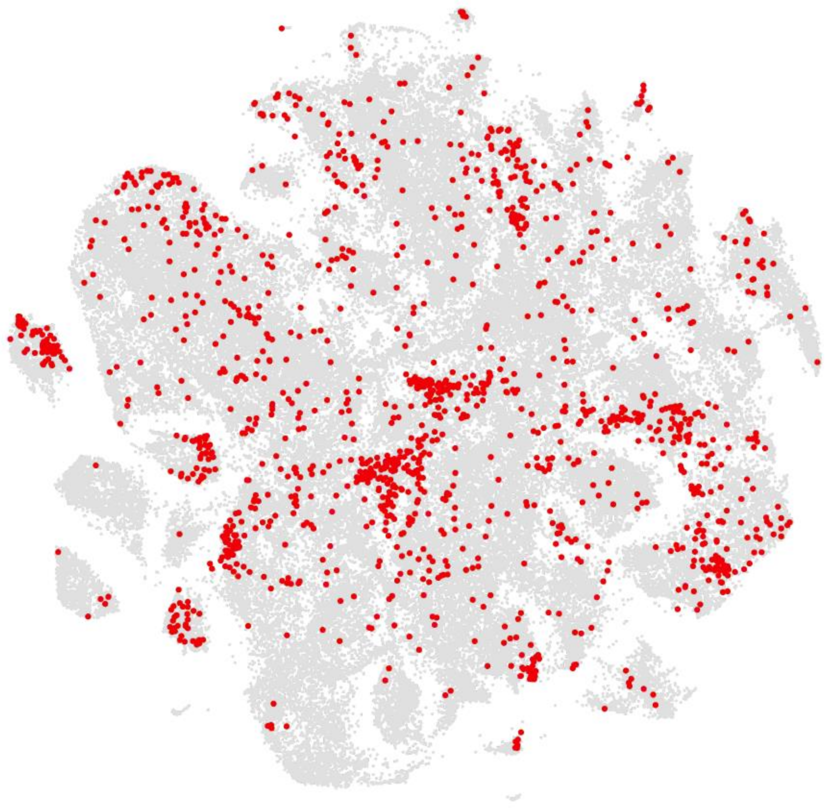

Figure S60. Caption Cluster plot for Nickel.

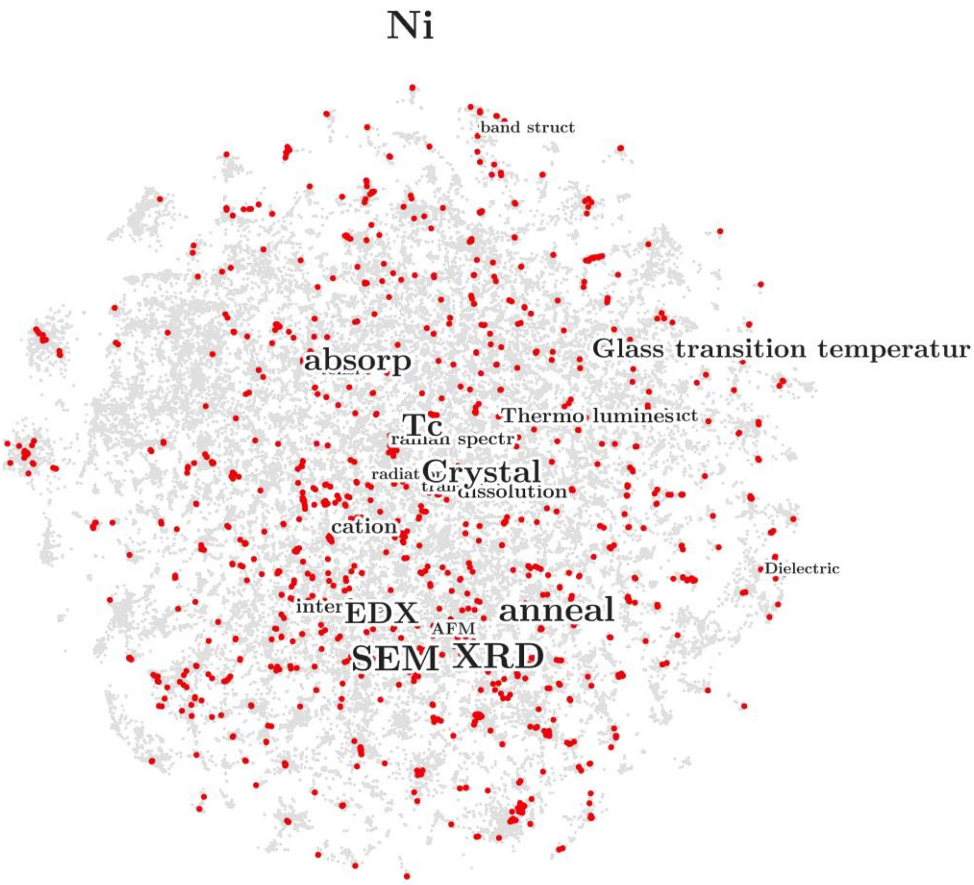

Figure S61. Latent Dirichlet Allocation plot for Nickel.

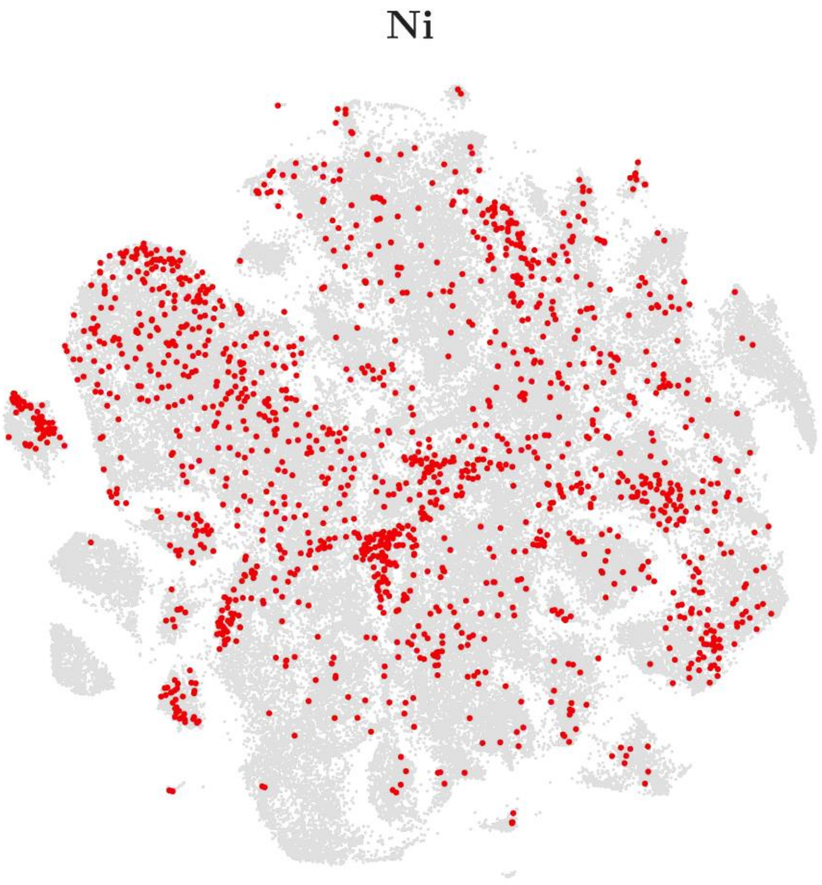

Figure S62. Caption Cluster plot for Copper.

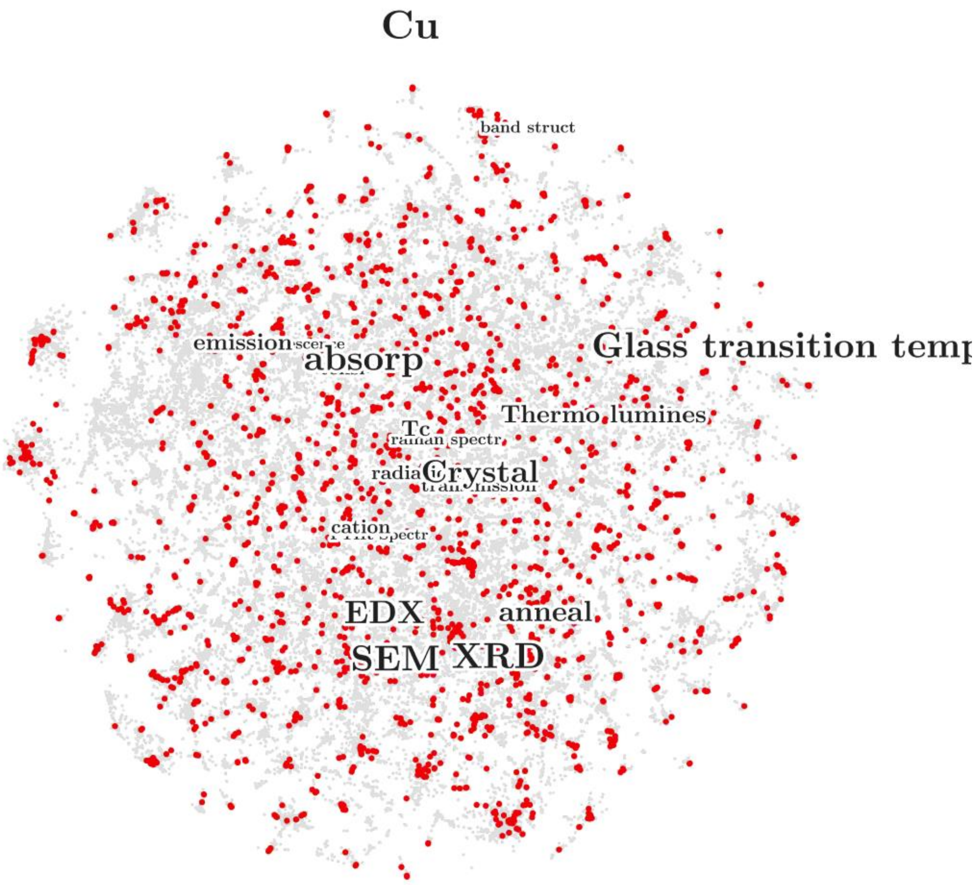

Figure S63. Latent Dirichlet Allocation plot for Copper.

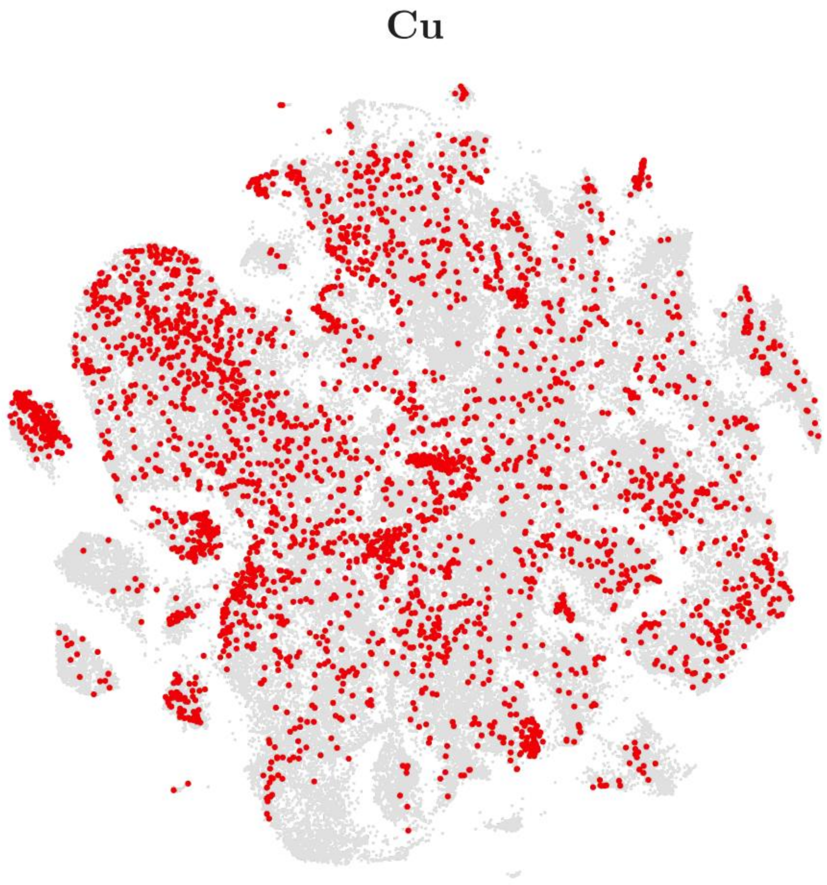

Figure S64. Caption Cluster plot for Zinc.

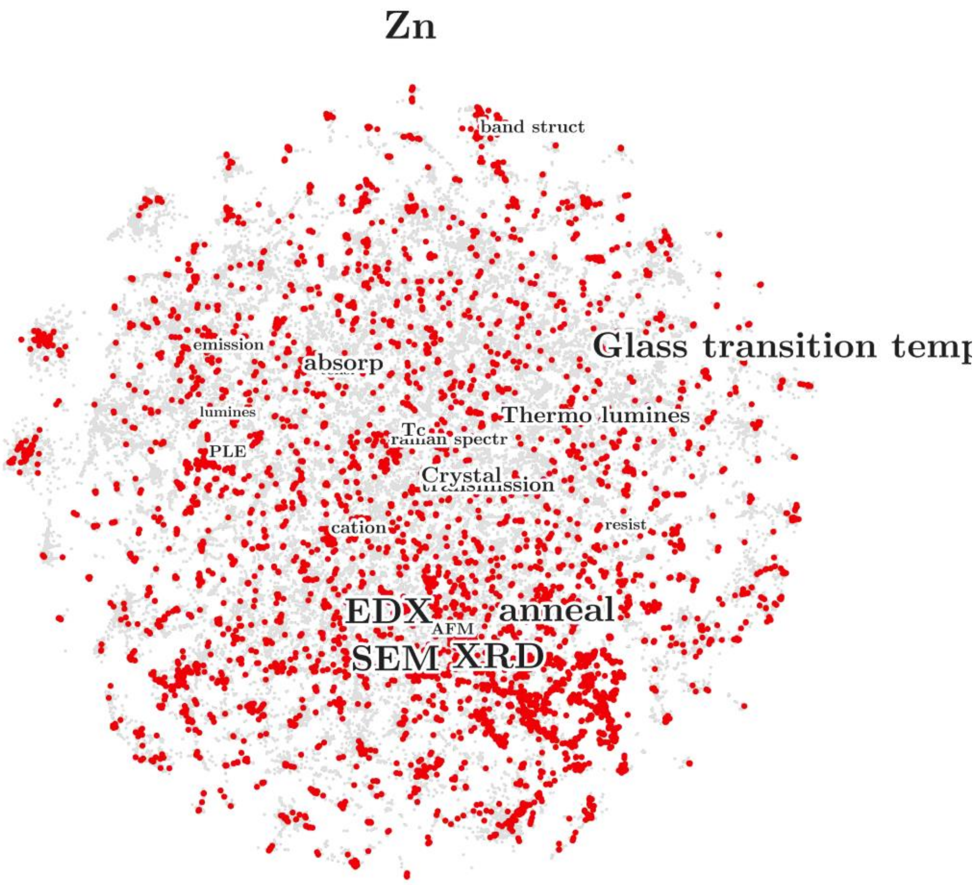

Figure S65. Latent Dirichlet Allocation plot for Zinc.

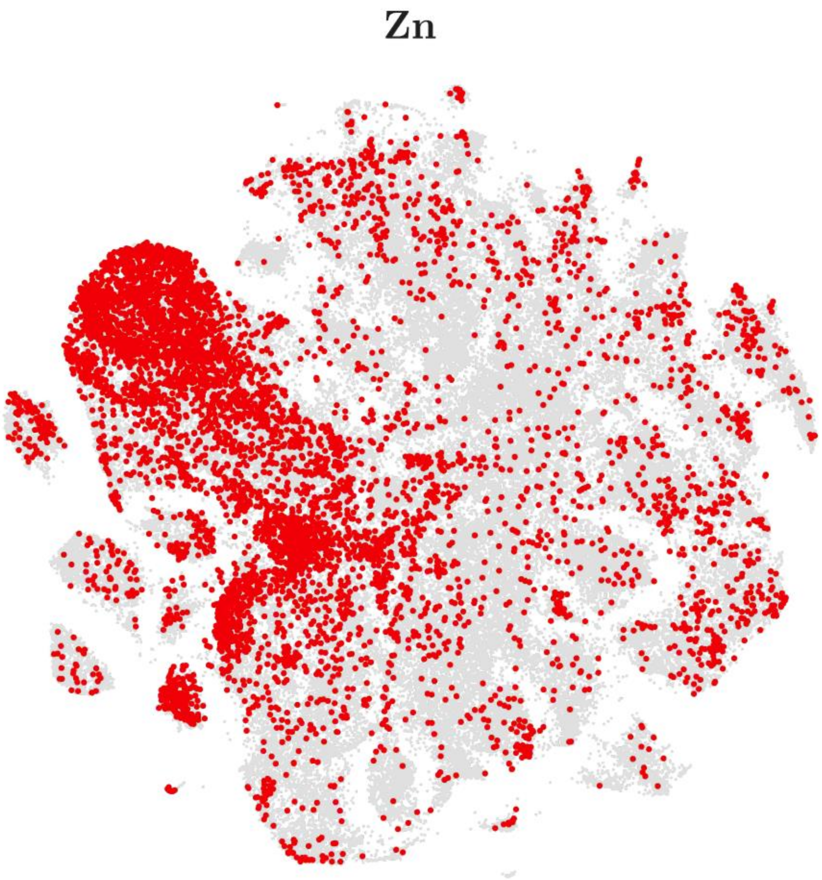

Figure S66. Caption Cluster plot for Gallium.

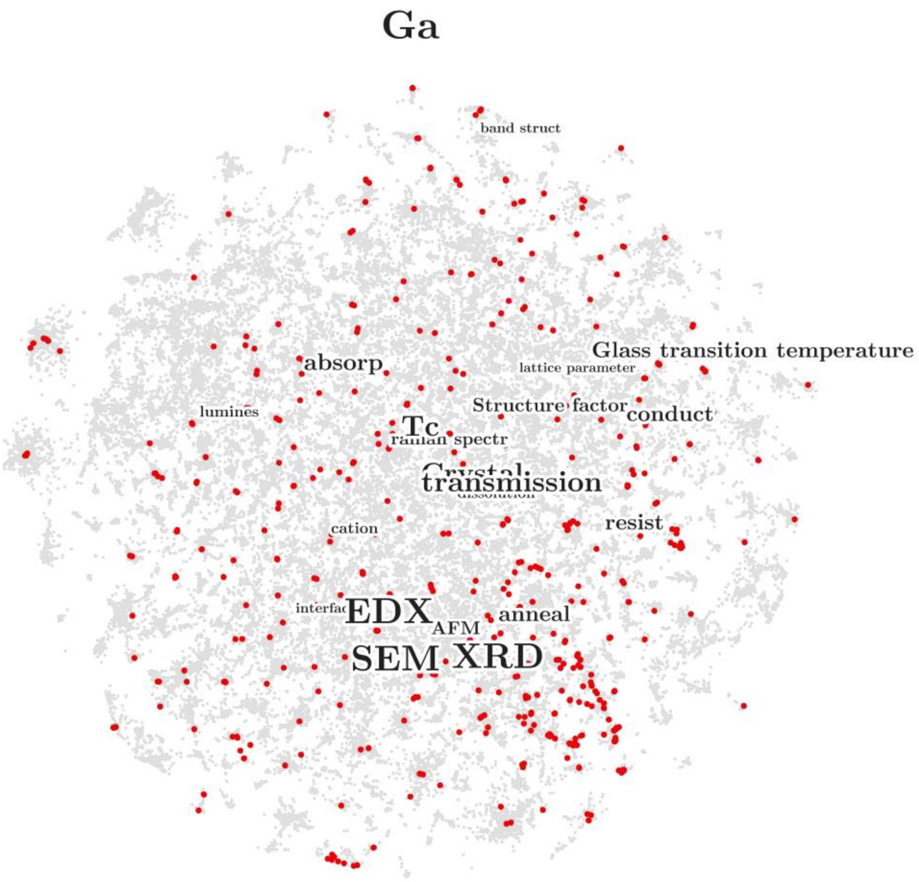

Figure S67. Latent Dirichlet Allocation plot for Gallium.

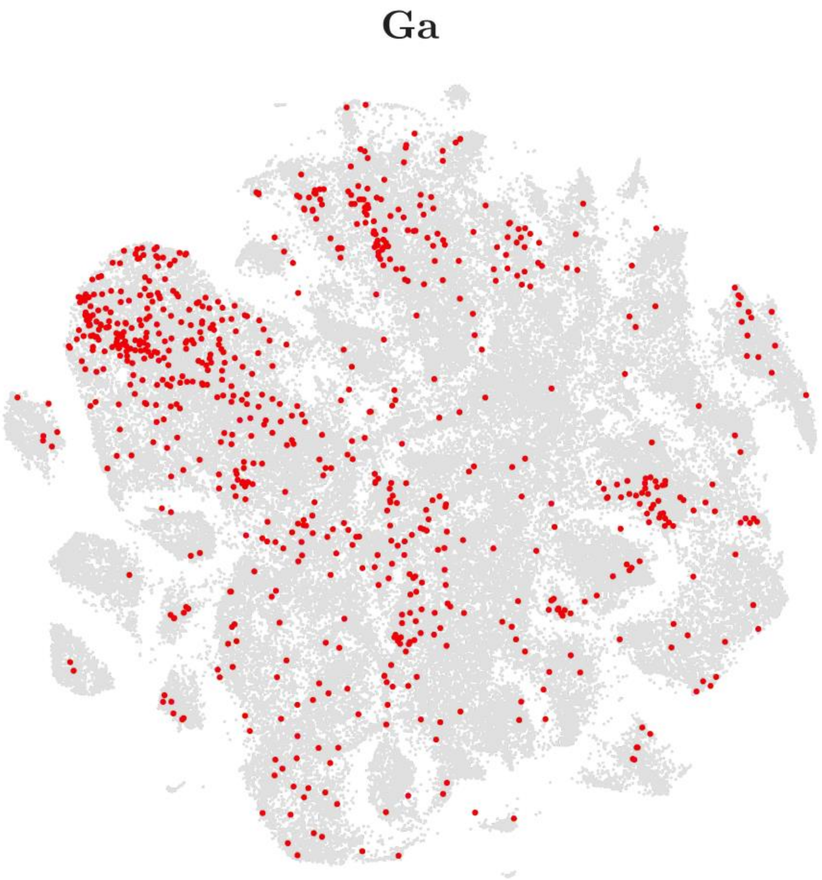

Figure S68. Caption Cluster plot for Germanium.

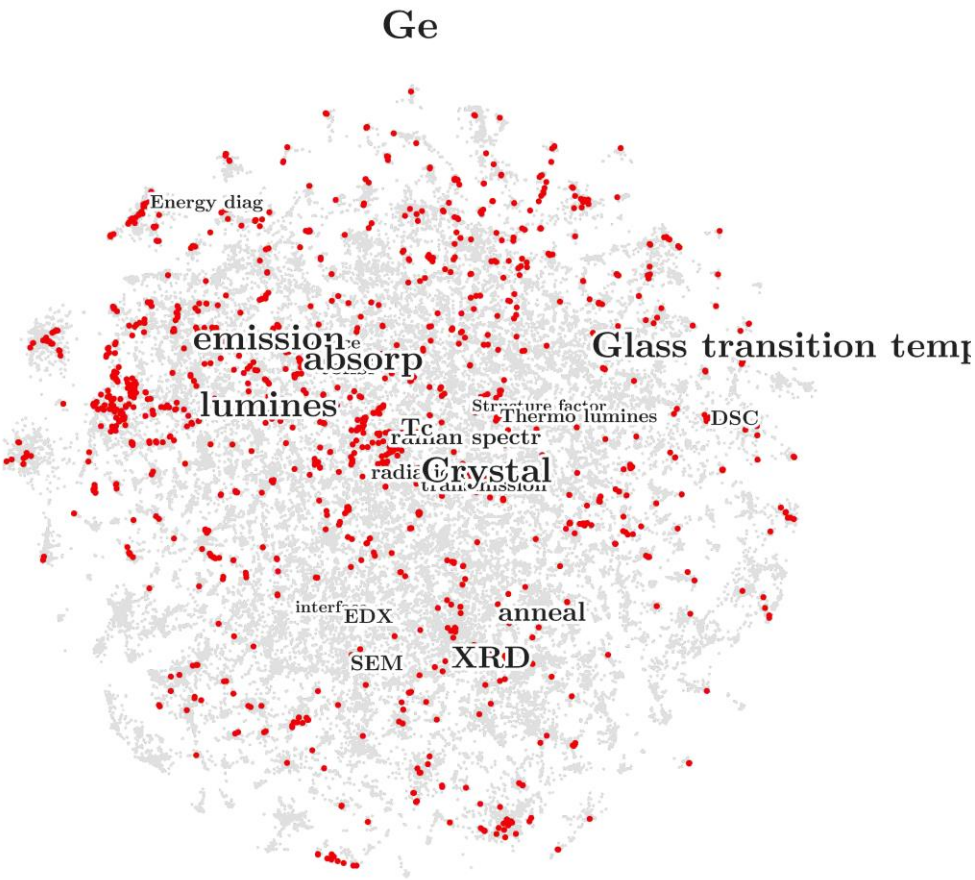

Figure S69. Latent Dirichlet Allocation plot for Germanium.

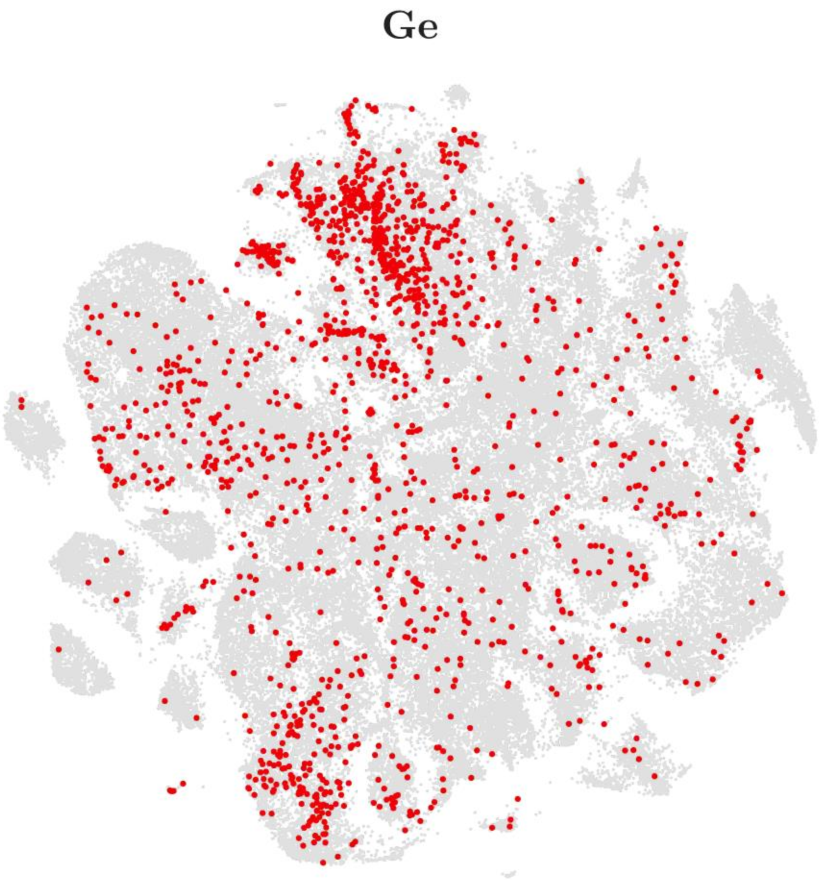

Figure S70. Caption Cluster plot for Arsenic.

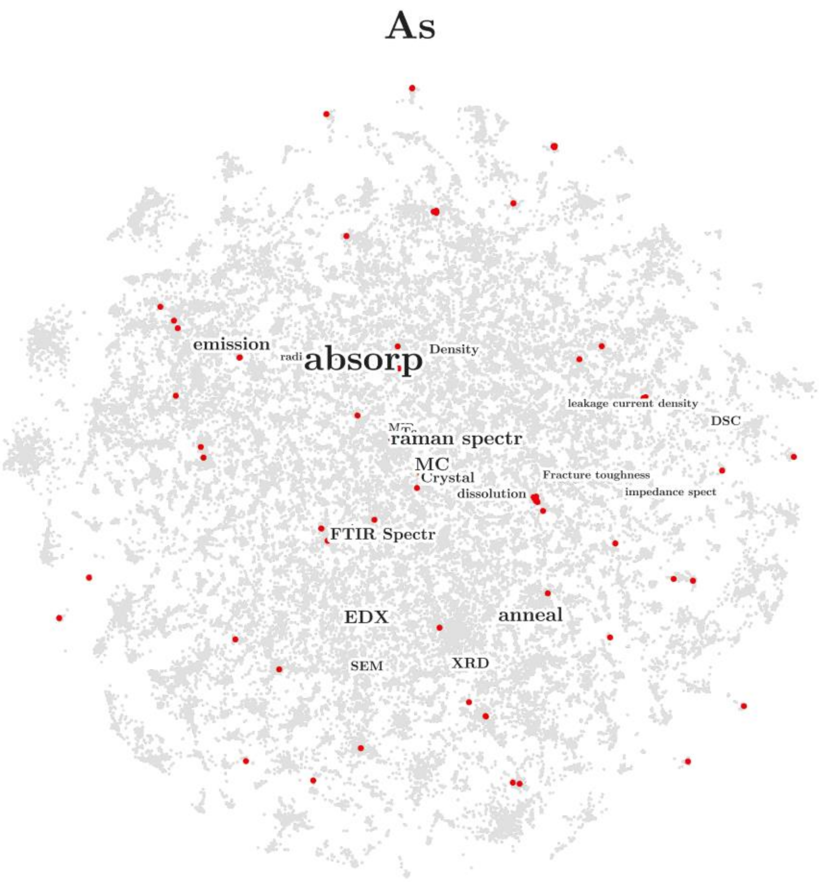

Figure S71. Latent Dirichlet Allocation plot for Arsenic.

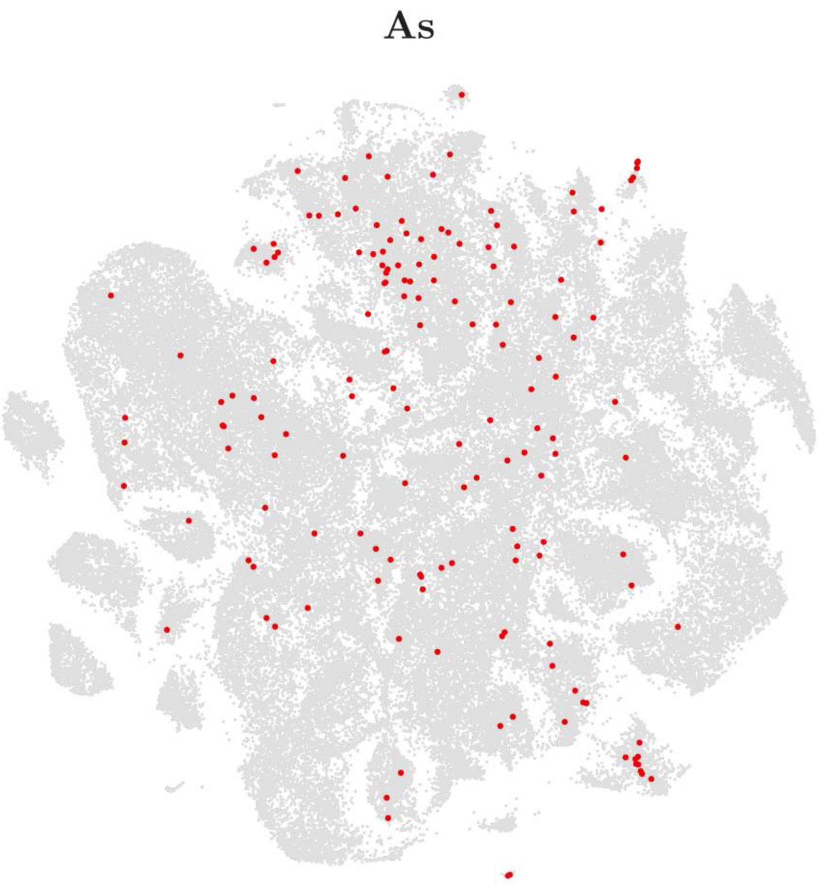

Figure S72. Caption Cluster plot for Selenium.

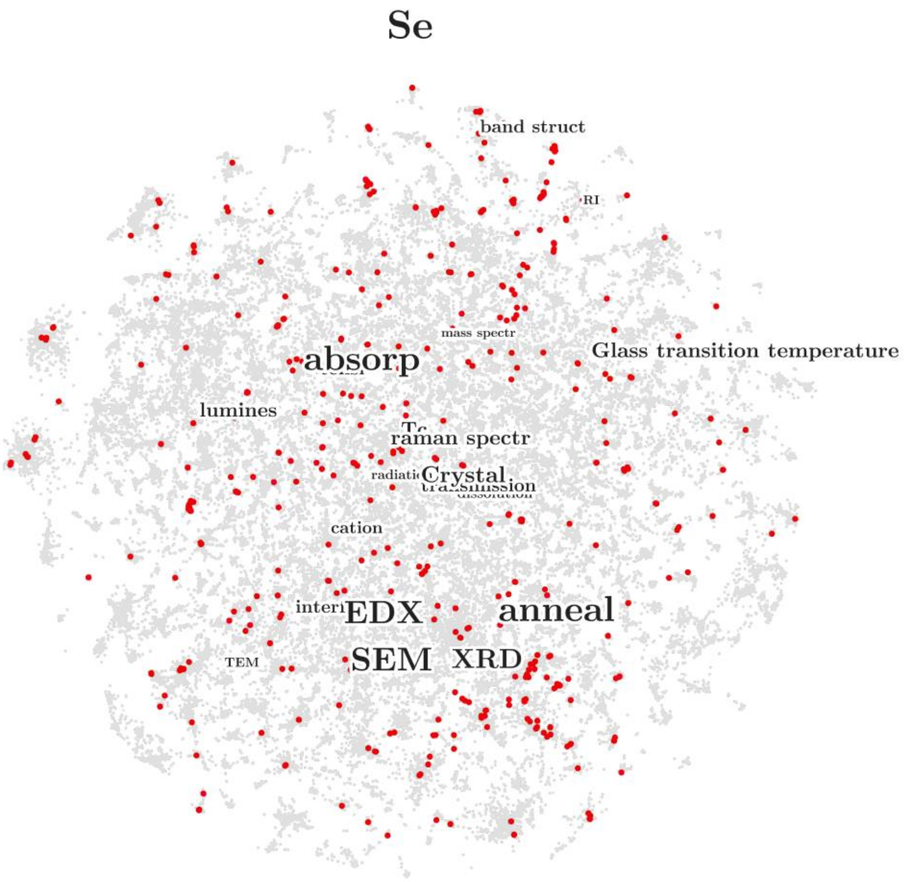

Figure S73. Latent Dirichlet Allocation plot for Selenium.

.

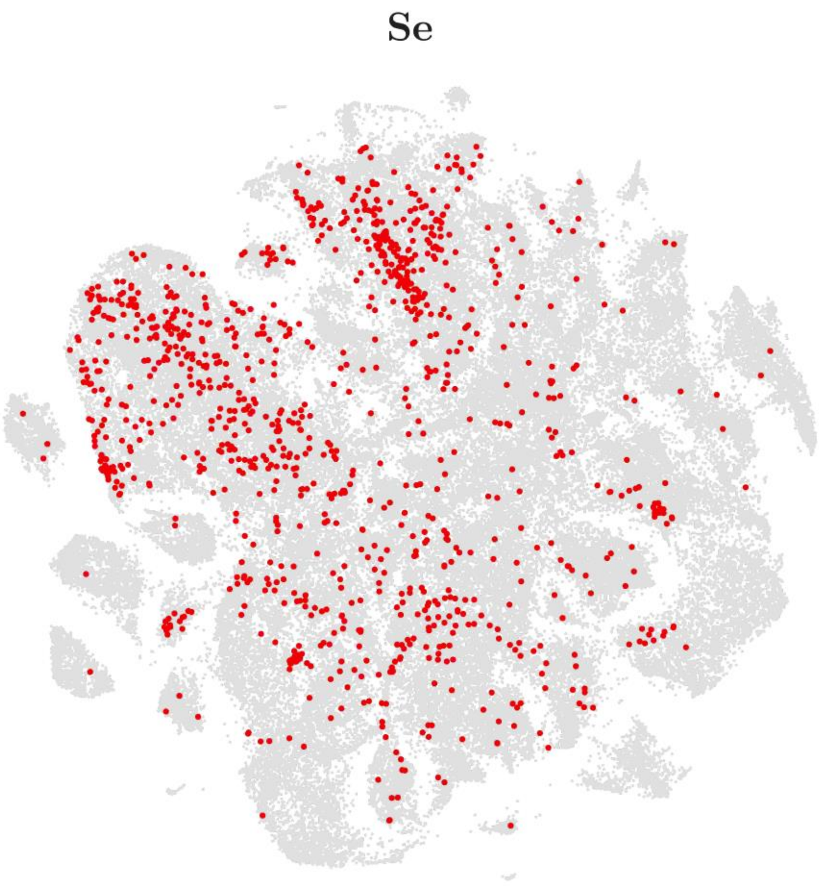

Figure S74. Caption Cluster plot for Bromine.

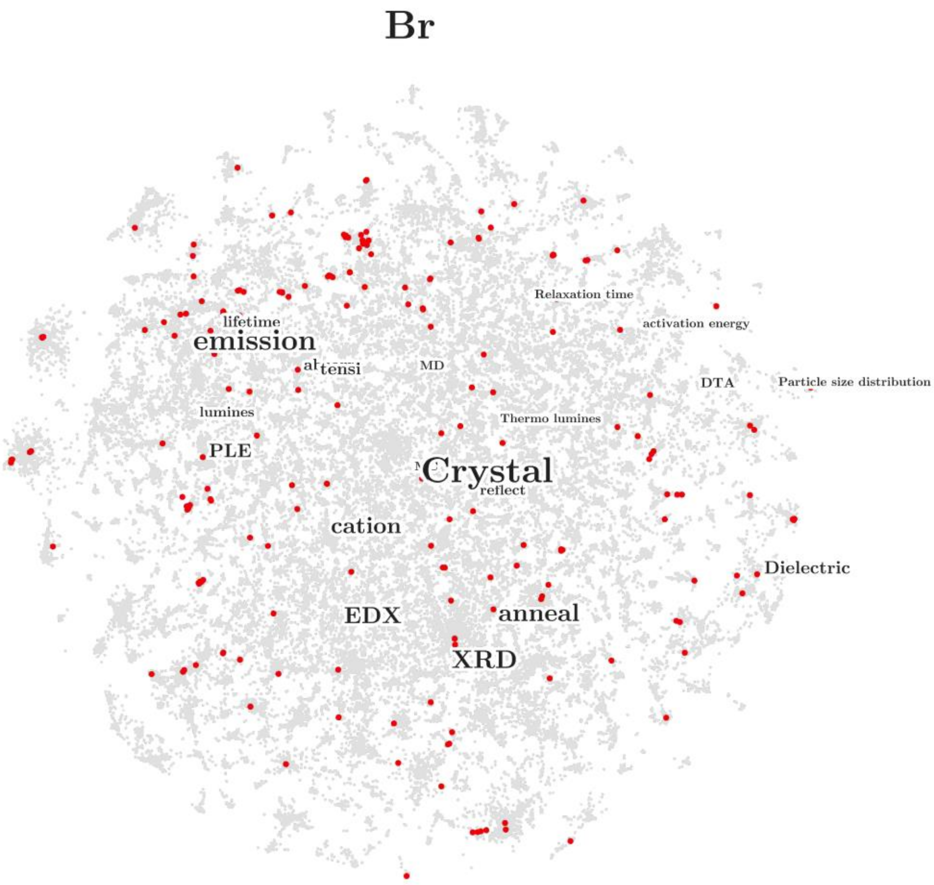

Figure S75. Latent Dirichlet Allocation plot for Bromine.

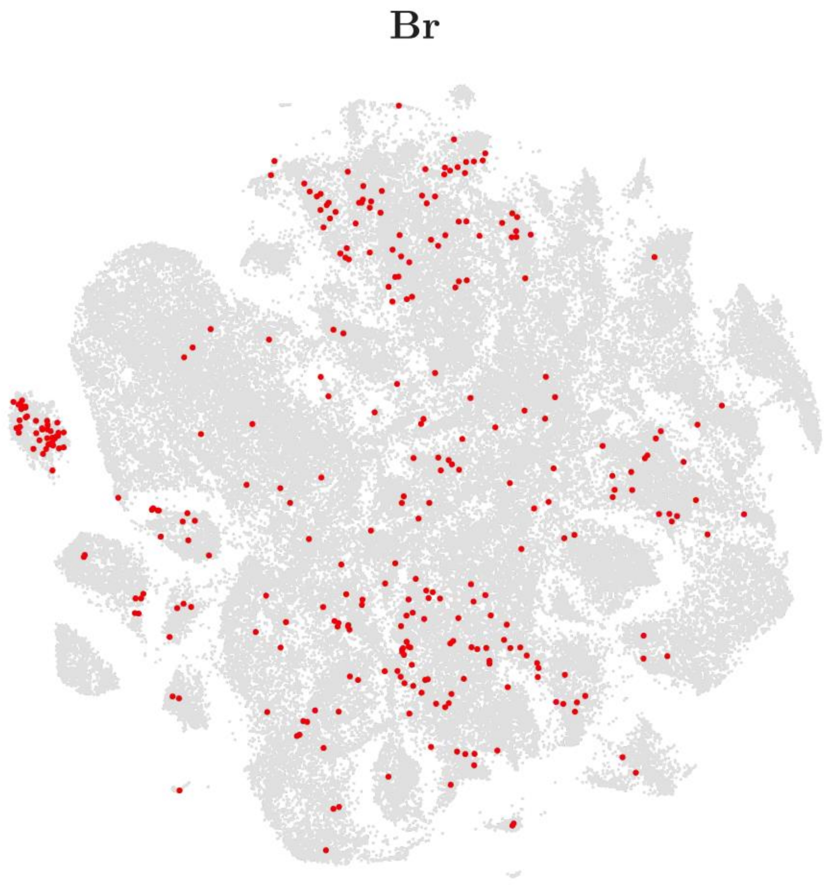

Figure S76. Caption Cluster plot for Krypton.

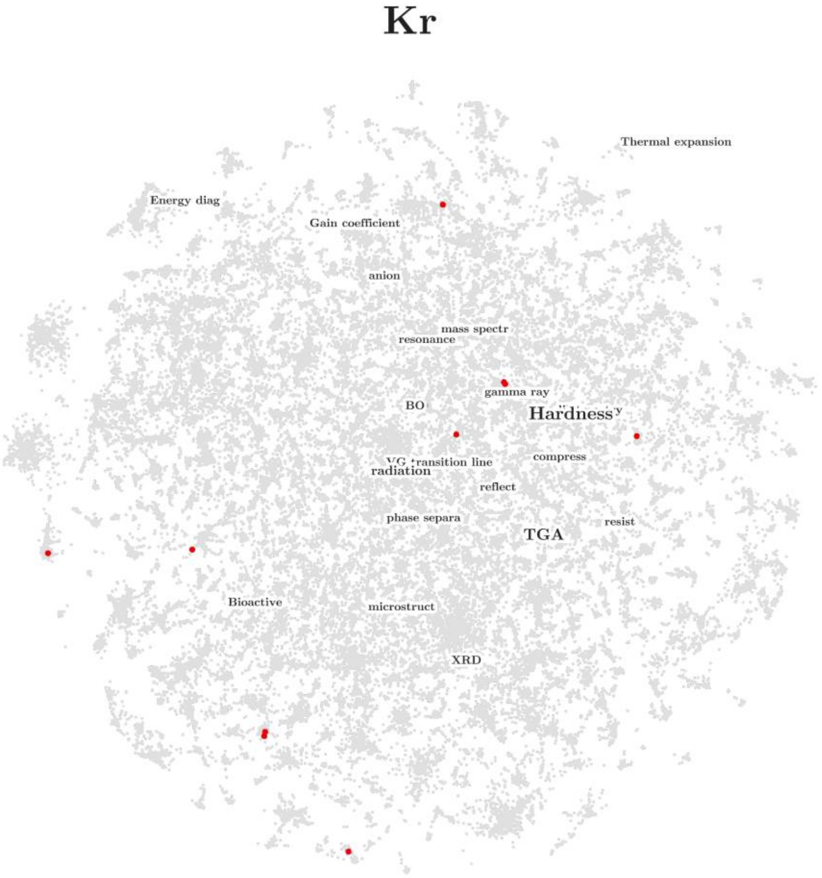

Figure S77. Latent Dirichlet Allocation plot for Krypton.

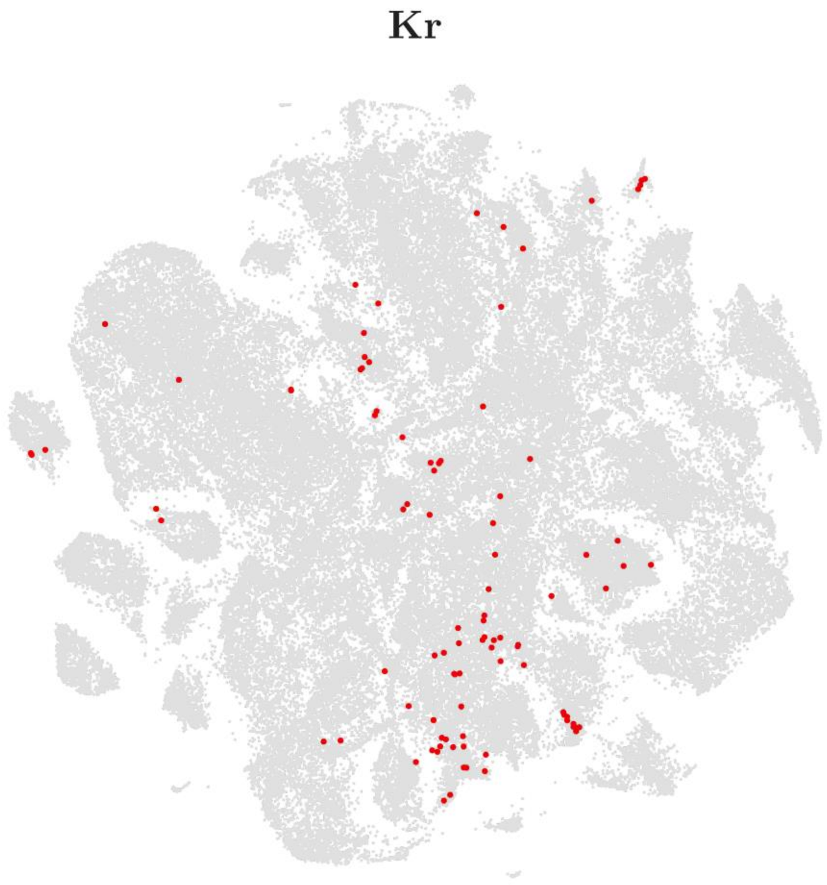

Figure S78. Caption Cluster plot for Rubidium.

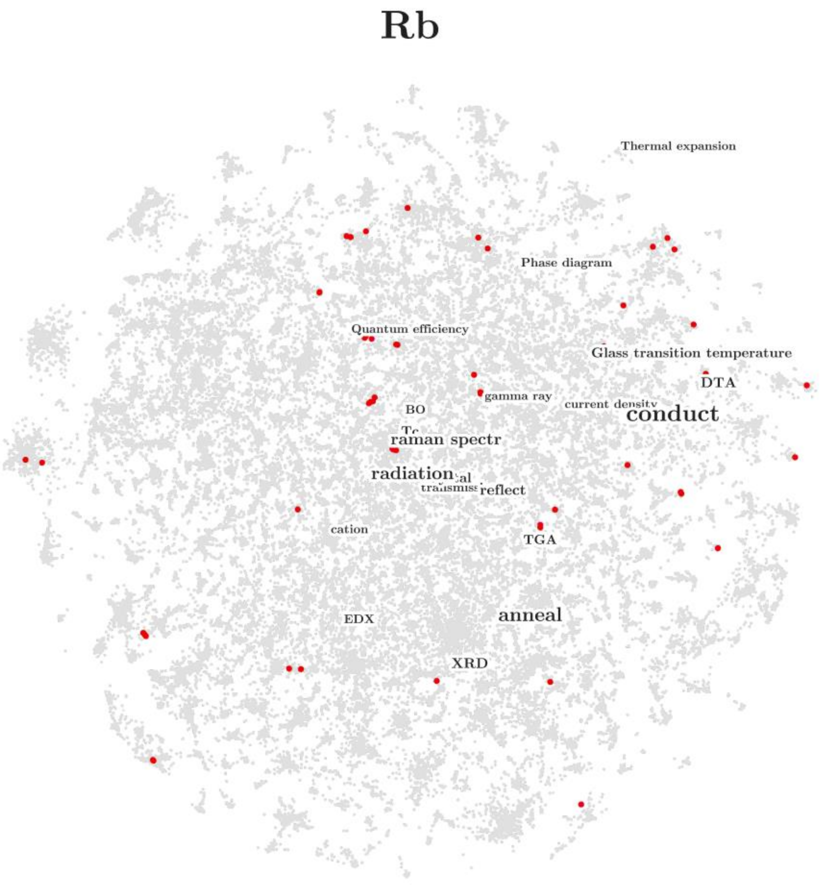

Figure S79. Latent Dirichlet Allocation plot for Rubidium.

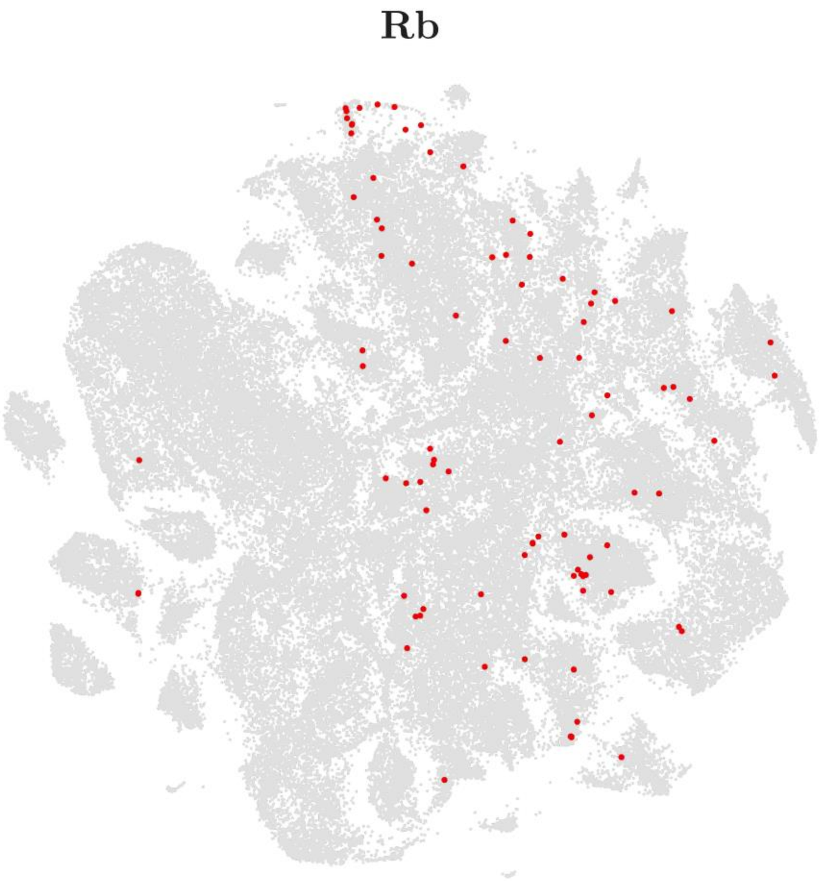

Figure S80. Caption Cluster plot for Strontium.

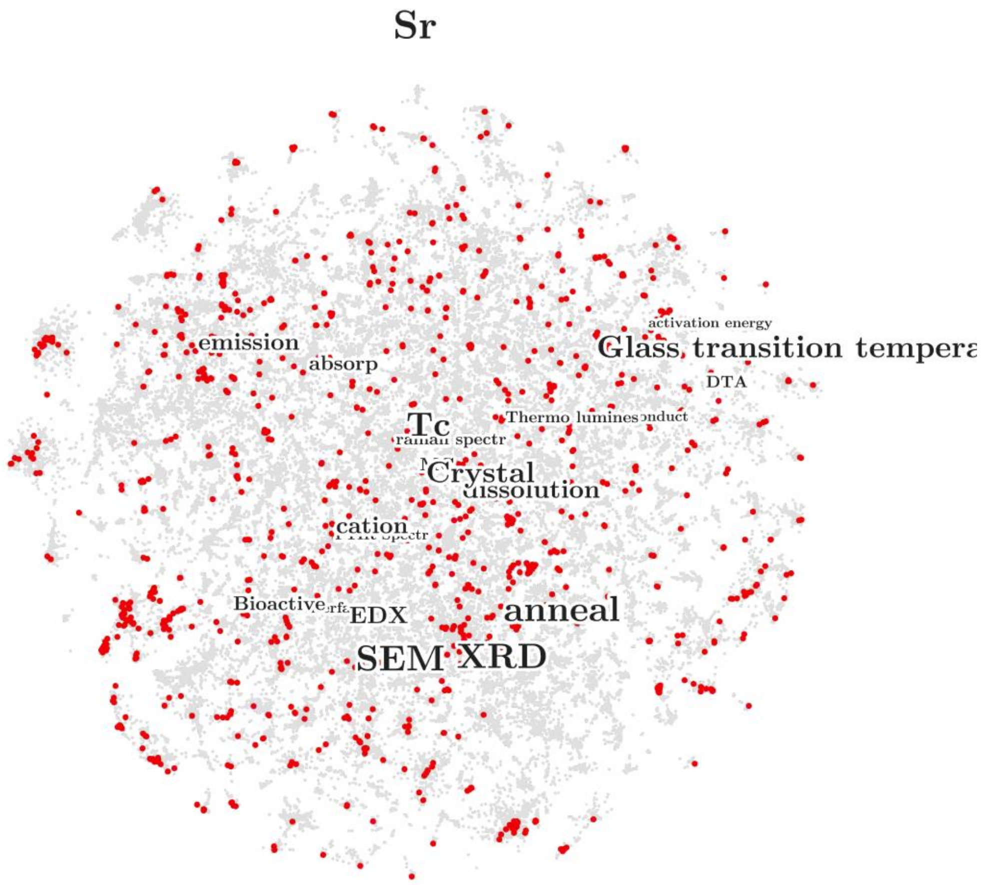

Figure S81. Latent Dirichlet Allocation plot for Strontium.

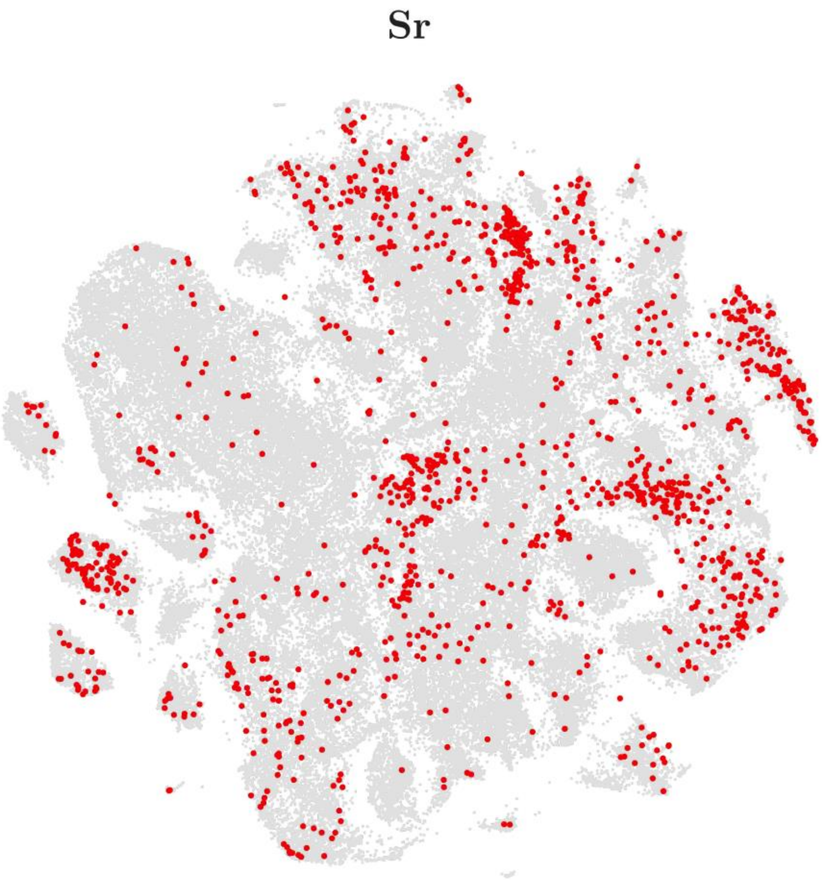

Figure S82. Caption Cluster plot for Yttrium.

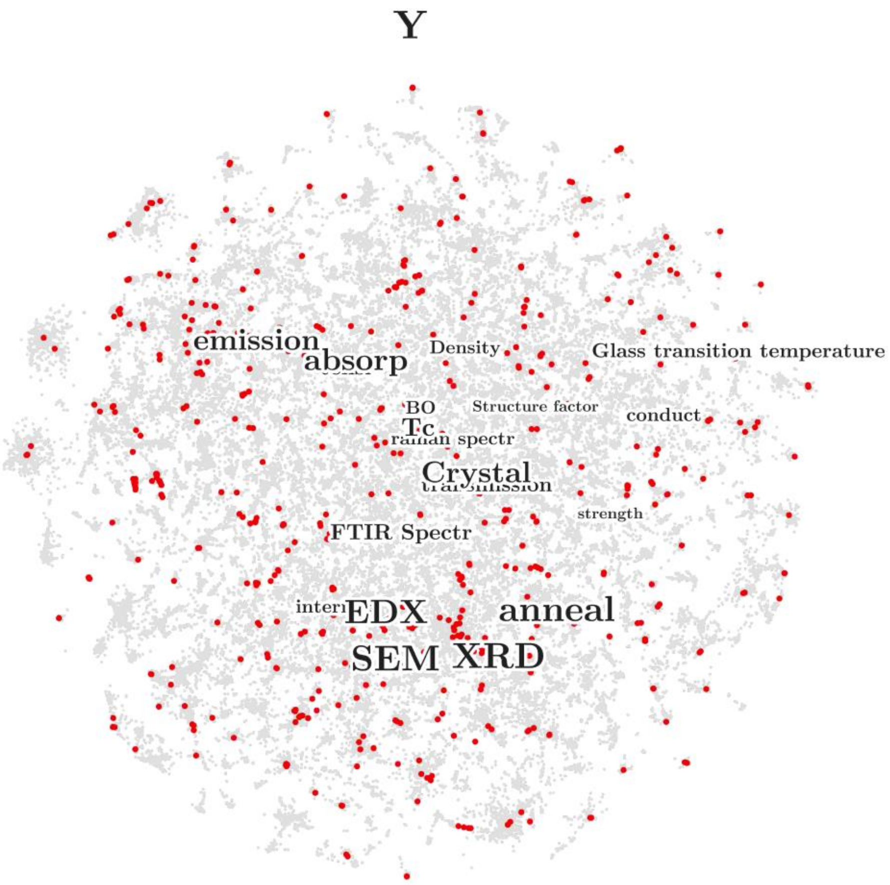

Figure S83. Latent Dirichlet Allocation plot for Yttrium.

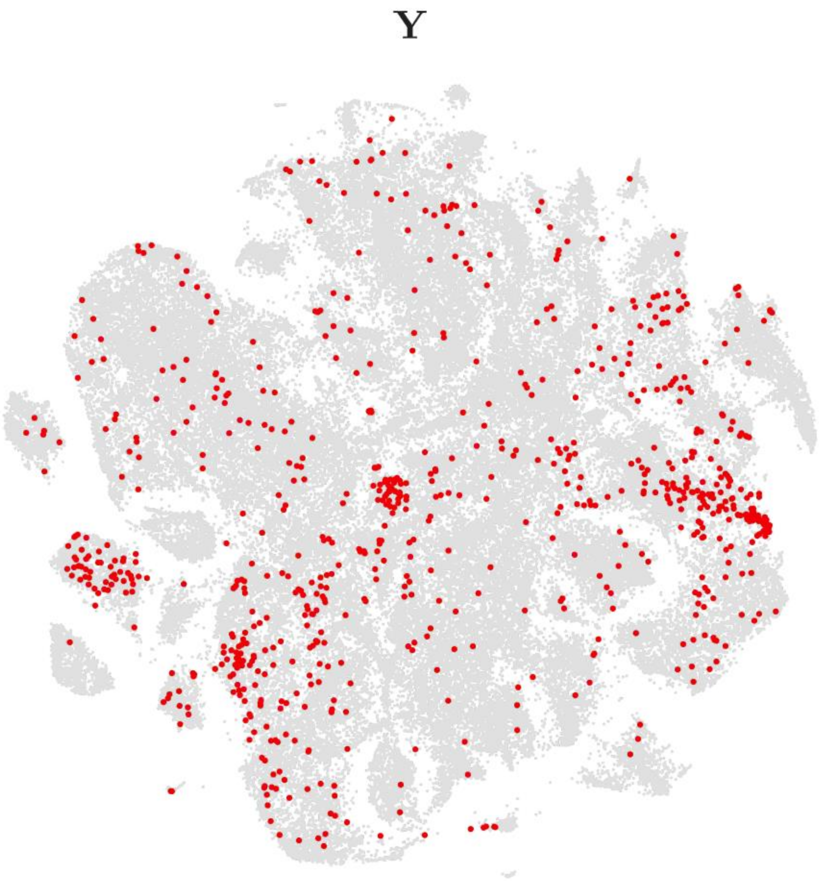

Figure S84. Caption Cluster plot for Zirconium.

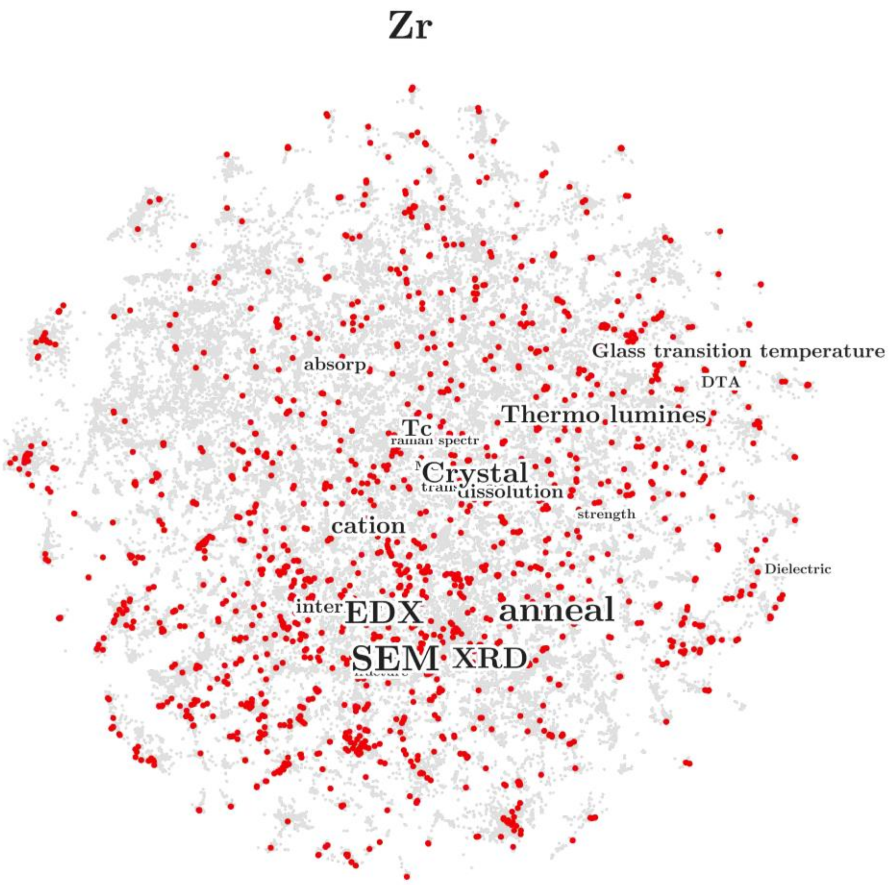

Figure S85. Latent Dirichlet Allocation plot for Zirconium.

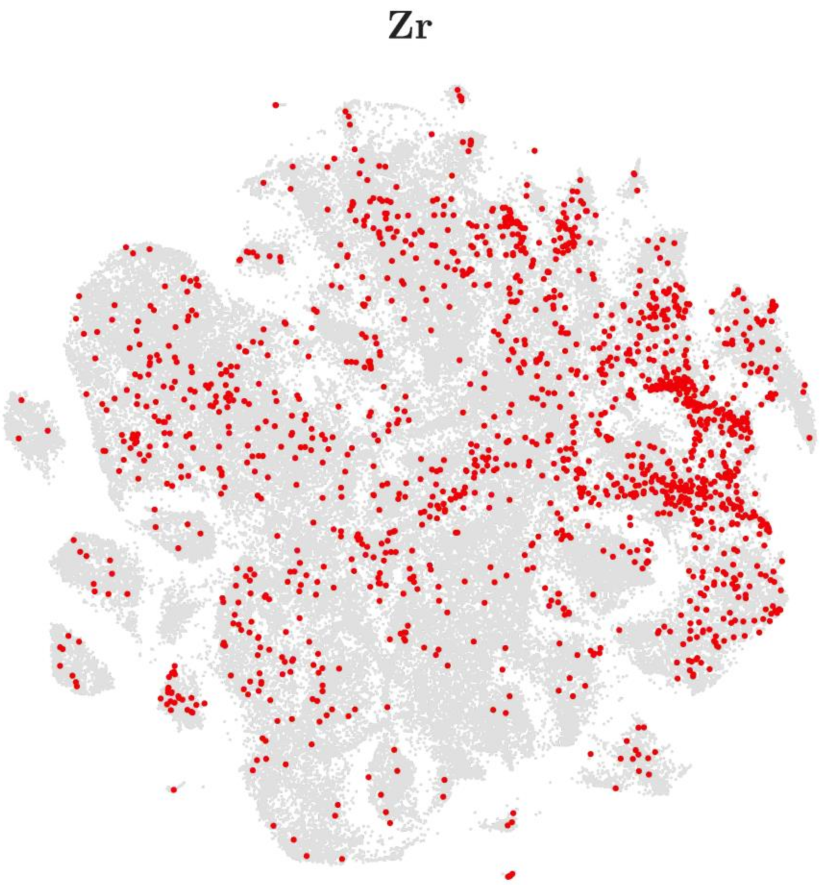

Figure S86. Caption Cluster plot for Niobium.

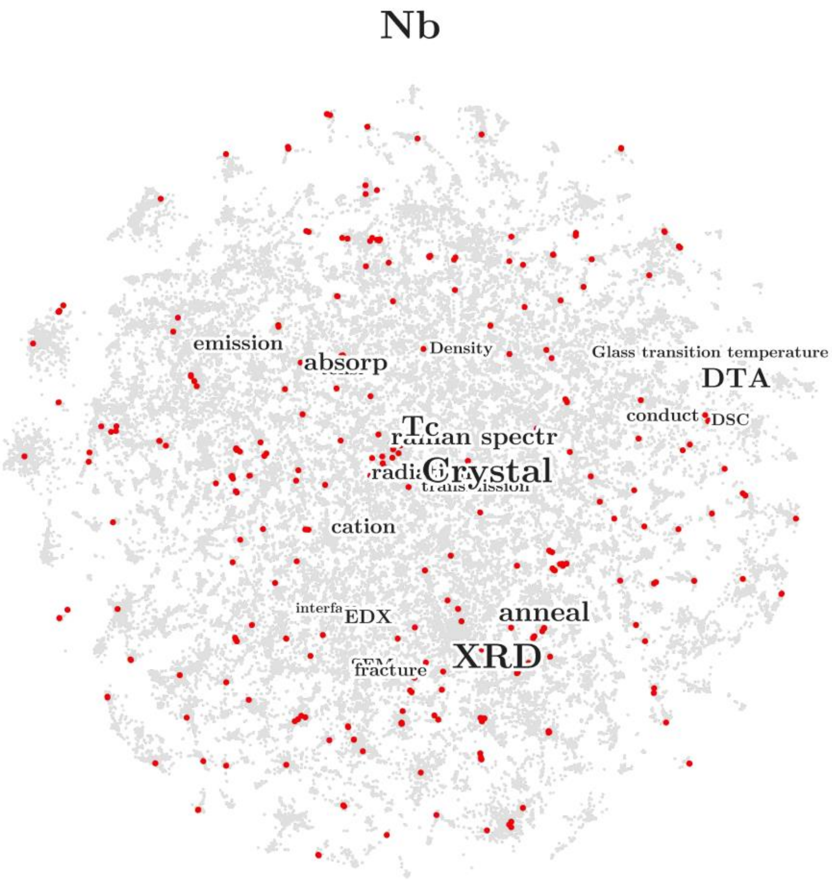

Figure S87. Latent Dirichlet Allocation plot for Niobium.

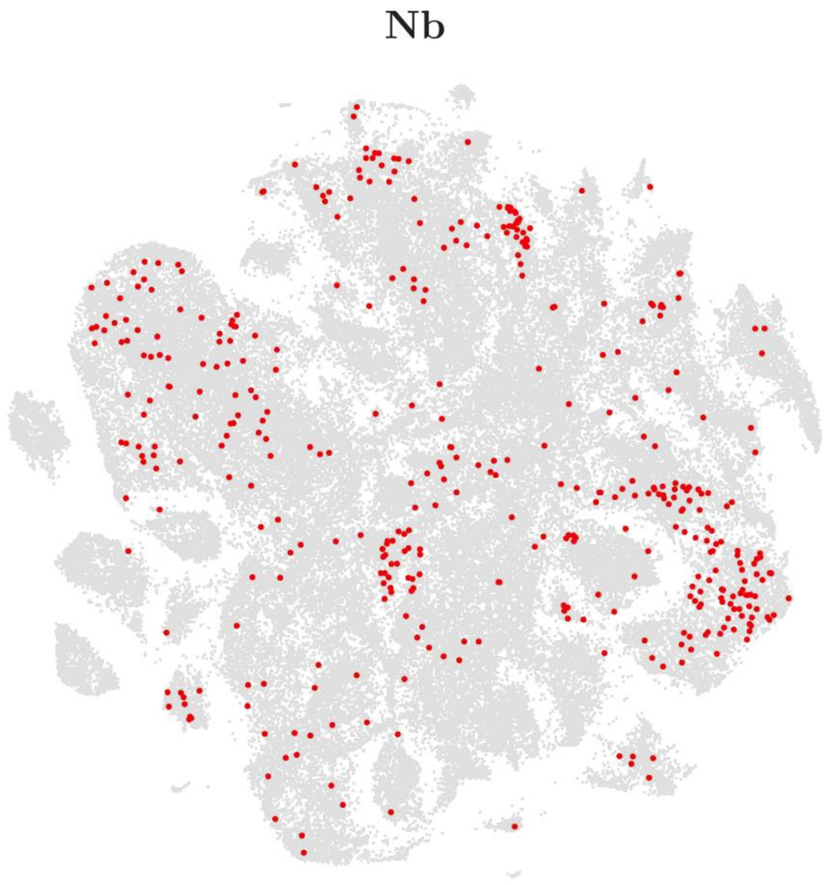

Figure S88. Caption Cluster plot for Molybdenum.

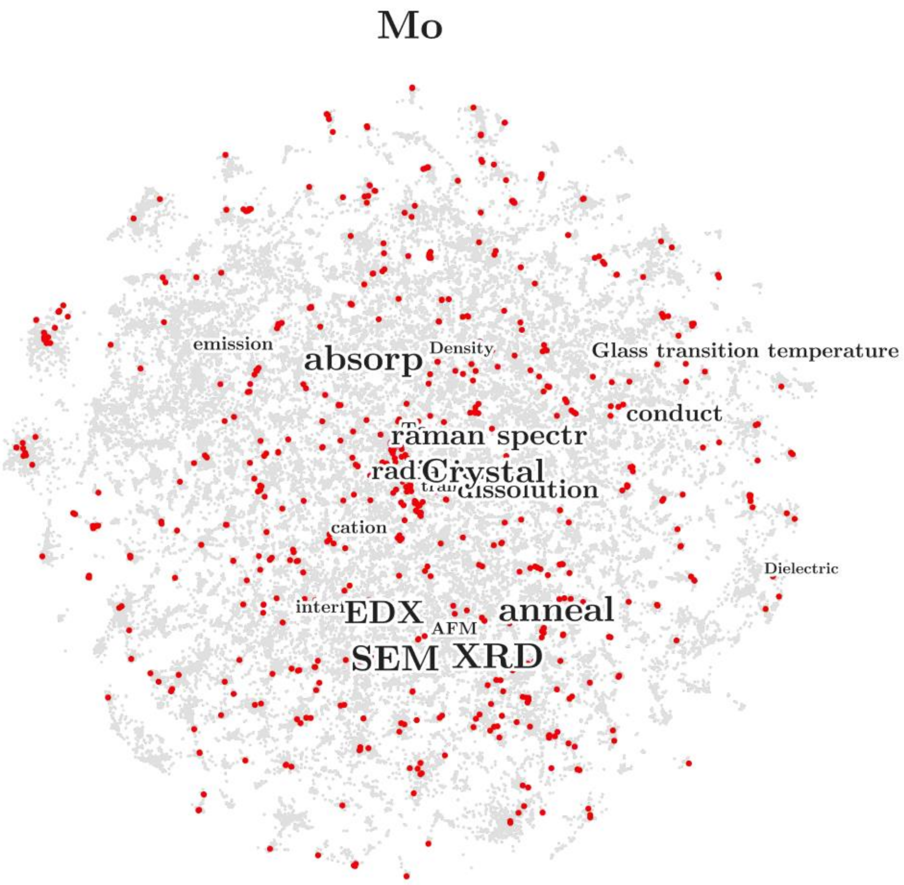

Figure S89. Latent Dirichlet Allocation plot for Molybdenum.

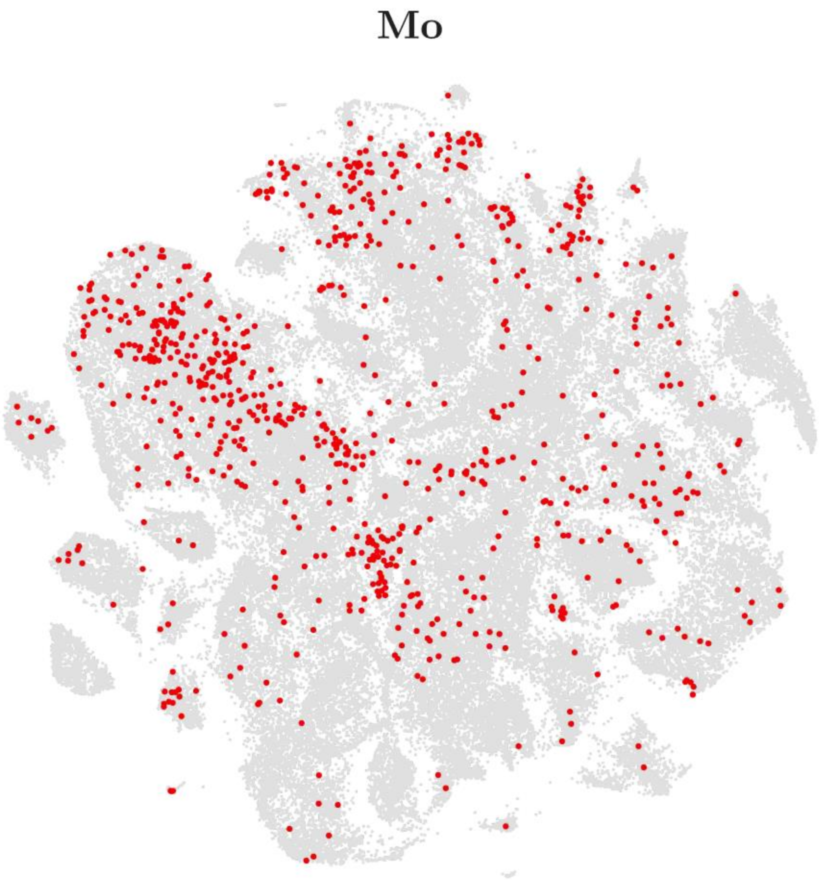

Figure S90. Caption Cluster plot for Technetium.

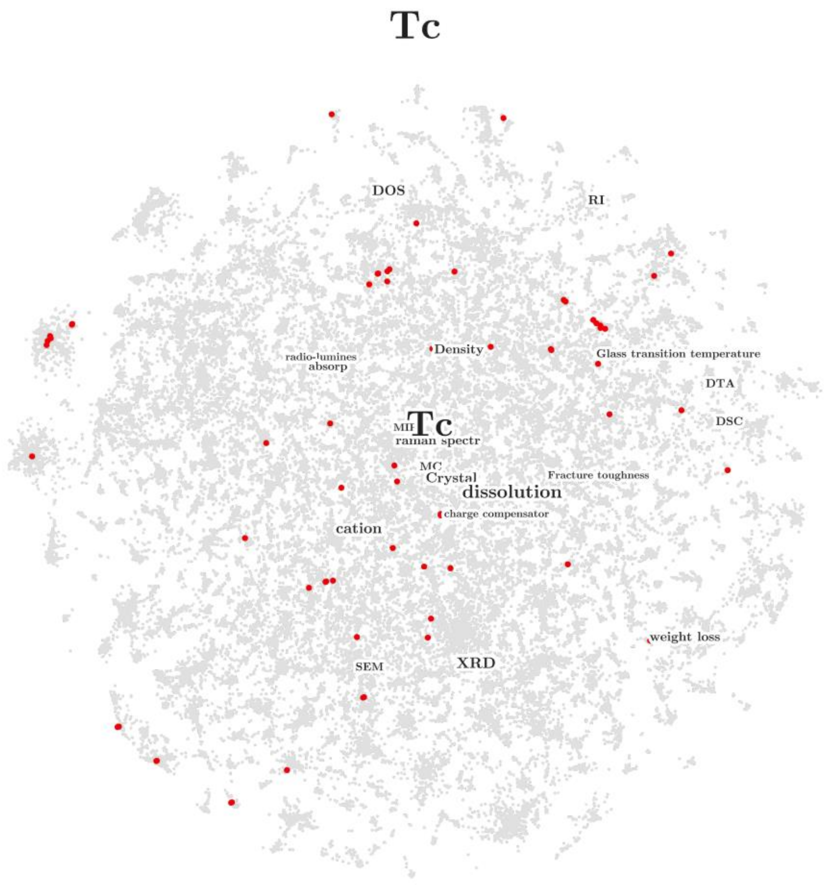

Figure S91. Latent Dirichlet Allocation plot for Technetium.

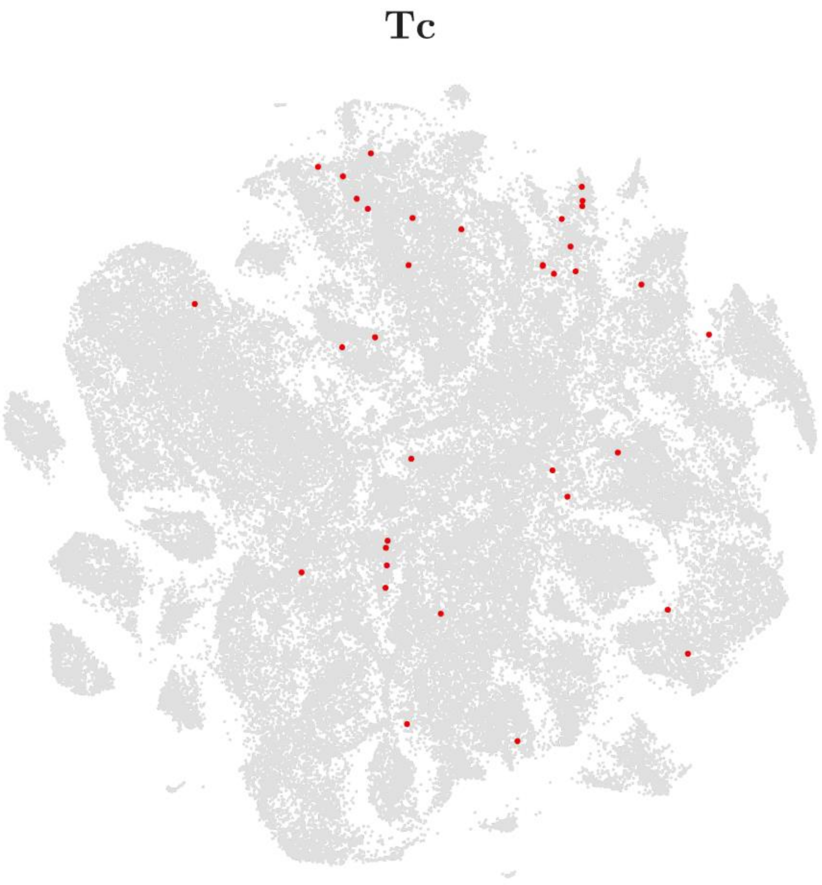

Figure S92. Caption Cluster plot for Ruthenium.

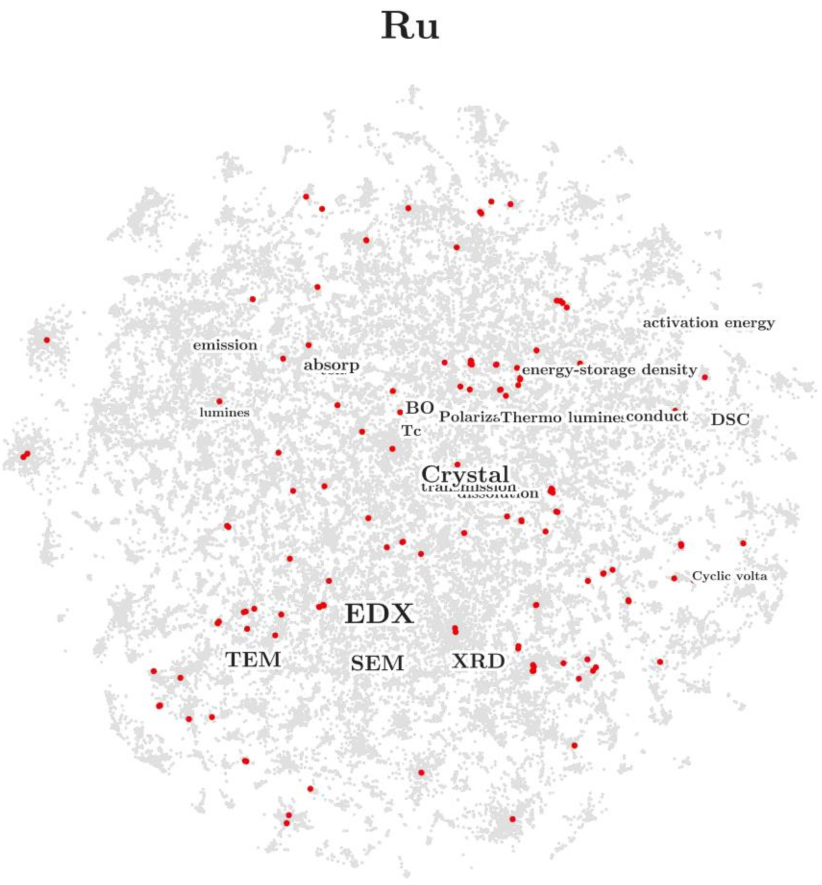

Figure S93. Latent Dirichlet Allocation plot for Ruthenium.

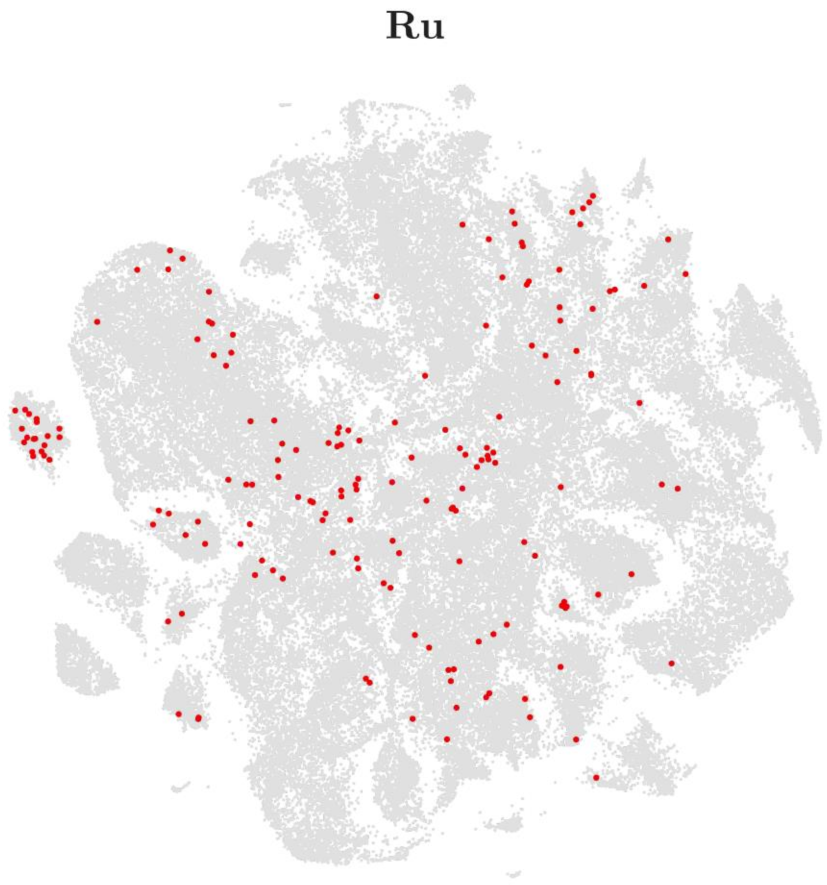

Figure S94. Caption Cluster plot for Rhodium.

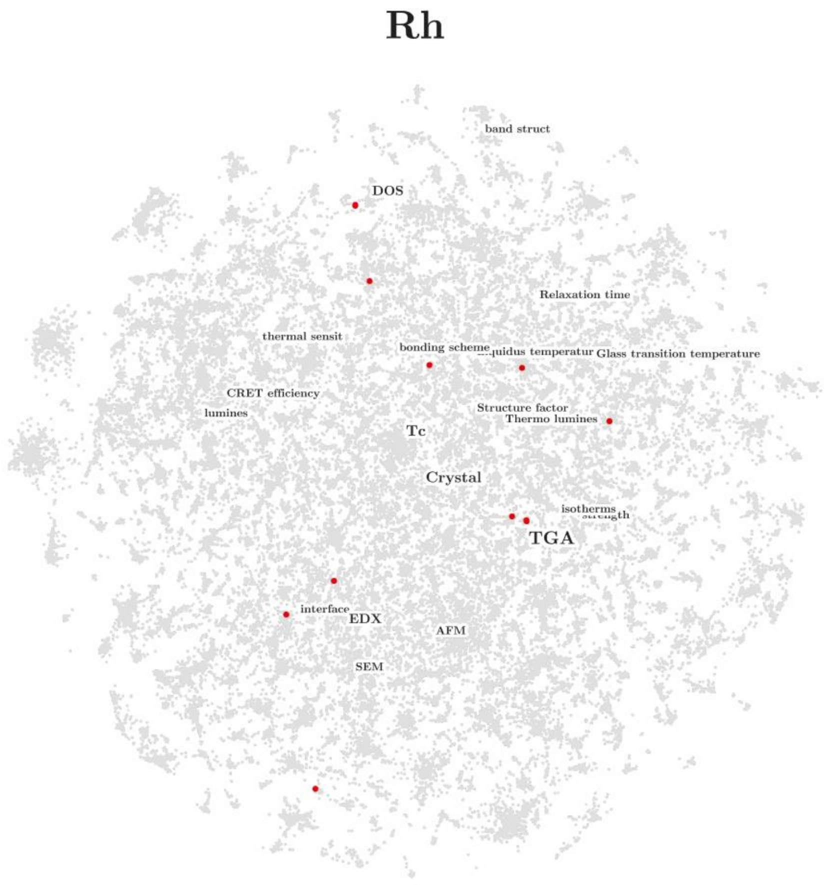

Figure S95. Latent Dirichlet Allocation plot for Rhodium.

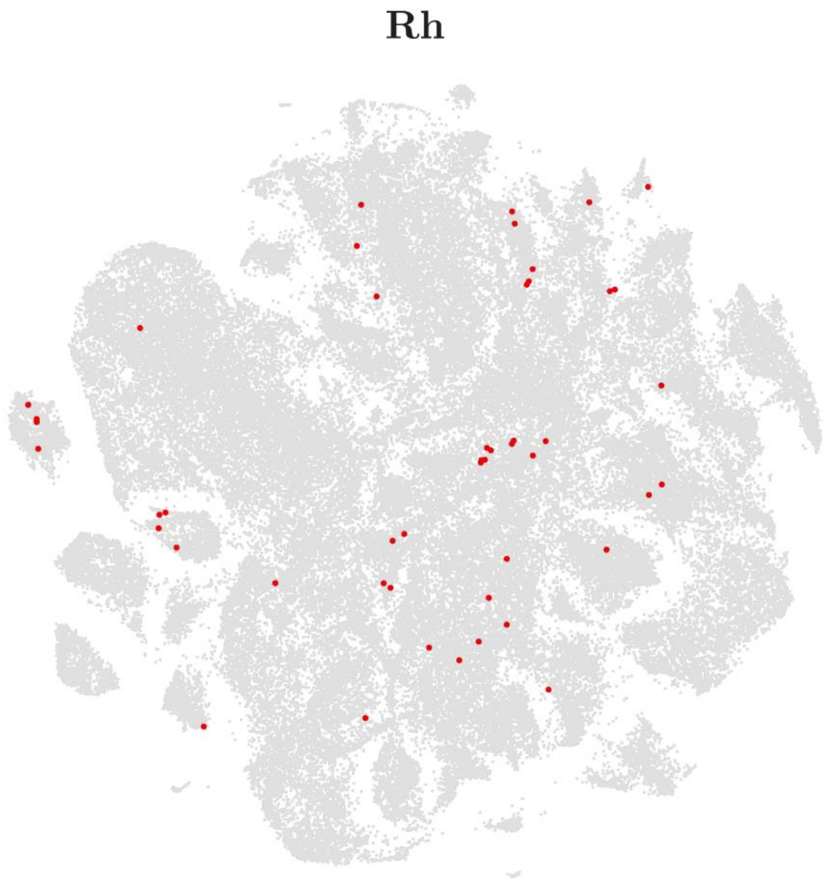

Figure S96. Caption Cluster plot for Palladium.

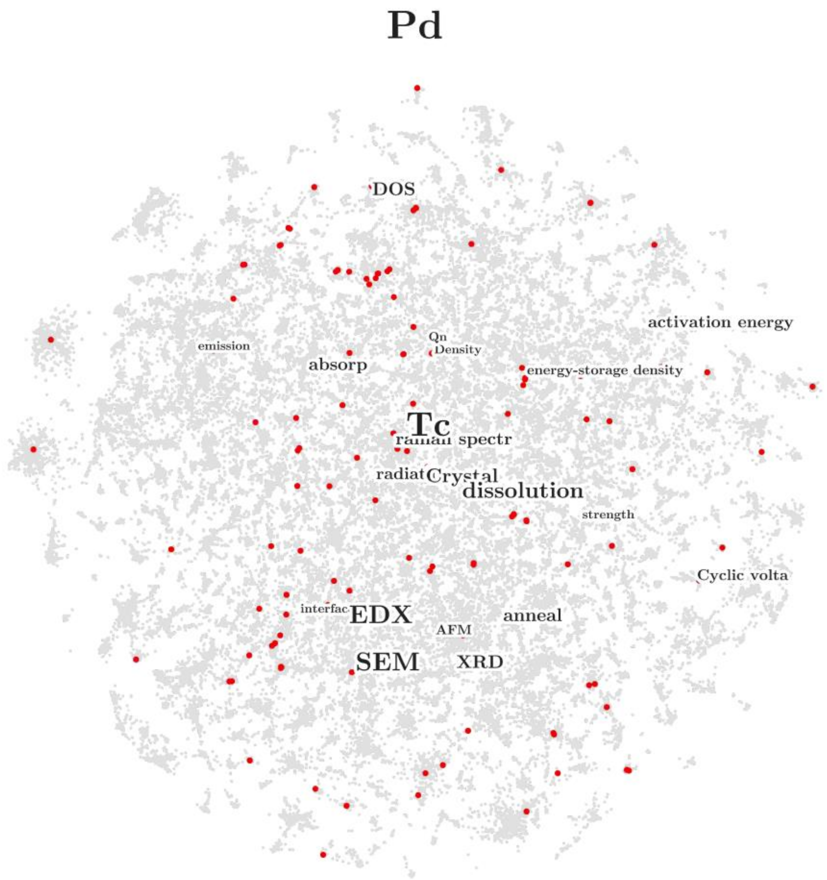

Figure S97. Latent Dirichlet Allocation plot for Palladium.

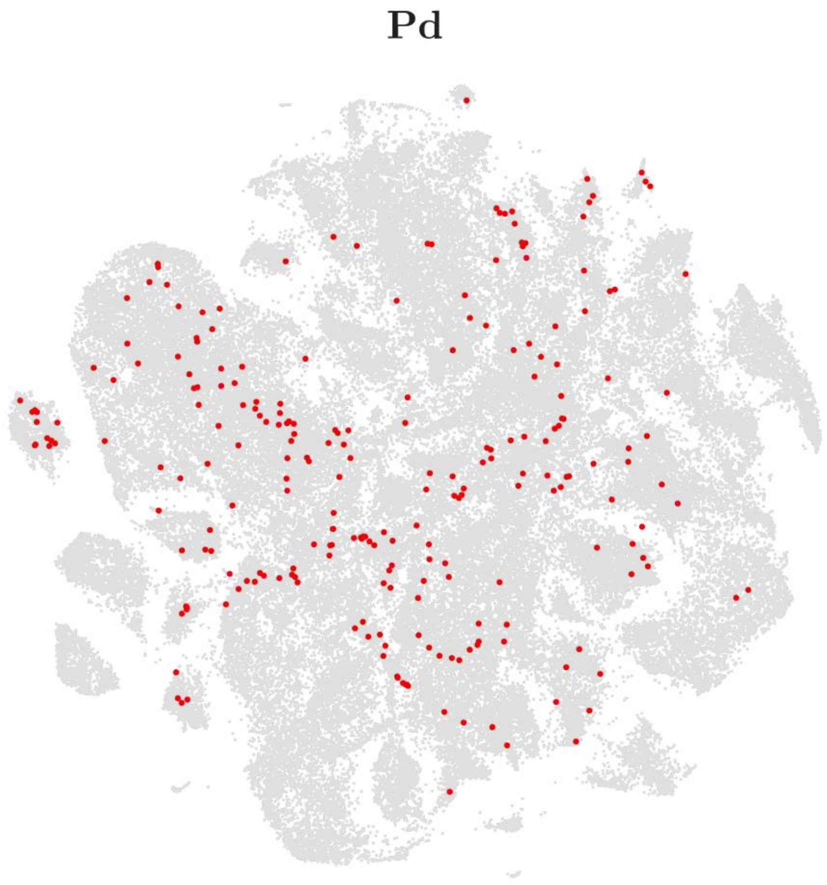

Figure S98. Caption Cluster plot for Silver.

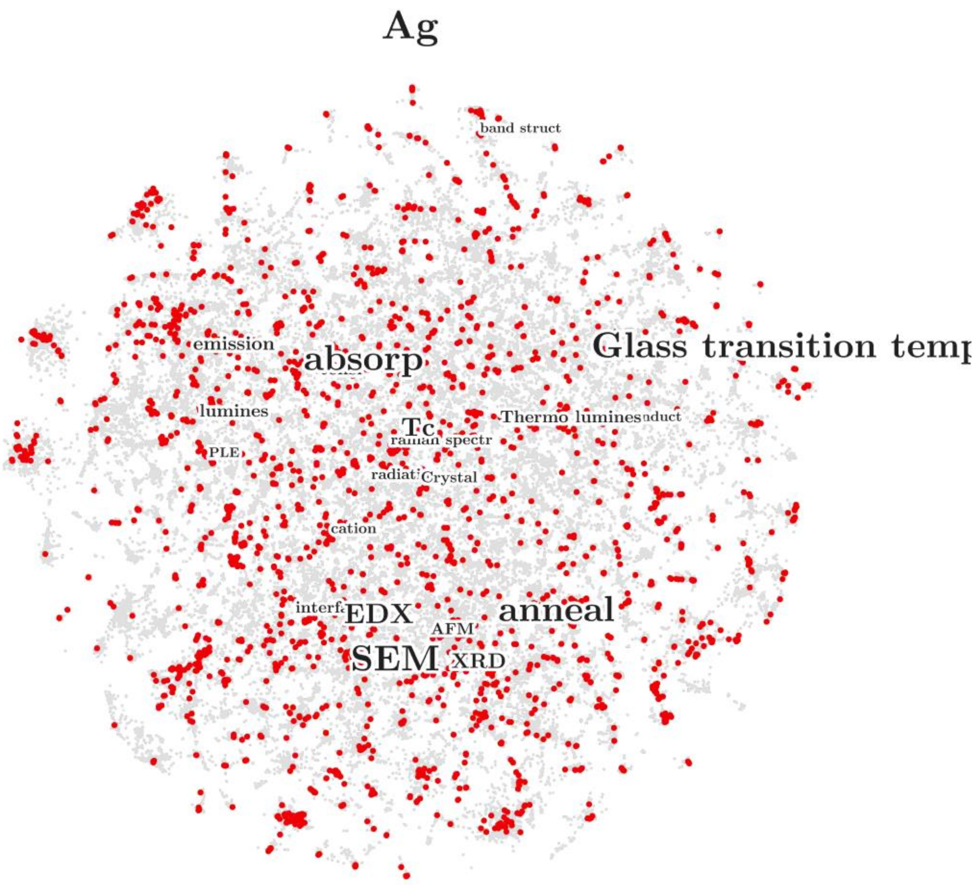

Figure S99. Latent Dirichlet Allocation plot for Silver.

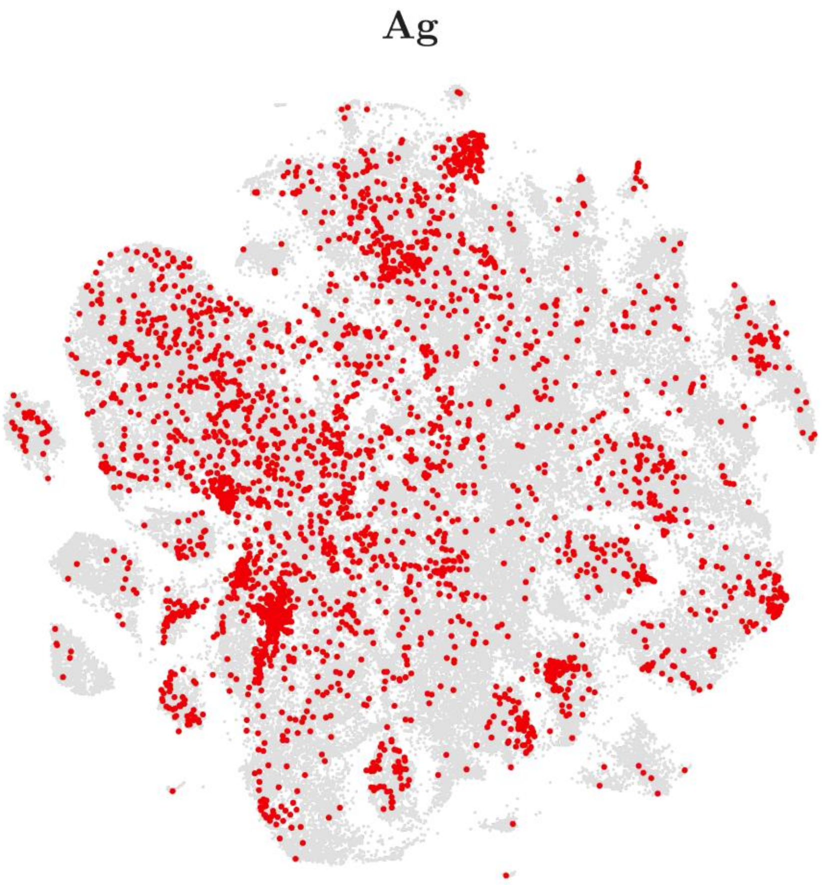

Figure S100. Caption Cluster plot for Cadmium.

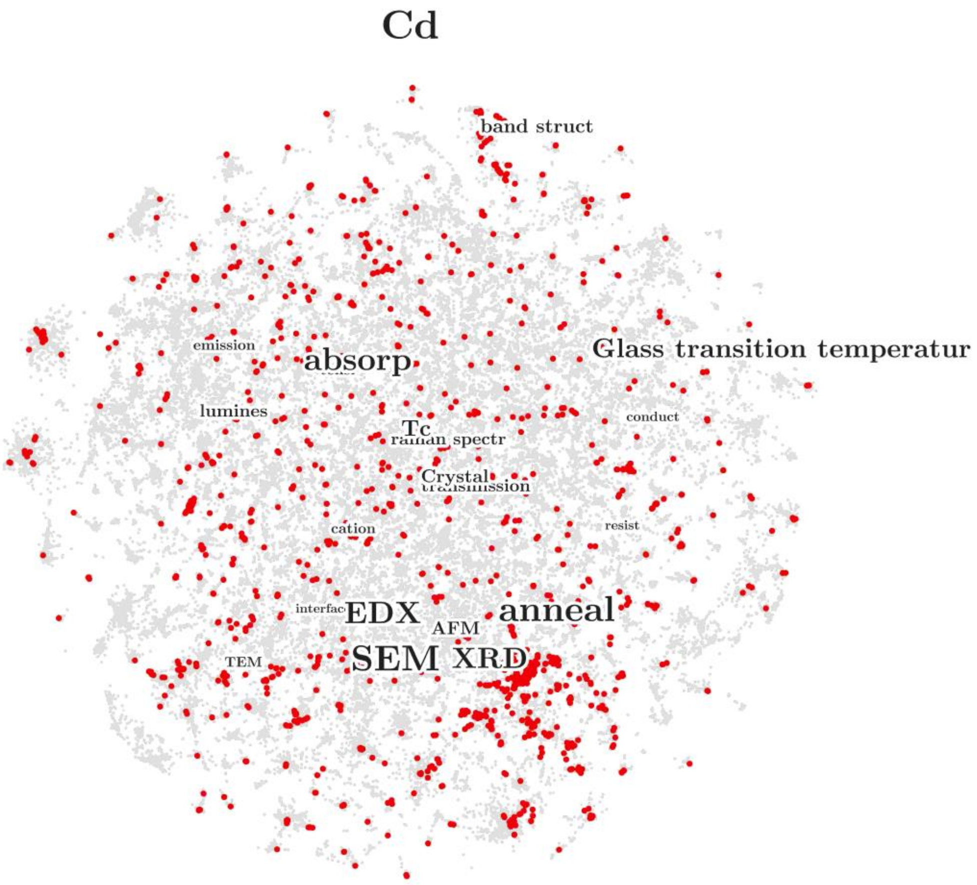

Figure S101. Latent Dirichlet Allocation plot for Cadmium.

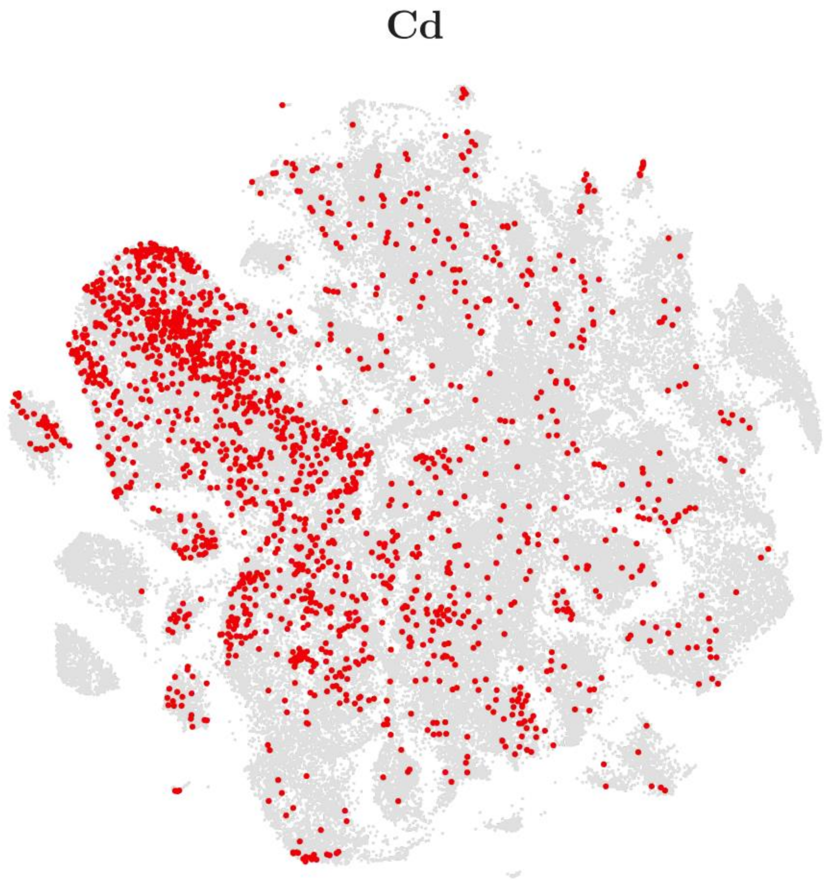

Figure S102. Caption Cluster plot for Indium.

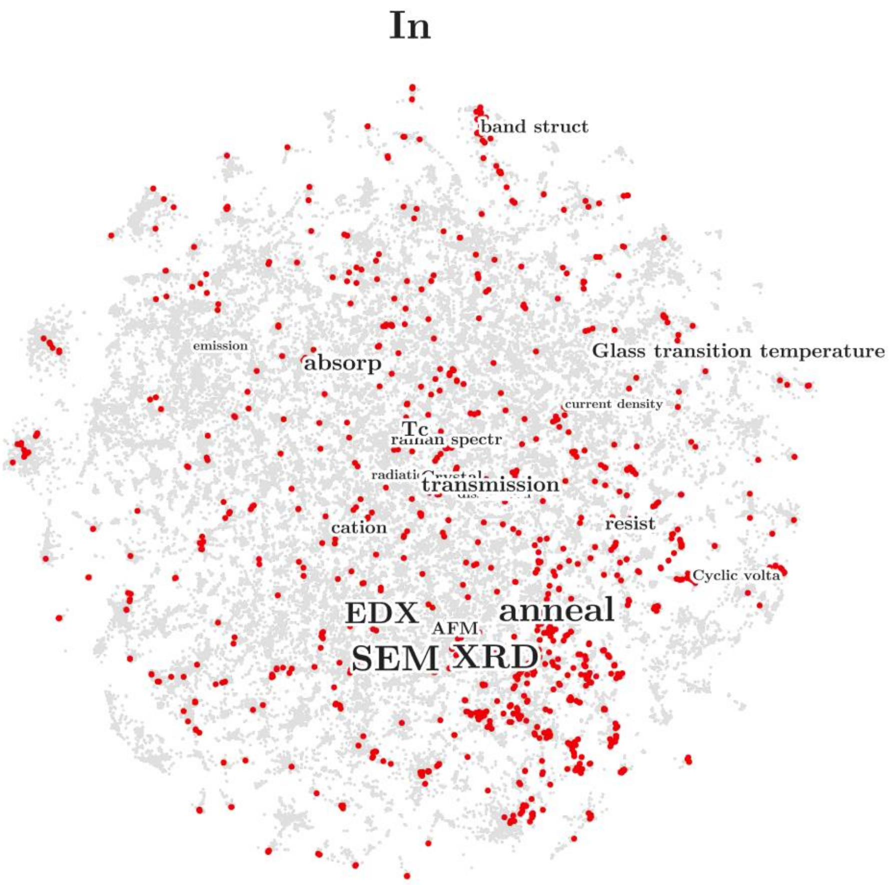

Figure S103. Latent Dirichlet Allocation plot for Indium.

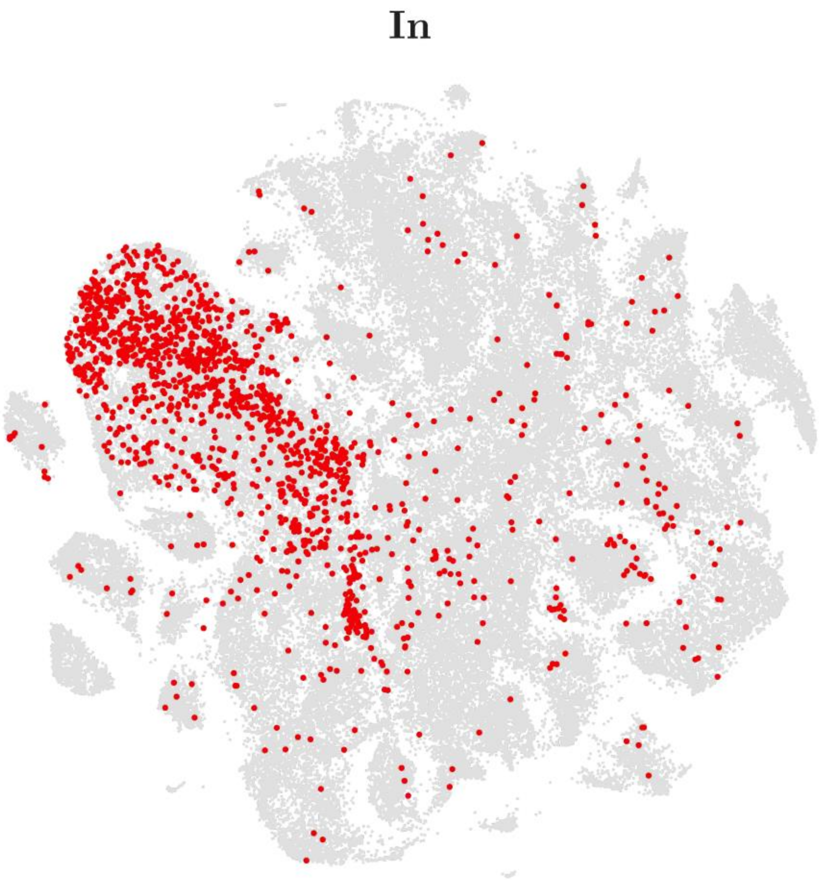

Figure S104. Caption Cluster plot for Tin.

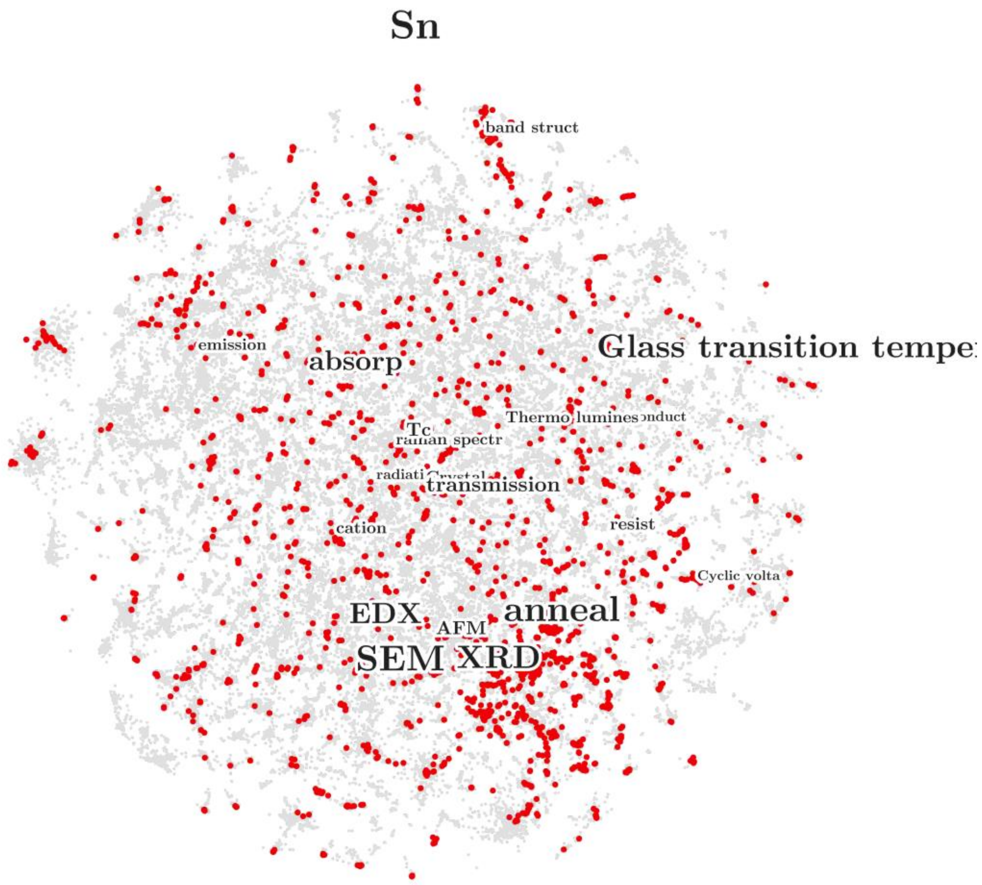

Figure S105. Latent Dirichlet Allocation plot for Tin.

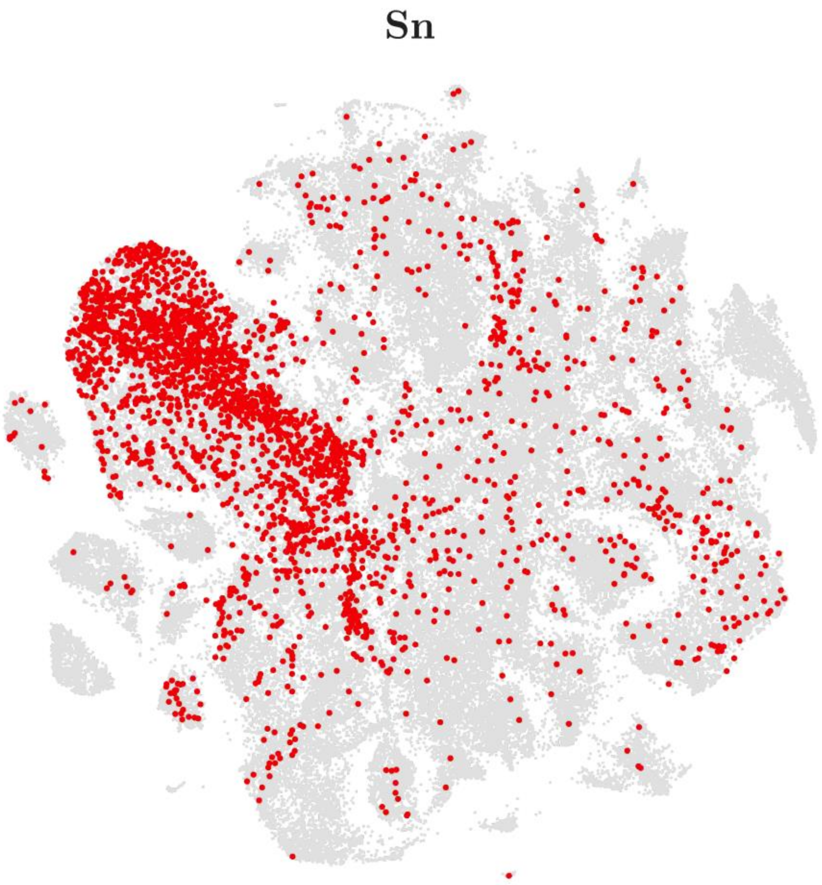

Figure S106. Caption Cluster plot for Antimony.

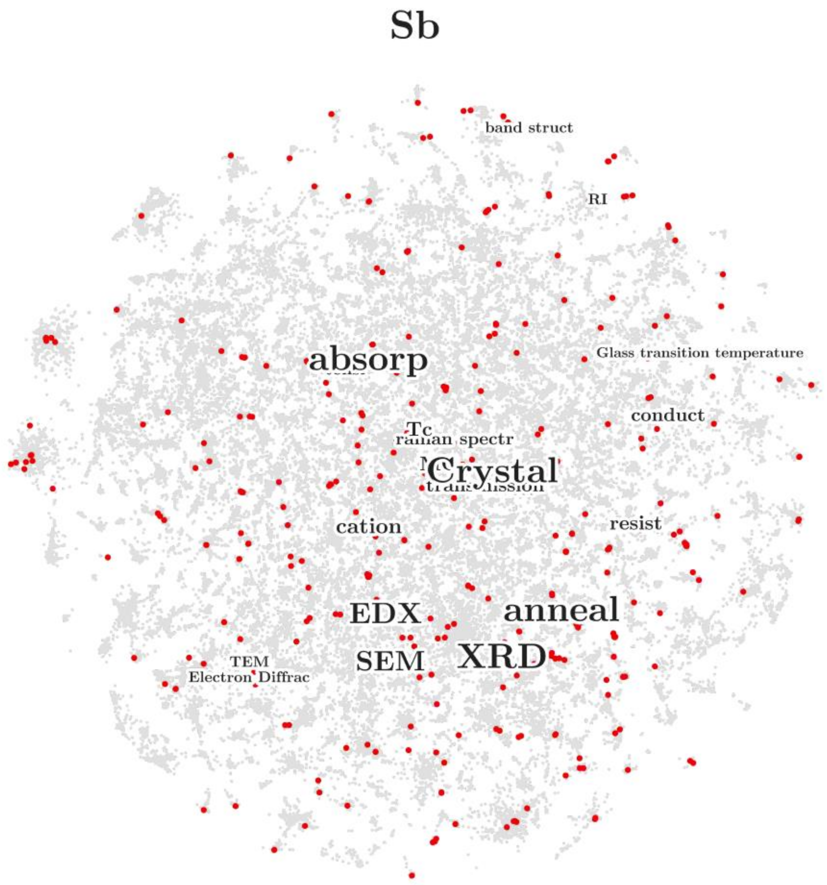

Figure S107. Latent Dirichlet Allocation plot for Antimony.

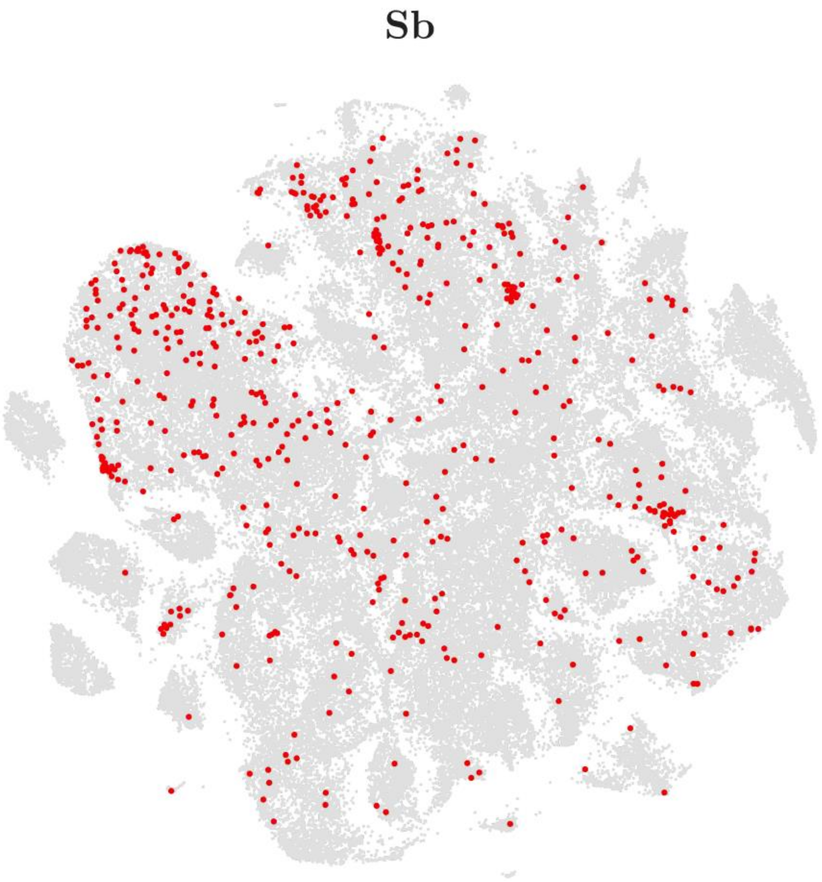

Figure S108. Caption Cluster plot for Tellurium.

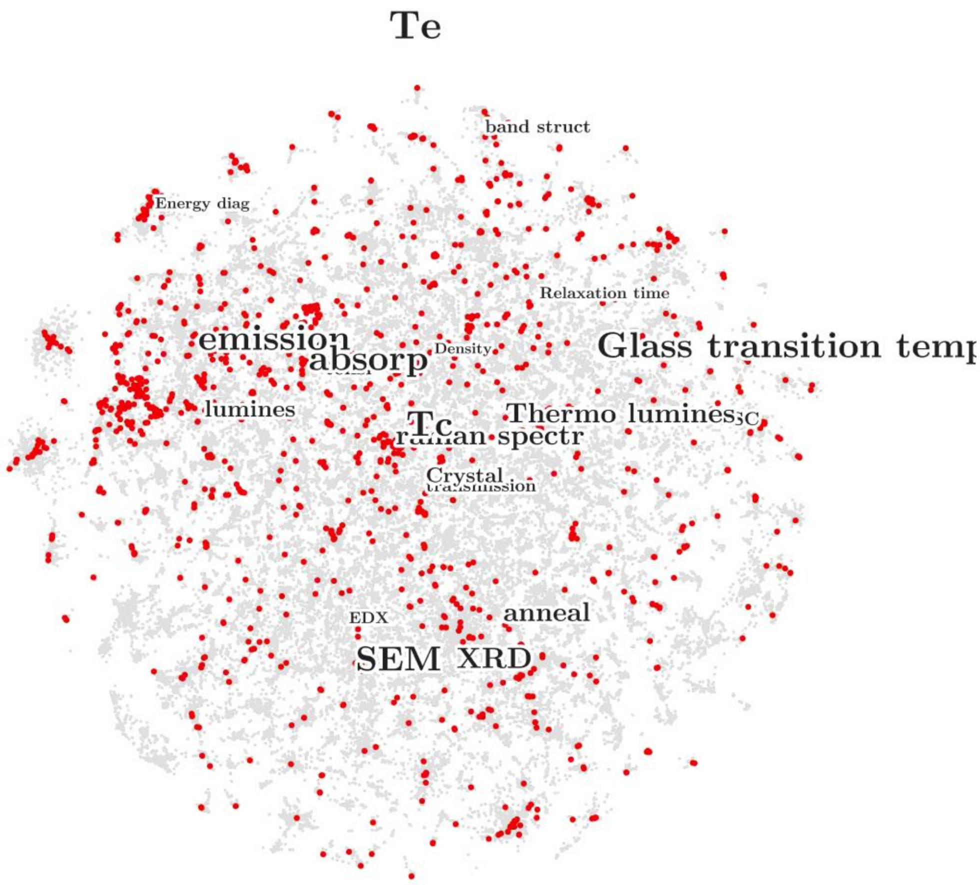

Figure S109. Latent Dirichlet Allocation plot for Tellurium.

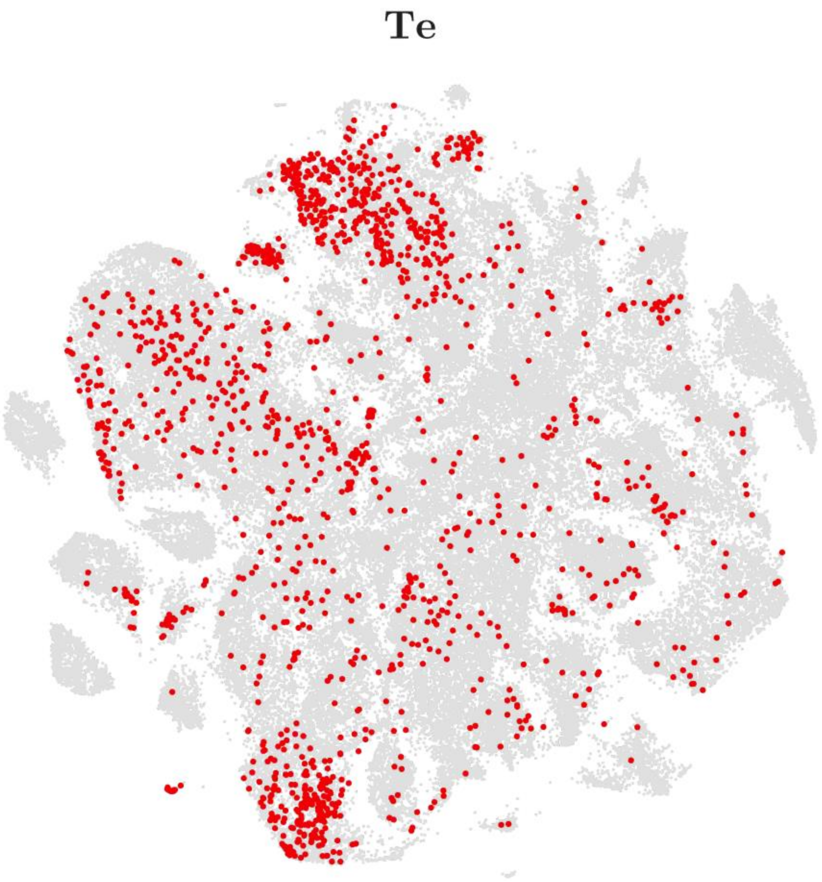

Figure S110. Caption Cluster plot for Iodine.

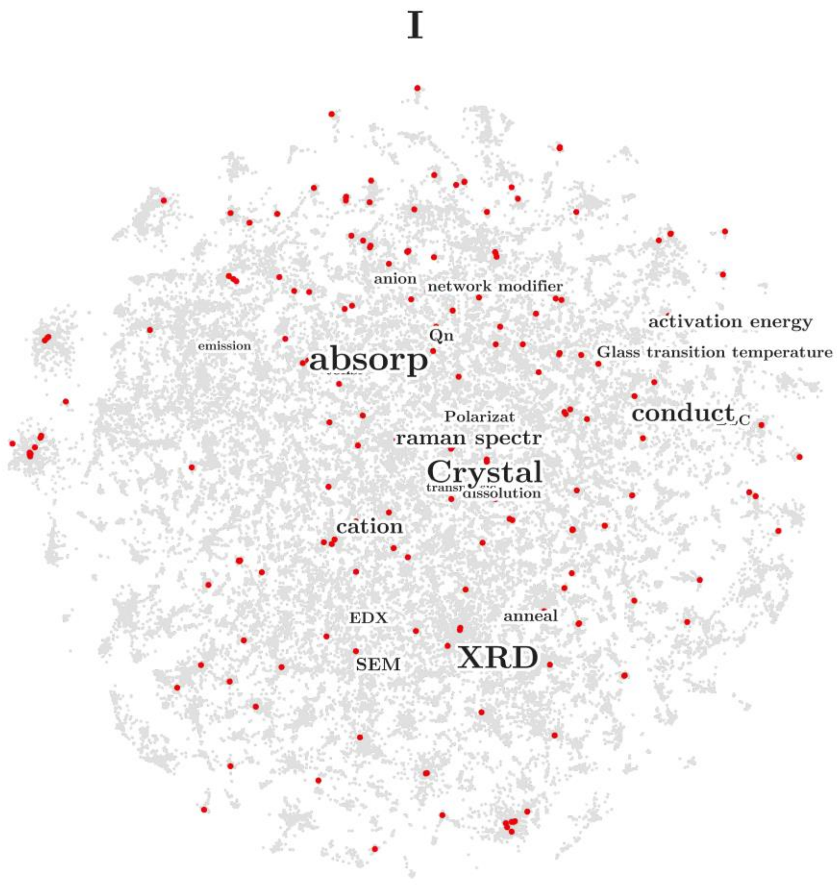

Figure S111. Latent Dirichlet Allocation plot for Iodine.

I

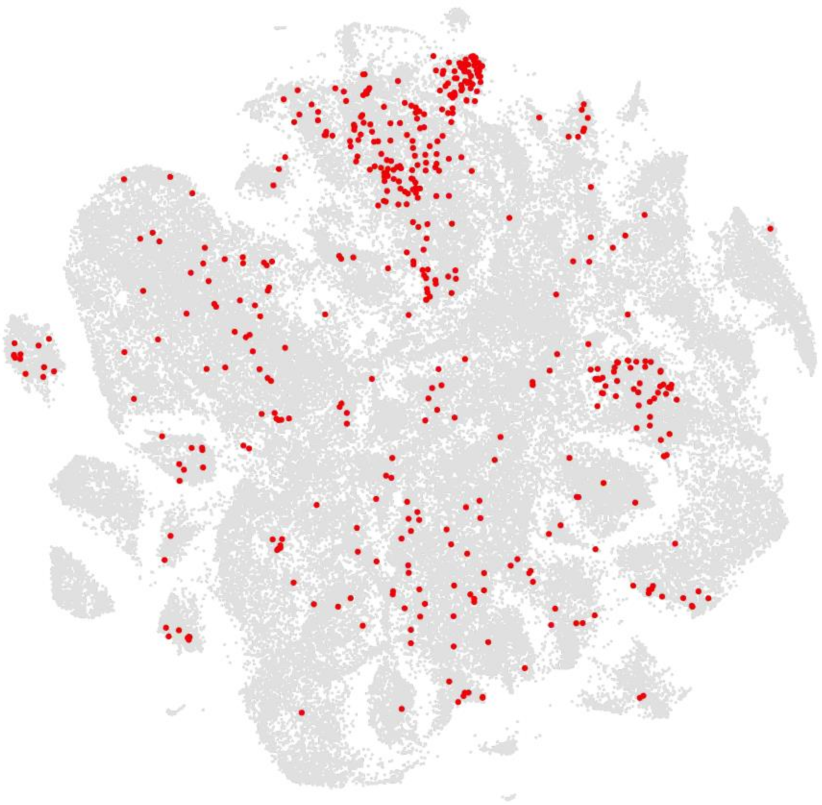

Figure S112. Caption Cluster plot for Xenon.

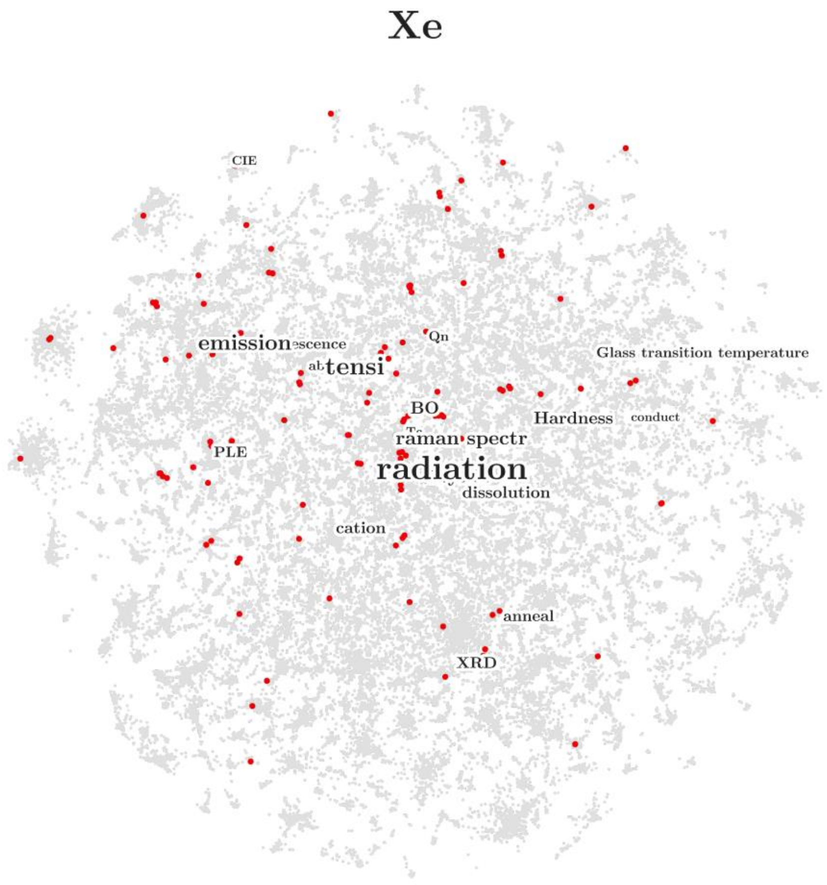

Figure S113. Latent Dirichlet Allocation plot for Xenon.

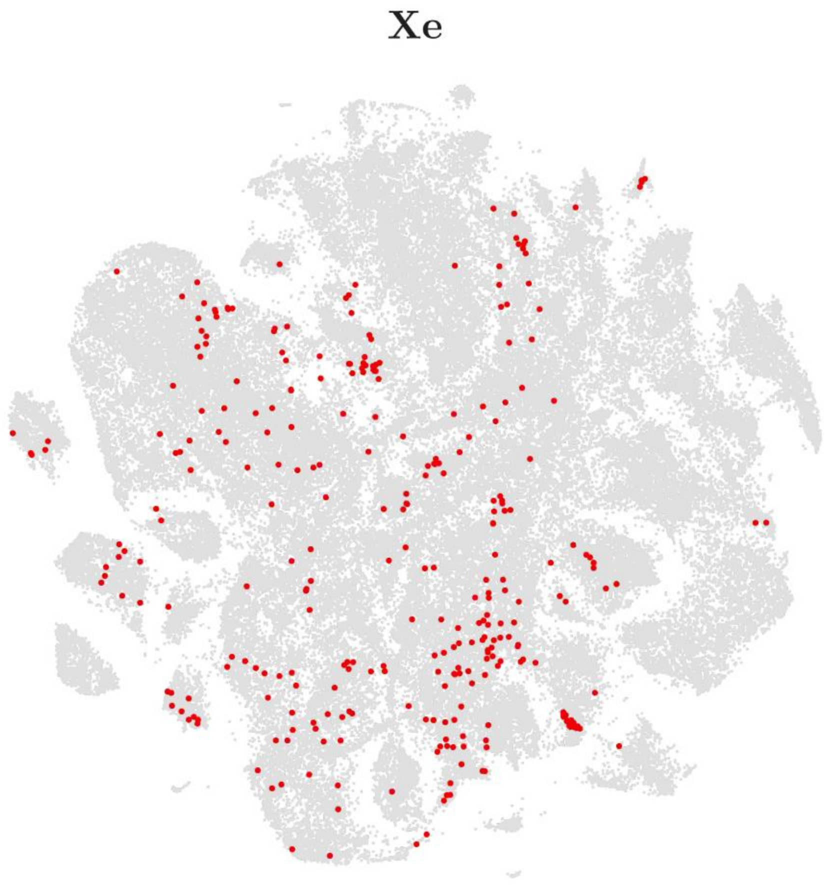

Figure S114. Caption Cluster plot for Cesium.

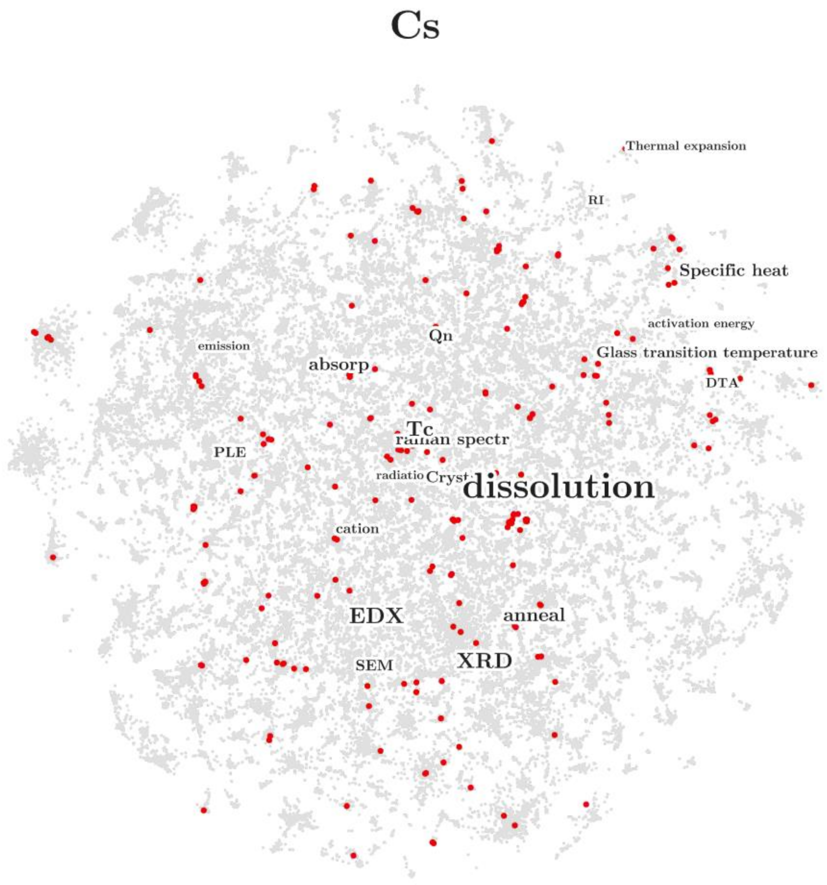

Figure S115. Latent Dirichlet Allocation plot for Cesium.

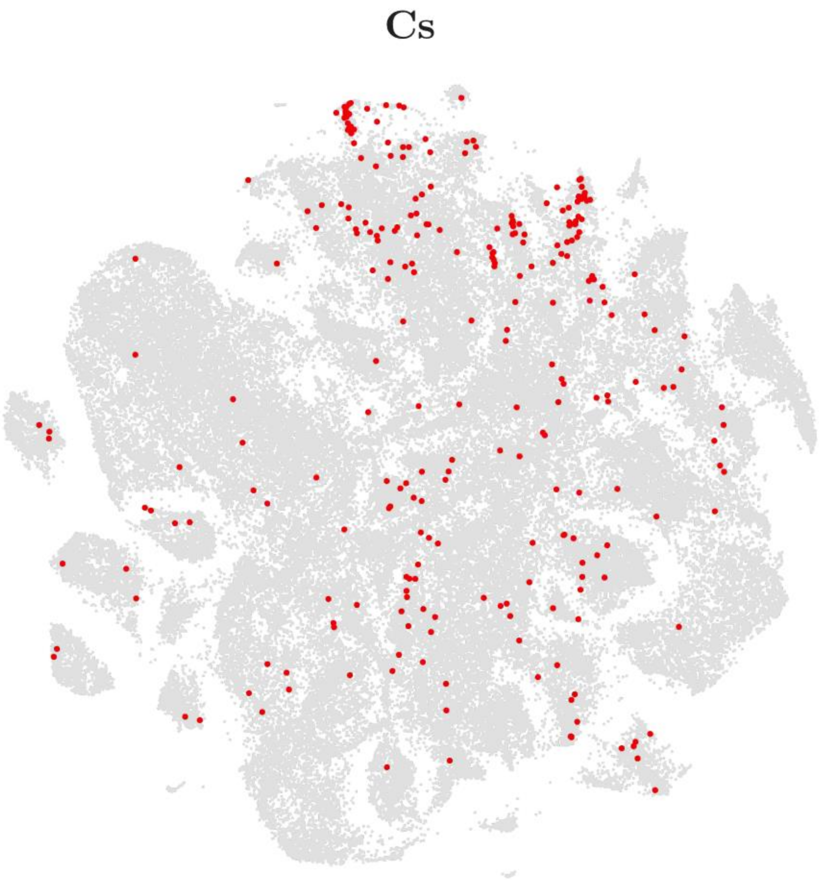

Figure S116. Caption Cluster plot for Barium.

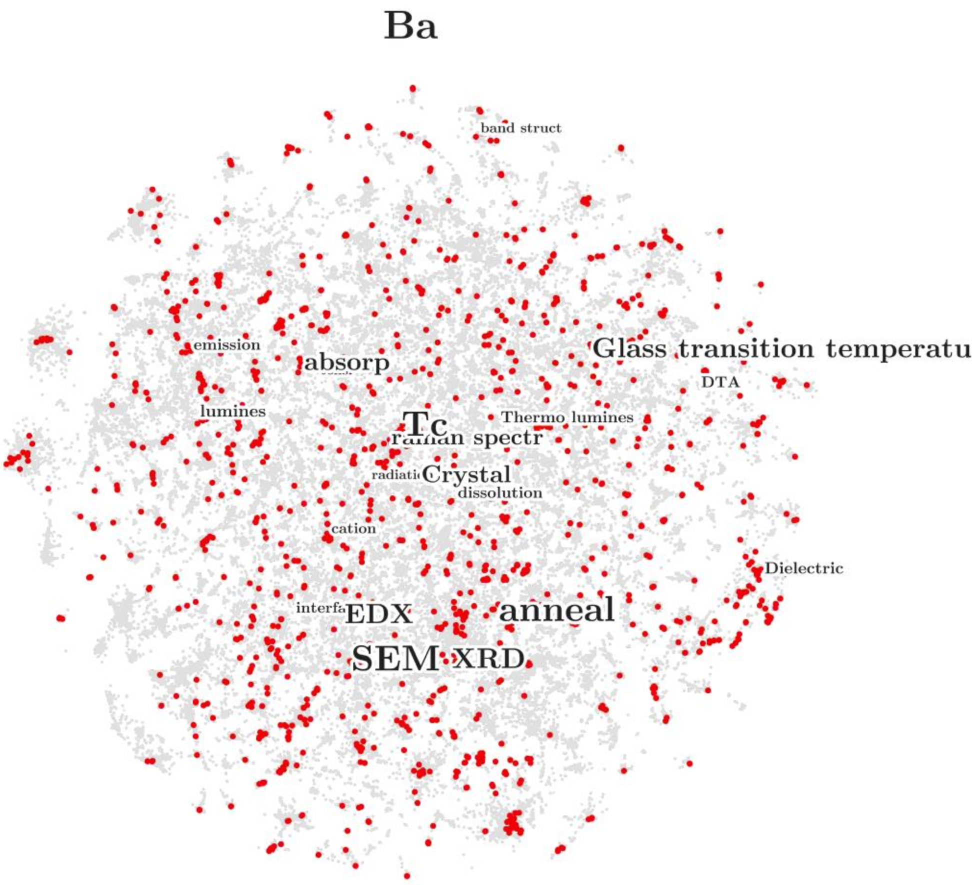

Figure S117. Latent Dirichlet Allocation plot for Barium.

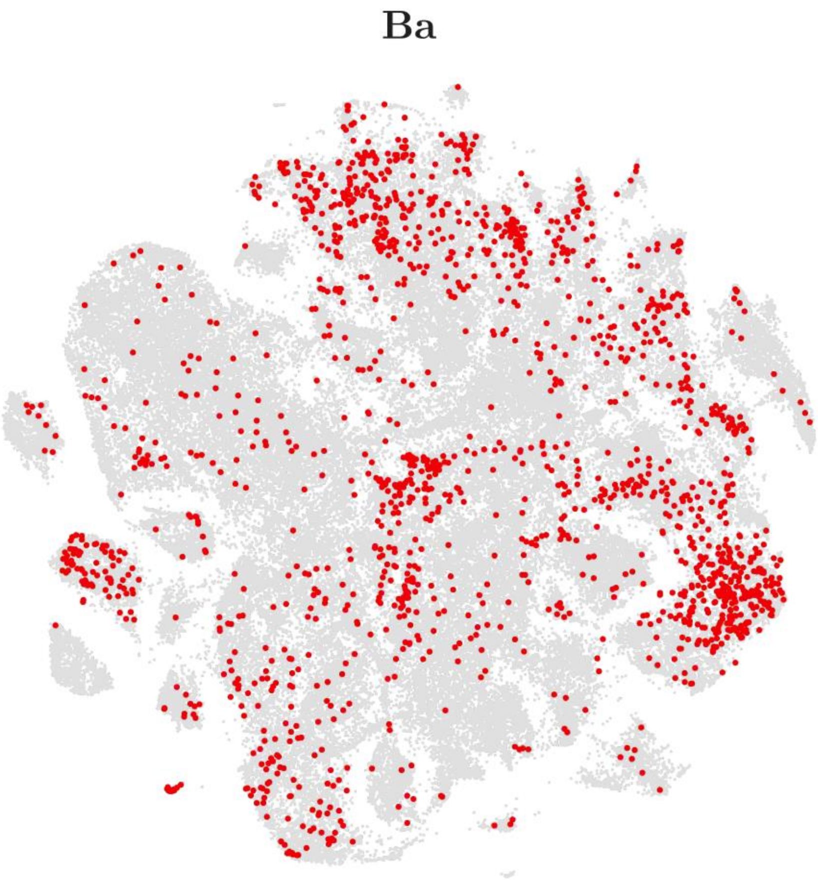

Figure S118. Caption Cluster plot for Lanthanum.

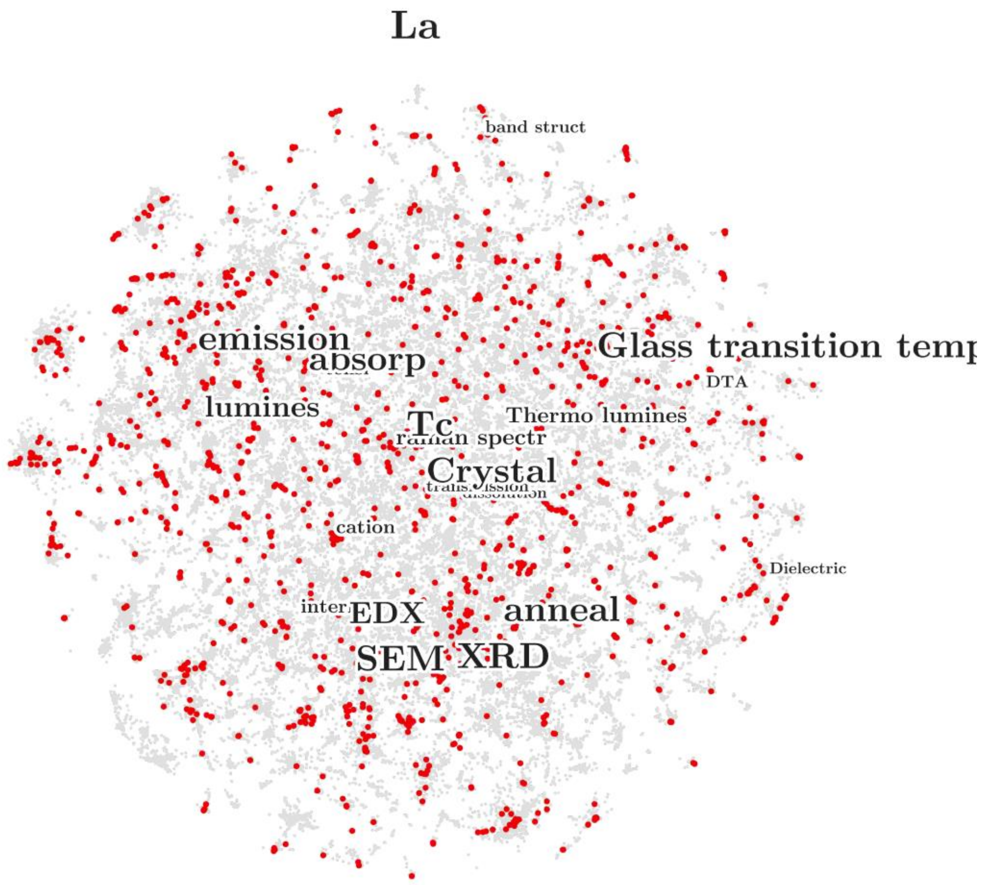

Figure S119. Latent Dirichlet Allocation plot for Lanthanum.

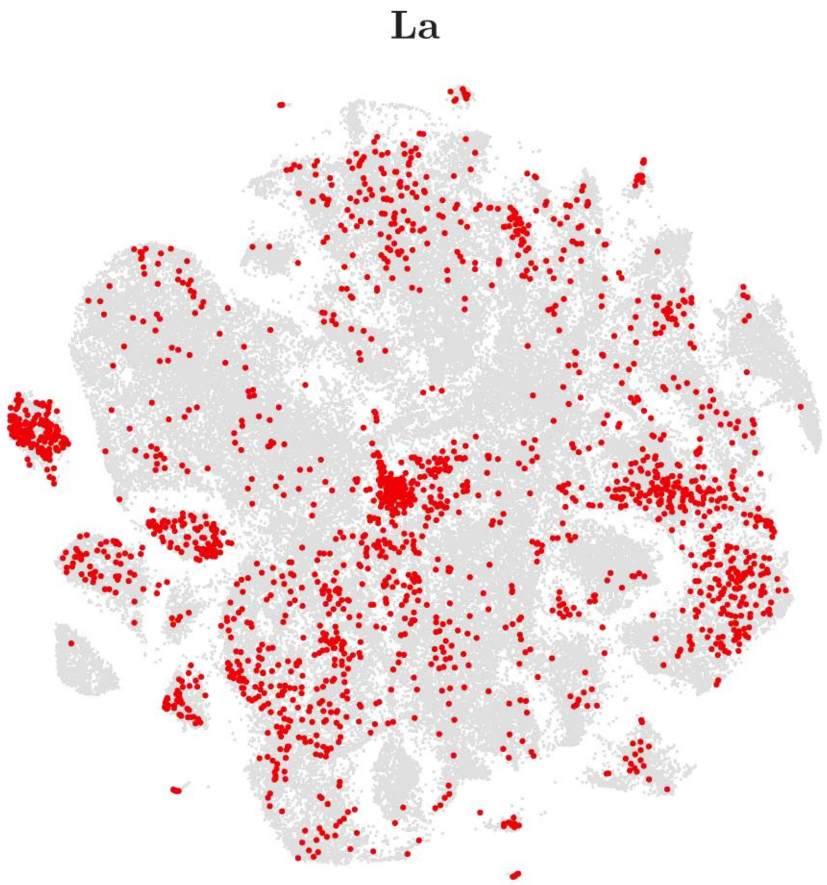

Figure S120. Caption Cluster plot for Cerium.

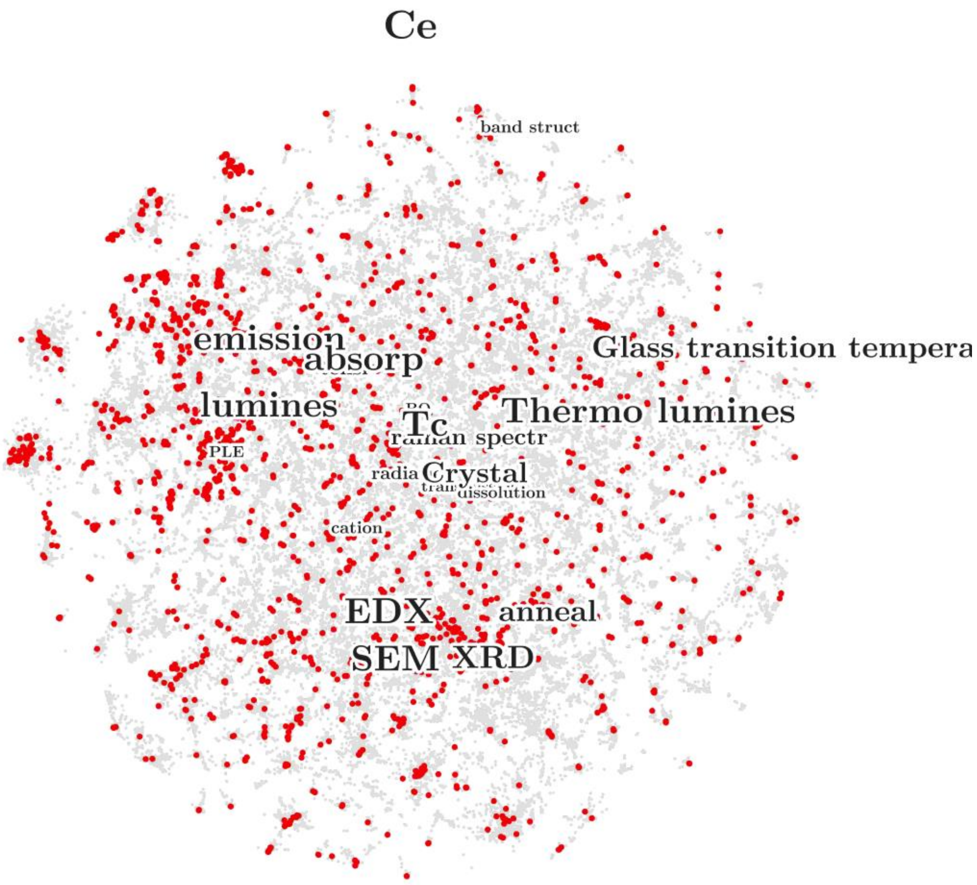

Figure S121. Latent Dirichlet Allocation plot for Cerium.

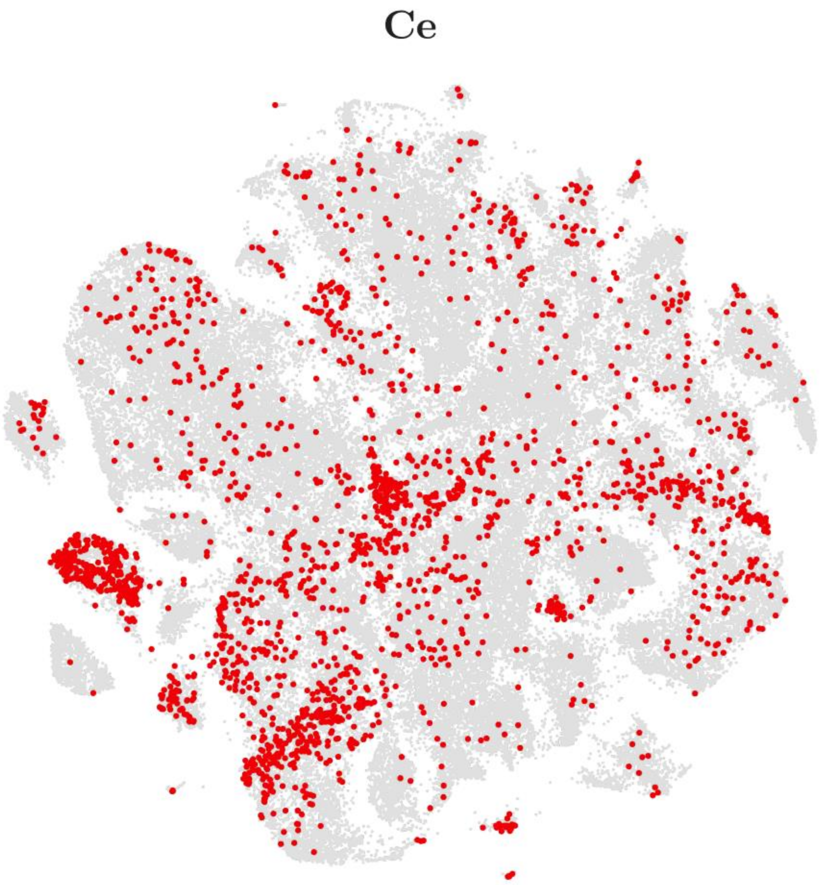

Figure S122. Caption Cluster plot for Praseodymium.

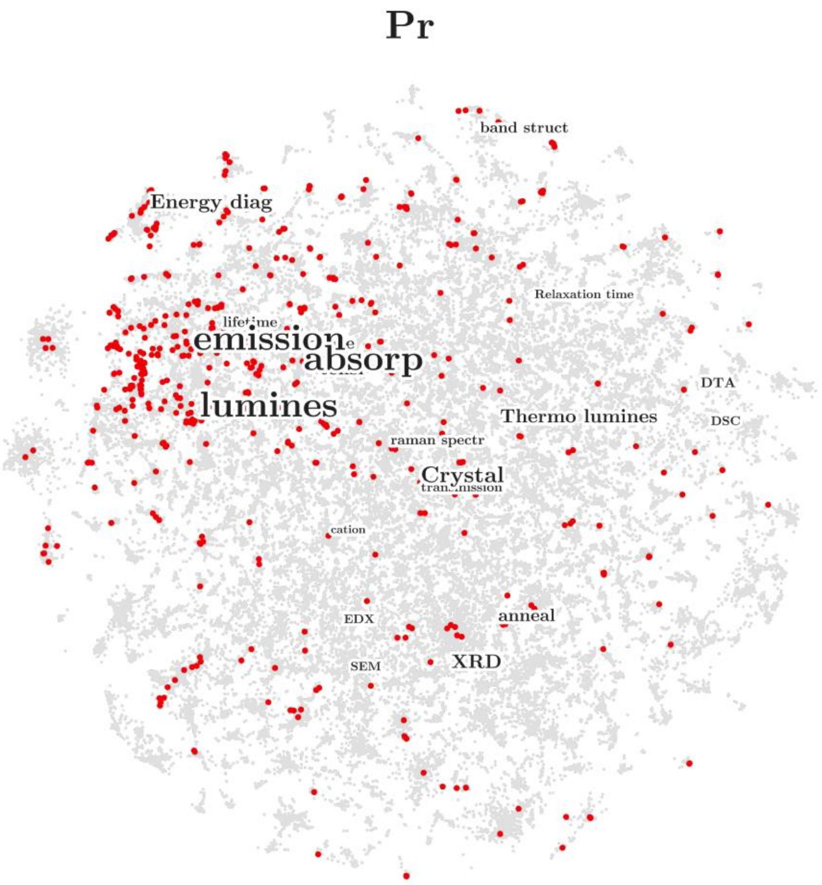

Figure S123. Latent Dirichlet Allocation plot for Praseodymium.

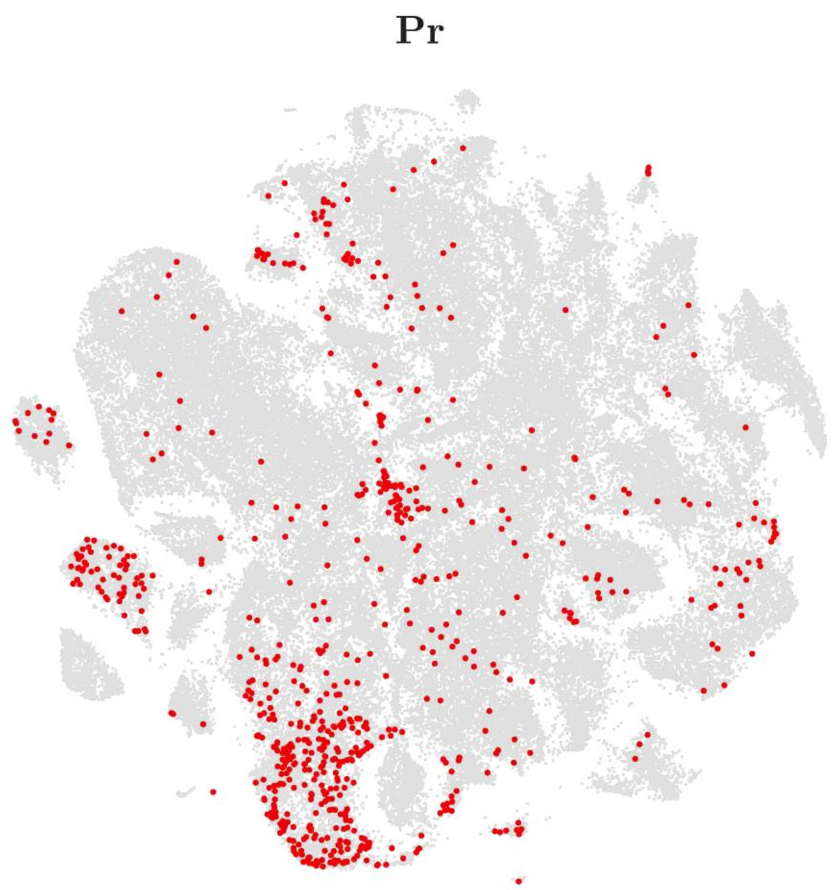

Figure S124. Caption Cluster plot for Neodymium.

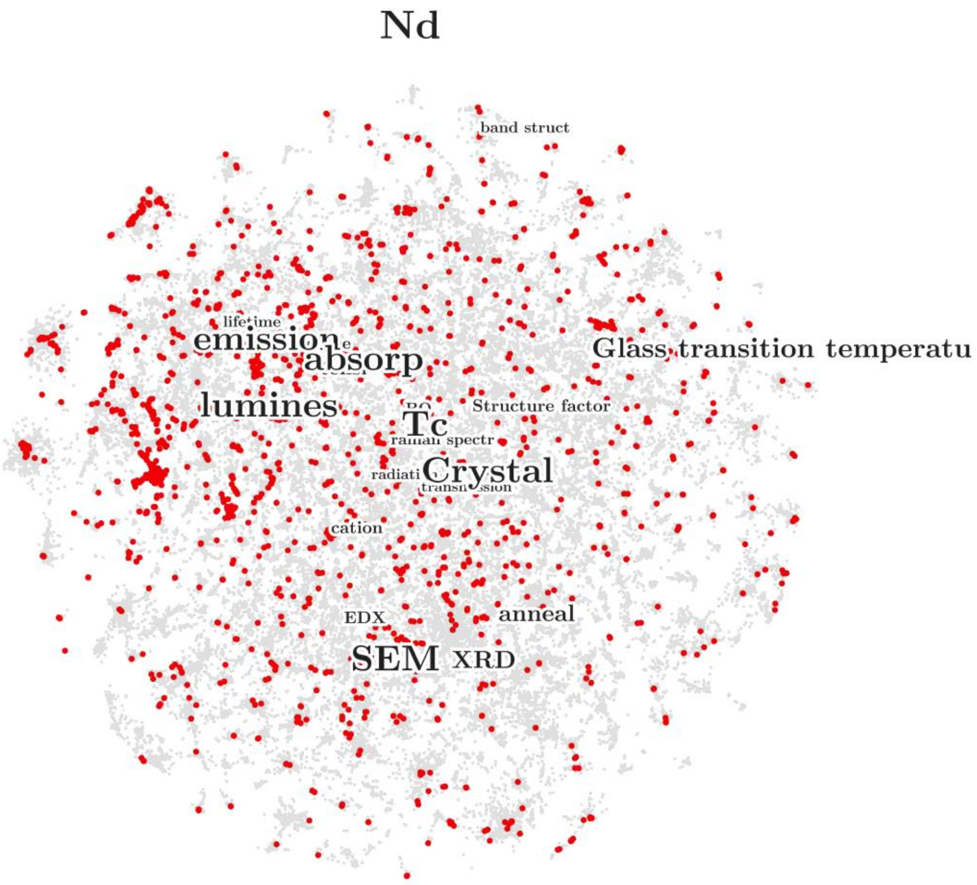

Figure S125. Latent Dirichlet Allocation plot for Neodymium.

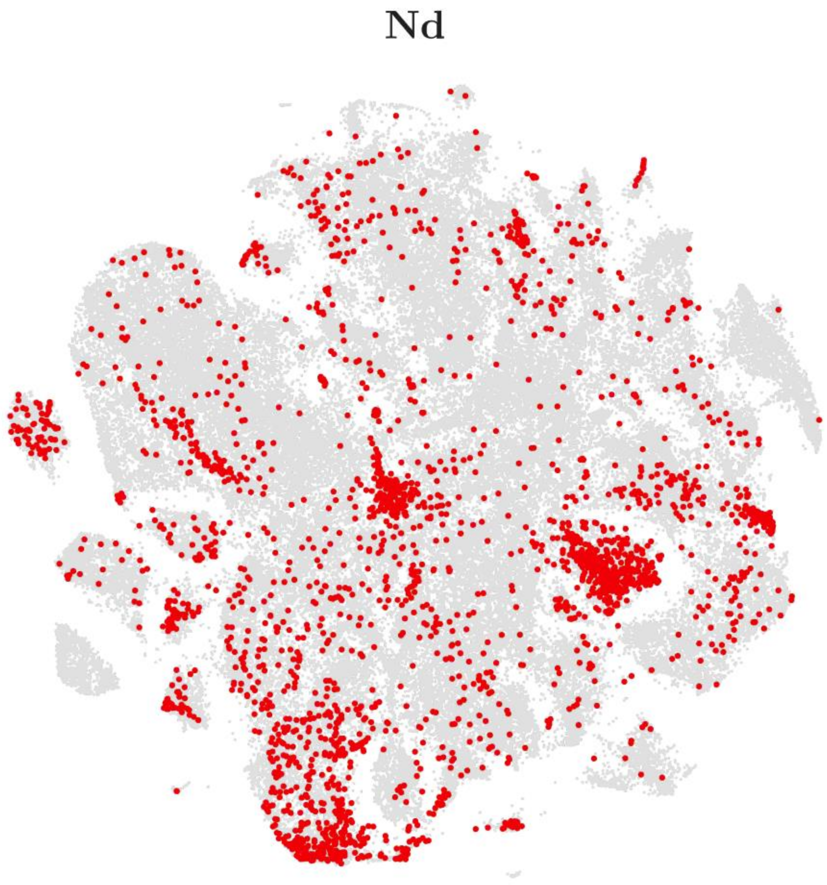

Figure S126. Caption Cluster plot for Promethium.

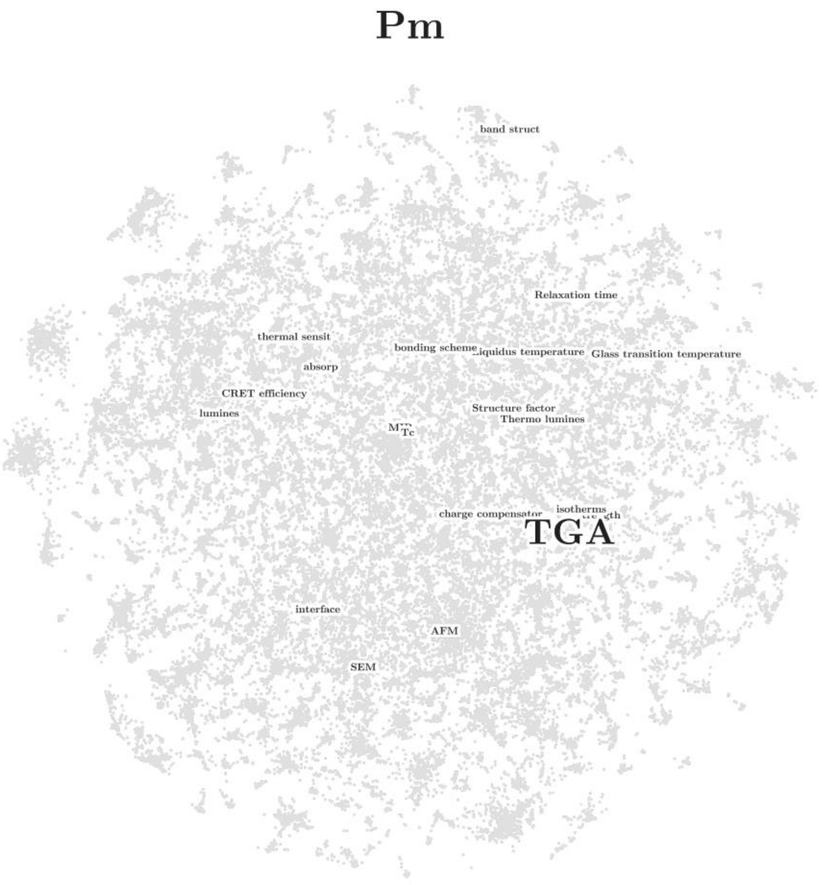

Figure S127. Latent Dirichlet Allocation plot for Promethium.

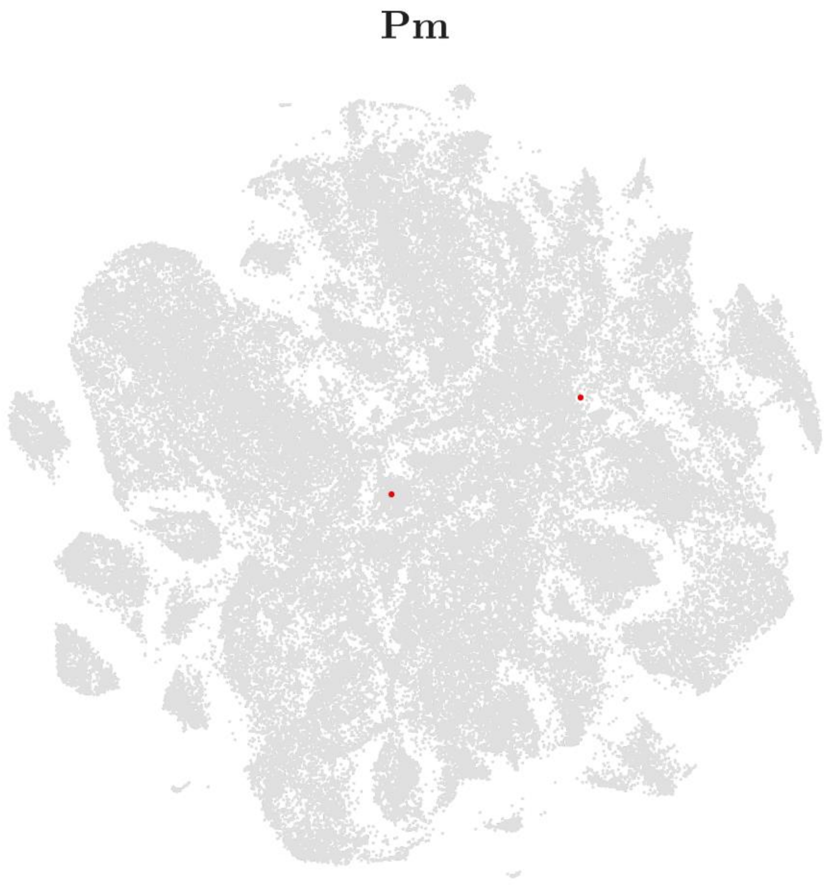

Figure S128. Caption Cluster plot for Samarium.

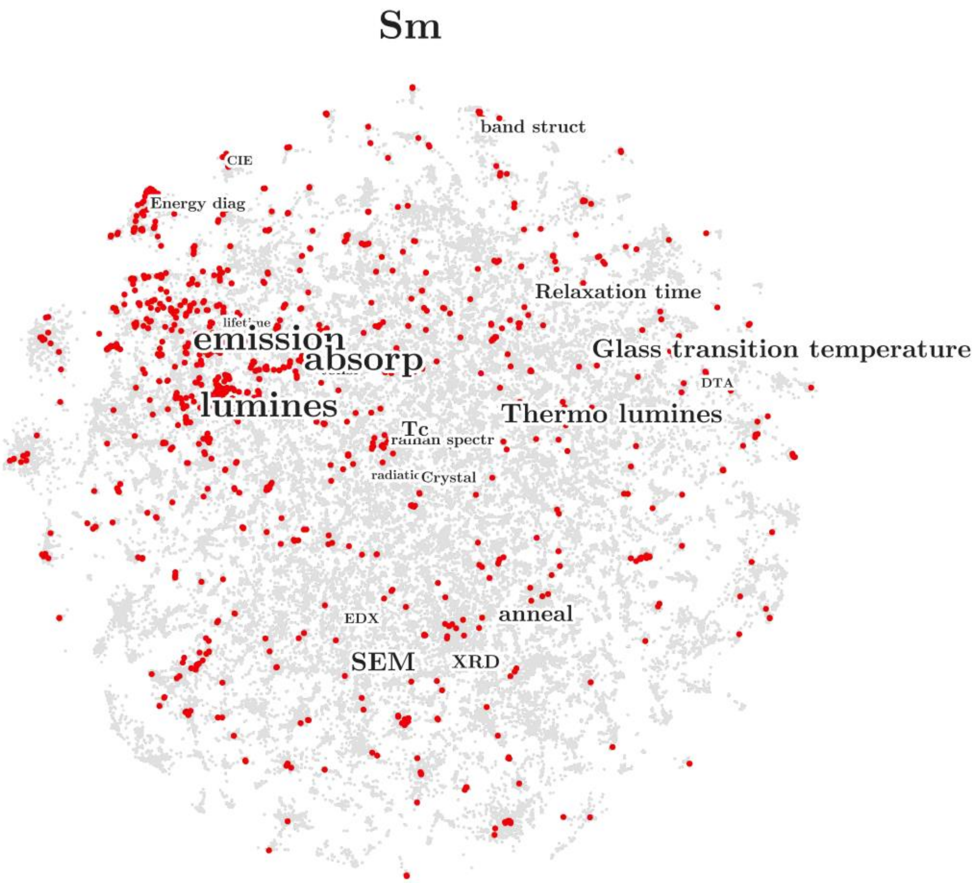

Figure S129. Latent Dirichlet Allocation plot for Samarium.

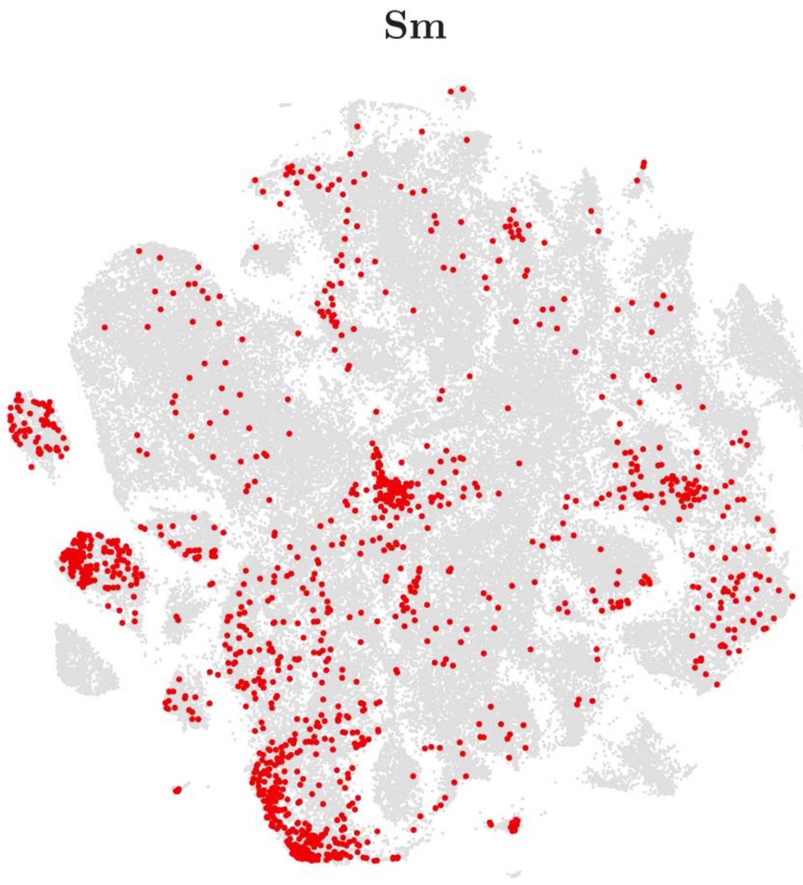

Figure S130. Caption Cluster plot for Europium.

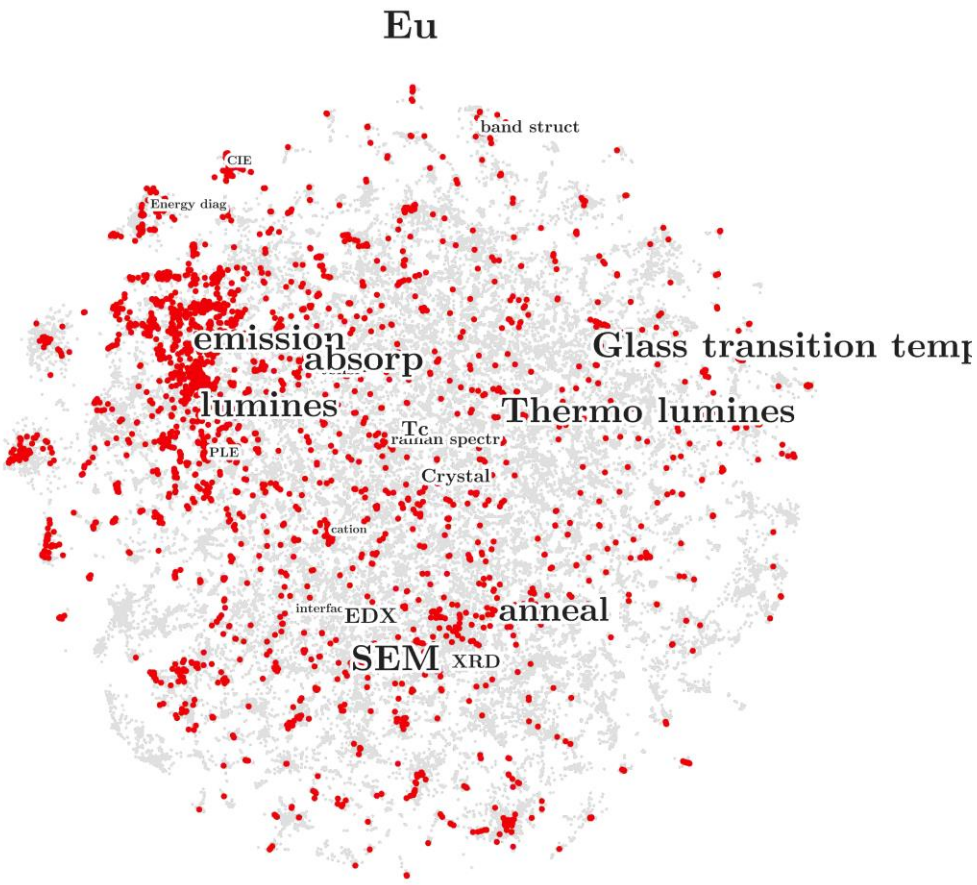

Figure S131. Latent Dirichlet Allocation plot for Europium.

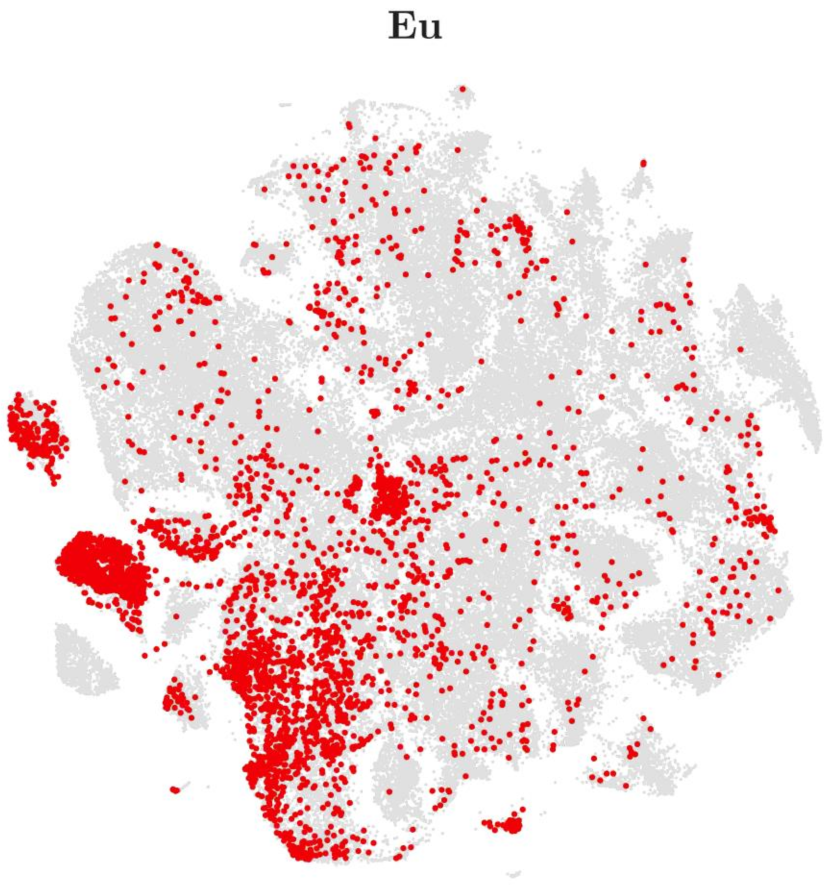

Figure S132. Caption Cluster plot for Gadolinium.

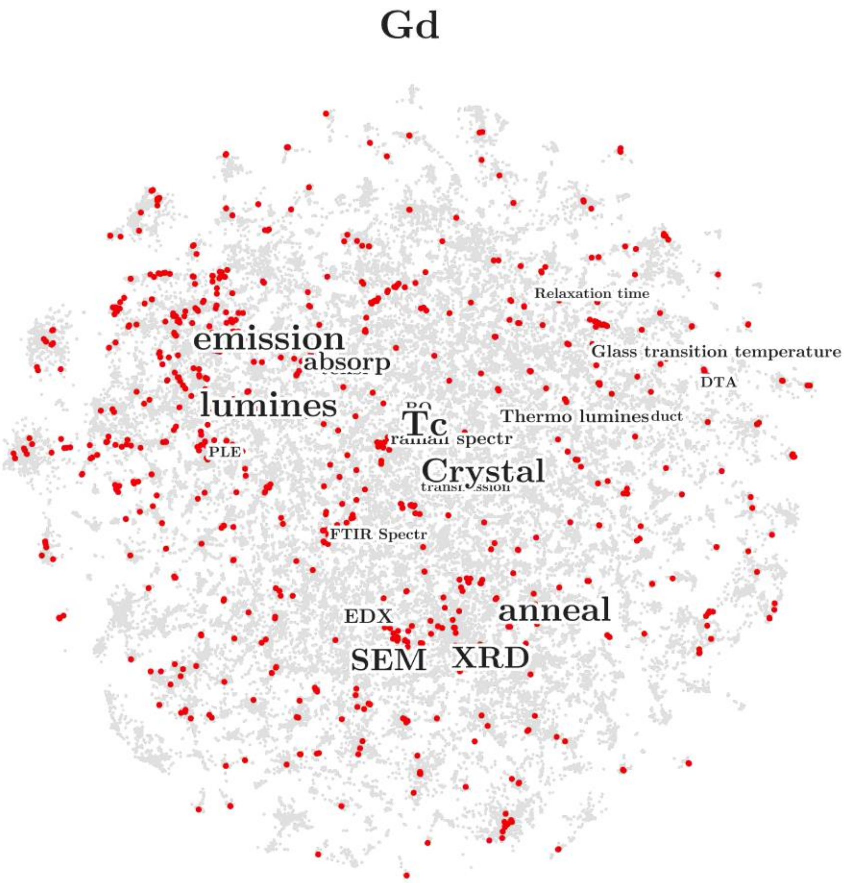

Figure S133. Latent Dirichlet Allocation plot for Gadolinium.

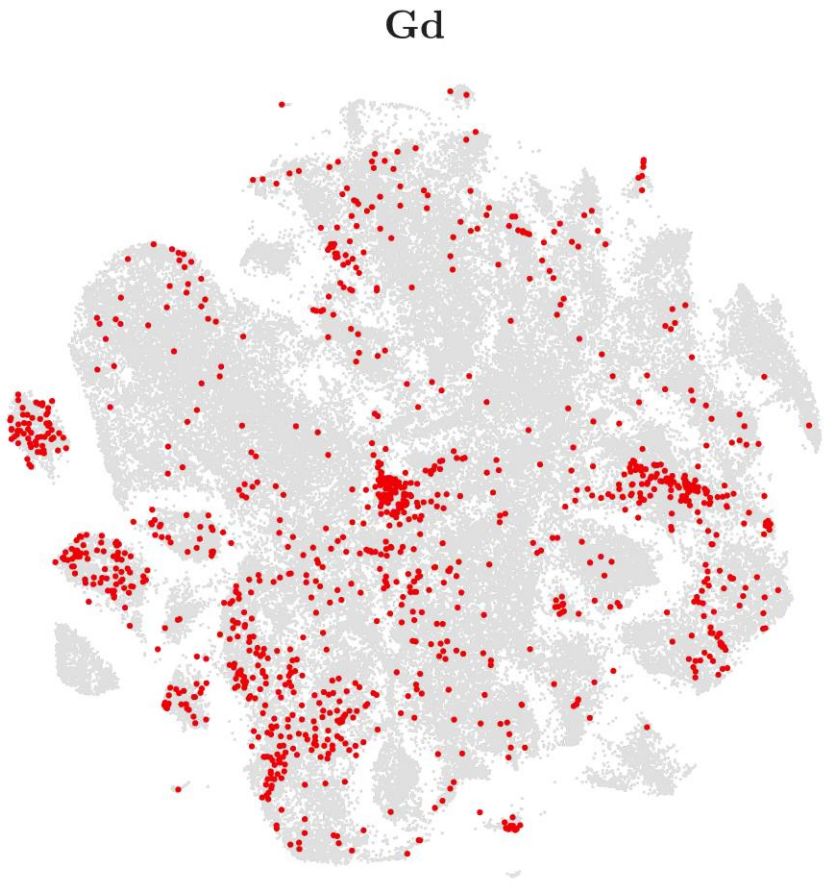

Figure S134. Caption Cluster plot for Terbium.

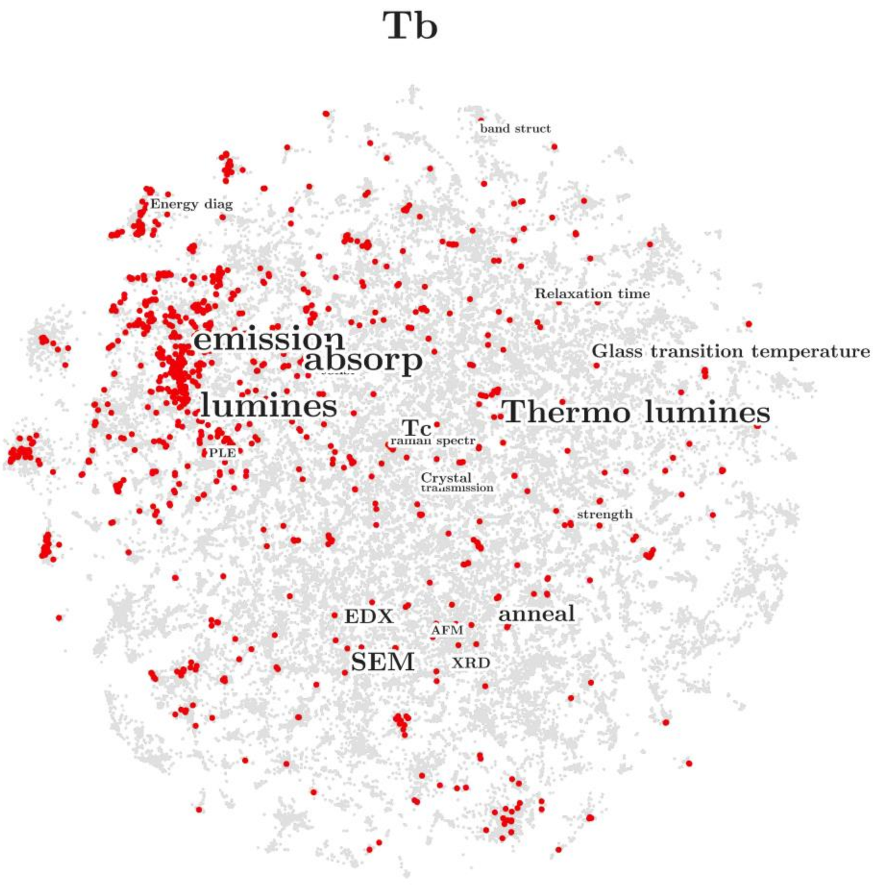

Figure S135. Latent Dirichlet Allocation plot for Terbium.

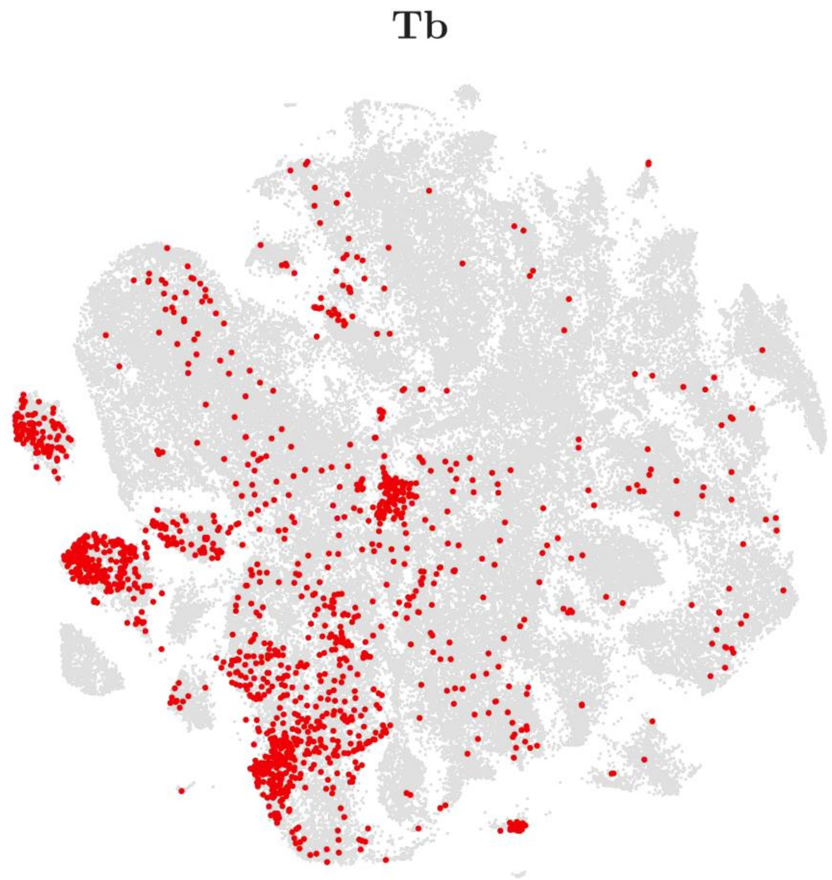

Figure S136. Caption Cluster plot for Dysprosium.

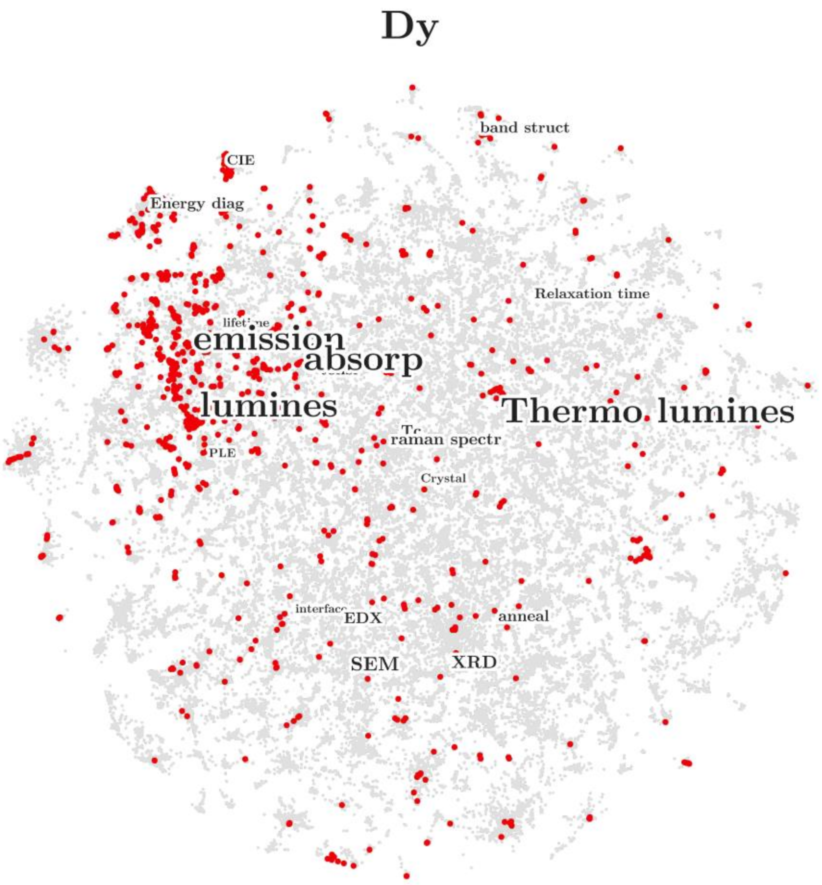

Figure S137. Latent Dirichlet Allocation plot for Dysprosium.

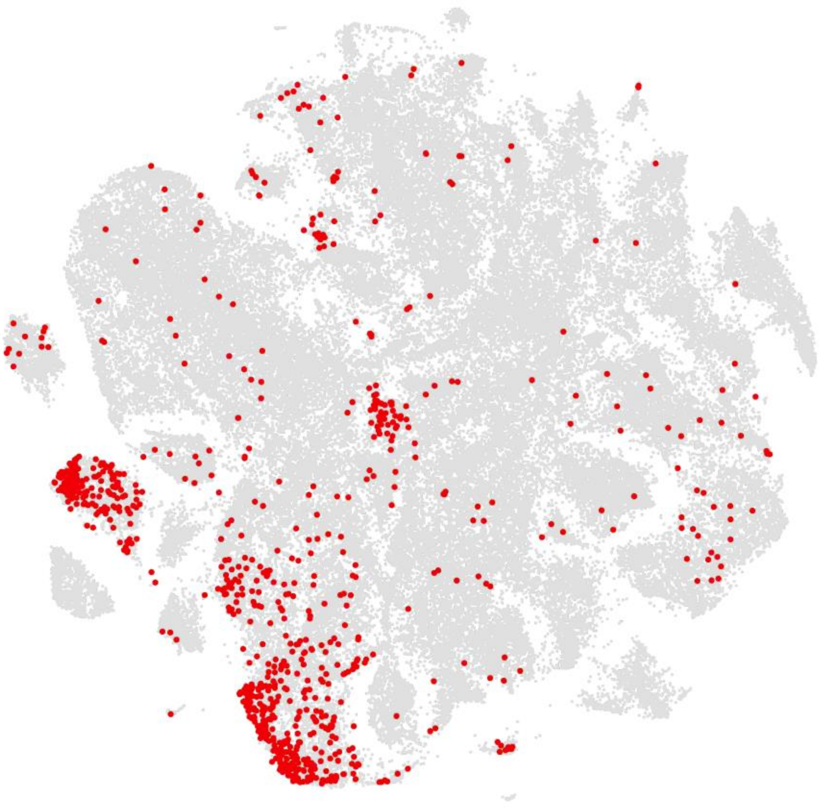

Figure S138. Caption Cluster plot for Holmium.

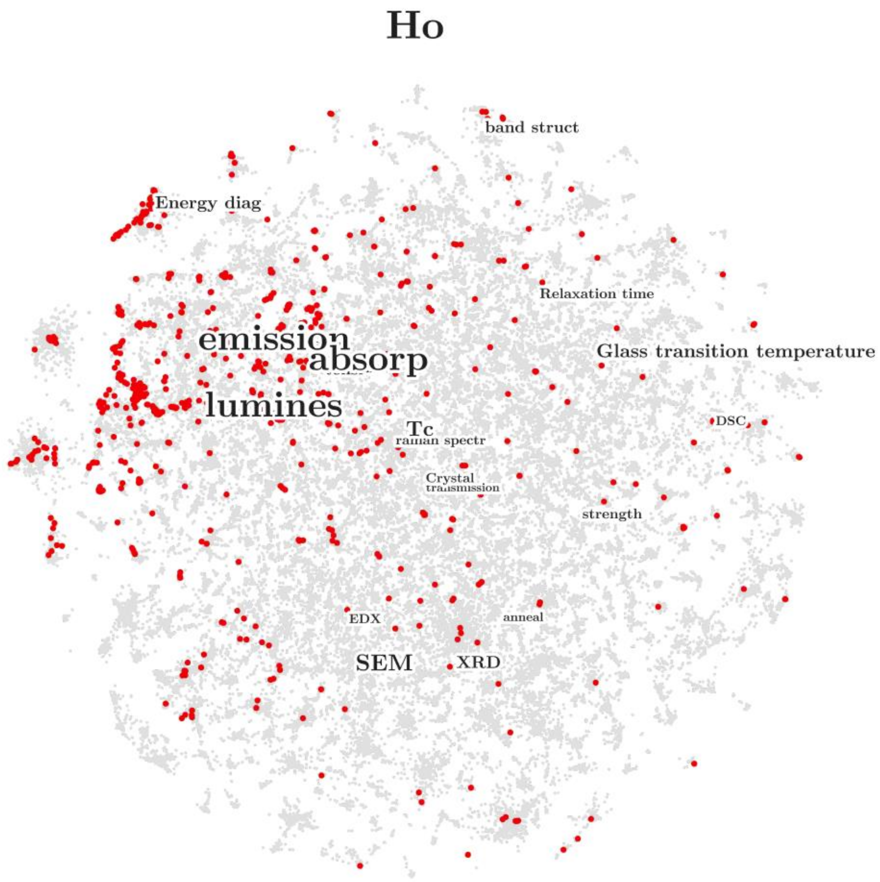

Figure S139. Latent Dirichlet Allocation plot for Holmium.

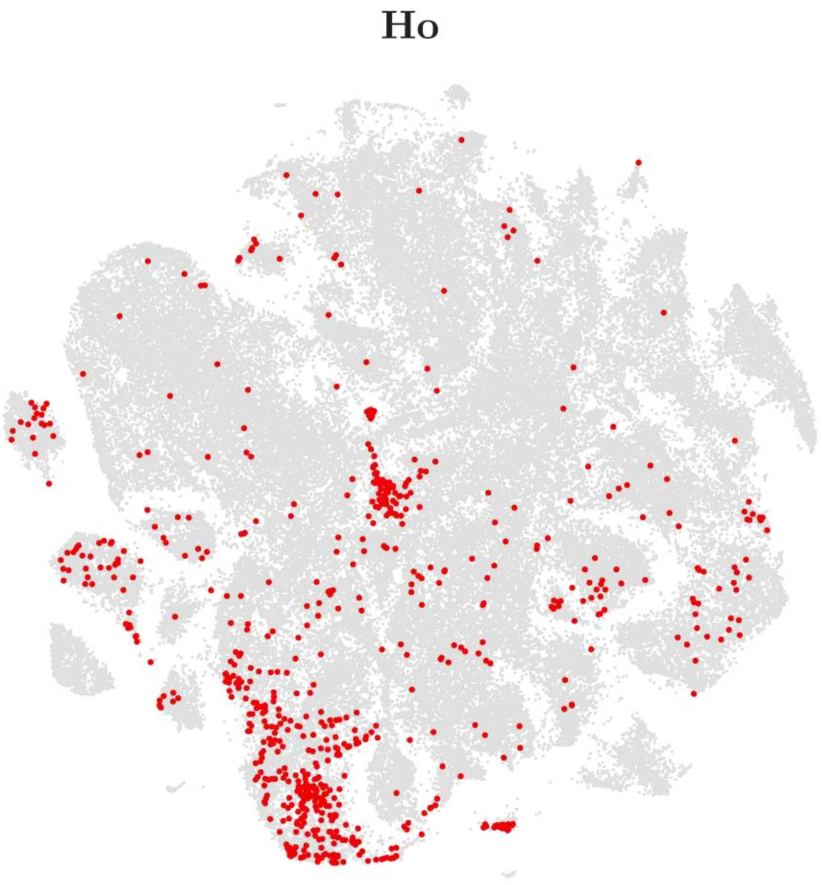

Figure S140. Caption Cluster plot for Erbium.

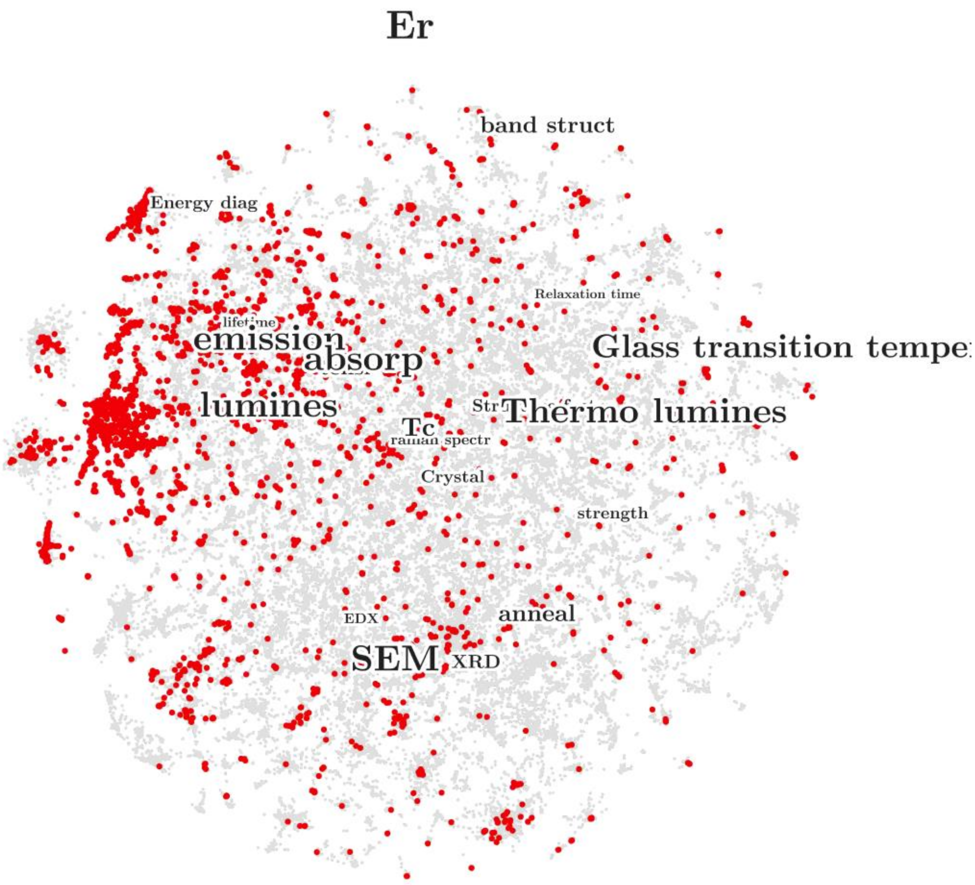

Figure S141. Latent Dirichlet Allocation plot for Erbium.

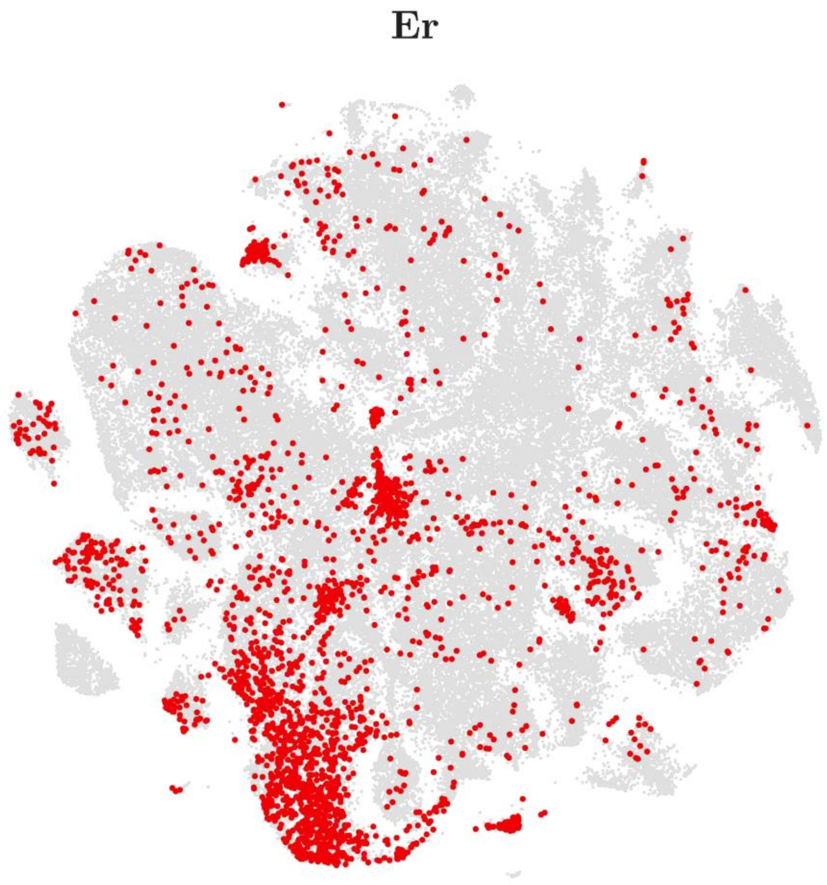

Figure S142. Caption Cluster plot for Thulium.

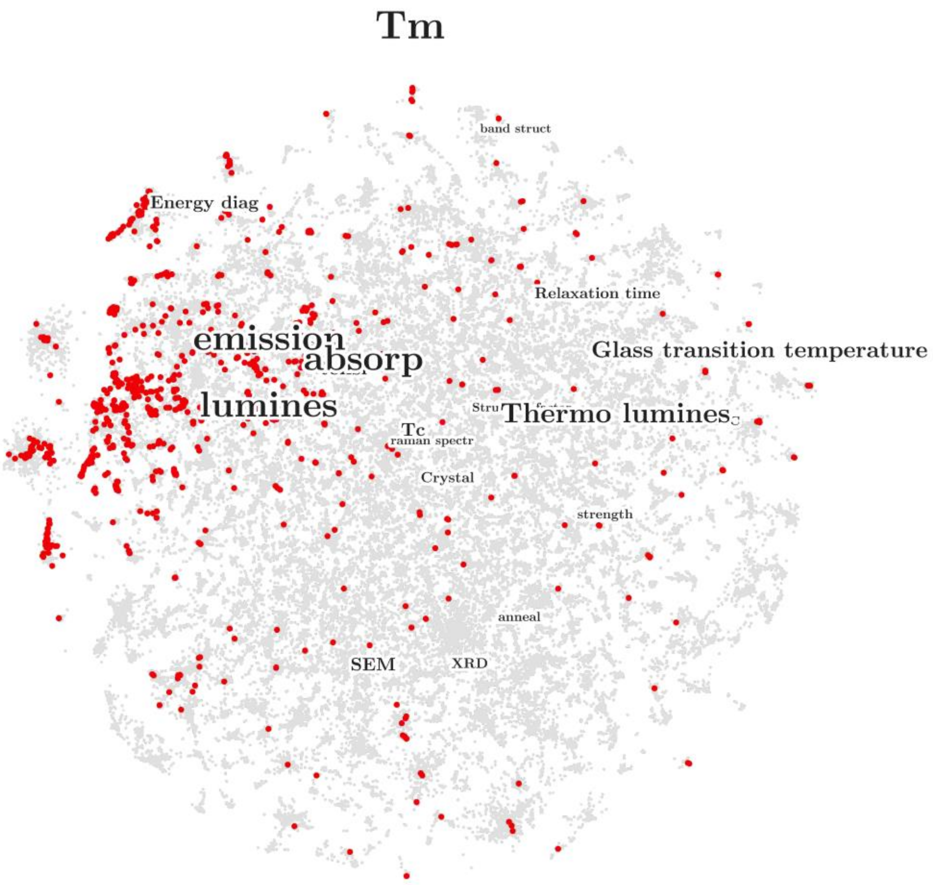

Figure S143. Latent Dirichlet Allocation plot for Thulium.

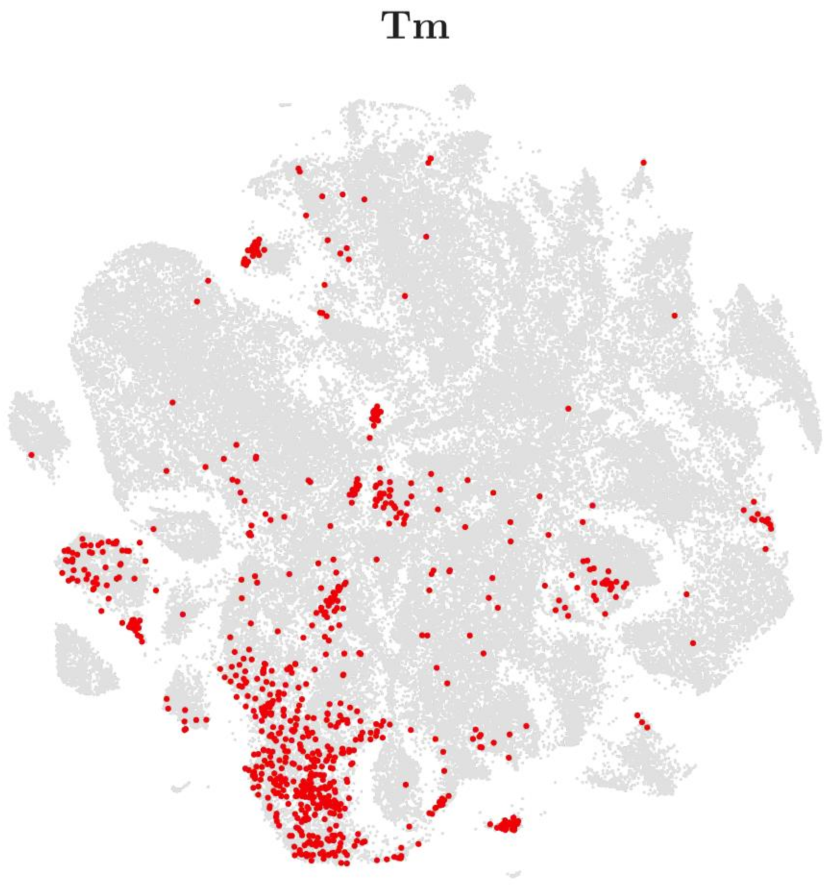

Figure S144. Caption Cluster plot for Ytterbium.

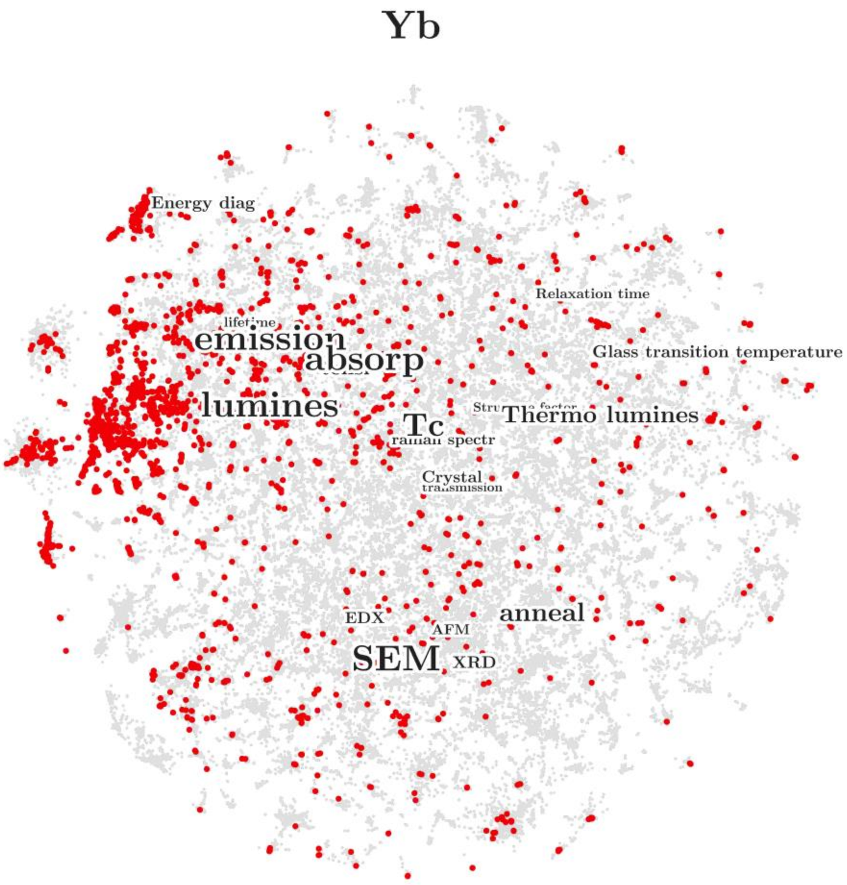

Figure S145. Latent Dirichlet Allocation plot for Ytterbium.

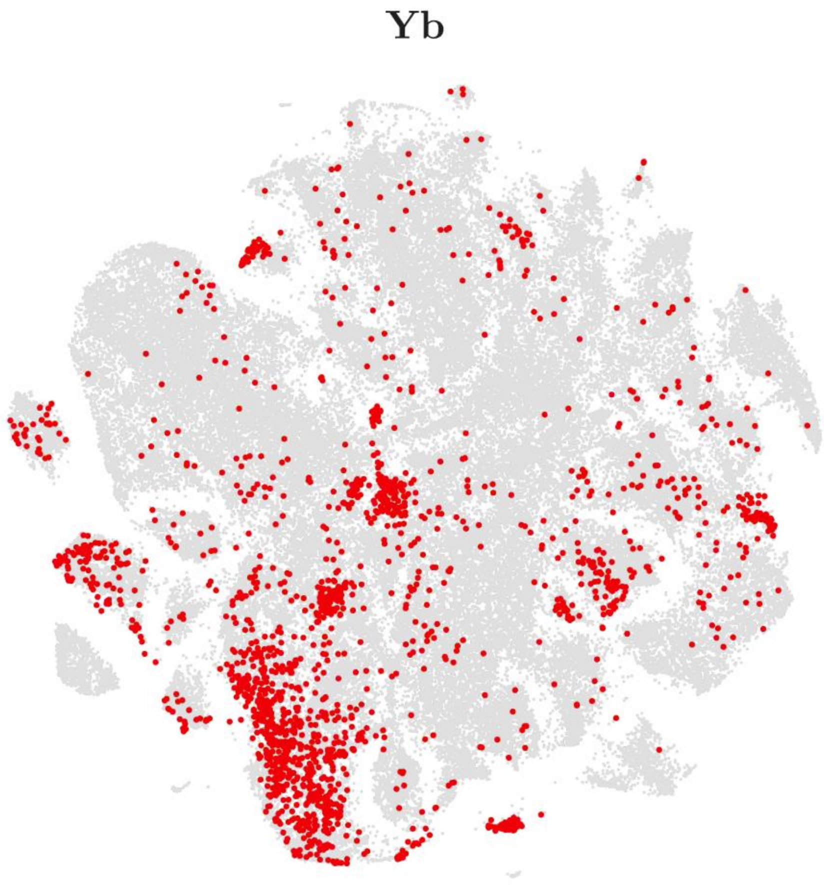

Figure S146. Caption Cluster plot for Lutetium.

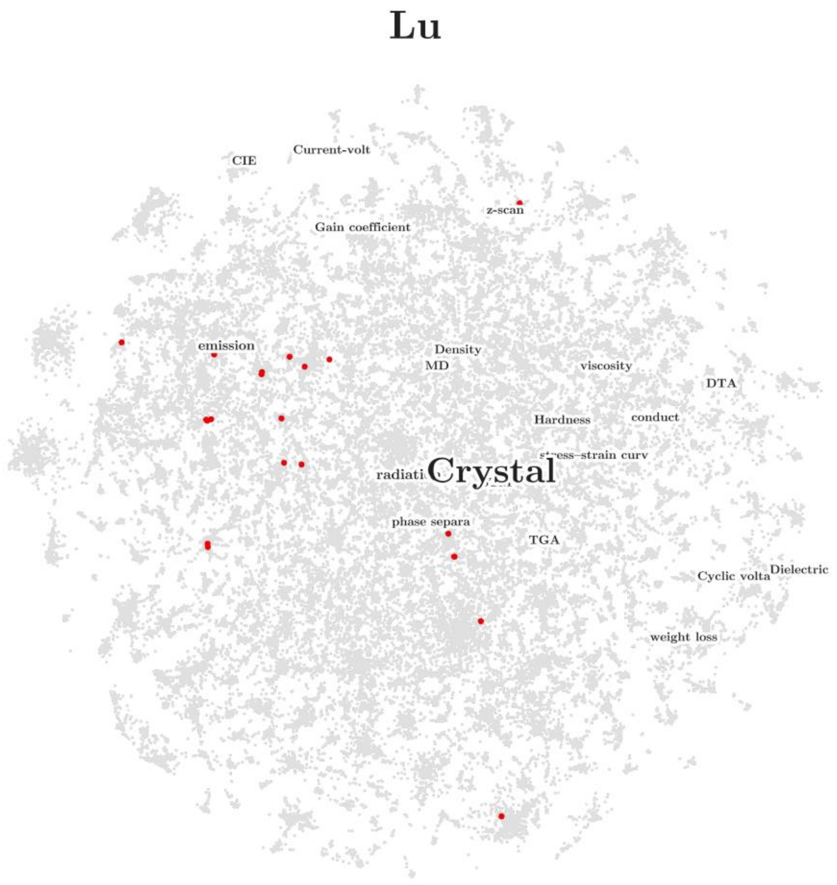

Figure S147. Latent Dirichlet Allocation plot for Lutetium.

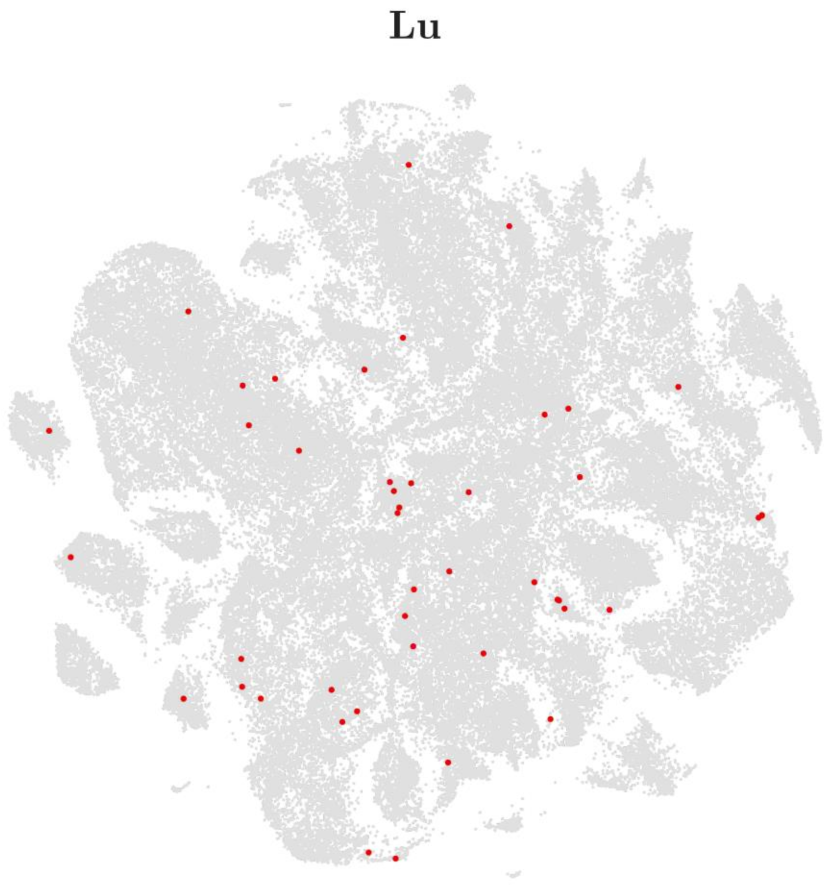

Figure S148. Caption Cluster plot for Hafnium.

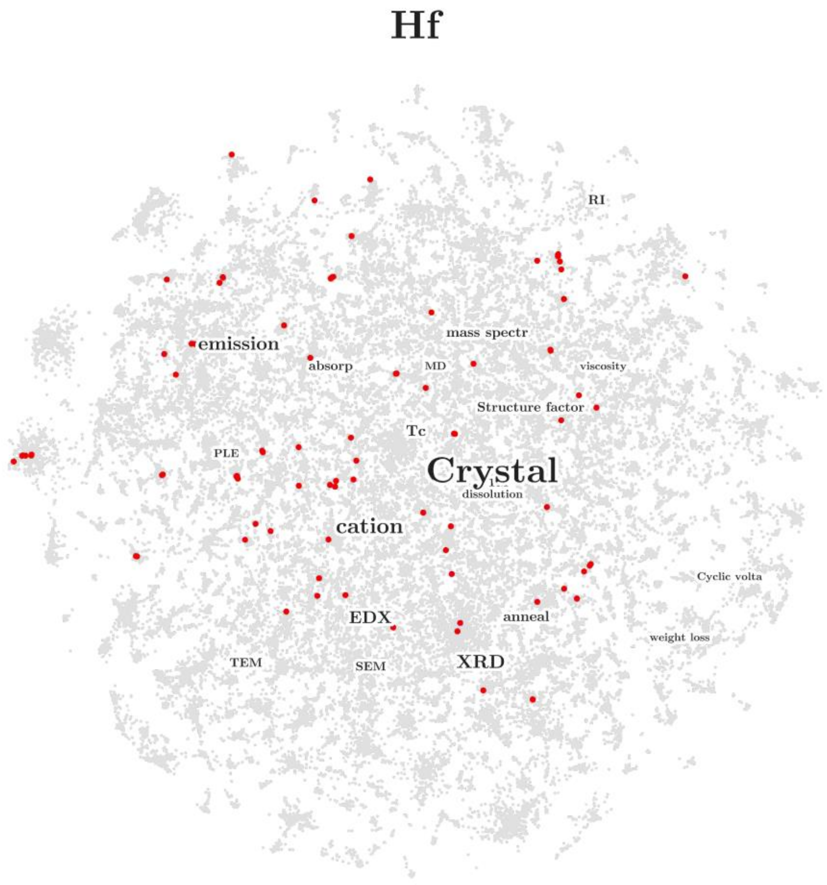

Figure S149. Latent Dirichlet Allocation plot for Hafnium.

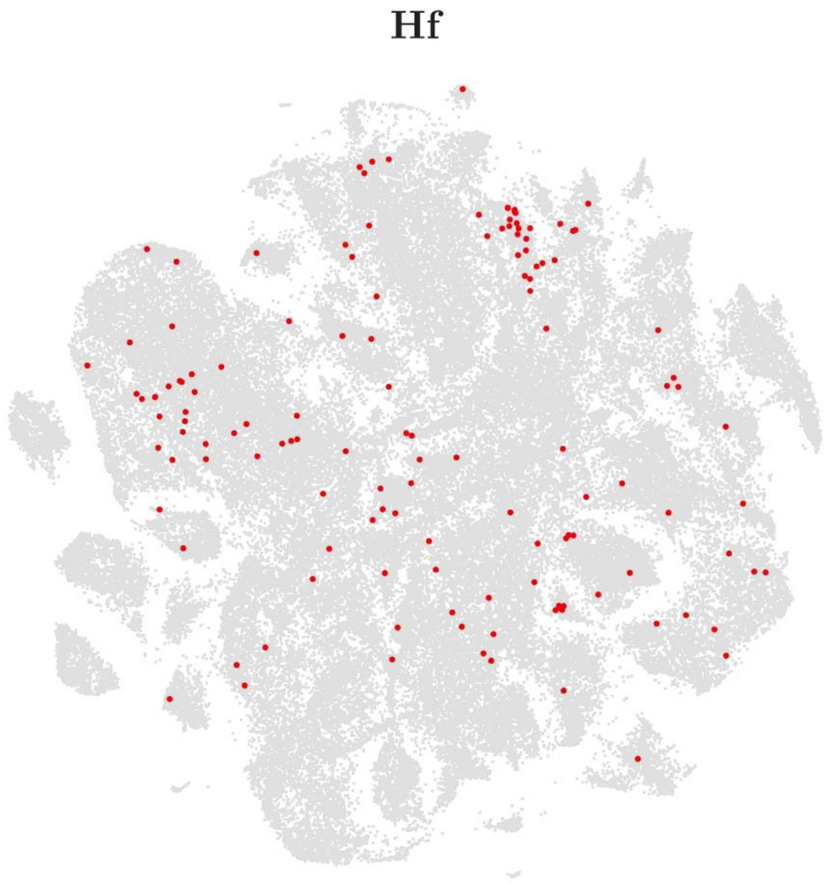

Figure S150. Caption Cluster plot for Tantalum.

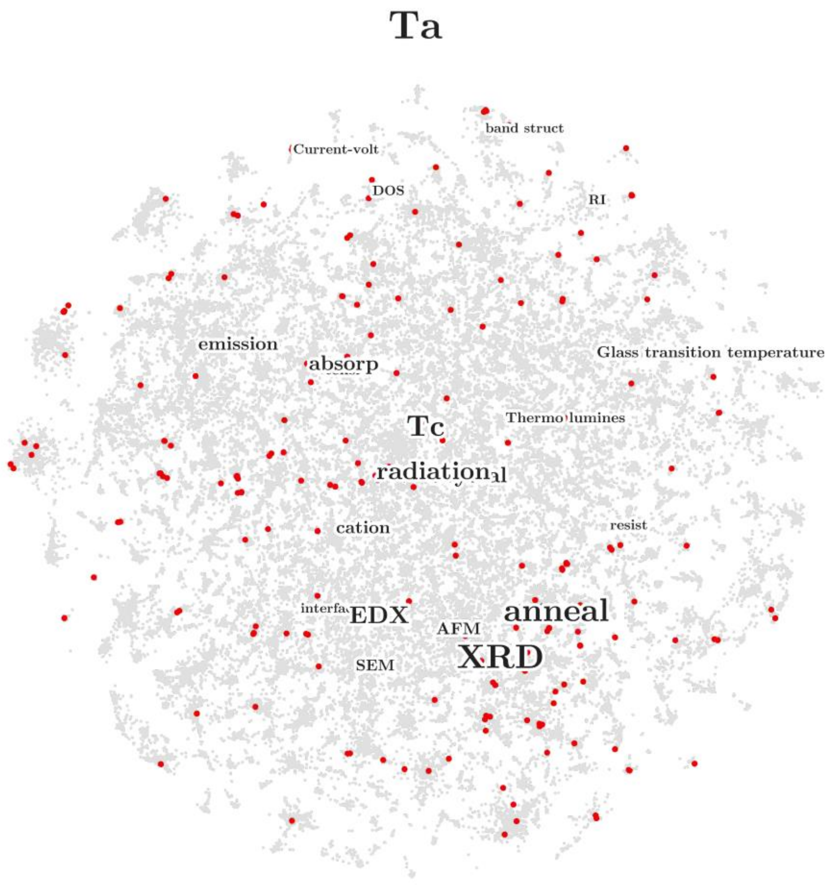

Figure S151. Latent Dirichlet Allocation plot for Tantalum.

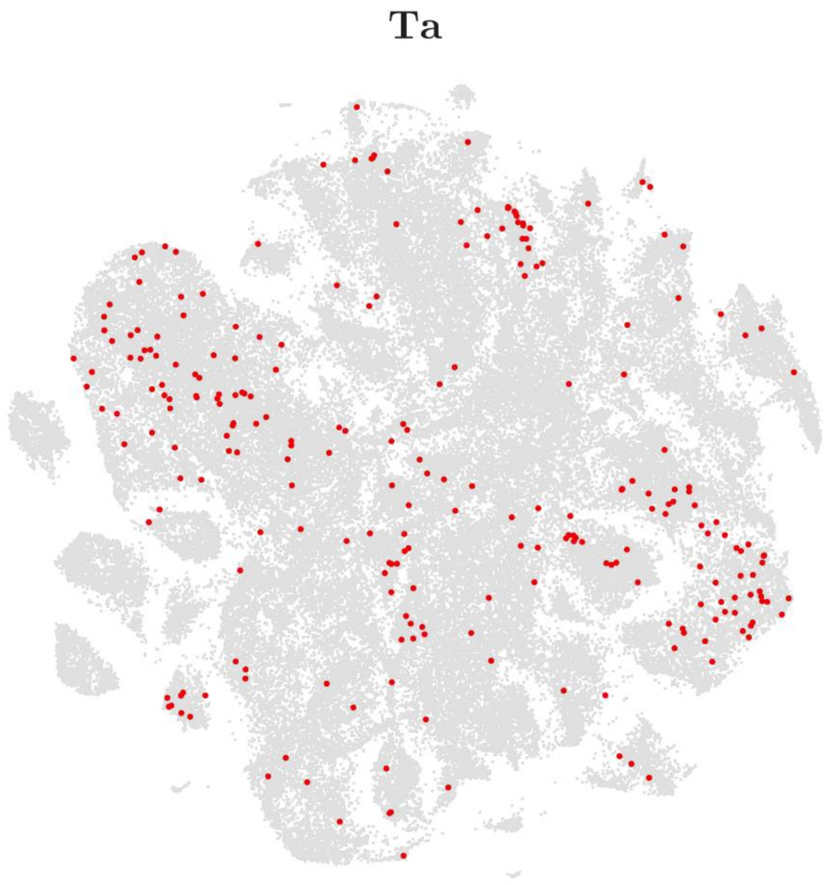

Figure S152. Caption Cluster plot for Tungsten.

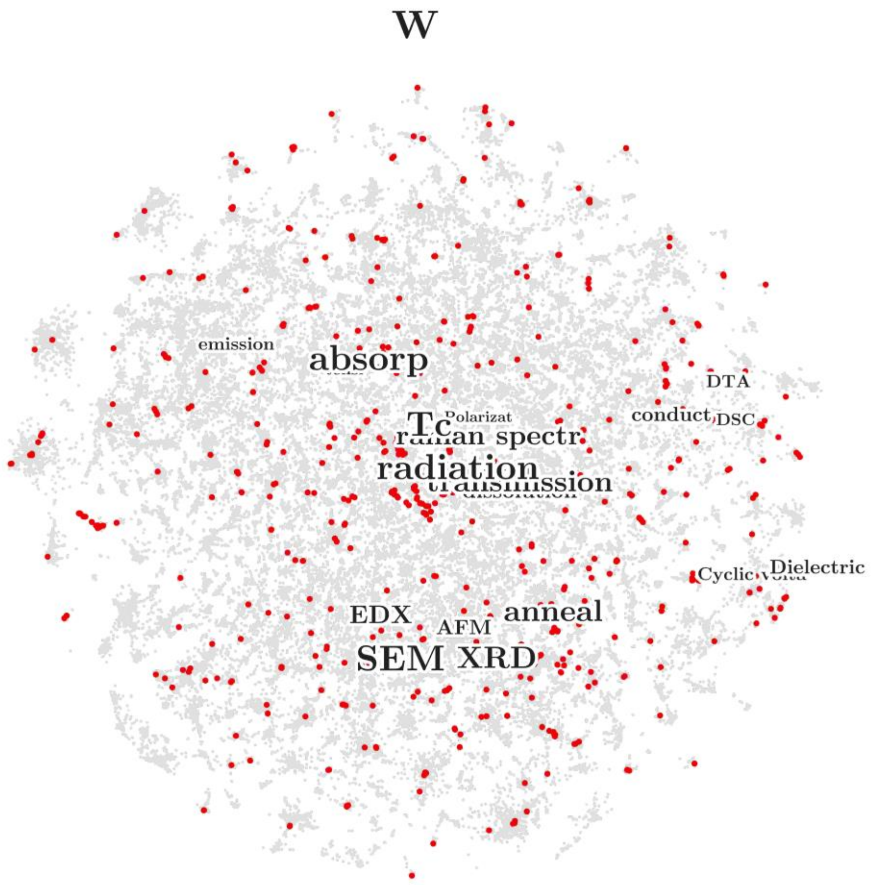

Figure S153. Latent Dirichlet Allocation plot for Tungsten.

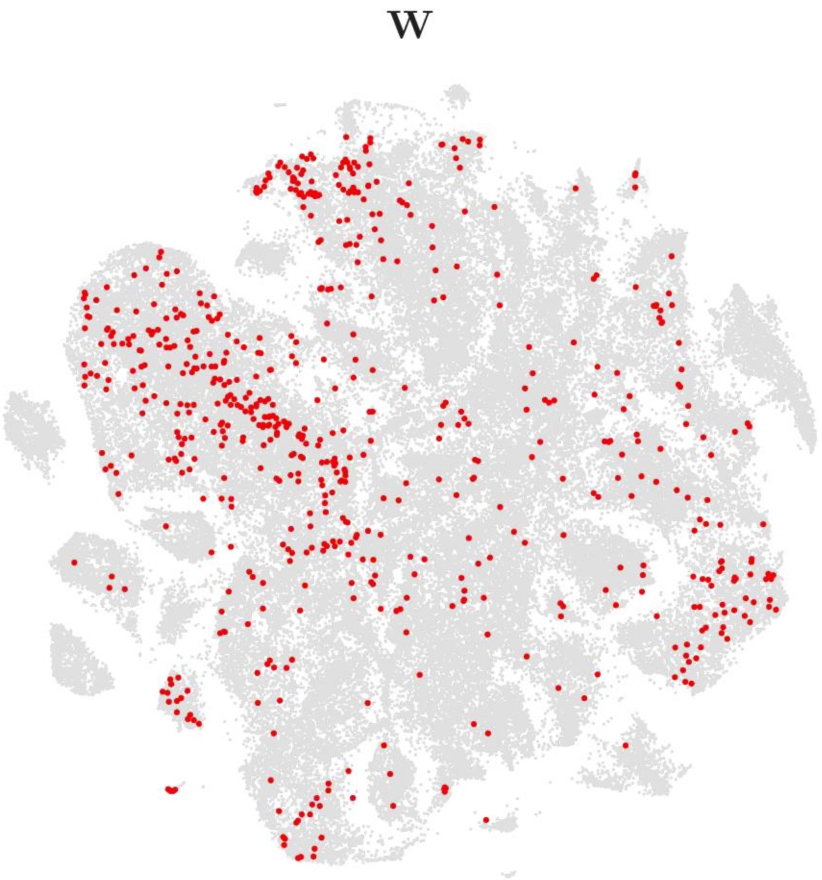

Figure S154. Caption Cluster plot for Rhenium.

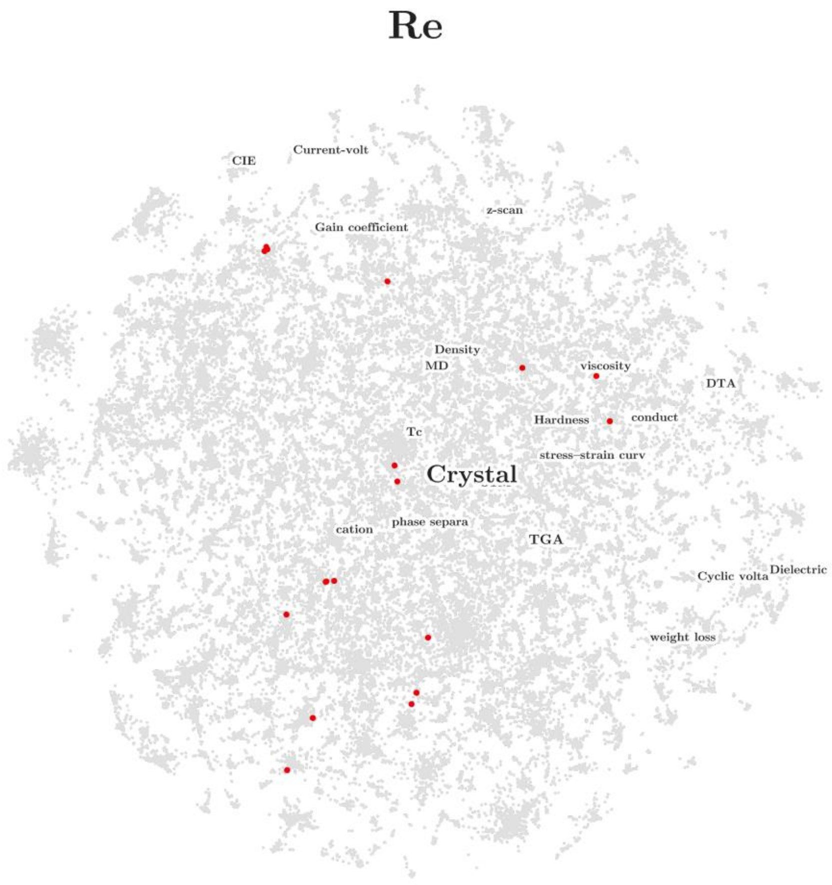

Figure S155. Latent Dirichlet Allocation plot for Rhenium.

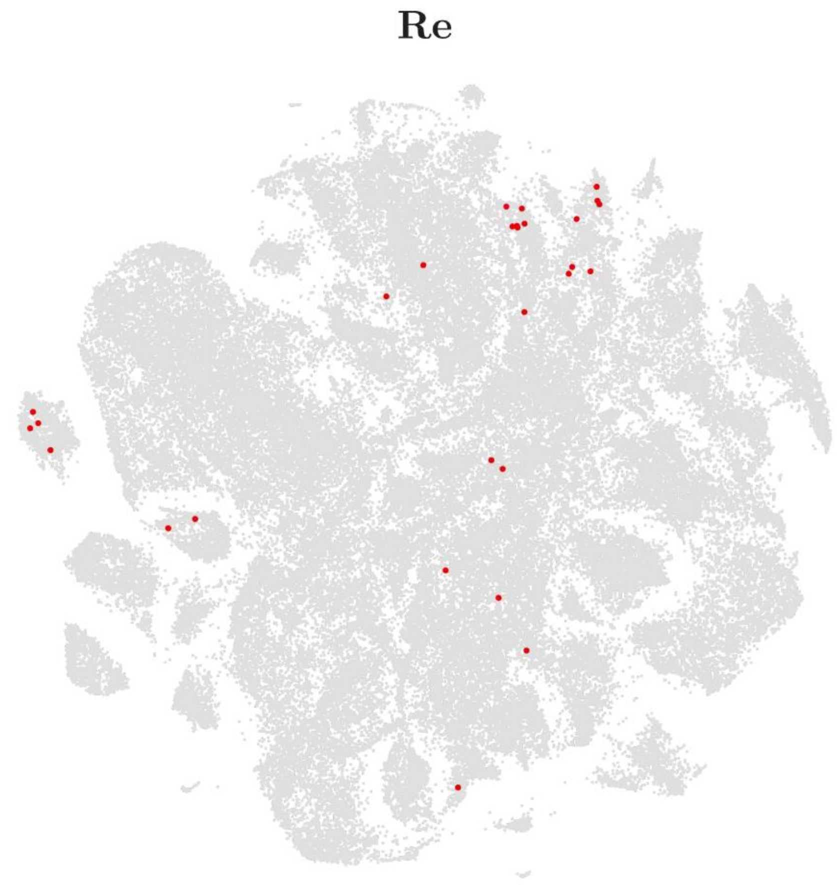

Figure S156. Caption Cluster plot for Osmium.

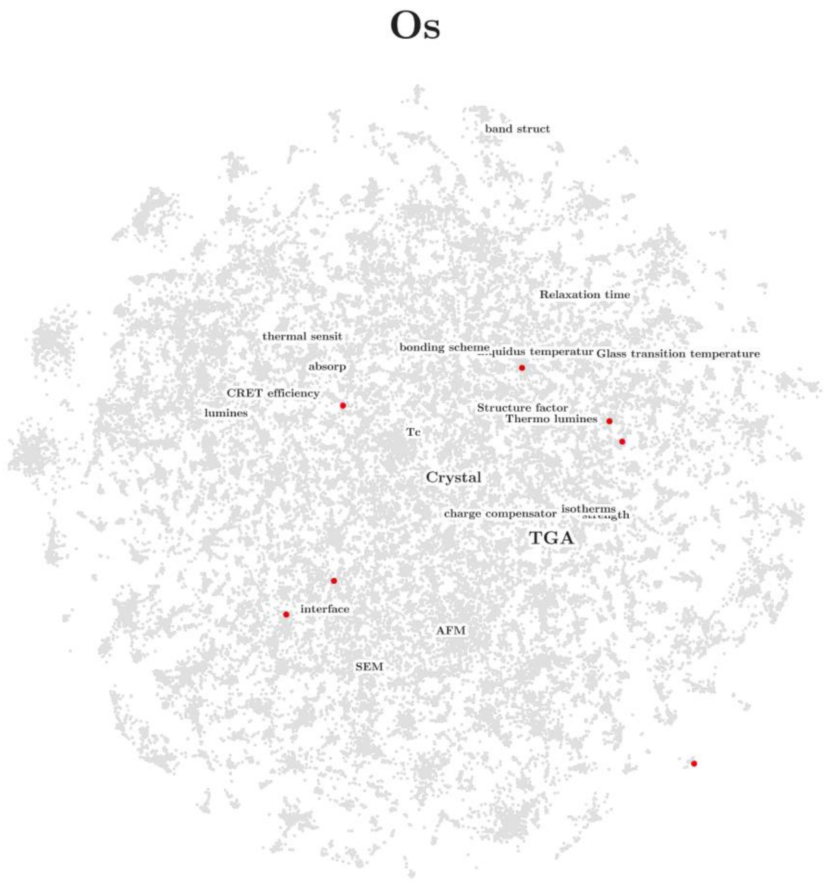

Figure S157. Latent Dirichlet Allocation plot for Osmium.

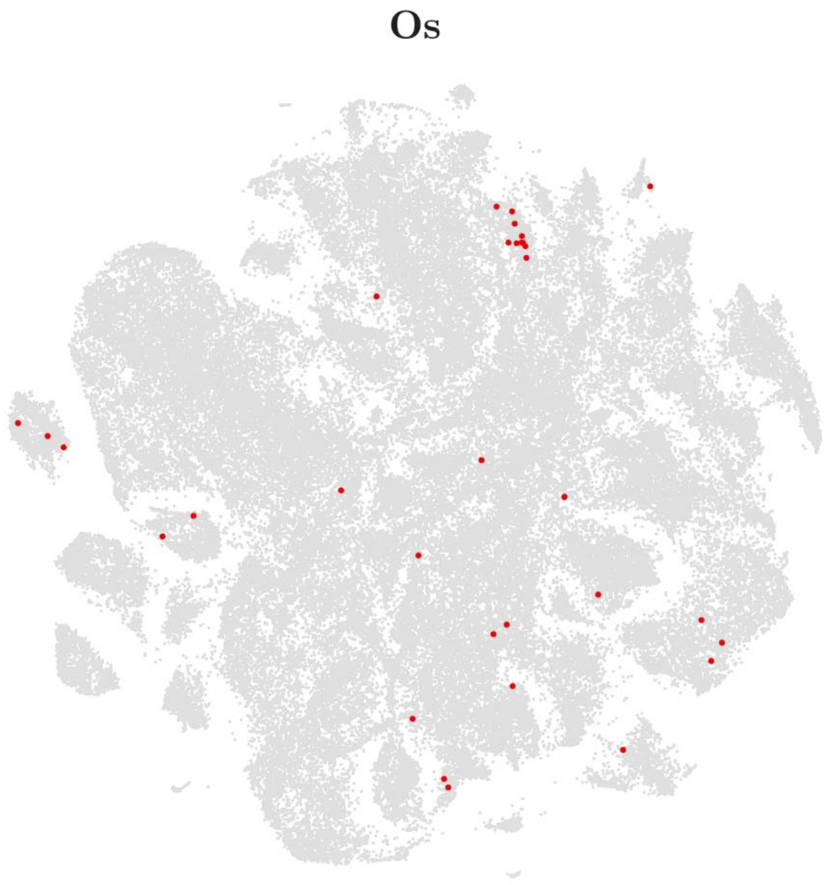

Figure S158. Caption Cluster plot for Iridium.

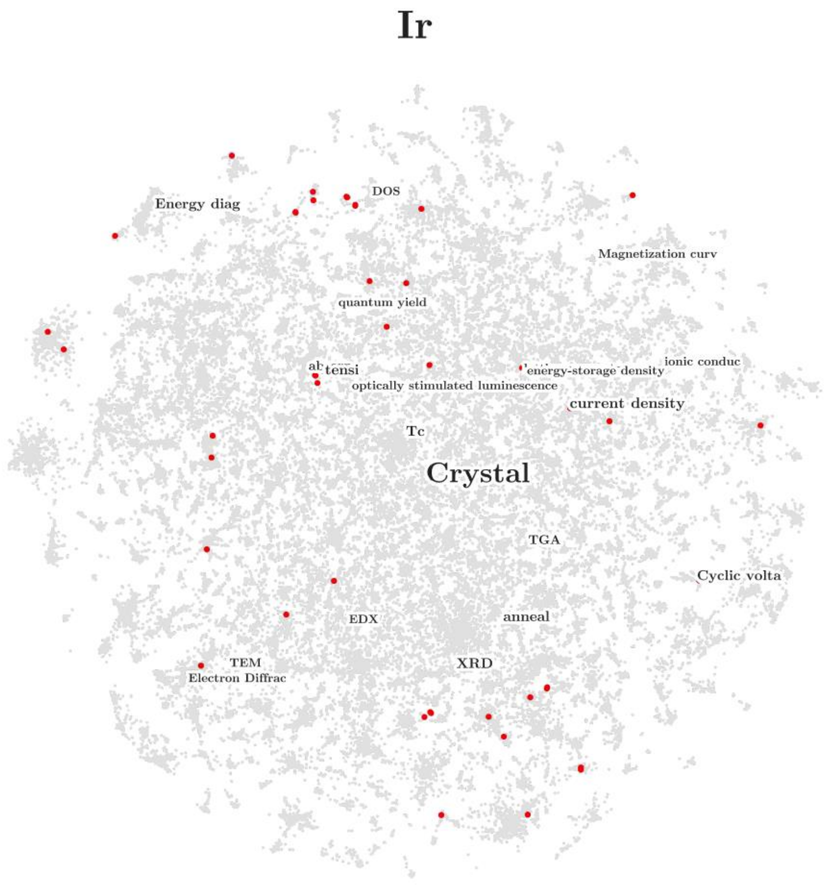

Figure S159. Latent Dirichlet Allocation plot for Iridium.

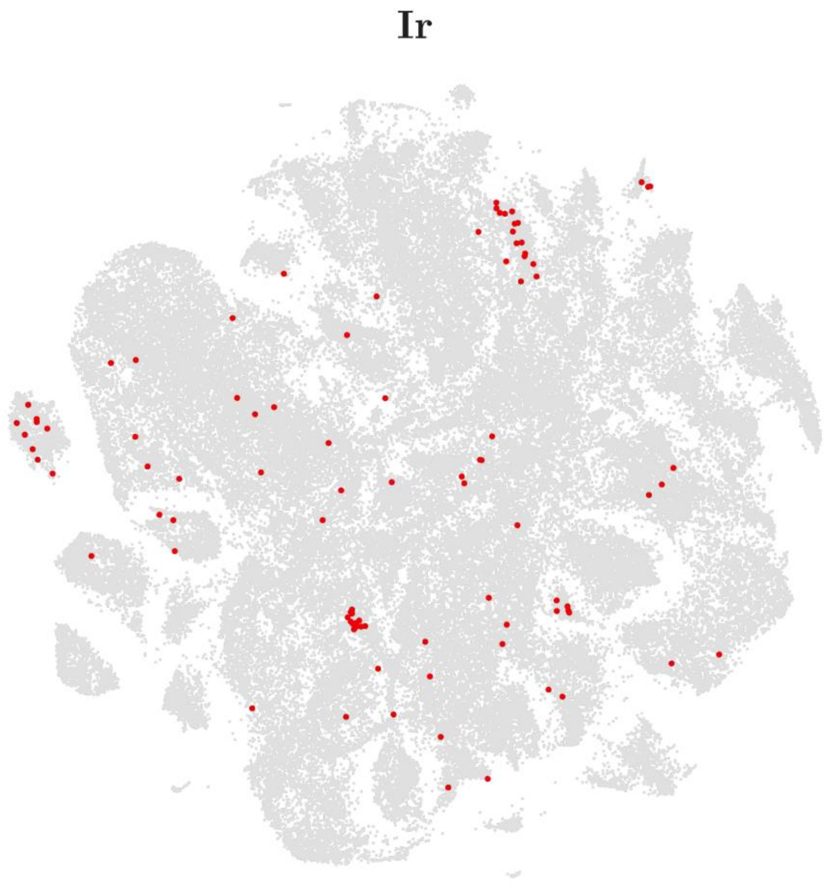

Figure S160. Caption Cluster plot for Platinum.

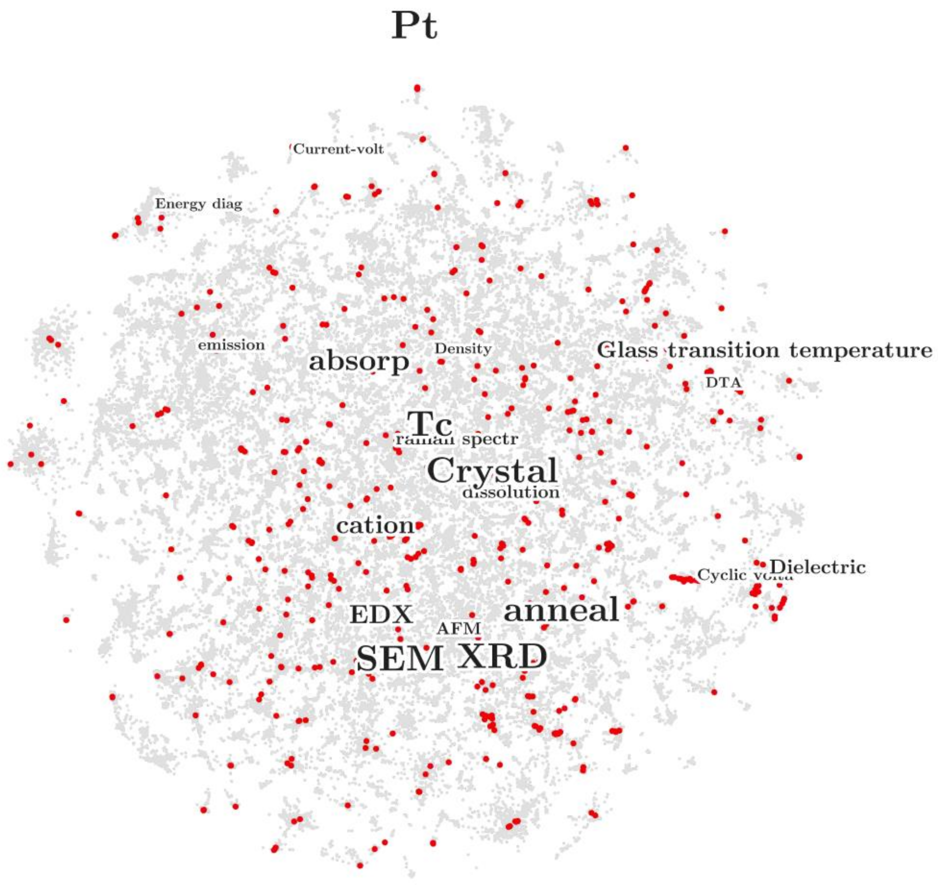

Figure S161. Latent Dirichlet Allocation plot for Platinum.

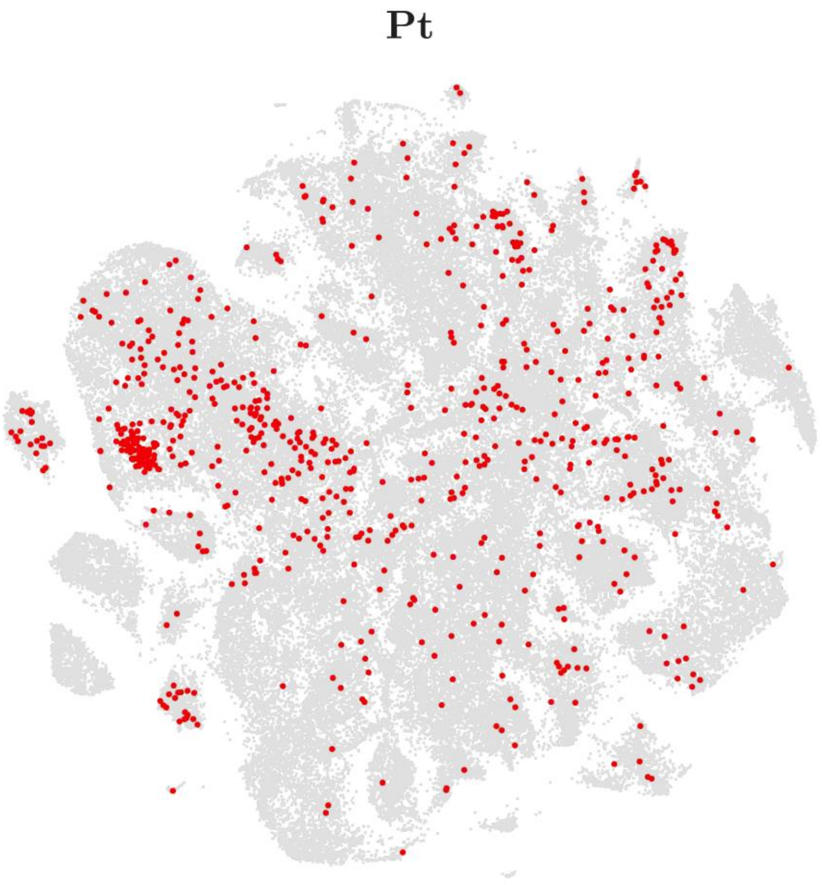

Figure S162. Caption Cluster plot for Gold.

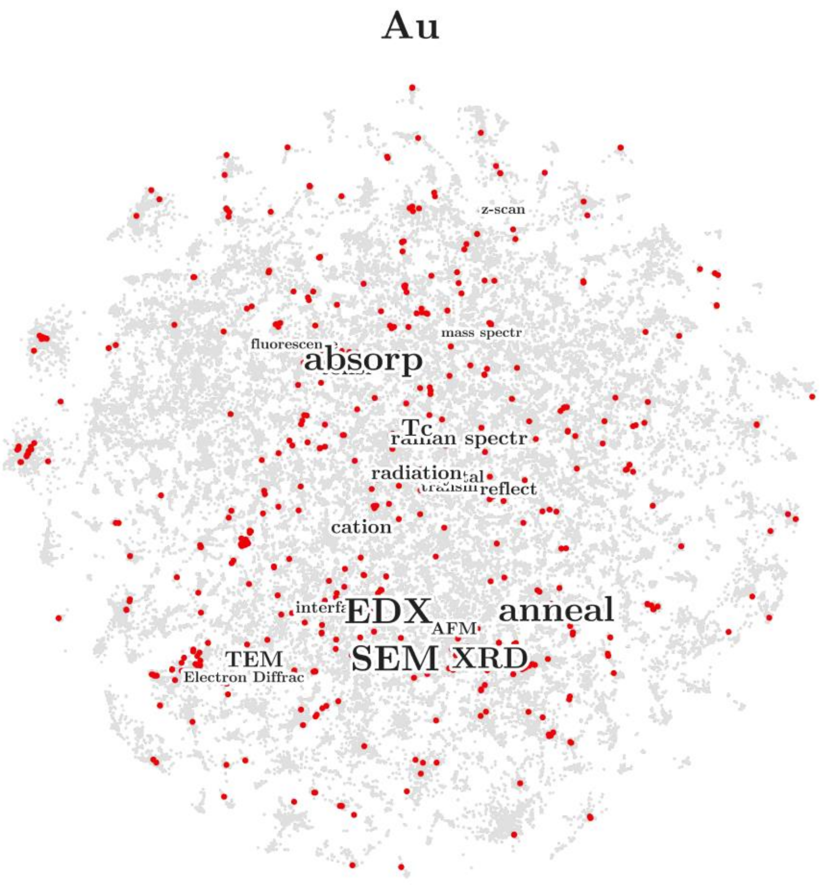

Figure S163. Latent Dirichlet Allocation plot for Gold.

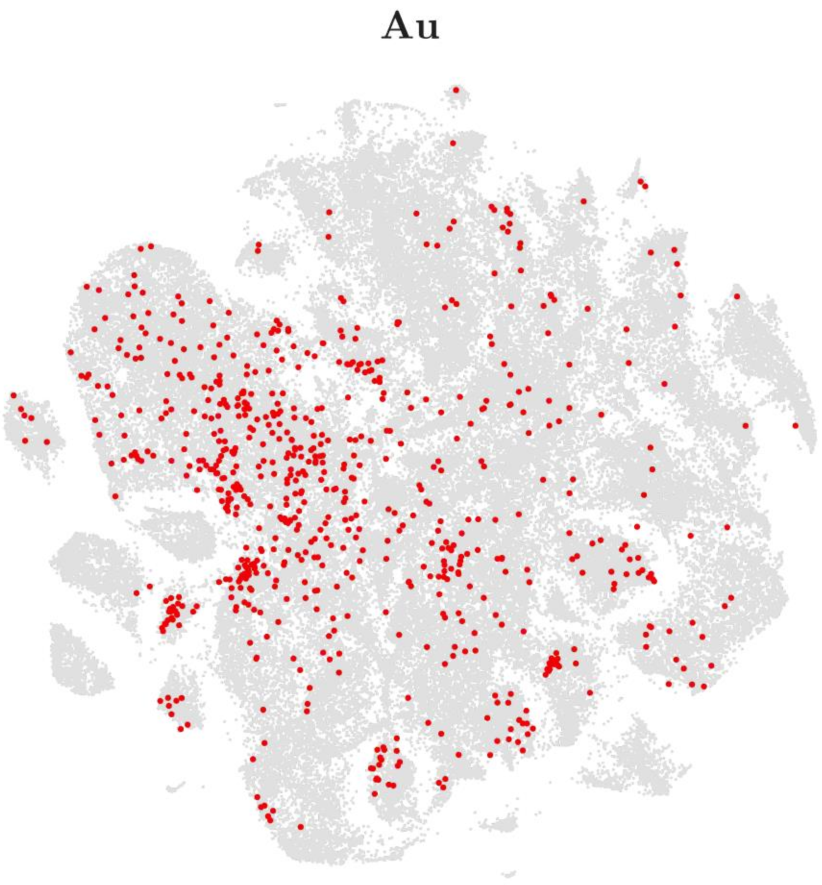

Figure S164. Caption Cluster plot for Mercury.

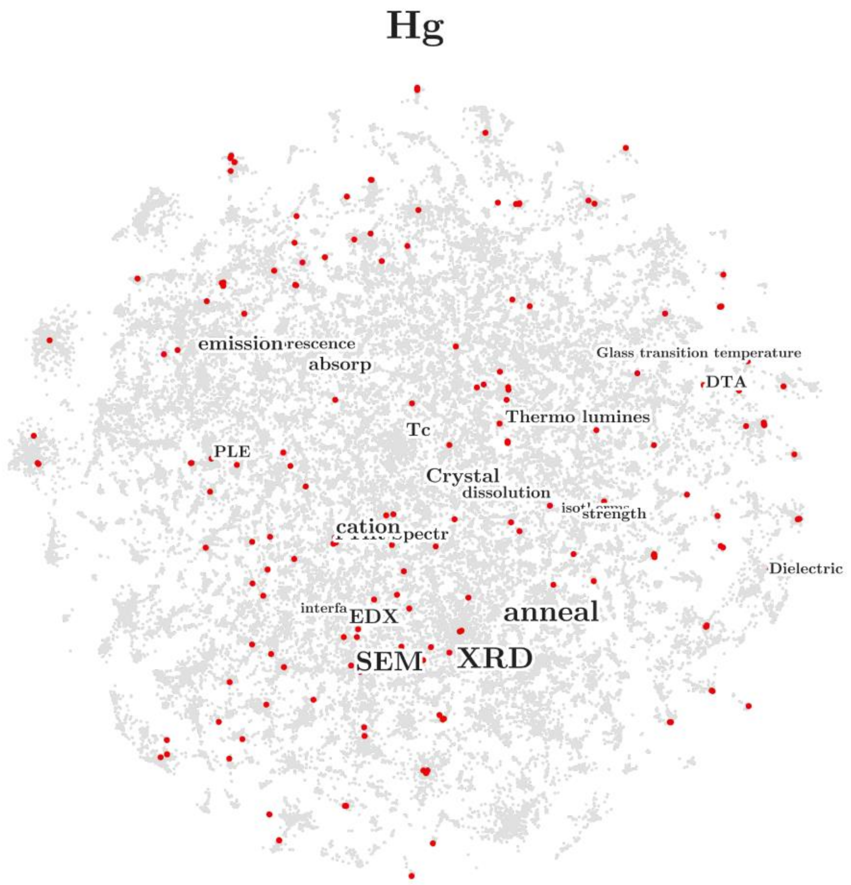

Figure S165. Latent Dirichlet Allocation plot for Mercury.

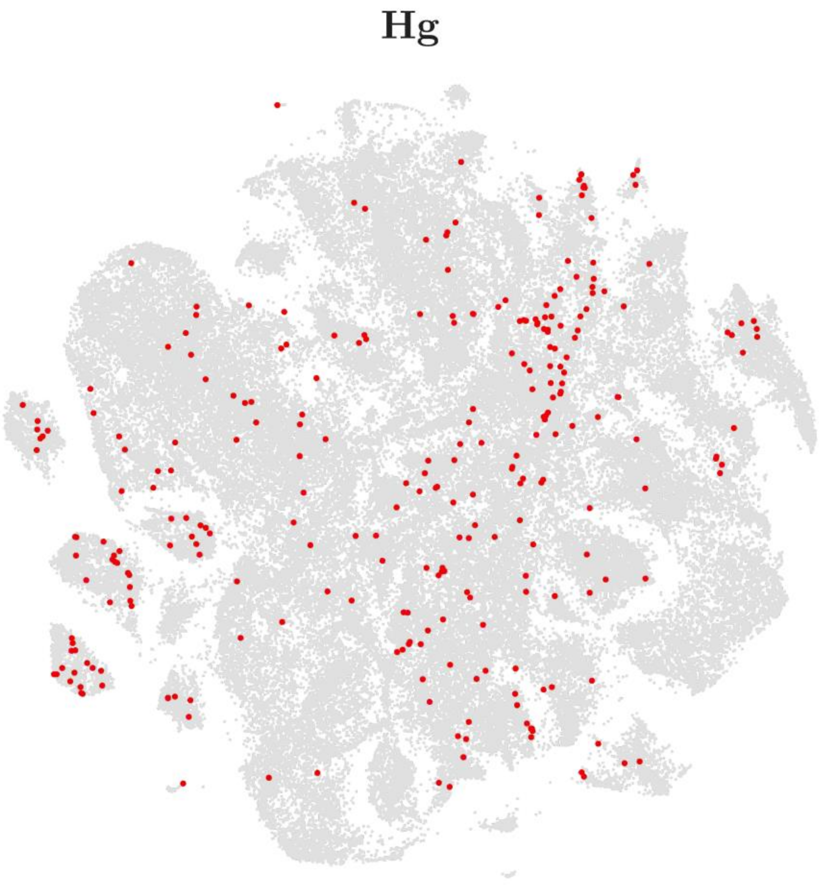

Figure S166. Caption Cluster plot for Thallium.

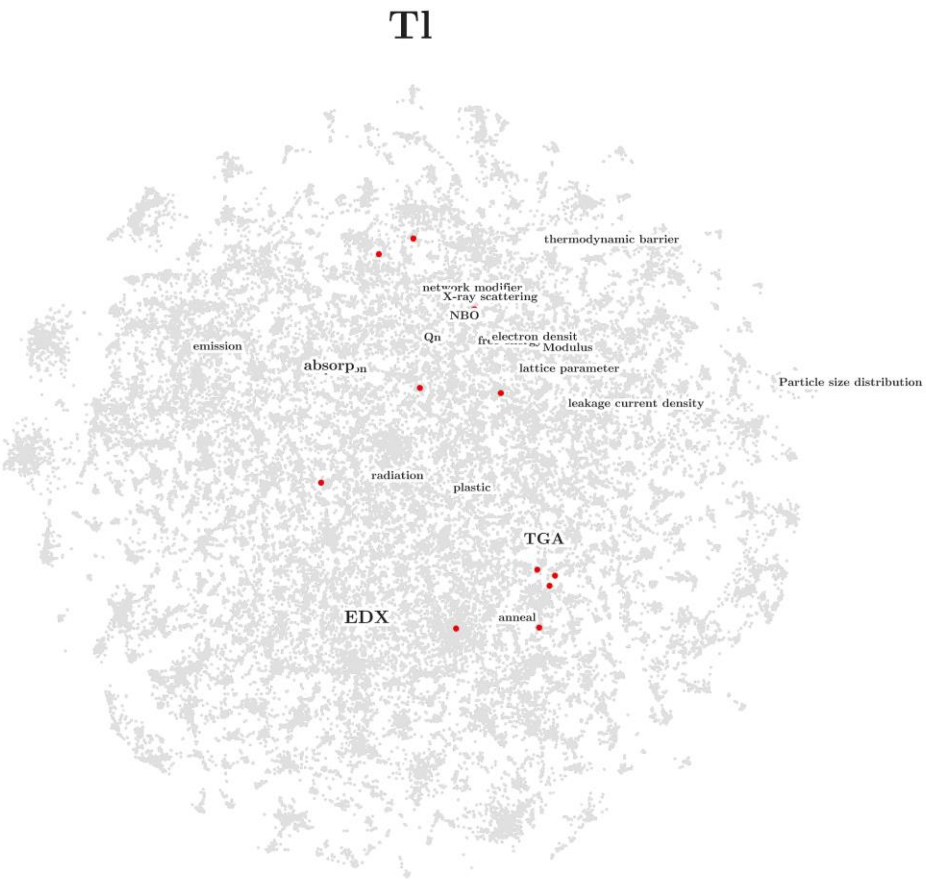

Figure S167. Latent Dirichlet Allocation plot for Thallium.

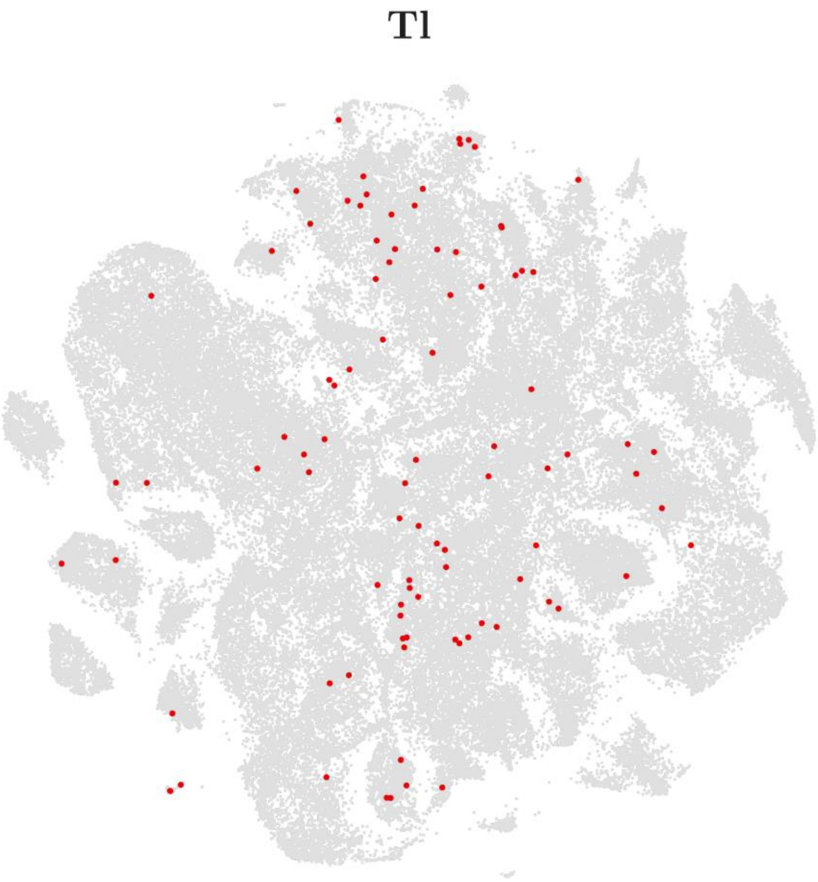

Figure S168. Caption Cluster plot for Lead.

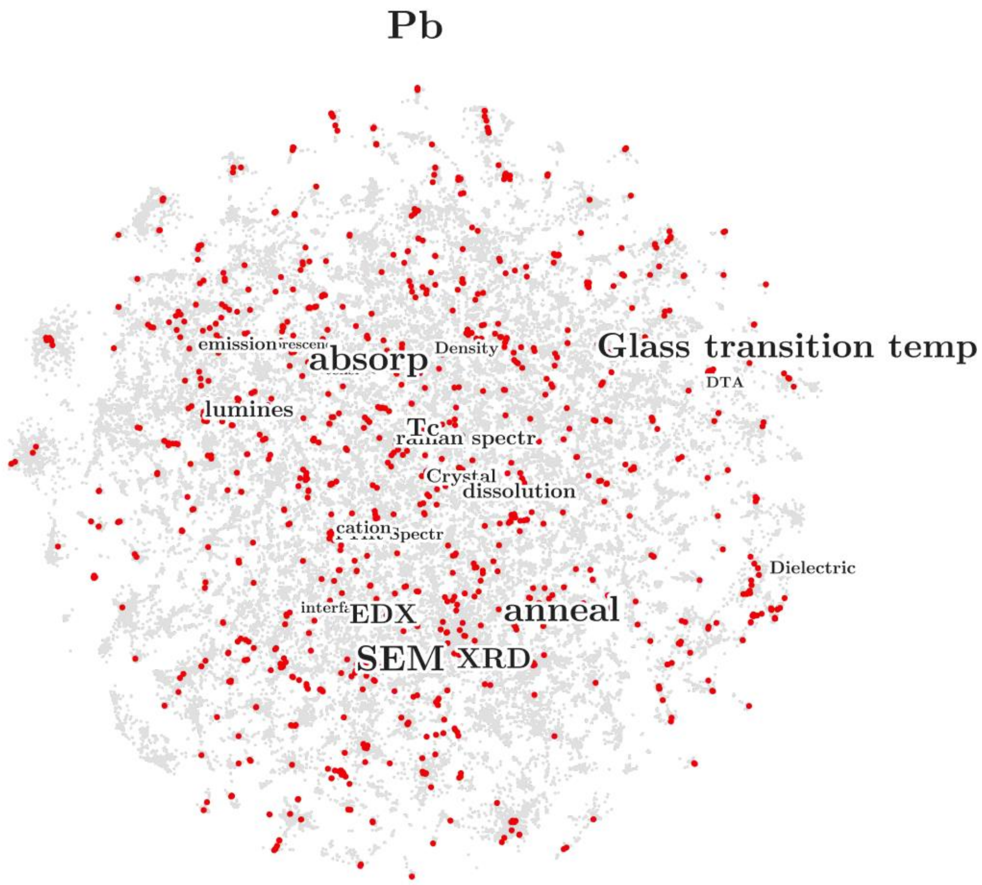

Figure S169. Latent Dirichlet Allocation plot for Lead.

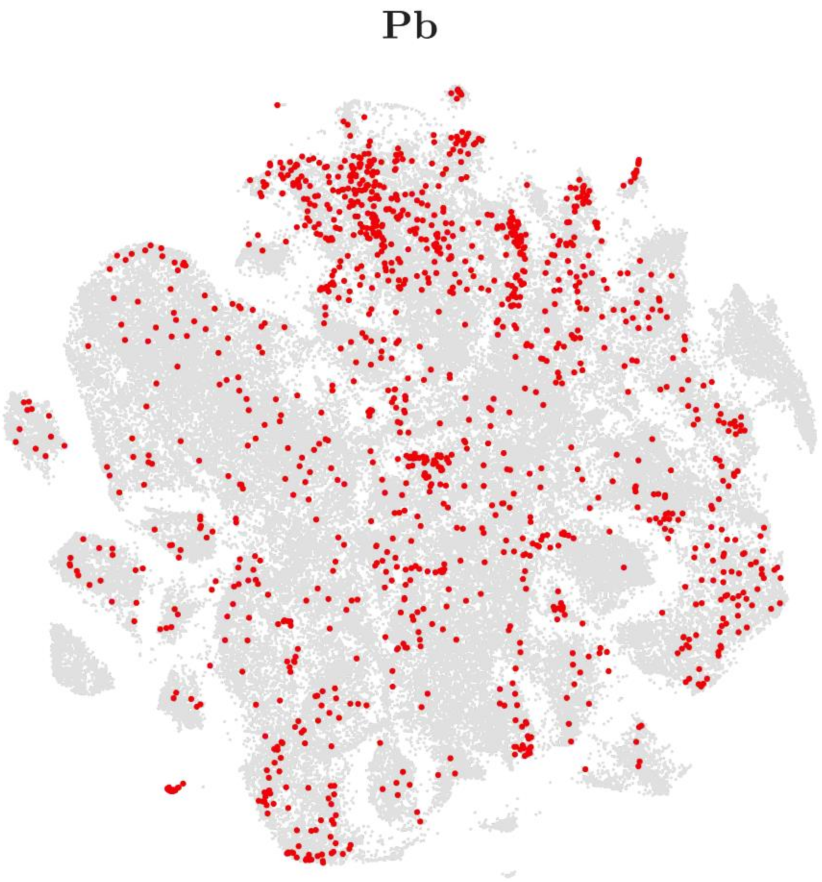

Figure S170. Caption Cluster plot for Bismuth.

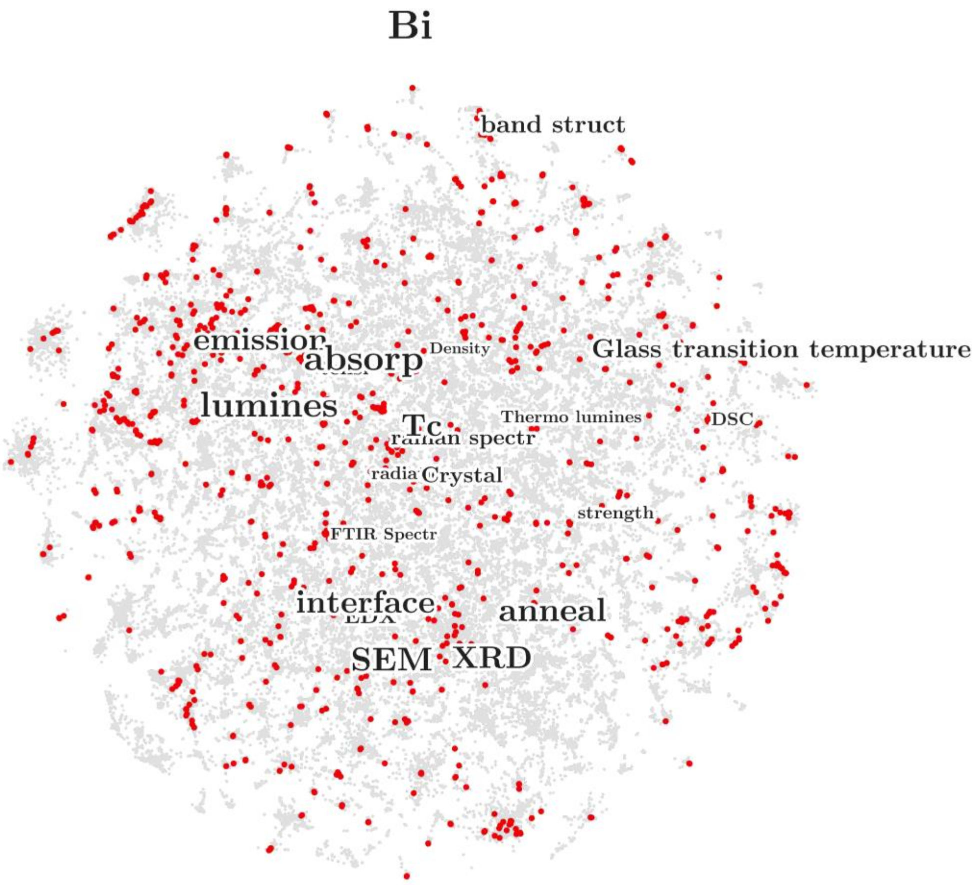

Figure S171. Latent Dirichlet Allocation plot for Bismuth.

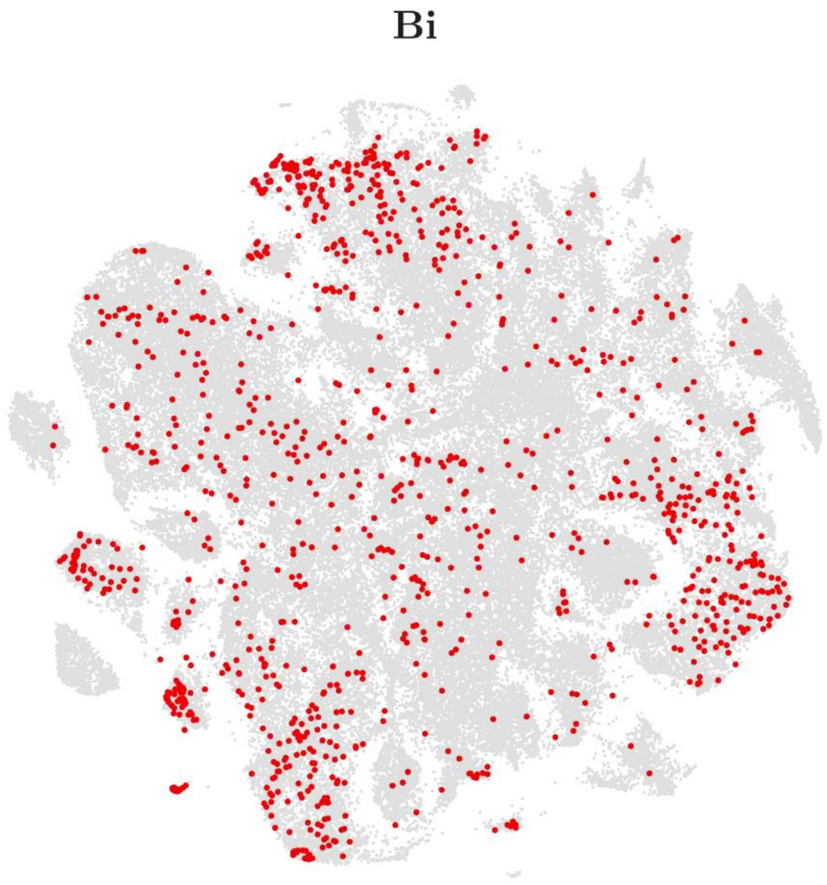

Figure S172. Caption Cluster plot for Polonium.

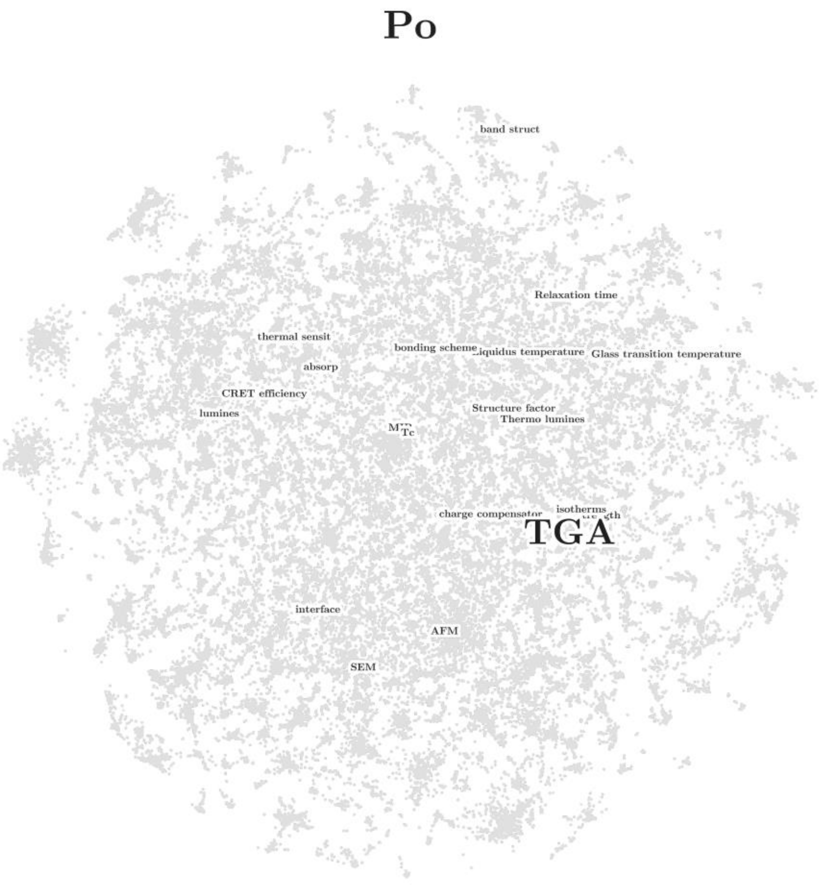

Figure S173. Latent Dirichlet Allocation plot for Polonium.

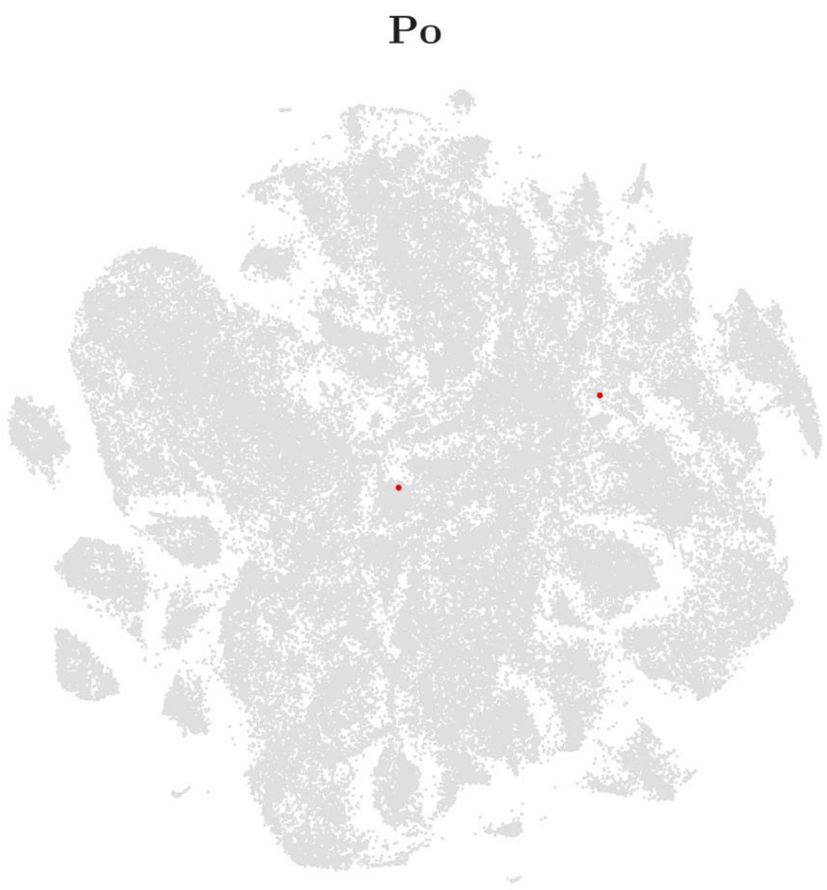

Figure S174. Caption Cluster plot for Astatine.

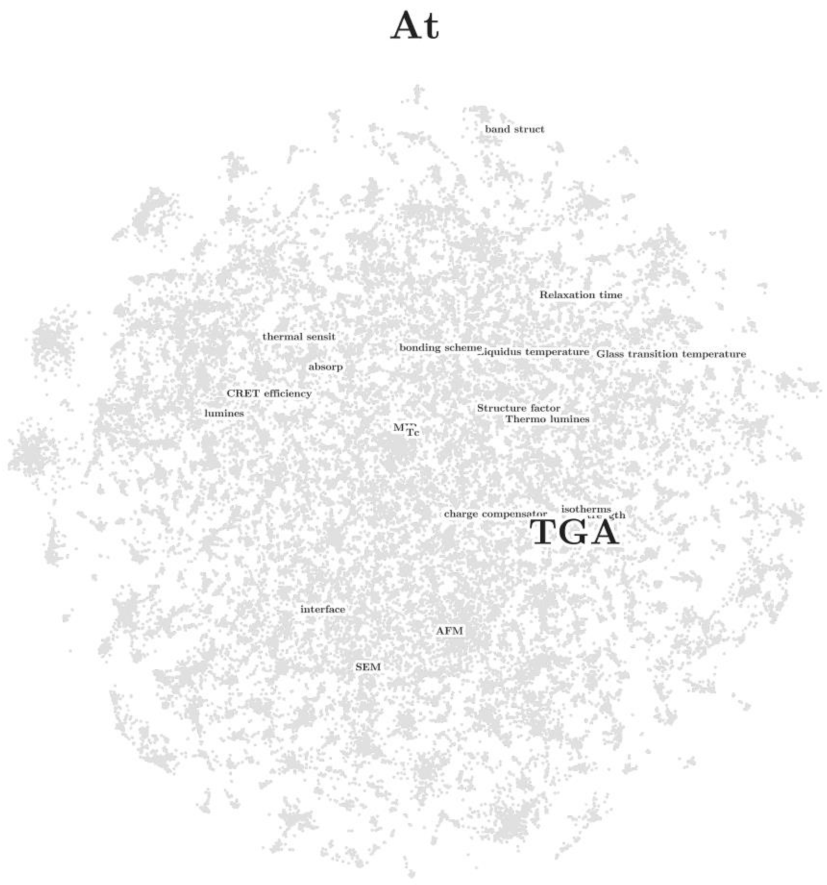

Figure S175. Latent Dirichlet Allocation plot for Astatine.

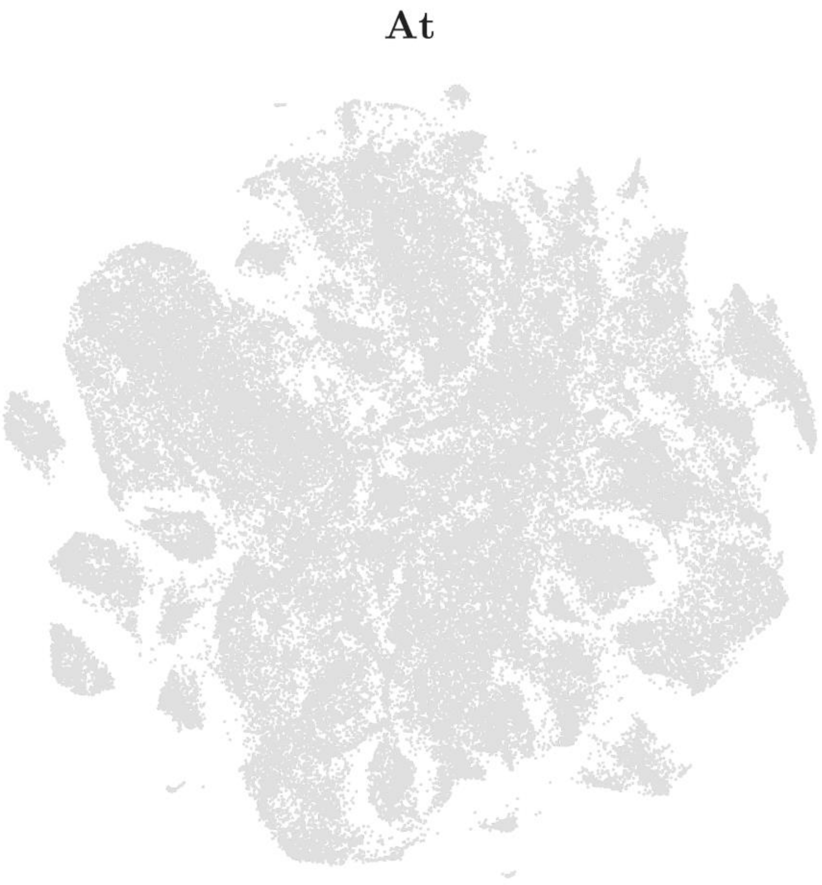

Figure S176. Caption Cluster plot for Radon.

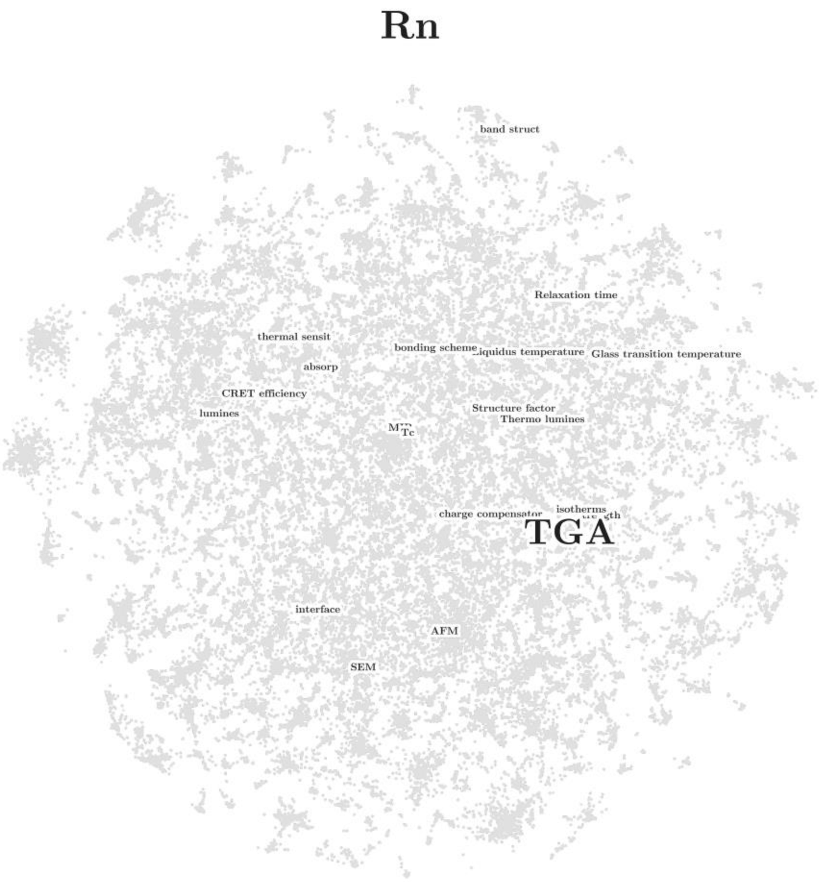

Figure S177. Latent Dirichlet Allocation plot for Radon.

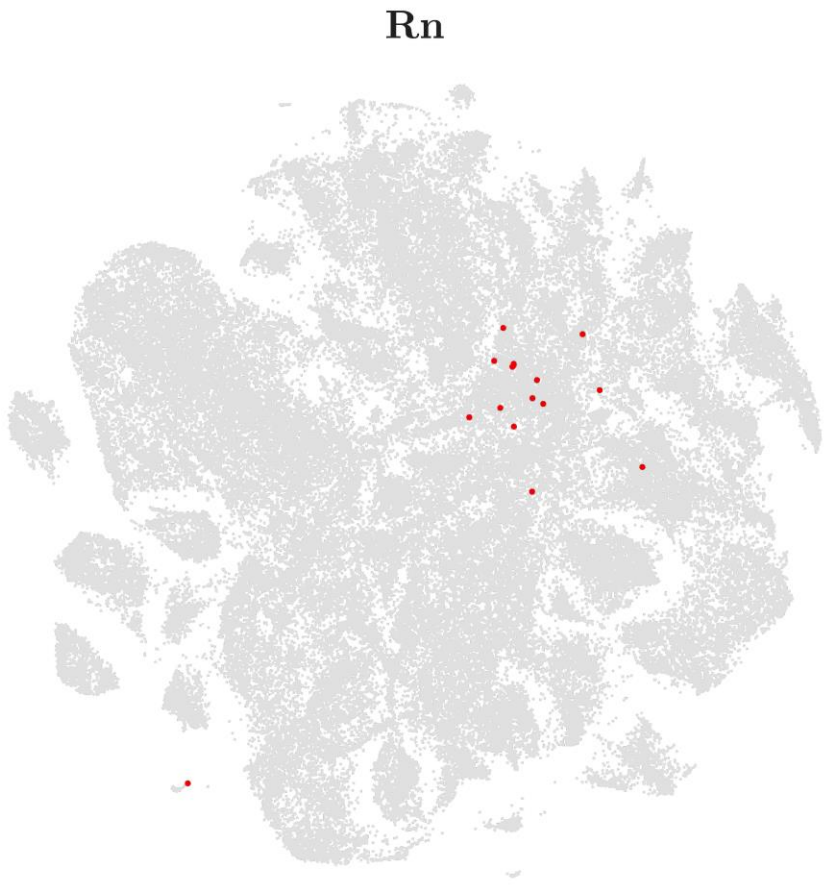

Figure S178. Caption Cluster plot for Francium.

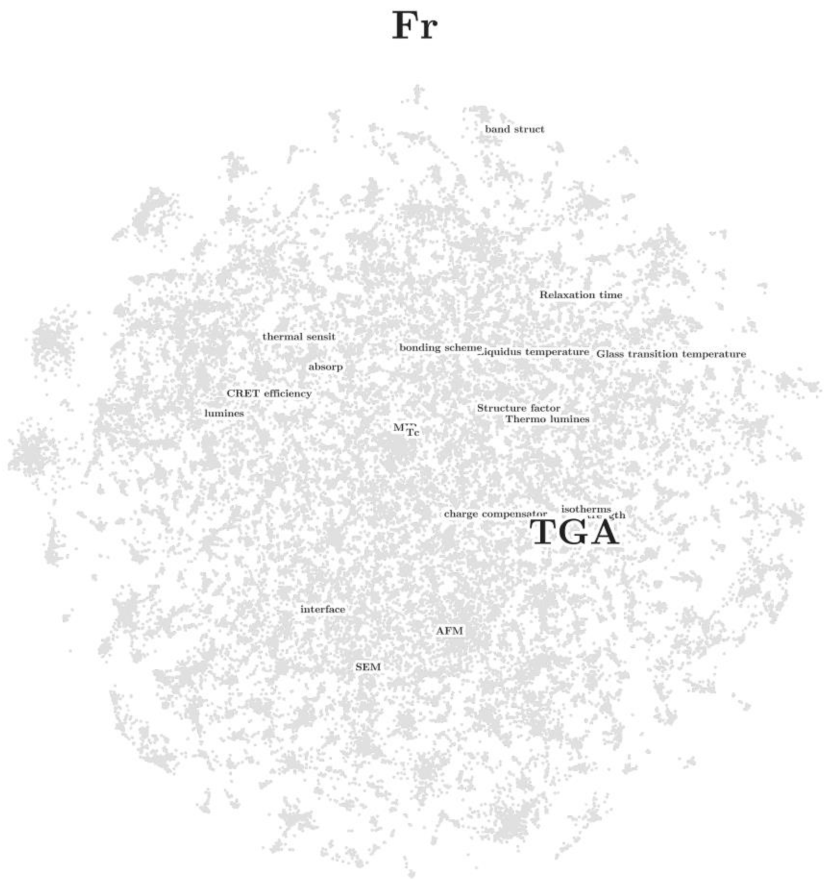

Figure S179. Latent Dirichlet Allocation plot for Francium.

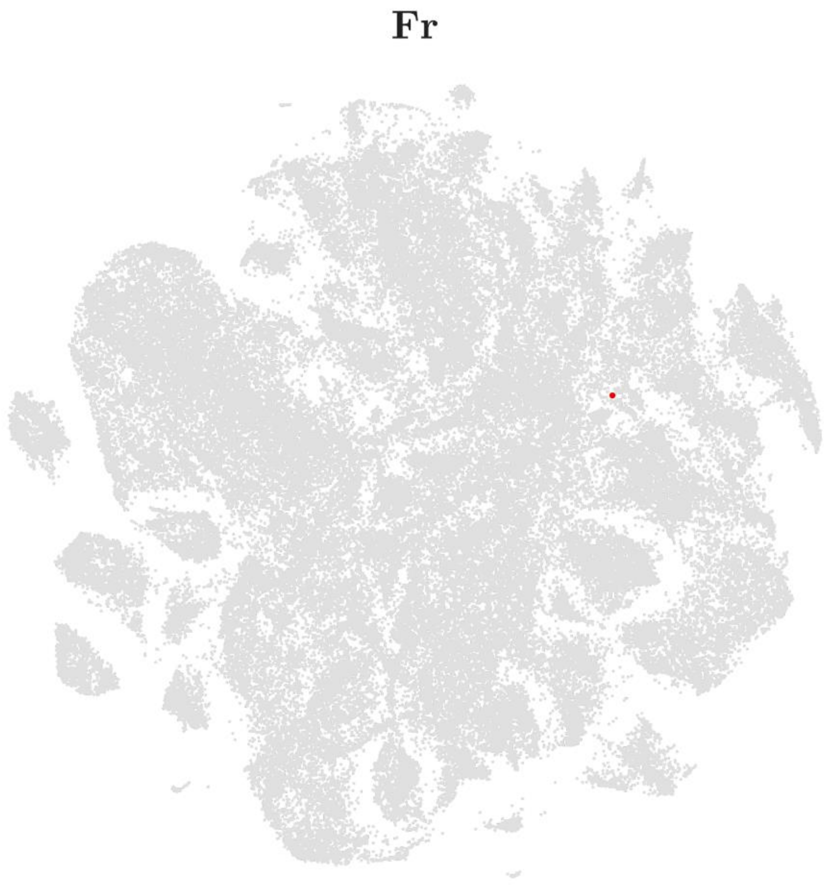

Figure S180. Caption Cluster plot for Radium.

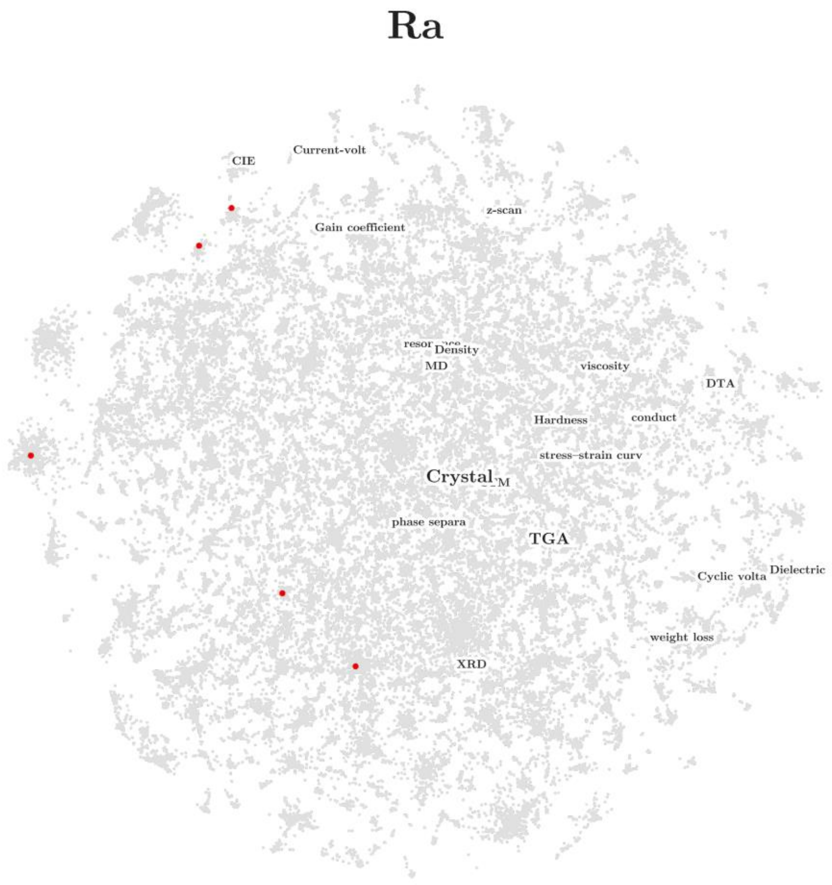

Figure S181. Latent Dirichlet Allocation plot for Radium.

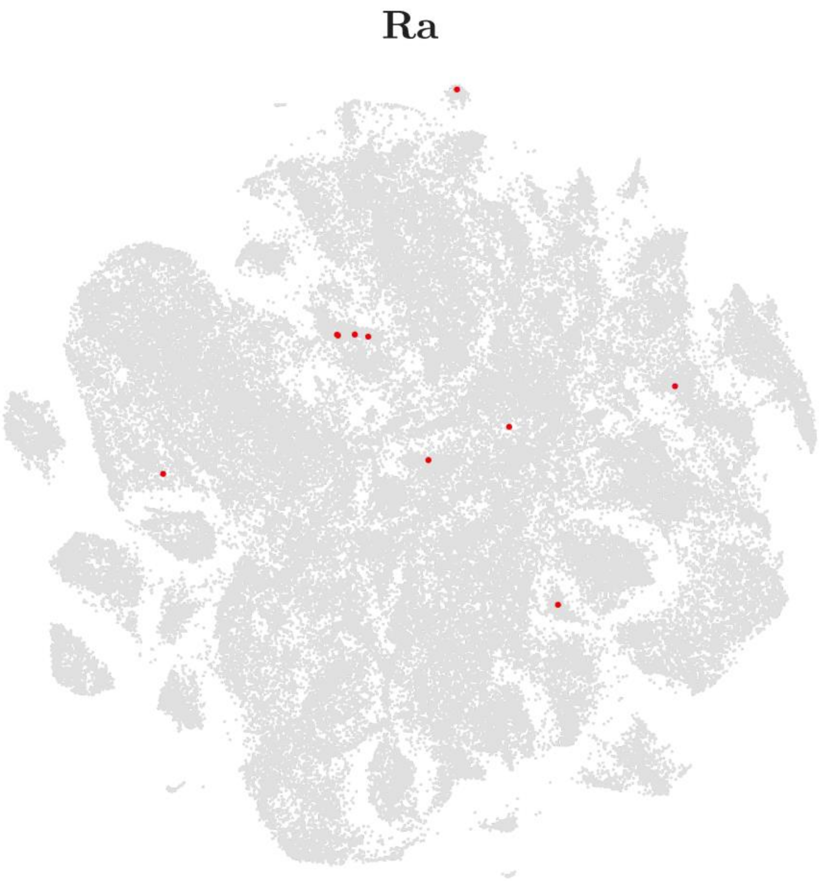

Figure S182. Caption Cluster plot for Actinium.

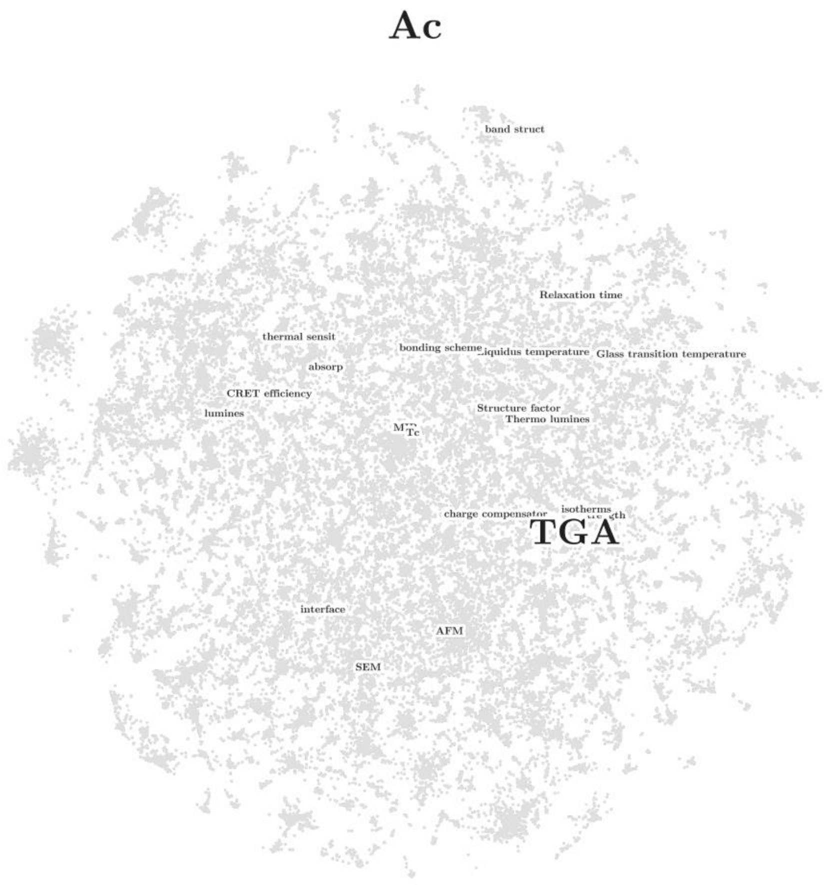

Figure S183. Latent Dirichlet Allocation plot for Actinium.

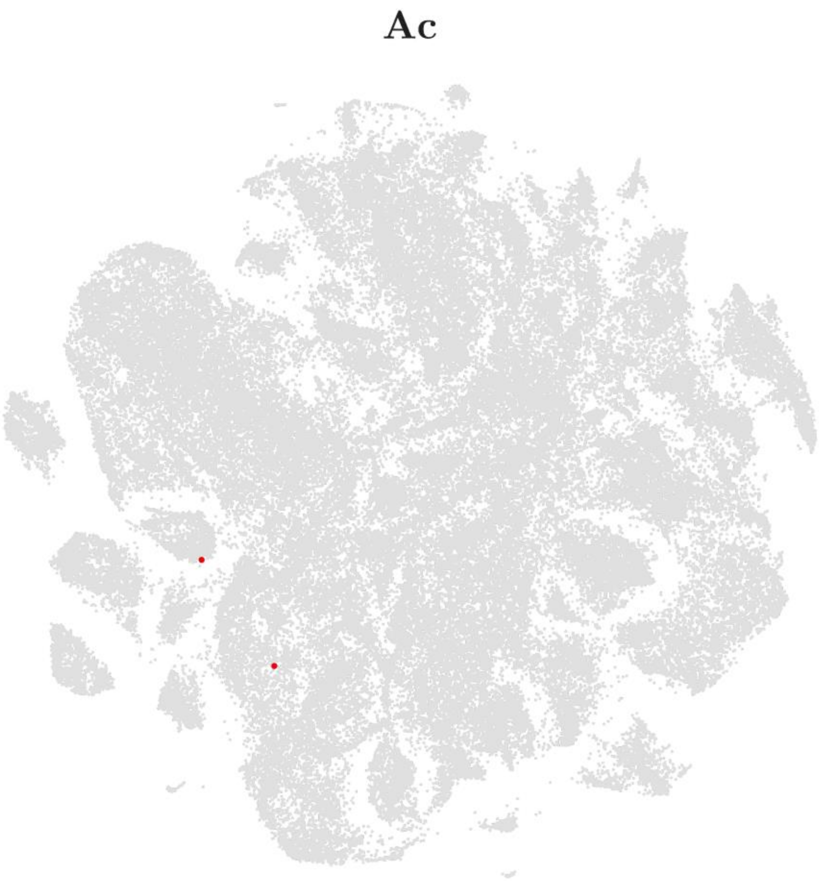

Figure S184. Caption Cluster plot for Thorium.

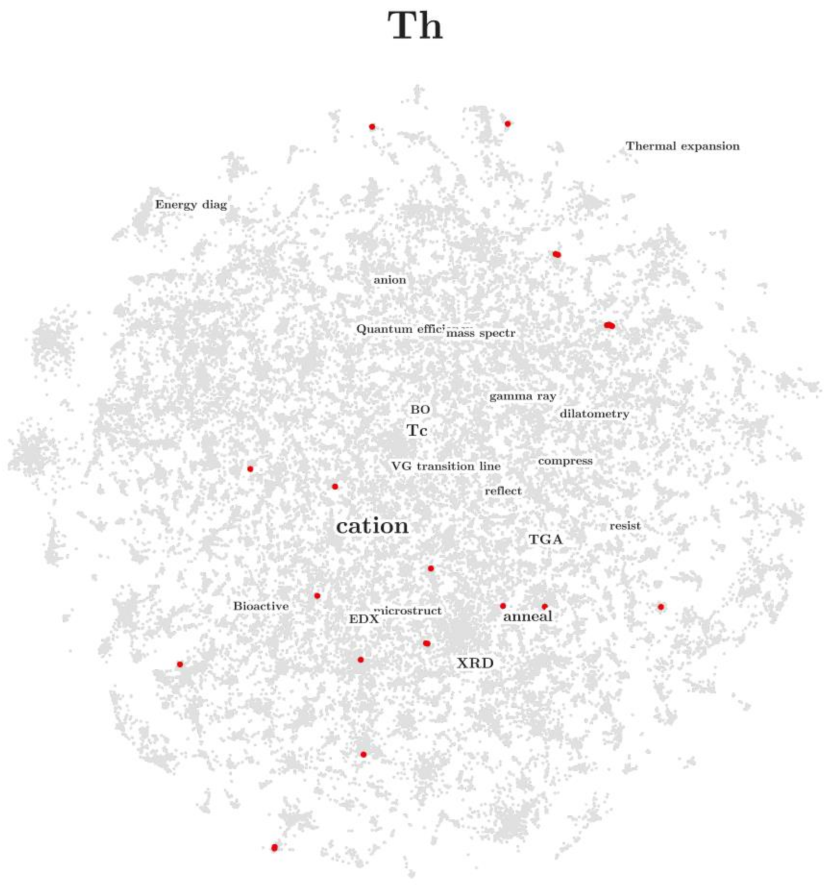

Figure S185. Latent Dirichlet Allocation plot for Thorium.

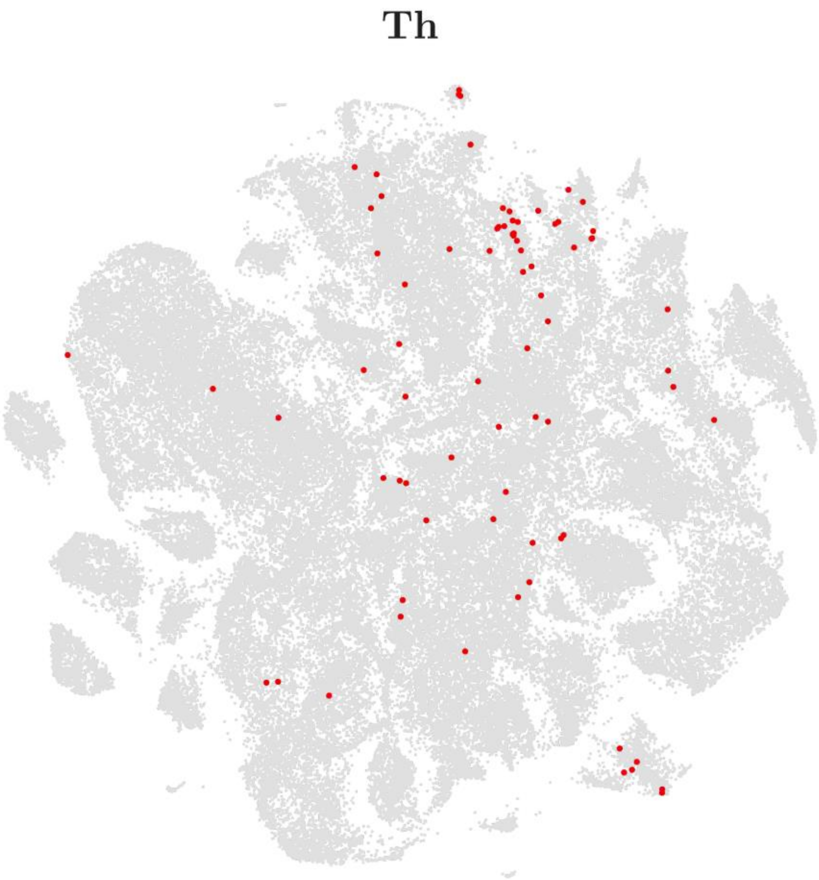

Figure S186. Caption Cluster plot for Protactinium.

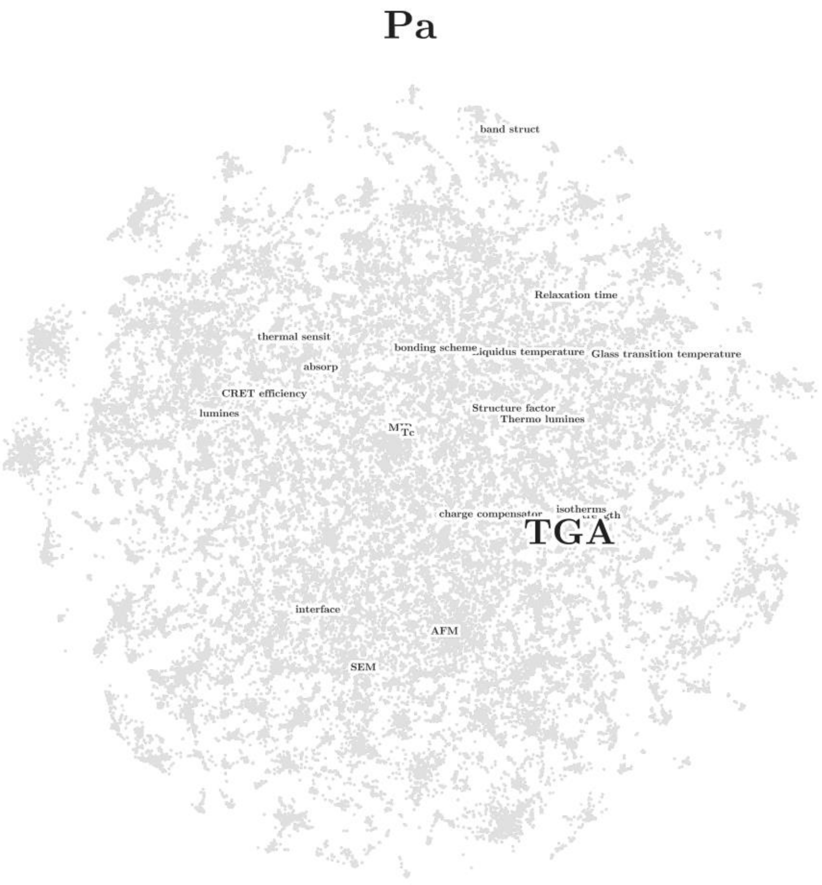

Figure S187. Latent Dirichlet Allocation plot for Protactinium.

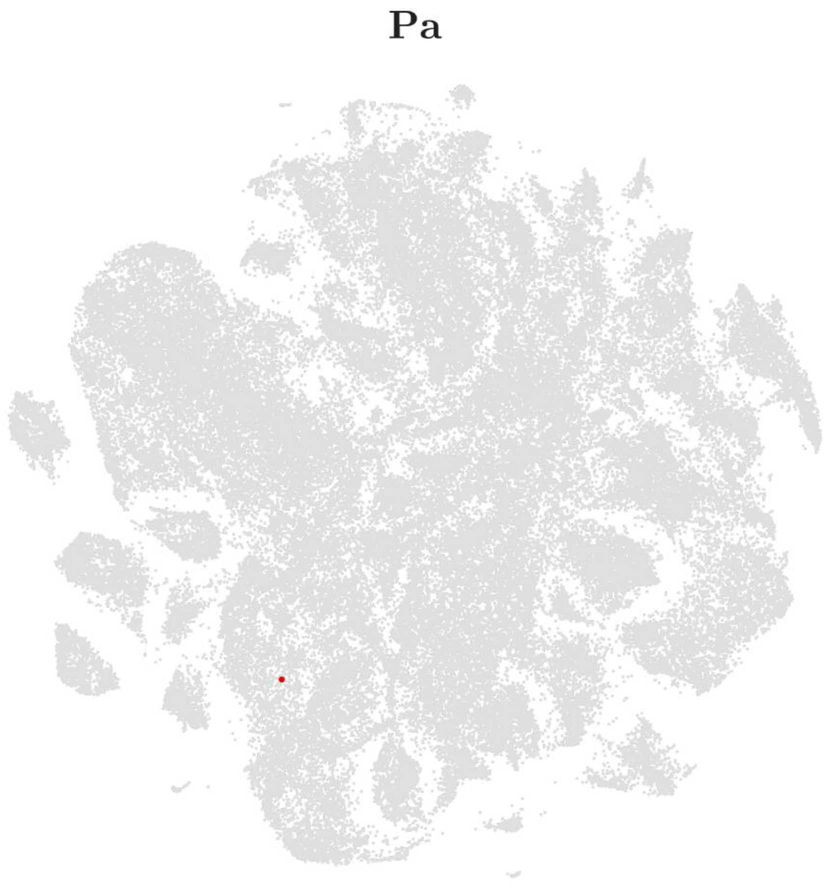

Figure S188. Caption Cluster plot for Uranium.

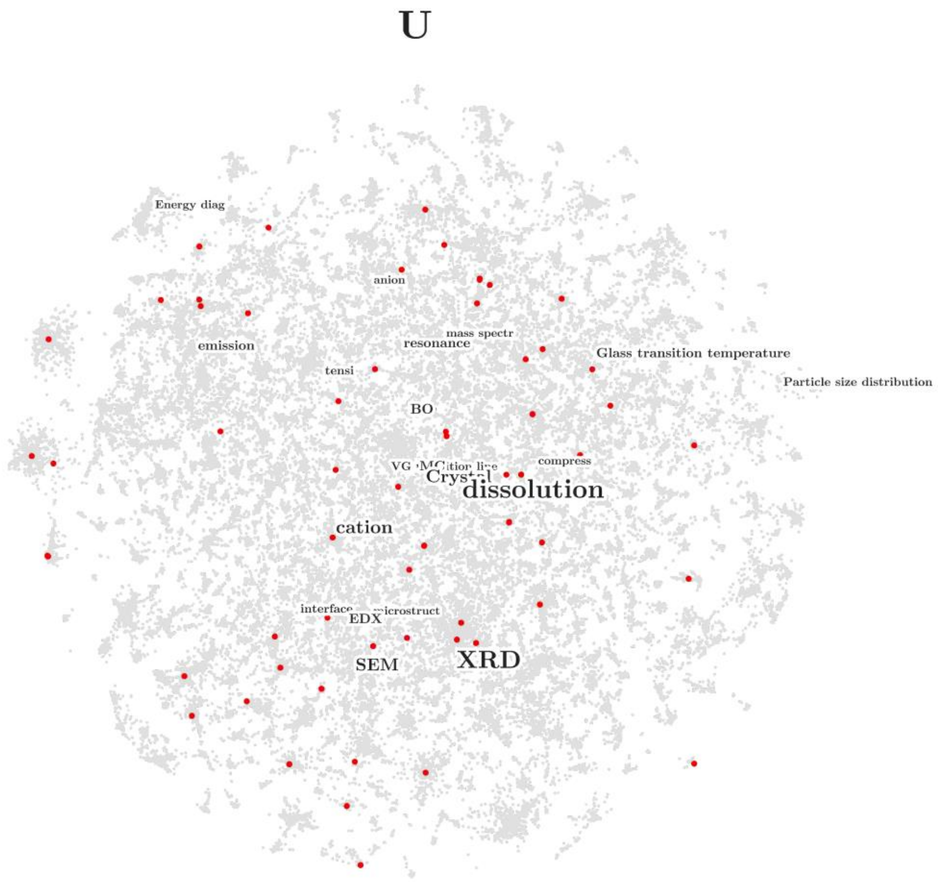

Figure S189. Latent Dirichlet Allocation plot for Uranium.

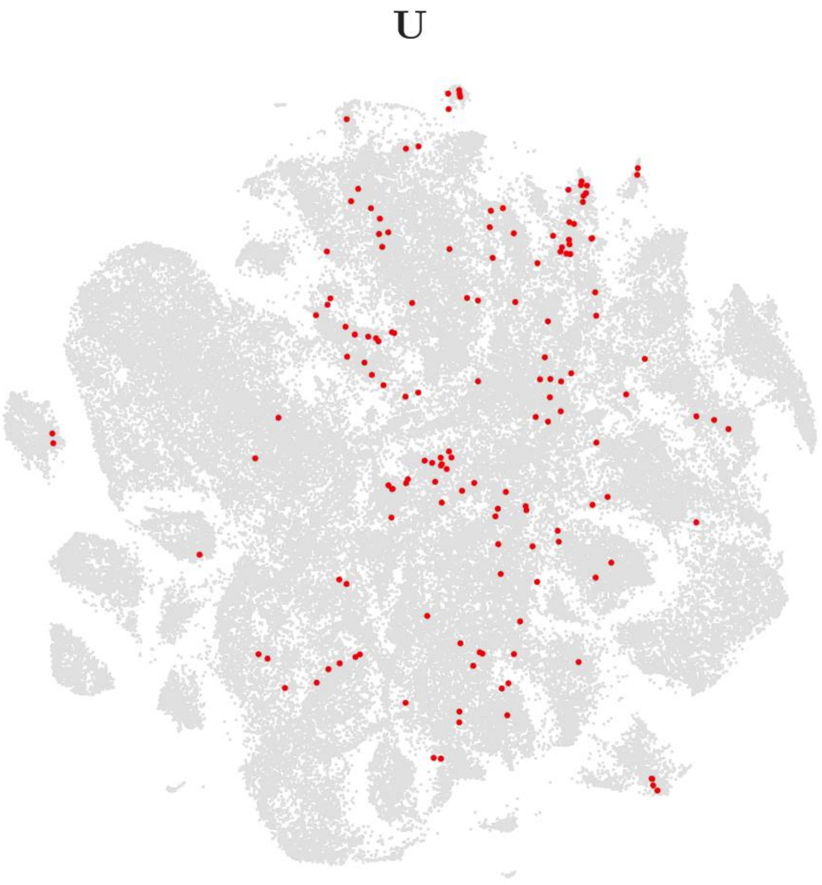

Figure S190. Caption Cluster plot for Neptunium.

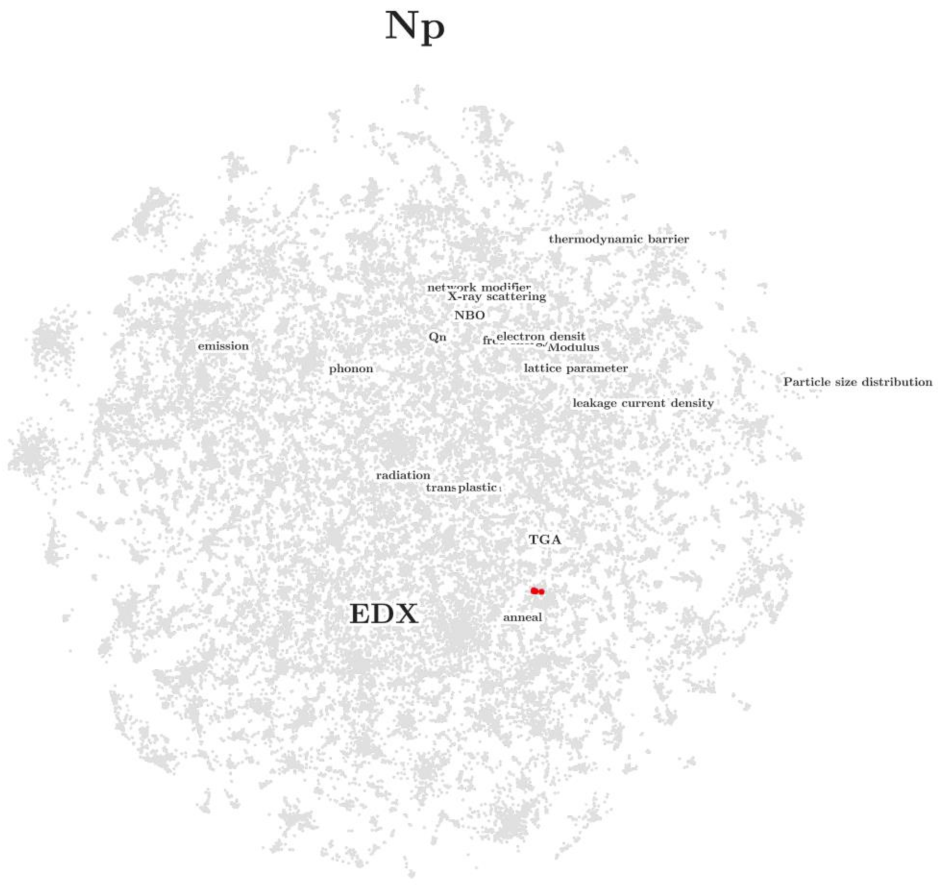

Figure S191. Latent Dirichlet Allocation plot for Neptunium.

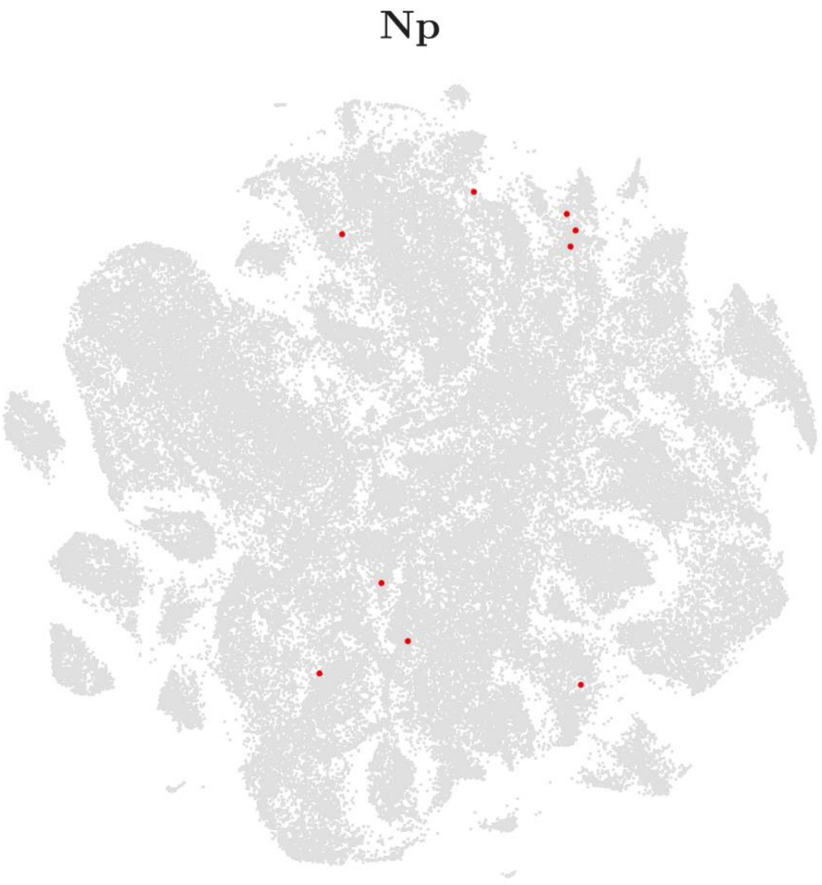

Figure S192. Caption Cluster plot for Plutonium.

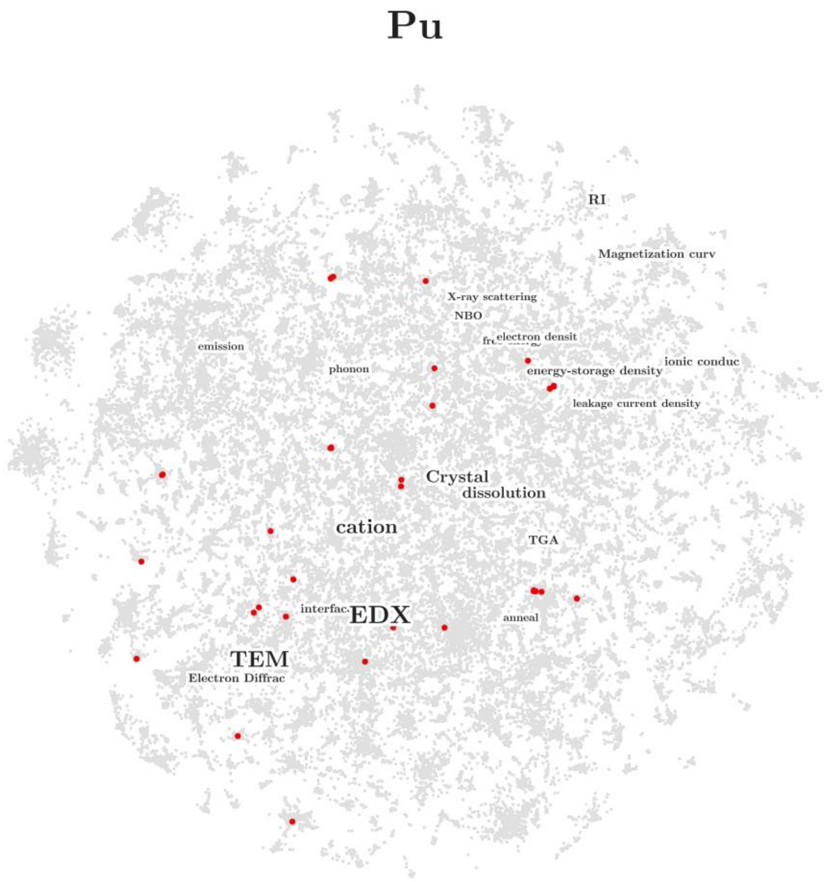

Figure S193. Latent Dirichlet Allocation plot for Plutonium.

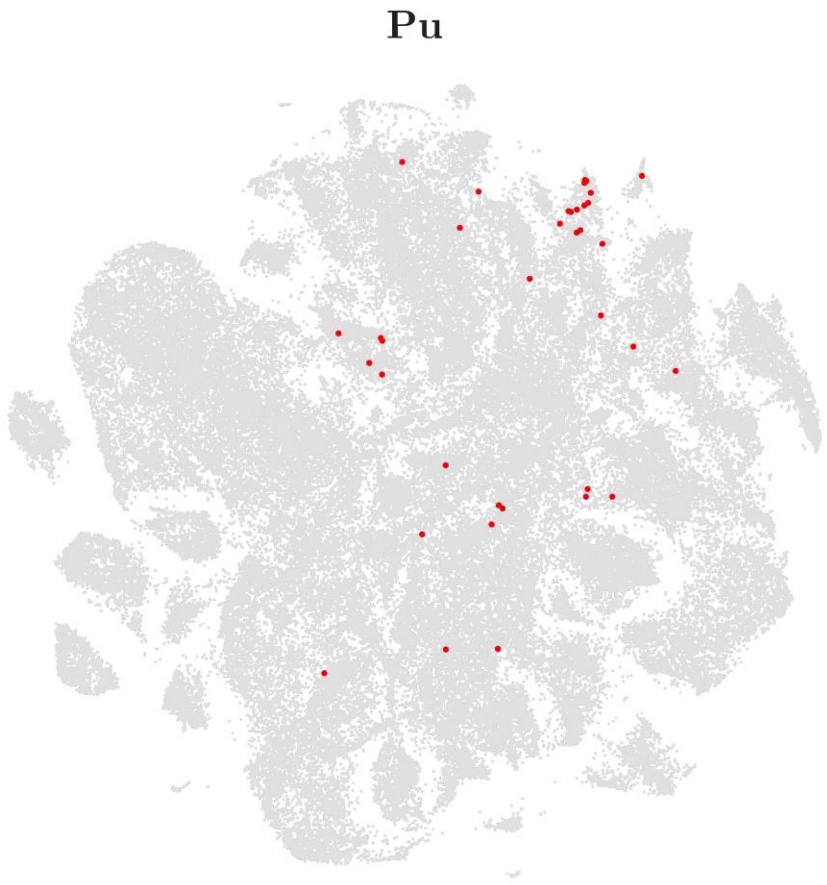

Figure S194. Caption Cluster plot for Americium.

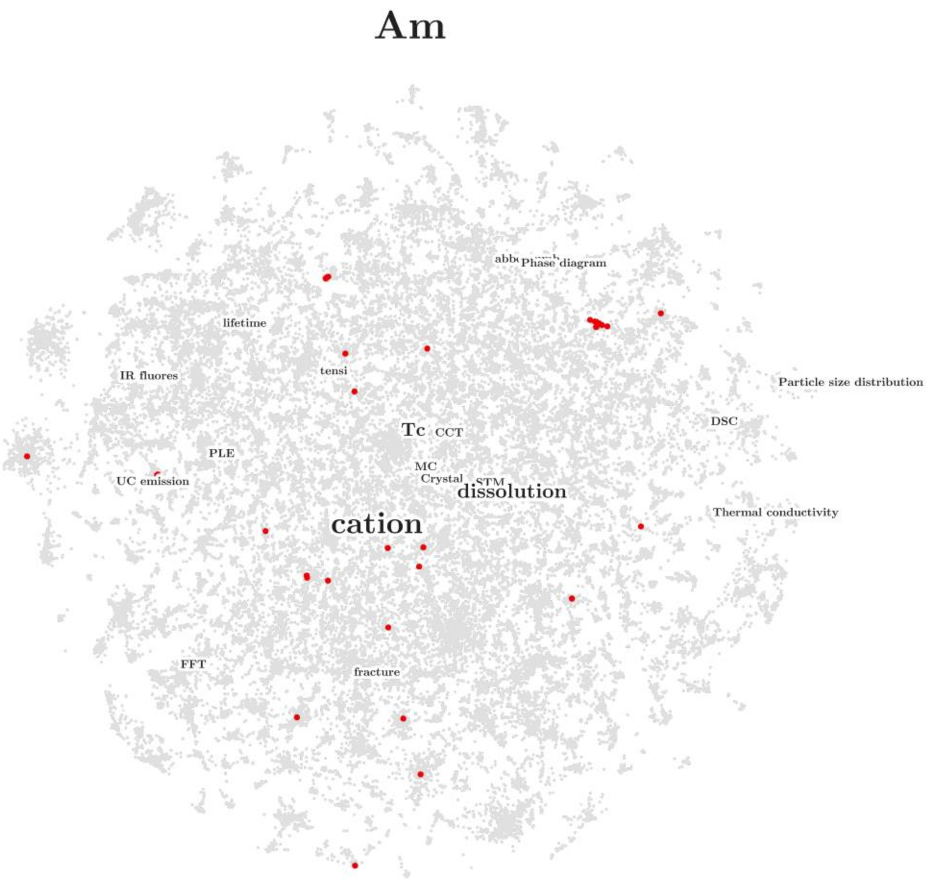

Figure S195. Latent Dirichlet Allocation plot for Americium.

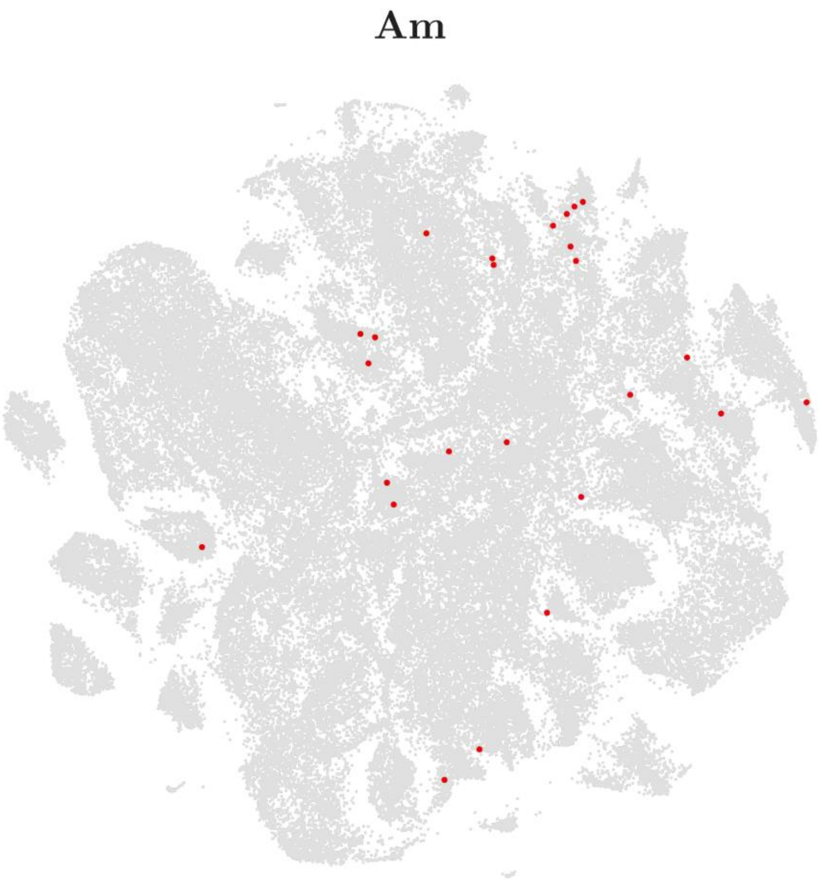

Figure S196. Caption Cluster plot for Curium.

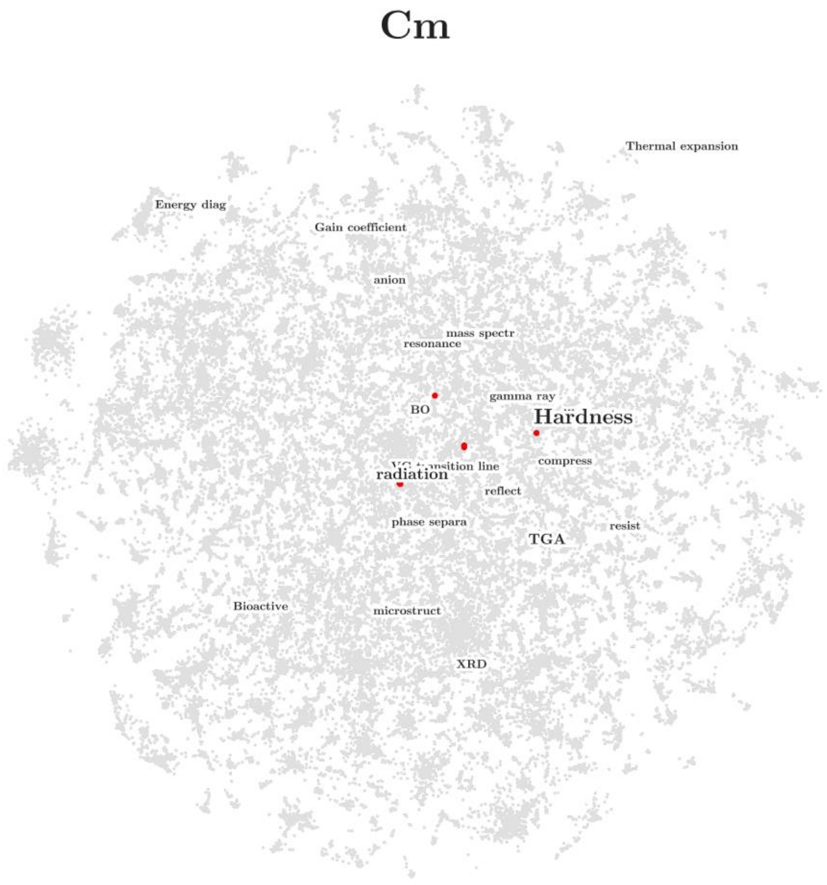

Figure S197. Latent Dirichlet Allocation plot for Curium.

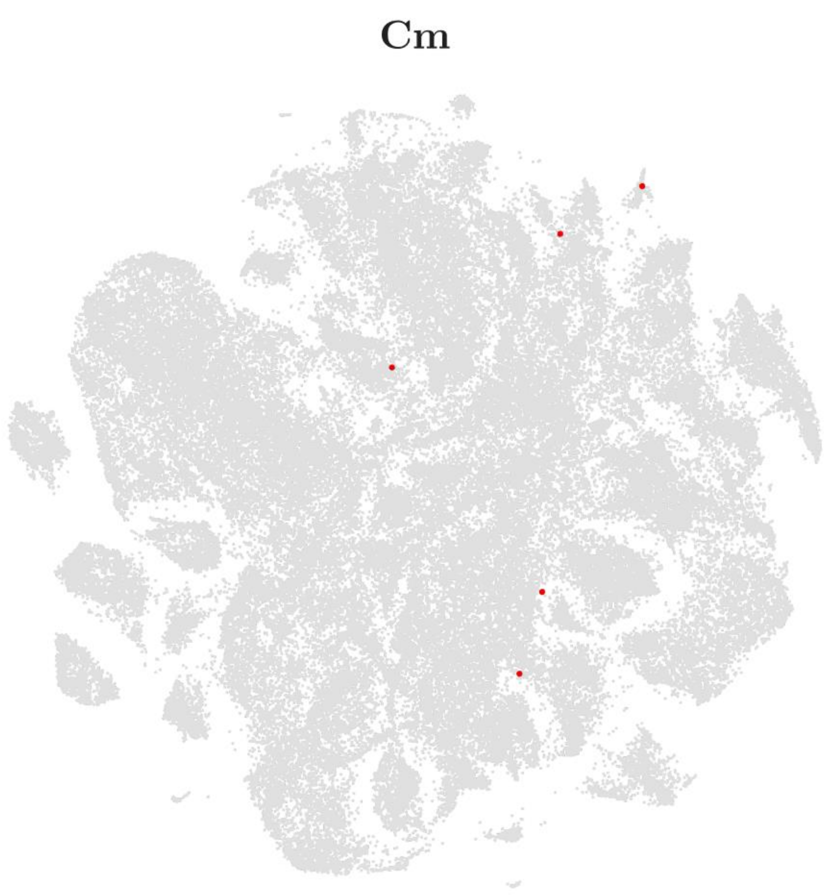

Figure S198. Caption Cluster plot for Berkelium.

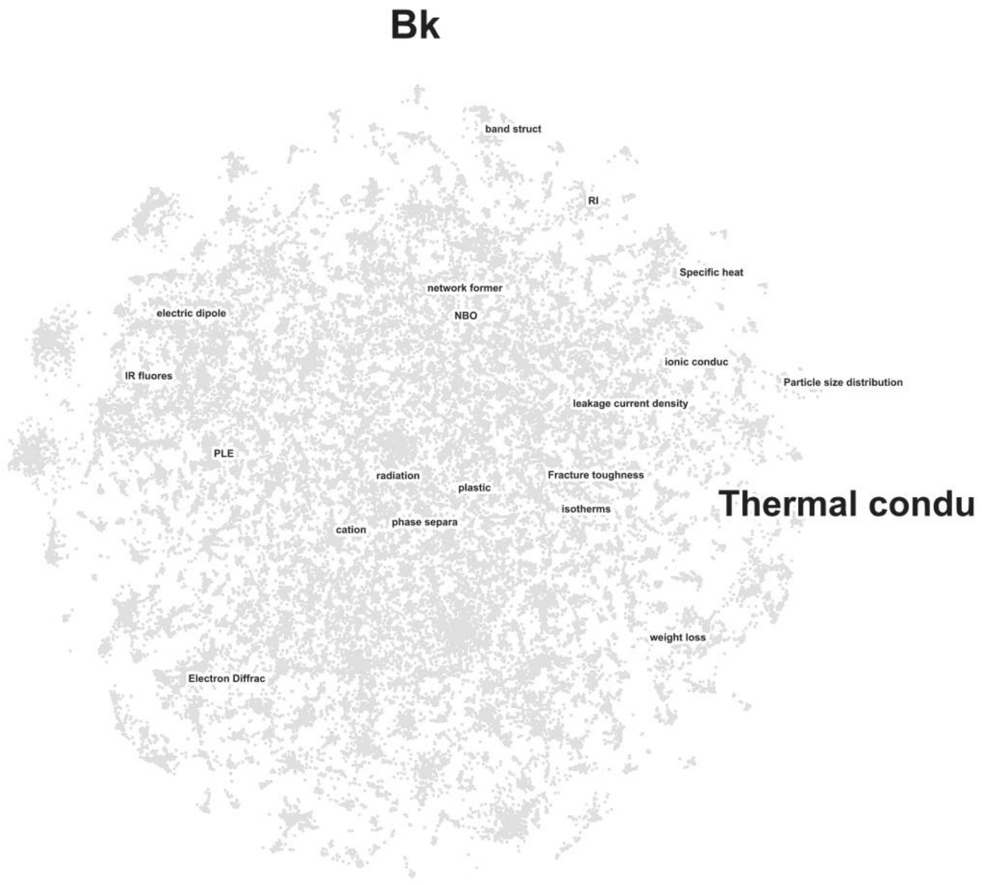

Figure S199. Latent Dirichlet Allocation plot for Berkelium.

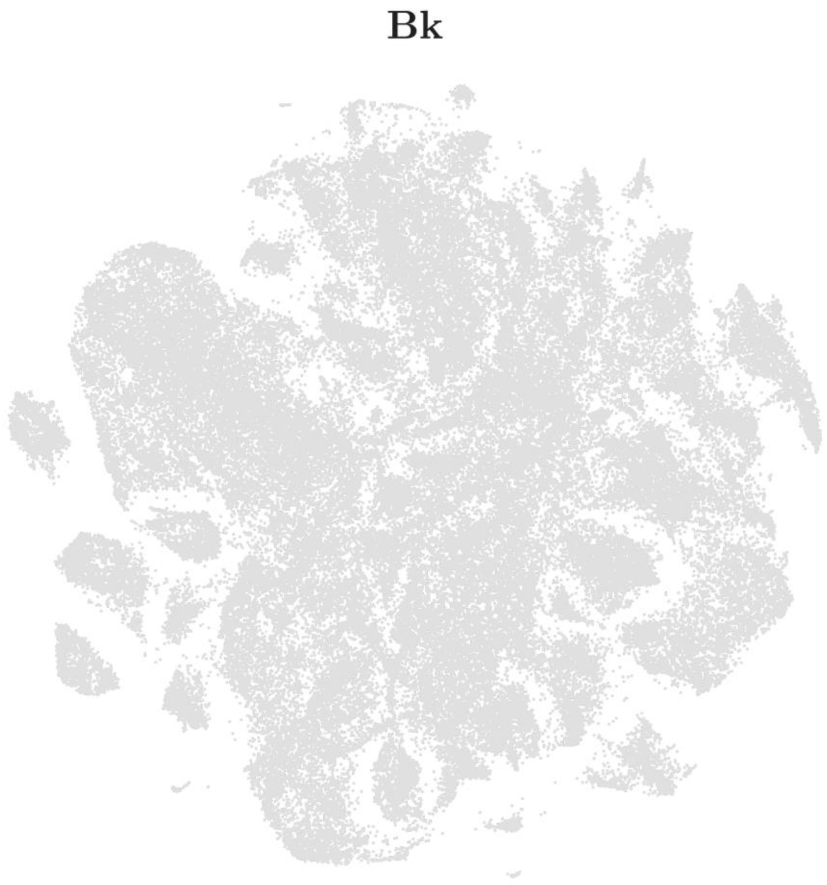

Figure S200. Caption Cluster plot for Californium.

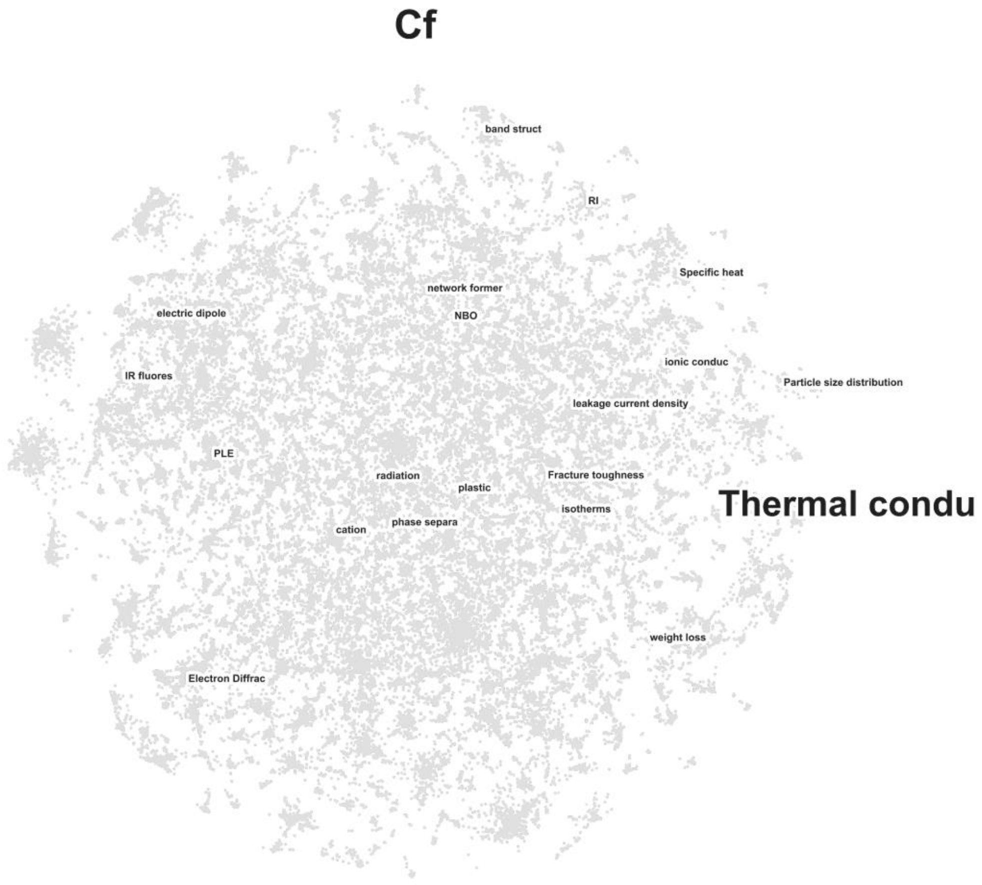

Figure S201. Latent Dirichlet Allocation plot for Californium.

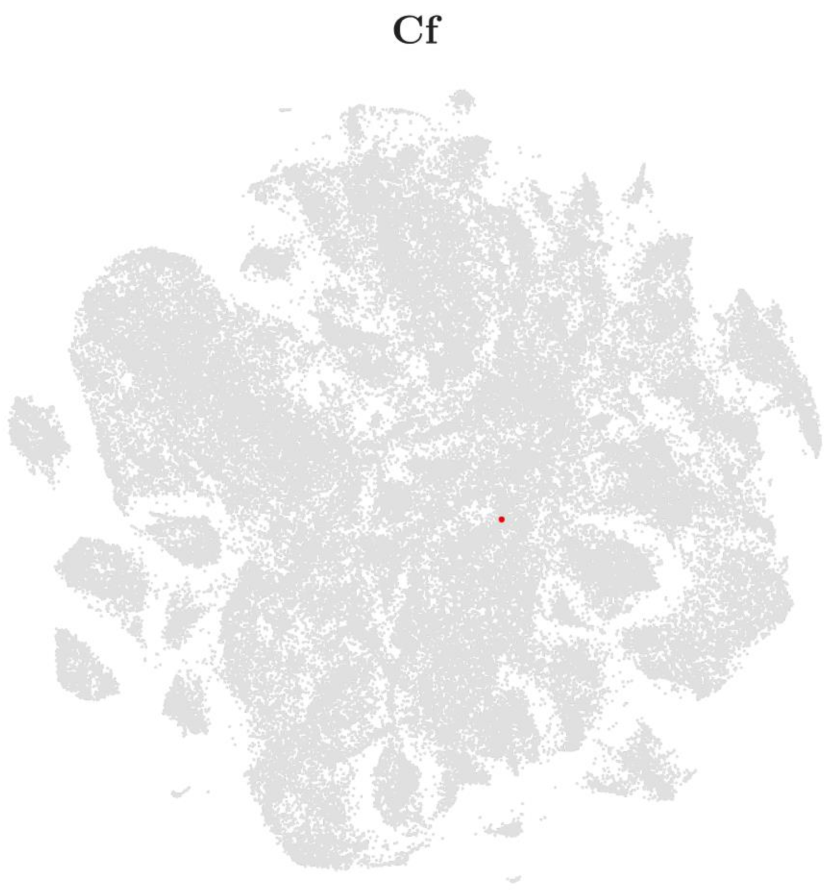

Figure S202. Caption Cluster plot for Einsteinium.

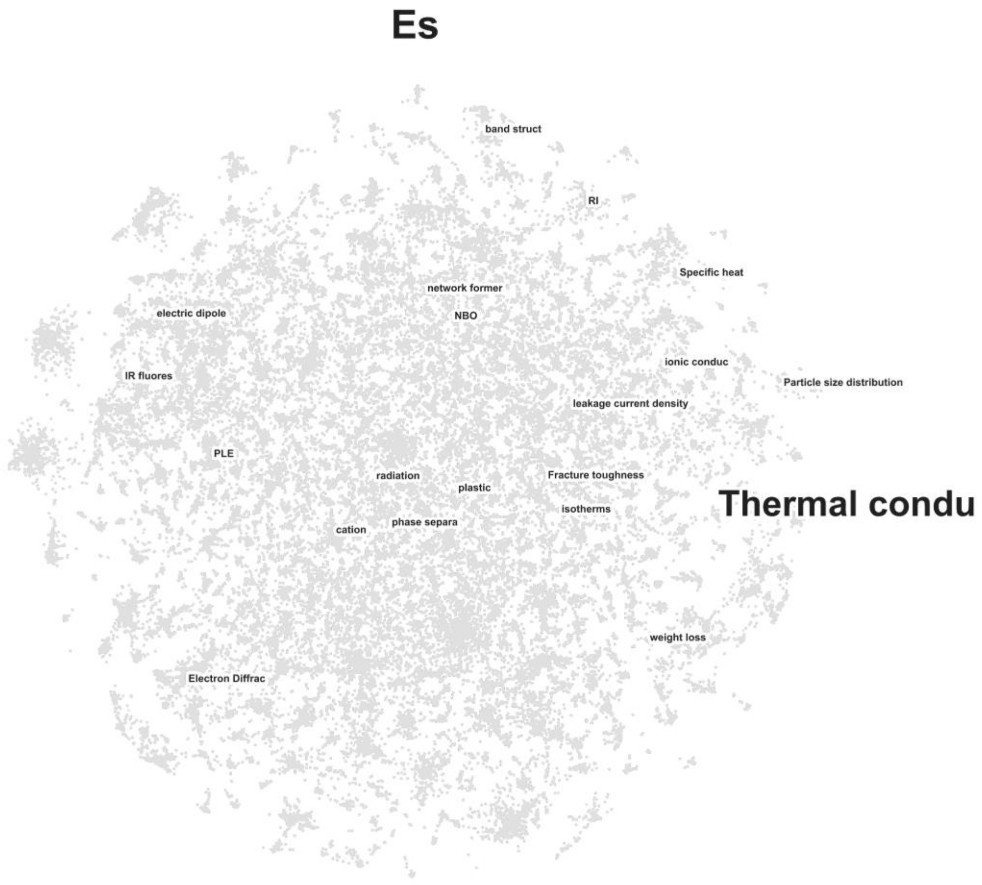

Figure S203. Latent Dirichlet Allocation plot for Einsteinium.

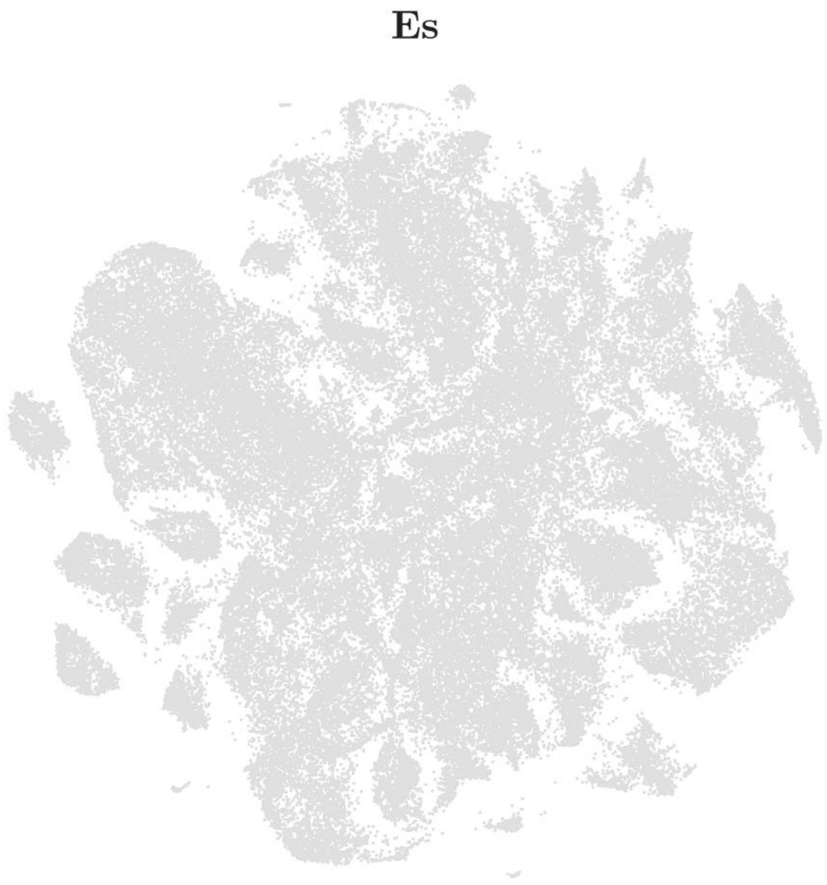

Figure S204. Caption Cluster plot for Fermium.

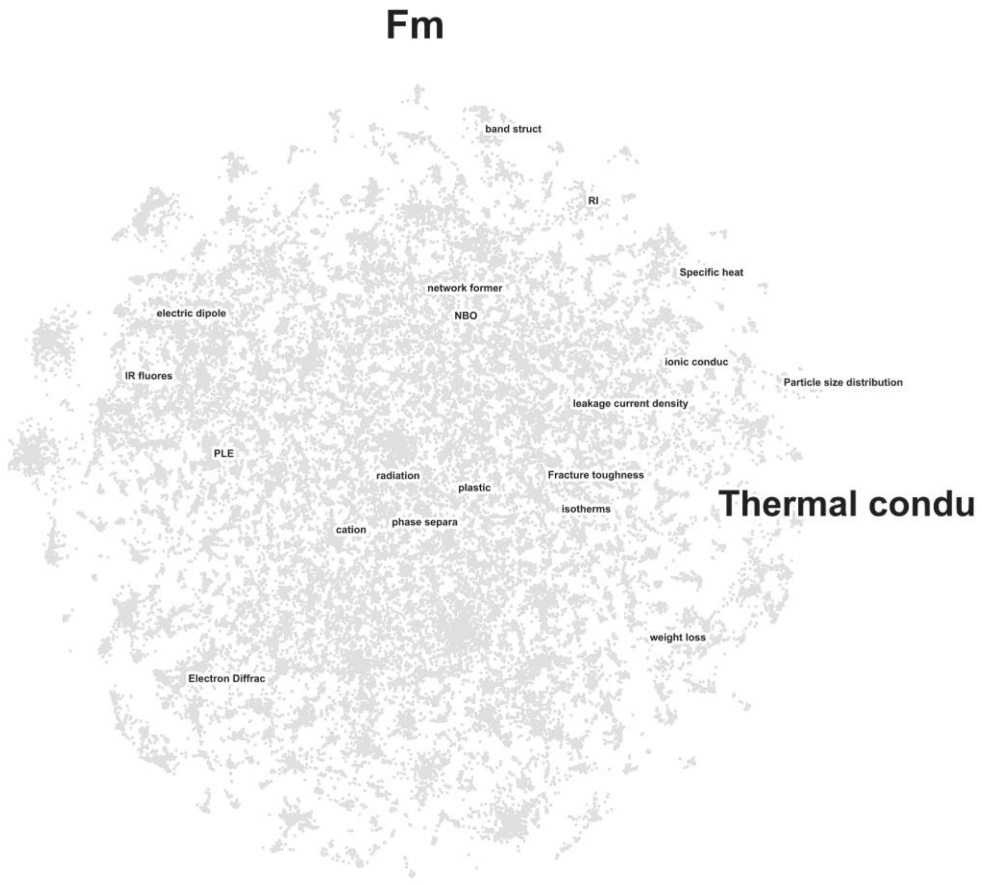

Figure S205. Latent Dirichlet Allocation plot for Fermium.

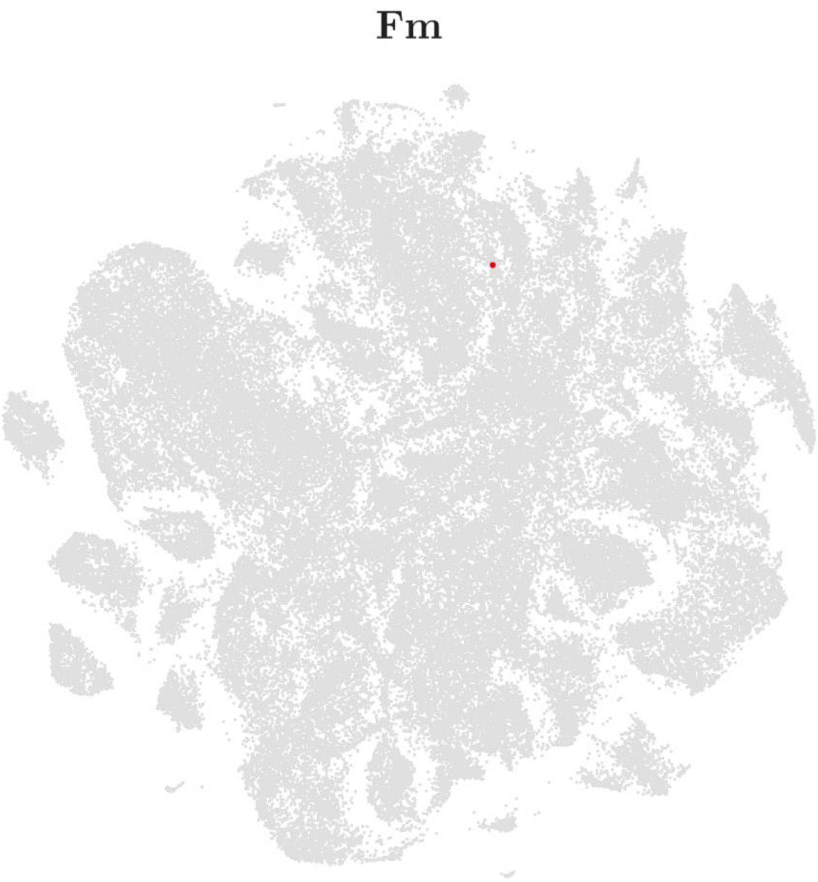

Figure S206. Caption Cluster plot for Mendelevium.

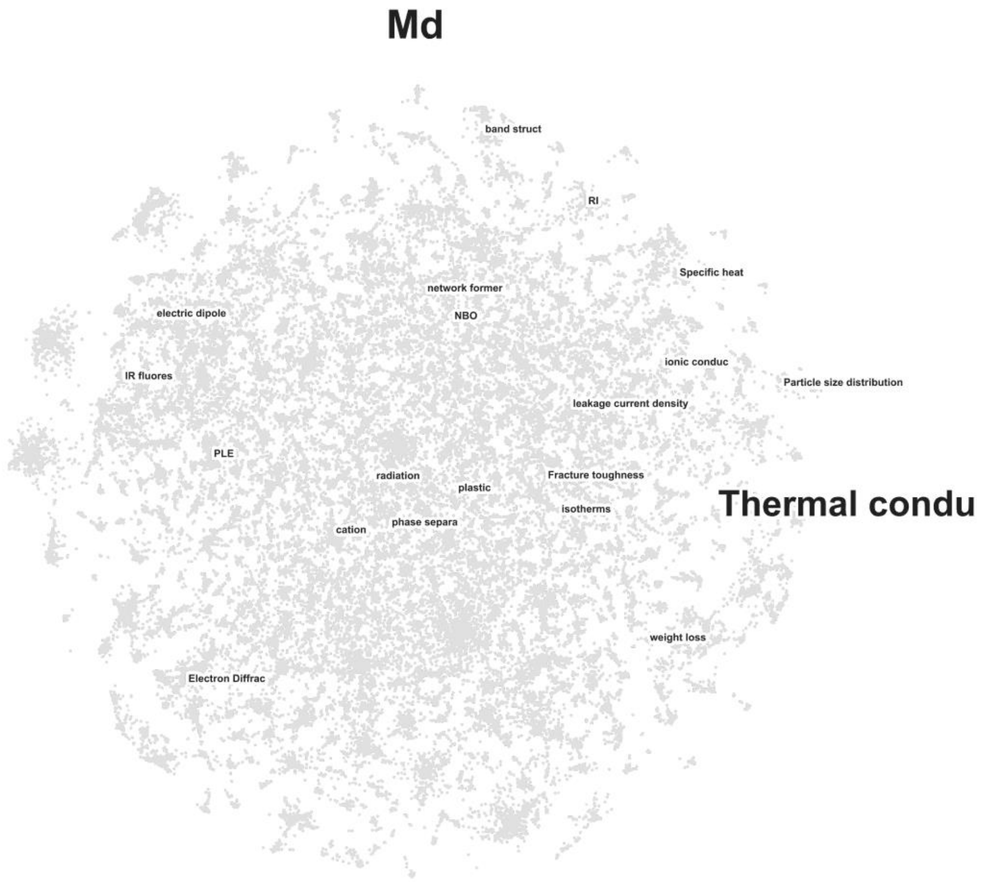

Figure S207. Latent Dirichlet Allocation plot for Mendelevium.

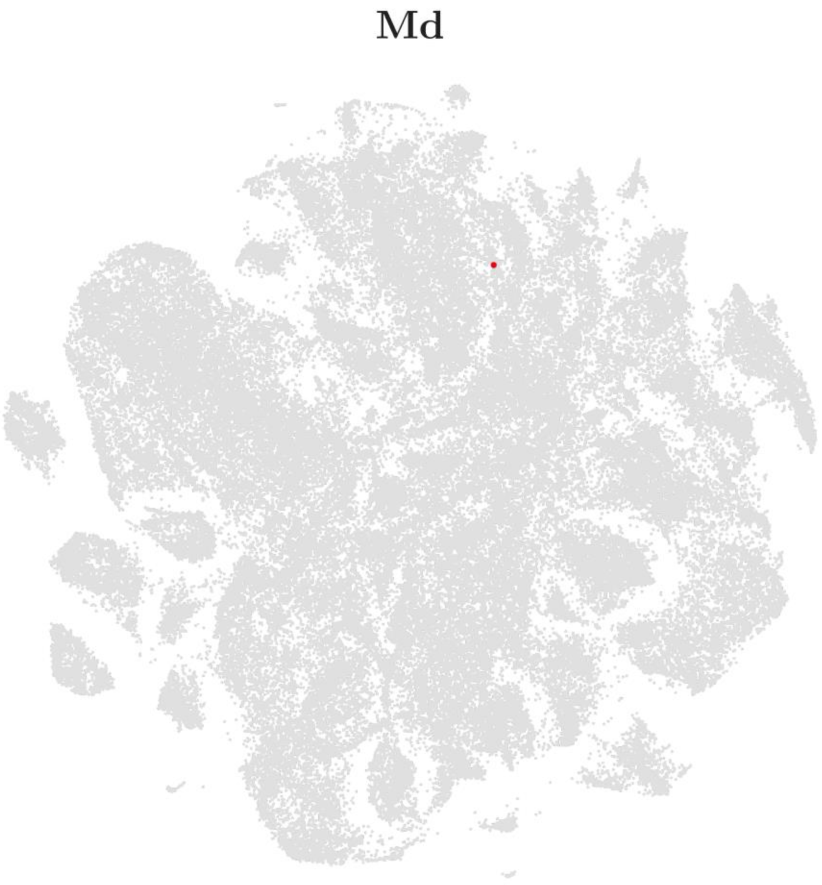

Figure S208. Latent Dirichlet Allocation plot for Nobelium.

Figure S209. Latent Dirichlet Allocation plot for Lawrencium.

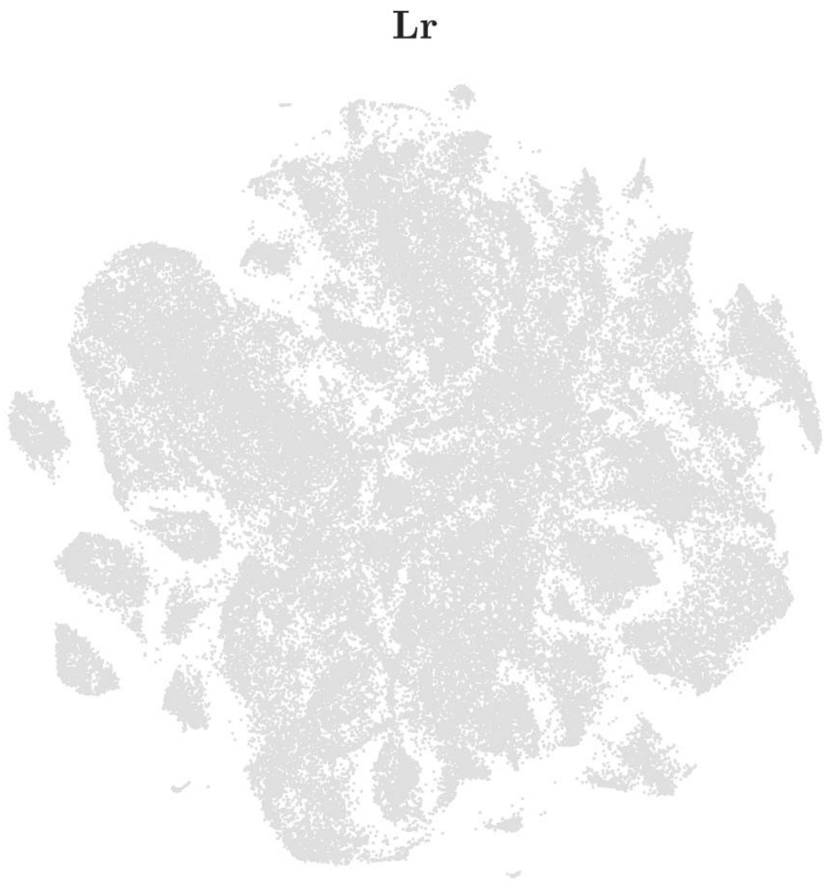

Figure S210. Latent Dirichlet Allocation plot for Rutherfordium.

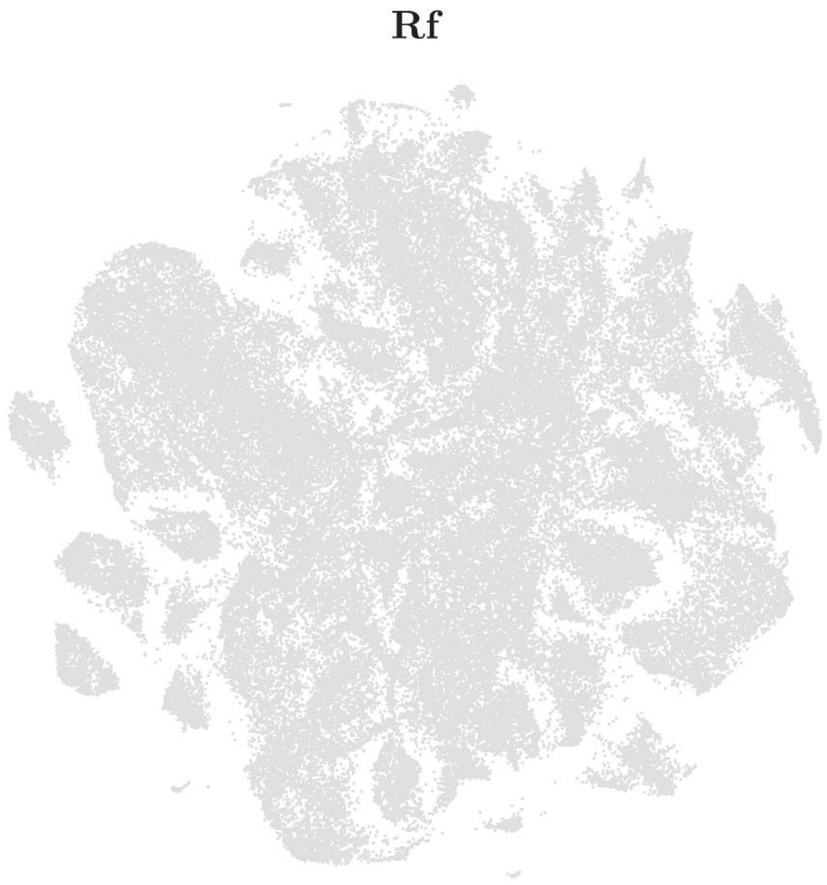

Figure S211. Latent Dirichlet Allocation plot for Dubnium.

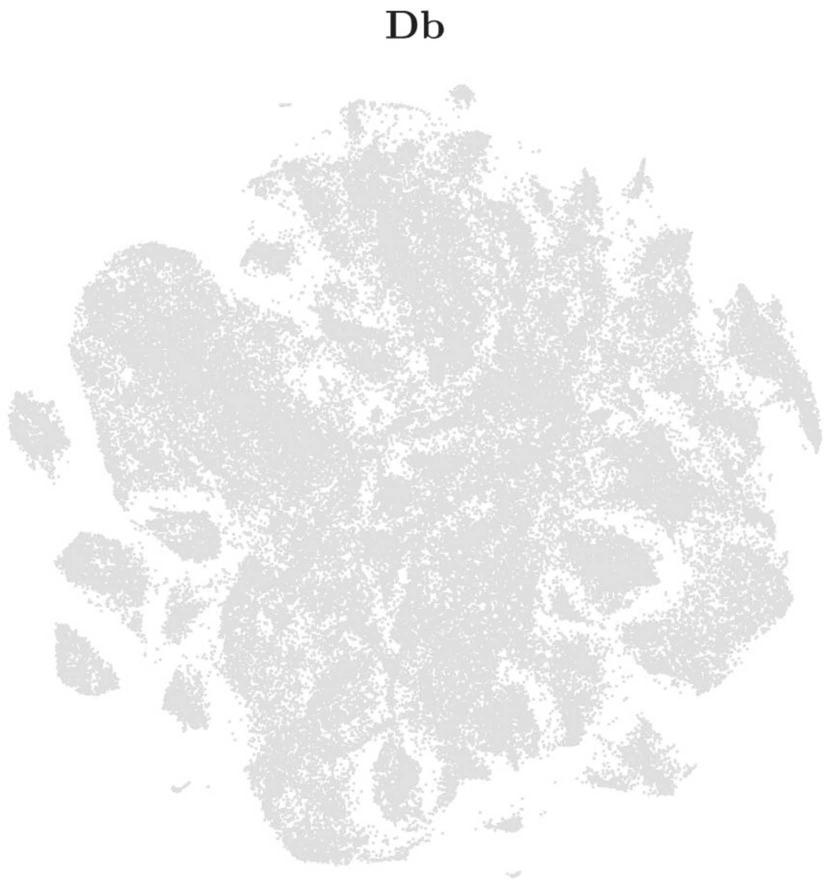

Figure S212. Latent Dirichlet Allocation plot for Seaborgium.

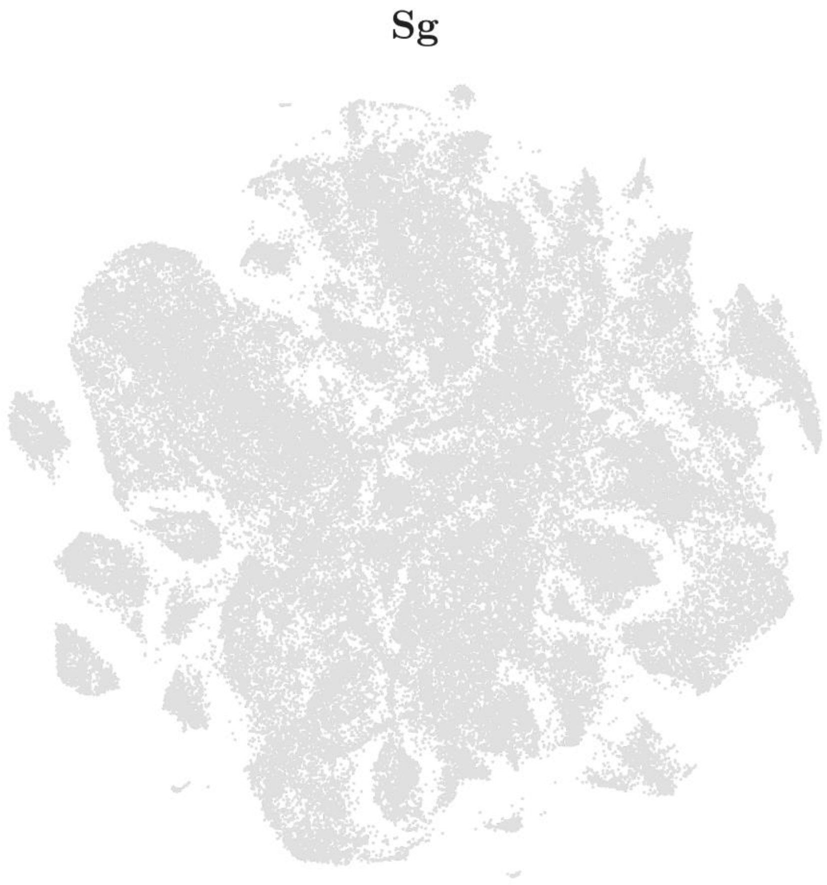

Figure S213. Latent Dirichlet Allocation plot for Bohrium.

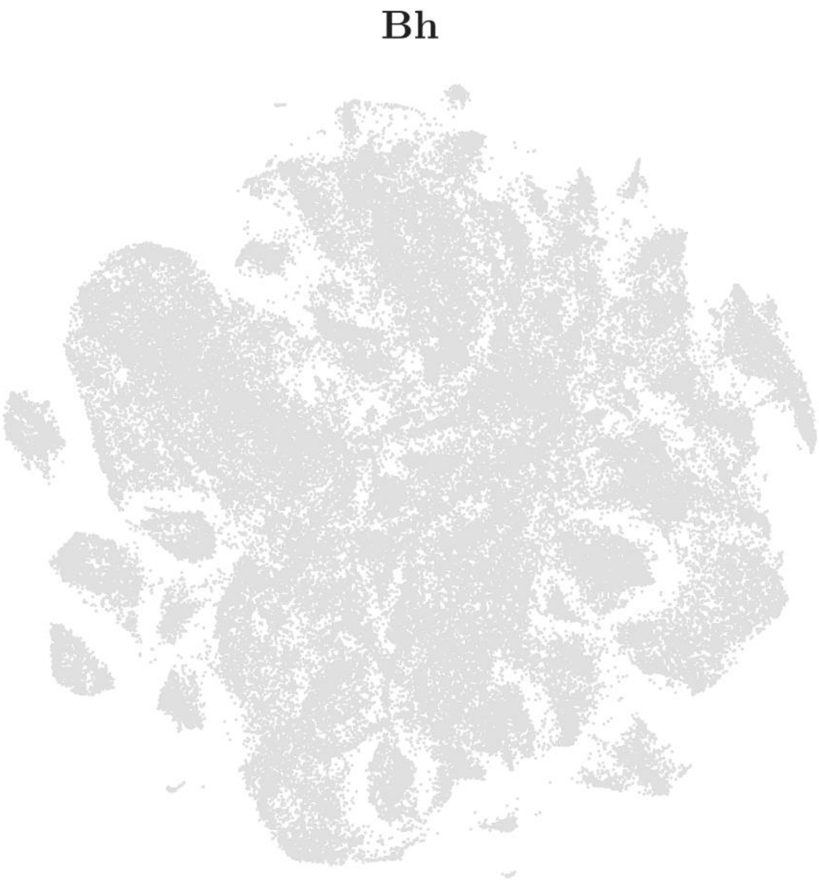

Figure S214. Latent Dirichlet Allocation plot for Hassium.

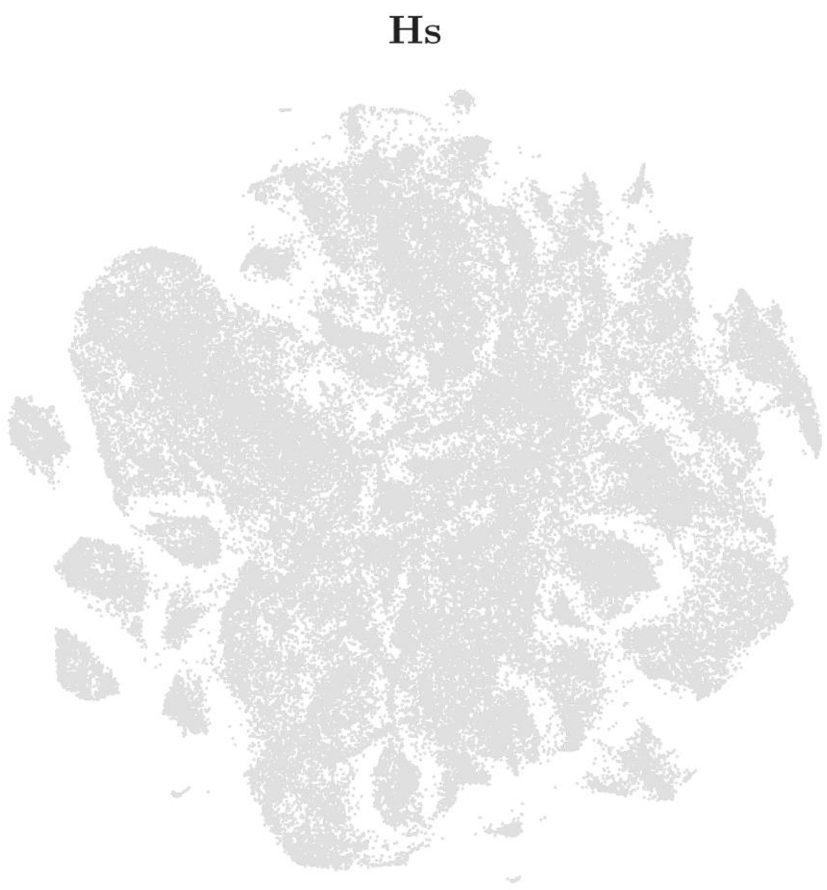

Figure S215. Latent Dirichlet Allocation plot for Meitnerium.

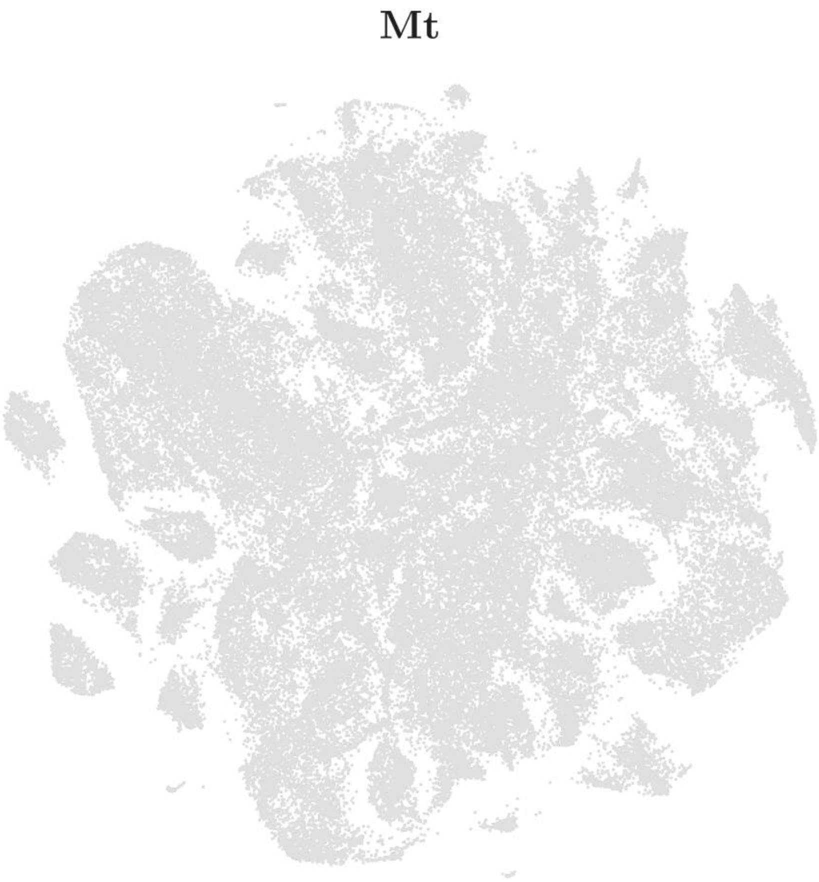

Figure S216. Latent Dirichlet Allocation plot for Darmstadtium.

Figure S217. Latent Dirichlet Allocation plot for Roentgenium.

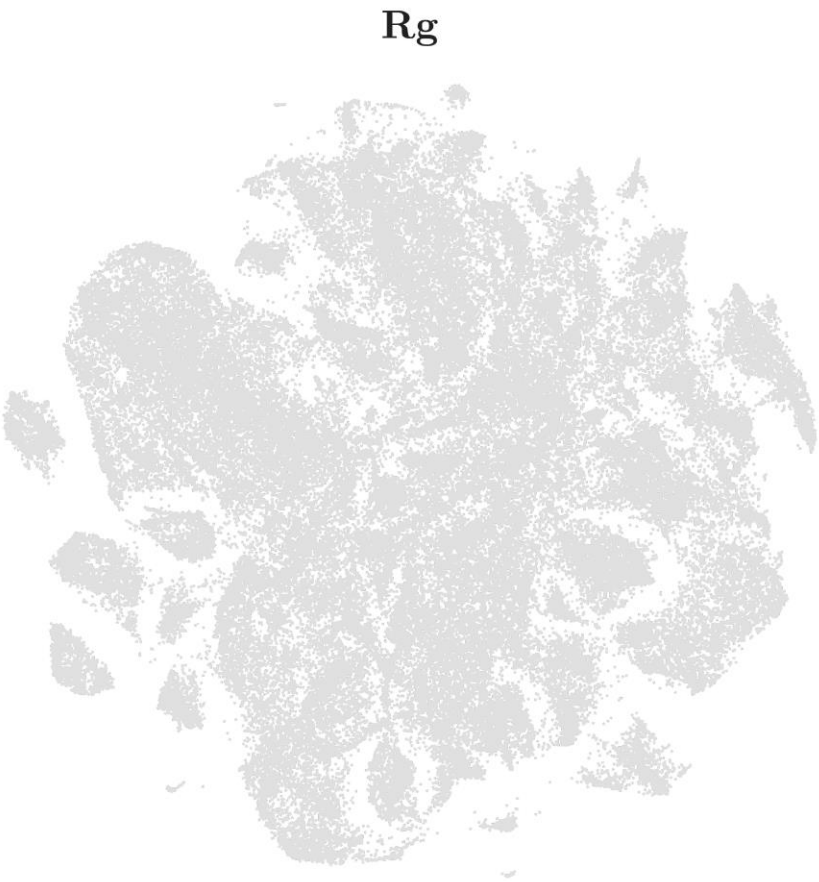

Figure S218. Latent Dirichlet Allocation plot for Copernicium.

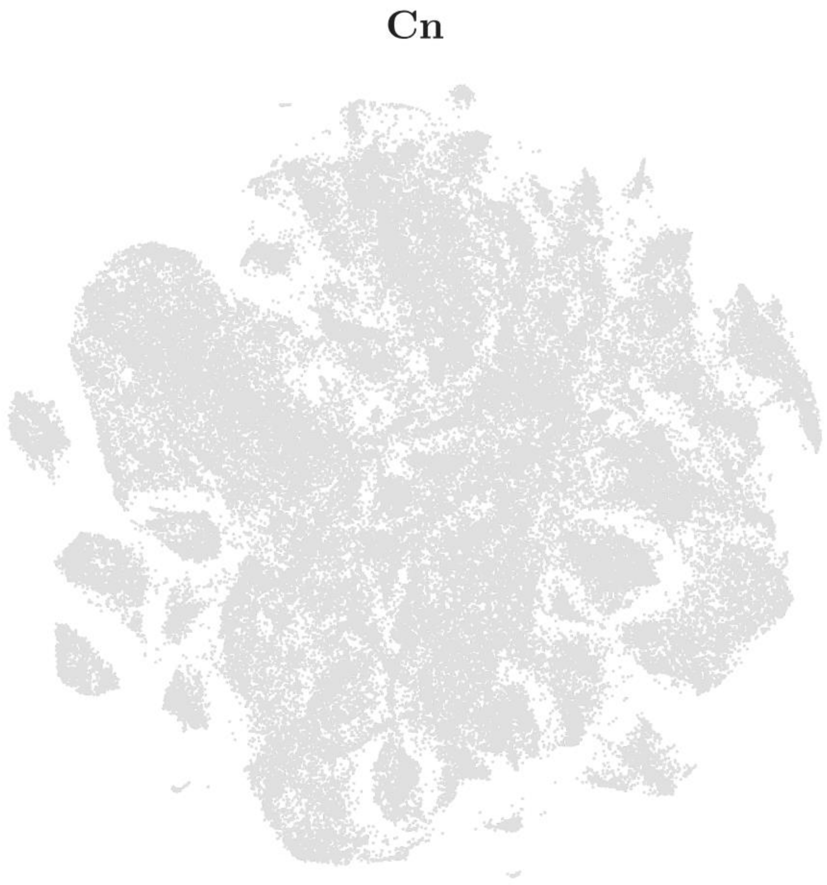

Figure S219. Latent Dirichlet Allocation plot for Nihonium.

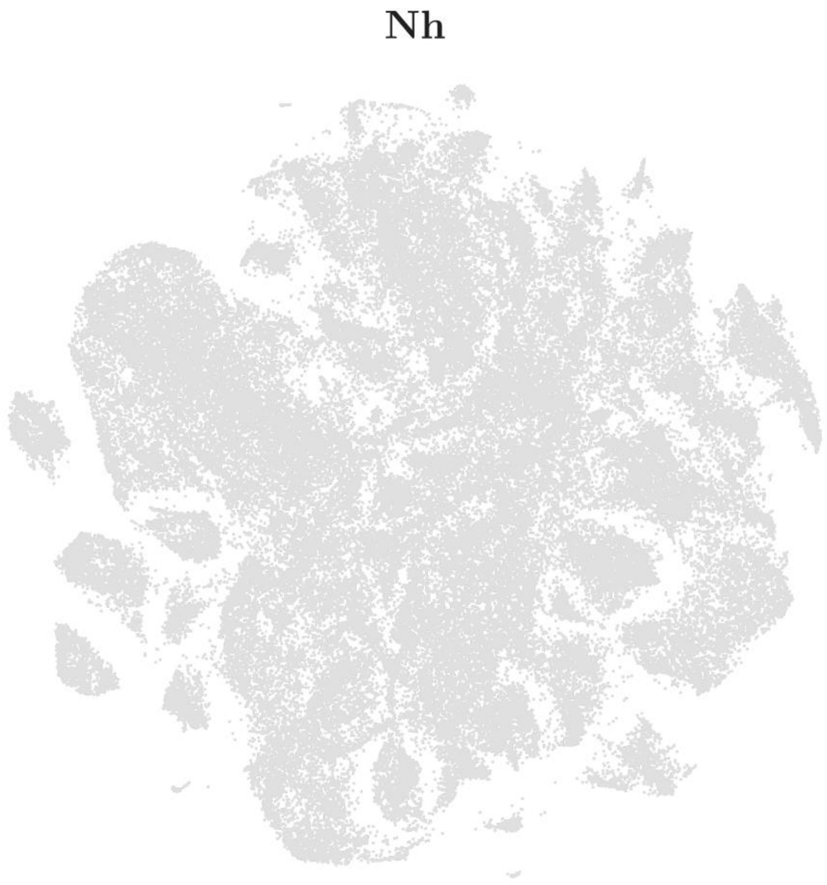

Figure S220. Latent Dirichlet Allocation plot for Flerovium.

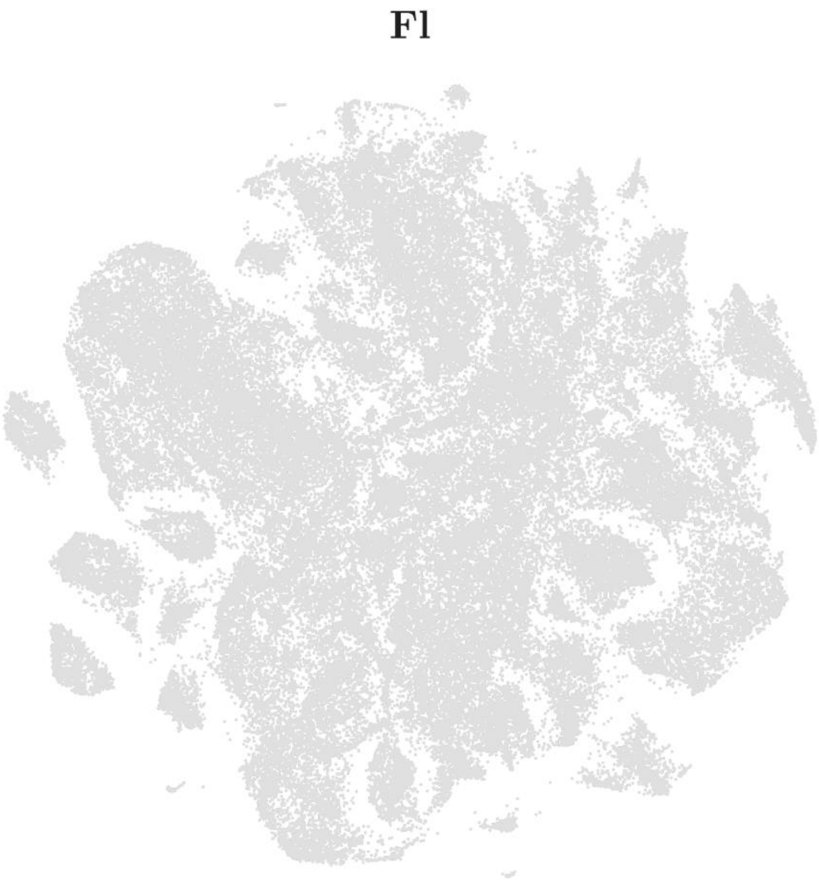

Figure S221. Latent Dirichlet Allocation plot for Moscovium.

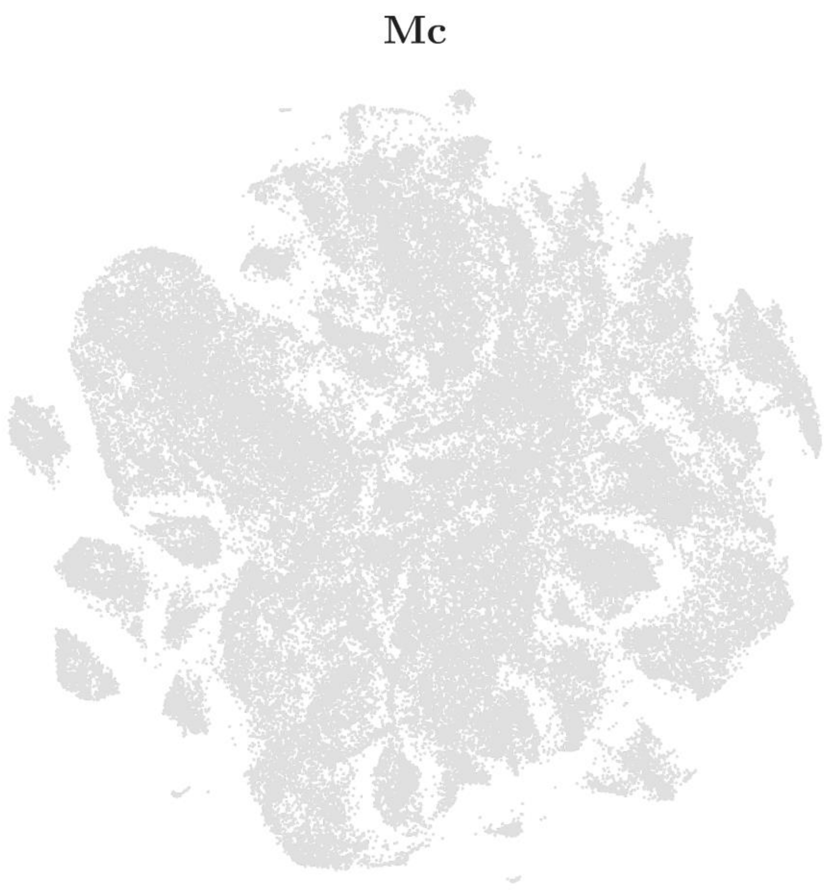

Figure S222. Latent Dirichlet Allocation plot for Livermorium.

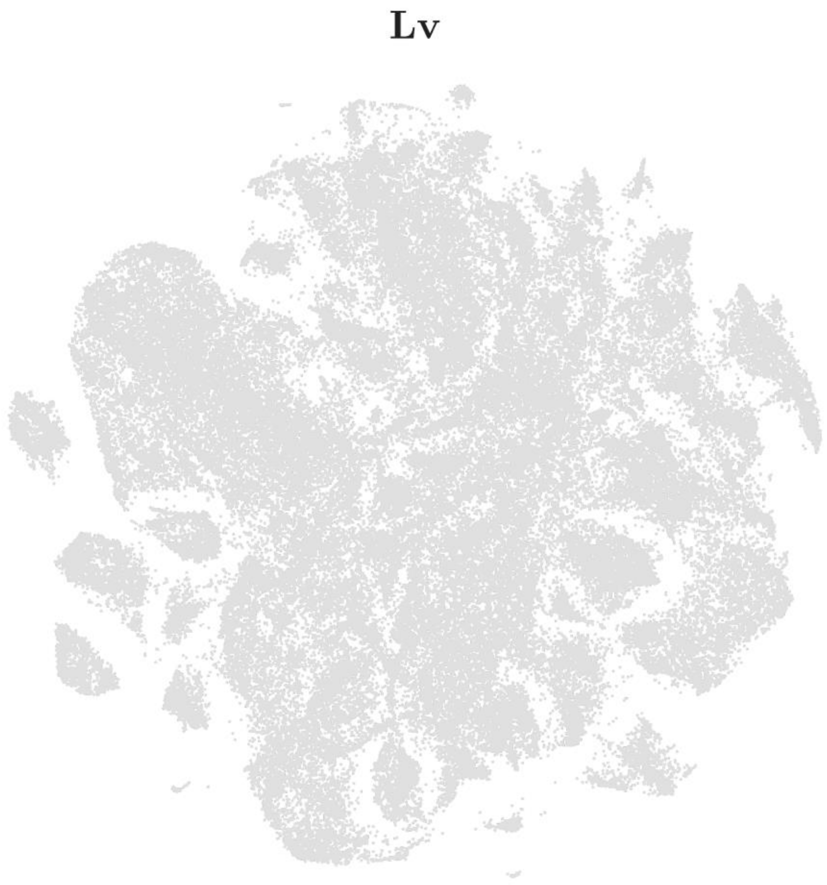

Figure S223. Latent Dirichlet Allocation plot for Tennessine.

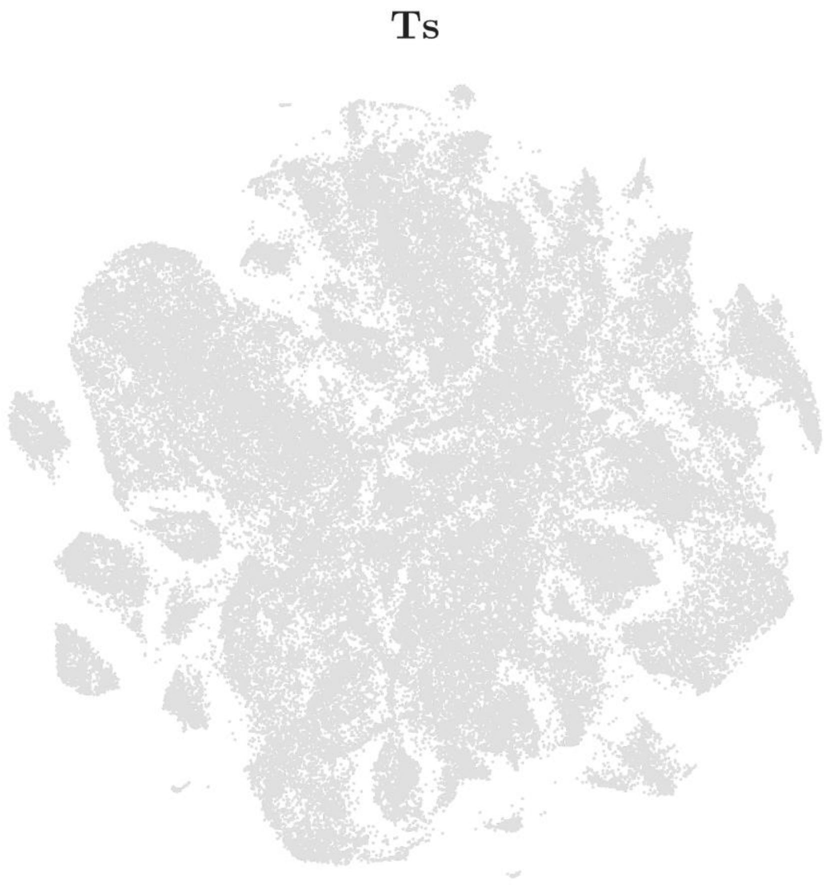

Figure S224. Latent Dirichlet Allocation plot for Oganesson.

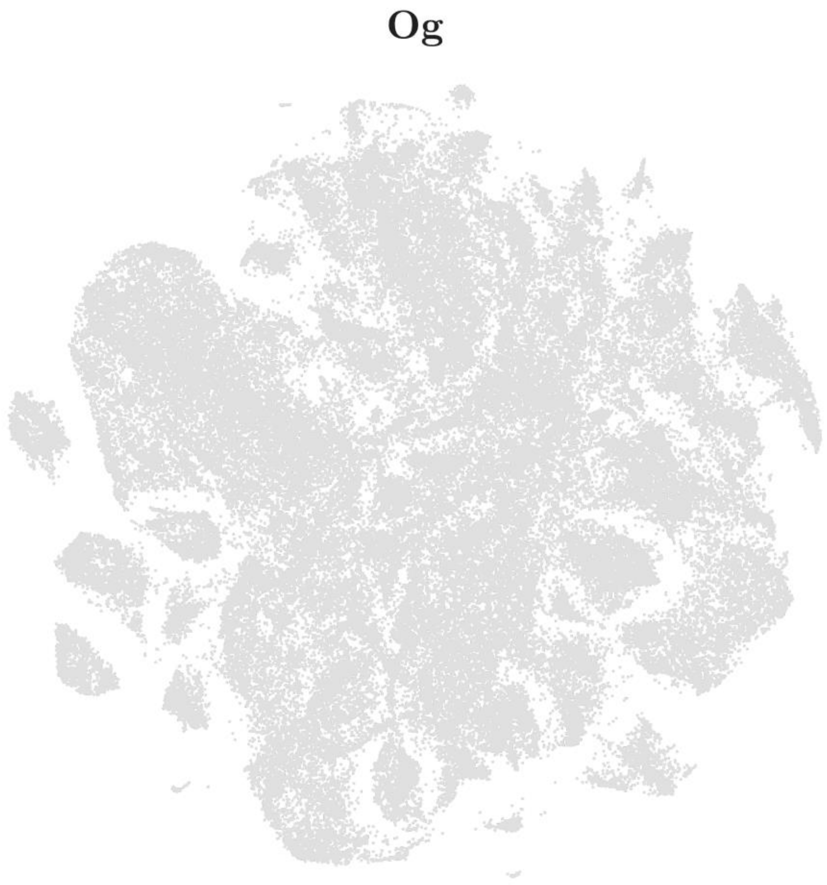

Figure S225. Latent Dirichlet Allocation plot for Ununennium.

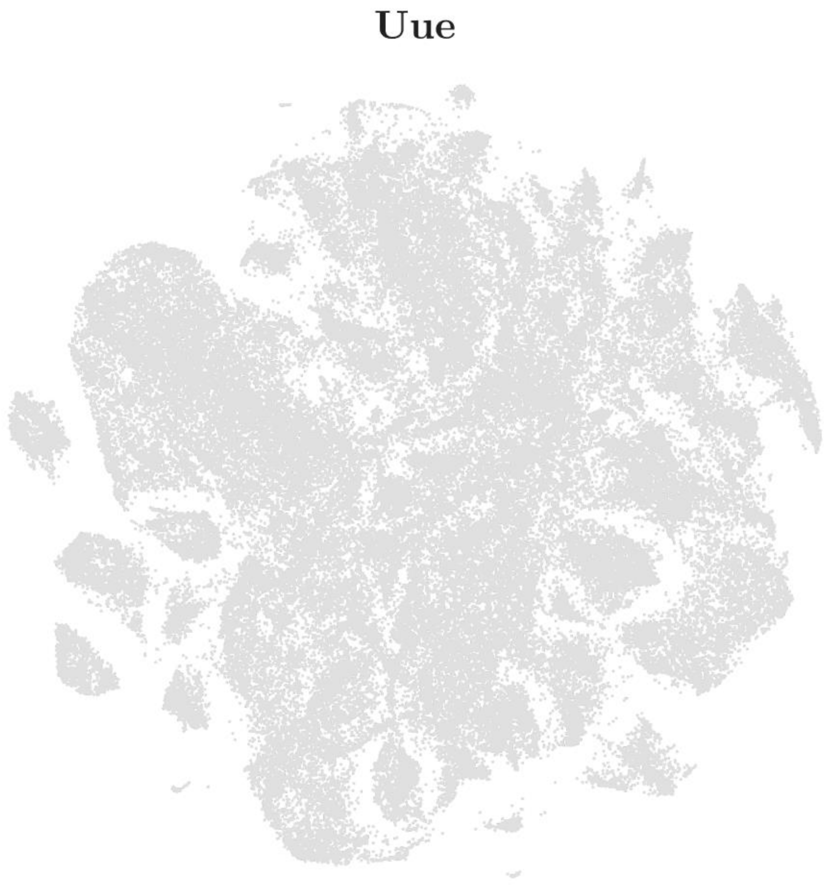

Supplement: Document S1. Supplemental experimental procedures, Figures S1–S225, and Tables S1–S5 [file mmc1.pdf]
