# Supplementary material for: Looking through glass: Knowledge discovery from materials science literature using natural language processing
Source: Patterns (N Y). 2021 Jun 24;2(7):100290. doi: 10.1016/j.patter.2021.100290 (PMC8276010; doi:10.1016/j.patter.2021.100290)
Supplement: Document S2. Article plus supplemental information [file mmc2.pdf]

Article

Patterns

Looking through glass: Knowledge discovery from materials science literature using natural language processing

Highlights

- Natural language processing is used for information extraction from research papers
- Caption cluster plots are used for exploring figure captions across the entire corpus
- Elemental maps are used to identify the chemical elements reported in a study
- A framework to extract domain-specific queries from the literature

Authors

Vineeth Venugopal, Sourav Sahoo, Mohd Zaki, Manish Agarwal, Nitya Nand Gosvami, N. M. Anoop Krishnan

Correspondence

vinven7@gmail.com (V.V.), krishnan@iitd.ac.in (N.M.A.K.)

In brief

This work demonstrates the use of natural language processing tools to extract information from a large corpus of scientific literature in the field of inorganic glasses. The scalable approach presented here combines information from the text and images to answer domain-specific queries, thereby enabling accelerated materials discovery.

Venugopal et al., 2021, Patterns *2*, 100290
July 9, 2021 © 2021 The Author(s).
https://doi.org/10.1016/j.patter.2021.100290

CellPress

Patterns

CellPress
OPEN ACCESS

Article

Looking through glass: Knowledge discovery from materials science literature using natural language processing

Vineeth Venugopal,[1,*] Sourav Sahoo,[2] Mohd Zaki,[1] Manish Agarwal,[3] Nitya Nand Gosvami,[2] and N. M. Anoop Krishnan[1,2,4,*]

[1]Department of Civil Engineering, Indian Institute of Technology Delhi, Hauz Khas, New Delhi 110016, India
[2]Department of Materials Science and Engineering, Indian Institute of Technology Delhi, Hauz Khas, New Delhi 110016, India
[3]Computer Services Center, Indian Institute of Technology Delhi, Hauz Khas, New Delhi 110016, India
[4]Lead contact
*Correspondence: vinven7@gmail.com (V.V.), krishnan@iitd.ac.in (N.M.A.K.)
https://doi.org/10.1016/j.patter.2021.100290

THE BIGGER PICTURE Most knowledge generated through scientific enquiry in materials domain is presented in the form of unstructured data. Among the available sources such as online websites, digital data, and publications, peer-reviewed journals serve as the undisputed source of reliable information regarding materials synthesis, characterization, and properties. Despite the availability of large data, only a limited fraction is compiled in the form of machine-readable databases, most of which are manually curated. Here, applying natural language processing on a large corpus of journal publications on inorganic glasses, we present a framework of information extraction from text and images, which answers queries related to synthesis and characterization techniques, and even chemical elements used. The scalable approach presented here can be applied to other domains for efficient information retrieval from scientific literature.

1 2 3 4 5 **Development/Pre-production:** Data science output has been rolled out/validated across multiple domains/problems

SUMMARY

Most of the knowledge in materials science literature is in the form of unstructured data such as text and images. Here, we present a framework employing natural language processing, which automates text and image comprehension and precision knowledge extraction from inorganic glasses' literature. The abstracts are automatically categorized using latent Dirichlet allocation (LDA) to classify and search semantically linked publications. Similarly, a comprehensive summary of images and plots is presented using the caption cluster plot (CCP), providing direct access to images buried in the papers. Finally, we combine the LDA and CCP with chemical elements to present an elemental map, a topical and image-wise distribution of elements occurring in the literature. Overall, the framework presented here can be a generic and powerful tool to extract and disseminate material-specific information on composition–structure–processing–property dataspaces, allowing insights into fundamental problems relevant to the materials science community and accelerated materials discovery.

INTRODUCTION

The overwhelmingly large amount of knowledge generated through scientific enquiry is mostly stored as unstructured data in the form of texts and images. These range from expository archives, such as books, journals, and dissertations, to condensed representations, such as handbooks and manuals. Materials science, being a highly interdisciplinary area, commands a large repository of scientific publications. However, only a limited fraction of this knowledge is collected and curated in the form of structured data, for example, a database of composition–structure–property relationships. The information on material science is increasingly siloed and simply too large for the efficient utilization of any one individual or group. Just as in all other branches of science, the materials community is afflicted by the curse of knowledge incommensurate with the available information.[1,2]

This is an open access article under the CC BY license (http://creativecommons.org/licenses/by/4.0/).

Thus, the accessibility to the vast majority of knowledge in the literature is limited, as it (1) is time-consuming to manually read and analyze texts and images, and (2) requires a domain expert to understand, interpret, and summarize the information.

Recent advancements in natural language processing (NLP) provide a promising solution to this problem through the automation of text comprehension, querying, and knowledge extraction from scientific texts. NLP has been applied extensively to scientific literature specifically in biological sciences for more than 2 decades.[3–5] A biomedical-specific language model, namely, BioBERT,[4] which is used extensively for biomedical text mining, stands as a testimony to the advances and contributions of NLP in biological sciences. In contrast, the applications of NLP to materials science remain sparse.[6–8] Similar to biological sciences, the study of materials present some unique challenges to the direct application of NLP to mine text data due to the domain-specific jargons and lack of uniform conventions in scientific writing.[6,9] Despite these challenges, recent studies have shown that NLP can indeed be used to address some open challenges in materials science such as novel materials discovery,[7] unraveling synthesis pathways,[10] and extracting composition–property databases.[11]

Cole et al[12–14] have demonstrated the automated generation of databases for magnetic[15] and battery materials[11] using ChemDataExtractor,[16] which has also been used in predicting phase diagrams.[17] Olivetti et al[6,10,18] have used NLP together with artificial neural networks to predict synthesis parameters of inorganic oxides[10,19,20] and in extracting the properties of zeolites[21] and cementitious materials.[22] Jain et al[7] have demonstrated the use of word vectors in converting semantic queries to vector algebra and extended the method to the prediction of thermoelectrics. Ceder et al[10] have shown the extraction of automated synthesis recipes of inorganic oxides through a semi-supervised approach. Recently, Matscholar[9] has been introduced as a comprehensive material science search and discovery engine that is able to automatically identify materials, properties, characterization methods, phase descriptors, synthesis methods, and applications from a given text through a custom-built named entity recognition (NER) system. These developments suggest that artificial intelligence approaches using NLP can be a promising route to condense and represent knowledge in materials science leading to novel materials discovery and development.

Very few studies have, however, focused on extracting information related to images and plots in literature.[23,24] The adage, "a picture is worth a thousand words," is even more relevant to scientific literature, as images hold the most crucial information related to scientific hypothesis and theories.[25] Till date, there has been no framework that allows direct search or compilation of images presented in scientific literature. Further, the images of a manuscript should be read in conjunction with the text to understand the context. While many of the applications of NLP in material science have focused on extraction and processing of textual information, no effort has been made thus far to connect this textual information with the images and plots to allow knowledge dissemination in a holistic manner.

Here, we demonstrate a comprehensive NLP framework that extracts information from a large corpus of text and images to provide highly specific, nuanced, and automated exploration of materials science literature. Specifically, we analyze the texts and images from approximately 100,000 research articles in the area of glasses, an archetypical disordered material. Glasses are one of the most common and widely used among engineering materials with uses spanning architectural, functional, and biomedical applications.[26,27] Recently, machine learning (ML) approaches have been used to develop predictive models for optical, electronic, and mechanical properties of glasses.[26,28–38] Several recent works have shared composition–property databases along with the trained ML models.[28,30,36,39] For instance, the software package, Python for Glass Genomics (PyGGi), has a large composition–property database, ML models for predicting nine key properties and an optimization framework for targeted glass discovery.[40] These models, however, have relied on existing databases for their training and analysis,[41] and hence have been restricted to parameter predictions through regression models. It is well known that the properties of glasses, a nonequilibrium state, are not just a function of composition, but are also fundamentally influenced by the processing history and testing conditions.[27,42–44]

Through a combination of NLP algorithms, chemical entity extraction protocols, and visualization tools, we show that the answers to very specific questions on glass literature can be answered. These include material/property specific questions as well as broader community issues, such as the following:

1. What are the common microstructural characterizations for glasses?
2. Are more of the papers published in glass science theoretical as opposed to experimental?
3. Where is americium used in glasses?
4. What chemical elements have been used in LEDs?
5. Are there photoluminescence studies of bioactive glasses that contain Fluorine?
6. Can we find papers on optical glasses that have been manufactured using solid state synthesis?

Overall, the generic framework developed here allow highly specific exploration of scientific literature using the abstracts, text, and image captions of publications.

RESULTS

Topic modeling

To demonstrate the proposed approach, we downloaded more than 600,000 research articles, full texts, and images related to the keyword "oxide glasses" and "materials science" using the CrossRef metadata query API[45] and the Elsevier Science Direct API.[46] Following this, supervised learning was performed on the abstracts of the manuscripts to filter them (see Methods for details). Abstracts are the most information-dense organ of a scientific paper, containing information on the material under study, property being explored, and characterization/synthesis methods being used in service of the investigation. As such, they are unlikely to contain spurious information or refer to materials or properties not mentioned in the text. This specificity makes an abstract the most useful part of a text, and it is therefore not surprising that many NLP studies on materials have only

A

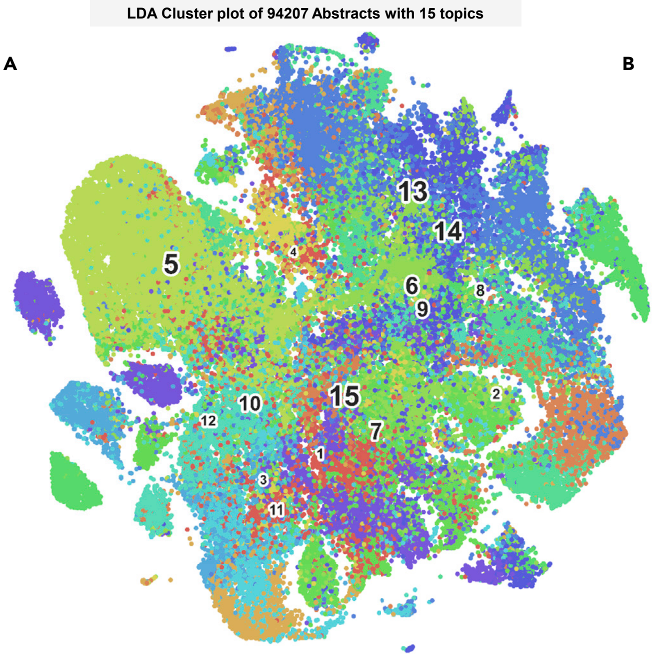

B

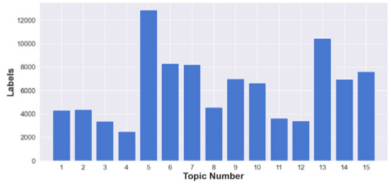

C

Topic	Label
1	Luminescence studies
2	Dielectric properties
3	Spectroscopic properties
4	Irradiation studies
5	Thin films
6	Methodological studies
7	Non-linear optical properties
8	Bioactive glasses
9	Electrical properties
10	Glass nanoparticles
11	Rare earth glasses
12	LEDs and Phosphors
13	Glass ceramics
14	Chemical properties
15	Miscellaneous

Figure 1. LDA plot of the abstracts

(A) The LDA plot presents the clusters of vectorized abstracts colored based on topics identified by LDA.

(B) The number of abstracts on each topic as identified by LDA.

(C) The descriptive label assigned to LDA topics by a human expert.

taken paper abstracts as the input.[7] Based on the supervised learning, approximately 100,000 research articles were classified as relevant to the topic "glass," with precision, accuracy, and recall of 92%, 86%, and 67%, respectively, on the test set. Note that the model with the highest recall was selected to include as many glass-related articles as possible. Although not an exhaustive list, the total number of articles downloaded are in the same range as the number of texts identified in other comprehensive literature surveys on glass.[47,48]

An unsupervised NLP algorithm called the latent Dirichlet allocation[49] (LDA) was used to automatically classify the corpus into 15 "topics," where each topic is defined by the set of words that have the highest probability of occurrence within the topic. LDA allows a rapid and efficient organization of the text corpus with minimal human supervision—a capability provided by no other automated tool available today. The categories generated by LDA are visualized in the LDA plot in Figure 1A. Each abstract in the corpus is vectorized using Term Frequency–Inverse Document Frequency[50] (TFIDF), which maps each document in the corpus to a unique vector in a higher dimensional space. T-distributed stochastic neighbor embedding (t-SNE) projects these vectors to a 2-dimensional (2D) plane such that vectors with the highest cosine similarity group together. The color of a pixel is determined by the topic number assigned to it by LDA.

It is seen immediately from Figure 1A that the points with similar color are grouped together. This suggests that the TFIDF vectorization followed by t-SNE clustering is able to group the abstracts with similar topics, as identified by LDA. The LDA plot is a graphical representation of the entire field of glass literature and succinctly summarizes the details mentioned earlier.

Note that the descriptive label is assigned to these topics by a human expert that maps the automatically generated lexical probability distribution to established categories in glass literature. For example, the words with the highest probability of occurrence in Topic 11 are "er," "yb," "emission," "doped," "luminescence," "nd," and "tm." Analysis of these high-frequency words by human experts suggests that the topic is related to the luminescence of glasses doped with rare earth ions, and hence it is labeled as "Rare Earth glasses." The distribution of abstracts into the identified topics is shown schematically in the histogram in Figure 1B. The descriptive labels for all the other topics are similarly identified and listed in Figure 1C.

A mere visual inspection confirms that Topic 5–thin films–is the single largest group followed by topics 13 and 6. The most common set of articles related to "Thin films" includes both the luminescence properties of oxides such as ZnO on glass substrates, as well as the studies of transparent glasses on a thin film geometry. This is followed by "glass ceramics," "methodological studies" of glasses (including both theoretical and modeling studies), and "nonlinear optical properties of glasses." In general, the list is found to be comprehensive covering all facets of glass literature in terms of applications, properties, and ingredients including bioactive glasses, dielectric glasses, nanomaterials, and chemical and electromagnetic properties. Other categories, such as mechanical and failure studies of glasses, are seen to be subsumed within these broad topics. Note that a more detailed classification can be further performed to obtain the subtopics by performing LDA recursively on each of the topics separately (see Figure S1 and Table S1).

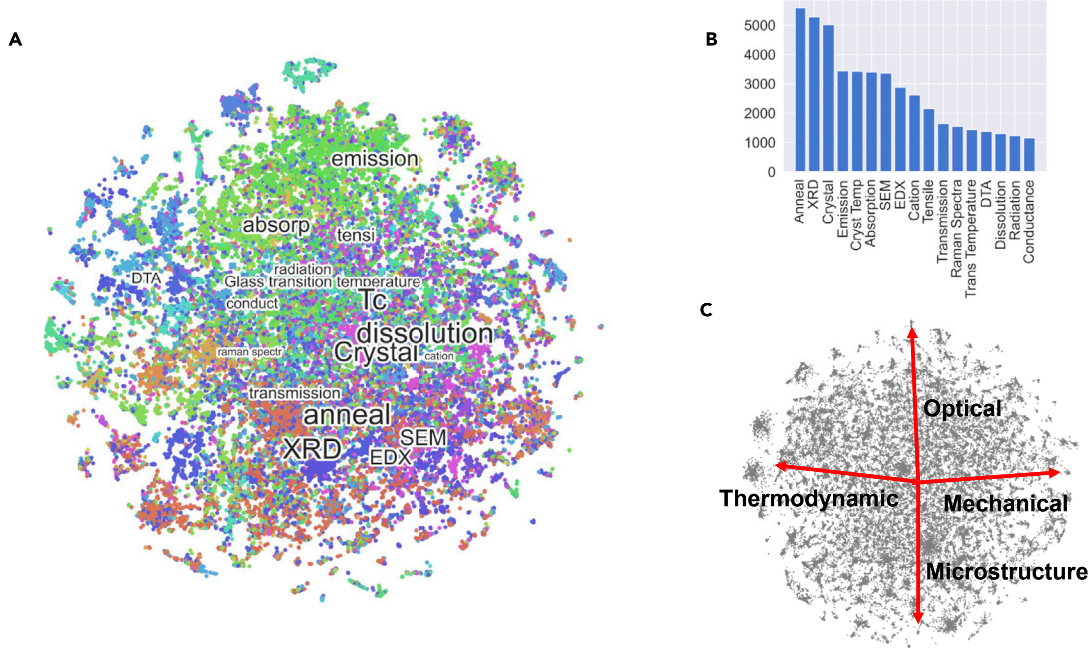

Figure 2. CCP

(A) The CCP presents the clusters of vectorized captions colored by preselected keywords.

(B) The labels with the highest number of counts in the caption database.

(C) A grayscale image of the plot showing the four ontological axes.

Caption cluster plots

The corpus also contains a collection of 106,238 figures and their captions. It is well known that the information content of scientific articles is expressed mostly through graphics. These images and their corresponding captions, therefore, provide a technical summary of the documents that they belong to. The caption cluster plot[25] (CCP) shown in Figure 2A is a graphical representation of the information contained in all the captions, grouped by their semantic similarity using NLP. The captions are tokenized and vectorized using TFIDF and t-SNE, as explained earlier for the LDA plot. The pixels are colored based on categorical keywords identified by a human expert as explained in the methods section. Finally, these labels are positioned at the median (x,y) positions of the corresponding pixels, with the size of the label proportional to the number of images in that category.

Figure 2B shows the distribution of captions with respect to the topics for each image type identified in the CCP. We observe that "Anneal," "X-ray Diffraction" (XRD), "Crystal," "Emission," and "Crystallization temp" (Tc) are the most common types of images in glass literature, underlining the importance of thermal synthesis routines, microstructure, and optical characterization methods in glass science. Captions representing similar types of images are found to cluster next to each other. For example, microstructure measurements such as scanning electron microscopy (SEM), XRD, atomic force microscopy (AFM), energy dispersive X-ray spectroscopy (EDX), and transmission electron microscopy (TEM) all cluster at the lower half of the CCP while optical properties, such as emission, absorption, fluorescence, and luminescence, are found at the very top. The use of TFIDF as the vectorization algorithm ensures that labels, and hence the captions representing images, are found to group together organically, based on their semantic similarity. The CCP of Figure 2 immediately informs us the answer to the first question raised in the introduction: "what are the most common characterization methods for glasses?" The answer is seen to be XRD and SEM. Also, most of the larger tags in the image are experimental methods, as opposed to modeling techniques like finite element methods or molecular dynamics that are too small to be seen unaided. This in turn, informs the second question: most of the papers in glass literature are clearly experimental.

Interestingly, the CCP is found to have four distinct axes that capture complementary information on glass literature. These are the Optical, Mechanical, Microstructural and Thermodynamic axes, as shown in Figure 2C. Optical axis are represented by images related to "emission," "luminescence," and "fluorescence," to name a few. Images that study mechanical properties, such as "crack," "stress-strain," "strength," and "compressibility" are found closest to the Mechanical axis, while thermodynamic properties, such as glass transition temperature, activation energy, specific heat, differential scanning calorimetry, and differential thermal analysis (DTA), to name a few, lie along the Thermodynamic axis. Finally, the images related to "XRD," "EDX," "anneal," and "Crystal" fall along the Microstructural axis. This observation can be formalized by computing the Euclidean distance between caption labels and the four axes. The thermodynamic properties are closest to the Thermodynamic axis, while the optical properties are most proximate to Optical axis, etc. This observation is a direct result of using the t-SNE algorithm based on the cosine similarity of caption vectors, which ensures that the Euclidean distance between pixels is a measure of the semantic similarity of the underlying captions.

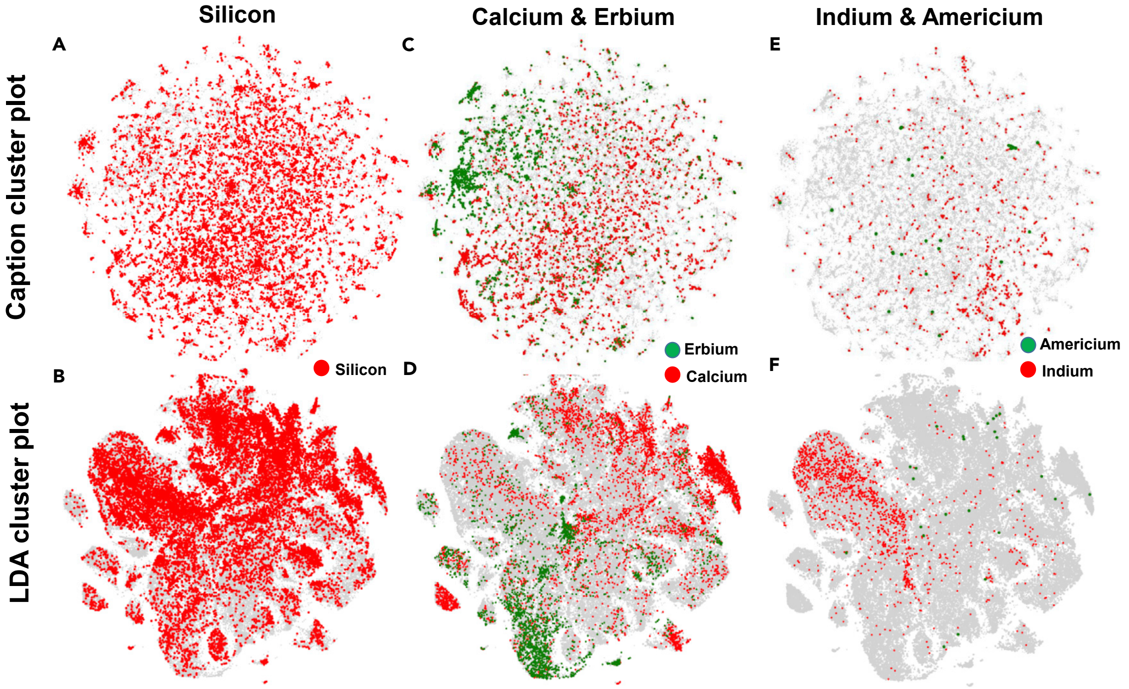

Figure 3. Elemental maps

The elemental maps of (A) Si, (C) Ca and Er, and (E) In and Am superimposed on the CCP showing the presence of the respective elements in the abstracts of the manuscripts from which the images are taken. The elemental maps of (B) Si, (D) Ca and Er, and (F) In and Am superimposed on the LDA plot showing the abstracts where the elements have been identified to be present.

Certain captions do not belong to any of the axes but are found to be equally spaced from two or more of them. For example, ‘‘Fracture’’ and ‘‘Interface’’ are located at nearly equal distances from the Mechanical axis and Microstructural axis. These terms represent microstructural features that are critical determiners of mechanical properties. The captions likely contain terms that relate equally to both axes categories, justifying their position in the CCP. The position of cluster labels is an indicator of their relative co-occurrence frequency, thereby showing that ‘‘anneal’’ is strongly related to ‘‘Crystal’’ (and variations thereof including ‘‘crystalline’’ and ‘‘crystal structure’’), while ‘‘PLE’’ is more related to ‘‘absorption’’ than to ‘‘emission.’’ At the same time, the proximity of ‘‘bioactive’’ to the Mechanical axis suggests that many experiments on bioactive materials pertain to the measurement of strength and hardness thereby bringing the captions into semantic convergence. Overall, we observe that the CCP provides invaluable insights into the contents of these figures, which when combined with a predefined ontology solve many of the problems stated earlier. In particular, the analysis of captions through CCP provides a visual tool that rapidly summarizes the entire field of glass literature, allowing a user to quickly comprehend the trends, themes, and common characterization methods in the community.

Elemental maps

Next, the Python library, ChemDataExtractor, was used to automatically extract chemical species—including names of compounds, chemical formulae, and symbols—from the abstracts. The chemicals that occur with the highest frequency in the database are shown in Figure S2. The chemical names and symbols were standardized following which chemical elements present in each compound were separately identified. This creates a binary marker for each element such that if the element is mentioned in the abstract, the marker assumes the value 1 and 0 otherwise. The LDA plot is redrawn such that only if the abstract contains the marker for an element is the corresponding pixel colored. Similarly, if a caption is drawn from a text wherein the element is contained in the abstract, the pixel is marked with a color in the CCP. The results are the elemental maps given in Figure 3, where the images at the top are elements mapped to the CCP and the images at the bottom are maps of LDA. The elemental maps provide a direct visual representation of the distribution of elements in glass literature. The juxtaposition of these maps with the caption plot and the LDA provides a highly specific graphic tool to analyze the intersection of selected chemistries with a topic in glass science or a specific property/characterization technique.

As a validation of this concept, it is seen easily from Figures 3A and 3B that silicon is abundantly distributed among all topics, properties, and characterization methods in glass literature. A similar result can be seen for oxygen (see Figures S20 and S21 of supplemental information). This is hardly a surprise, as silicate glasses are one of the most common family of inorganic glasses. The map for calcium and erbium in Figures 3C and 3D is more illustrative. Calcium is found to be less uniformly distributed than silicon, with high concentration in the clusters identified

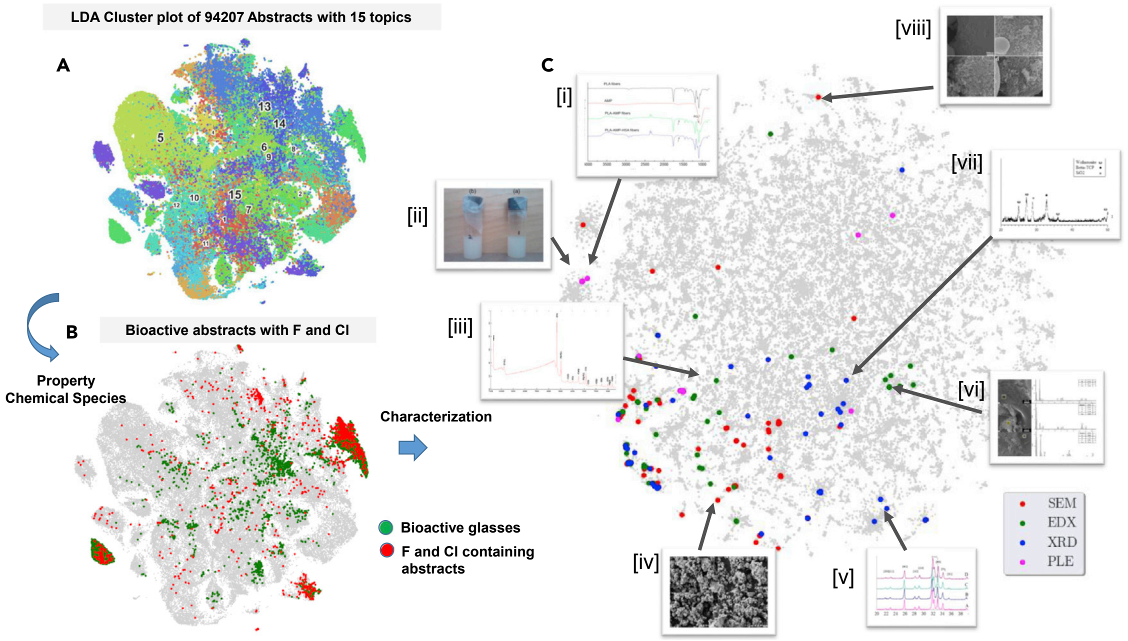

Figure 4. Knowledge extraction combining CCP, LDA, and elemental maps

(A) The bioactive glass cluster is identified from the LDA plot.

(B) The abstracts that contain F and Cl are marked on the plot in red. The area of overlap are abstracts on bioactive glasses that contain F and Cl.

(C) The SEM, EDX, XRD, and PLE images from journal articles belonging only to this parameter space are marked by colored pixels in the CCP. Inset images (i–viii) are arbitrarily selected examples of these images as identified by the plot.[32,39,51–55]

as bioactive by LDA. This is a representation of the fact that calcium is one of the major constituents of bioactive glasses. The overlapping data for erbium in Figures 3C and 3D show that this element is mostly present in the LDA topic ‘‘rare earth glasses’’ and in the caption clusters ‘‘emission,’’ ‘‘PLE,’’ ‘‘band structure,’’ and ‘‘energy diagrams.’’ The elemental map, therefore, provides a visual diagram of the presence of an element in glass literature. Rare earth elements such as erbium, dysprosium, and ytterbium are used largely for LEDs and laser applications, which is confirmed by the region of the LDA plot that is highlighted by these elements (‘‘Rare earth glasses’’ and ‘‘LEDs and Phosphors’’), as well as by the image categories where they predominate.

The third question in the introduction ‘‘Where is americium used in glasses’’ is answered in Figures 3E and 3F. Only 25 articles were found with americium mentioned in the abstract. They are seen to fall over regions identified as ‘‘glass ceramic’’ and ‘‘glass irradiation studies’’ from the LDA plot. Upon inspection, it is indeed found that most of these abstracts relate to studies of nuclear exposure and radiation on glass ceramics, demonstrating a practical use of the concepts developed so far in answering a question that is otherwise not reachable through any other approach today. Similarly, indium is found to be distributed in Figures 3E and 3F mostly in the section identified as ‘‘Thin films’’ relating to the large body of work that has been carried out on the transparent conducting indium tin oxide electrodes on glass substrates and glass ceramics. The elemental maps for all the 120 known elements overlapping with the LDA and CCPs are presented in section 2 of supplemental information (see Figures S6–S225).

DISCUSSION

The LDA and CCPs provide a highly specific, detailed, and succinct graphical summary of the available corpus of glass literature. The knowledge extraction pipeline for obtaining these plots has been shown in Figure 6 (see Methods). They provide answers to some of the questions raised in the introduction. For example, the CCP visually conveys the fact that the most studied aspects of glasses in literature relate to their annealing behavior and microstructure, and that studies on optical emission are slightly more common than that of absorption. The database generated through the CCP provides a useful source of specialized images—say SEM microstructural images or AFM images of glasses—which can then be used for learning images through artificial intelligence and ML algorithms such as convolutional neural networks. These can be used to answer some of the pressing problems in the field today such as identifying the causes of fracture or dissolution by linking to other structure—processing parameters.

Similarly, the LDA plot provides insights on the broad themes within glass ontology into which the text is divided. It is seen immediately through visual inspection that the amount of work done on bioactive glasses is less than that on glass ceramics or that irradiation studies on glasses occupy the bottom of glass hierarchy in terms of the sheer number of publications. The LDA plot is a topological map of glass literature, where each text is assigned a unique position next to other works of similar nature, content, and theme. This provides a way to efficiently search for publications that are similar to a given paper—once the position of the publication in the vector space is determined, the nearest

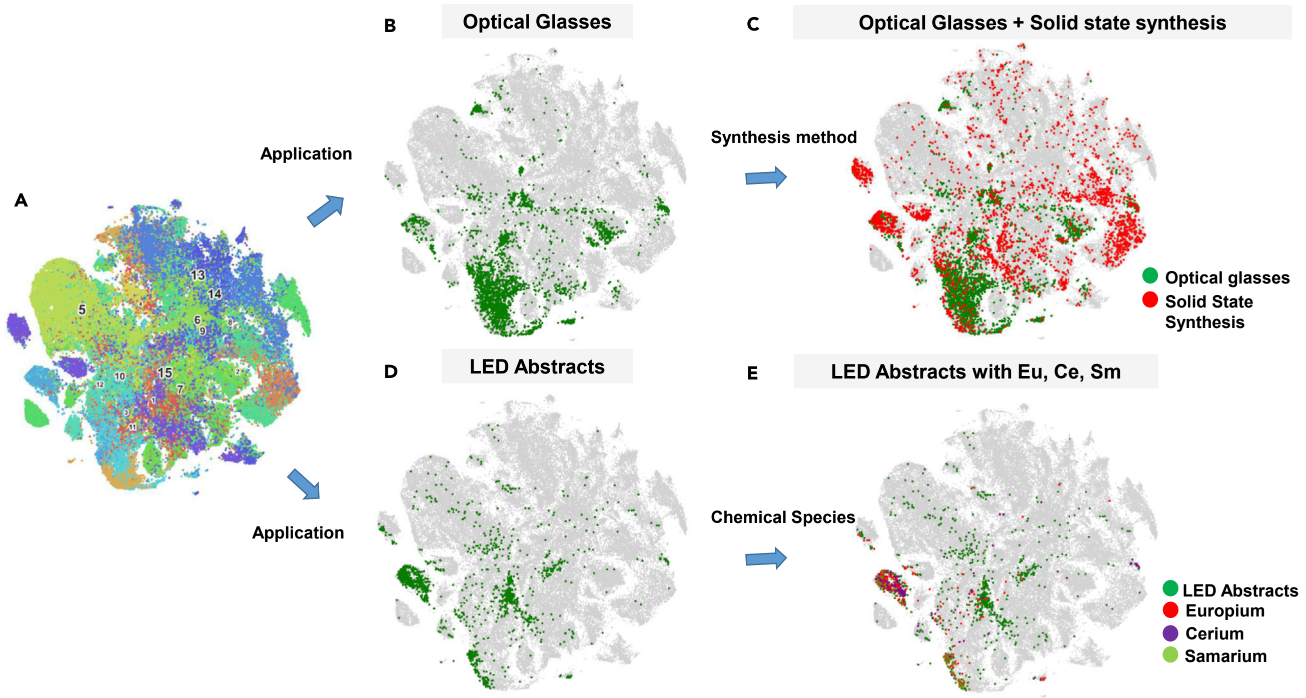

Figure 5. Knowledge extraction framework
(A) The LDA plot.
(B) Abstracts with "optical glasses" in the text.
(C) Abstracts with "solid state synthesis" in the text overlapping with optical glasses.
(D) Abstracts with "LED" in the text.
(E) Abstracts with rare earths.

neighbors are by default the ones that are the most semantically similar. There are currently no other tools, even in established scientific databases and search engines, that allow the detailed exploration, analysis, and easy visual analysis offered by the CCPs and the LDA plots. At the same time, combining the caption cluster, LDA, and elemental plots results in the creation of a tool that can query, explore, and analyze the information content in glass literature with unprecedented detail and specificity.

An example toward such an attempt for knowledge extraction and dissemination combining CCP, LDA, and elemental map is presented in Figure 4. The LDA plot identifies abstracts in the database that belong to the category of "bioactive glasses." The elemental maps allow the selection of only those abstracts among these that have been marked with the presence of fluorine and chlorine. This is shown in Figure 4B where the red pixels corresponding to the F and Cl containing abstracts are found to overlap with the green pixels corresponding to abstracts on bioactive glasses. The region of the greatest overlap is seen to be the three islands on the LDA plot that are marked as bioactive glasses, thereby confirming that among all fields of glass science, F and Cl most commonly find their application within this topic. This in itself is a remarkable capability—the identification of only those scientific publications subject to the dual constraints of topical category and chemistry. It bears repeating that there is no other method currently available that can do this.

Adding the CCP to this information allows even deeper exploration of literature by extracting a processing, characterization, or property image from this parameter space. For example, Figure 4C shows the SEM, EDX, XRD, and PLE captions of figures from abstracts that contain F and Cl on the topic of bioactive glasses. Arbitrarily selected images from this parameter space are displayed for reference in the inset of Figure 4C (i–viii). The captions of these images confirm that the figures do correspond to the selected image type, while their abstracts span a broad topic range include appetites, glass microparticles, mesospheres, and bioactive scaffolds—all of which relate broadly to the subset of bioactive glasses under consideration. This, in turn, answers the fifth question: yes indeed, there are photoluminescence studies of bioactive glasses that contain fluorine. Thus, this method allows the rapid exploration of scientific data to access extremely nuanced and specific information sets. Such a method might be very useful for a researcher who wishes to access the microstructure of chloride or fluoride glasses without conducting an extensive literature survey—the only alternative available today. Elemental tagging adds a chemical marker to images for computer vision tasks, as in the training of predictive algorithms that link microstructure or property to composition.

The methods of systematic scientific exploration of literature that have been developed so far can be generalized by querying the abstract database for arbitrary search terms. Figure 5 presents an example where the application strings "optical glass" and "LED," as well as the synthesis string "solid state" has been used to identify all the abstracts in the document space that contain the respective strings. This allows us to categorize

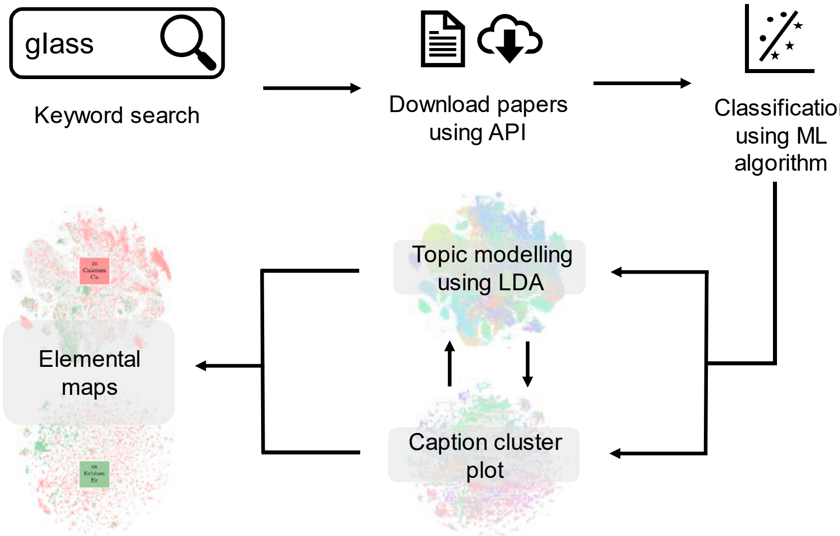

Figure 6. Knowledge extraction pipeline summary
First, we search the CrossRef database for a search query, followed by downloading the papers from Elsevier Science Direct database. To obtain relevant research papers, a supervised classification algorithm is used. After obtaining the articles, we do topic modeling and make CCPs followed by elemental maps.

the abstracts based on specific applications, synthesis methods, characterizations, or properties. The Figure 5A shows the LDA plot. In Figure 5B, all the abstracts relating to optical glasses are identified from corpus and are visually represented as green pixels in the LDA plot, where they are seen to overlap with the topics identified as "LEDs and Phosphors," "spectroscopic studies," and "rare earth glasses." The solution for the final question in the introduction is seen in Figure 5C, which shows all the abstracts that contain the search string "solid state synthesis," which is a common method for fabricating oxides and oxide glasses. The overlap of the two sets of complementary information are the set of abstracts on optical glasses synthesized through solid state synthesis. This analysis can be carried further by combining these data with the CCP, through which specific image types such as the DTA or XRD of optical glasses made through solid state synthesis can be extracted. While the level of specificity offered by this approach is in itself useful to a researcher who wishes to learn more about the solid state synthesis of optical glasses, the method allows the use of any number of search strings—allowing for a detailed multidimensional extraction of information from literature.

Similarly, the green points in Figure 5D represent abstracts with the string "LED" in it. By mapping these data to the elemental maps, the presence of abstracts with specific chemical compositions can be marked, such as in Figure 5E, which highlights the elements europium, cerium, and samarium. These are therefore LEDs that contain any of these elements or any combination of them; this, in turn, answers the fourth question raised in the introduction. This data allow the user to read through these abstracts, and only these abstracts, to learn more about the subject. The user is then able to look through microstructural, luminescence, or thermodynamic data that are linked to this dataset through the CCP.

Altogether, we demonstrate that the application of NLP to glass literature enables the curation and selection of data sources sorted by specific applications, properties, characterization methods, and chemistries. In turn, this allows for custom composition–processing–property databases to be compiled automatically, and in linking together parts of the information space in a way that has so far not been possible.

When used together, text vectorization, LDA, and elemental maps solve many of the challenging questions impeding the accelerated discovery of glasses, some of which are stated in the introduction. We demonstrate this in the paper through six questions that were raised in the introduction and subsequently solved in the results and discussion sections. NLP tools combined with curated glass databases accurately show that most of the published work explored in the analysis is experimental rather than theoretical and that XRD, SEM, etc. are the most common characterization methods for glasses. They also provide solutions to highly specific queries, such as identifying papers on optical glasses that are manufactured using solid state sintering, glass compositions that are both bioactive and fluorine-containing, chemicals used in LED glasses, etc. Many of these questions currently have no other means of being answered, as demonstrated by identifying all the glasses that contain americium.

The techniques and tools developed in this article can be easily extended to other topics. We illustrate this with two topical examples: metallic glasses and magnesium alloys (see Figures S4 and S5). The relevant literature for each topic is identified and the NLP pipeline is applied to extract the CCP, LDA plots, and elemental maps for both topics. The CCP for magnesium alloys show that "dissolution" is a major topic of study in the field, underscoring the interest of the scientific community in assessing the chemical stability of these alloys. Similarly, the elemental maps for metallic glasses provide a quantities measure of the importance of various metals in glass forming. By comparing the maps of copper and iron, for example, we are able to see that they are used together in many applications, while iron is preferable over copper for other applications.

However, the major contribution of the present study is that it opens a way to ask deeper and broader questions of topical literature, that can ultimately enrich the field by solving outstanding problems–old and new. Thus, our study establishes the baseline for extracting images from scientific literature related to specific keywords and topics. Further, we have developed a web-based platform making the knowledge accessible to wider community through PyGGi Pictionary as part of the Python for Glass Genomics Package (PyGGi, see: https://pyggi.iitd.ac.in/toolkits/pictionary).

At this point, it is worth mentioning some of the limitations in the present approach.

1. Classification of abstracts as glass and nonglass has a relatively low recall. This leads to the missing of several abstracts belonging to the topic in the final classified set.
2. The number of topics that can be identified using LDA is quite small at present. Further, LDA being an unsupervised approach requires an expert to understand the topic from the keywords and manually label them.
3. The keyword list associated with the CCP is developed by domain experts.

These problems can be tackled by algorithms that could possibly identify a larger number of categories and automatically label them. In particular, the development of a glass-specific ontology, a curated image repository, and an NER tool tailored for this community can go a long way in enhancing the methods demonstrated for the first time in this paper. Further, by combining these tools with a natural language model, such as a "GlassBert," can open the field to many more advances in artificial intelligence and ML by making available rapid, efficient, and tailored composition–processing–property databases. The scalable methods demonstrated in this study are broadly applicable to any scientific field and consequently is of universal relevance in accelerating the scientific enquiry.

EXPERIMENTAL PROCEDURES

Resource availability

Lead contact

Further information and requests related to this study should be directed to and will be fulfilled by the lead contact, N. M. Anoop Krishnan (krishnan@iitd.ac.in).

Materials availability

This study did not generate any new unique materials.

Data and code availability

All the data and codes used in the present work are available at: https://github.com/m3rg-repo/machine_learning_glass/tree/master/Looking_through_glass.

Methods

The workflow for information extraction is summarized in Figure 6. The CrossRef metadata API was used to query existing literature databases using keywords specific to the glass community. These include (1) descriptive application identifiers, such as "chalcogenide glasses," "bioactive glasses," "laser glasses," "optical glasses," etc.; (2) property/processing terms, such as "glass transition temperature," "oxide glasses," "optical luminescence," etc.; and (3) conjugated keywords such as "glass mechanical properties," "glass-dissolution," "glass AND fracture," etc. This query returned an initial list of more than 6 million DOIs out of which the full texts of 600,000 articles were downloaded using the Elsevier Science Direct API. A custom XML parser was written to extract specific sections of the article including the metadata, abstract, images, image captions, and individual sections identified by their headings.

To understand the distribution of topics in the downloaded database, an NLP algorithm, the LDA, was used to identify the number of distinct "topics" in the corpus where a topic is defined as the set of words with the highest probability of occurrence in a document belonging to the topic. The LDA plot for a given sub-topic is presented in the supplemental information (see Figure S3 and Table S5). It is seen that while some topics are relevant to the scientific literature on glass, many are from the intersection of glasses with peripheral topics such as women's health, economy, and environment. In fact, one topic only includes non-English articles identified by the French vowels "un," "es," "le," etc. Note that the present work focuses only on the articles presented in the English language. However, a similar approach can be extended to other language literature as well. The full texts of articles belonging to topics of little relevance to the materials science literature on glasses were removed, along with editorial notes, commentaries, book reviews, retractions, and conference proceedings. The use of LDA is thus demonstrated to greatly aid the curation of topic-specific text databases, a nontrivial effort by any other means.

The remaining database was further refined by the use of a ML classifier model that performed a binary classification of the article abstracts into "relevant" and "nonrelevant." For this, 3,060 randomly selected abstracts were manually tagged as being "glass" and "not-glass." Using the Python SciKit library, a logistic regression classifier model was found to have an accuracy of 86% and a recall of 67% on the test set for classification task. This model was found to outperform other text classification algorithms such as naive Bayes and Random Forests. The details of the model performance are given in the supplemental information (Tables S2–S4). Finally, the binary classifier categorized 94,207 articles as being relevant to the material science study of glasses. This list contained articles published from 1 August 1997 to 10 June 2020. All further natural language processing–driven analyses were done only on this text corpus. The search query and the corresponding DOIs are shared in the GitHub repository: https://github.com/m3rg-repo/machine_learning_glass/tree/master/Looking_through_glass.

Caption cluster plot

The final corpus contains a total of 106,238 figures and their captions. With the help of the Stanford NLTK[56] package, the caption texts were tokenized after removal of punctuations, numerals, and stop words. These tokens, which include words of the English language, chemical symbols, and abbreviations, form the corpus dictionary of size (N) which determines the number of dimensions of the vector space for the caption cluster plot. Each caption is mapped to a unique vector in this vector space by calculating the TFIDF of every word in the caption. TFIDF is a statistical count that reflects the relevance of a word in a document and is a common vectorization technique for text mining and information retrieval. For a term "t" appearing in "d" documents within a collection D:

$$\text{TFIDF} = \textit{tfidf}\ (t,\ d, D) = \textit{tf}\ (t,\ d)\ \times\ \textit{idf}(t, D)$$

where

$$tf(t,\ d) = \begin{cases} 1, & \textit{if t is present in D} \\ 0, & \textit{otherwise} \end{cases}$$

$$idf\ (t, D) = log\frac{N}{|\{d \in D : t \in d\}|}$$

The cosine distance of all the captions from each other is calculated and used as the metric for t-SNE, which projects the vectors into a 2D plane so that vectors with the highest cosine similarities group together. Finally, each caption is assigned a unique label corresponding to the type of image it represents, such as SEM, XRD, TEM, Luminescence, Fracture, etc., through rule-based string search. The color of the pixel in the t-SNE plot is determined by its label. The TFIDF vectorization and the cosine metric ensure that the geometric distance between pixels on the 2D plot correlate with the semantic similarity of captions, and therefore that identical images cluster together.

Latent Dirichlet Allocation

LDA[49] is one of the most commonly used unsupervised topic modeling approaches. LDA classifies the documents present in a corpus into different topics based on the frequency distribution of the words occurring in the document. The specific steps in the LDA algorithm are as follows:

1. Assume there are articles belonging to k topics in the glass corpus.
2. Randomly assign a topic k_i to each word w_i in all the documents.
3. Compute the probabilities of a word w_i belonging to topic k_i in document d_i as $p(k_i|d_i)$ and probability of a document belonging to topic k_i due to the word w_i as $p(w_i|t_i)$.
4. Update the $p(w_i|t_i)$ as $p(t_i|d_i) \times p(w_i|t_i)$.
5. Repeat steps 1 to 4.

The 94,207 abstracts are further categorized using LDA. The optimum number of topics was found by the coherence plot, which was found to converge after 15 topics. After 500 passes, the LDA algorithm identified the topics listed in Figure 1C. Similar to the CCP, each abstract was tokenized, vectorized, and plotted in 2D using t-SNE. The coloring of the pixels is based on the topic number

identified by the LDA. Once again, the pixels are seen to cluster strongly based on color, indicating that abstracts with similar lexical content have been grouped together by the algorithm. All the hyper parameters for t-SNE, TFIDF, and LDA are provided in section 1 of the supplemental information.

Extraction of chemical species

ChemDataExtractor was used to identify and extract the individual chemical species from every abstract. This includes individual chemical elements and compounds identified by their symbols, names, and chemical formulae. A custom Python script is used to extract the individual chemical elements from the most frequent of these compounds.

Elemental maps

If an element X is identified as being present in an abstract, the pixel corresponding to the abstract in the LDA plot is given a different color. This allows the mapping of elemental compositions as identified in the step above to the information content present in the LDA plot. Similarly, the elemental information and captions are correlated by merging the CCP and the LDA plot. A caption pixel is given a different color if the abstract of the text that the caption belongs to contains the element.

SUPPLEMENTAL INFORMATION

Supplemental information can be found online at https://doi.org/10.1016/j.patter.2021.100290.

ACKNOWLEDGMENTS

N.M.A.K. acknowledges the financial support for this research provided by the Department of Science and Technology, India under the INSPIRE faculty scheme (DST/INSPIRE/04/2016/002774) and DST SERB Early Career Award (ECR/2018/002228). The authors thank the IIT Delhi HPC facility for providing the computational and storage resources.

AUTHOR CONTRIBUTIONS

N.M.A.K. and V.V. conceived the work. V.V. and M.Z. carried out the data download, and M.A. managed the computing cluster. V.V., S.S., and M.Z. carried out the data annotation. V.V. performed the model training. V.V. did the analysis of the paper, with contributions from all the authors. V.V. and N.M.A.K. wrote the manuscript with input from all the authors.

DECLARATION OF INTERESTS

The authors declare no competing interests.

Received: January 26, 2021
Revised: May 10, 2021
Accepted: May 24, 2021
Published: June 24, 2021

REFERENCES

1. De Guire, E., Bartolo, L., Brindle, R., Devanathan, R., Dickey, E.C., Fessler, J., French, R.H., Fotheringham, U., Harmer, M., Lara-Curzio, E., et al. (2019). Data-driven glass/ceramic science research: insights from the glass and ceramic and data science/informatics communities. J. Am. Ceram. Soc. *102*, 6385–6406. https://doi.org/10.1111/jace.16677.
2. Rajan, K. (2015). Materials informatics: the materials "gene" and big data. Annu. Rev. Mater. Res. *45*, 153–169. https://doi.org/10.1146/annurev-matsci-070214-021132.
3. Friedman, C., Kra, P., Yu, H., Krauthammer, M., and Rzhetsky, A. (2001). GENIES: a natural-language processing system for the extraction of molecular pathways from journal articles. Bioinformatics *17*, S74–S82. https://doi.org/10.1093/bioinformatics/17.suppl_1.S74.
4. Lee, J., Yoon, W., Kim, S., Kim, D., Kim, S., So, C.H., and Kang, J. (2020). BioBERT: a pre-trained biomedical language representation model for biomedical text mining. Bioinformatics *36*, 1234–1240. https://doi.org/10.1093/bioinformatics/btz682.
5. Yandell, M.D., and Majoros, W.H. (2002). Genomics and natural language processing. Nat. Rev. Genet. *3*, 601–610. https://doi.org/10.1038/nrg861.
6. Kim, E., Huang, K., Kononova, O., Ceder, G., and Olivetti, E. (2019). Distilling a materials synthesis ontology. Matter *1*, 8–12. https://doi.org/10.1016/j.matt.2019.05.011.
7. Tshitoyan, V., Dagdelen, J., Weston, L., Dunn, A., Rong, Z., Kononova, O., Persson, K.A., Ceder, G., and Jain, A. (2019). Unsupervised word embeddings capture latent knowledge from materials science literature. Nature *571*, 95–98. https://doi.org/10.1038/s41586-019-1335-8.
8. Venugopal, V., Bishnoi, S., Singh, S., Zaki, M., Grover, H.S., Bauchy, M., Agarwal, M., and Krishnan, N.M.A. (2021). Artificial intelligence and machine learning in glass science and technology: 21 challenges for the 21(st) century. Int. J. Appl. Glass Sci. https://doi.org/10.1111/ijag.15881.
9. Weston, L., Tshitoyan, V., Dagdelen, J., Kononova, O., Trewartha, A., Persson, K.A., Ceder, G., and Jain, A. (2019). Named entity recognition and normalization applied to large-scale information extraction from the materials science literature. J. Chem. Inf. Model. *59*, 3692–3702. https://doi.org/10.1021/acs.jcim.9b00470.
10. Kim, E., Jensen, Z., van Grootel, A., Huang, K., Staib, M., Mysore, S., Chang, H.S., Strubell, E., McCallum, A., Jegelka, S., and Olivetti, E. (2020). Inorganic materials synthesis planning with literature-trained neural networks. J. Chem. Inf. Model. *60*, 1194–1201. https://doi.org/10.1021/acs.jcim.9b00995.
11. Huang, S., and Cole, J.M. (2020). A database of battery materials auto-generated using ChemDataExtractor. Sci. Data *7*, 260. https://doi.org/10.1038/s41597-020-00602-2.
12. Beard, E.J., and Cole, J.M. (2020). ChemSchematicResolver: a toolkit to decode 2D chemical diagrams with labels and R-groups into annotated chemical named entities. J. Chem. Inf. Model. *60*, 2059–2072. https://doi.org/10.1021/acs.jcim.0c00042.
13. Cole, J.M. (2020). A design-to-device pipeline for data-driven materials discovery. Acc. Chem. Res. *53*, 599–610. https://doi.org/10.1021/acs.accounts.9b00470.
14. Court, C.J., Yildirim, B., Jain, A., and Cole, J.M. (2020). 3-D inorganic crystal structure generation and property prediction via representation learning. J. Chem. Inf. Model. *60*, 4518–4535. https://doi.org/10.1021/acs.jcim.0c00464.
15. Court, C.J., and Cole, J.M. (2018). Auto-generated materials database of Curie and Néel temperatures via semi-supervised relationship extraction. Sci. Data *5*, 180111. https://doi.org/10.1038/sdata.2018.111.
16. Swain, M.C., and Cole, J.M. (2016). ChemDataExtractor: a toolkit for automated extraction of chemical information from the scientific literature. J. Chem. Inf. Model. *56*, 1894–1904. https://doi.org/10.1021/acs.jcim.6b00207.
17. Court, C.J., and Cole, J.M. (2020). Magnetic and superconducting phase diagrams and transition temperatures predicted using text mining and machine learning. Npj Comput. Mater. *6*, 18. https://doi.org/10.1038/s41524-020-0287-8.
18. Mahbub, R., Huang, K., Jensen, Z., Hood, Z.D., Rupp, J.L.M., and Olivetti, E.A. (2020). Text mining for processing conditions of solid-state battery electrolyte. Electrochem. Commun. *121*, 106860. https://doi.org/10.1016/j.elecom.2020.106860.
19. Kim, E., Huang, K., Jegelka, S., and Olivetti, E. (2017). Virtual screening of inorganic materials synthesis parameters with deep learning. NPJ Comput. Mater. *3*, 53. https://doi.org/10.1038/s41524-017-0055-6.
20. Kim, E., Huang, K., Tomala, A., Matthews, S., Strubell, E., Saunders, A., McCallum, A., and Olivetti, E. (2017). Machine-learned and codified synthesis parameters of oxide materials. Sci. Data *4*, 170127. https://doi.org/10.1038/sdata.2017.127.
21. Jensen, Z., Kim, E., Kwon, S., Gani, T.Z.H., Roman-Leshkov, Y., Moliner, M., Corma, A., and Olivetti, E. (2019). A machine learning approach to

zeolite synthesis enabled by automatic literature data extraction. ACS Cent. Sci. *5*, 892–899. https://doi.org/10.1021/acscentsci.9b00193.

22. Traynor, B., Uvegi, H., Olivetti, E., Lothenbach, B., and Myers, R.J. (2020). Methodology for pH measurement in high alkali cementitious systems. Cem. Concr. Res. *135*, 106122. https://doi.org/10.1016/j.cemconres.2020.106122.
23. Mukaddem, K.T., Beard, E.J., Yildirim, B., and Cole, J.M. (2020). ImageDataExtractor: a tool to extract and quantify data from microscopy images. J. Chem. Inf. Model. *60*, 2492–2509. https://doi.org/10.1021/acs.jcim.9b00734.
24. Tatum, W.K., Torrejon, D., O'Neil, P., Onorato, J.W., Resing, A.B., Holliday, S., Flagg, L.Q., Ginger, D.S., and Luscombe, C.K. (2020). Generalizable framework for algorithmic interpretation of thin film morphologies in scanning probe images. J. Chem. Inf. Model. *60*, 3387–3397. https://doi.org/10.1021/acs.jcim.0c00308.
25. Venugopal, V., Broderick, S.R., and Rajan, K. (2019). A picture is worth a thousand words: applying natural language processing tools for creating a quantum materials database map. MRS Commun. *9*, 1134–1141. https://doi.org/10.1557/mrc.2019.136.
26. Bhaskar, P., Kumar, R., Maurya, Y., Ravinder, R., Allu, A.R., Das, S., Gosvami, N.N., Youngman, R.E., Bødker, M.S., Mascaraque, N., et al. (2020). Cooling rate effects on the structure of 45S5 bioglass: insights from experiments and simulations. J. Non-cryst. Sol. *534*, 119952. https://doi.org/10.1016/j.jnoncrysol.2020.119952.
27. Varsheneya, A., and Mauro, J.C. (2019). Fundamentals of Inorganic Glasses, Third Edition (Elsevier).
28. Alcobaça, E., Mastelini, S.M., Botari, T., Pimentel, B.A., Cassar, D.R., de Carvalho, A.C.P., and Zanotto, E.D. (2020). Explainable machine learning algorithms for predicting glass transition temperatures. Acta Mater. *188*, 92–100. https://doi.org/10.1016/j.actamat.2020.01.047.
29. Anoop Krishnan, N.M., Mangalathu, S., Smedskjaer, M.M., Tandia, A., Burton, H., and Bauchy, M. (2018). Predicting the dissolution kinetics of silicate glasses using machine learning. J. Non-cryst. Sol. *487*, 37–45. https://doi.org/10.1016/j.jnoncrysol.2018.02.023.
30. Bishnoi, S., Ravinder, R., Grover, H.S., Kodamana, H., and Krishnan, N.M.A. (2021). Scalable Gaussian processes for predicting the optical, physical, thermal, and mechanical properties of inorganic glasses with large datasets. Mater. Adv. https://doi.org/10.1039/D0MA00764A.
31. Cassar, D.R., de Carvalho, A.C.P., and Zanotto, E.D. (2018). Predicting glass transition temperatures using neural networks. Acta Mater. *159*, 249–256. https://doi.org/10.1016/j.actamat.2018.08.022.
32. Covarrubias, C., Agüero, A., Maureira, M., Morelli, E., Escobar, G., Cuadra, F., Peñafiel, C., and Von Marttens, A. (2019). In situ preparation and osteogenic properties of bionanocomposite scaffolds based on aliphatic polyurethane and bioactive glass nanoparticles. Mater. Sci. Eng. C *96*, 642–653. https://doi.org/10.1016/j.msec.2018.11.085.
33. Han, T., Stone-Weiss, N., Huang, J., Goel, A., and Kumar, A. (2020). Machine learning as a tool to design glasses with controlled dissolution for healthcare applications. Acta Biomater. *107*, 286–298. https://doi.org/10.1016/j.actbio.2020.02.037.
34. Mauro, J.C., Tandia, A., Vargheese, K.D., Mauro, Y.Z., and Smedskjaer, M.M. (2016). Accelerating the design of functional glasses through modeling. Chem. Mater. *28*, 4267–4277. https://doi.org/10.1021/acs.chemmater.6b01054.
35. Montazerian, M., Zanotto, E.D., and Mauro, J.C. (2020). Model-driven design of bioactive glasses: from molecular dynamics through machine learning. Int. Mater. Rev. *65*, 297–321. https://doi.org/10.1080/09506608.2019.1694779.
36. Ravinder, R., Sridhara, K.H., Bishnoi, S., Grover, H.S., Bauchy, M., Jayadeva, Kodamana, H., and Krishnan, N.M.A. (2020). Deep learning aided rational design of oxide glasses. Mater. Horiz. *7*, 1819–1827. https://doi.org/10.1039/D0MH00162G.
37. Tandia, A., Onbasli, M.C., and Mauro, J.C. (2019). Machine learning for glass modeling. In Springer Handbook of Glass, J.D.M.J.H.L. Calvez, ed. (Springer), pp. 1157–1192.
38. Yang, K., Xu, X., Yang, B., Cook, B., Ramos, H., Krishnan, N.M.A., Smedskjaer, M.M., Hoover, C., and Bauchy, M. (2019). Predicting the young's modulus of silicate glasses using high-throughput molecular dynamics simulations and machine learning. Sci. Rep. *9*, 8739. https://doi.org/10.1038/s41598-019-45344-3.
39. Nabian, N., Jahanshahi, M., and Rabiee, S.M. (2011). Synthesis of nano-bioactive glass–ceramic powders and its in vitro bioactivity study in bovine serum albumin protein. J. Mol. Struct. *998*, 37–41. https://doi.org/10.1016/j.molstruc.2011.05.002.
40. Ravinder, R. (2020). Python for Glass Genomics (PyGGi). https://pyggi.iitd.ac.in.
41. Priven, A.I., and Mazurin, O.V. (2008). Glass property databases: their history, present state, and prospects for further development. Adv. Mat. Res. *39-40*, 147–152. 10.4028/www.scientific.net/AMR.39-40.147.
42. Anderson, P.W. (1995). Through the glass lightly. Science *267*, 1609–1618. https://doi.org/10.1126/science.267.5204.1615-e.
43. Kasimuthumaniyan, S., Reddy, A.A., Krishnan, N.M.A., and Gosvami, N.N. (2020). Understanding the role of post-indentation recovery on the hardness of glasses: case of silica, borate, and borosilicate glasses. J. Non-cryst. Sol. *534*, 119955. https://doi.org/10.1016/j.jnoncrysol.2020.119955.
44. Li, X., Song, W., Yang, K., Krishnan, N.M.A., Wang, B., Smedskjaer, M.M., Mauro, J.C., Sant, G., Balonis, M., and Bauchy, M. (2017). Cooling rate effects in sodium silicate glasses: bridging the gap between molecular dynamics simulations and experiments. J. Chem. Phys. *147*, 074501. https://doi.org/10.1063/1.4998611.
45. Metadata retrieval - Crossref. (2020). https://www.crossref.org/education/retrieve-metadata/.
46. Elsevier Developer Portal. (2020). https://dev.elsevier.com/.
47. Mauro, J.C., Philip, C.S., Vaughn, D.J., and Pambianchi, M.S. (2014). Glass science in the United States: current status and future directions. Int. J. Appl. Glass Sci. *5*, 2–15. https://doi.org/10.1111/ijag.12058.
48. Mauro, J.C., and Zanotto, E.D. (2014). Two centuries of glass research: historical trends, current status, and grand challenges for the future. Int. J. Appl. Glass Sci. *5*, 313–327. https://doi.org/10.1111/ijag.12087.
49. Blei, D.M., Ng, A.Y., and Jordan, M.I. (2003). Latent dirichlet allocation. J. Mach. Learn. Res. *3*, 993–1022. https://doi.org/10.5555/944919.944937.
50. Jones, K.S. (1972). A Statistical Interpretation of Term Specificity and its Application in Retrieval (MCB UP Ltd). https://doi.org/10.1108/eb026526.
51. Araújo, M., Miola, M., Bertone, E., Baldi, G., Perez, J., and Verné, E. (2015). On the mechanism of apatite-induced precipitation on 45S5 glass pellets coated with a natural-derived polymer. Appl. Surf. Sci. *353*, 137–149. https://doi.org/10.1016/j.apsusc.2015.06.088.
52. Ibrahim, N.F., Mohamad, H., Mohd Noor, S.N.F., and Ahmad, N. (2017). Apatite formation on melt-derived bioactive glass powder based on SiO2-CaO-Na2O-P2O5 system. Ceram. Int. *43*, 11676–11685. https://doi.org/10.1016/j.ceramint.2017.05.356.
53. Miao, G., Li, Z., Meng, Y., Wu, J., Li, Y., Hu, Q., Chen, X., Yang, X., and Chen, X. (2019). Preparation, characterization, in vitro bioactivity and protein loading/release property of mesoporous bioactive glass microspheres with different compositions. Adv. Powder Technol. *30*, 1848–1857. https://doi.org/10.1016/j.apt.2019.06.002.
54. Yao, F., LeGeros, J.P., and LeGeros, R.Z. (2009). Simultaneous incorporation of carbonate and fluoride in synthetic apatites: effect on crystallographic and physico-chemical properties. Acta Biomater. *5*, 2169–2177. https://doi.org/10.1016/j.actbio.2009.02.007.
55. Zhou, H., Nabiyouni, M., Lin, B., and Bhaduri, S.B. (2013). Fabrication of novel poly(lactic acid)/amorphous magnesium phosphate bionanocomposite fibers for tissue engineering applications via electrospinning. Mater. Sci. Eng. C *33*, 2302–2310. https://doi.org/10.1016/j.msec.2013.01.058.
56. Bird, S., Klein, E., and Loper, E. (2009). Natural Language Processing with Python: Analyzing Text with the Natural Language Toolkit (O'Reilly Media, Inc.).

Patterns, Volume *2*

Supplemental information

Looking through glass: Knowledge discovery

from materials science literature

using natural language processing

Vineeth Venugopal, Sourav Sahoo, Mohd Zaki, Manish Agarwal, Nitya Nand Gosvami, and N. M. Anoop Krishnan

Supplemental Items

Figure S1. Cluster plot of 50000 abstracts with 15 topics. From the initial corpus of 600000 abstracts, 50000 were randomly selected and put through an LDA algorithm which identified 15 topics as being the most predominant among them. The optimum number of topics were identified using the coherence plot shown below – which plateaus at 15. The abstracts were clustered and labelled according to their LDA topic number.

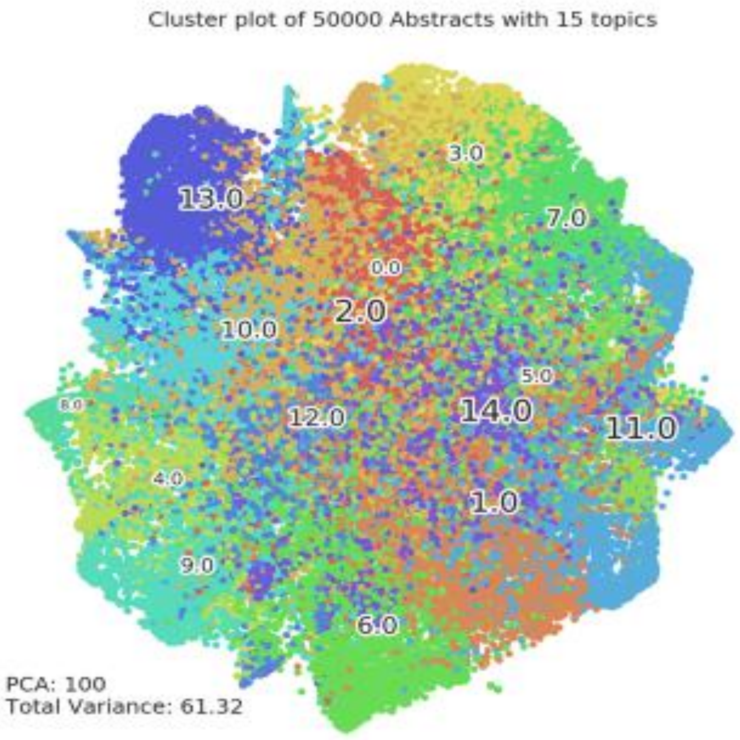

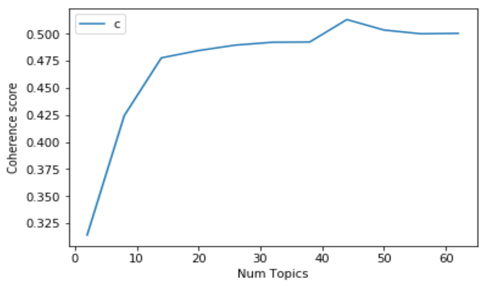

Table S1. Keywords corresponding to the topic number. The word distribution of the 15 topics showing the top ten words with the highest probability in each topic. It is seen that many topics contain words that are not directly relevant to the material science literature on oxide glasses. These abstracts were removed from the database and further classified using supervised learning.

Topic No	Keywords
0	['cells', 'protein', 'cell', 'activity', 'binding', 'structure', 'proteins', 'dna', 'human', 'enzyme']
1	['water', 'properties', 'concentration', 'ph', 'content', 'showed', 'results', 'temperature', 'polymer', 'different']
2	['glass', 'composite', 'fiber', 'fibers', 'properties', 'mechanical', 'materials', 'material', 'strength', 'results']
3	['based', 'use', 'new', 'paper', 'structure', 'methods', 'study', 'research', 'data', 'systems']
4	['temperature', 'phase', 'glass', 'transition', 'magnetic', 'fe', 'structure', 'properties', 'dielectric', 'samples']
5	['soil', 'study', 'patients', 'treatment', 'species', 'group', 'results', 'fluoride', 'significantly', 'rare']
6	['particles', 'size', 'particle', 'nanoparticles', 'ag', 'powder', 'surface', 'nm', 'silver', 'diameter']
7	['mantle', 'ma', 'pb', 'high', 'sr', 'rocks', 'ree', 'ratios', 'samples', 'composition']
8	['model', 'results', 'data', 'experimental', 'flow', 'method', 'structure', 'parameters', 'time', 'transition']
9	['la', 'ce', 'et', 'da', 'ra', 'des', 'en', 'ta', 'sa', 'une']
10	['surface', 'reaction', 'adsorption', 'high', 'fe', 'tio', 'carbon', 'oxidation', 'metal', 'prepared']
11	['water', 'data', 'area', 'zone', 'high', 'basin', 'region', 'formation', 'sediments', 'changes']
12	['films', 'optical', 'nm', 'film', 'emission', 'absorption', 'properties', 'glass', 'doped', 'energy']
13	['properties', 'mechanical', 'composites', 'strength', 'al', 'high', 'microstructure', 'temperature', 'tensile', 'alloy']
14	['structure', 'complexes', 'ii', 'compounds', 'complex', 'crystal', 'molecular', 'spectra', 'metal', 'structures']

Three thousand and sixty randomly selected abstracts were manually labelled as 'glass' and 'not glass' from among these articles. They were then subjected to 5-fold cross validation. Three machine learning models – logistic regression, naïve bayes, and random forest were trained on this data for classification. The results of the models on the training set are shown below.

Table S2. Model Performance on Training Set.

Method	Precision, %	Accuracy, %	Recall, %	Confusion Matrix
Logistic Regression	93	88	73	[1205, 40<br>195, 518]
Naïve Bayes	98	87	67	[1220, 8<br>238, 492]
Random Forest	100	100	100	[1253, 0<br>0, 705]

Naïve Bayes and Random Forest were found to perform the best in terms of precision and accuracy. However, logistic regression was found to give higher recall than random forest and also performed well on the test and validation set as shown below.

Table S3. Model Performance on Validation Set.

Method	Precision, %	Accuracy, %	Recall, %	Confusion Matrix
Logistic Regression	93	86	69	[295, 9<br>58, 128]
Naïve Bayes	99	88	66	[320, 1<br>58, 111]
Random Forest	97	84	63	[292, 4<br>72, 122]

Table S4. Model Performance on Test Set.

Method	Precision, %	Accuracy, %	Recall, %	Confusion Matrix
Logistic Regression	92	86	67	[374, 13<br>74, 151]
Naïve Bayes	98	83	55	[384, 3<br>102, 123]
Random Forest	95	84	60	[380, 7<br>91, 134]

We note that the precision of the model is very high, which suggests that number of false positives (“non-glass” classified as “glass”) is minimal. However, we note that the recall on the validation set is relatively low for all the models, which suggests that the false negatives (“glass” classified as “non-glass”) is very high. Low recall, thus, leads to the missing of several papers belonging to the “glass” topic. In our case, thus, the model with the highest recall on validation set was given preference to include as many glass-related papers as possible. This does mean that some non-glass articles are included in the final corpus but their effect on the final results was found to be minimal.

Figure S2. The distribution of chemicals in the database as identified by ChemDataExtractor.

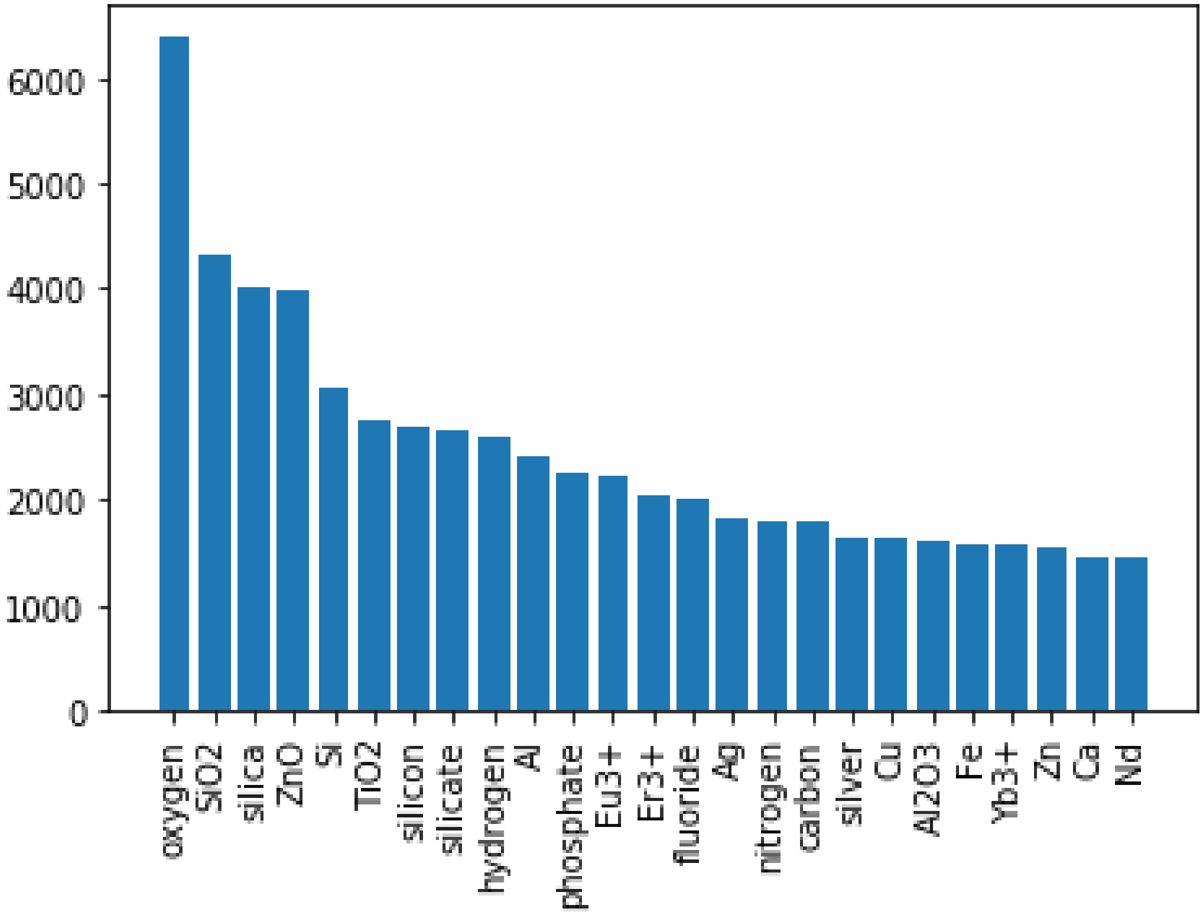

Figure S3. LDA topic modeling for topic number 5. Each topic from the LDA plot can be further subjected to another round of LDA topic classification – as shown here for topic no 5 –thin films on glasses. The optimum number of topics was found to be 7 through the coherence plot. 12866 abstracts are contained in Topic 5, which is clustered separately and labelled in the inset diagram shown alongside.

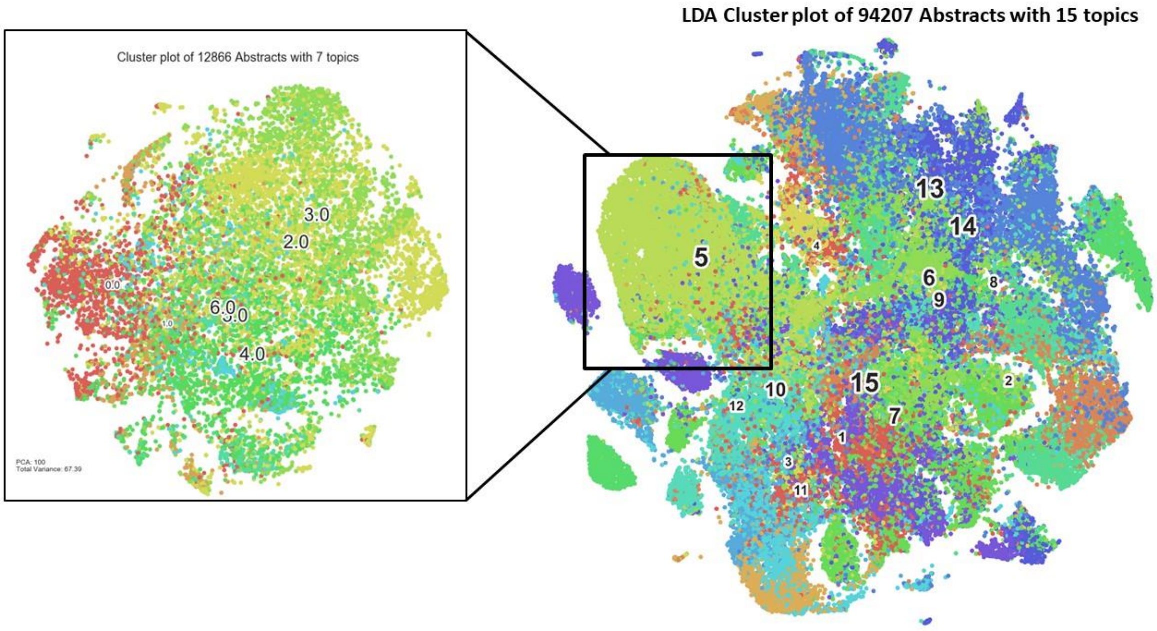

The words with the highest probability distribution in each topic is listed separately in the Table S5 at the bottom.

Table S5. List of keywords associated with each topic.

Topic No	Keywords
0	['glass', 'layer', 'tio', 'film', 'light', 'ito', 'oxide', 'nm', 'solar', 'ag']
1	['films', 'surface', 'glass', 'nm', 'film', 'ions', 'irradiation', 'emission', 'gas', 'luminescence']
2	['films', 'optical', 'band', 'ray', 'gap', 'deposited', 'ev', 'film', 'properties', 'energy']
3	['films', 'zno', 'doped', 'optical', 'properties', 'temperature', 'electrical', 'deposited', 'doping', 'film']
4	['films', 'si', 'deposition', 'deposited', 'glass', 'film', 'silicon', 'substrate', 'sio', 'temperature']
5	['laser', 'growth', 'grown', 'cu', 'temperature', 'pulsed', 'emission', 'substrate', 'ga', 'layer']
6	['films', 'doped', 'dielectric', 'ti', 'film', 'properties', 'mg', 'si', 'fe', 'doping']

Supplemental Experimental Procedures

Section 1. HYPER PARAMETERS for t-SNE, TFIDF and LDA

1. t-SNE
    a. n_components = 2
    b. metric = ‘precomputed’
    c. perplexity = 40
    d. n_iter = 300
    e. random_state = 92
2. TFIDF
    a. min_df = 100
    b. max_df = 0.9
    c. ngram_range = (1,3)
3. LDA
    a. num_topics = 15
    b. passes = 10
    c. chunksize = 1000

Demonstration of NLP algorithms to other topics in Material Science

The techniques developed in the paper can easily be applied to any field of knowledge. We demonstrate this here with two specific applications within material science: magnesium alloys and metallic glasses. Magnesium alloys are a class of metals that contain magnesium as the main alloying agent. They find many uses in structural applications such as in aeronautics. Metallic glasses are alloys of metals that resemble glasses in their atomic structure. They find applications where high strength is desirable, for example in making shaving blades. Both these topics are active areas of research. The NLP tools developed in this paper can greatly assist researchers and other members of the community to selectively query, visualize, and interact with the application space.

Elemental Map for Metallic Glasses

A corpus of around 30,000 articles on Metallic glasses were extracted from Elsevier. The abstracts of these articles were extracted and used to create an LDA plot as explained in the Methodology section. Chemical elements were extracted from these documents and individual markers for each element were created. By superimposing these markers on the LDA plot, the application subspace of each element can be visualized with respect to the total application space of Metallic glasses. Only the maps for copper and Iron are shown here. It is seen that these metals are areas where there are both used and certain applications where one metal is preferred over the other. The extraction and visualization of this information is currently impossible through any other means.

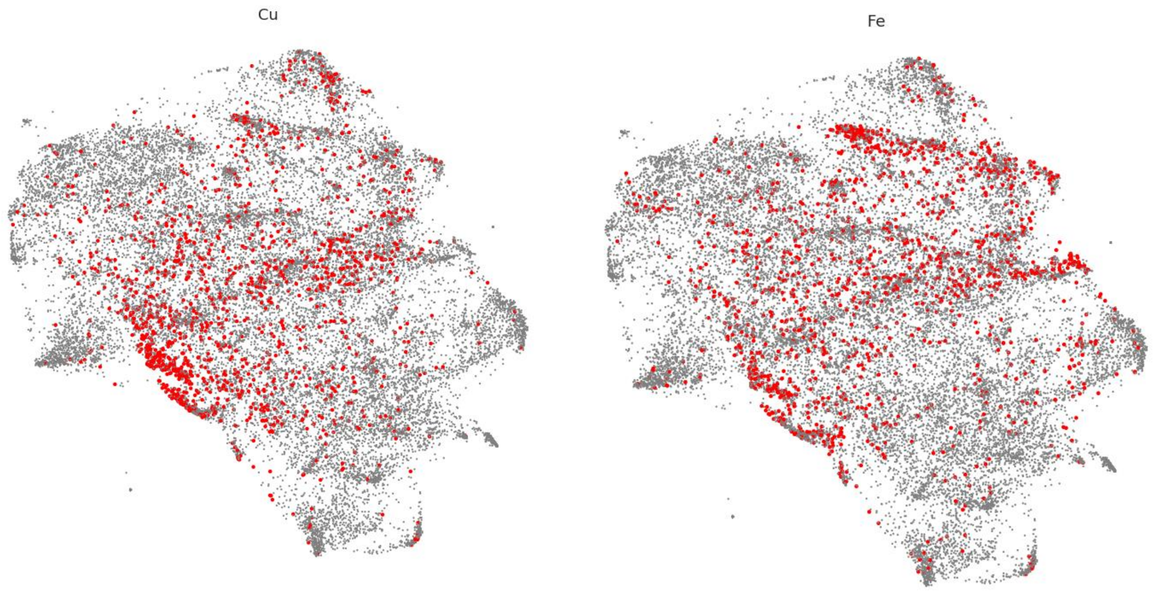

Figure S4. Elemental Map for Metallic Glasses. The Elemental maps for metallic glasses plotted here for copper and Iron. The graphs clearly demonstrate that they share common applications as well as regions where one is preferred over the other.

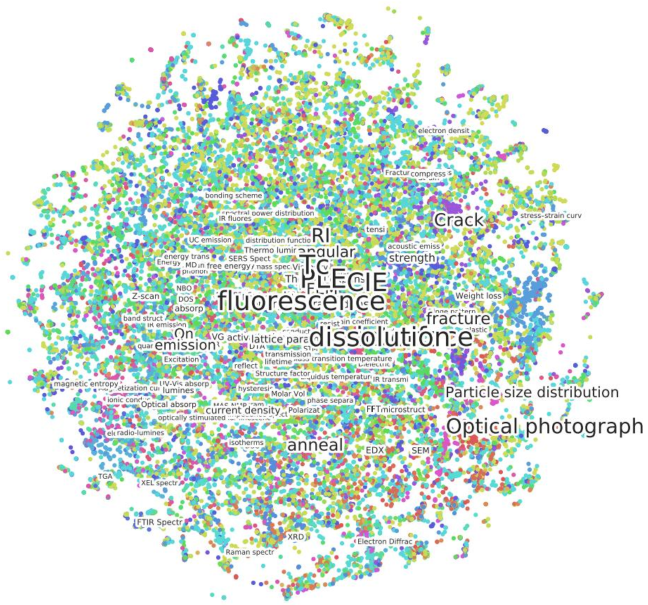

Figure S5. Caption Cluster Plot for Magnesium alloys. A corpus of around 40,000 articles on Magnesium alloys were extracted from Elsevier. The abstracts of these articles were extracted and used to create a CC plot as explained in the Methodology section.

Section 2. Elemental Maps (CCP and LDA plots for elements)

Figure S6. Caption Cluster plot for Hydrogen.

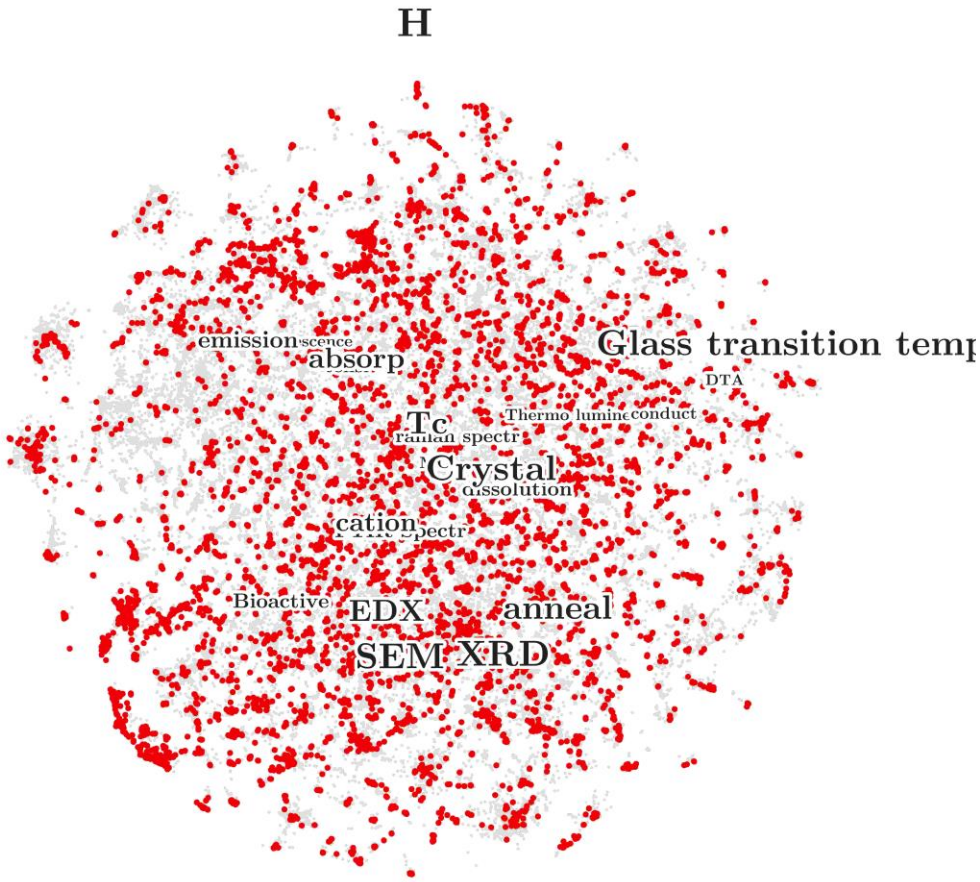

Figure S7. Latent Dirichlet Allocation plot for Hydrogen.

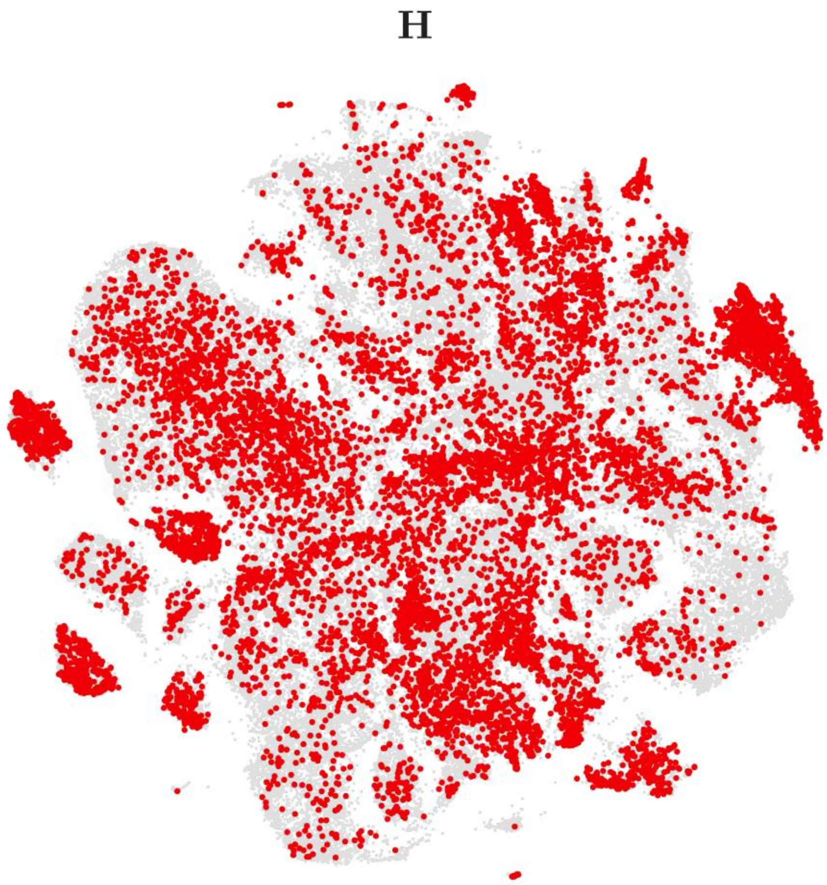

Figure S8. Caption Cluster plot for Helium.

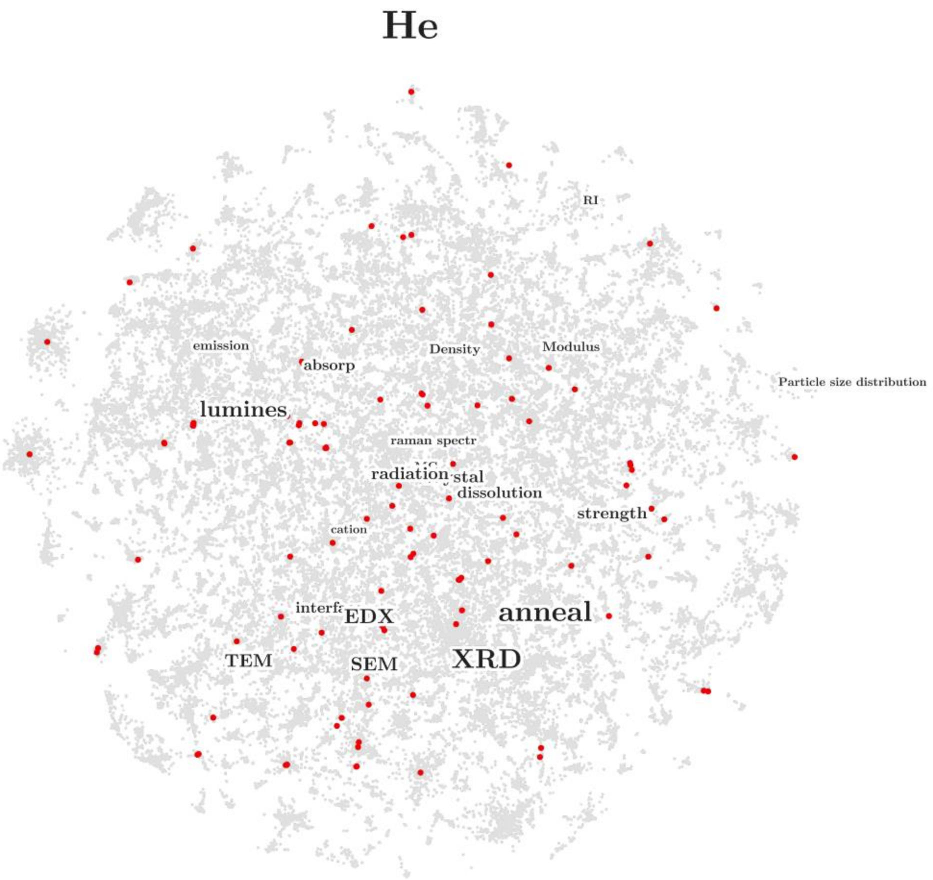

Figure S9. Latent Dirichlet Allocation plot for Helium.

He

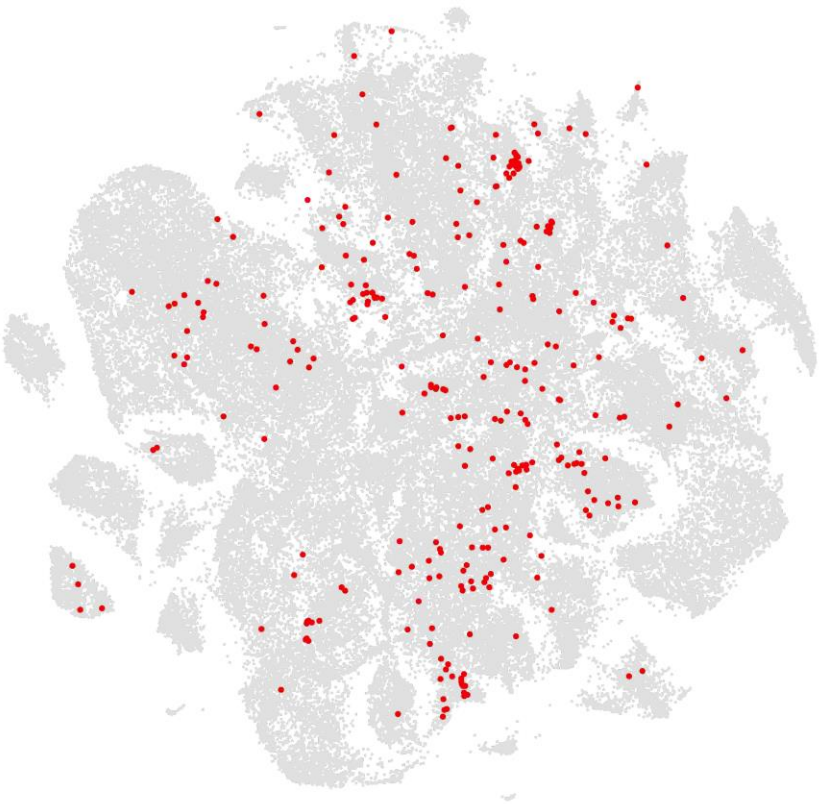

Figure S10. Caption Cluster plot for Lithium.

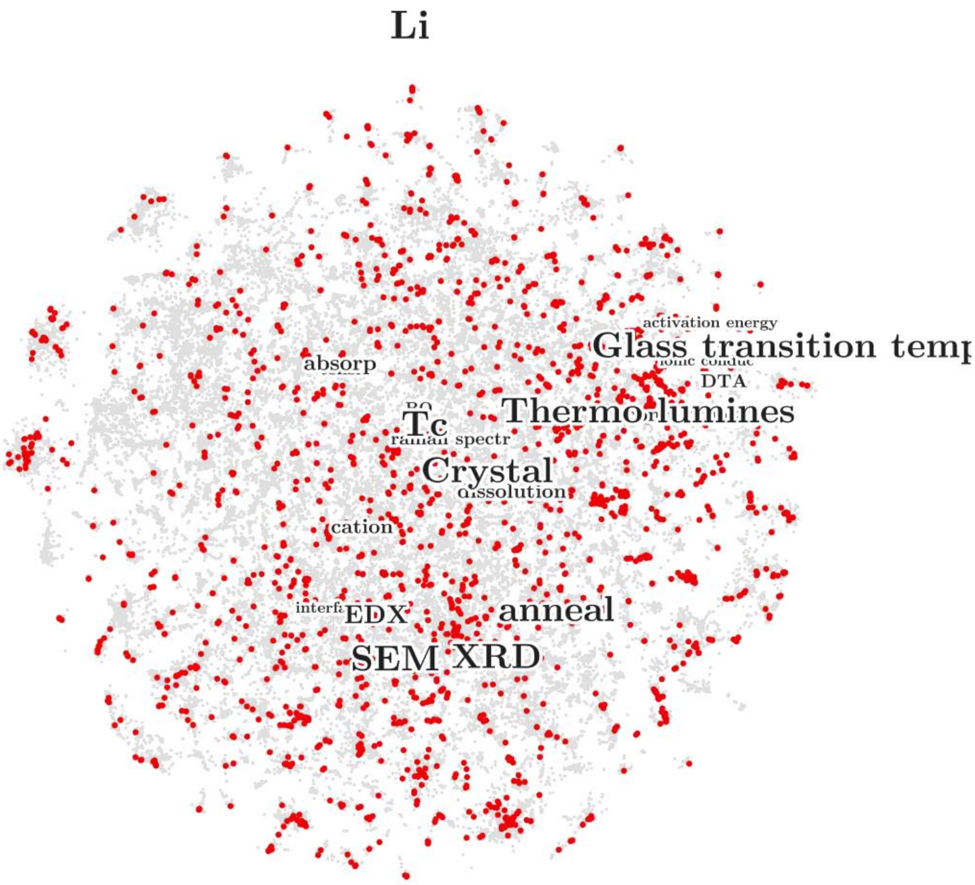

Figure S11. Latent Dirichlet Allocation plot for Lithium.

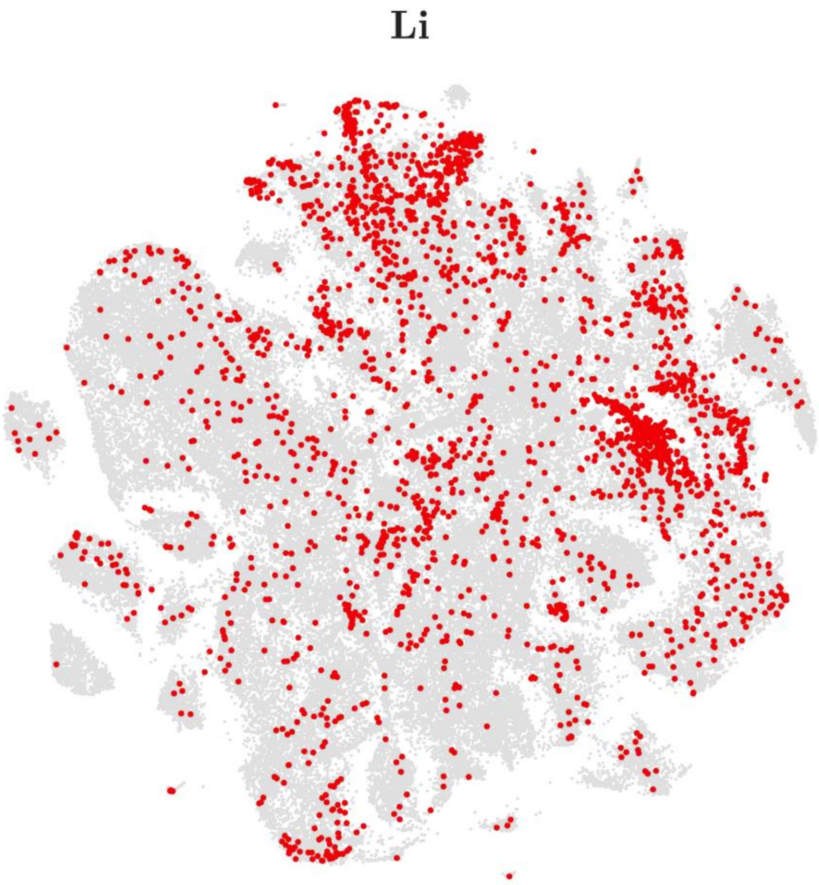

Figure S12. Caption Cluster plot for Beryllium.

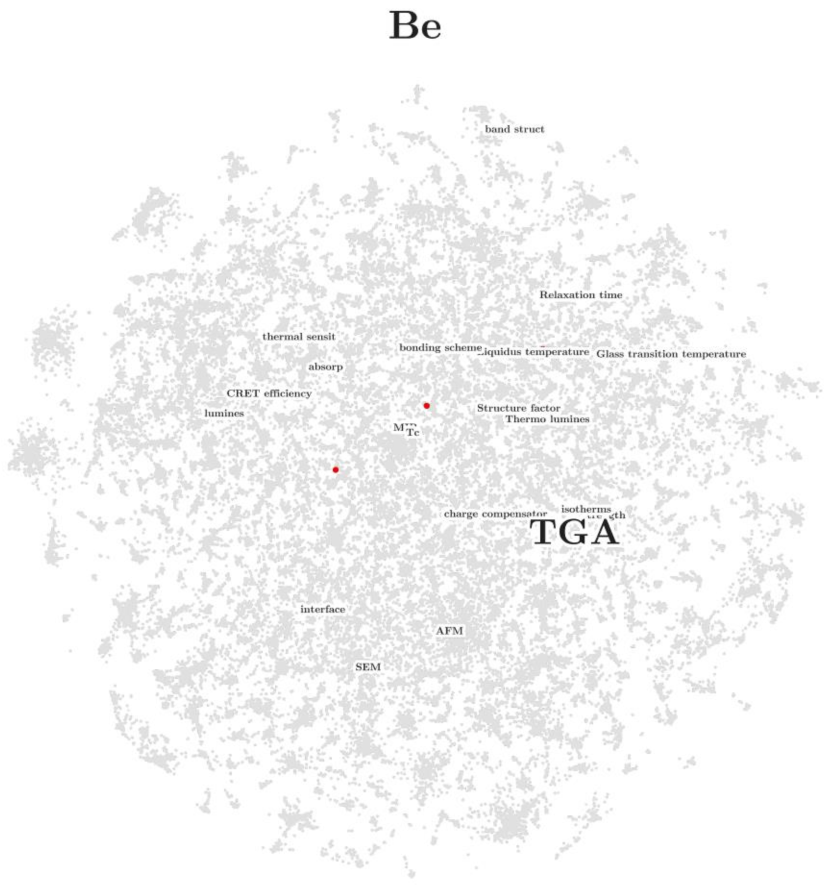

Figure S13. Latent Dirichlet Allocation plot for Beryllium.

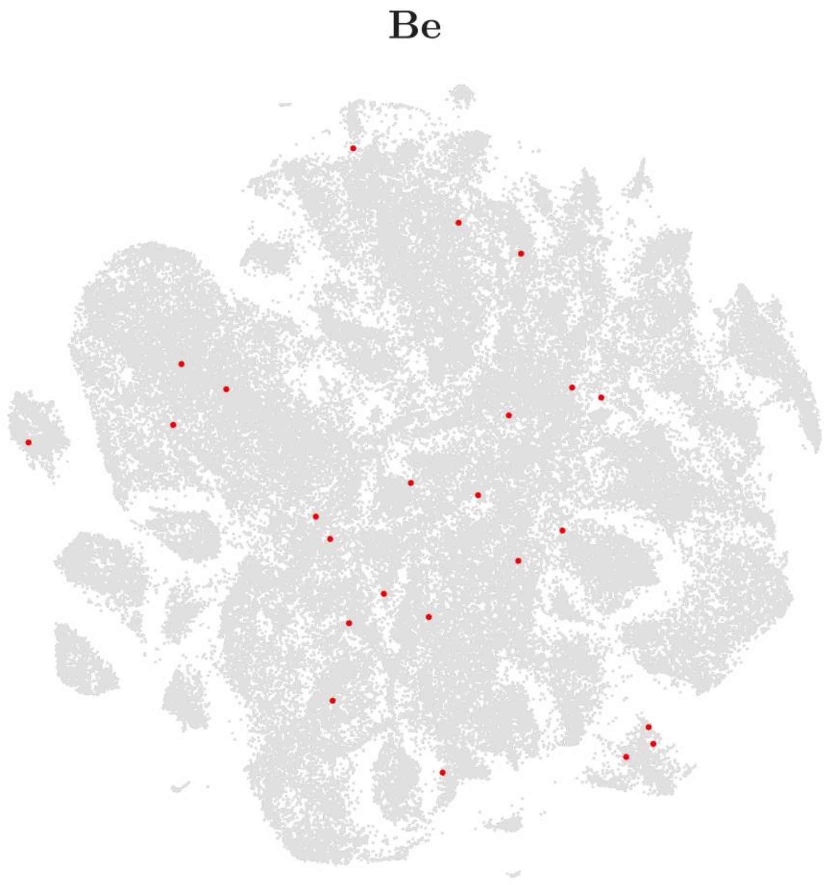

Figure S14. Caption Cluster plot for Boron.

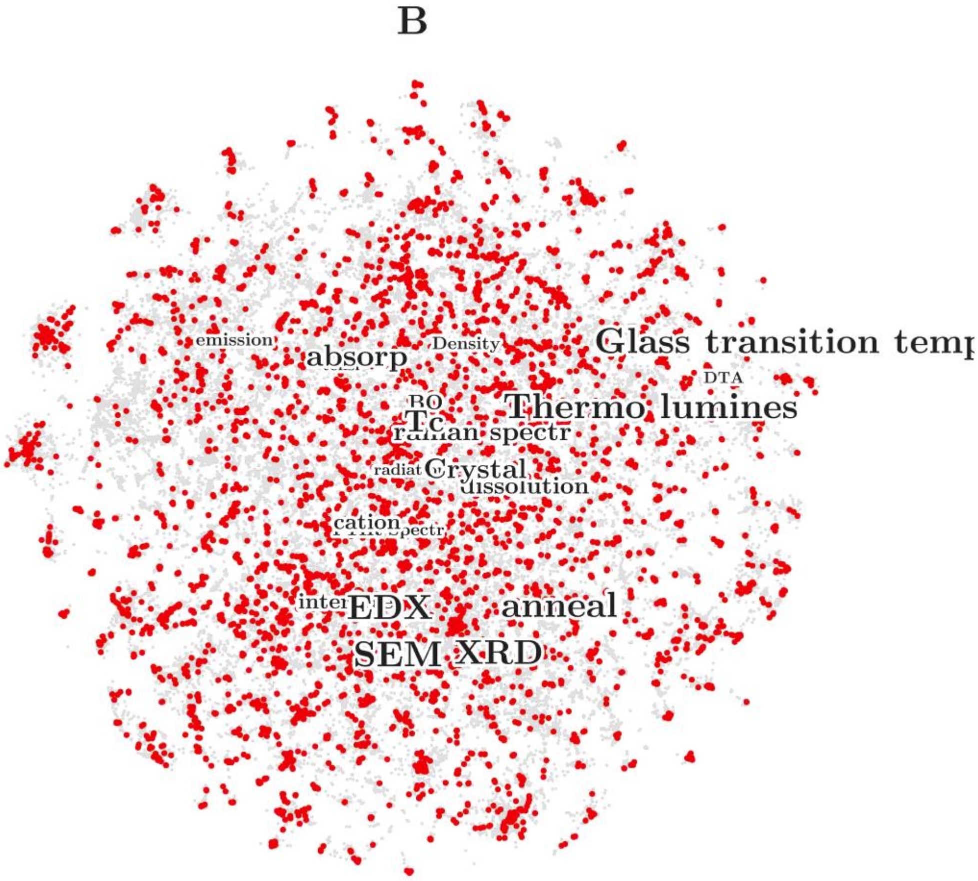

Figure S15. Latent Dirichlet Allocation plot for Boron.

B

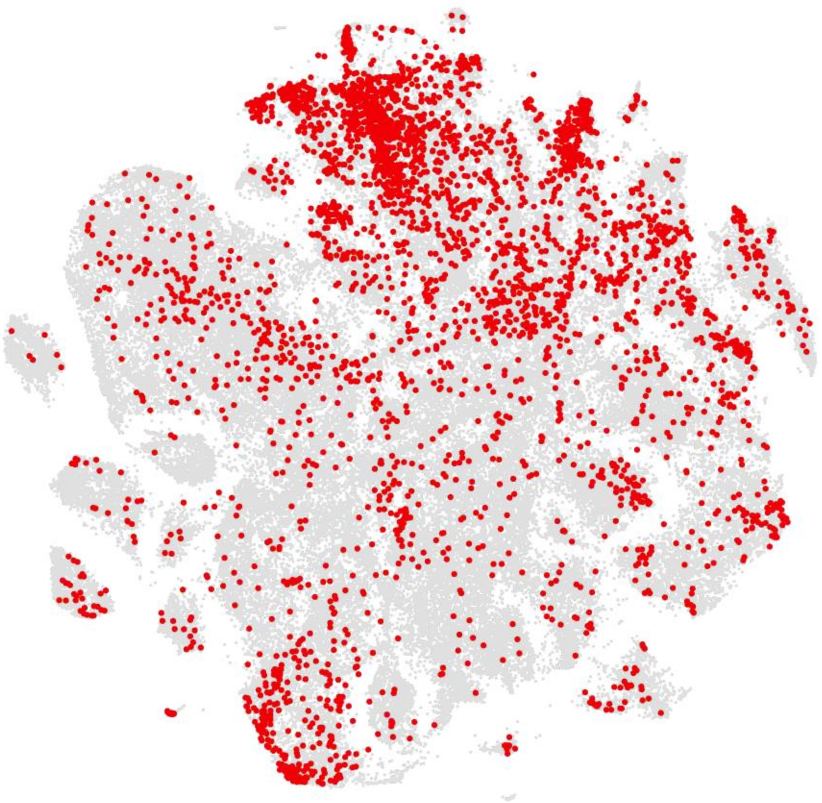

Figure S16. Caption Cluster plot for Carbon.

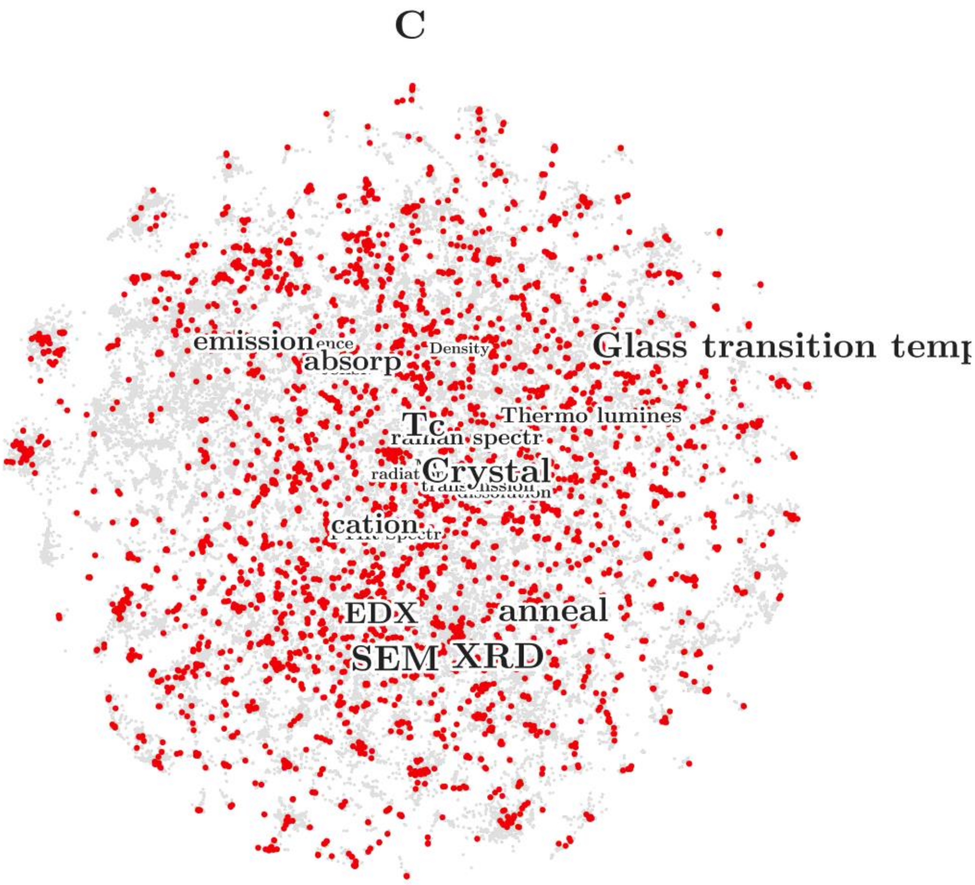

Figure S17. Latent Dirichlet Allocation plot for Carbon.

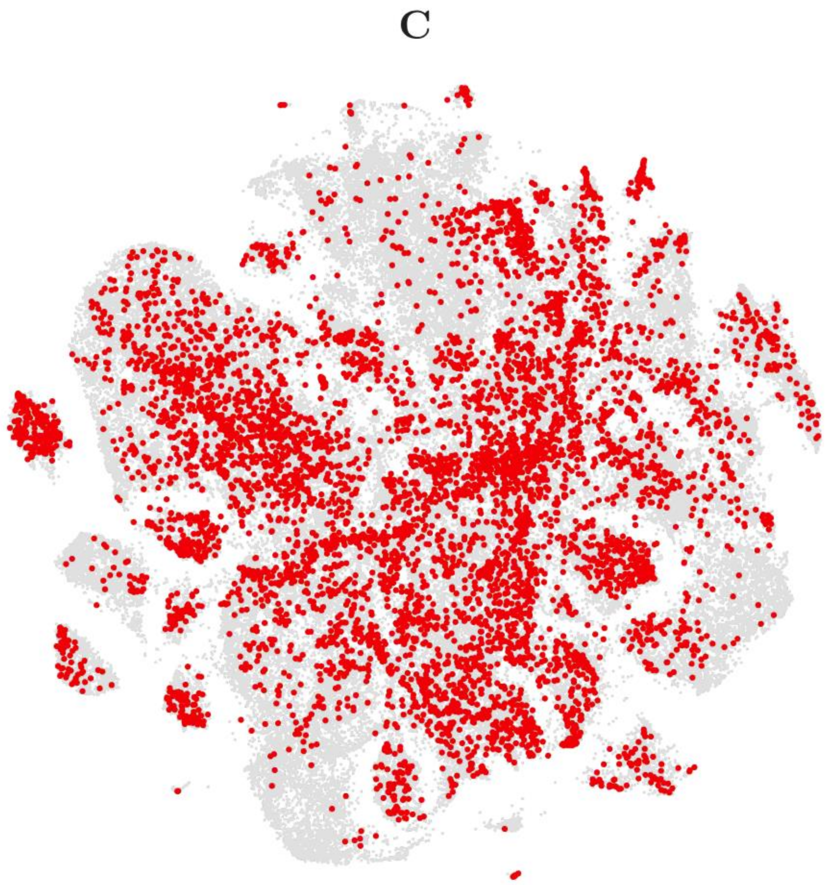

Figure S18. Caption Cluster plot for Nitrogen.

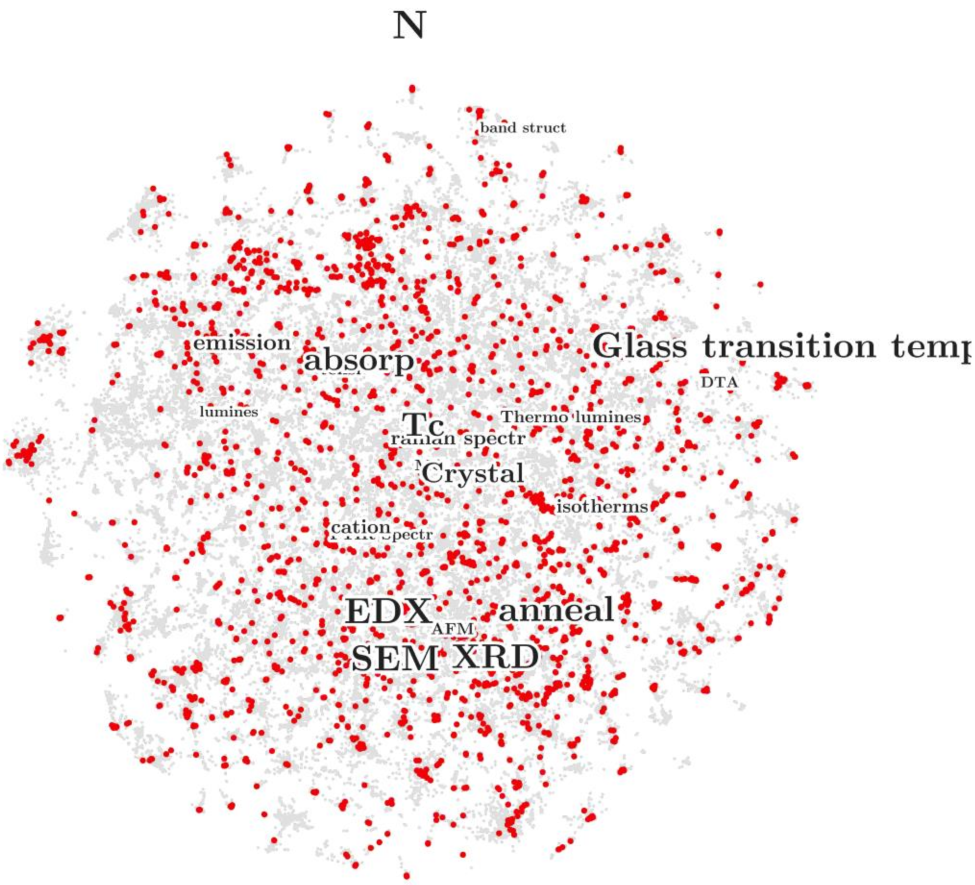

Figure S19. Latent Dirichlet Allocation plot for Nitrogen.

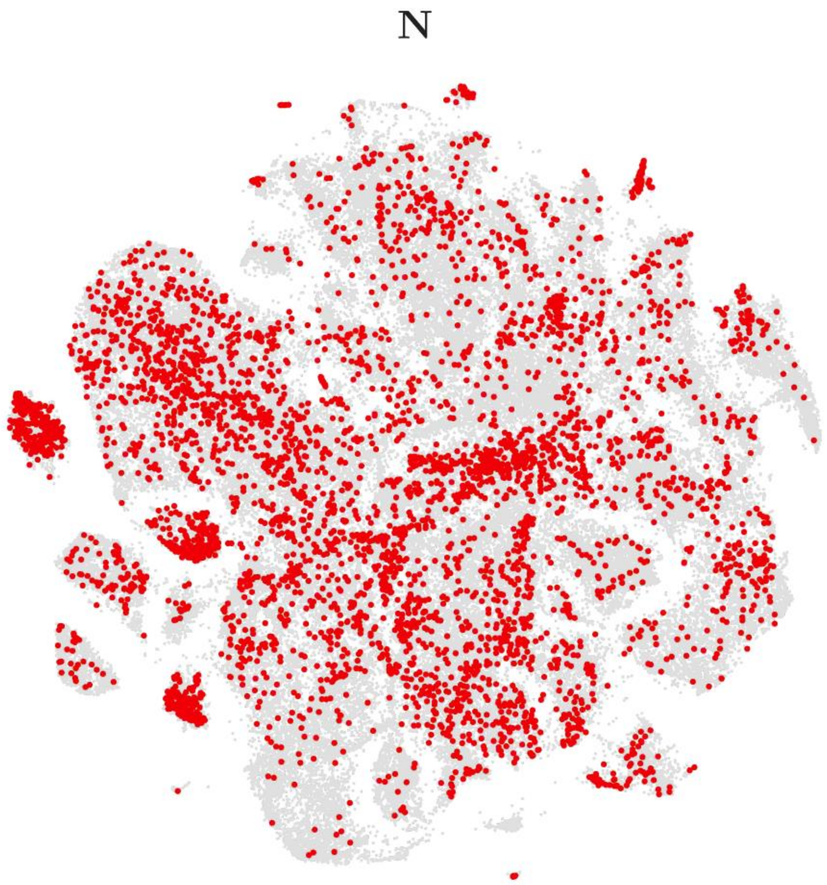

Figure S20. Caption Cluster plot for Oxygen.

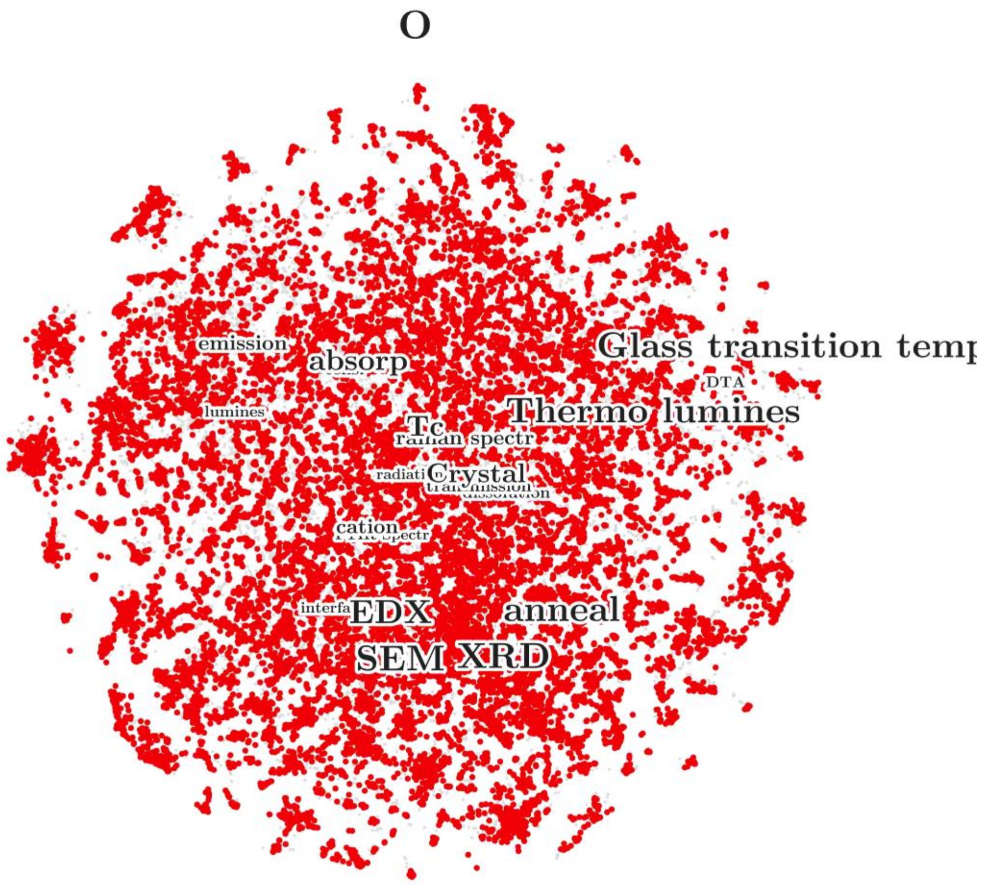

Figure S21. Latent Dirichlet Allocation plot for Oxygen.

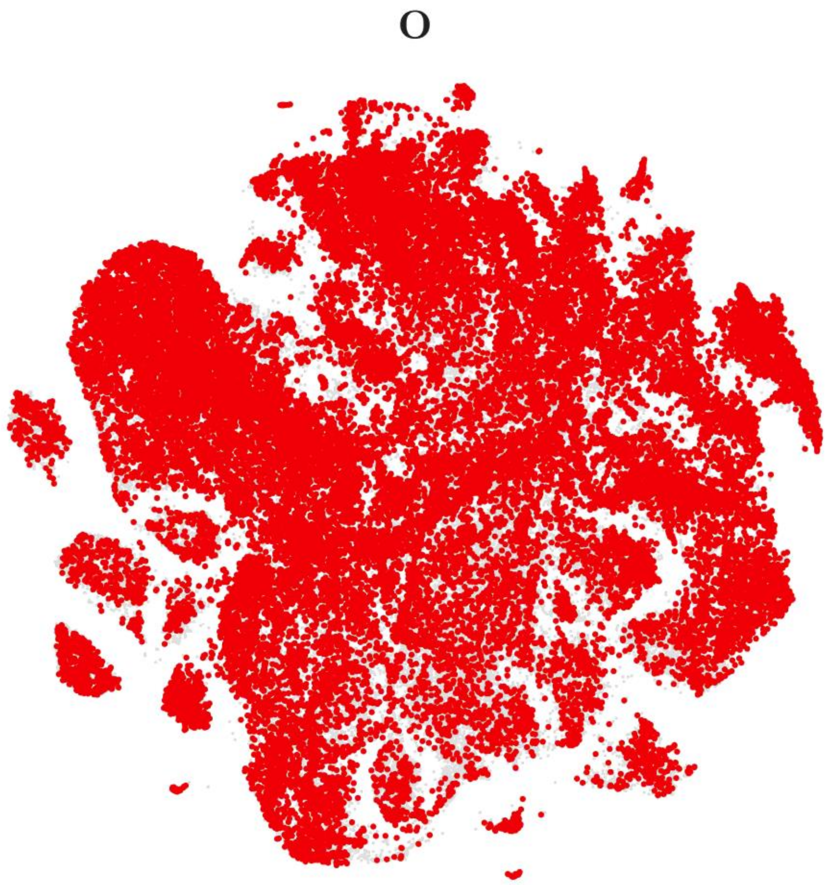

Figure S22. Caption Cluster plot for Fluorine.

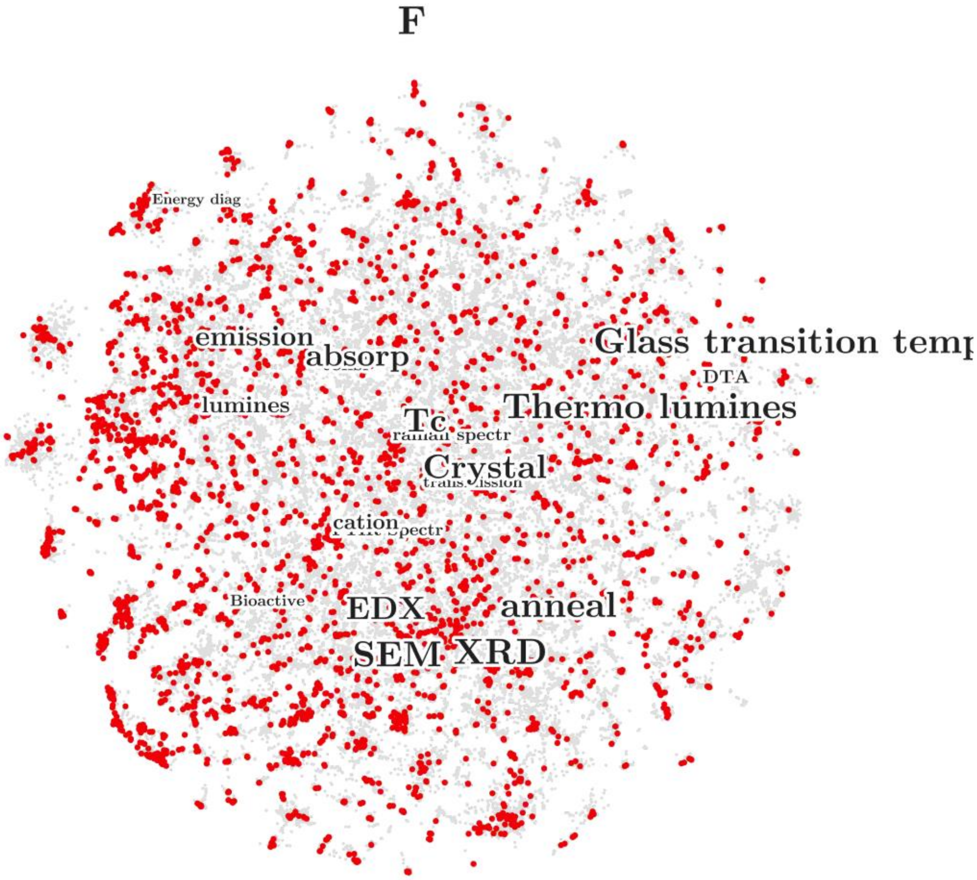

Figure S23. Latent Dirichlet Allocation plot for Fluorine.

F

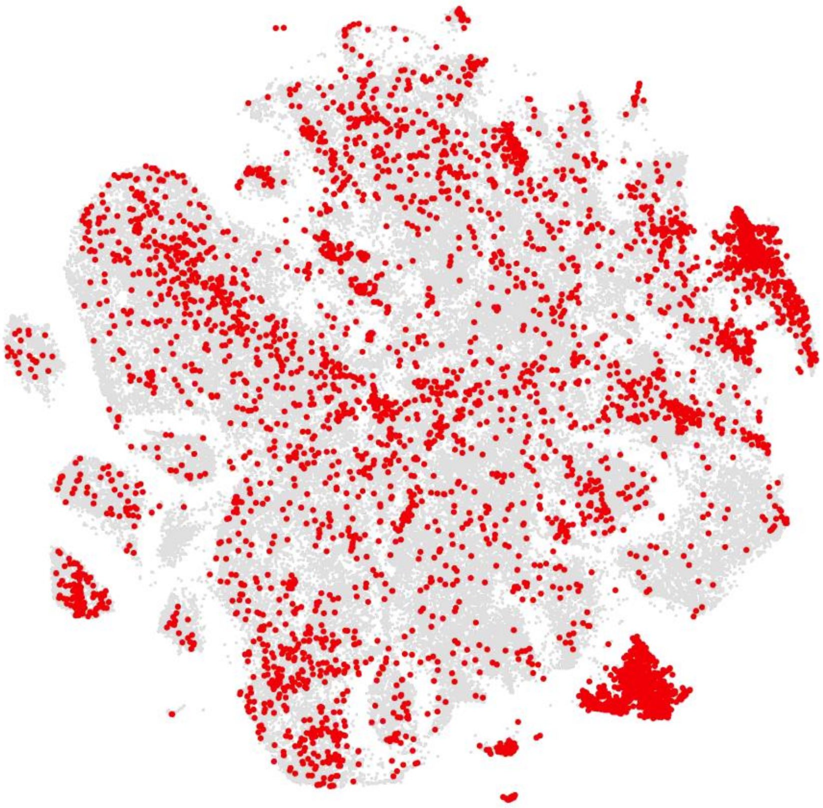

Figure S24. Caption Cluster plot for Neon.

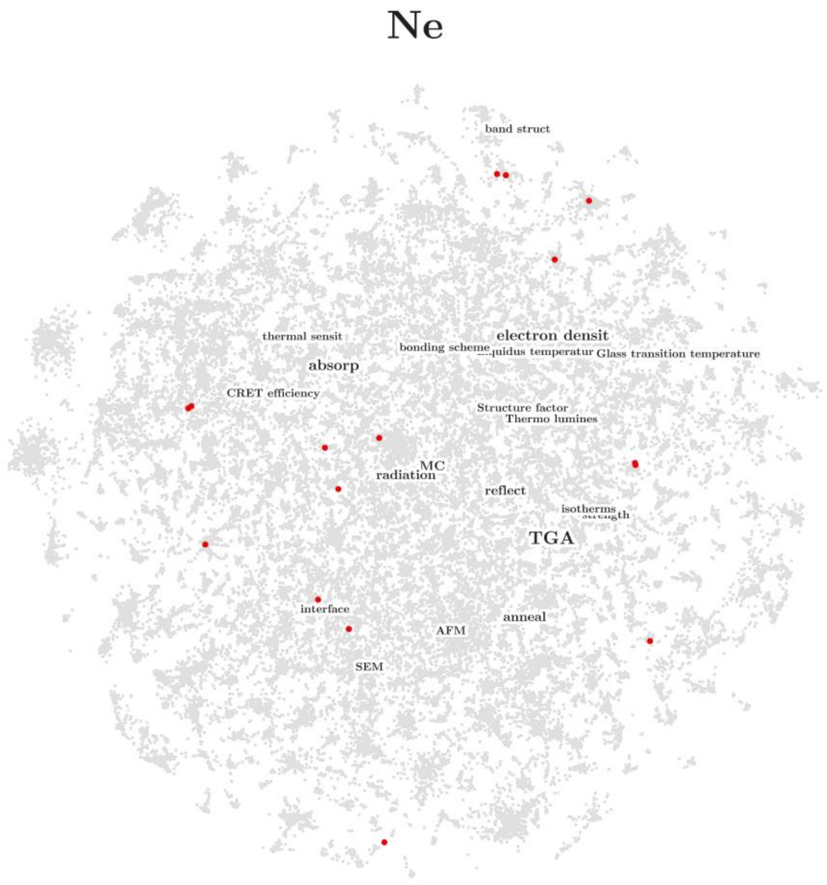

Figure S25. Latent Dirichlet Allocation plot for Neon.

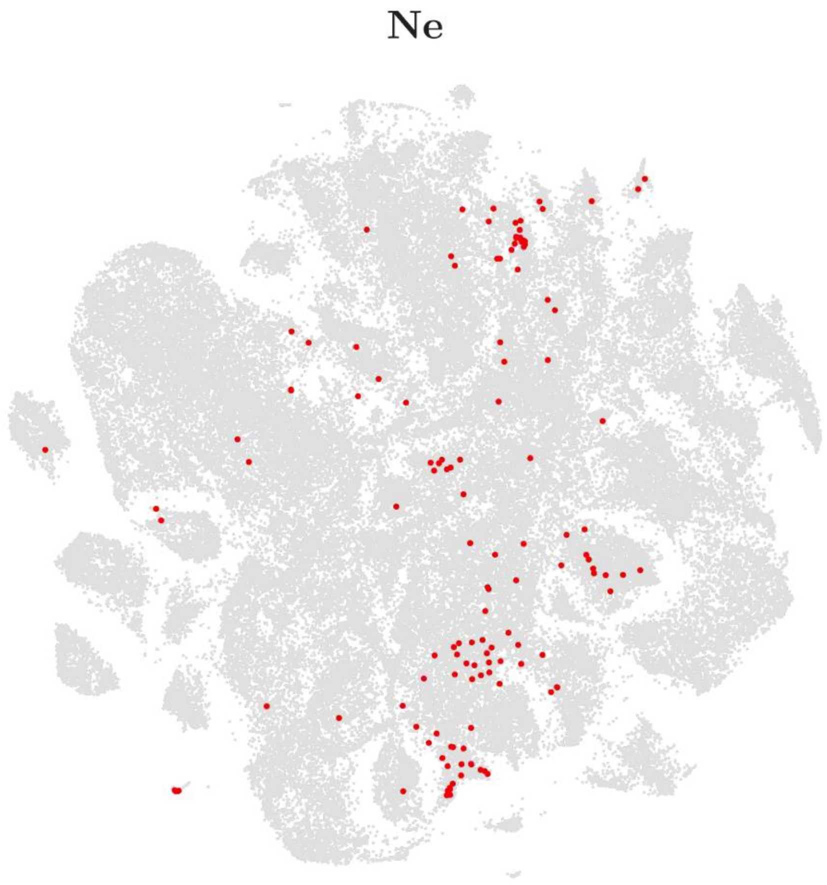

Figure S26. Caption Cluster plot for Sodium.

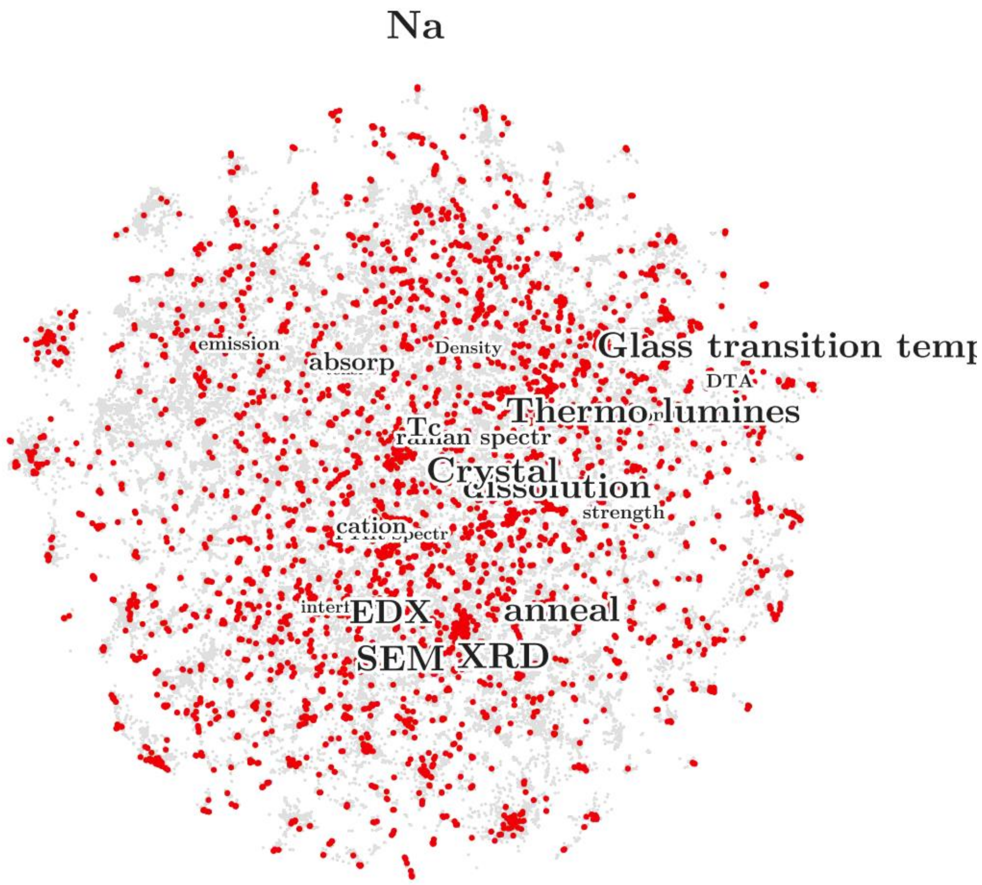

Figure S27. Latent Dirichlet Allocation plot for Sodium.

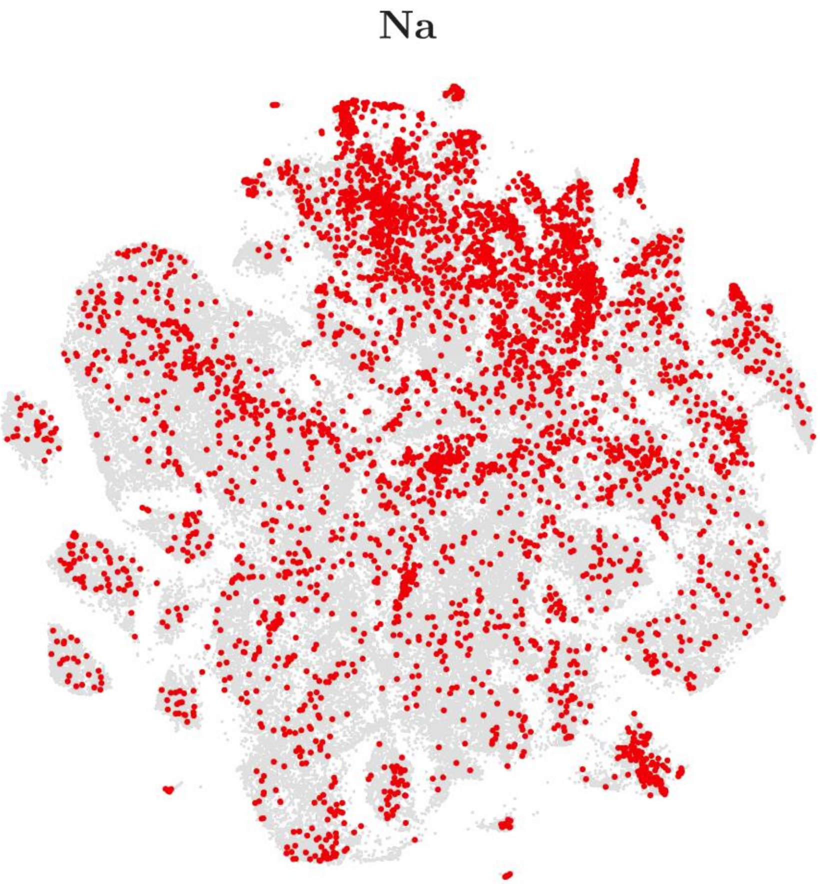

Figure S28. Caption Cluster plot for Magnesium.

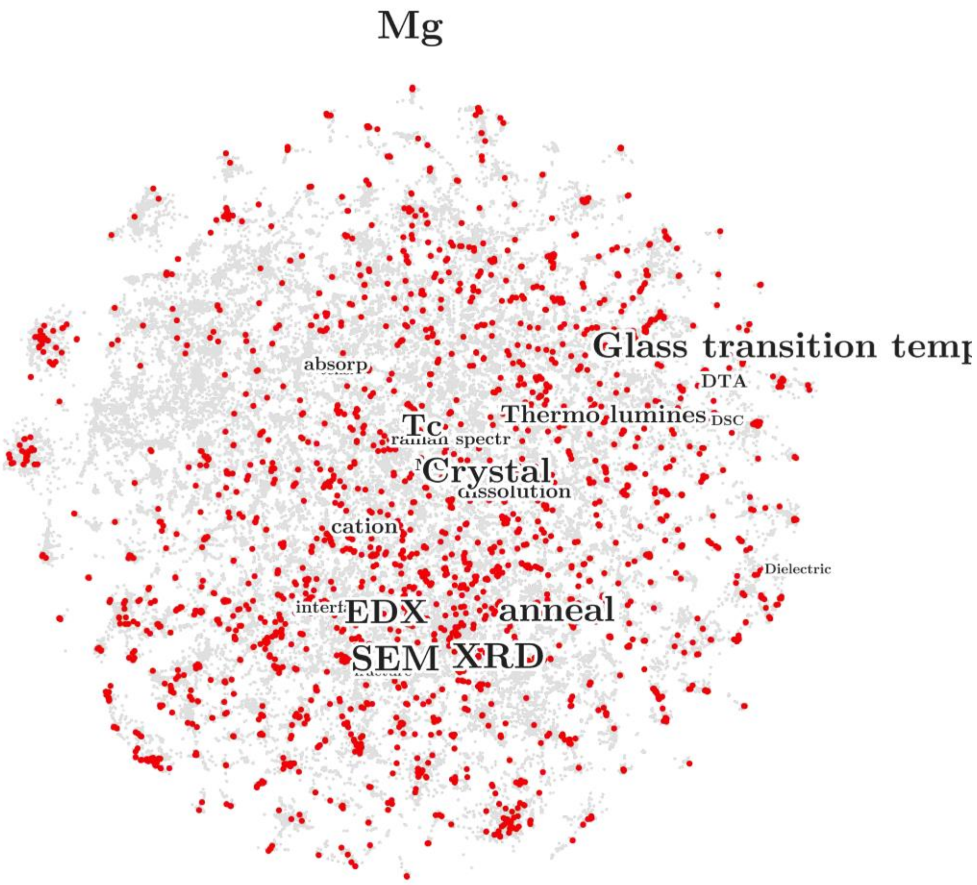

Figure S29. Latent Dirichlet Allocation plot for Magnesium.

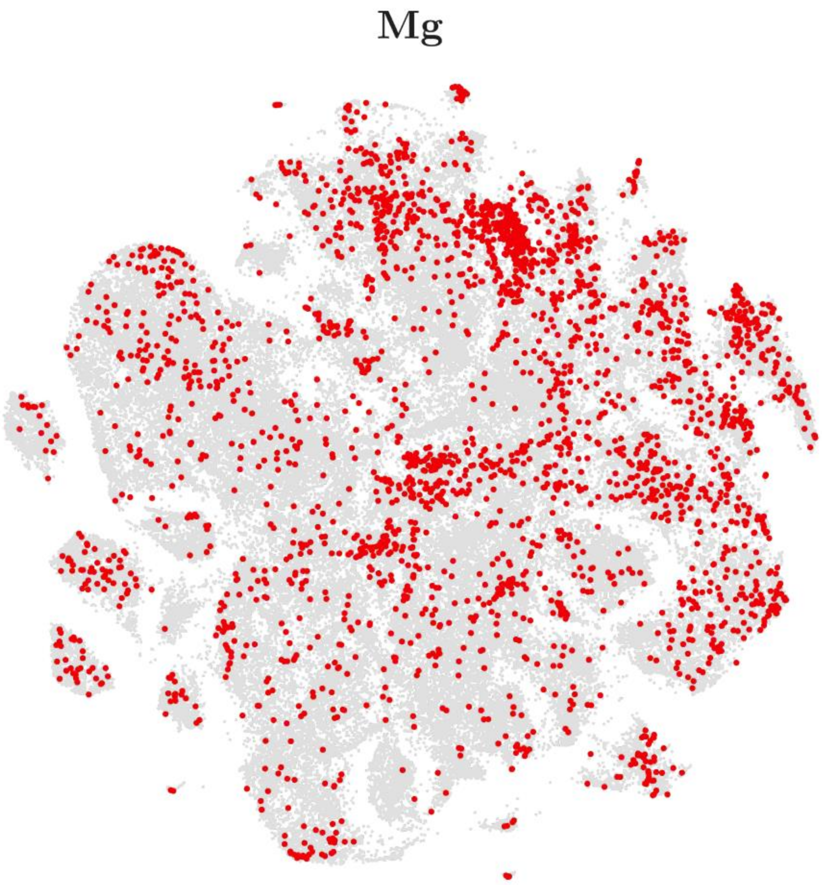

Figure S30. Caption Cluster plot for Aluminium.

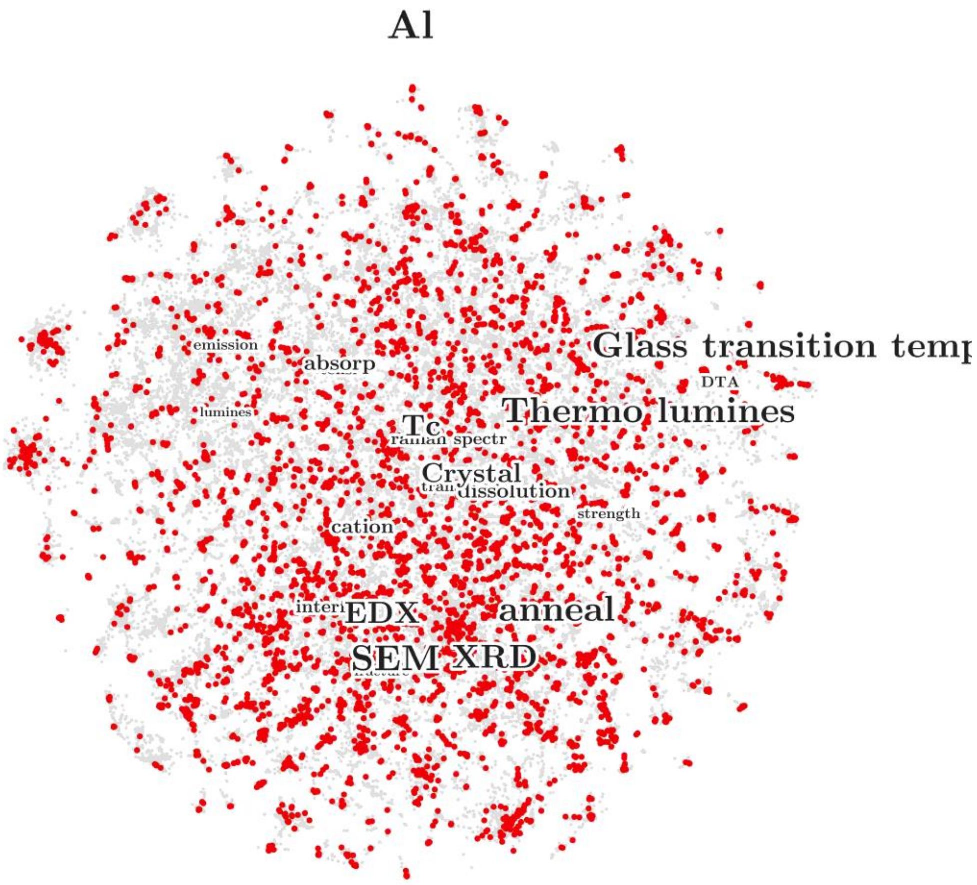

Figure S31. Latent Dirichlet Allocation plot for Aluminium.

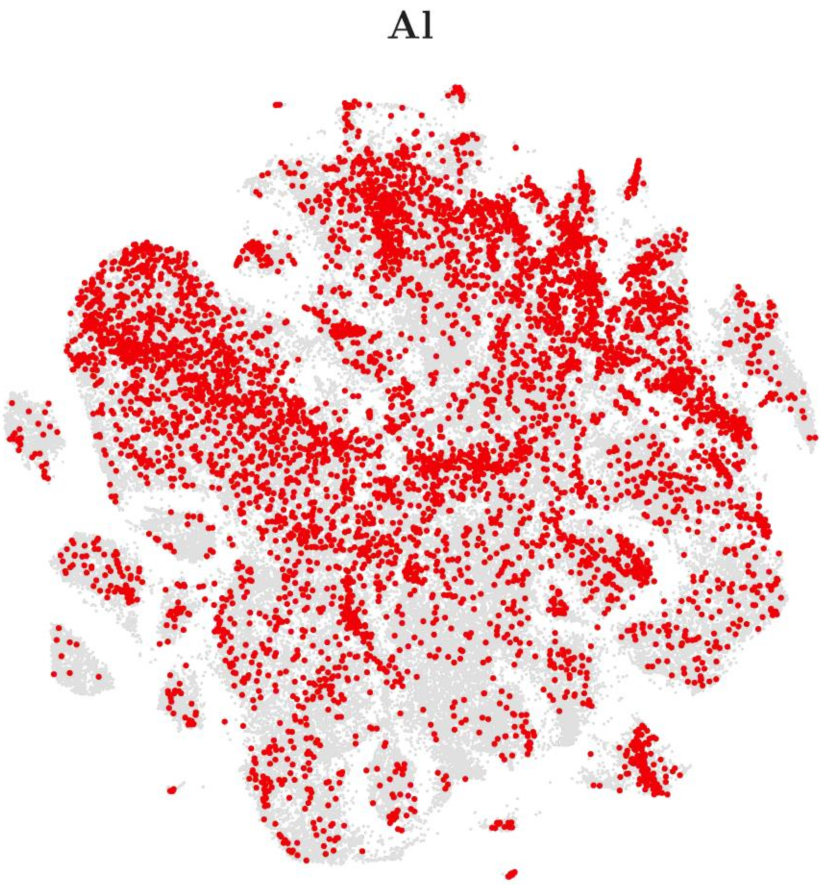

Figure S32. Caption Cluster plot for Silicon.

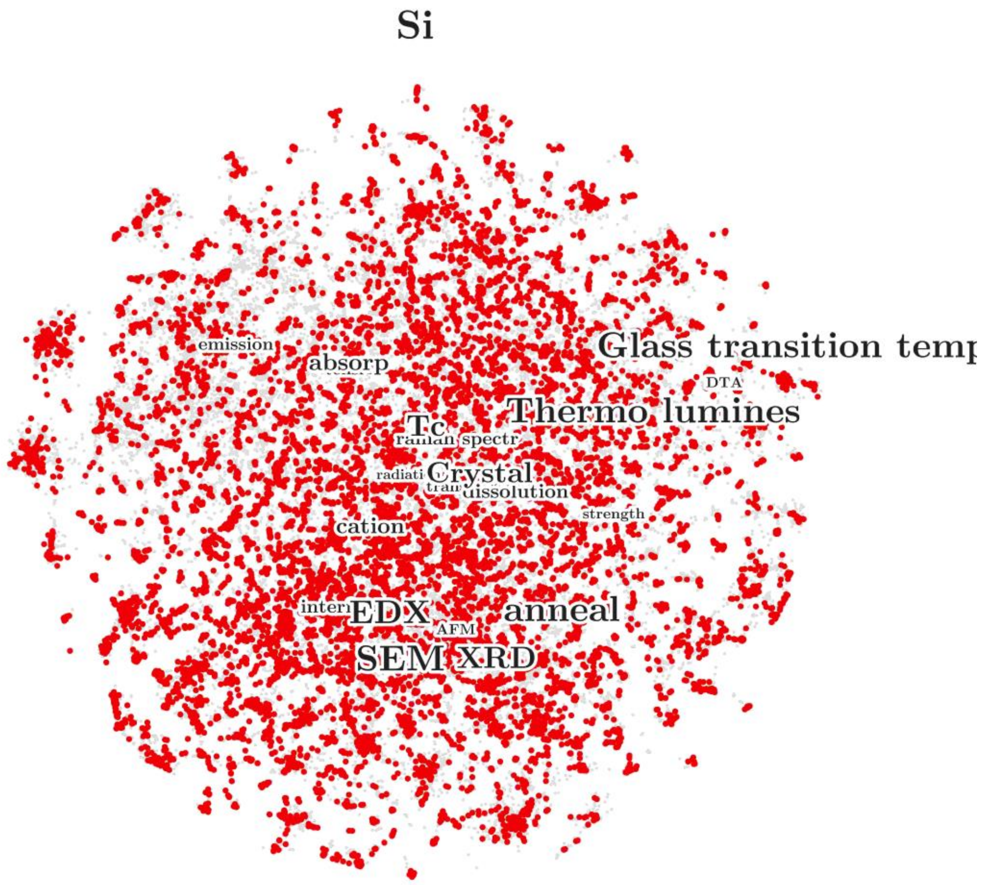

Figure S33. Latent Dirichlet Allocation plot for Silicon.

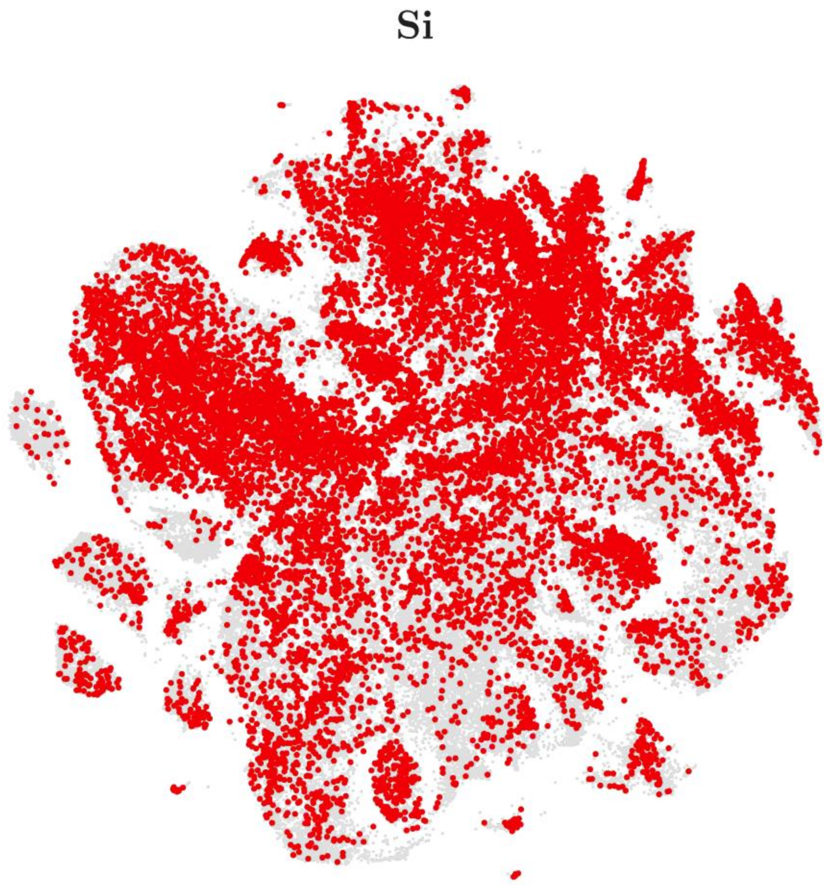

Figure S34. Caption Cluster plot for Phosphorus.

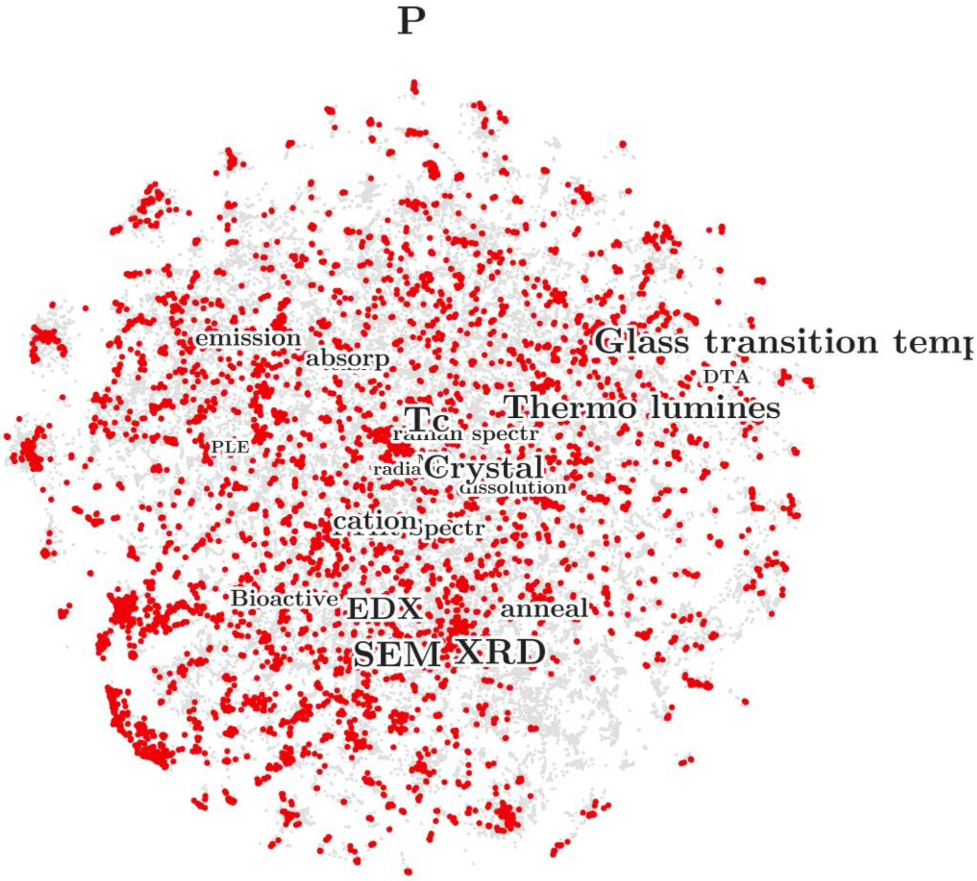

Figure S35. Latent Dirichlet Allocation plot for Phosphorus.

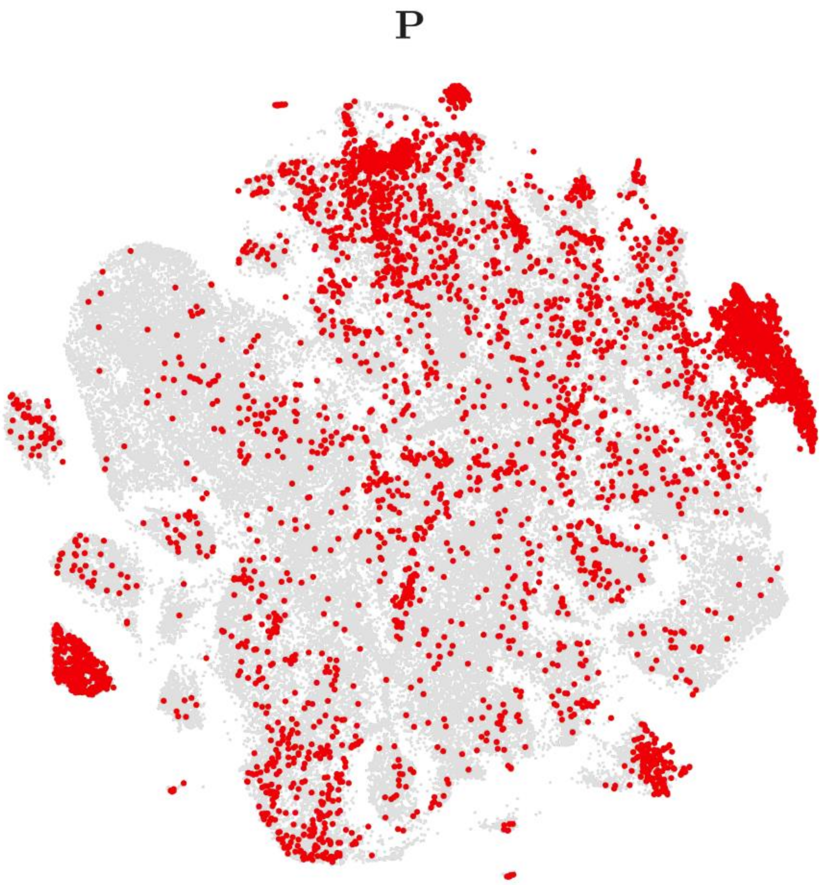

Figure S36. Caption Cluster plot for Sulfur.

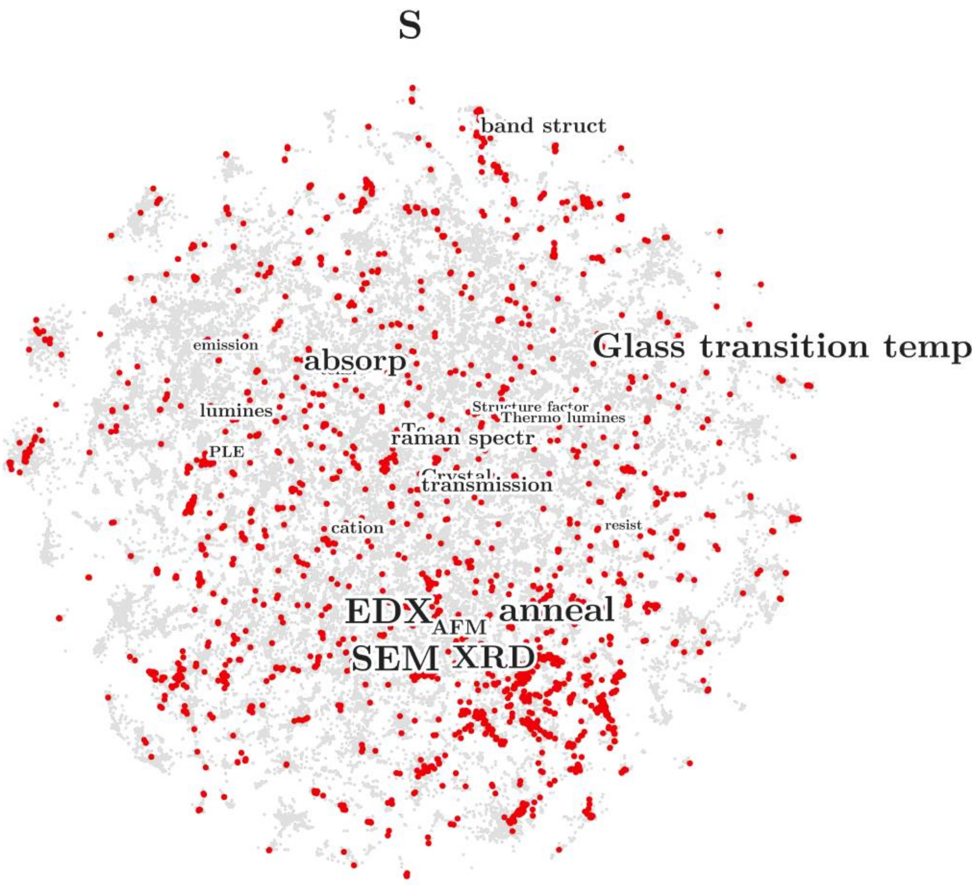

Figure S37. Latent Dirichlet Allocation plot for Sulfur.

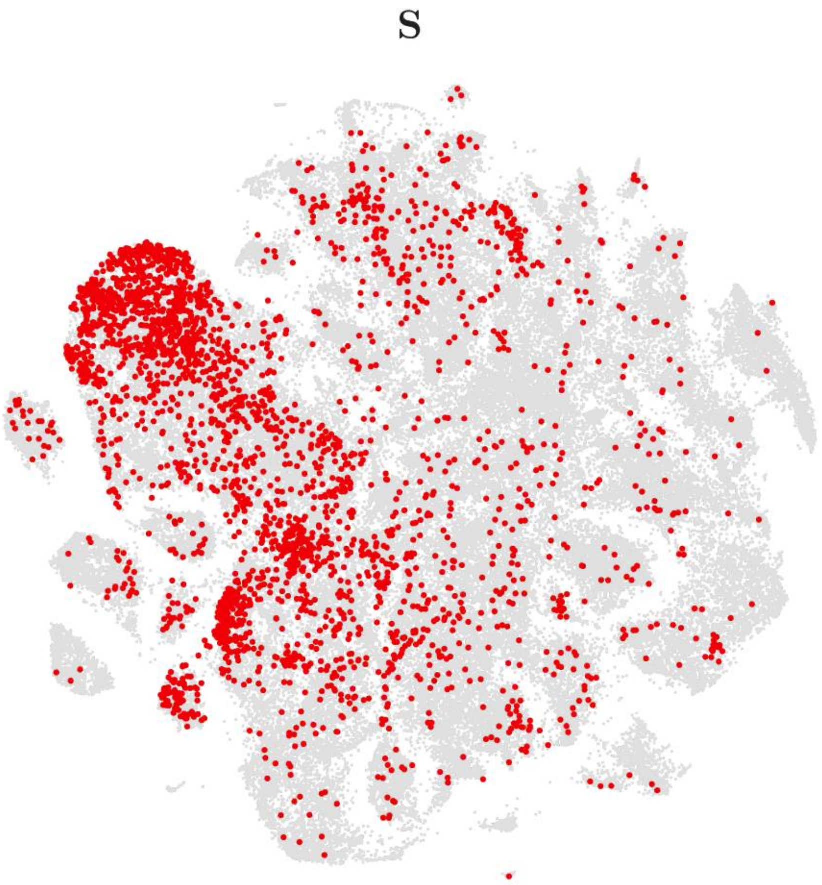

Figure S38. Caption Cluster plot for Chlorine.

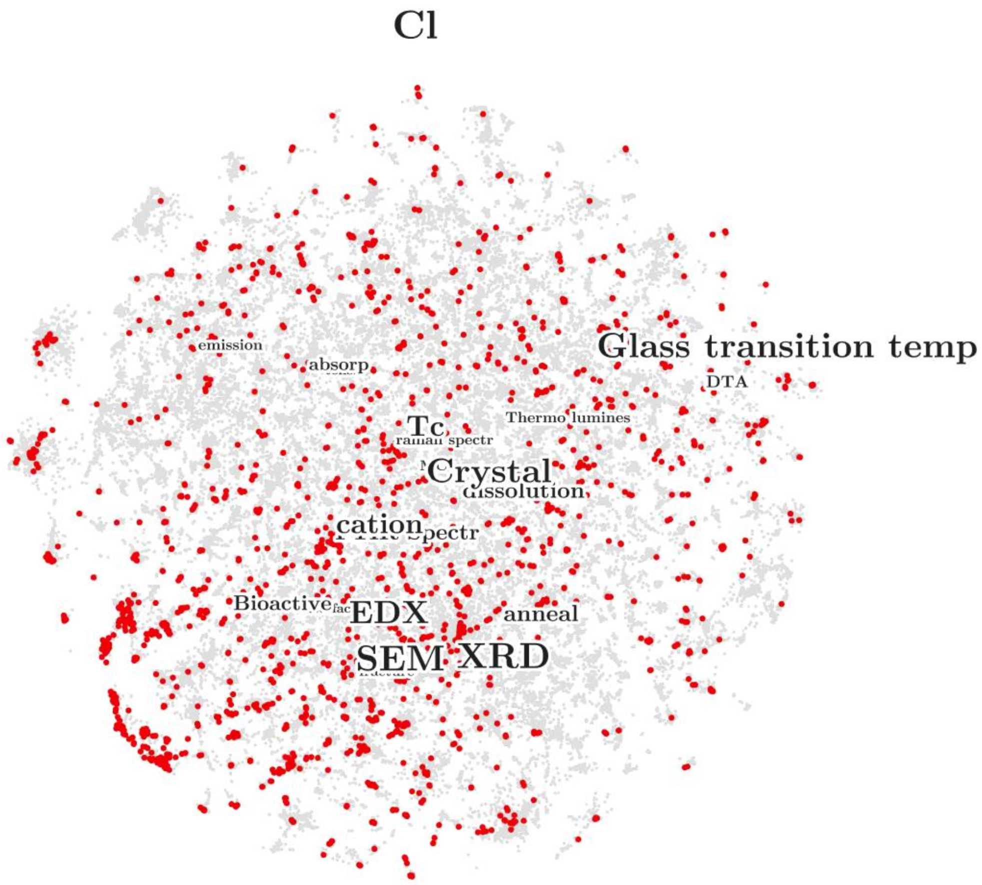

Figure S39. Latent Dirichlet Allocation plot for Chlorine.

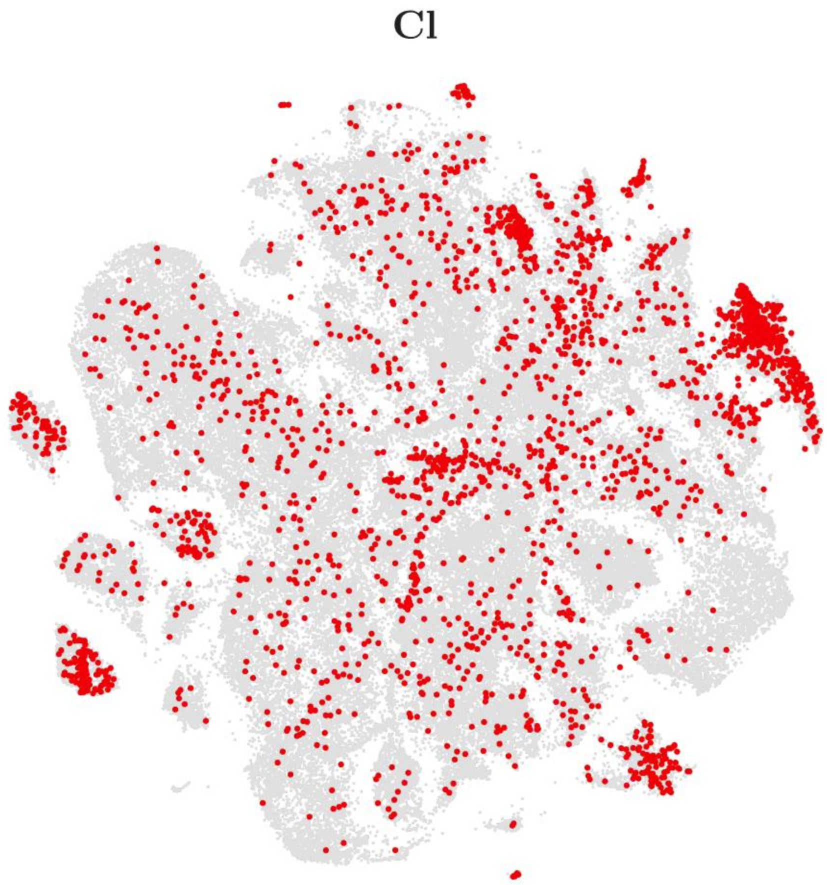

Figure S40. Caption Cluster plot for Argon.

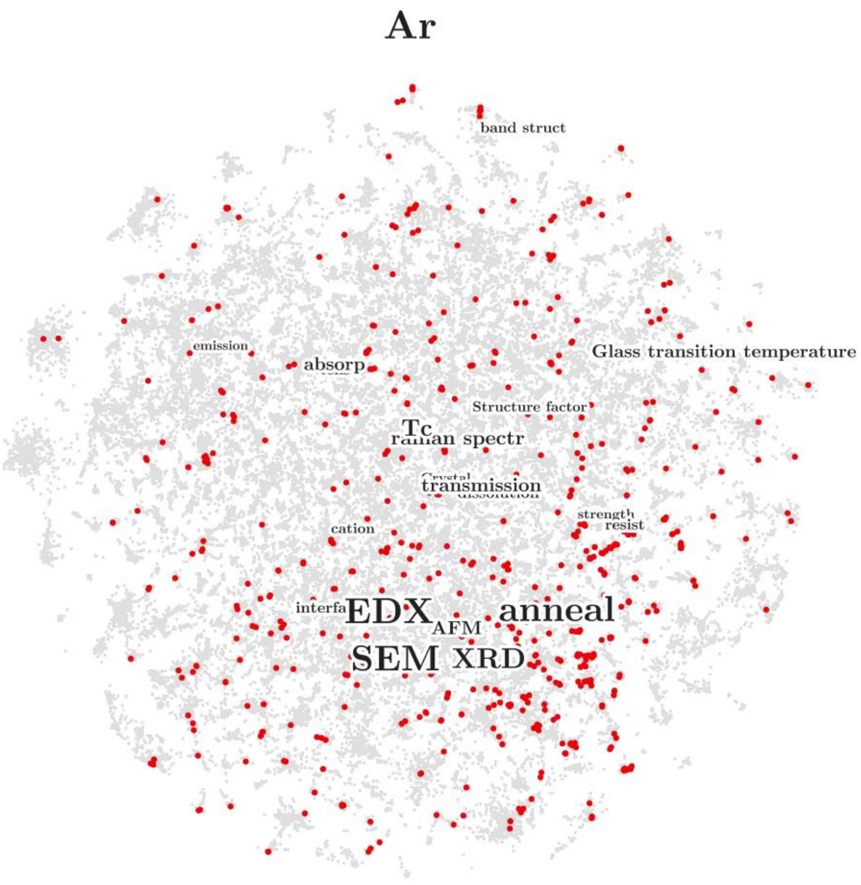

Figure S41. Latent Dirichlet Allocation plot for Argon.

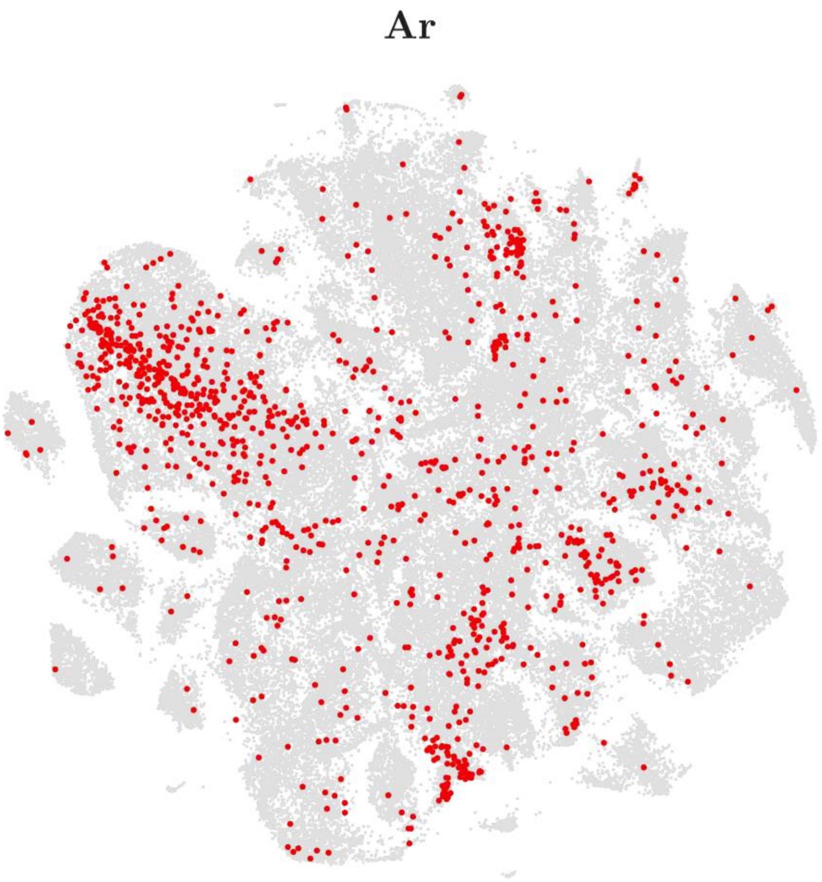

Figure S42. Caption Cluster plot for Potassium.

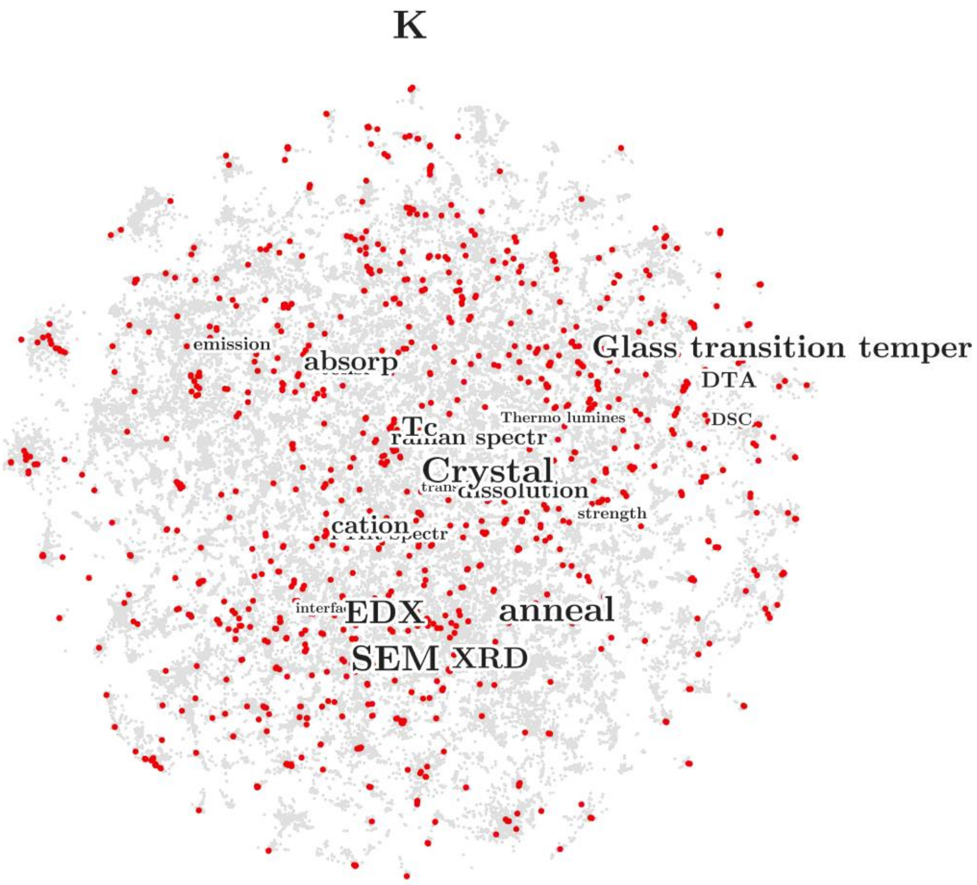

Figure S43. Latent Dirichlet Allocation plot for Potassium.

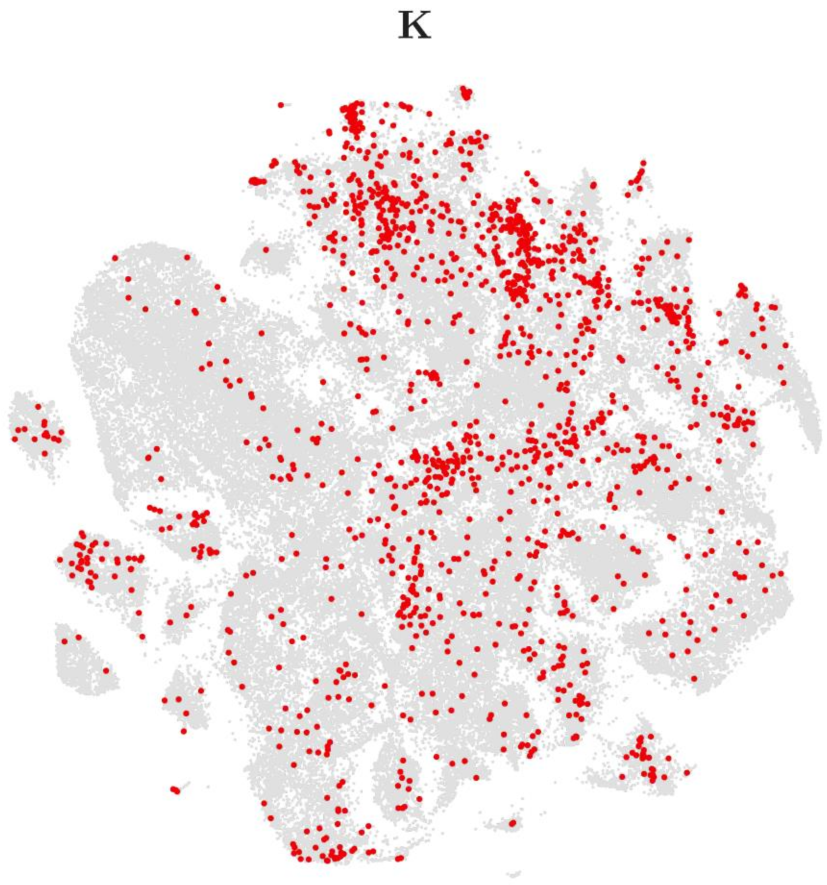

Figure S44. Caption Cluster plot for Calcium.

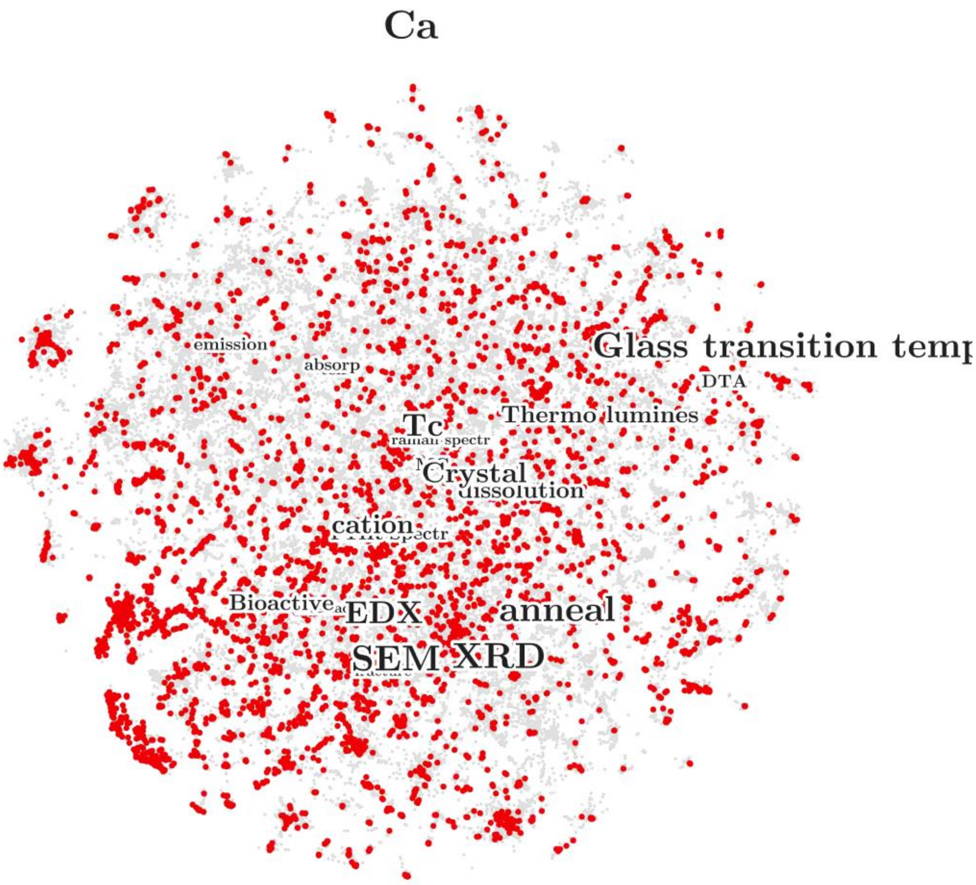

Figure S45. Latent Dirichlet Allocation plot for Calcium.

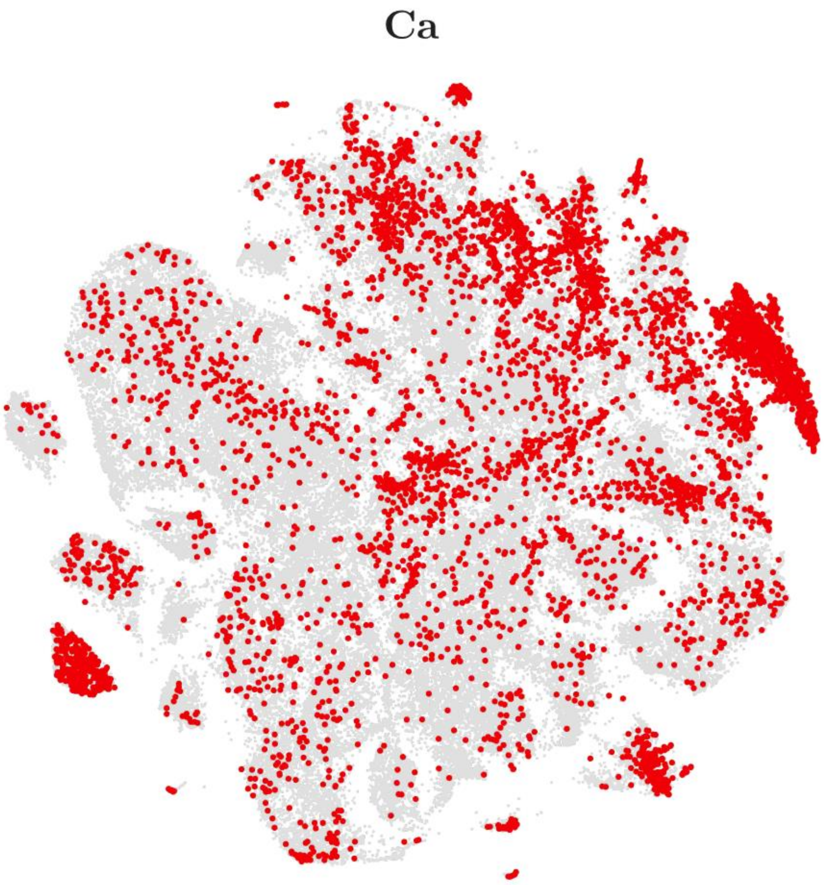

Figure S46. Caption Cluster plot for Scandium.

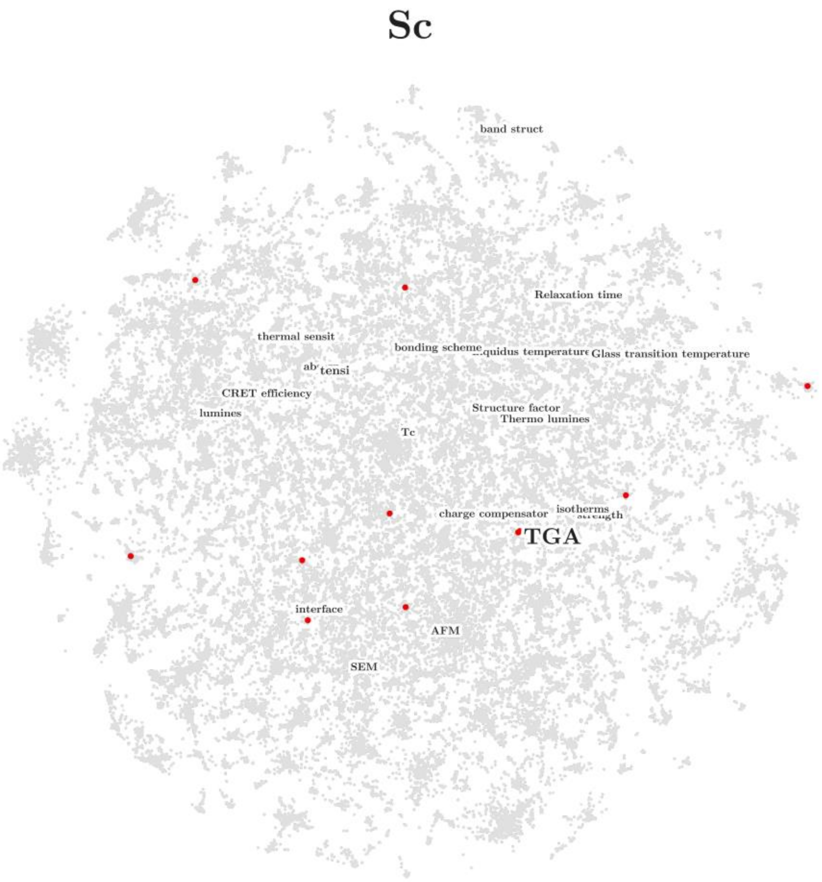

Figure S47. Latent Dirichlet Allocation plot for Scandium.

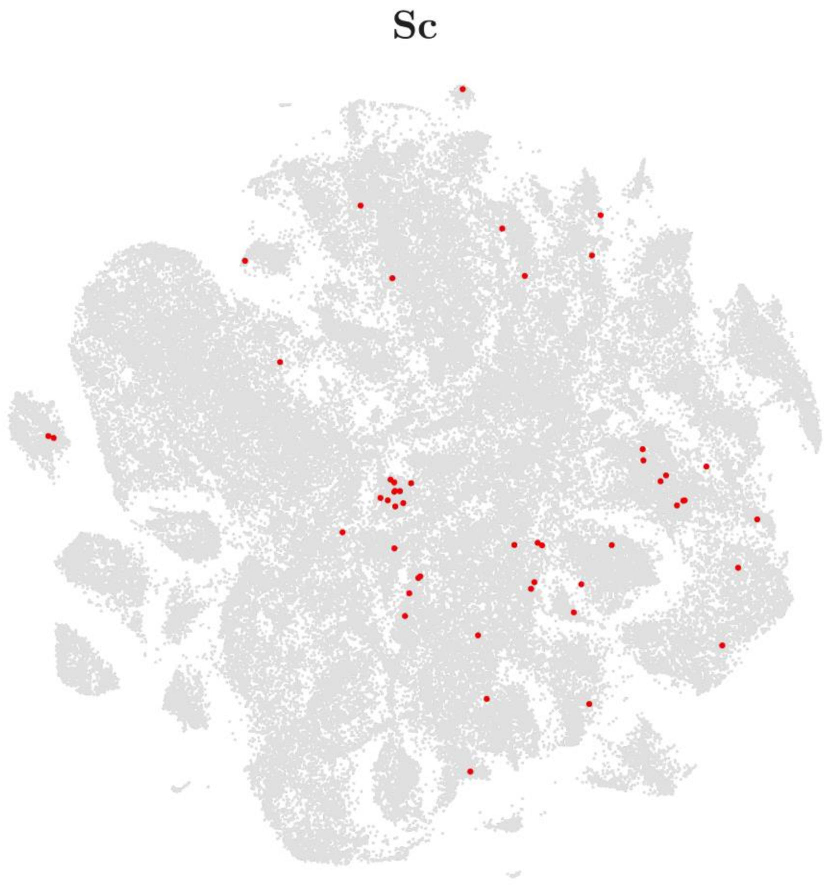

Figure S48. Caption Cluster plot for Titanium.

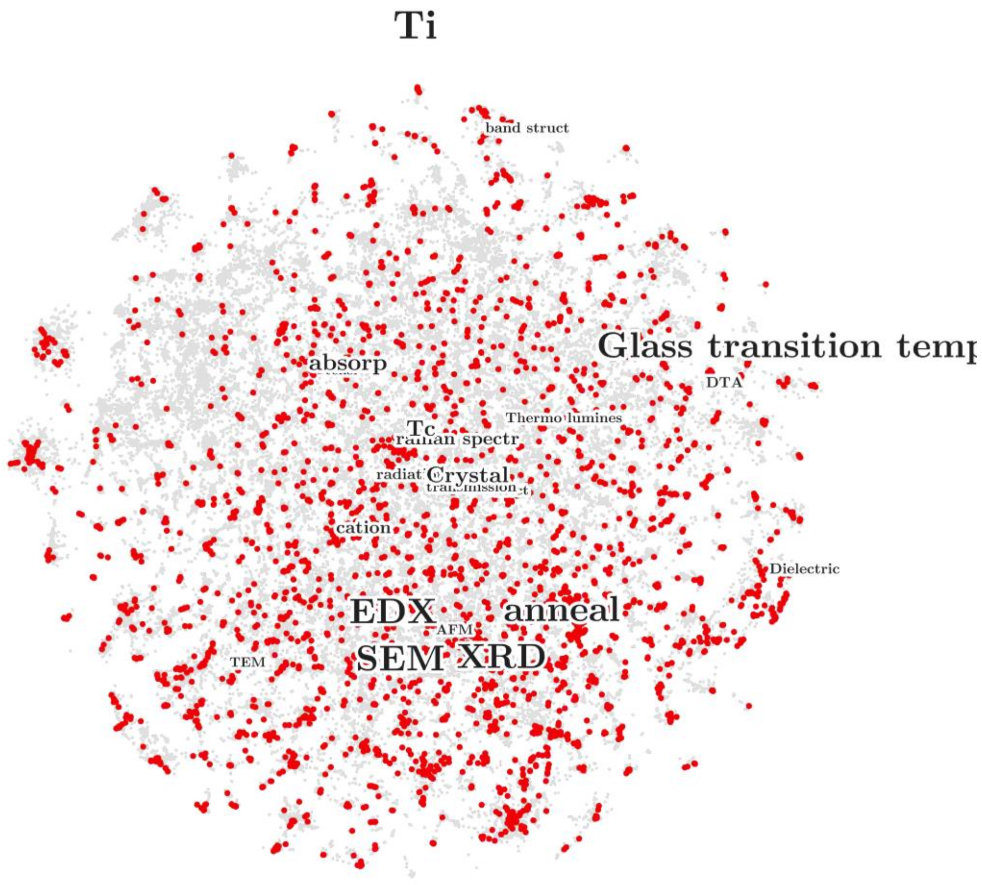

Figure S49. Latent Dirichlet Allocation plot for Titanium.

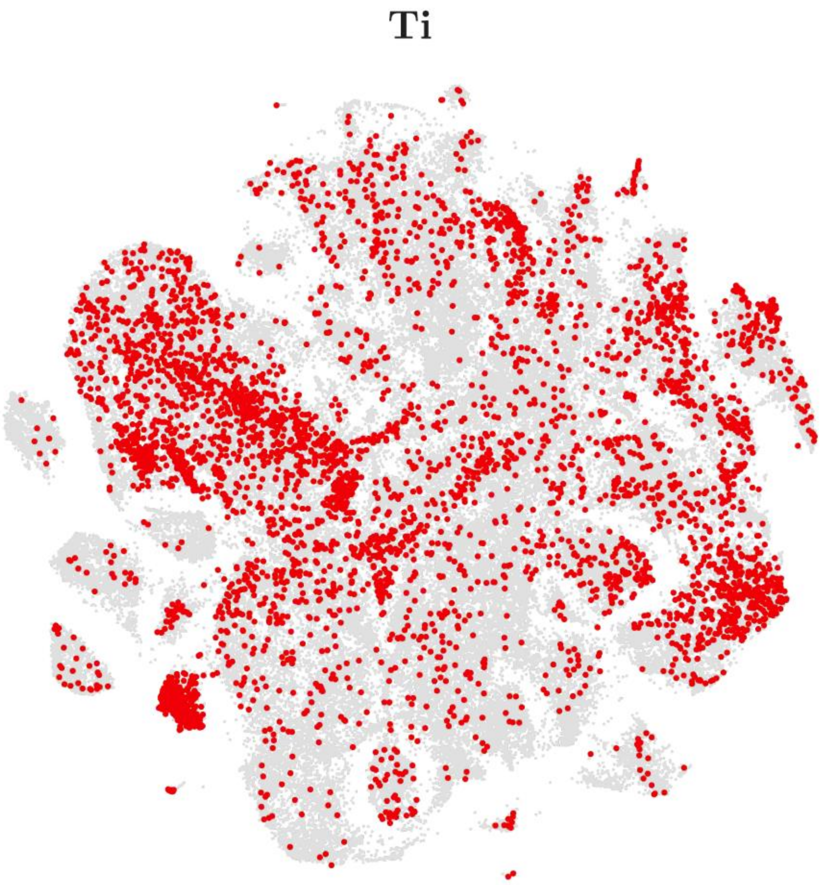

Figure S50. Caption Cluster plot for Vanadium.

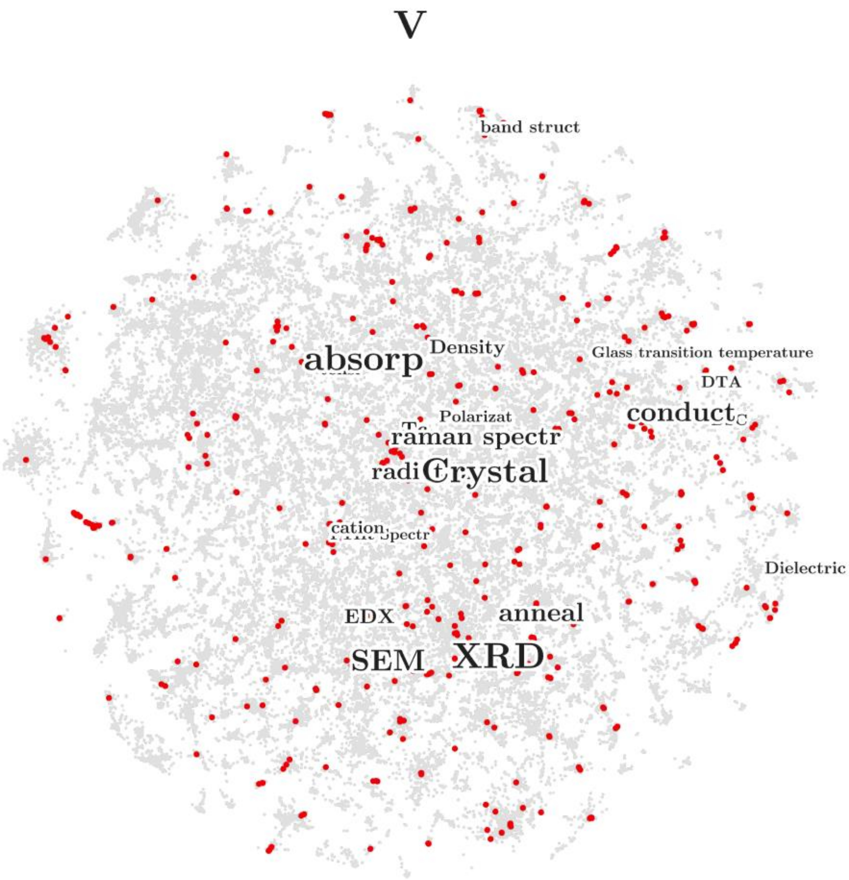

Figure S51. Latent Dirichlet Allocation plot for Vanadium.

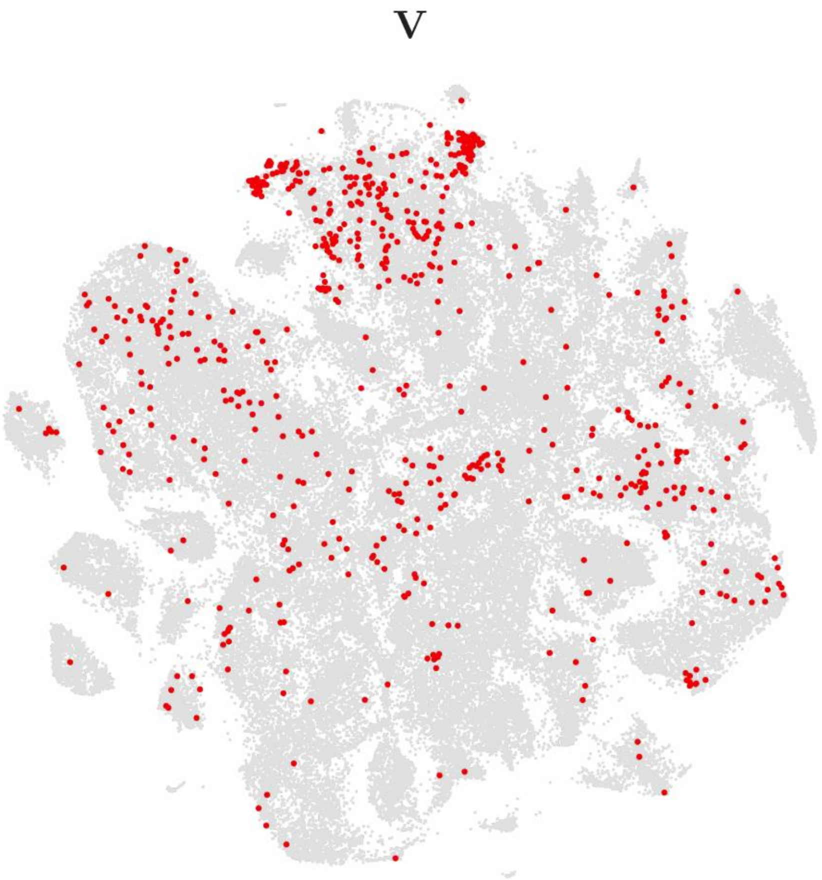

Figure S52. Caption Cluster plot for Chromium.

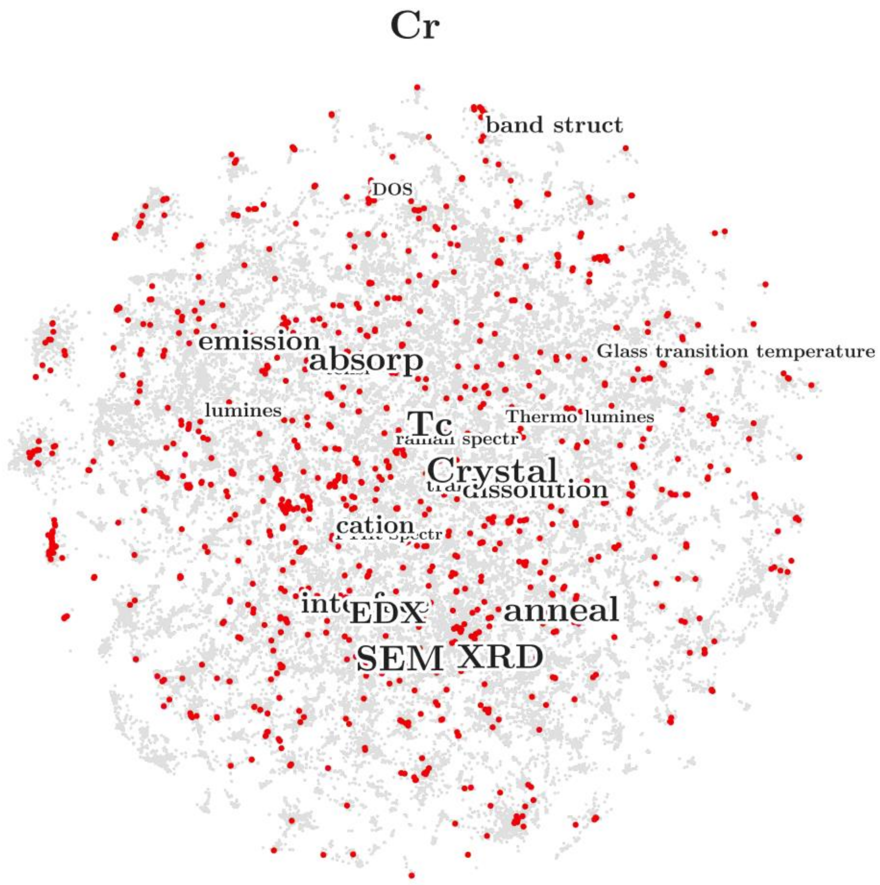

Figure S53. Latent Dirichlet Allocation plot for Chromium.

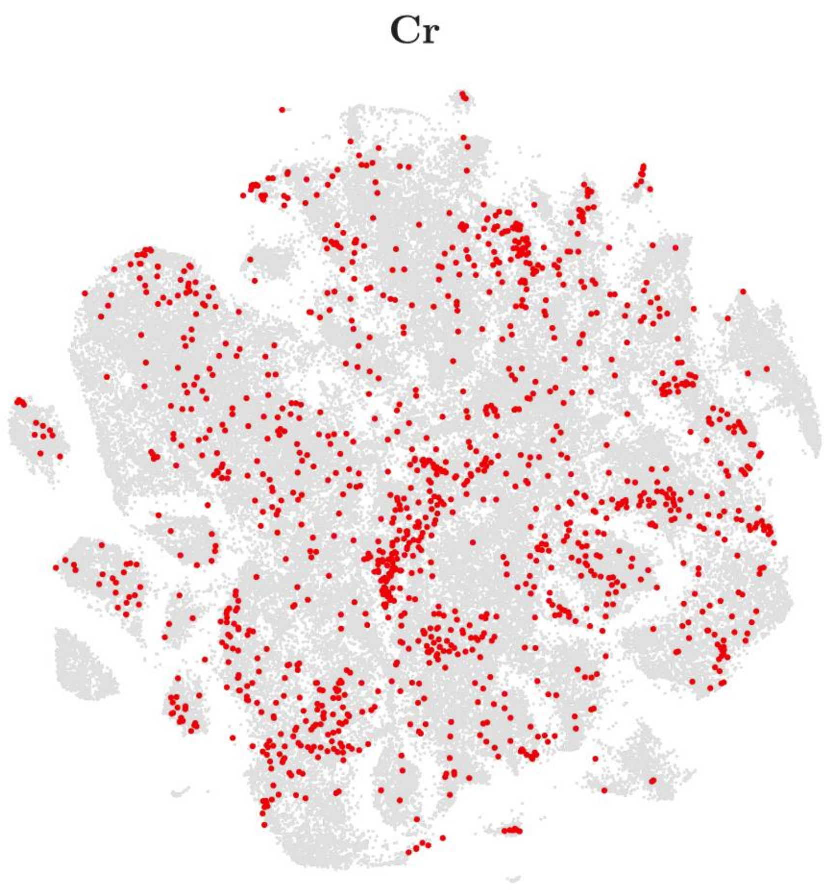

Figure S54. Caption Cluster plot for Manganese.

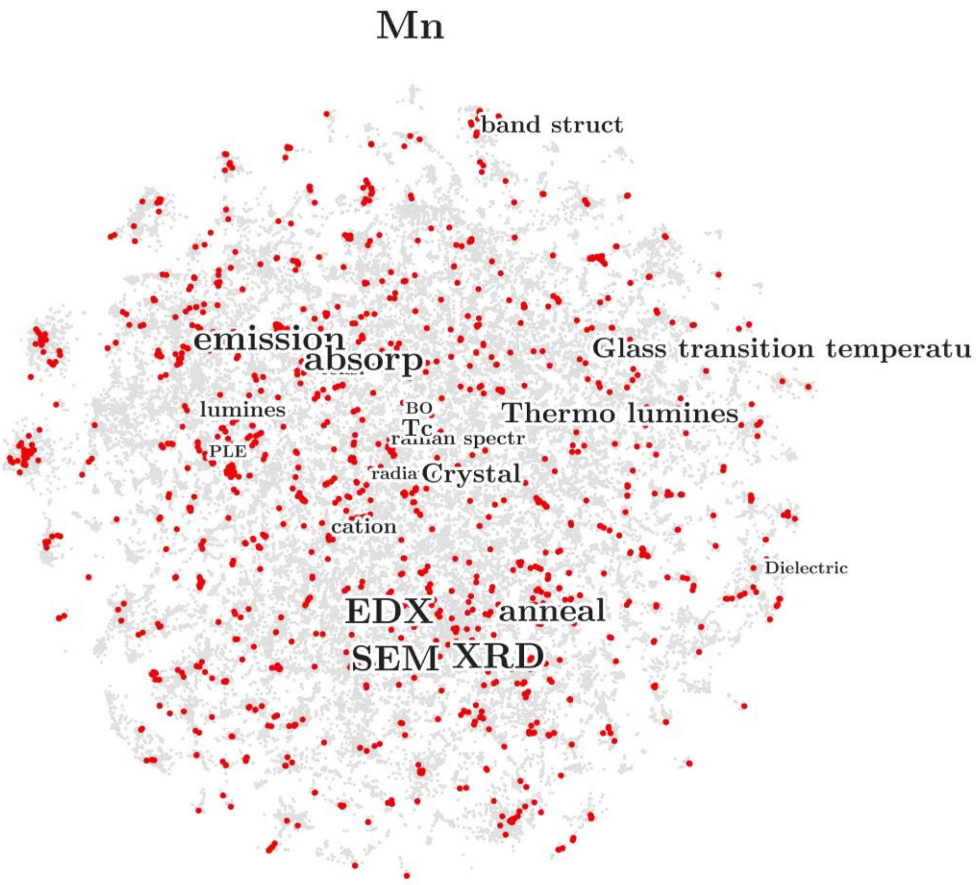

Figure S55. Latent Dirichlet Allocation plot for Manganese.

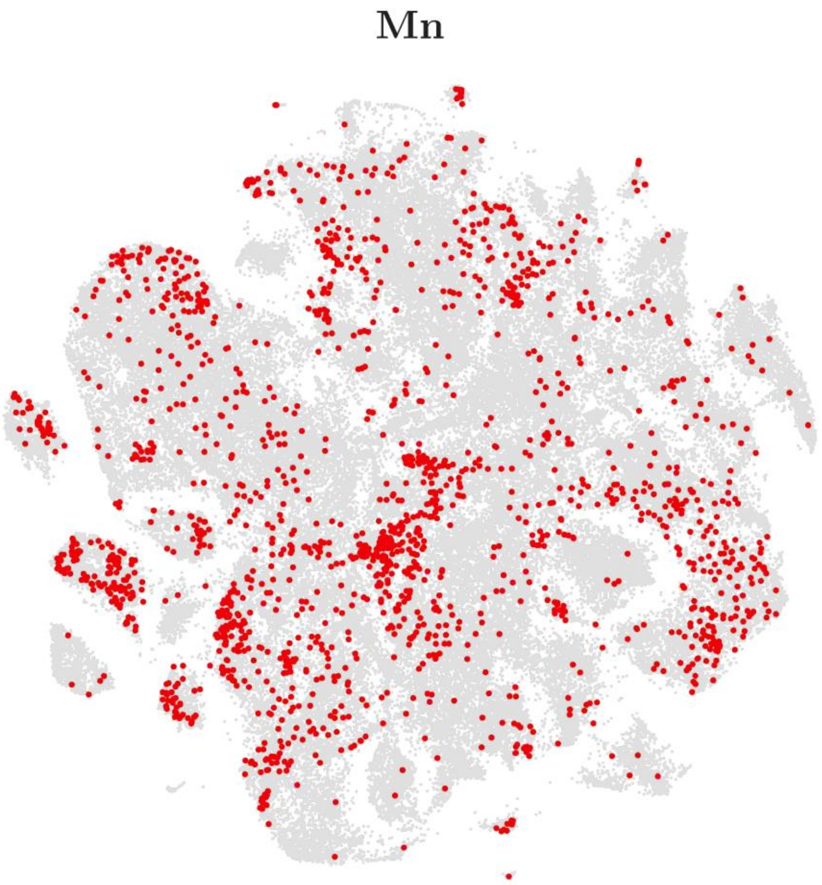

Figure S56. Caption Cluster plot for Iron.

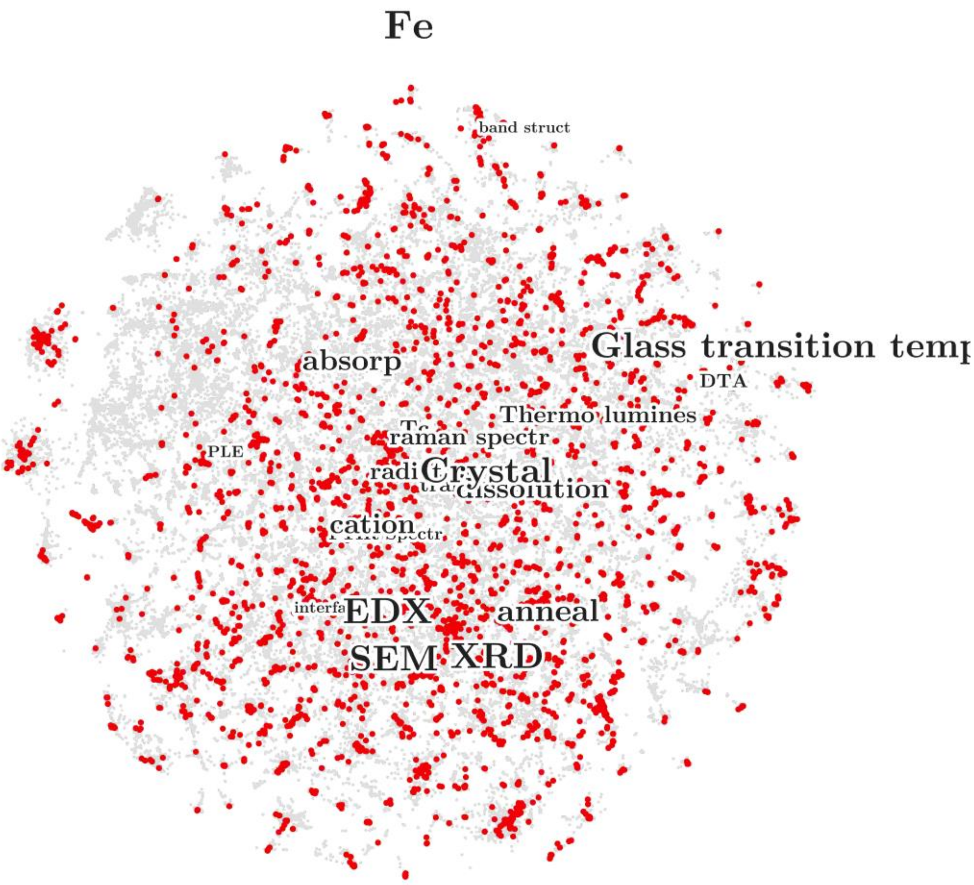

Figure S57. Latent Dirichlet Allocation plot for Iron.

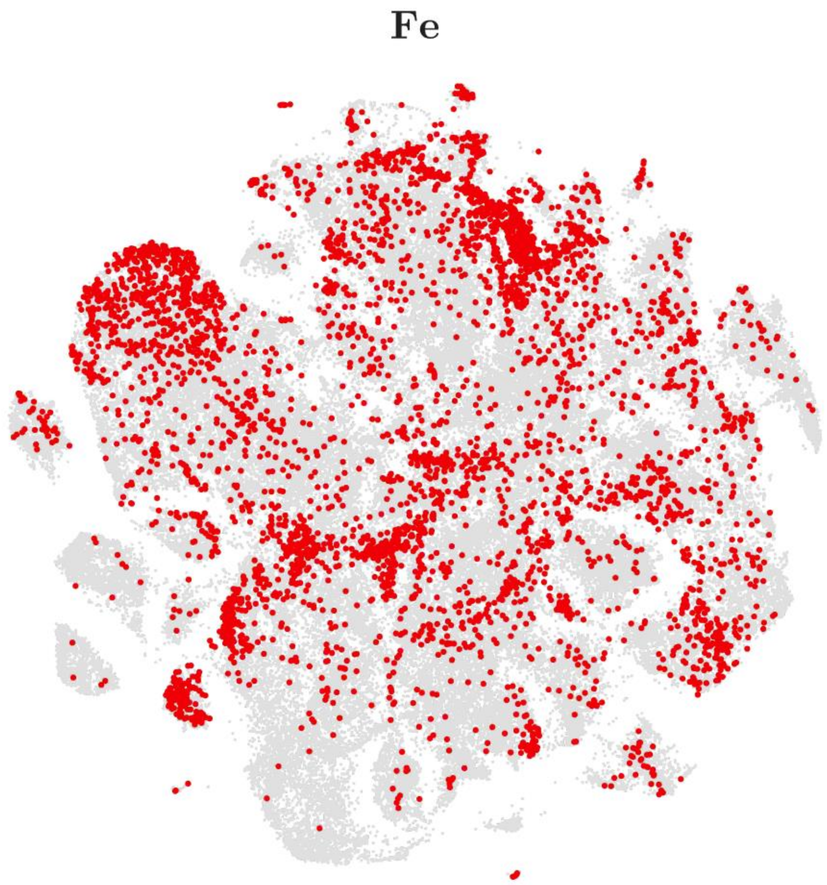

Figure S58. Caption Cluster plot for Cobalt.

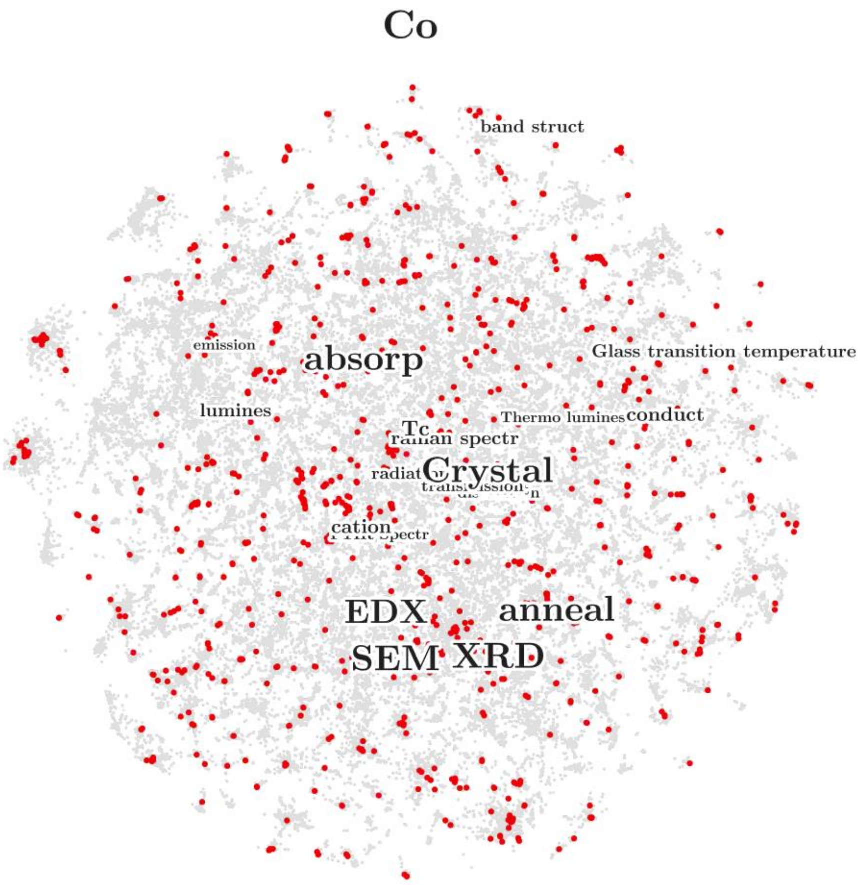

Figure S59. Latent Dirichlet Allocation plot for Cobalt.

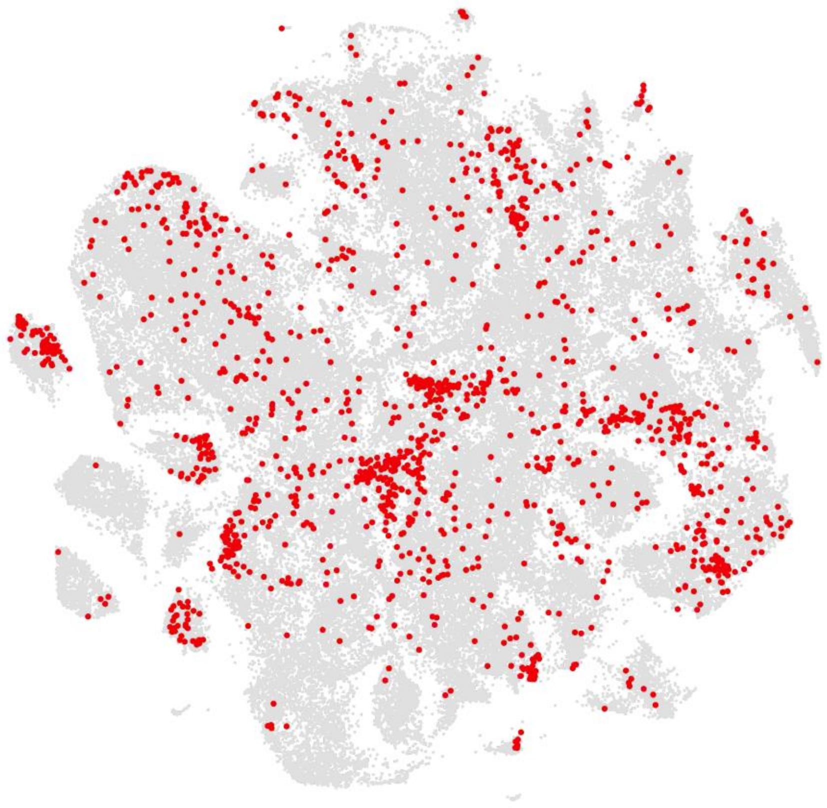

Figure S60. Caption Cluster plot for Nickel.

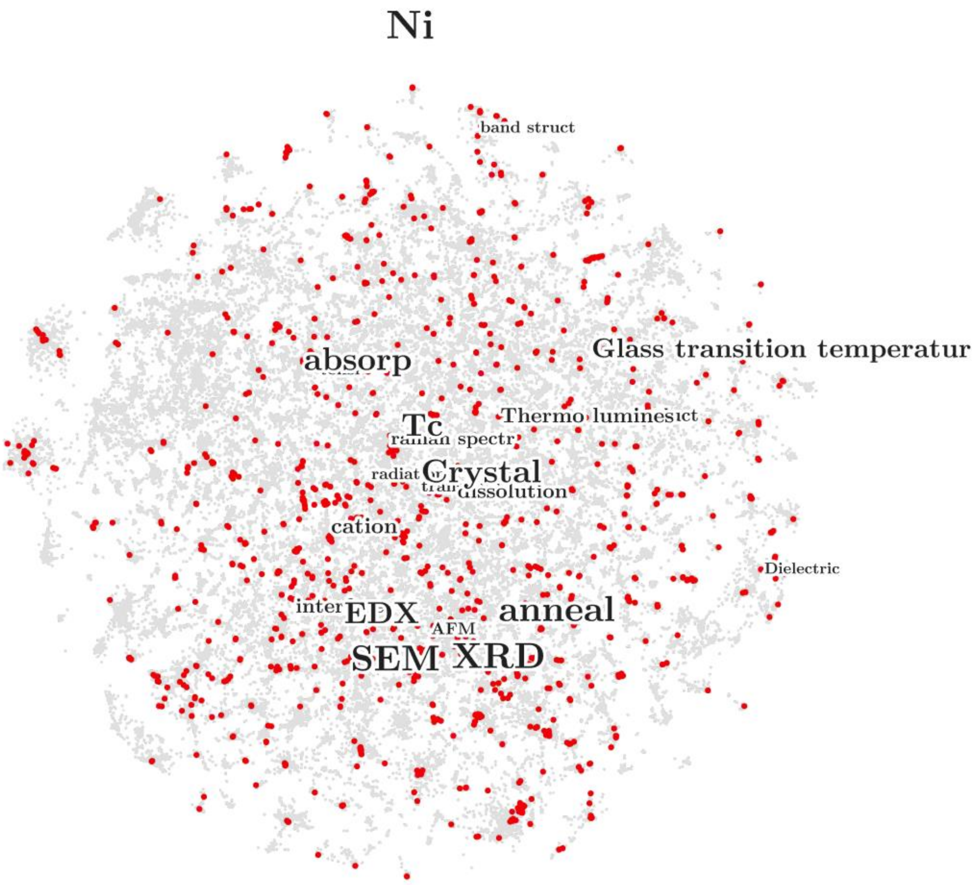

Figure S61. Latent Dirichlet Allocation plot for Nickel.

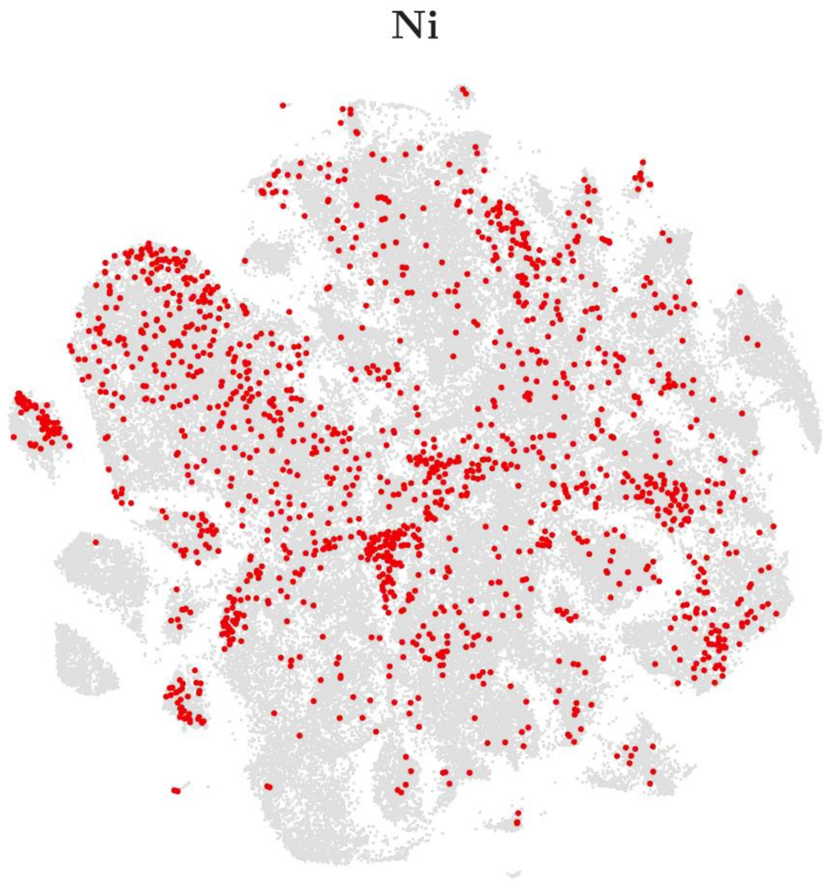

Figure S62. Caption Cluster plot for Copper.

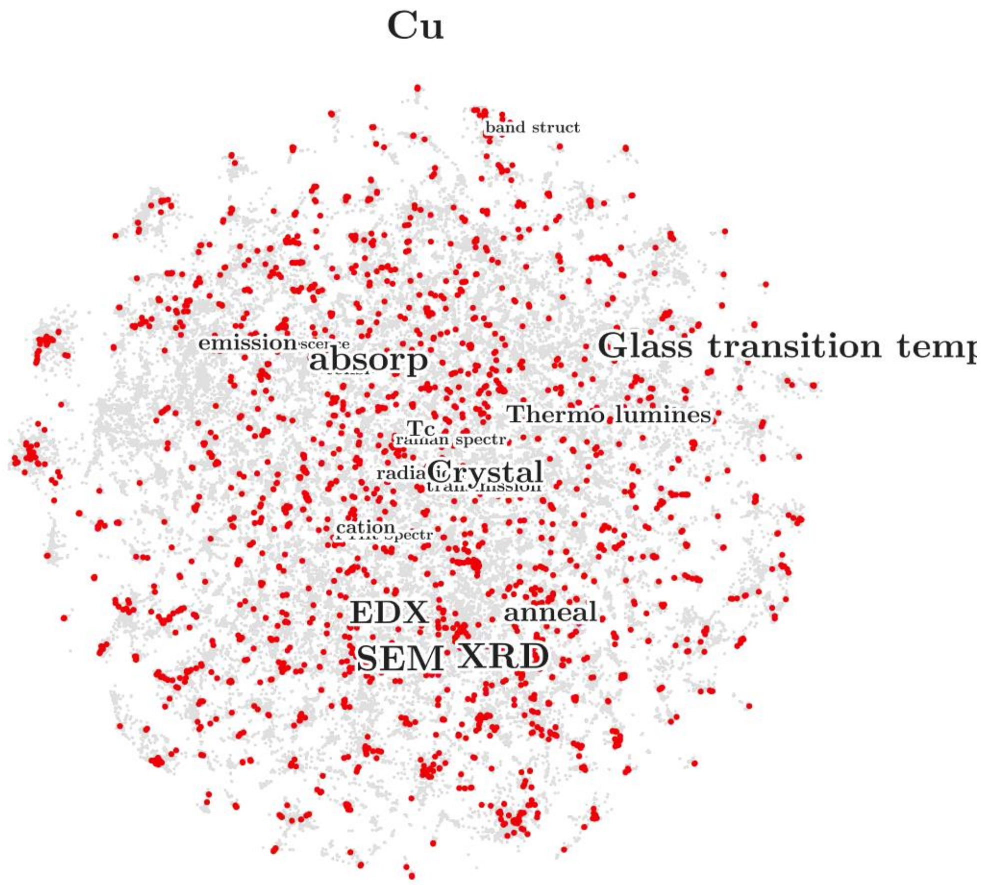

Figure S63. Latent Dirichlet Allocation plot for Copper.

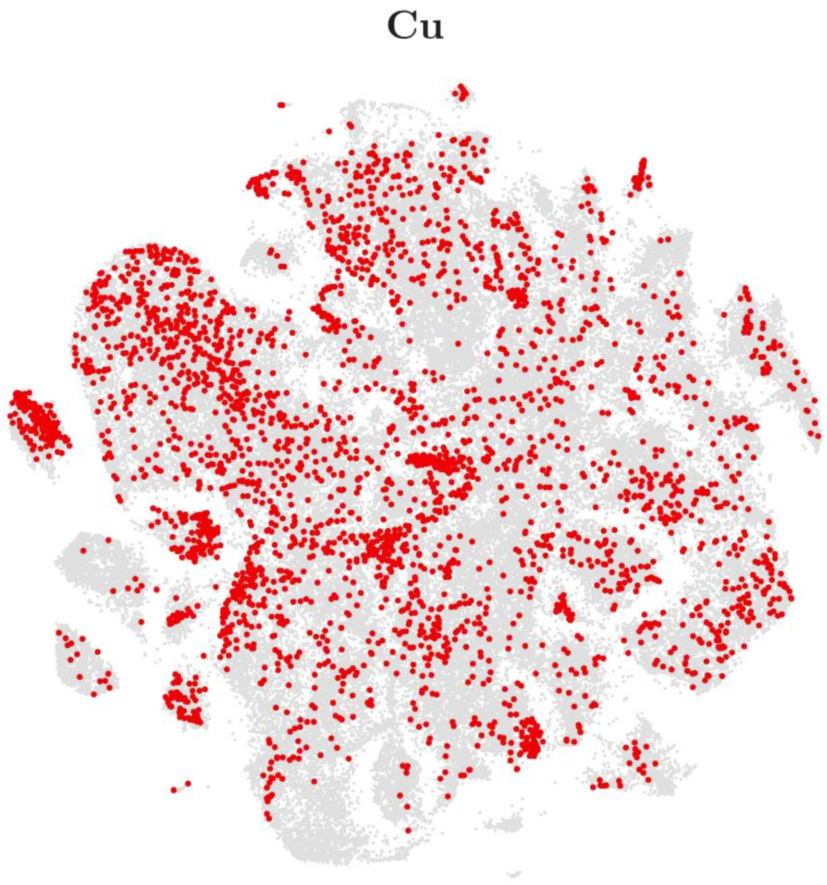

Figure S64. Caption Cluster plot for Zinc.

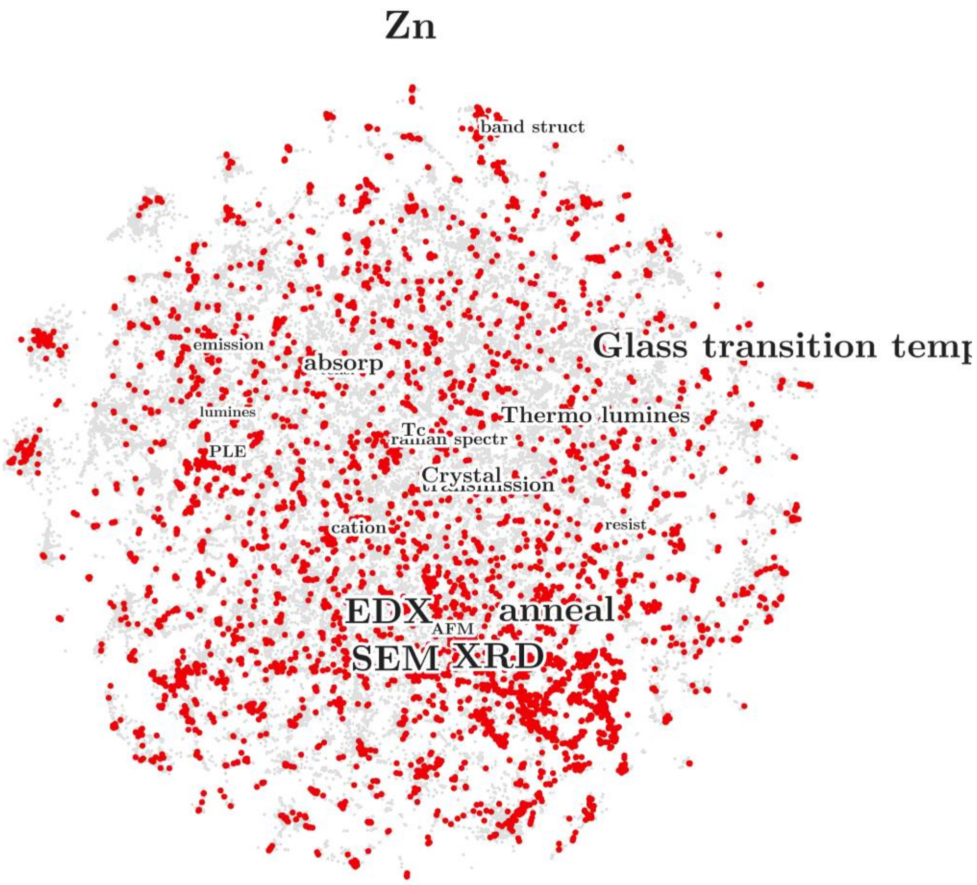

Figure S65. Latent Dirichlet Allocation plot for Zinc.

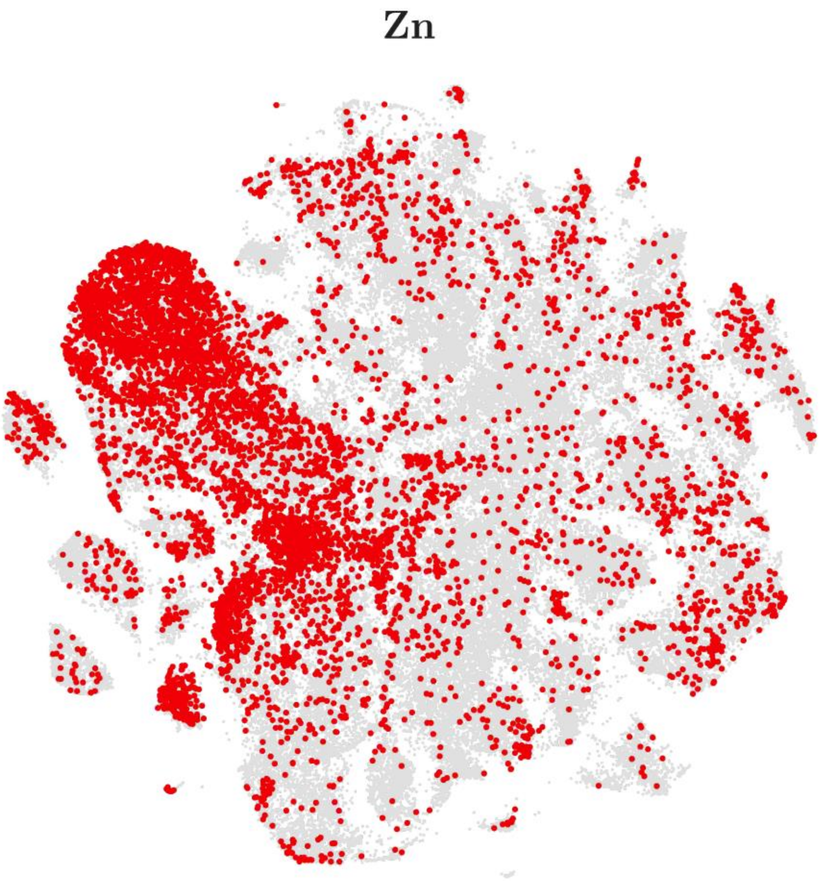

Figure S66. Caption Cluster plot for Gallium.

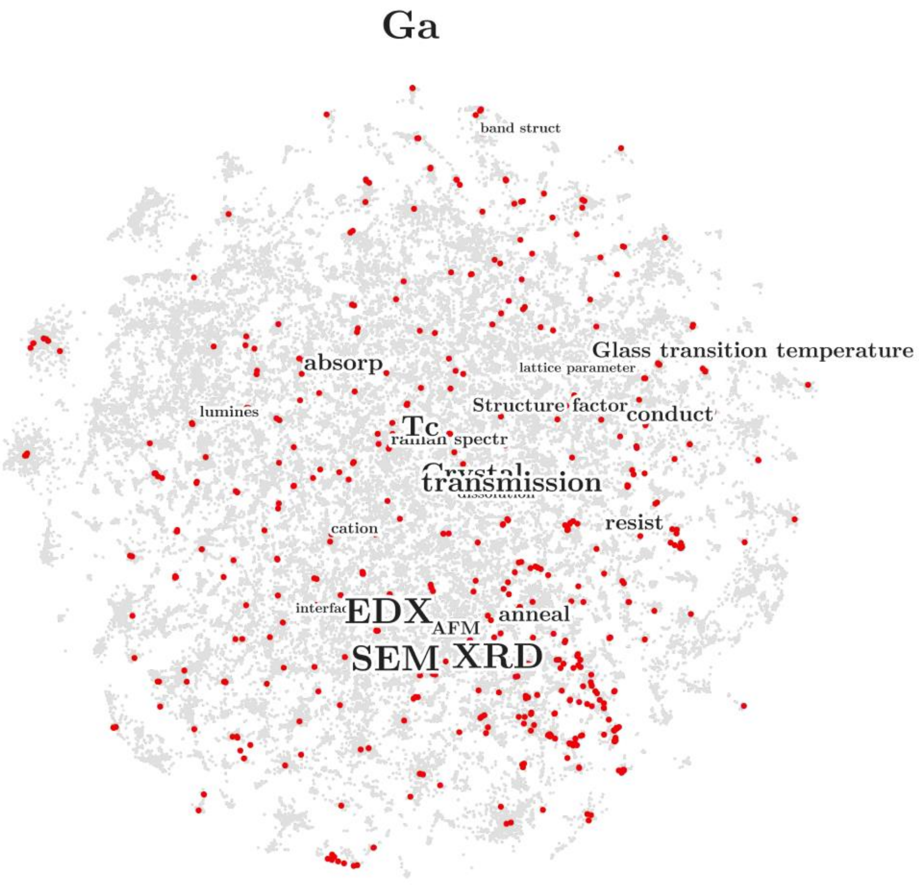

Figure S67. Latent Dirichlet Allocation plot for Gallium.

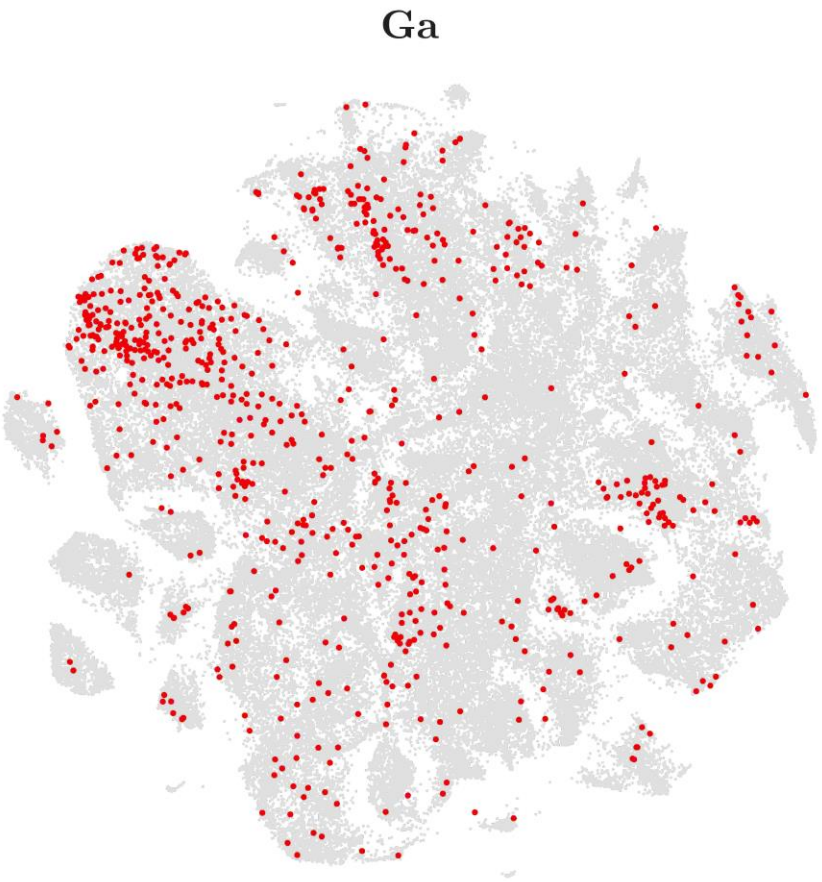

Figure S68. Caption Cluster plot for Germanium.

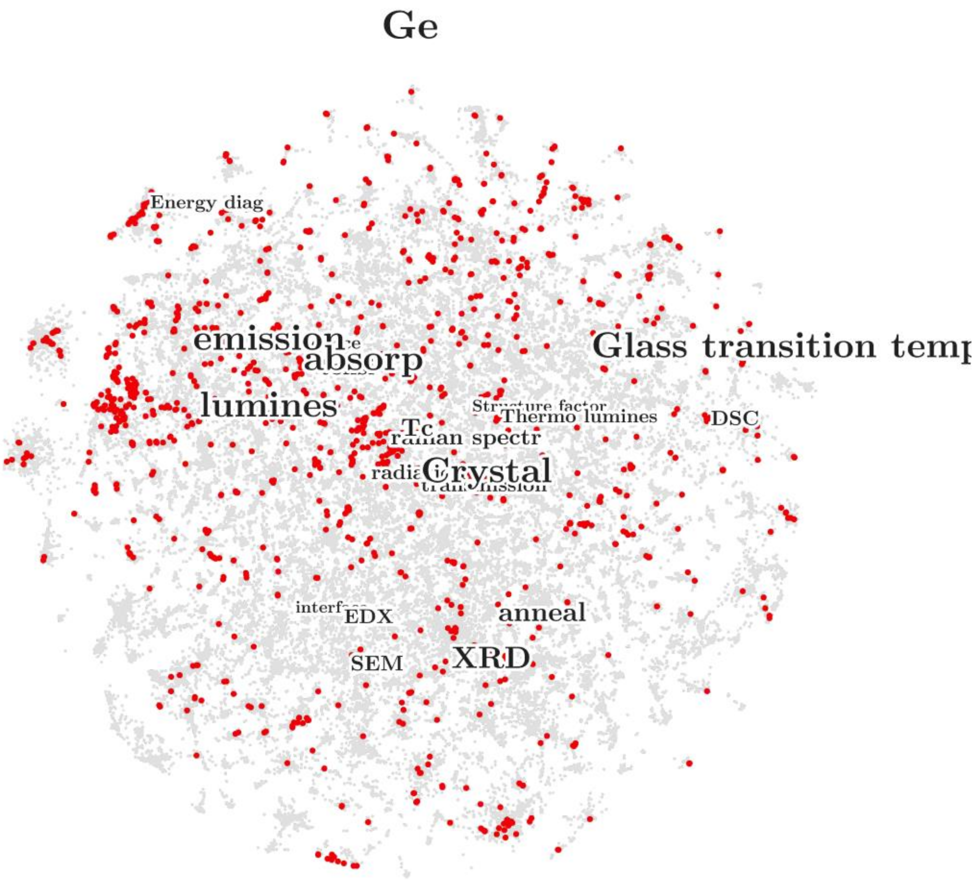

Figure S69. Latent Dirichlet Allocation plot for Germanium.

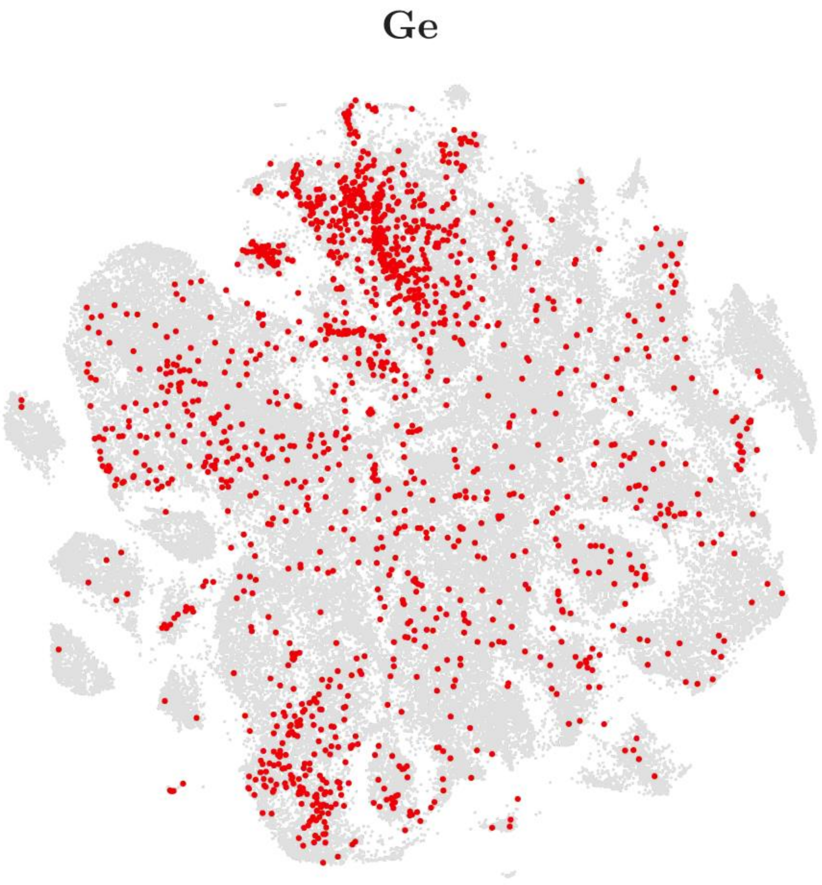

Figure S70. Caption Cluster plot for Arsenic.

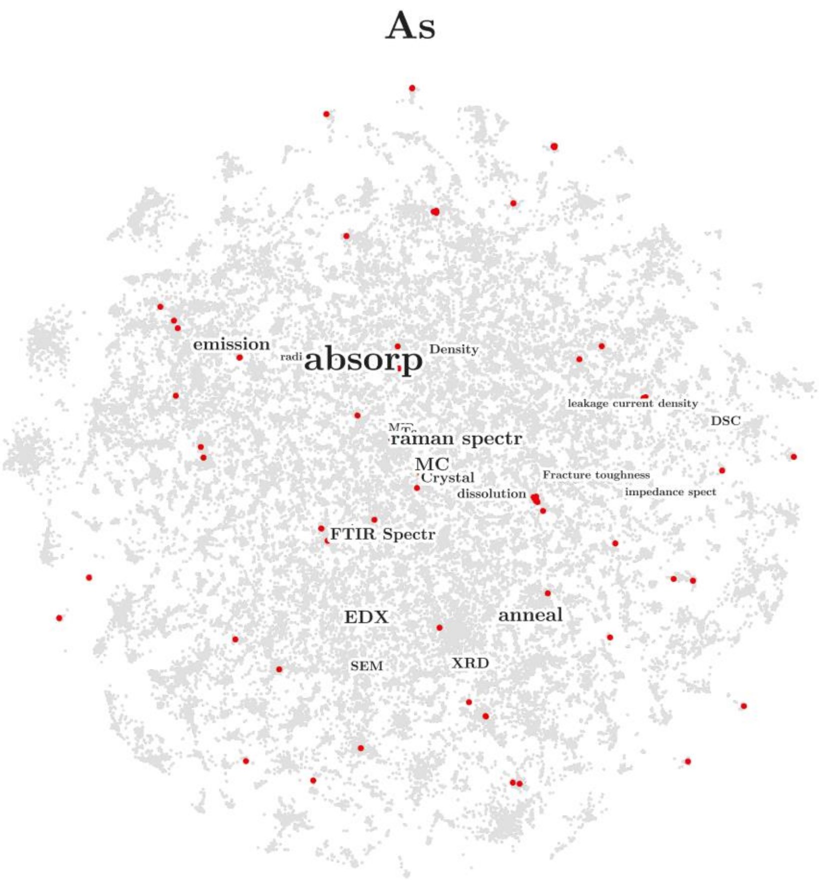

Figure S71. Latent Dirichlet Allocation plot for Arsenic.

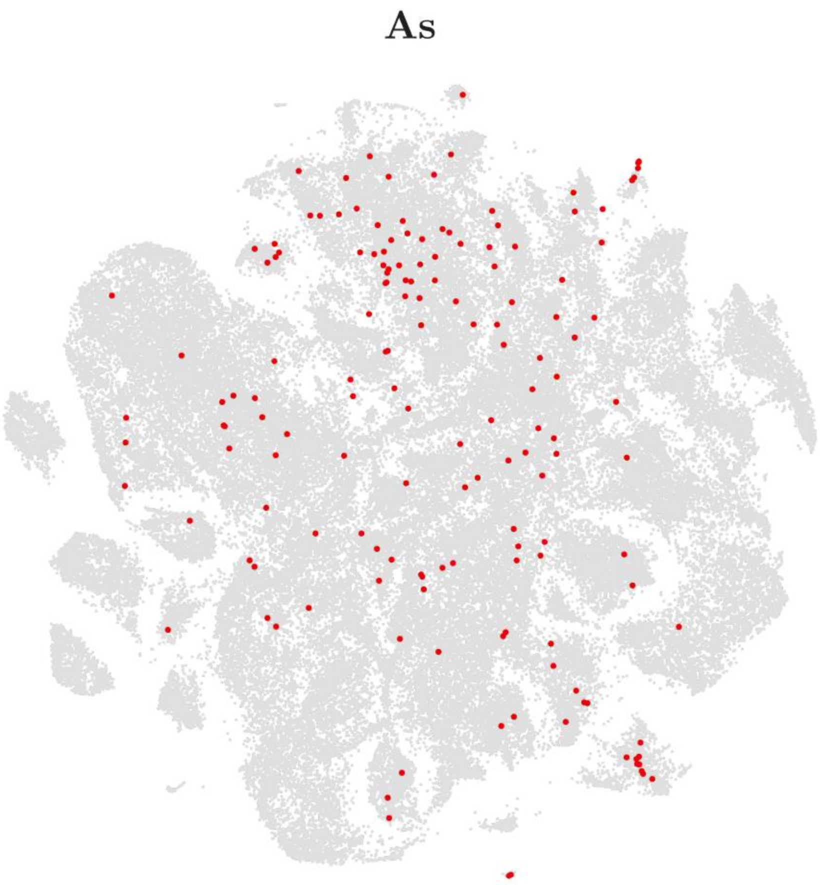

Figure S72. Caption Cluster plot for Selenium.

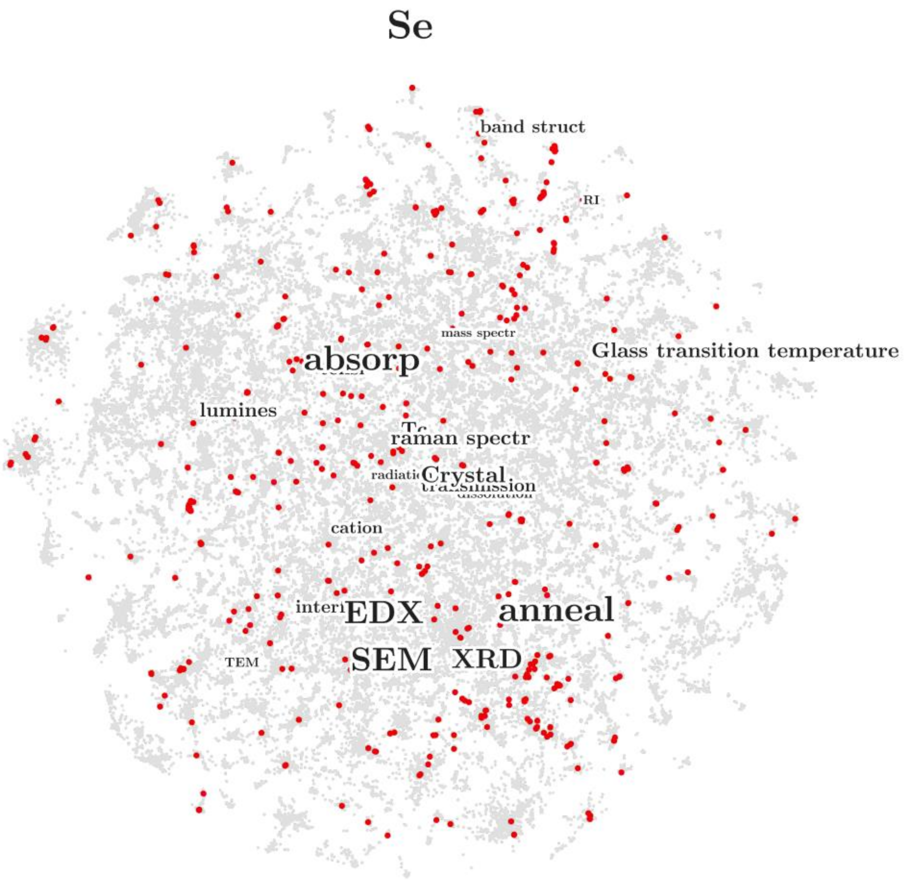

Figure S73. Latent Dirichlet Allocation plot for Selenium.

.

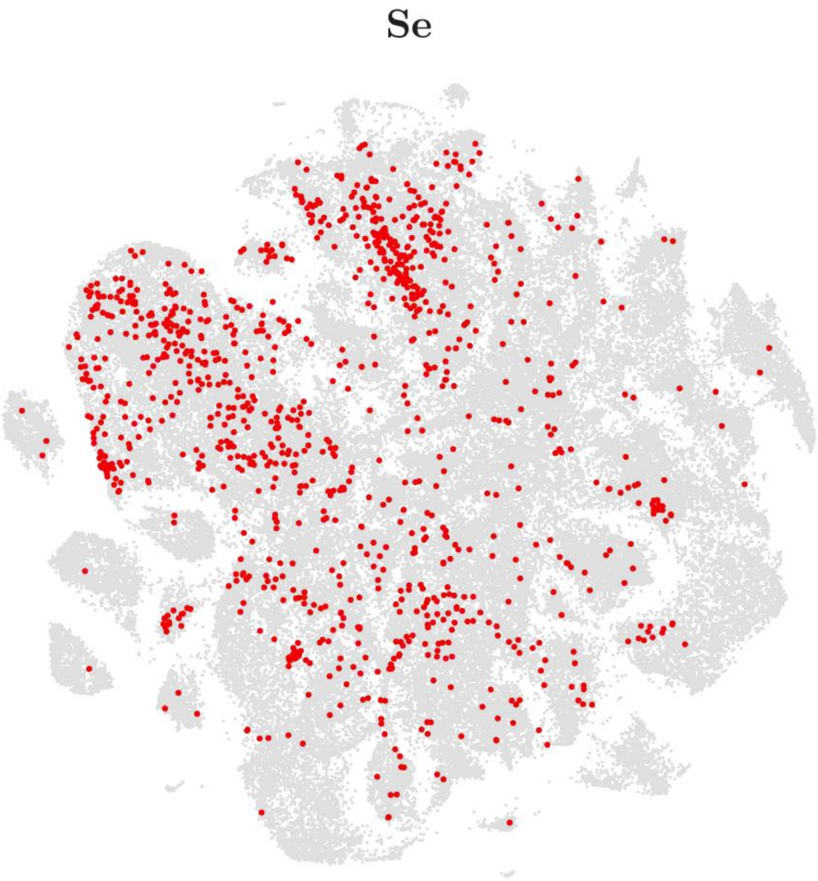

Figure S74. Caption Cluster plot for Bromine.

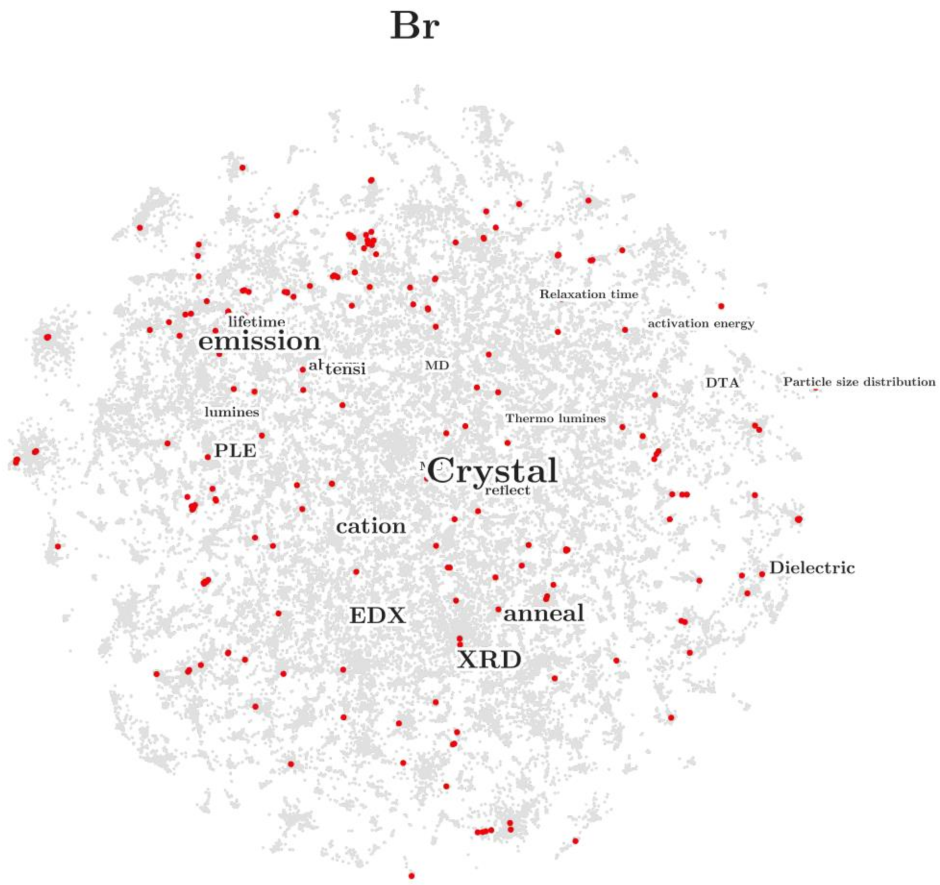

Figure S75. Latent Dirichlet Allocation plot for Bromine.

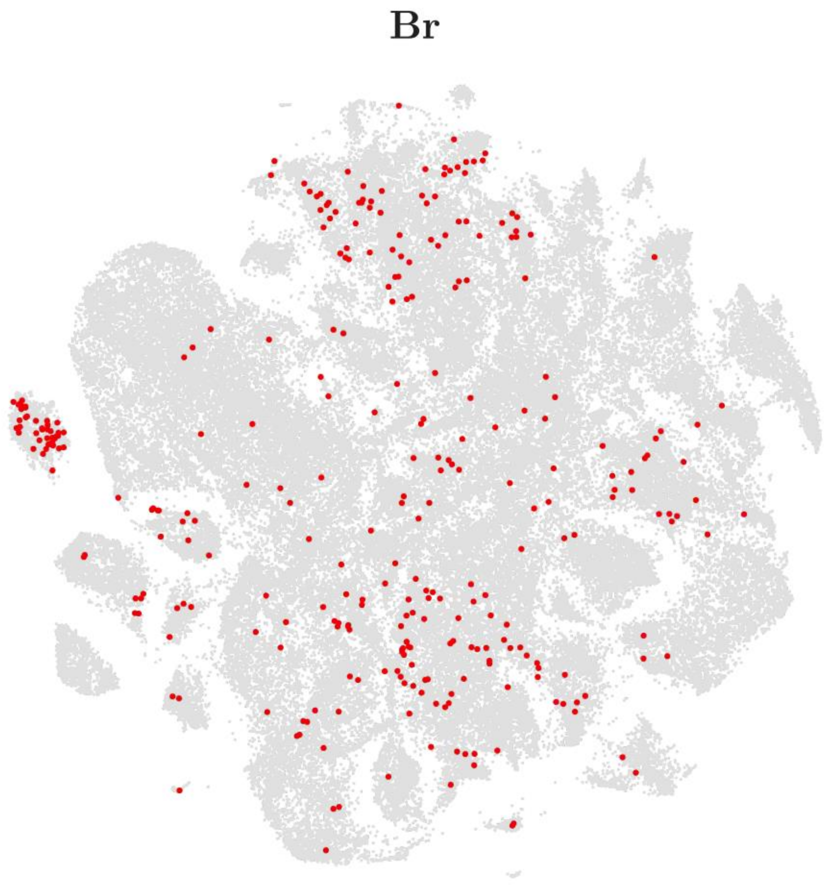

Figure S76. Caption Cluster plot for Krypton.

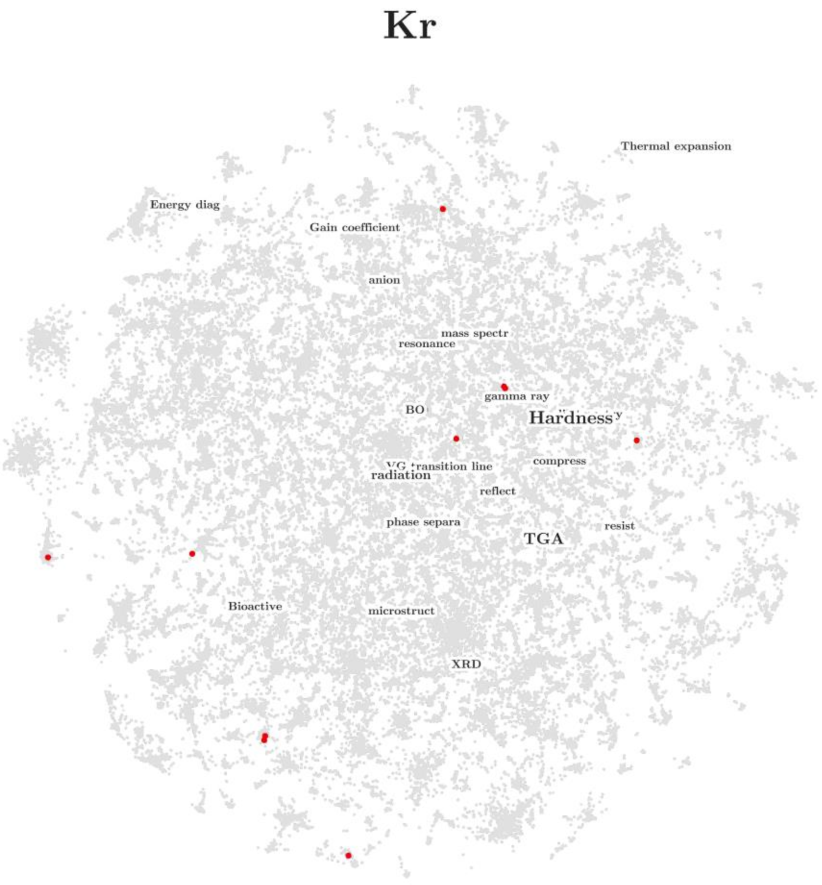

Figure S77. Latent Dirichlet Allocation plot for Krypton.

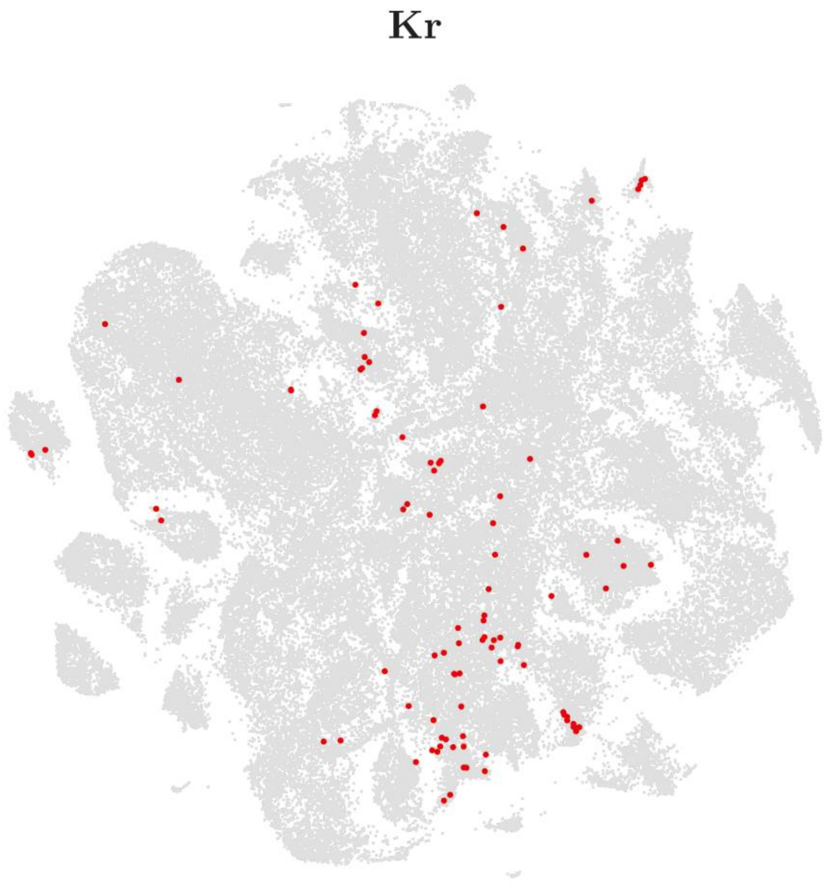

Figure S78. Caption Cluster plot for Rubidium.

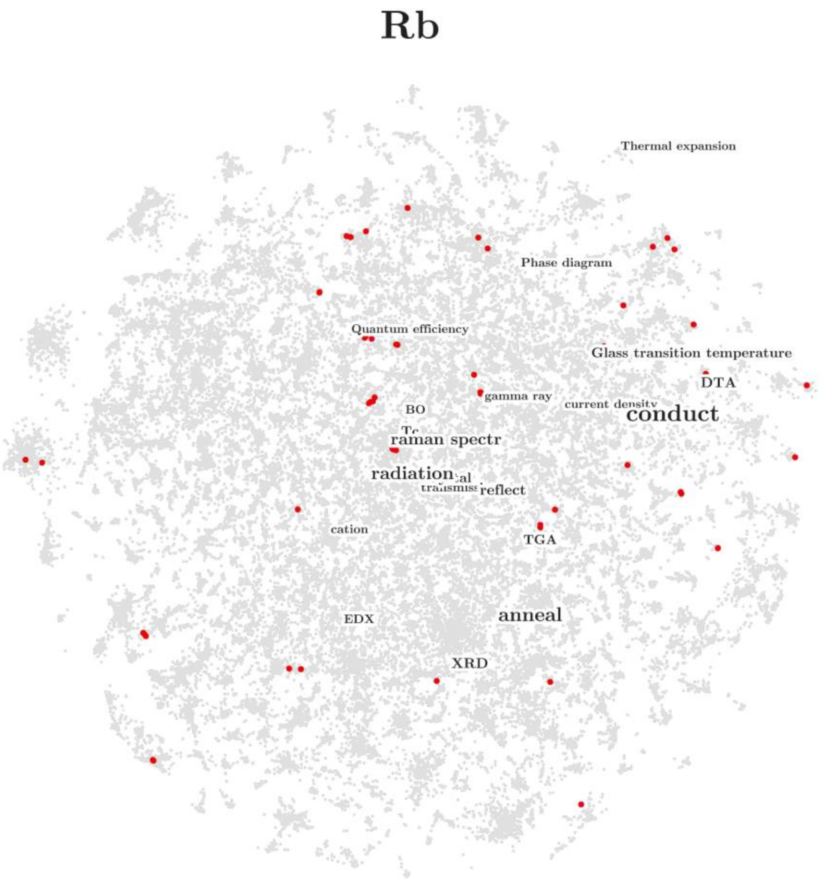

Figure S79. Latent Dirichlet Allocation plot for Rubidium.

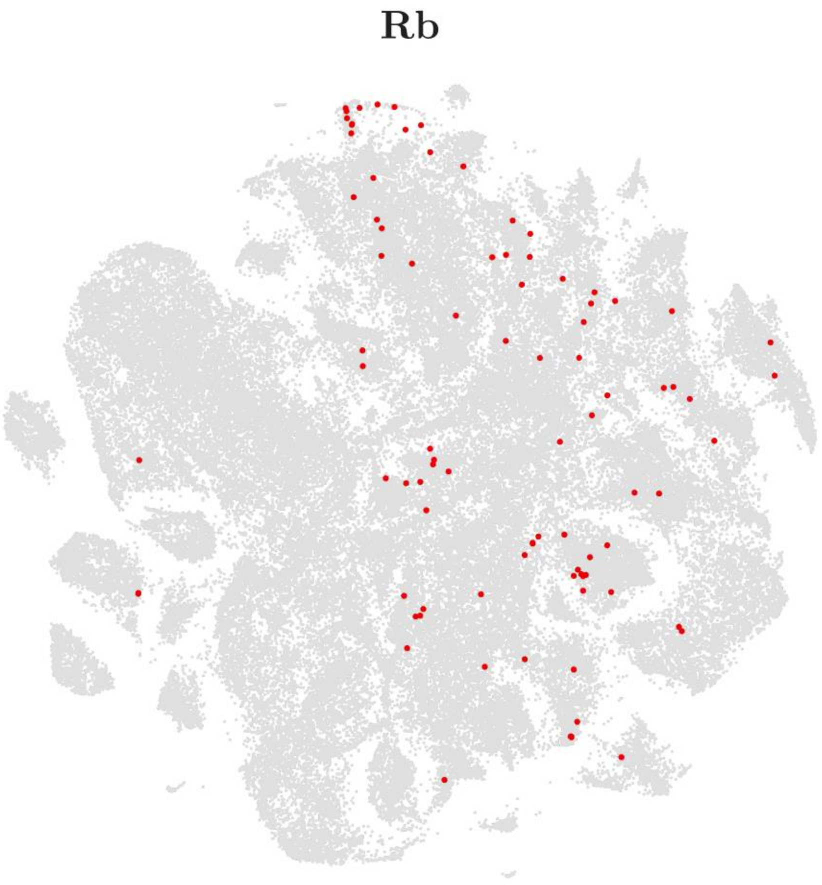

Figure S80. Caption Cluster plot for Strontium.

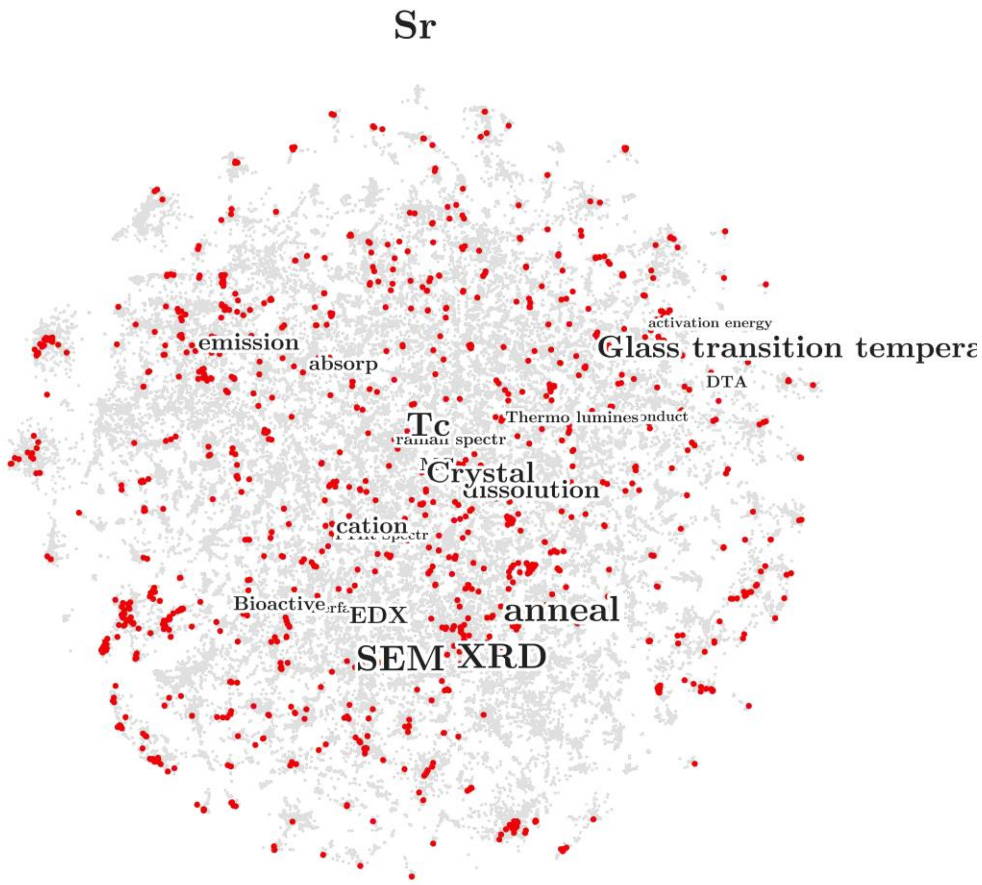

Figure S81. Latent Dirichlet Allocation plot for Strontium.

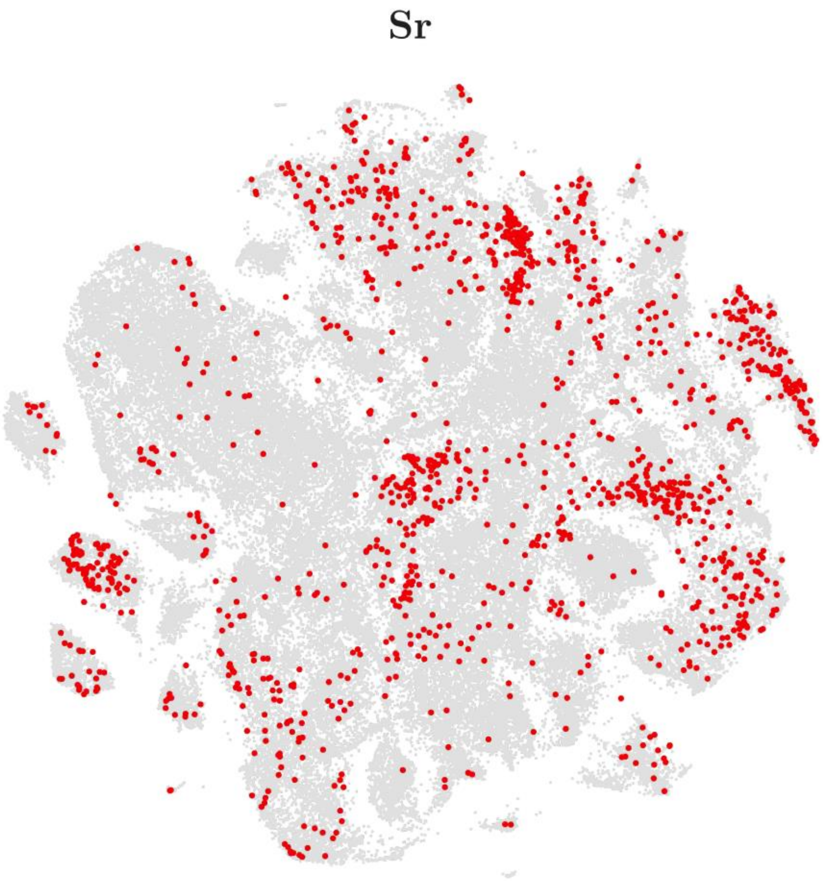

Figure S82. Caption Cluster plot for Yttrium.

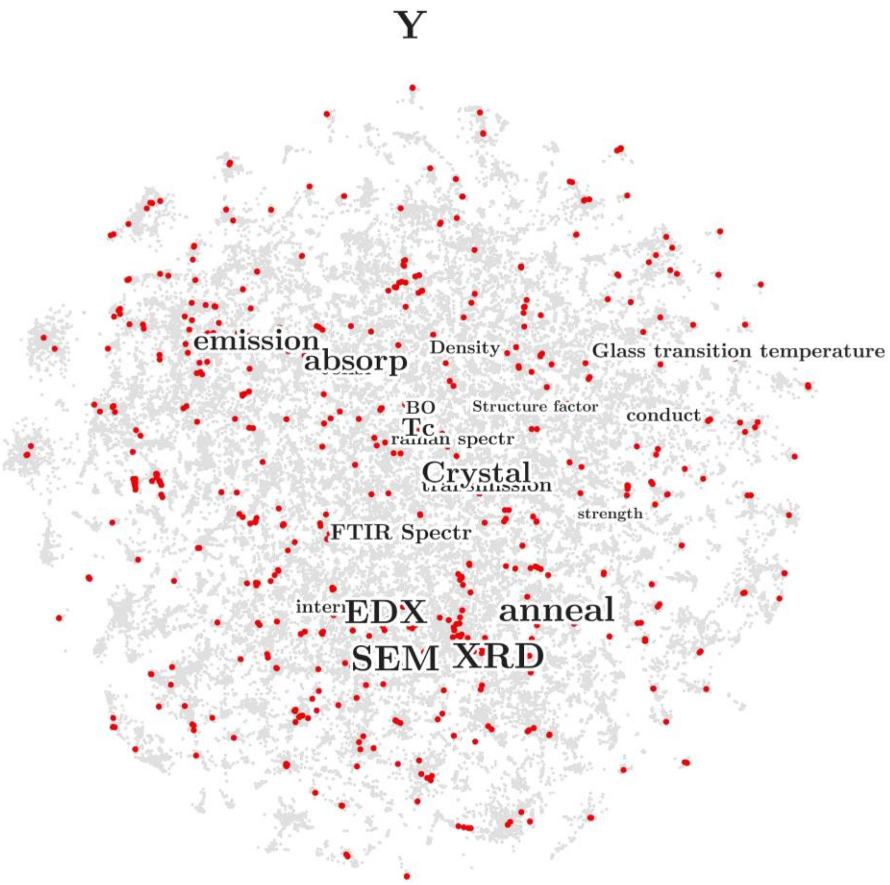

Figure S83. Latent Dirichlet Allocation plot for Yttrium.

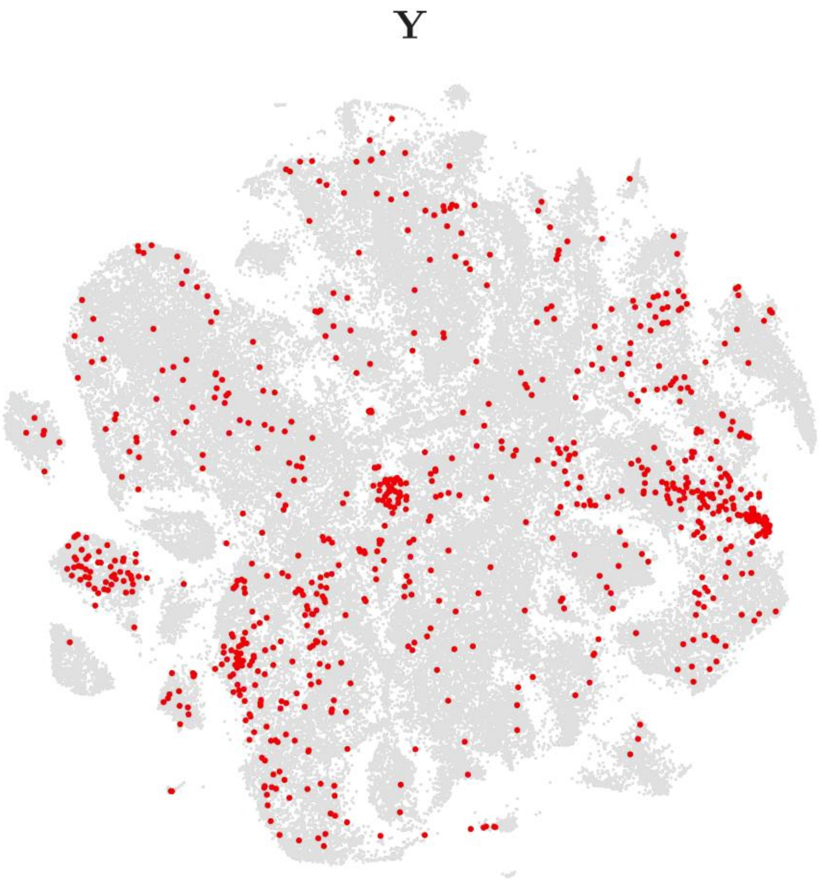

Figure S84. Caption Cluster plot for Zirconium.

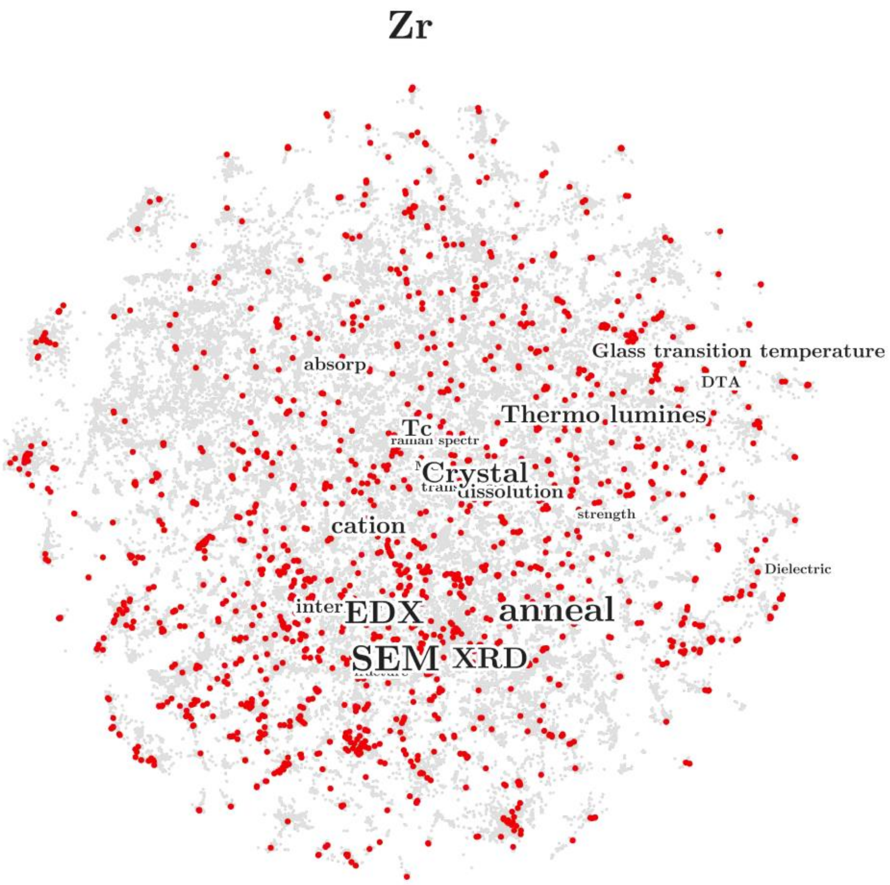

Figure S85. Latent Dirichlet Allocation plot for Zirconium.

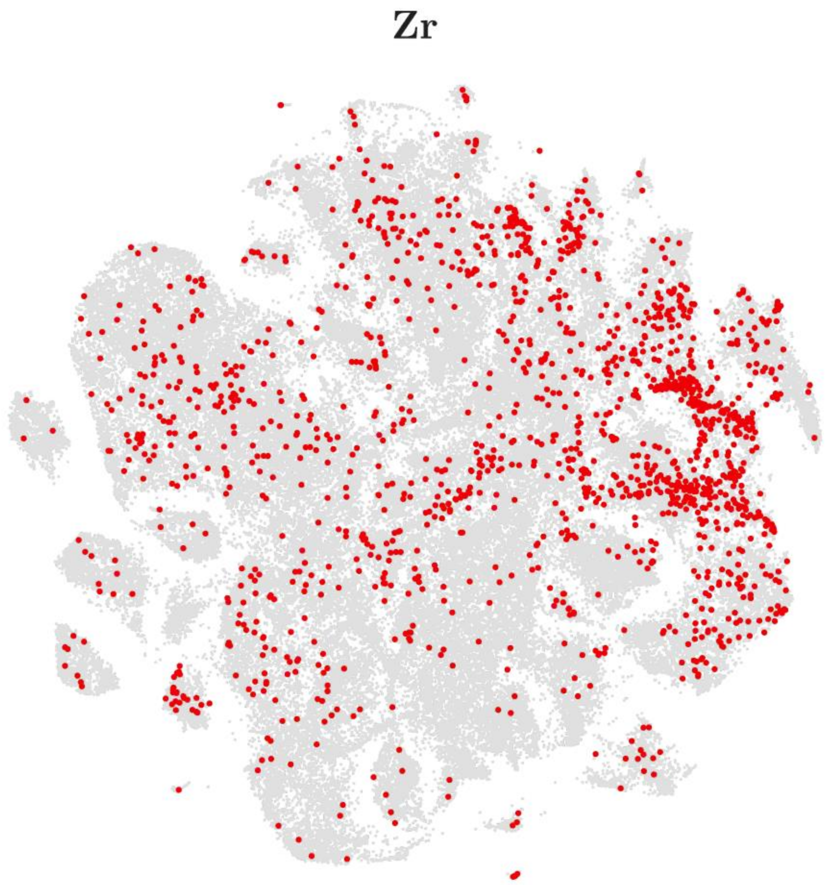

Figure S86. Caption Cluster plot for Niobium.

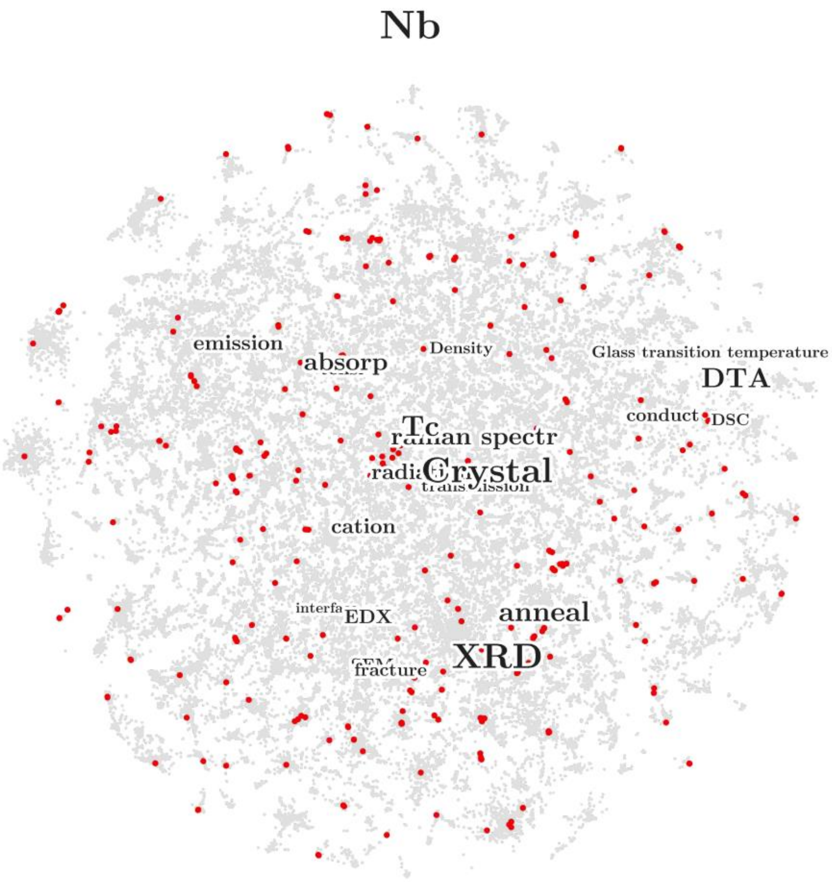

Figure S87. Latent Dirichlet Allocation plot for Niobium.

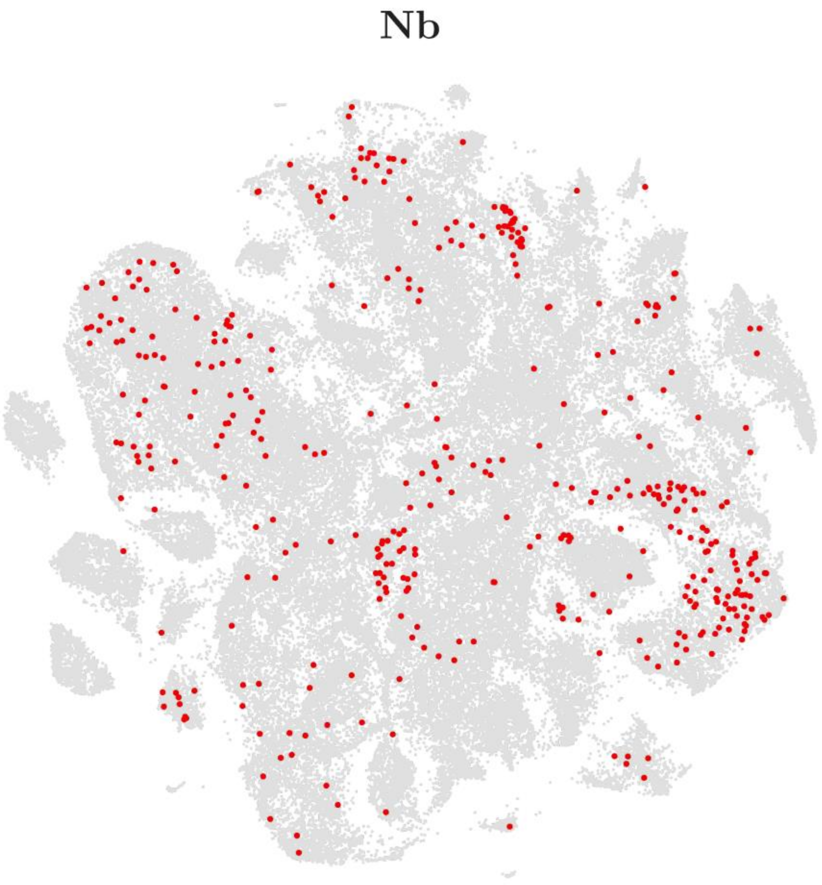

Figure S88. Caption Cluster plot for Molybdenum.

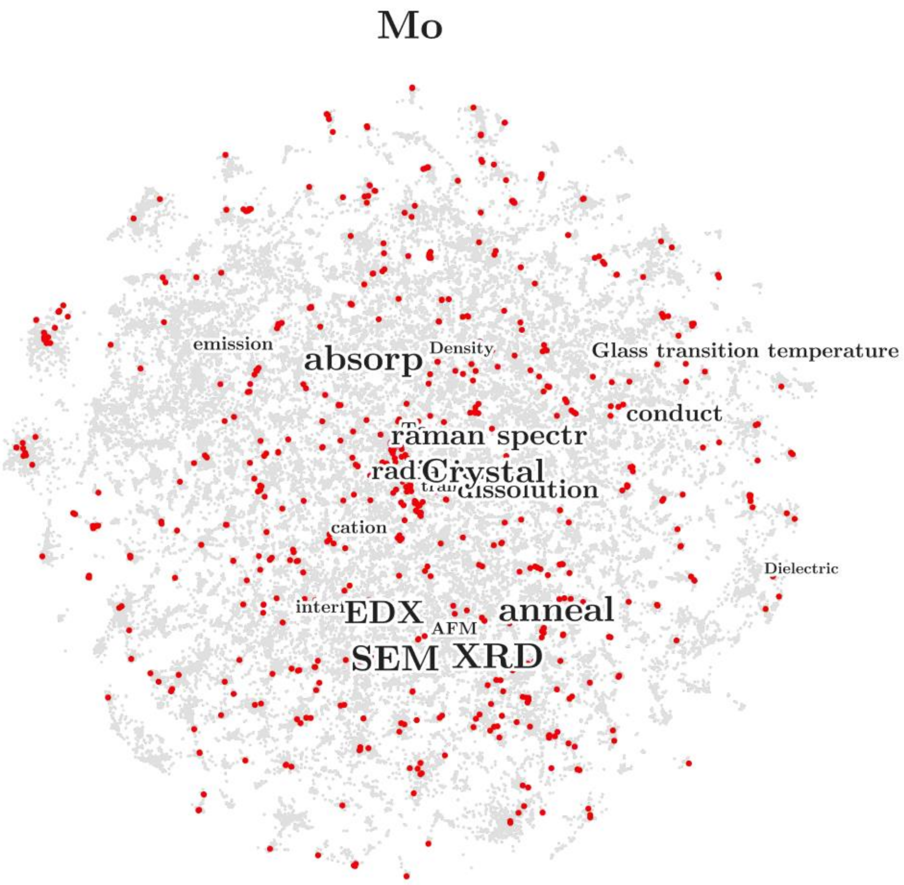

Figure S89. Latent Dirichlet Allocation plot for Molybdenum.

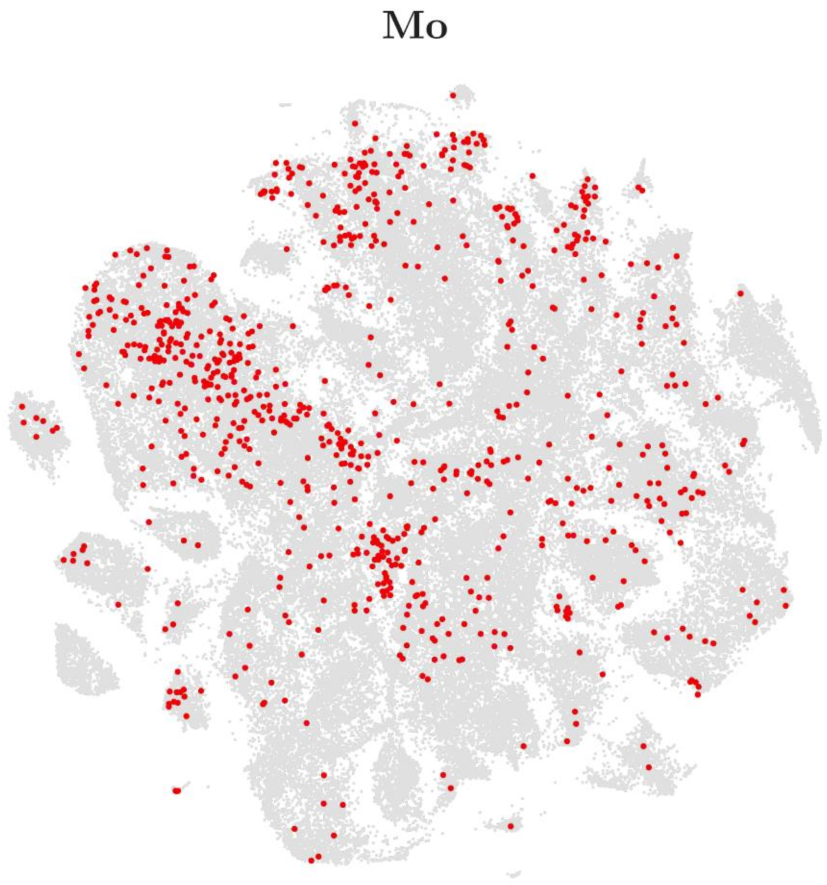

Figure S90. Caption Cluster plot for Technetium.

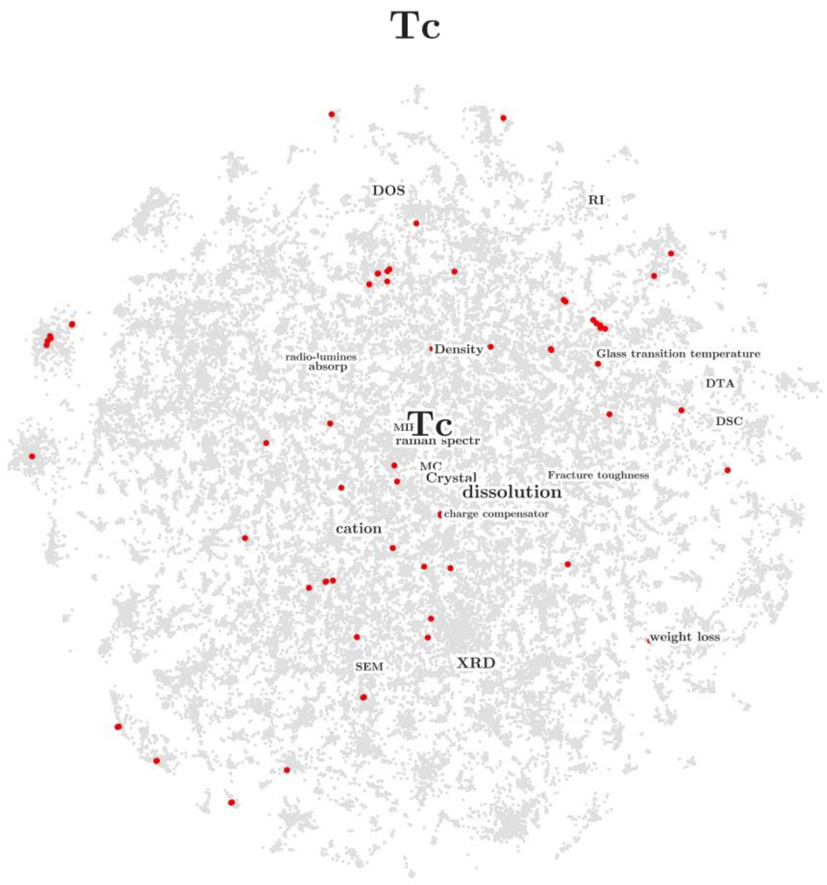

Figure S91. Latent Dirichlet Allocation plot for Technetium.

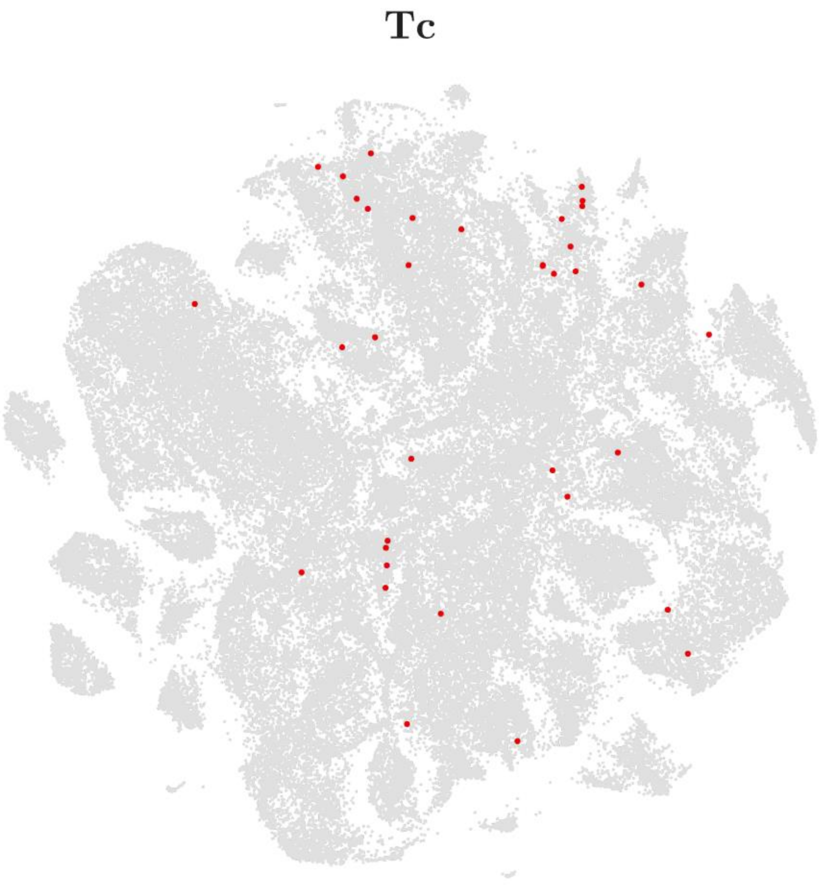

Figure S92. Caption Cluster plot for Ruthenium.

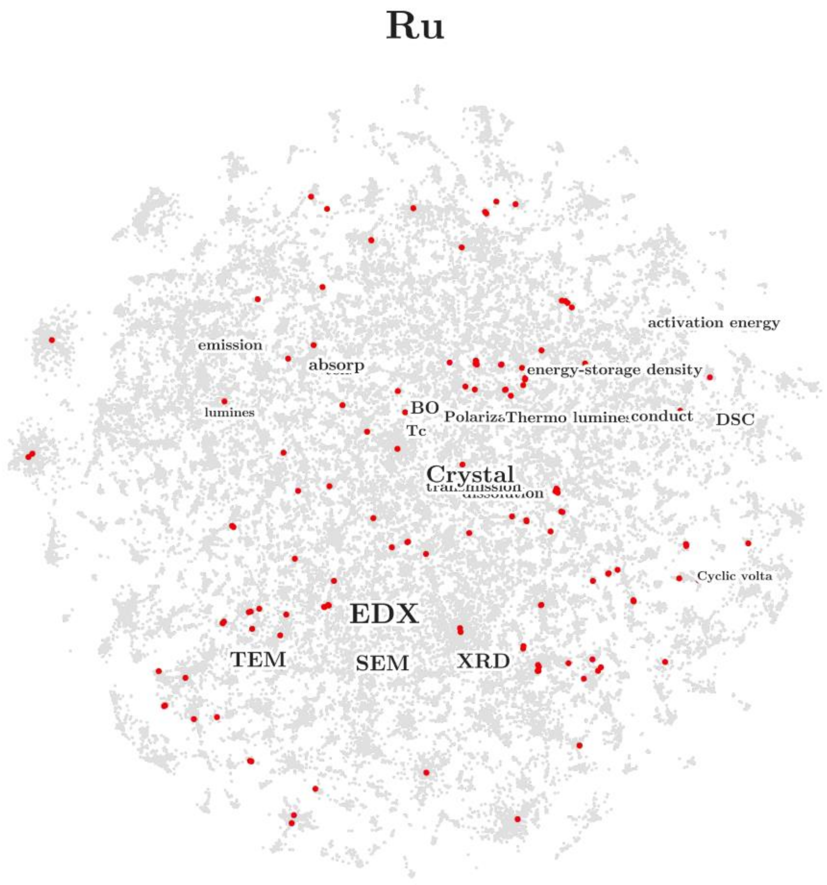

Figure S93. Latent Dirichlet Allocation plot for Ruthenium.

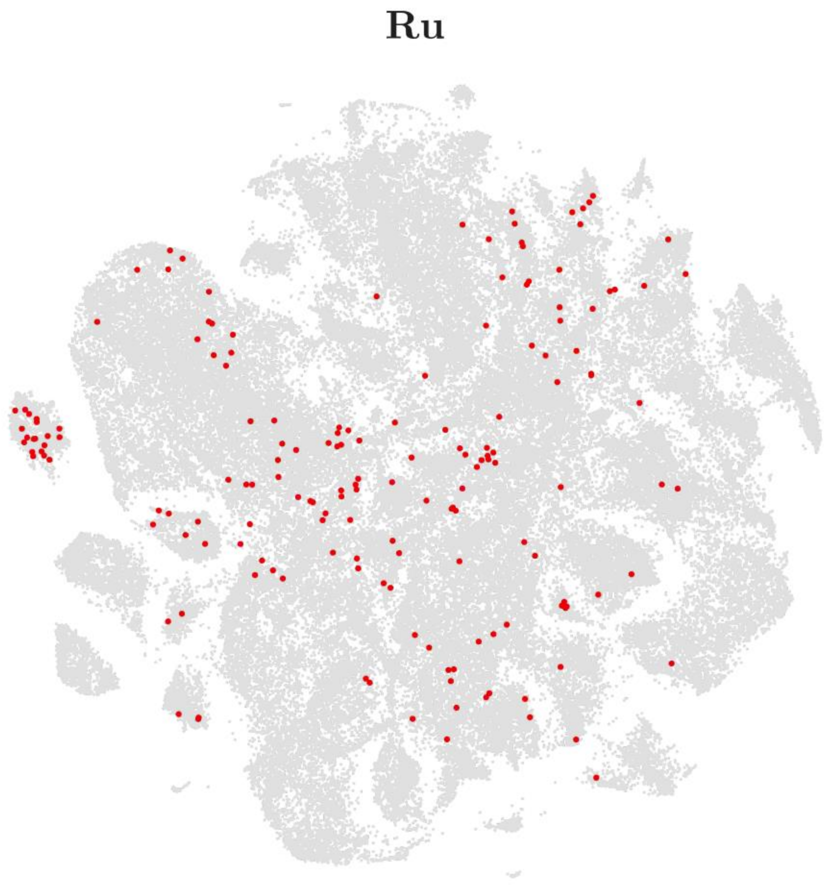

Figure S94. Caption Cluster plot for Rhodium.

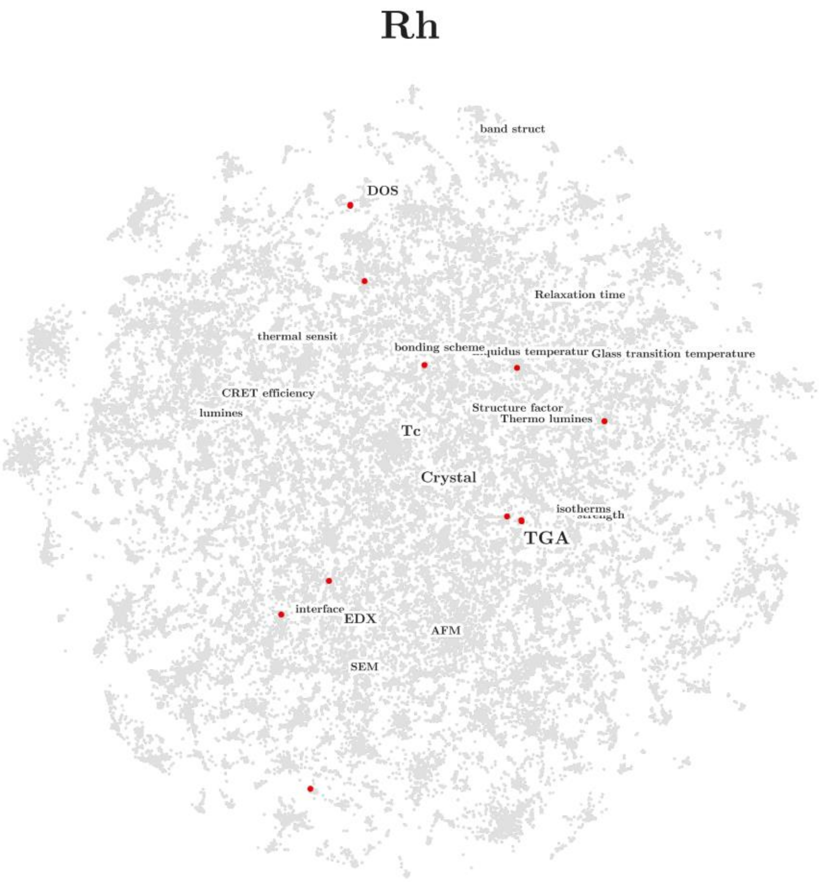

Figure S95. Latent Dirichlet Allocation plot for Rhodium.

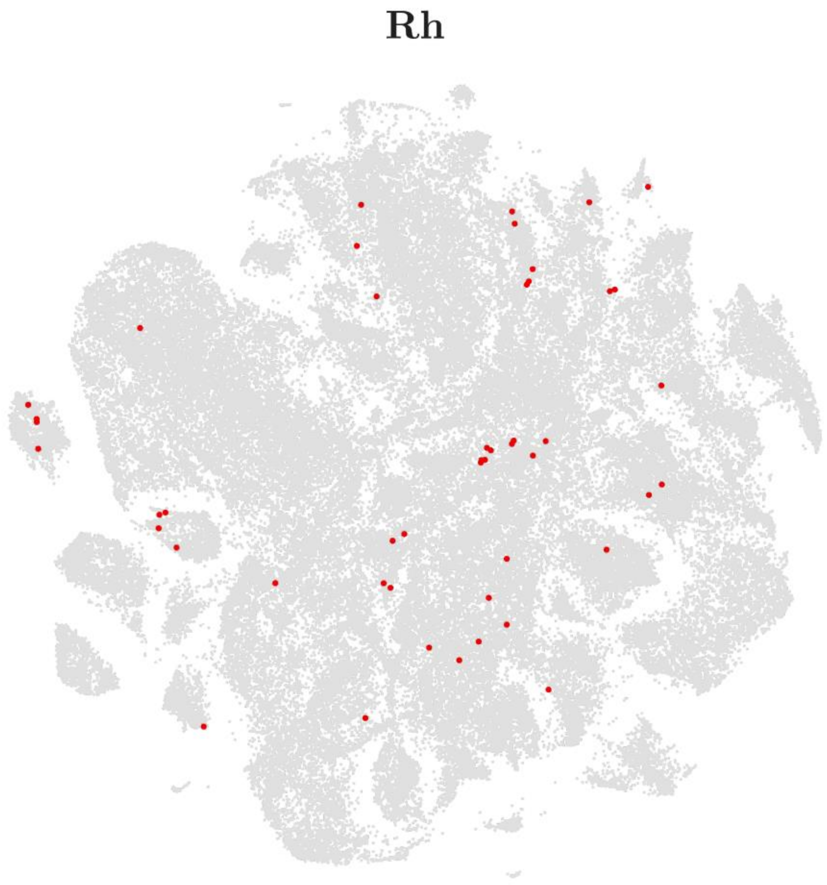

Figure S96. Caption Cluster plot for Palladium.

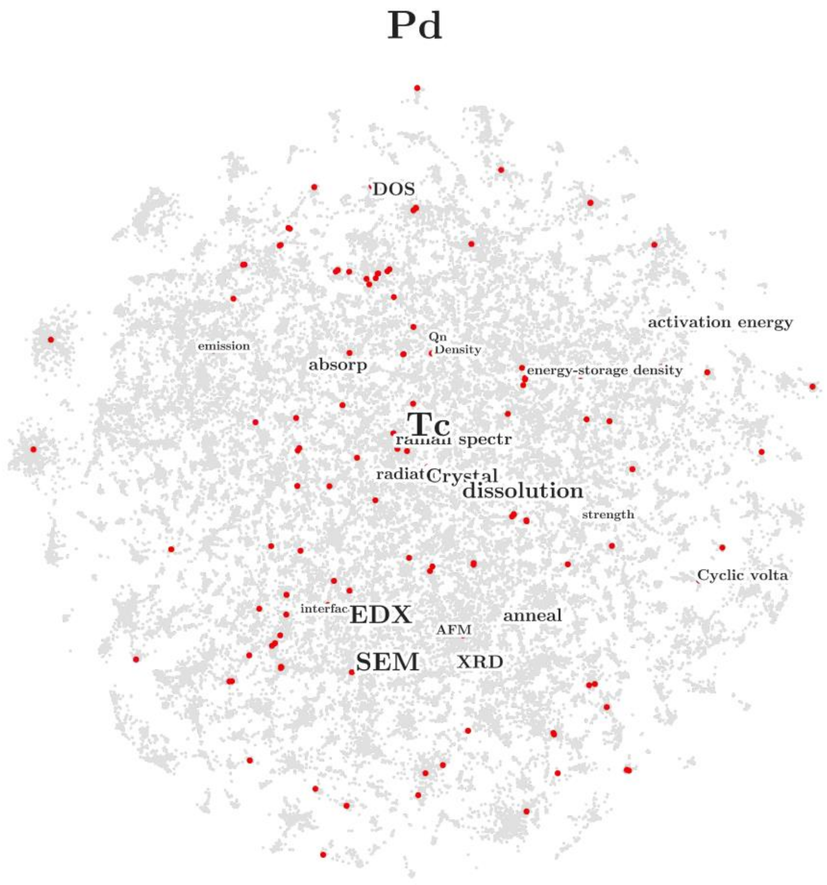

Figure S97. Latent Dirichlet Allocation plot for Palladium.

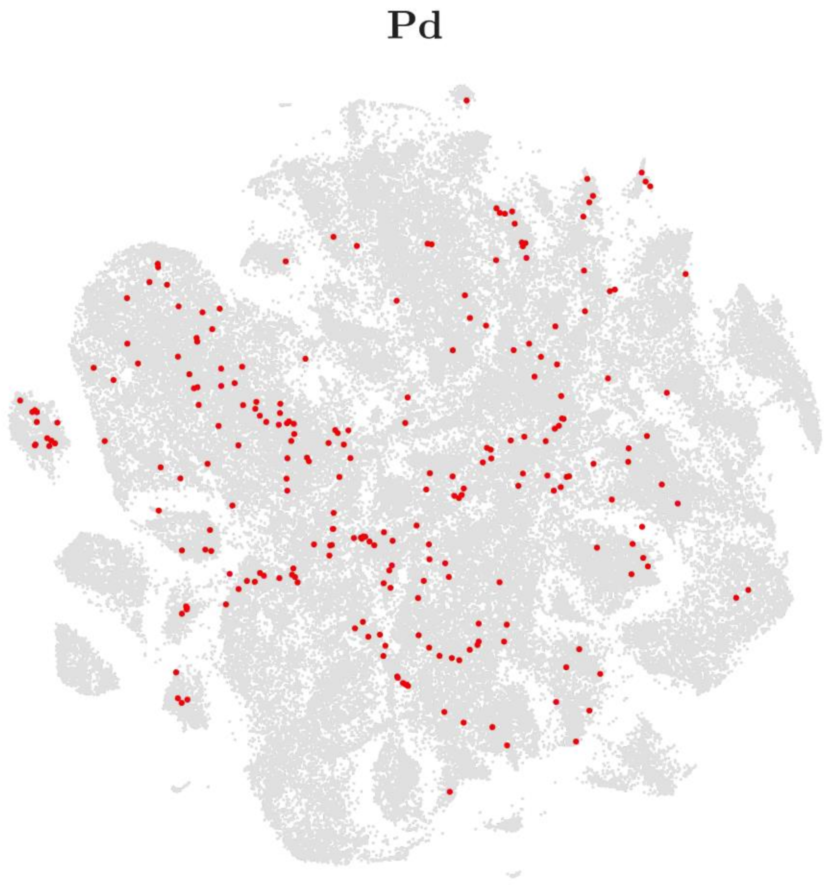

Figure S98. Caption Cluster plot for Silver.

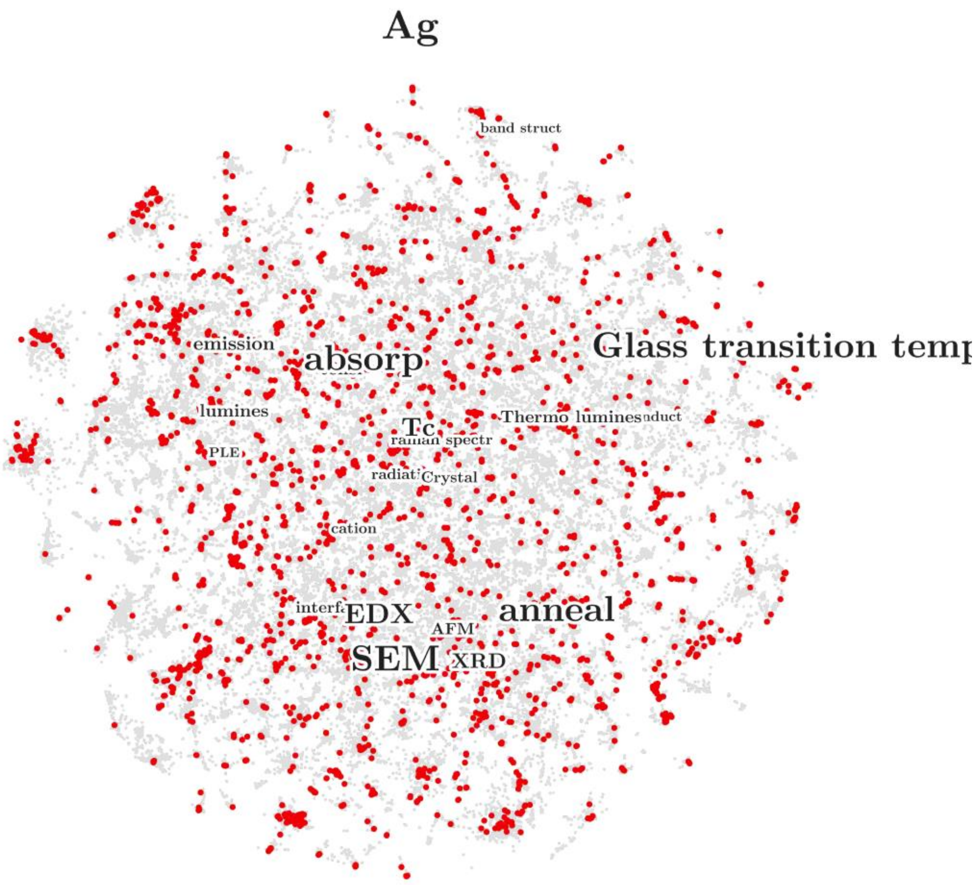

Figure S99. Latent Dirichlet Allocation plot for Silver.

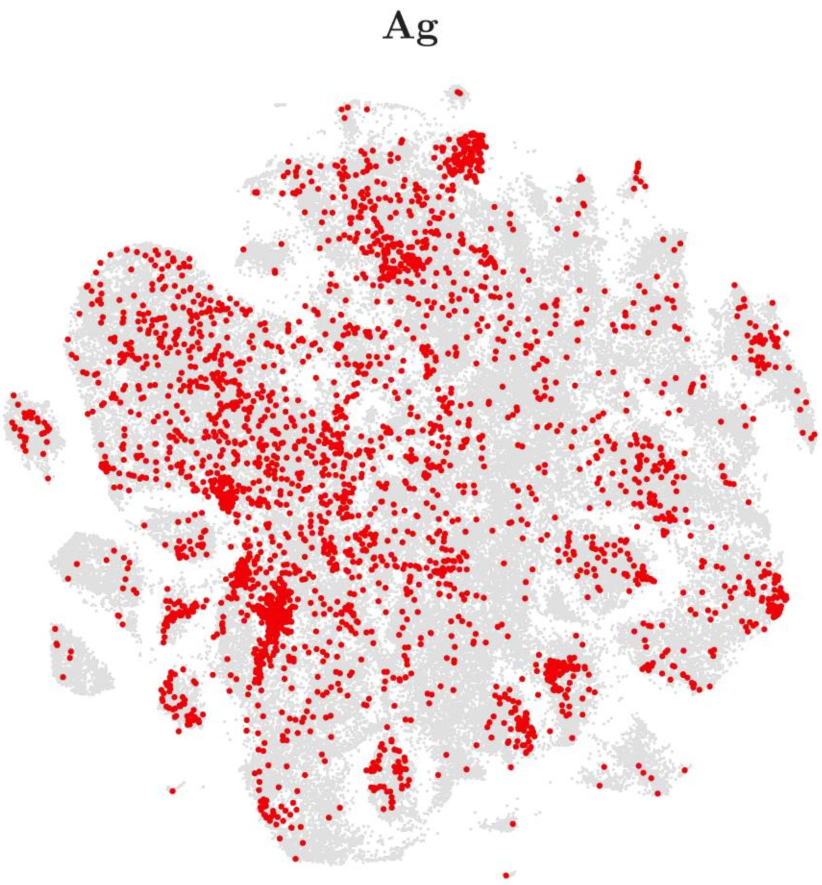

Figure S100. Caption Cluster plot for Cadmium.

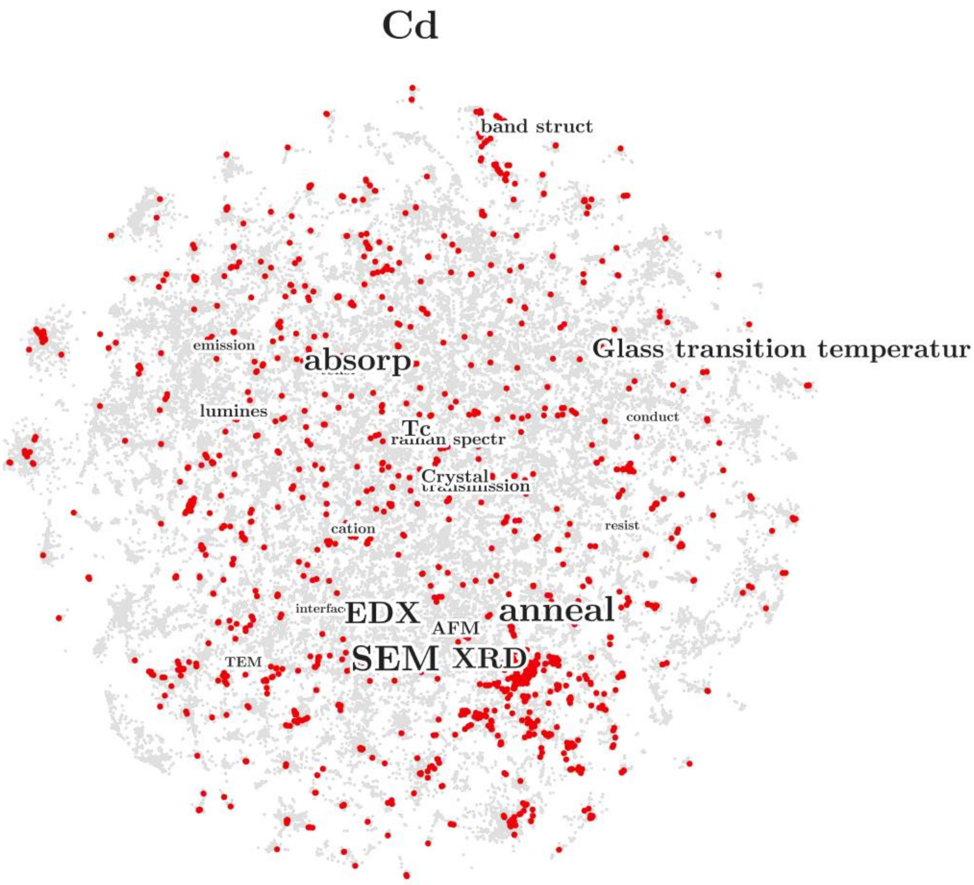

Figure S101. Latent Dirichlet Allocation plot for Cadmium.

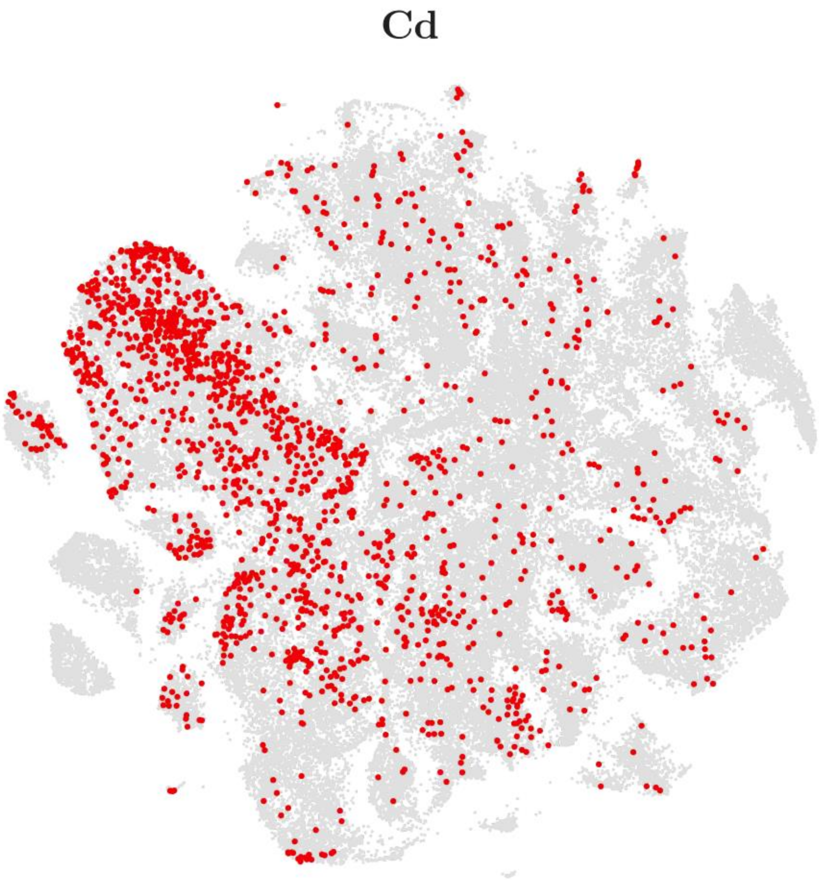

Figure S102. Caption Cluster plot for Indium.

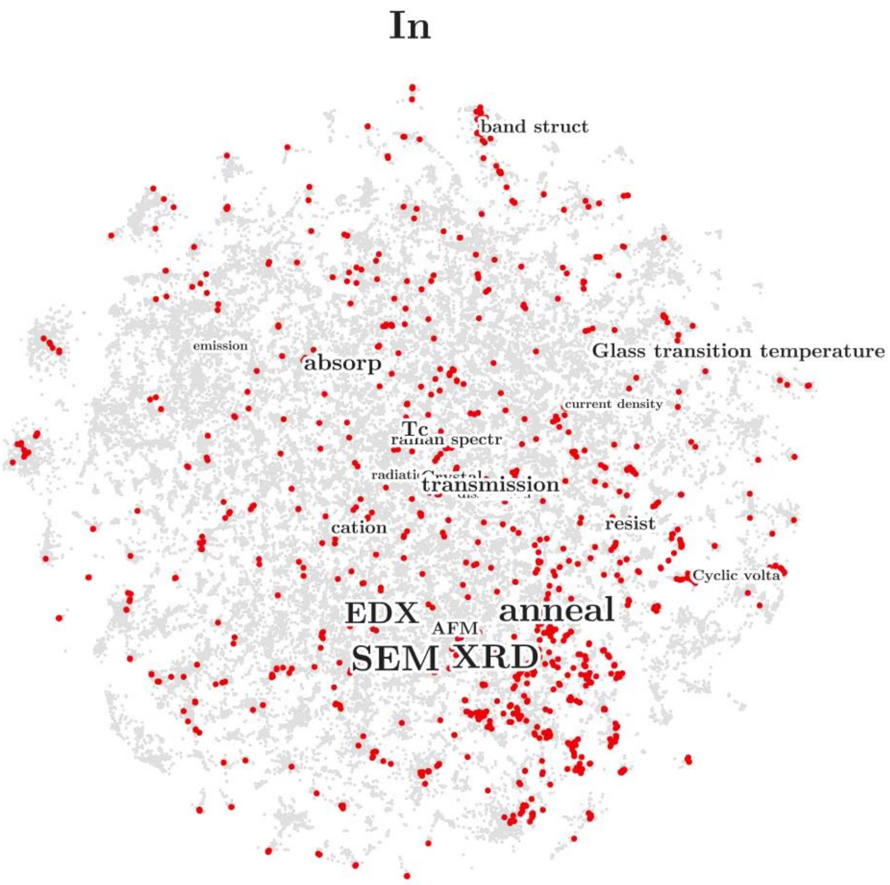

Figure S103. Latent Dirichlet Allocation plot for Indium.

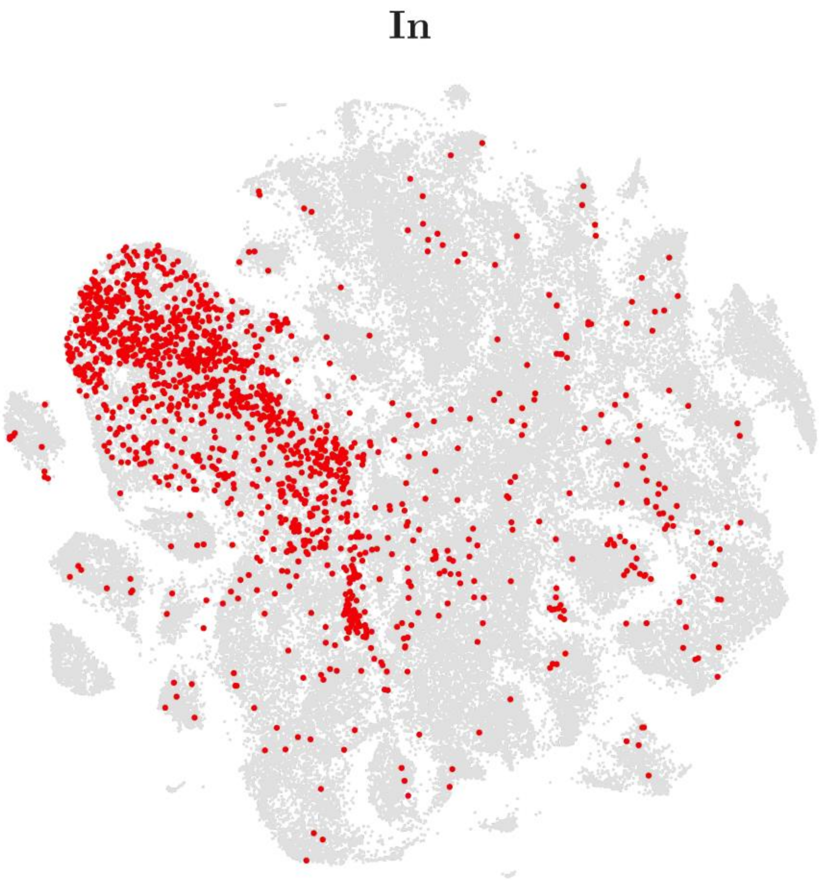

Figure S104. Caption Cluster plot for Tin.

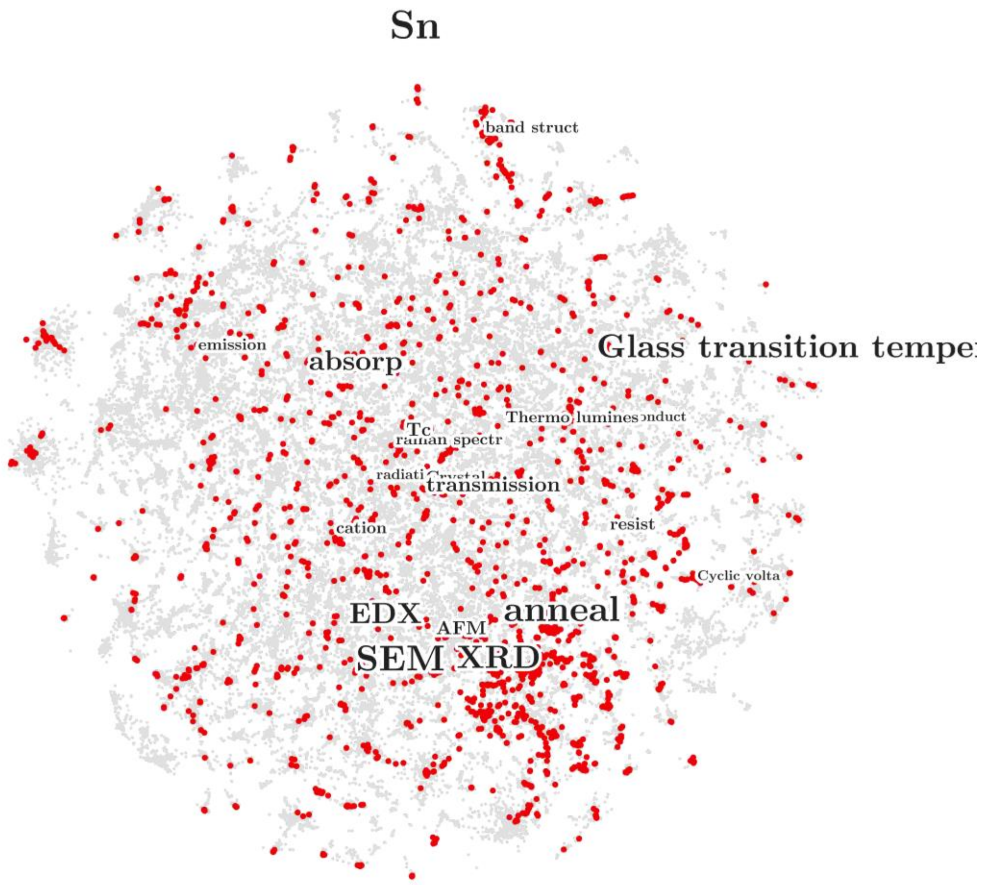

Figure S105. Latent Dirichlet Allocation plot for Tin.

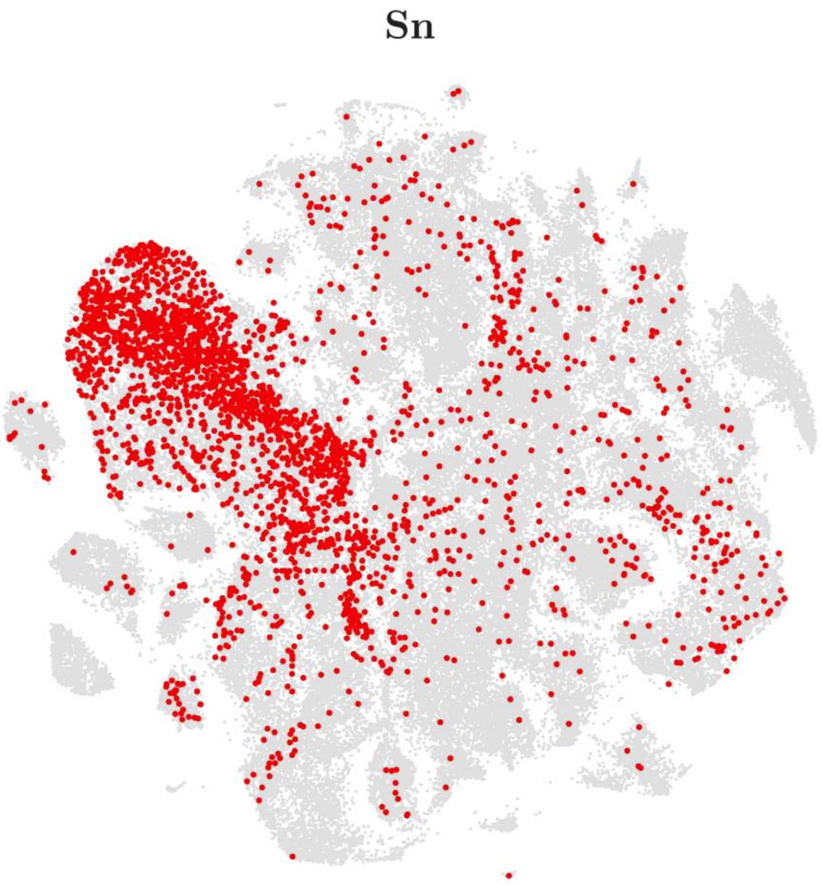

Figure S106. Caption Cluster plot for Antimony.

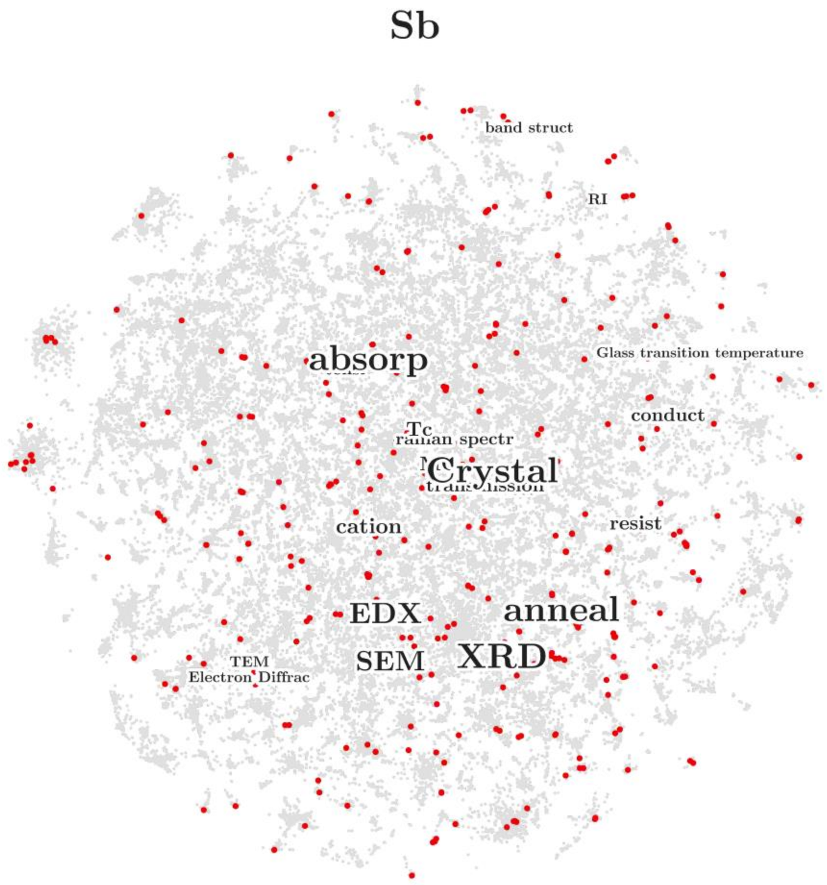

Figure S107. Latent Dirichlet Allocation plot for Antimony.

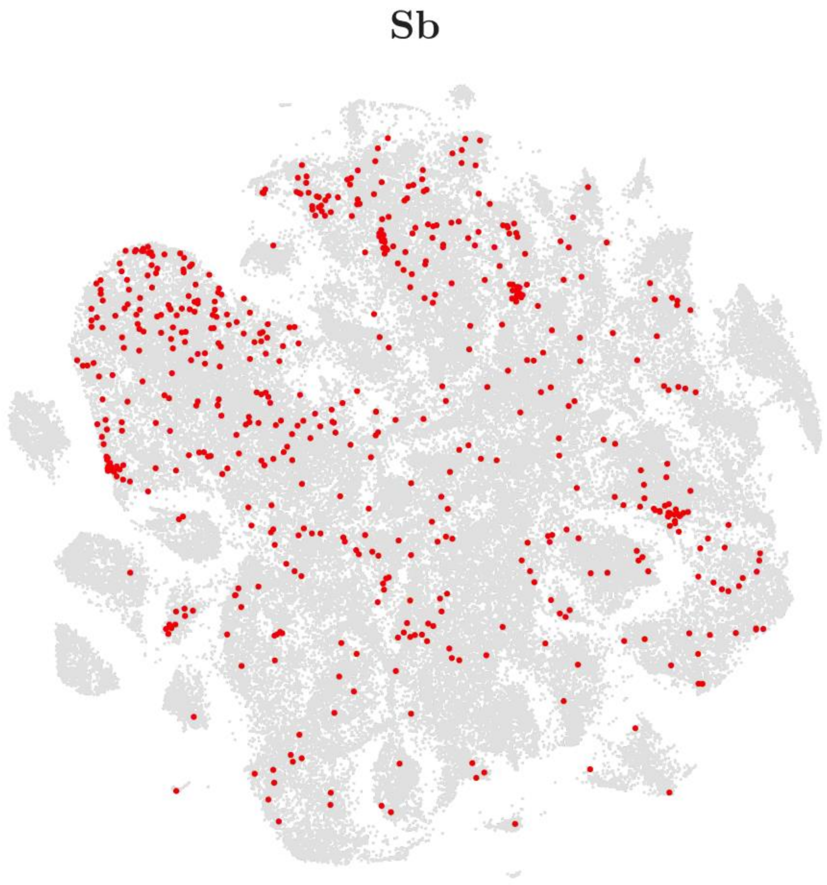

Figure S108. Caption Cluster plot for Tellurium.

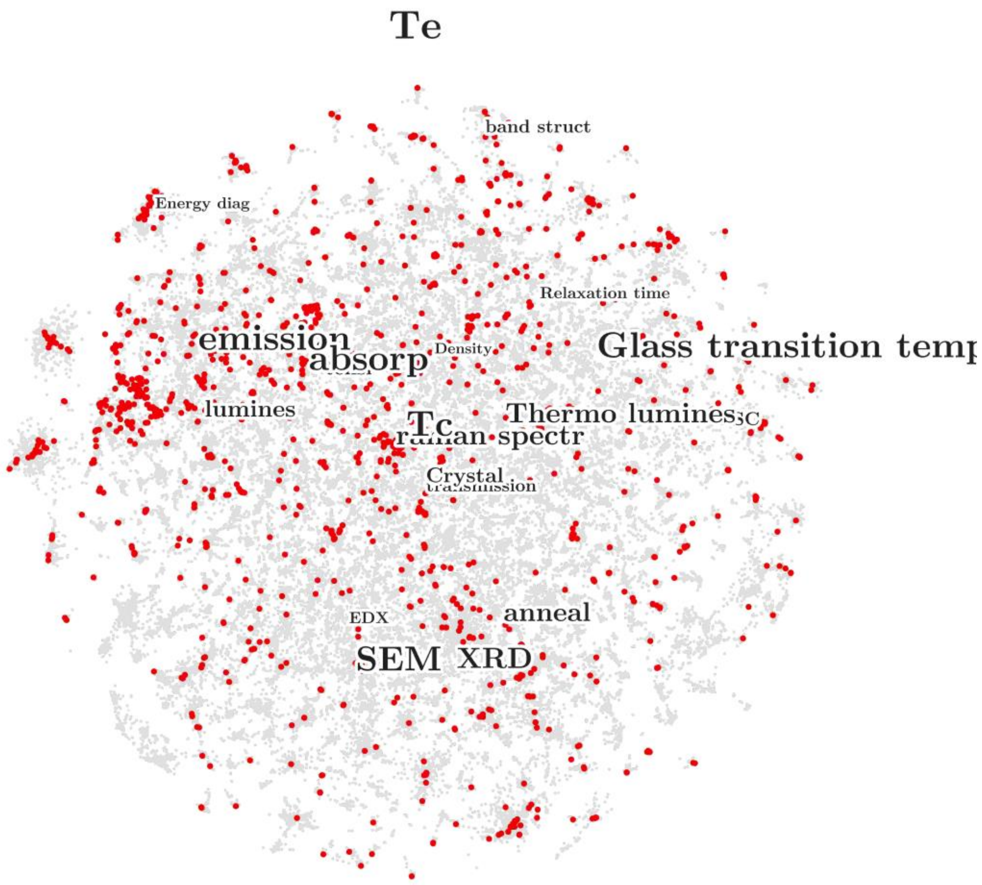

Figure S109. Latent Dirichlet Allocation plot for Tellurium.

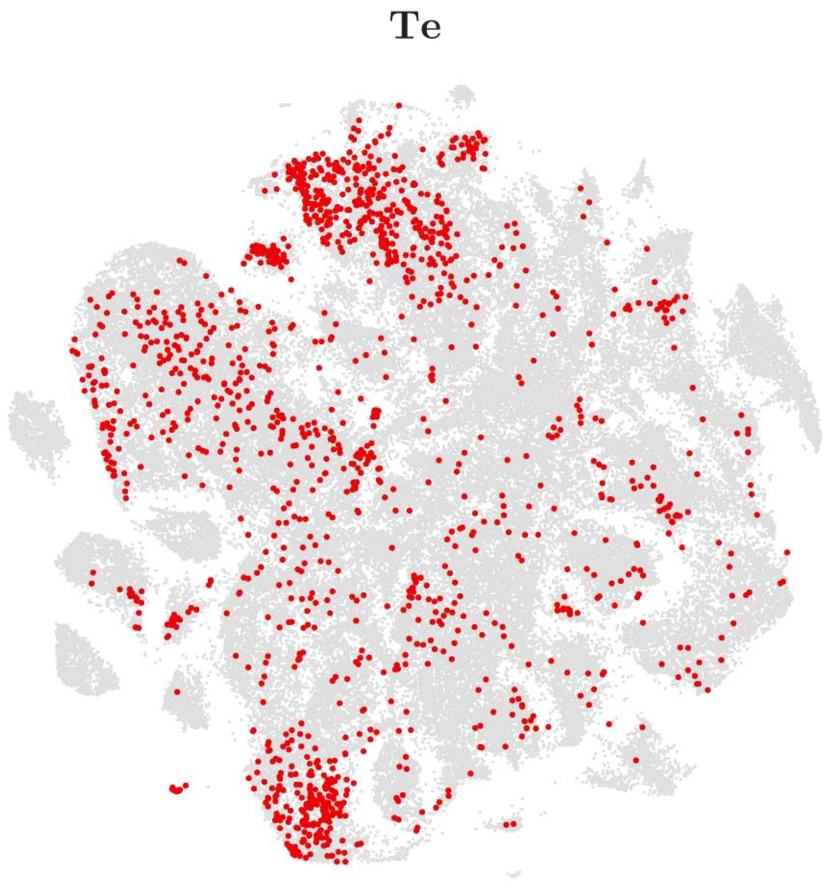

Figure S110. Caption Cluster plot for Iodine.

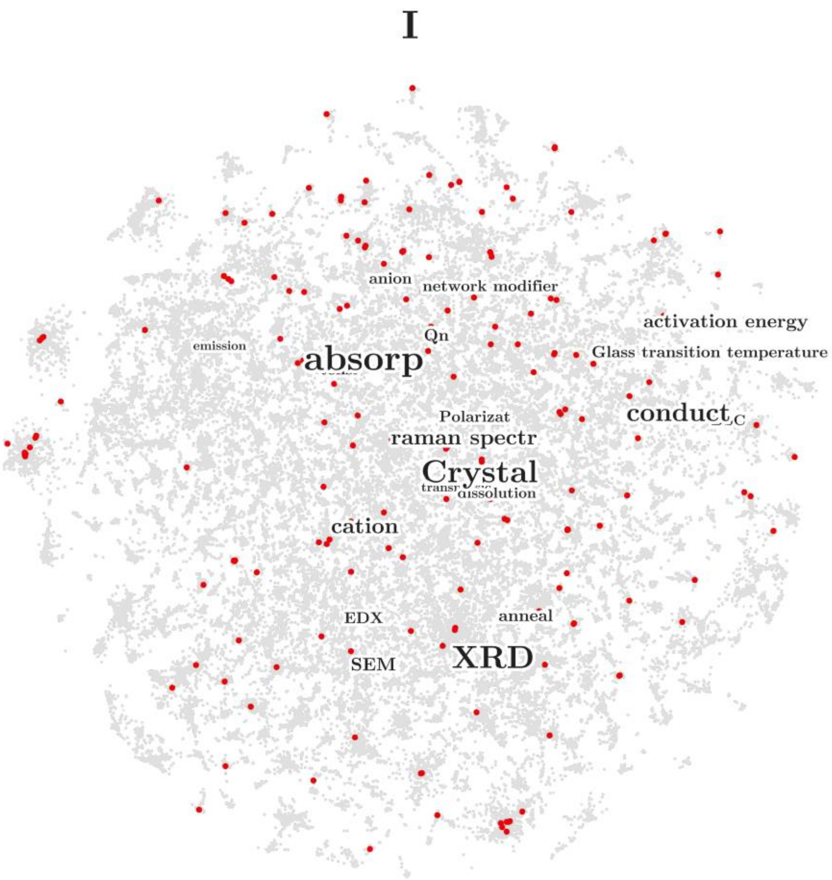

Figure S111. Latent Dirichlet Allocation plot for Iodine.

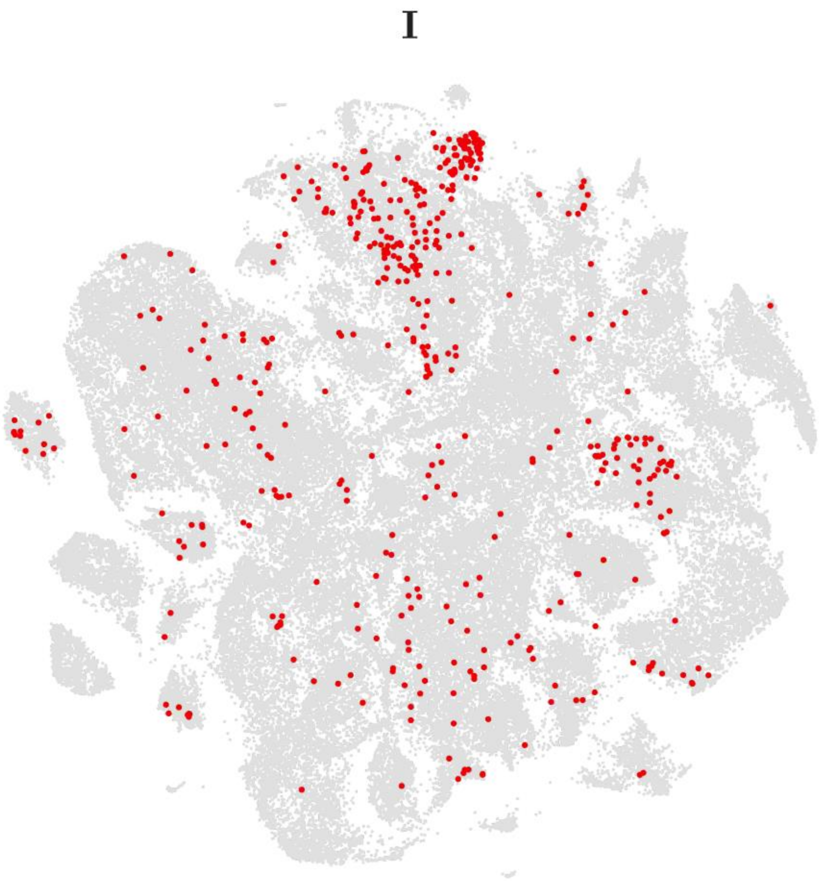

Figure S112. Caption Cluster plot for Xenon.

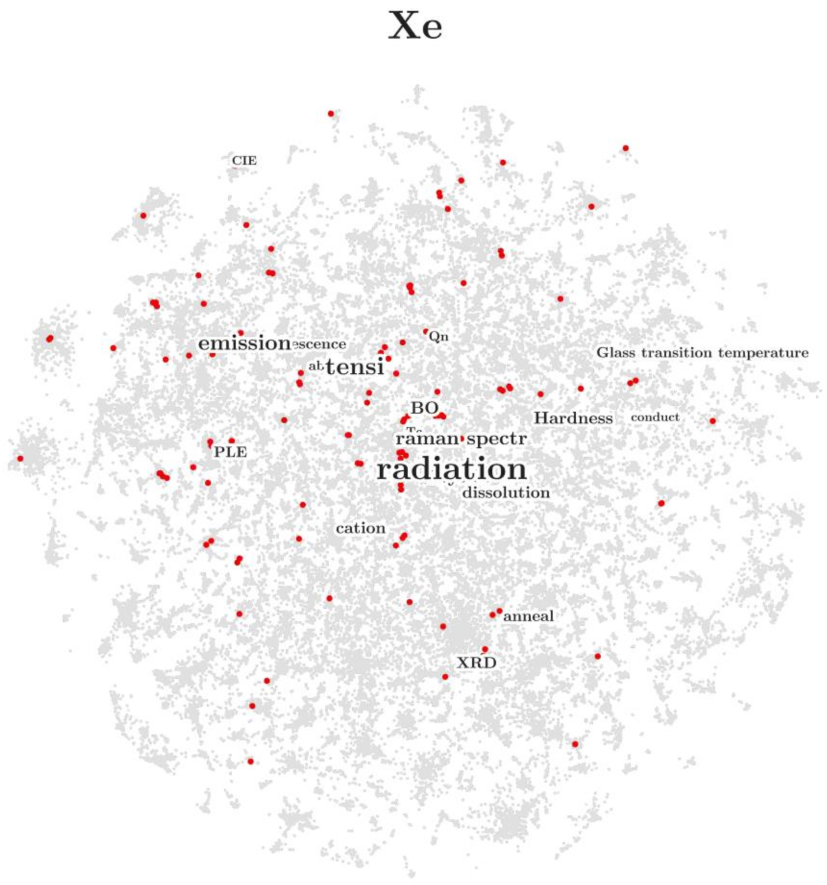

Figure S113. Latent Dirichlet Allocation plot for Xenon.

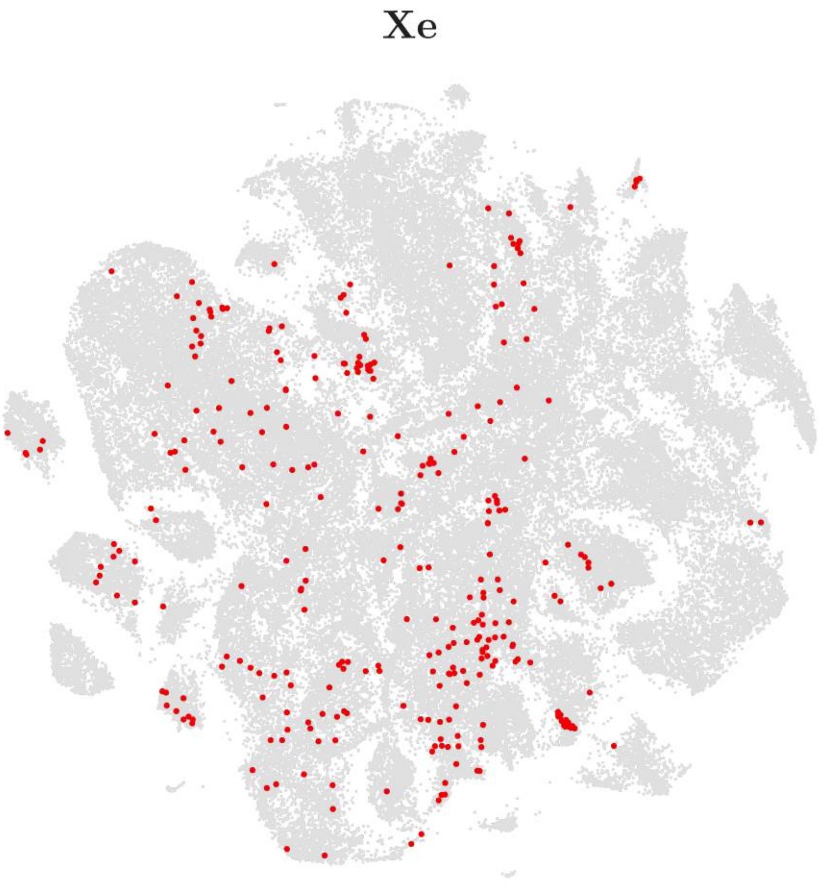

Figure S114. Caption Cluster plot for Cesium.

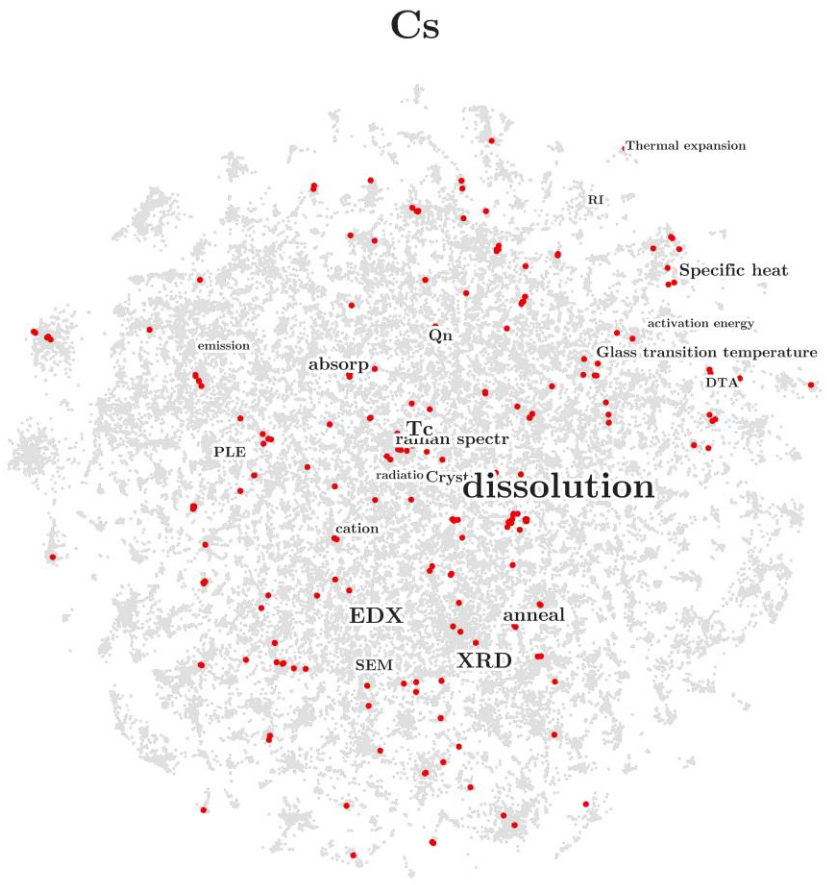

Figure S115. Latent Dirichlet Allocation plot for Cesium.

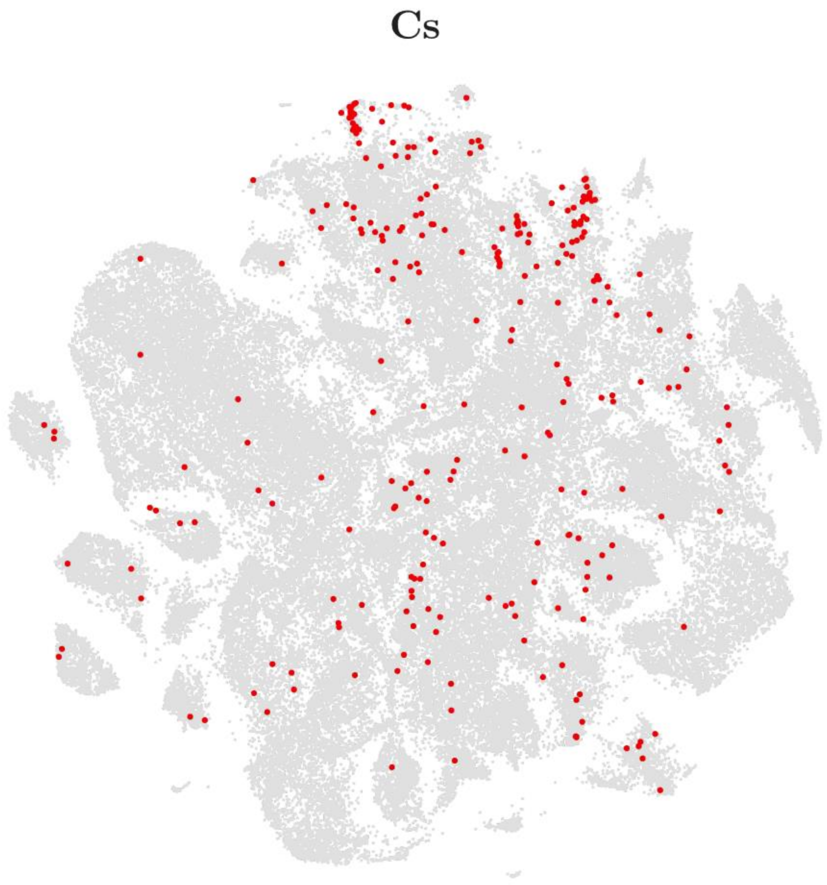

Figure S116. Caption Cluster plot for Barium.

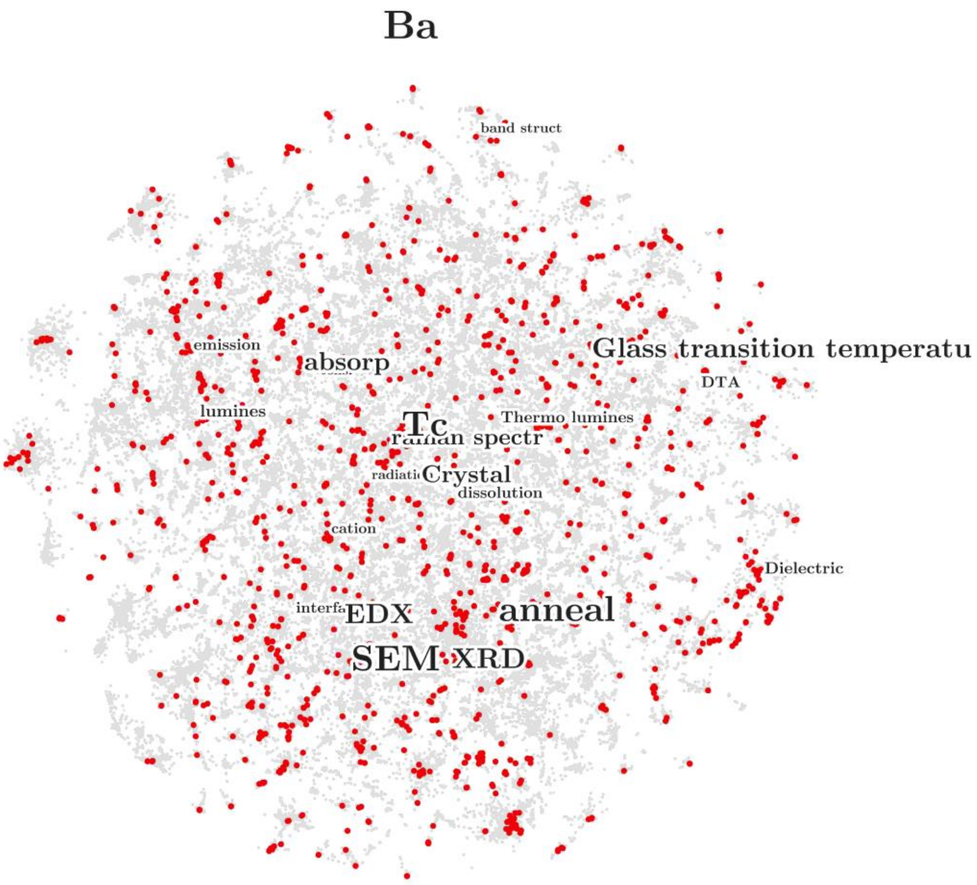

Figure S117. Latent Dirichlet Allocation plot for Barium.

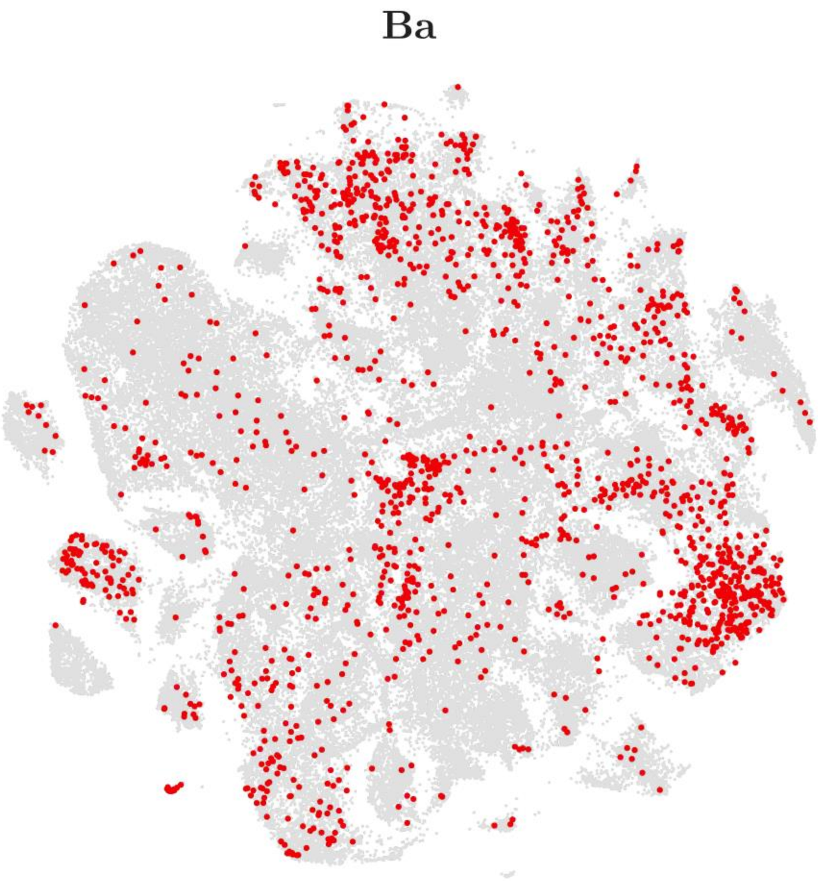

Figure S118. Caption Cluster plot for Lanthanum.

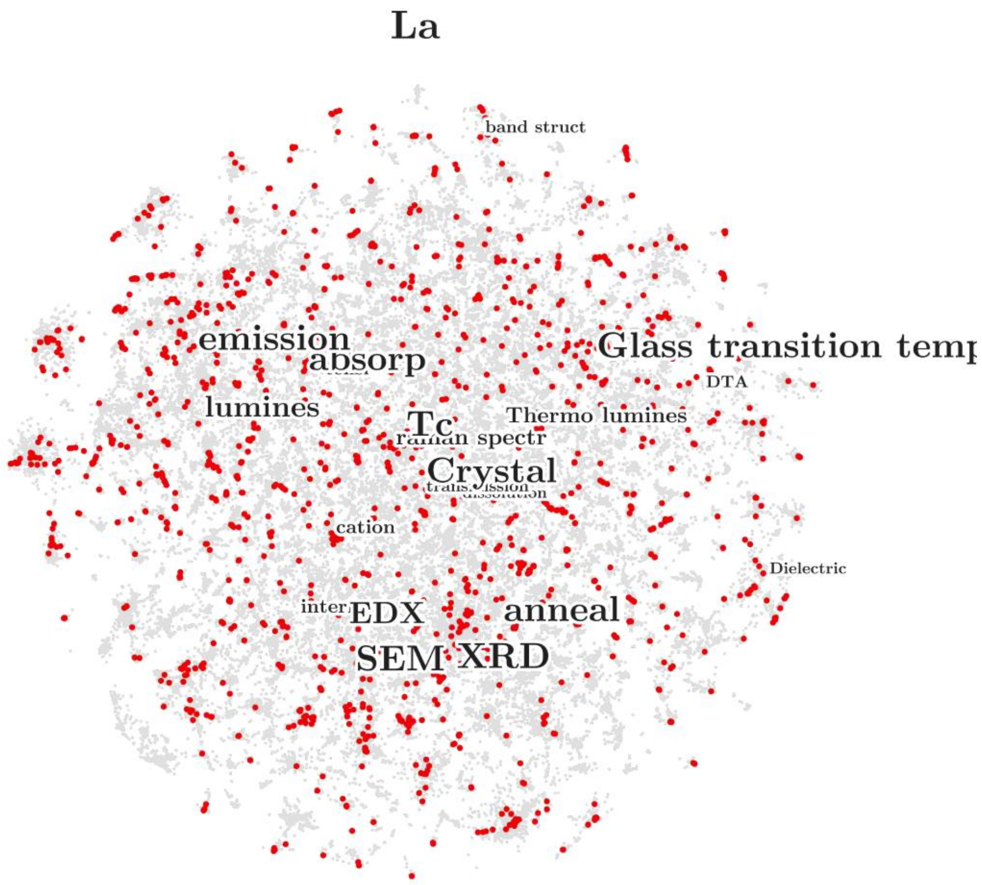

Figure S119. Latent Dirichlet Allocation plot for Lanthanum.

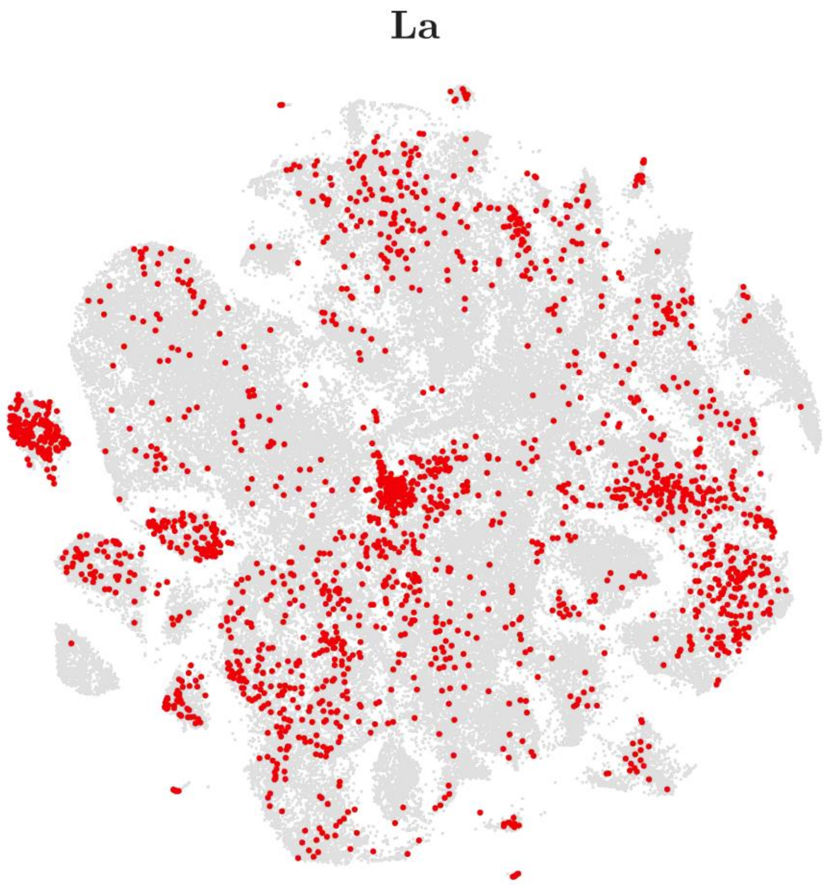

Figure S120. Caption Cluster plot for Cerium.

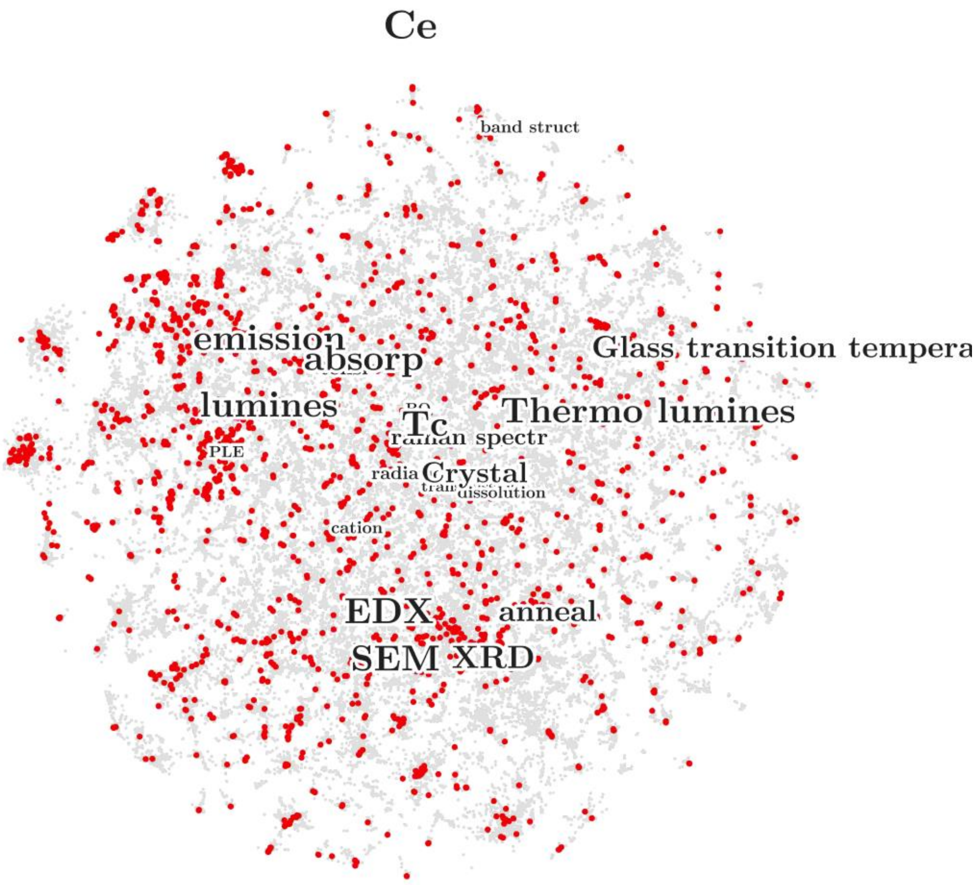

Figure S121. Latent Dirichlet Allocation plot for Cerium.

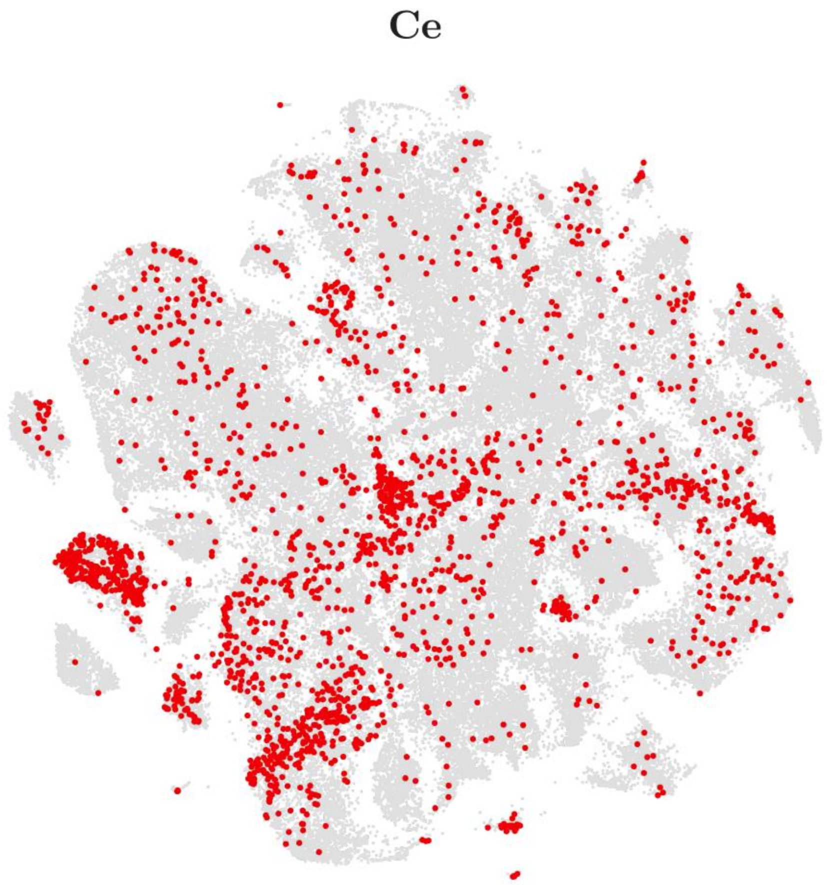

Figure S122. Caption Cluster plot for Praseodymium.

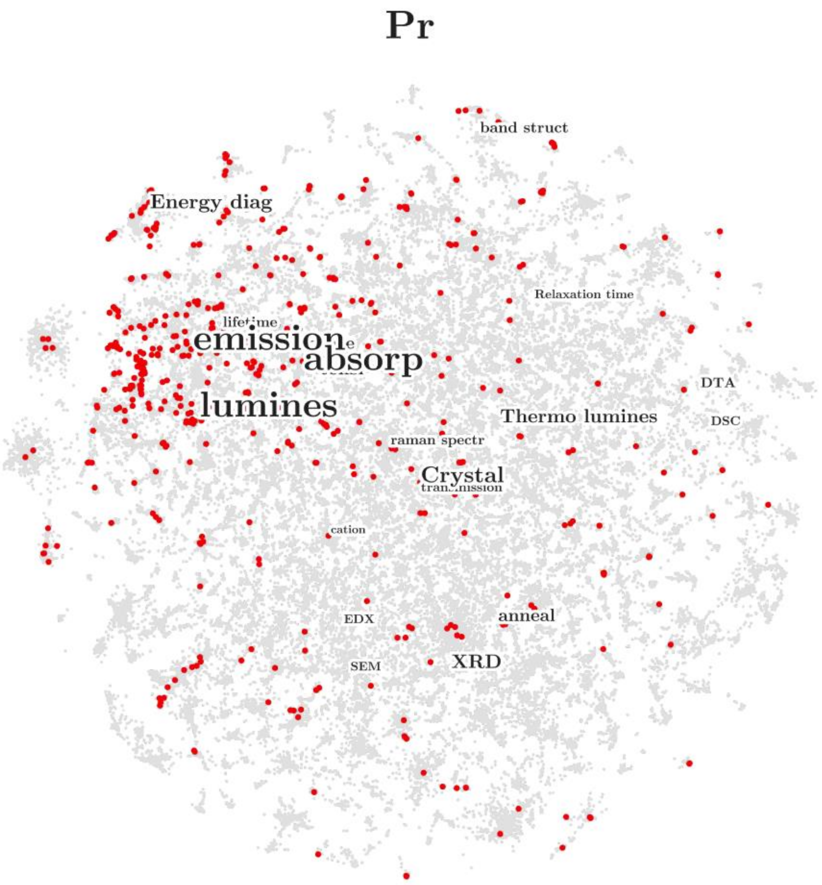

Figure S123. Latent Dirichlet Allocation plot for Praseodymium.

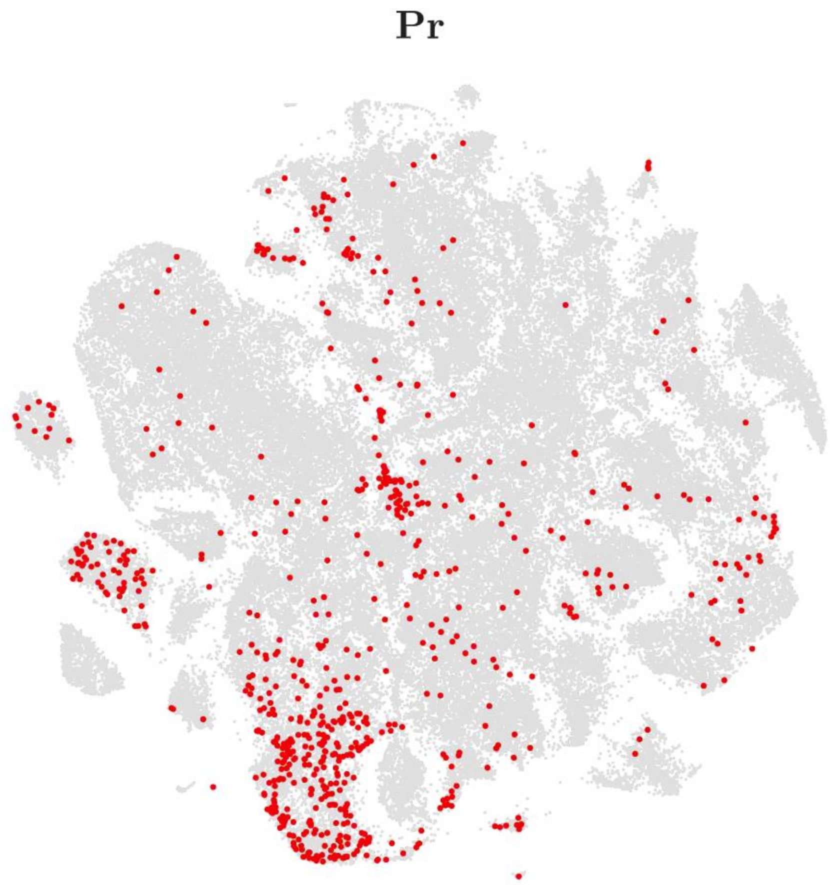

Figure S124. Caption Cluster plot for Neodymium.

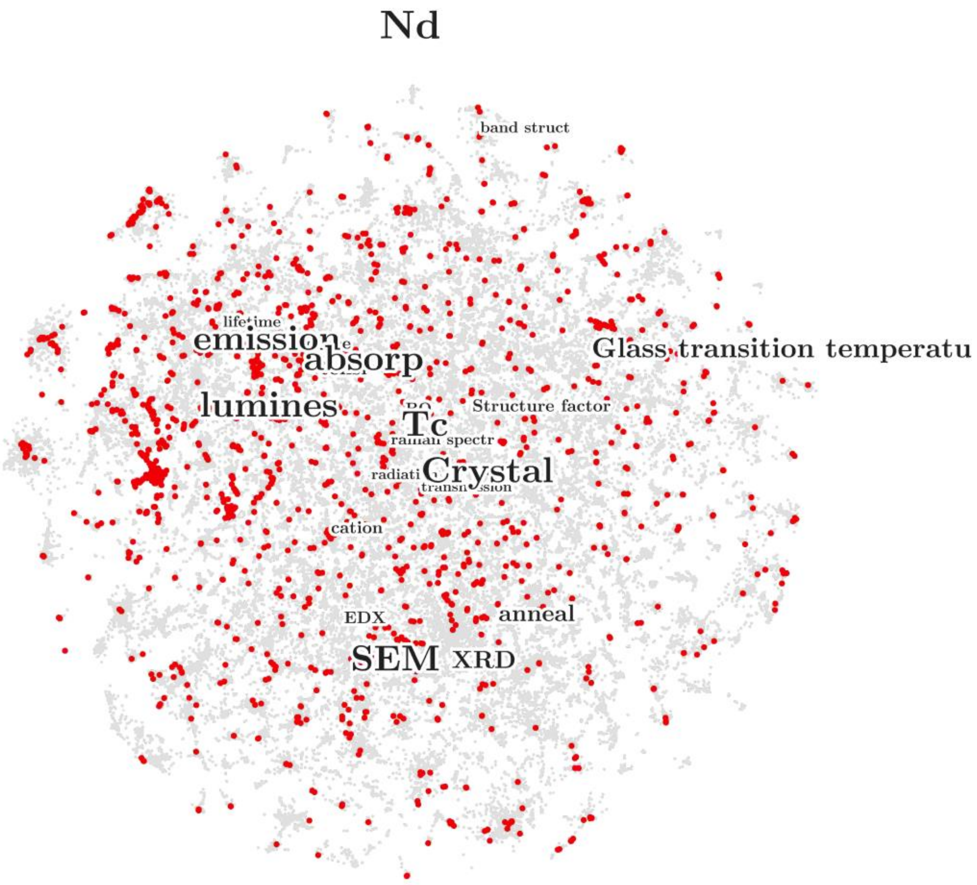

Figure S125. Latent Dirichlet Allocation plot for Neodymium.

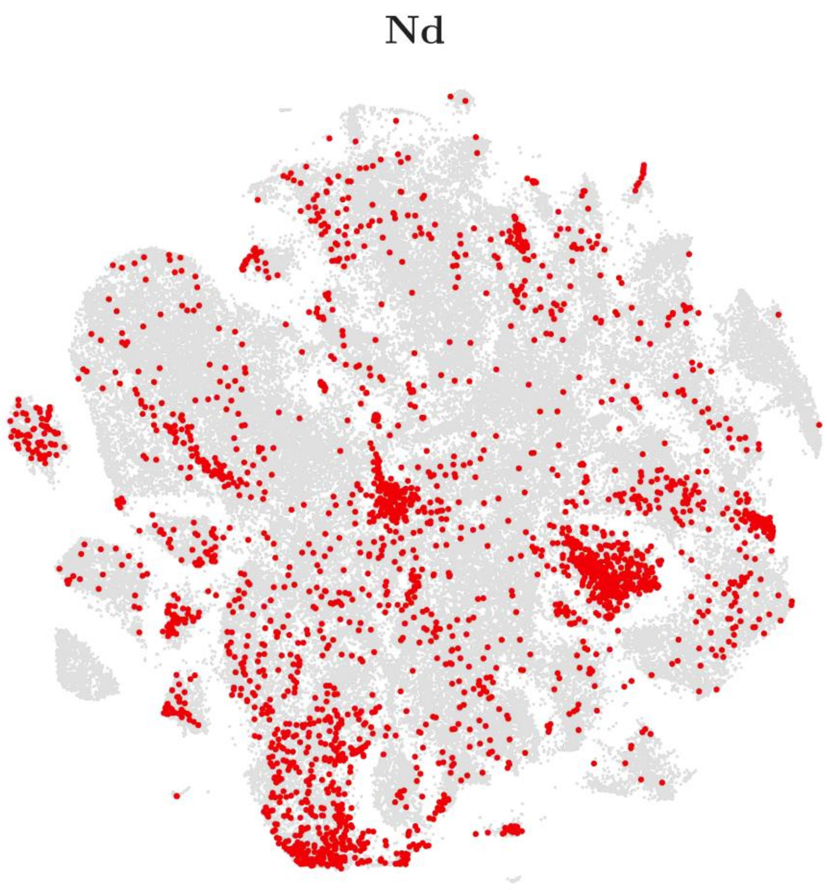

Figure S126. Caption Cluster plot for Promethium.

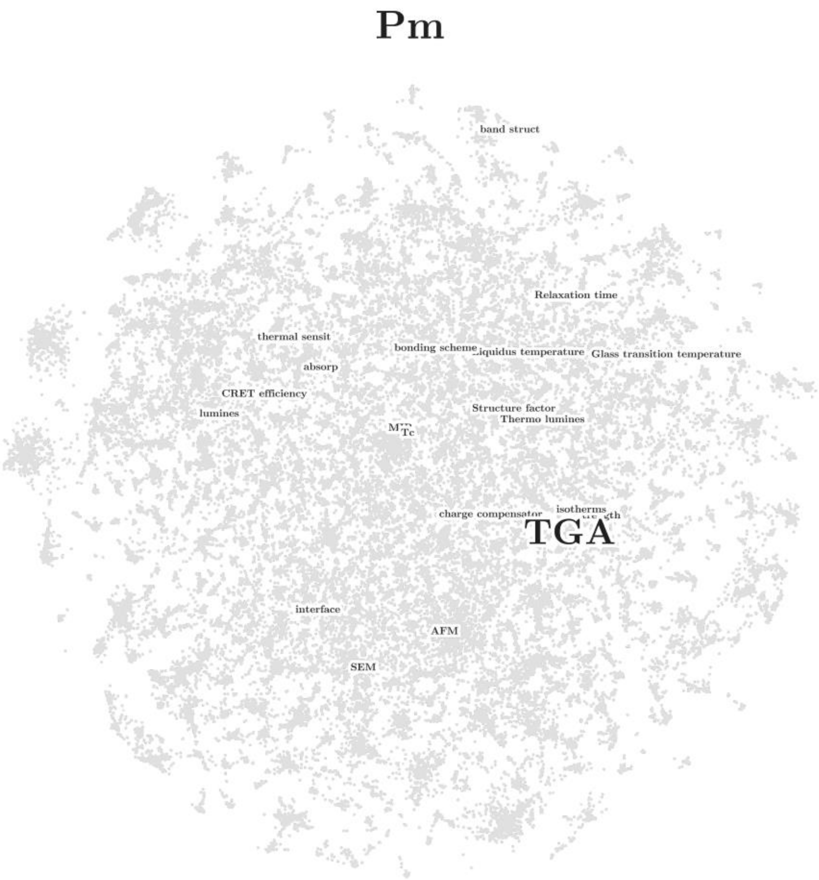

Figure S127. Latent Dirichlet Allocation plot for Promethium.

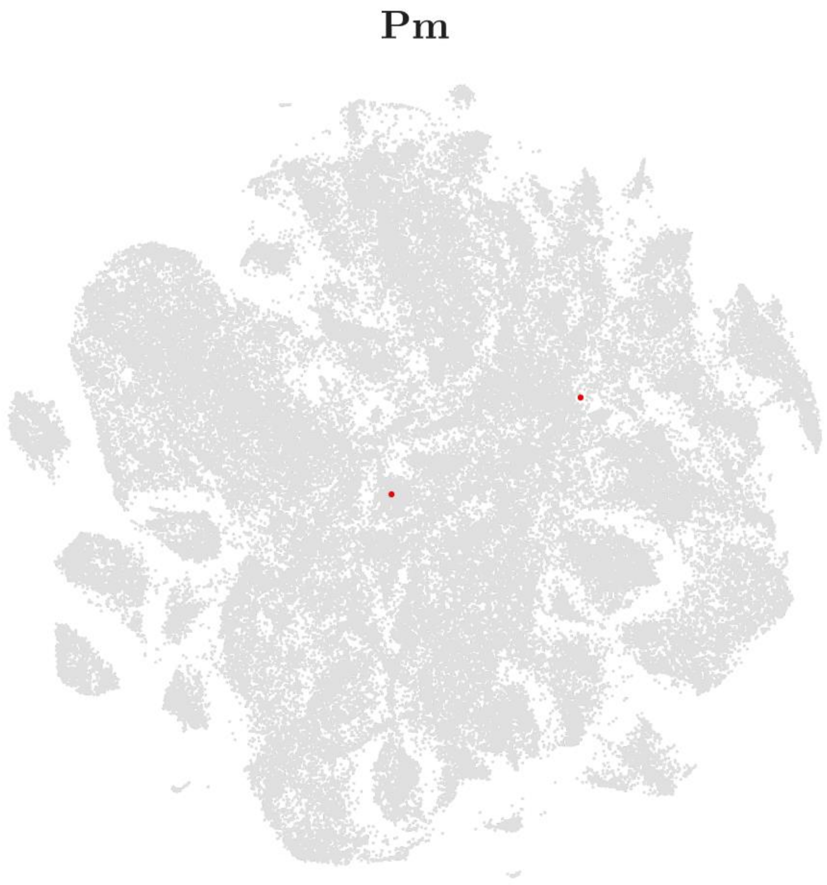

Figure S128. Caption Cluster plot for Samarium.

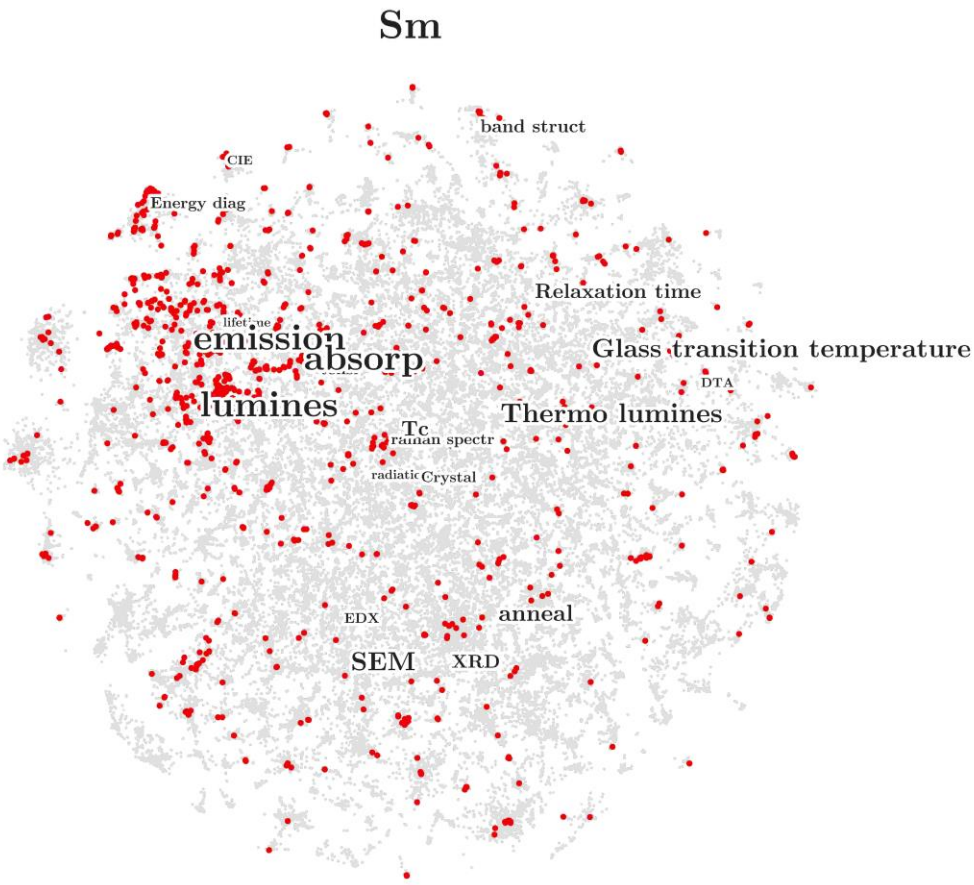

Figure S129. Latent Dirichlet Allocation plot for Samarium.

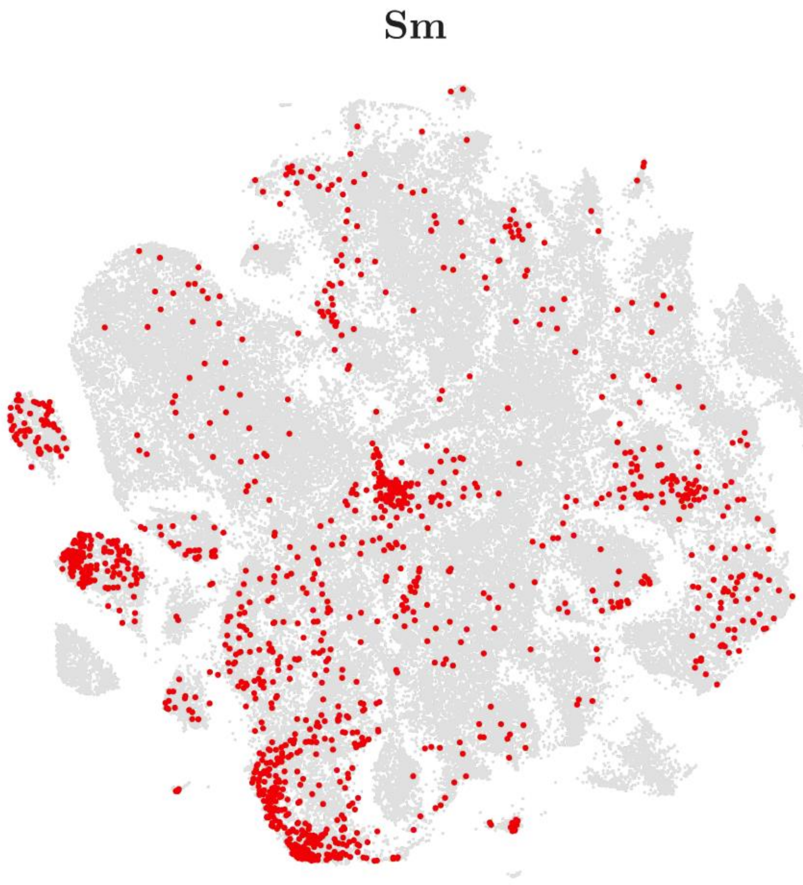

Figure S130. Caption Cluster plot for Europium.

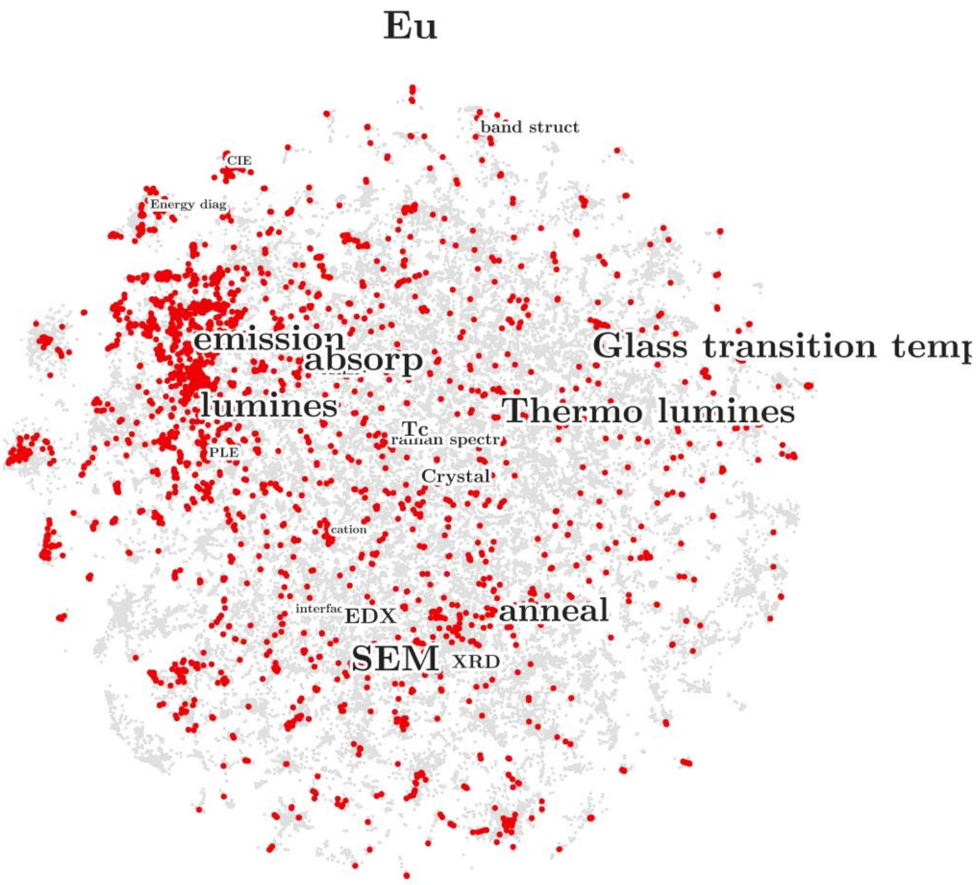

Figure S131. Latent Dirichlet Allocation plot for Europium.

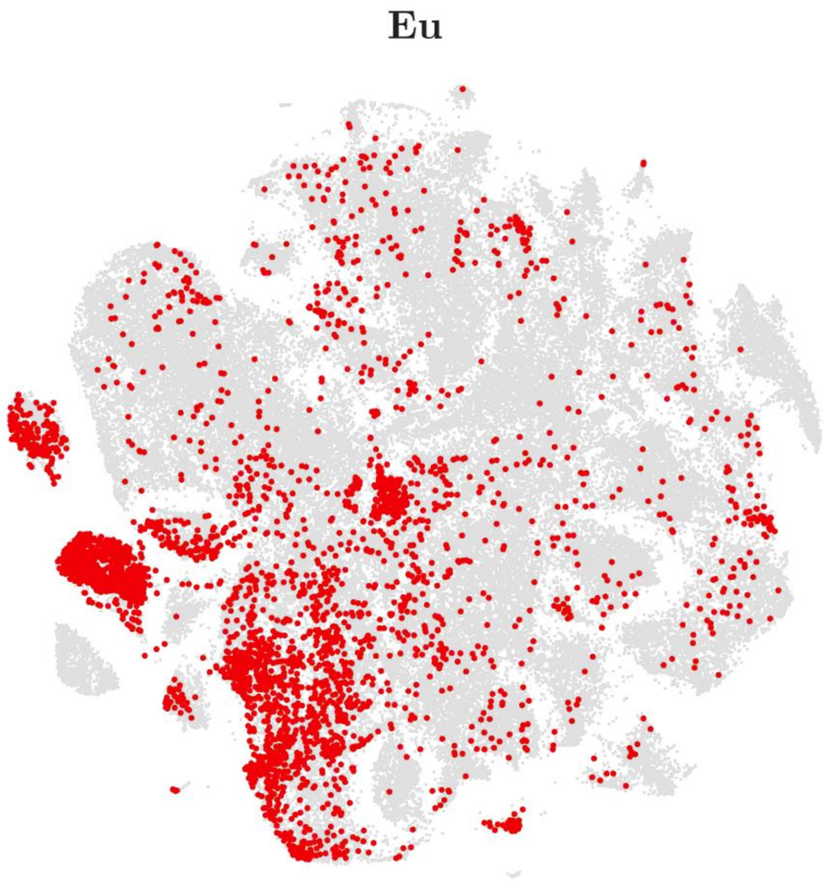

Figure S132. Caption Cluster plot for Gadolinium.

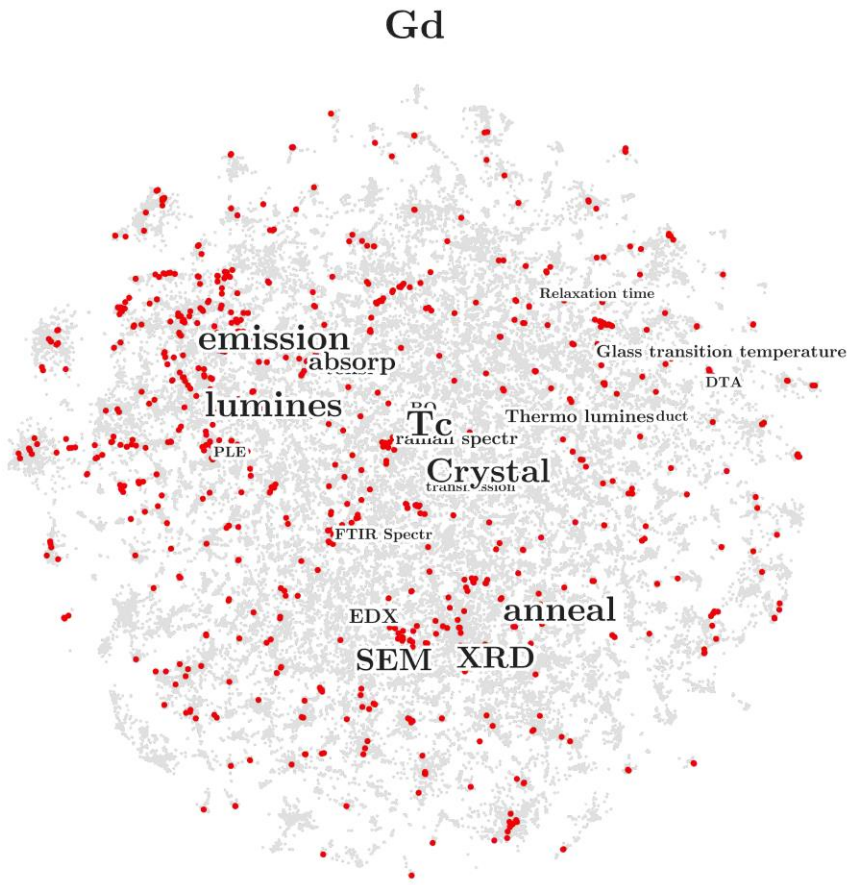

Figure S133. Latent Dirichlet Allocation plot for Gadolinium.

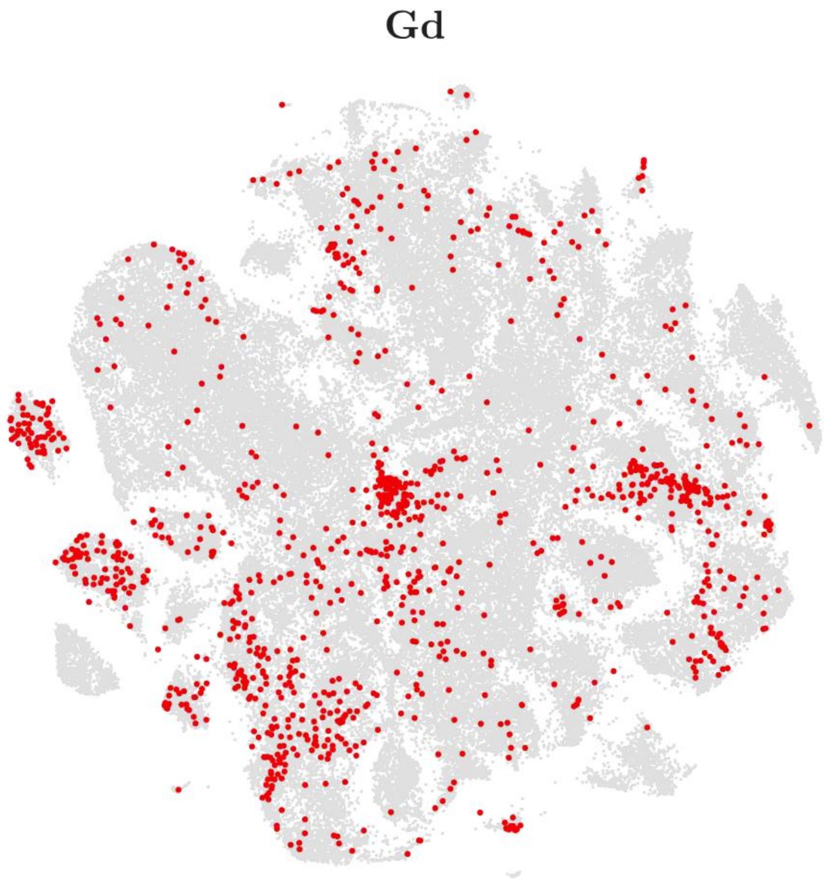

Figure S134. Caption Cluster plot for Terbium.

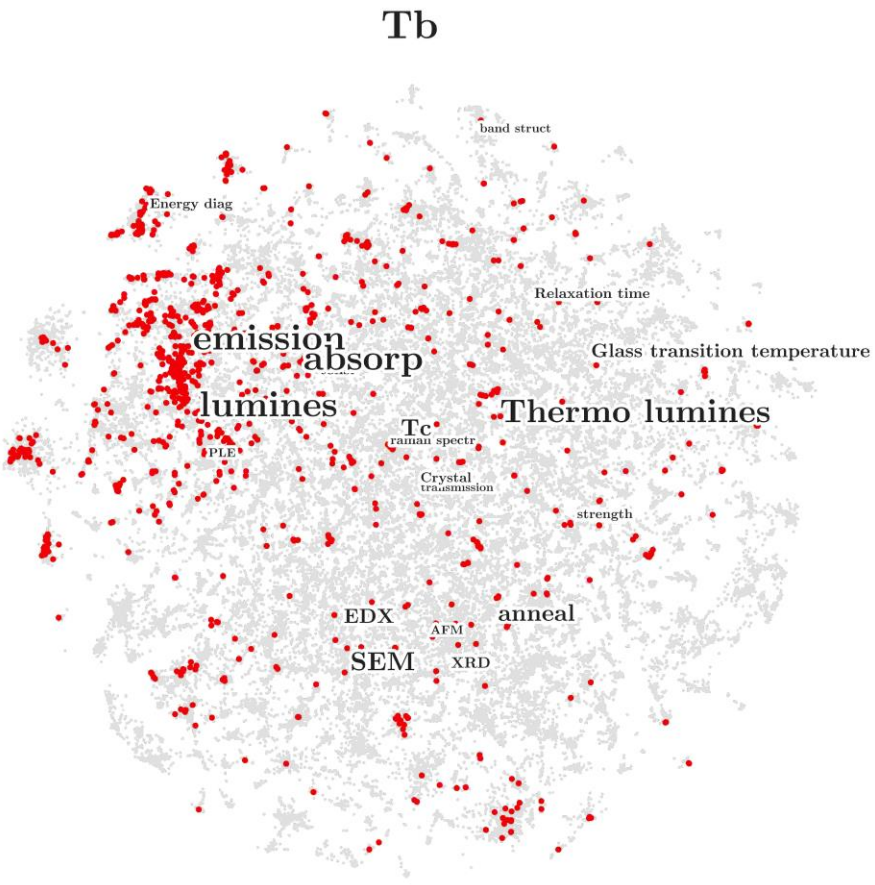

Figure S135. Latent Dirichlet Allocation plot for Terbium.

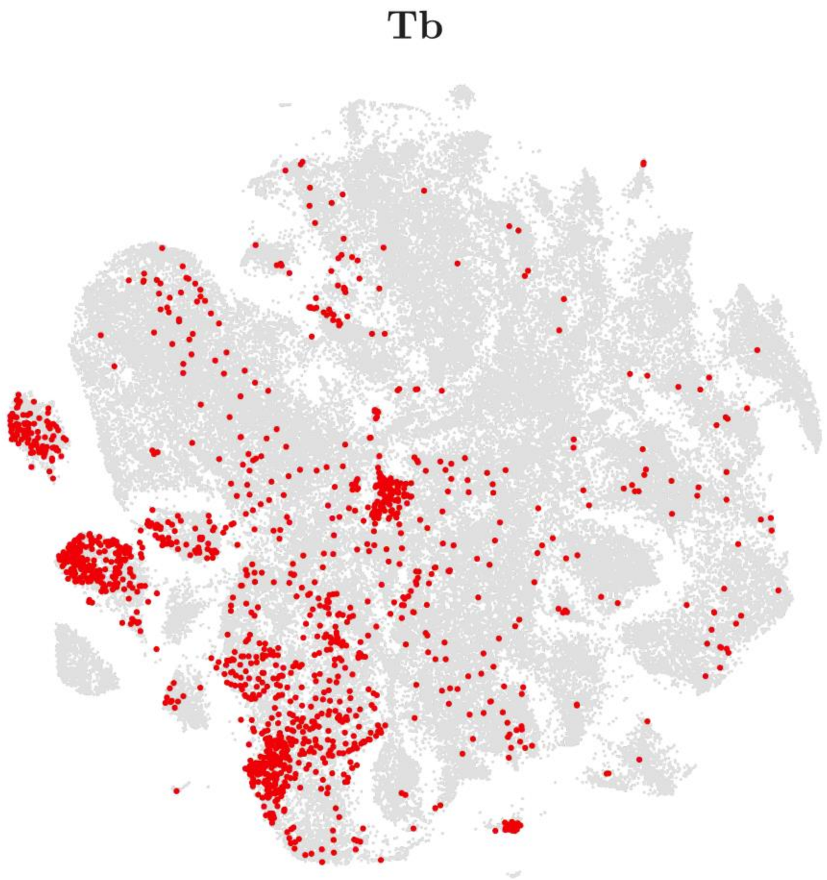

Figure S136. Caption Cluster plot for Dysprosium.

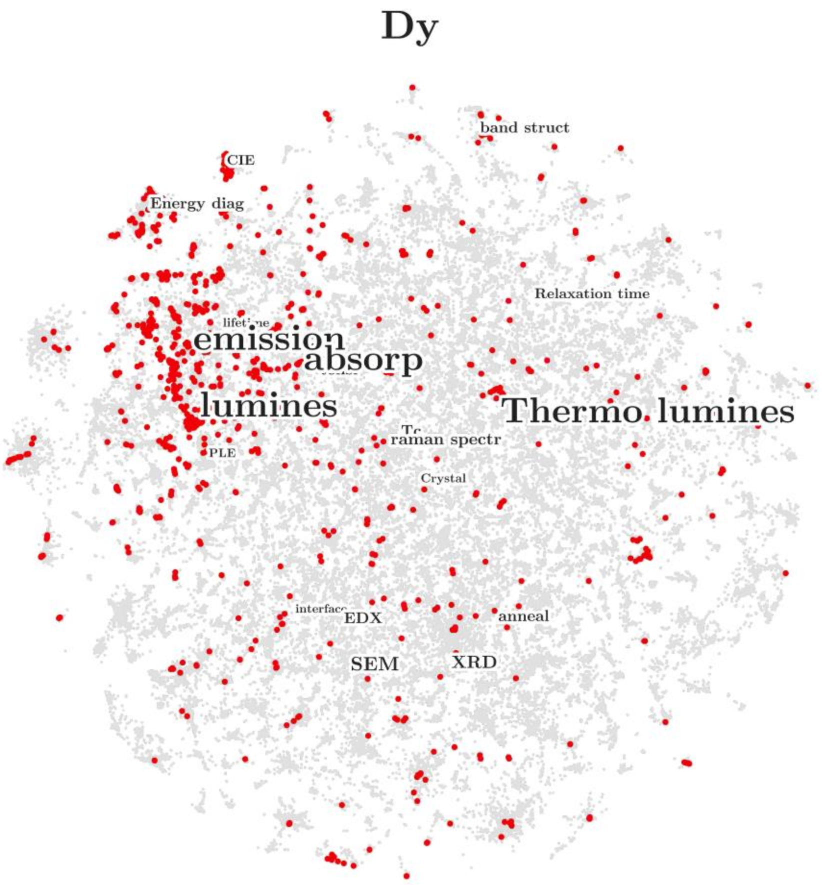

Figure S137. Latent Dirichlet Allocation plot for Dysprosium.

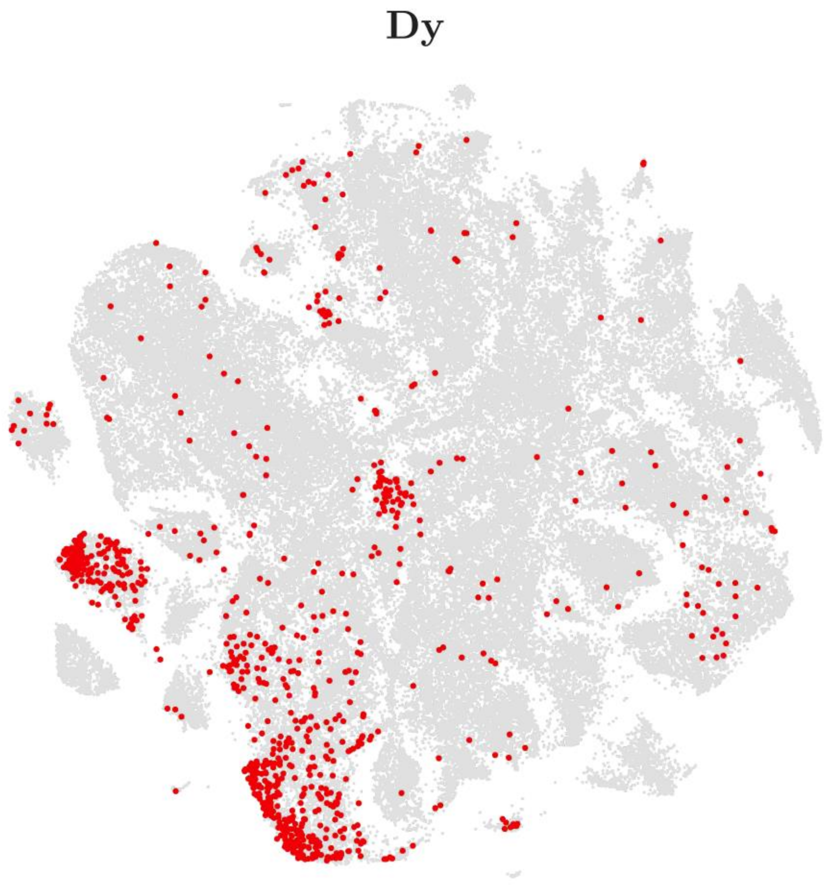

Figure S138. Caption Cluster plot for Holmium.

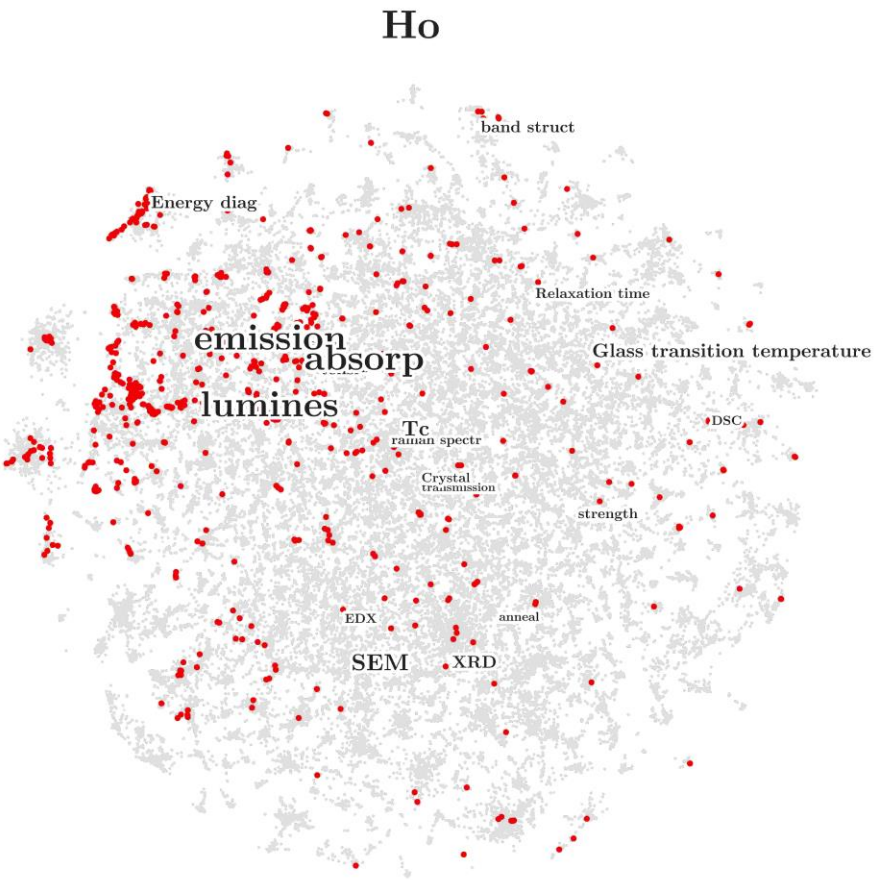

Figure S139. Latent Dirichlet Allocation plot for Holmium.

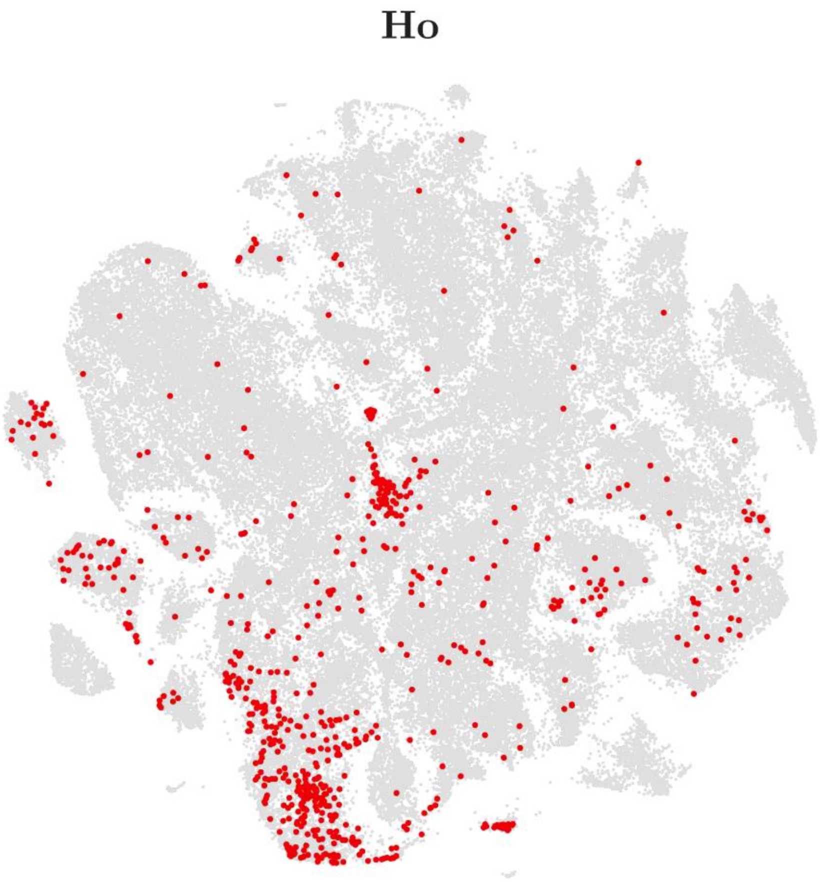

Figure S140. Caption Cluster plot for Erbium.

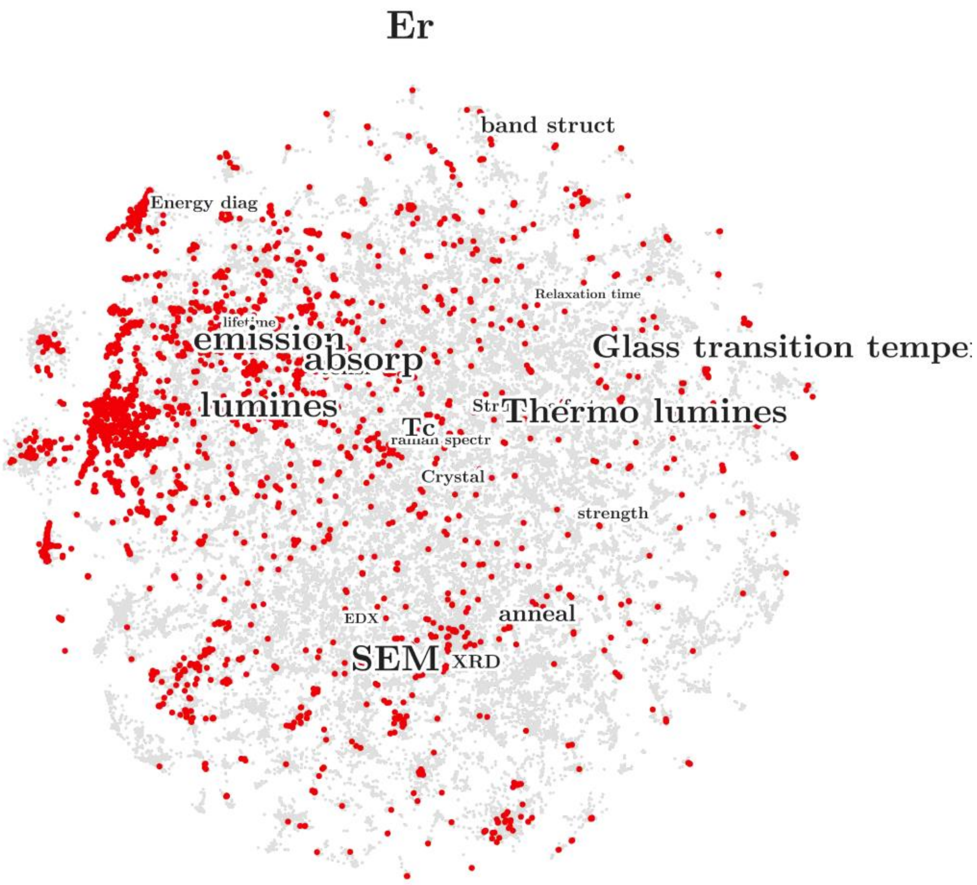

Figure S141. Latent Dirichlet Allocation plot for Erbium.

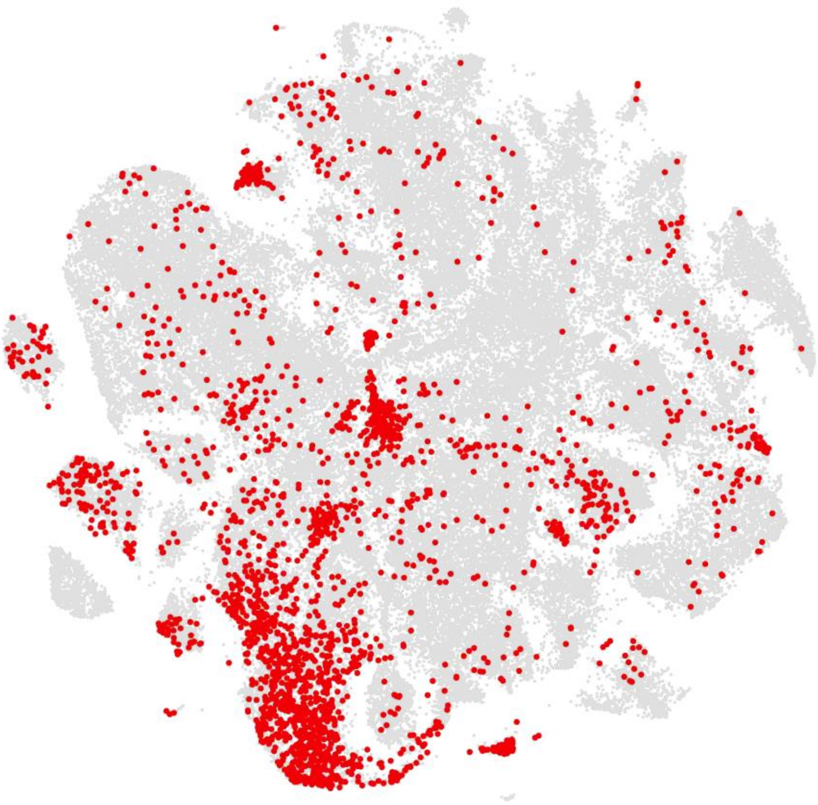

Figure S142. Caption Cluster plot for Thulium.

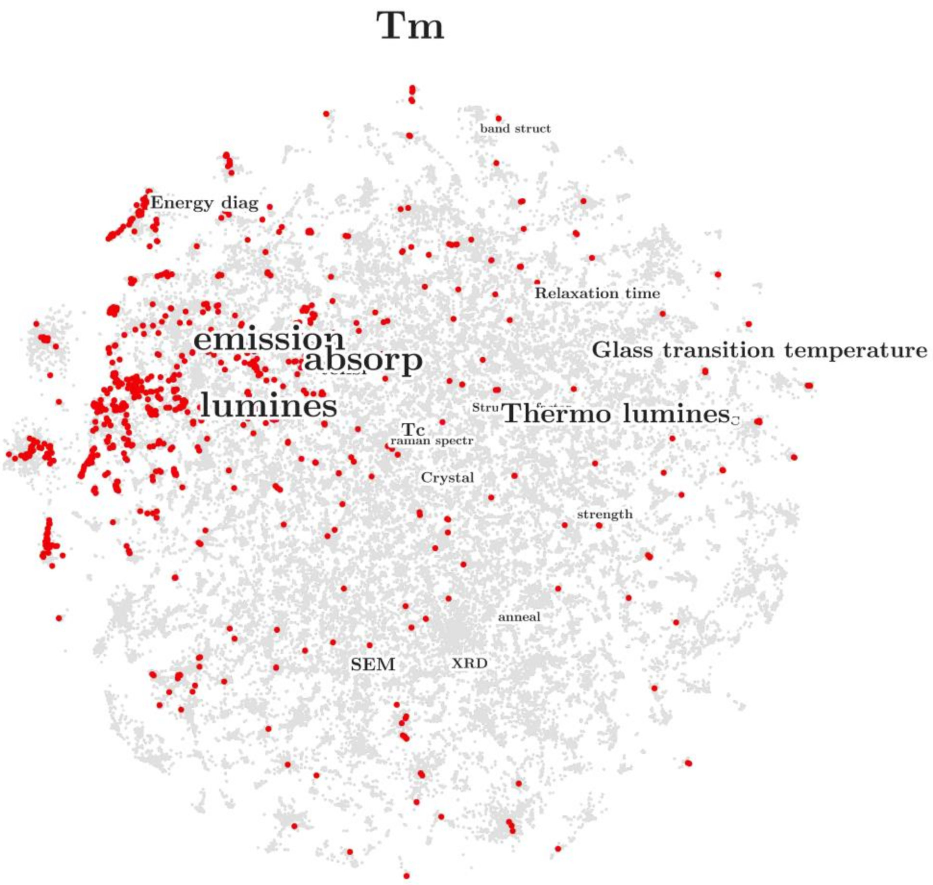

Figure S143. Latent Dirichlet Allocation plot for Thulium.

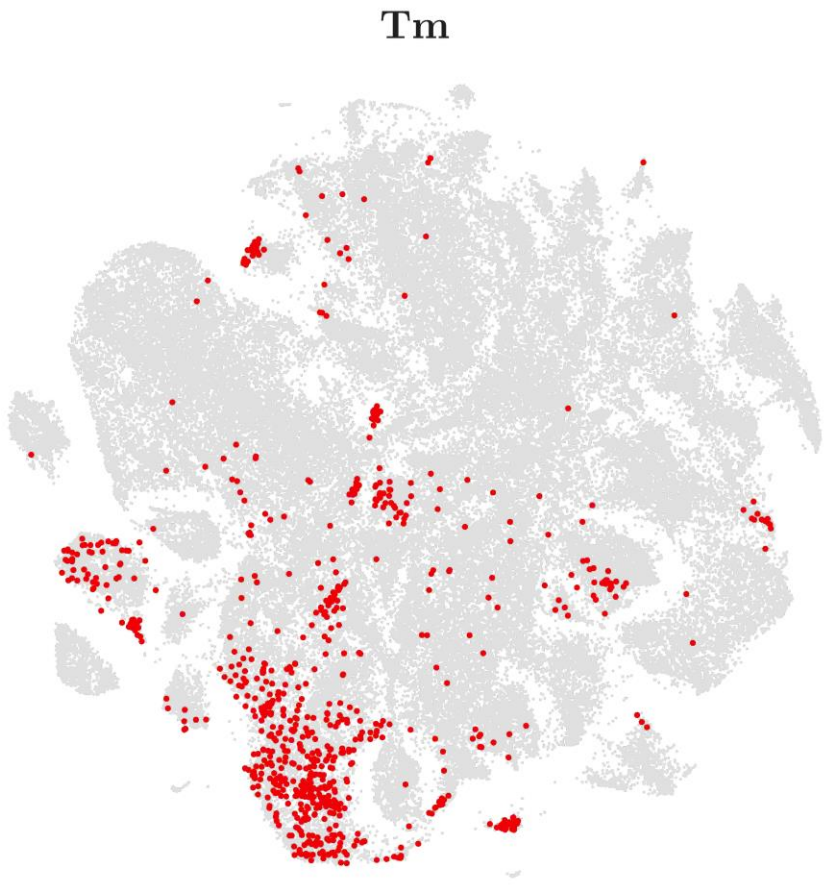

Figure S144. Caption Cluster plot for Ytterbium.

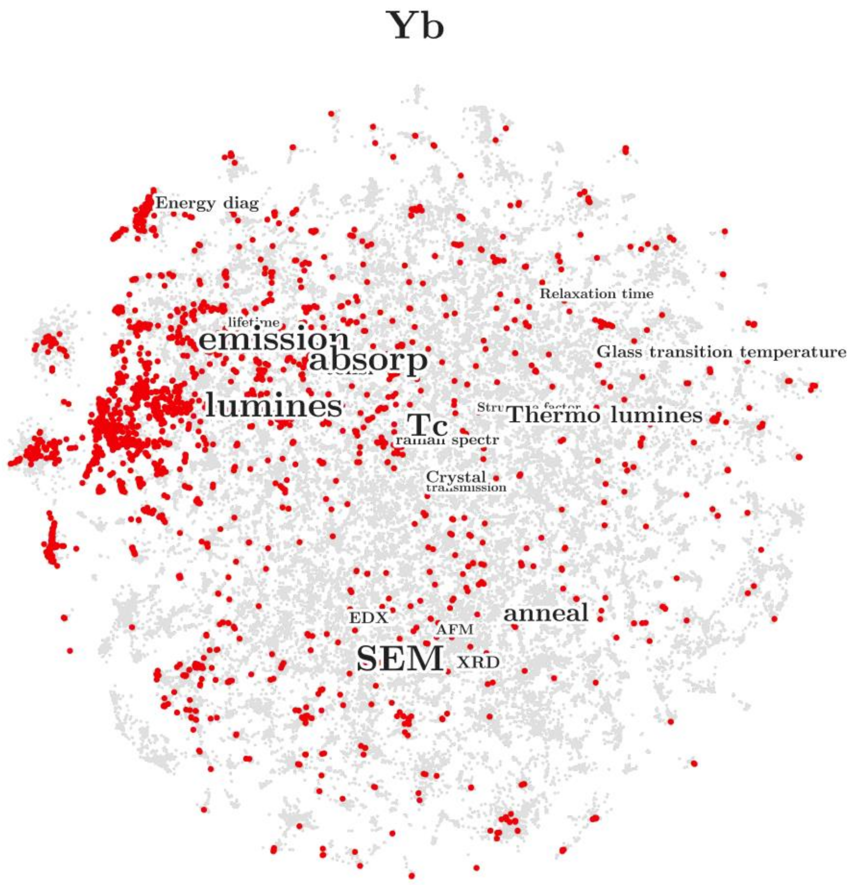

Figure S145. Latent Dirichlet Allocation plot for Ytterbium.

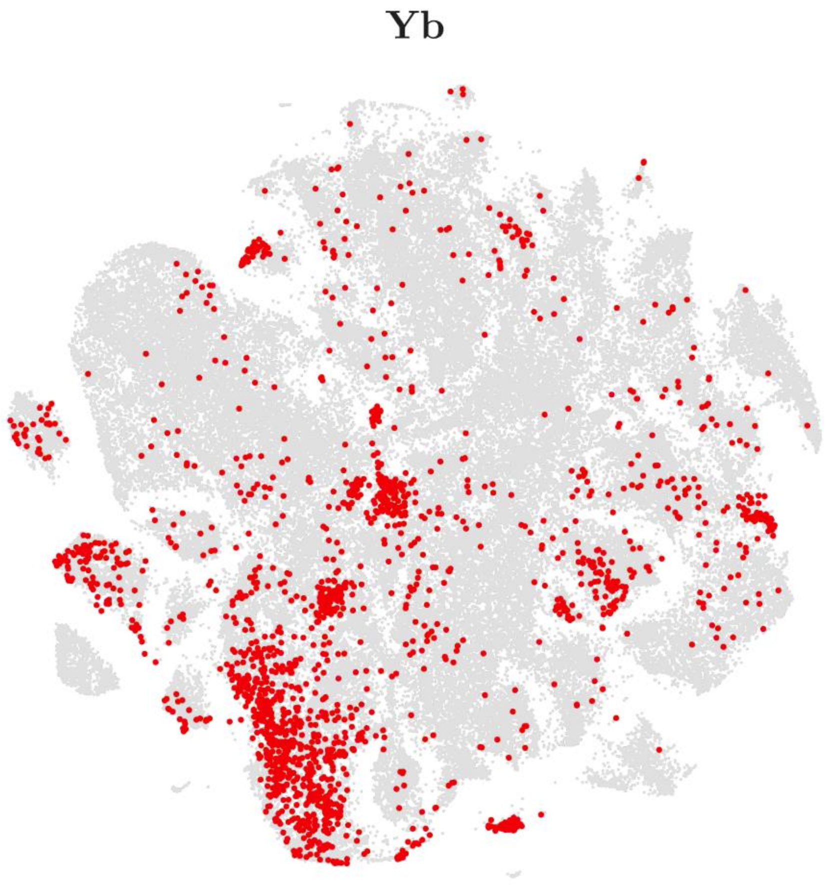

Figure S146. Caption Cluster plot for Lutetium.

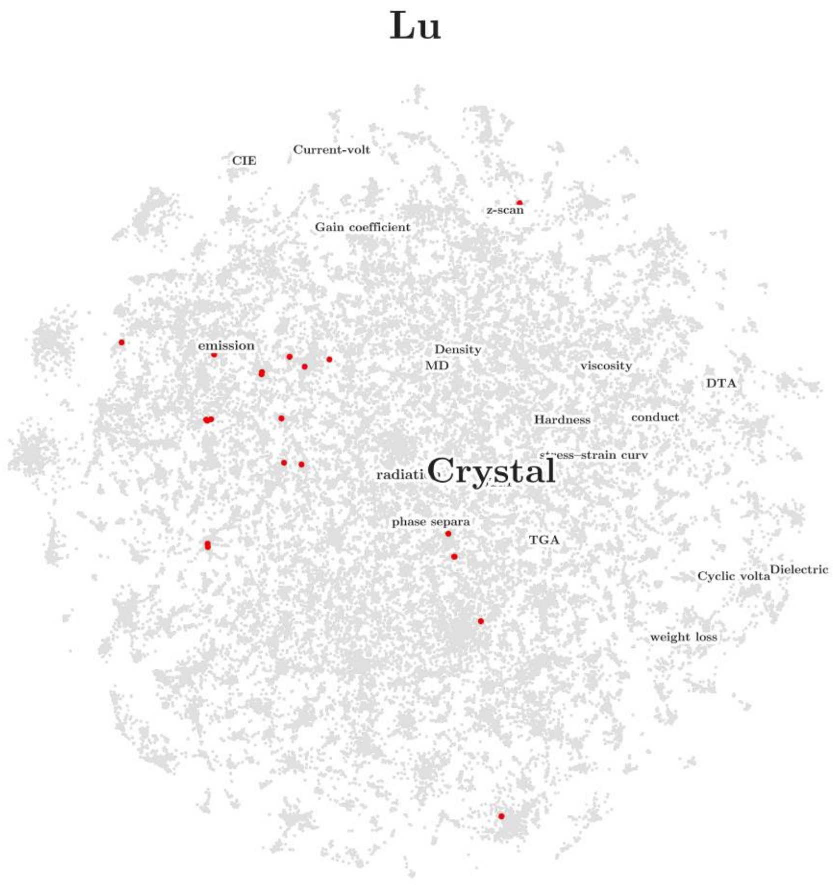

Figure S147. Latent Dirichlet Allocation plot for Lutetium.

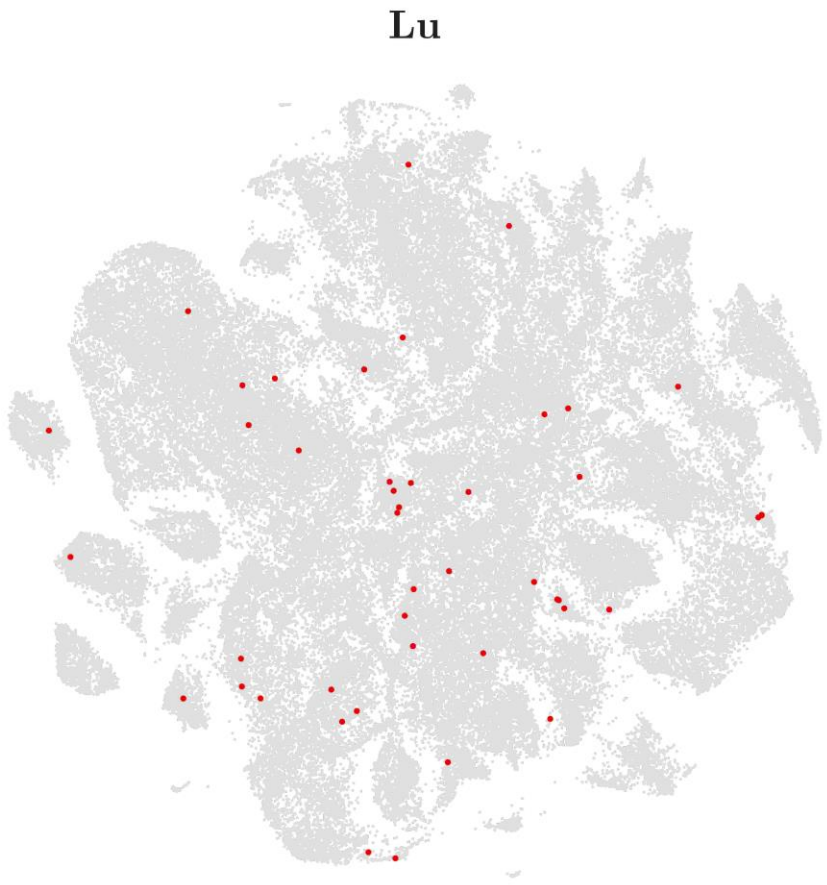

Figure S148. Caption Cluster plot for Hafnium.

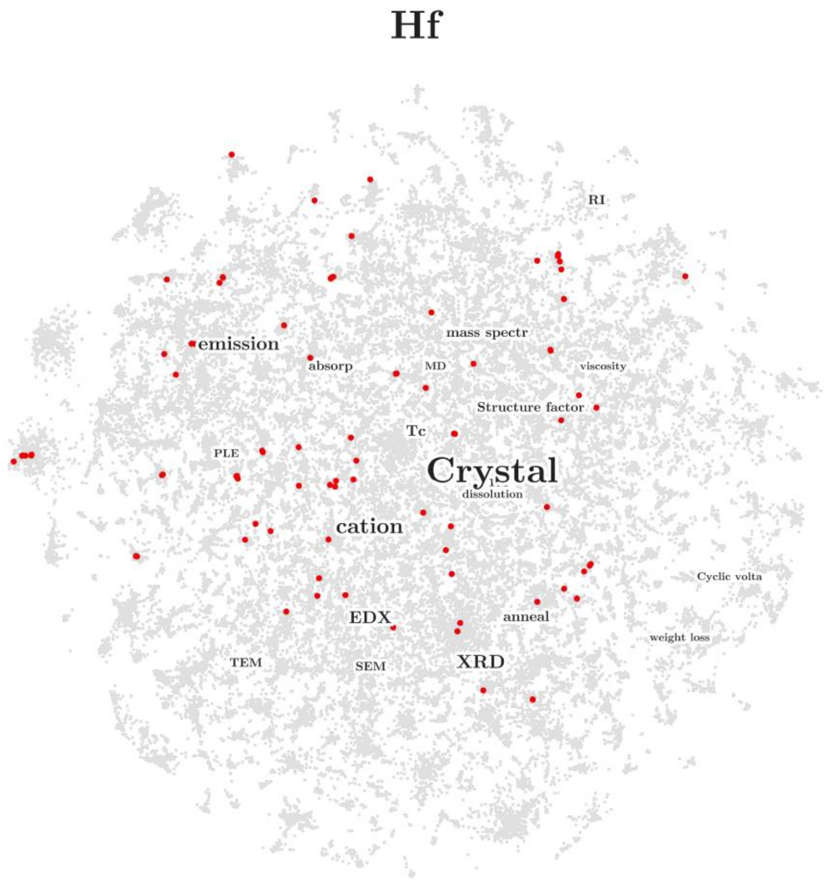

Figure S149. Latent Dirichlet Allocation plot for Hafnium.

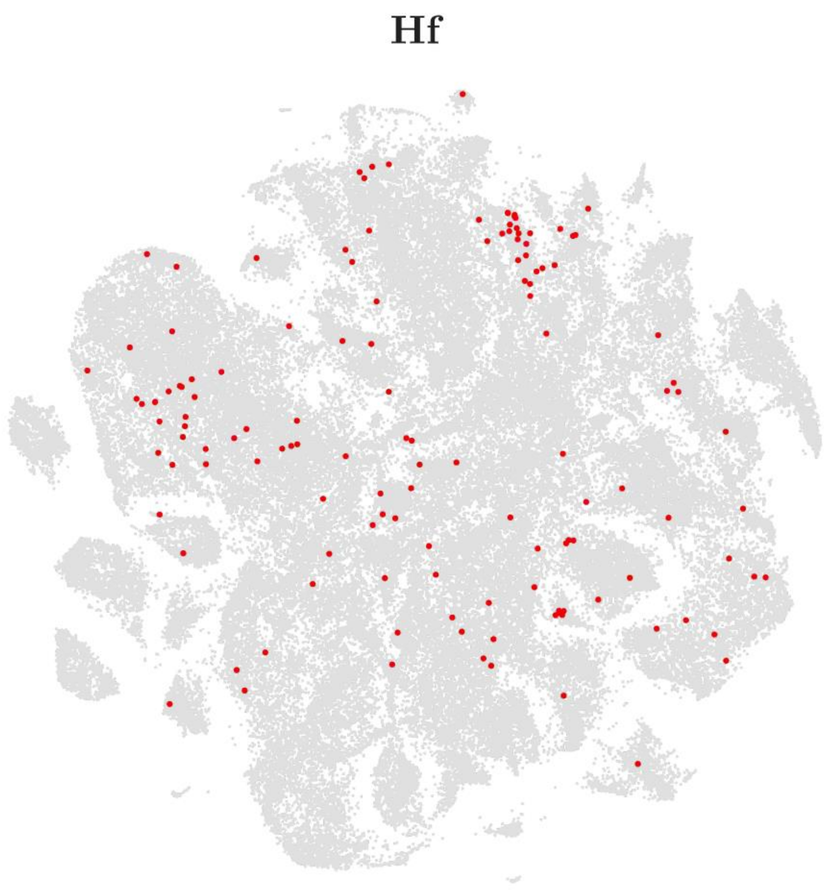

Figure S150. Caption Cluster plot for Tantalum.

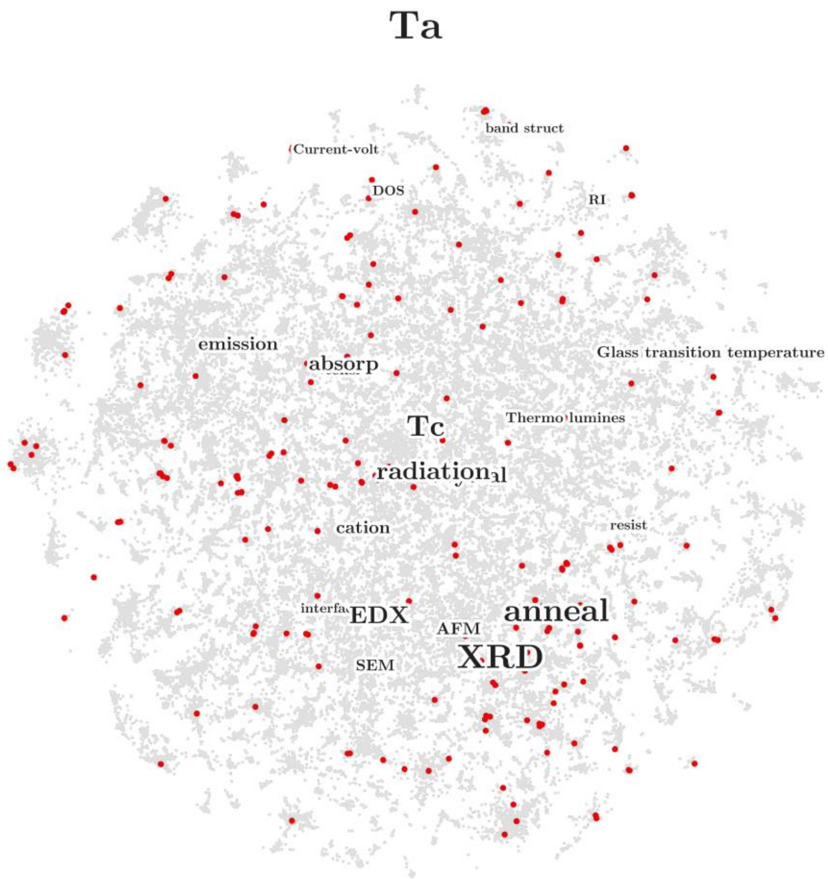

Figure S151. Latent Dirichlet Allocation plot for Tantalum.

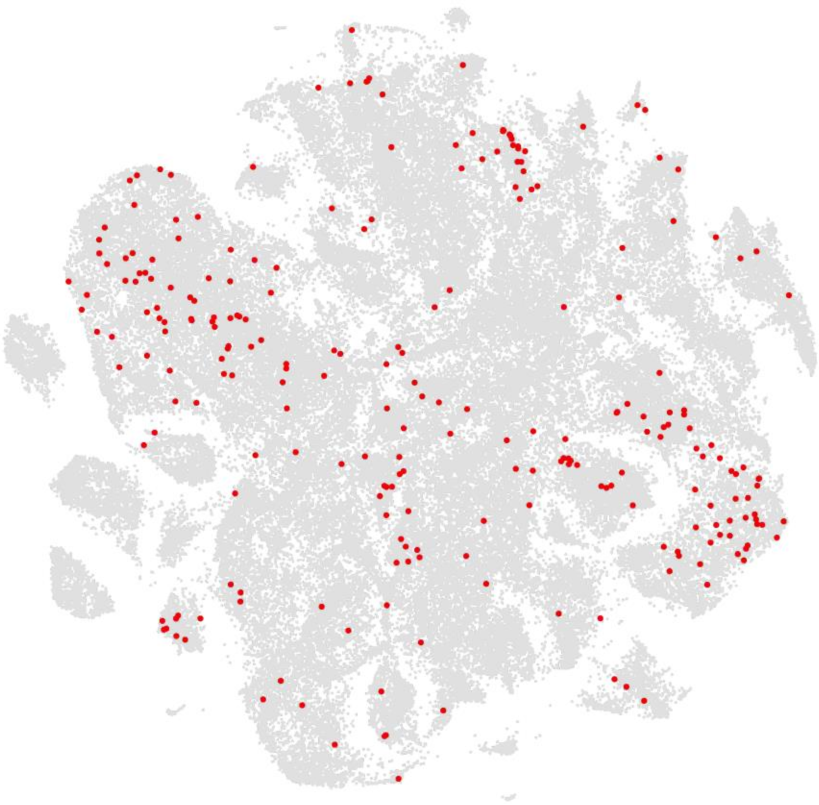

Figure S152. Caption Cluster plot for Tungsten.

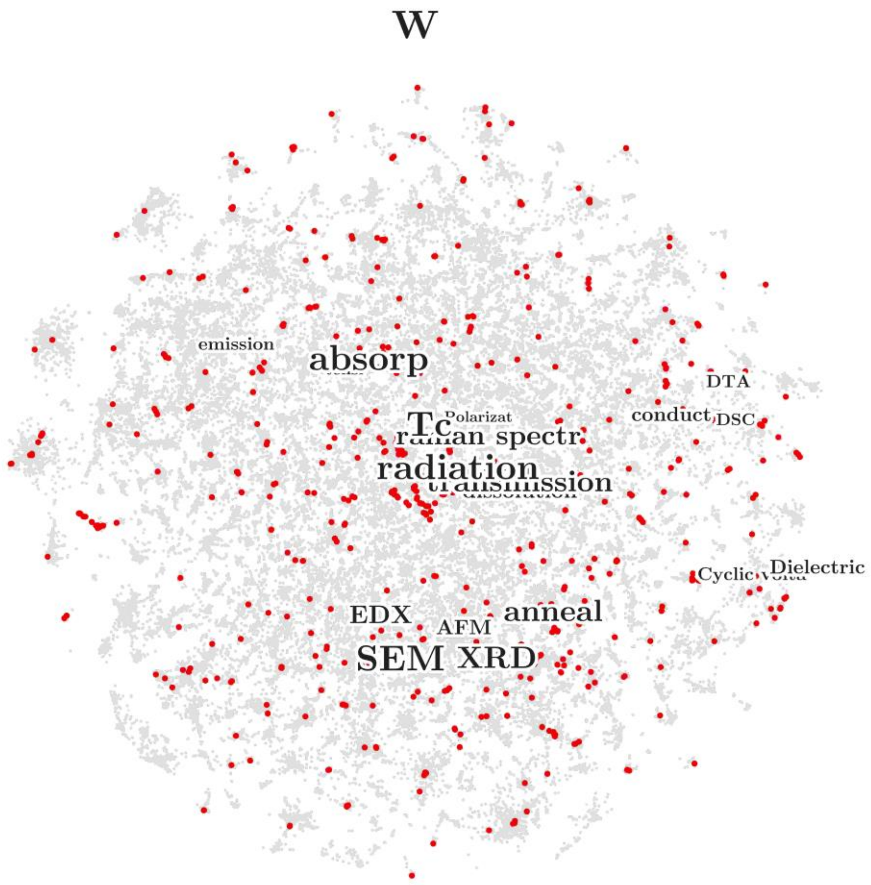

Figure S153. Latent Dirichlet Allocation plot for Tungsten.

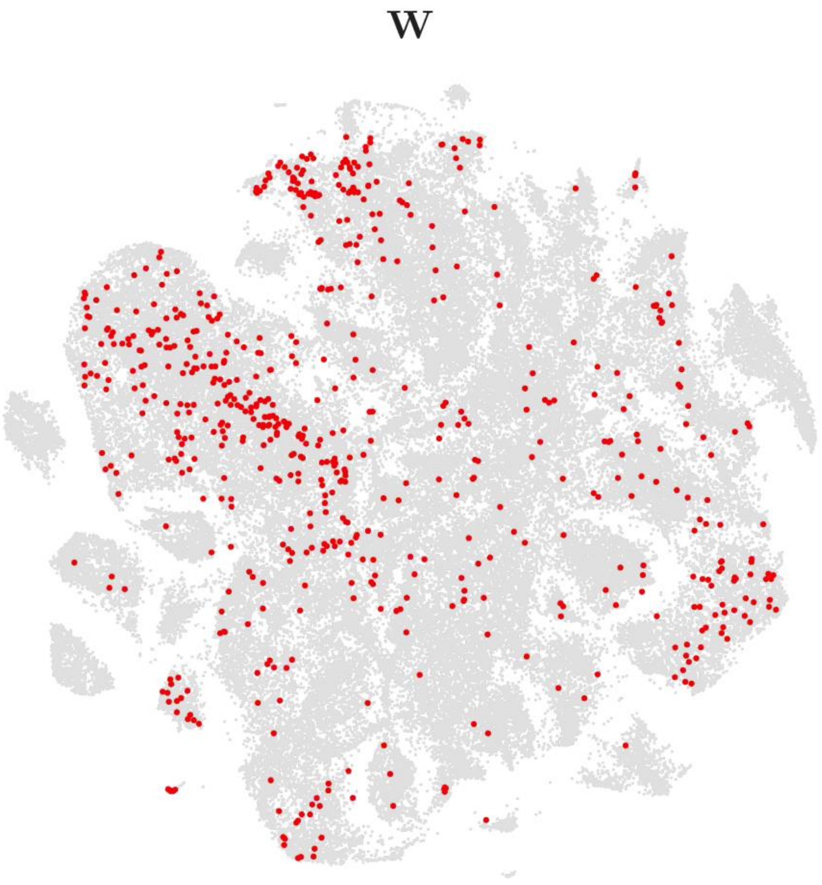

Figure S154. Caption Cluster plot for Rhenium.

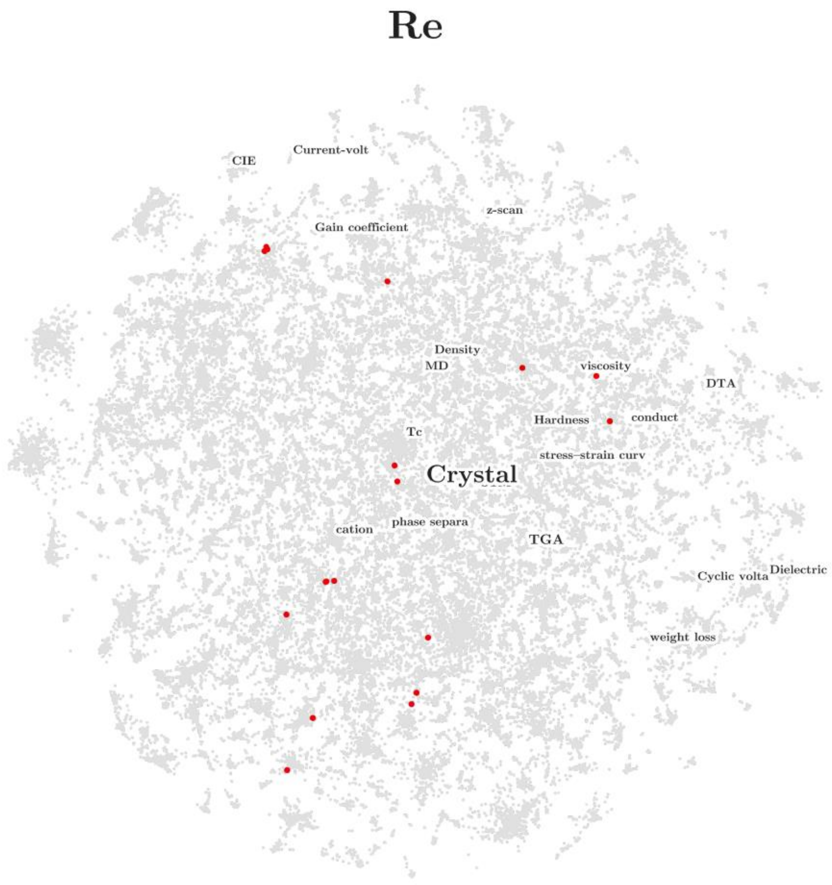

Figure S155. Latent Dirichlet Allocation plot for Rhenium.

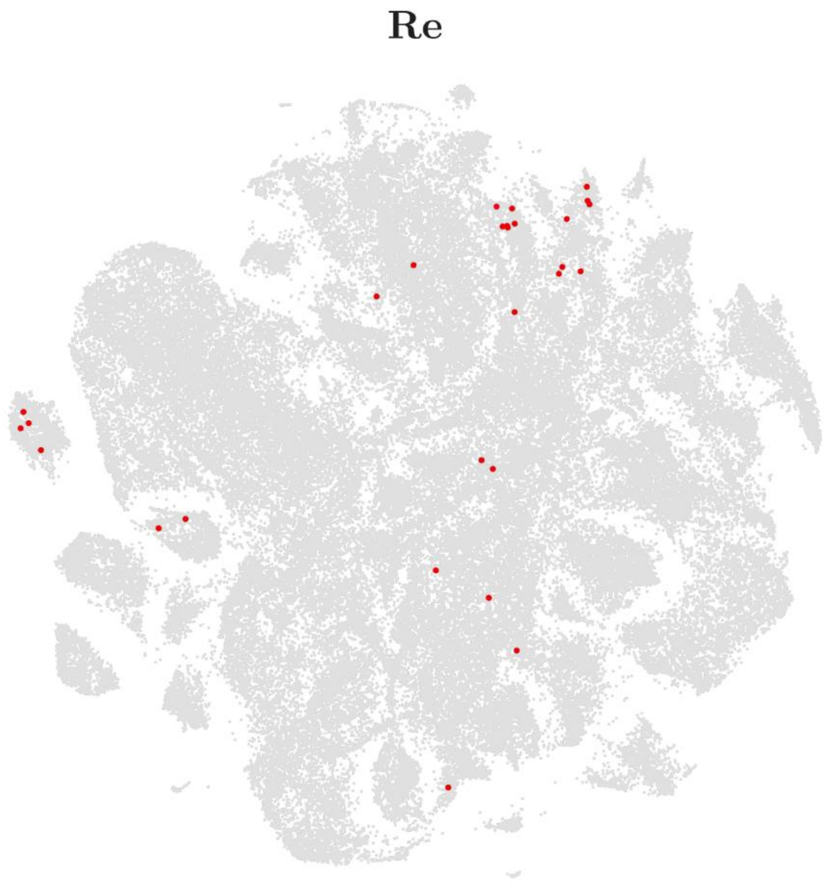

Figure S156. Caption Cluster plot for Osmium.

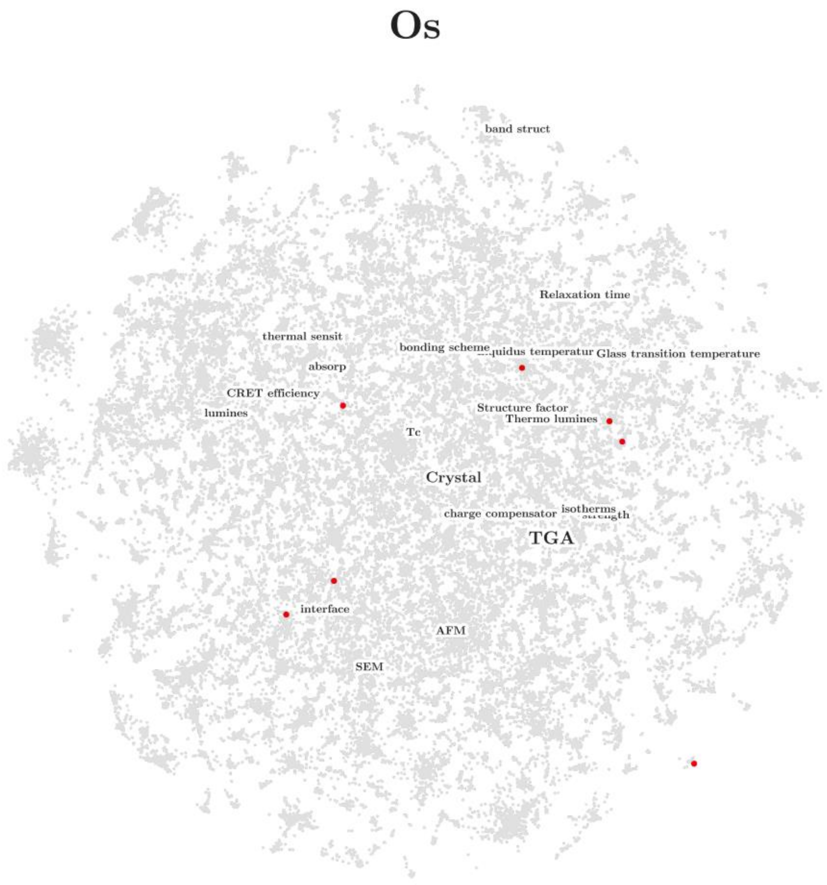

Figure S157. Latent Dirichlet Allocation plot for Osmium.

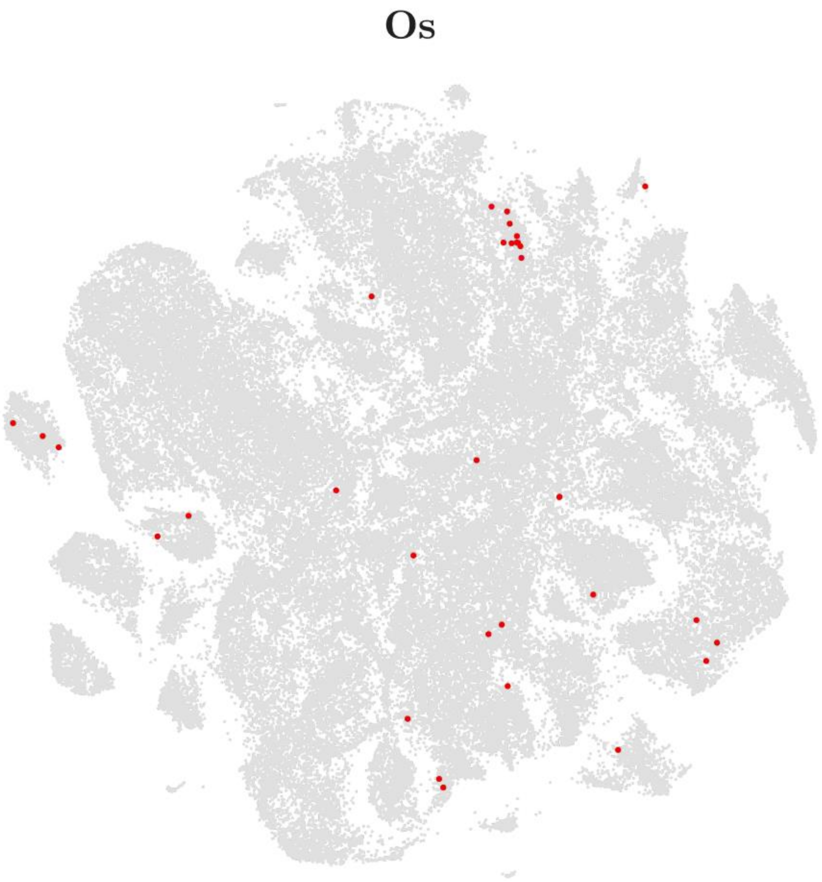

Figure S158. Caption Cluster plot for Iridium.

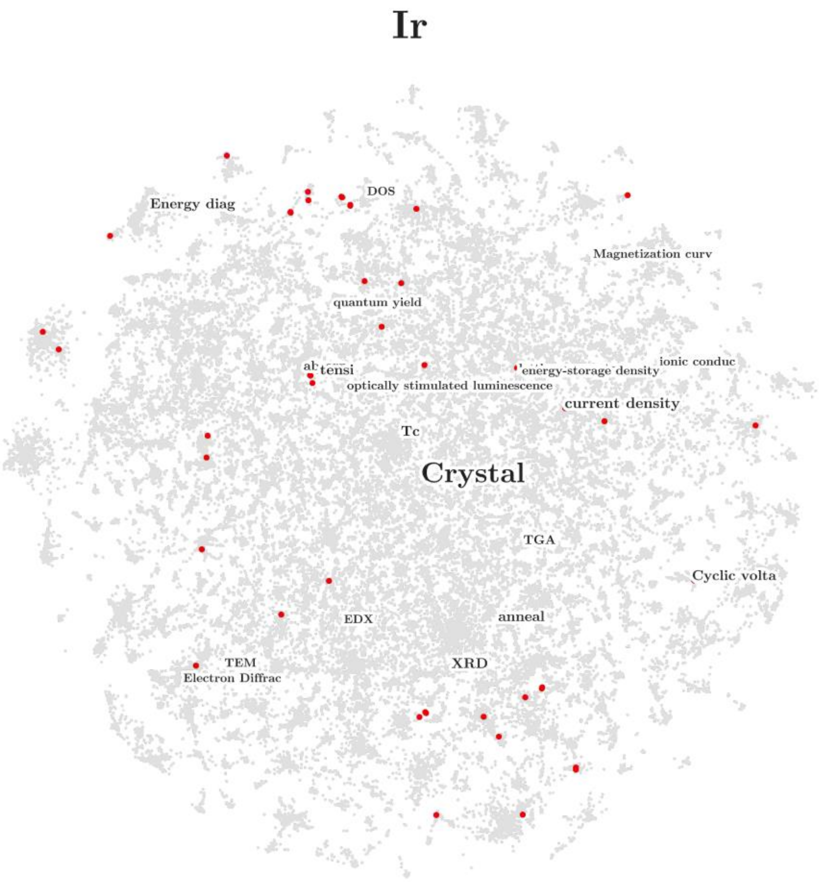

Figure S159. Latent Dirichlet Allocation plot for Iridium.

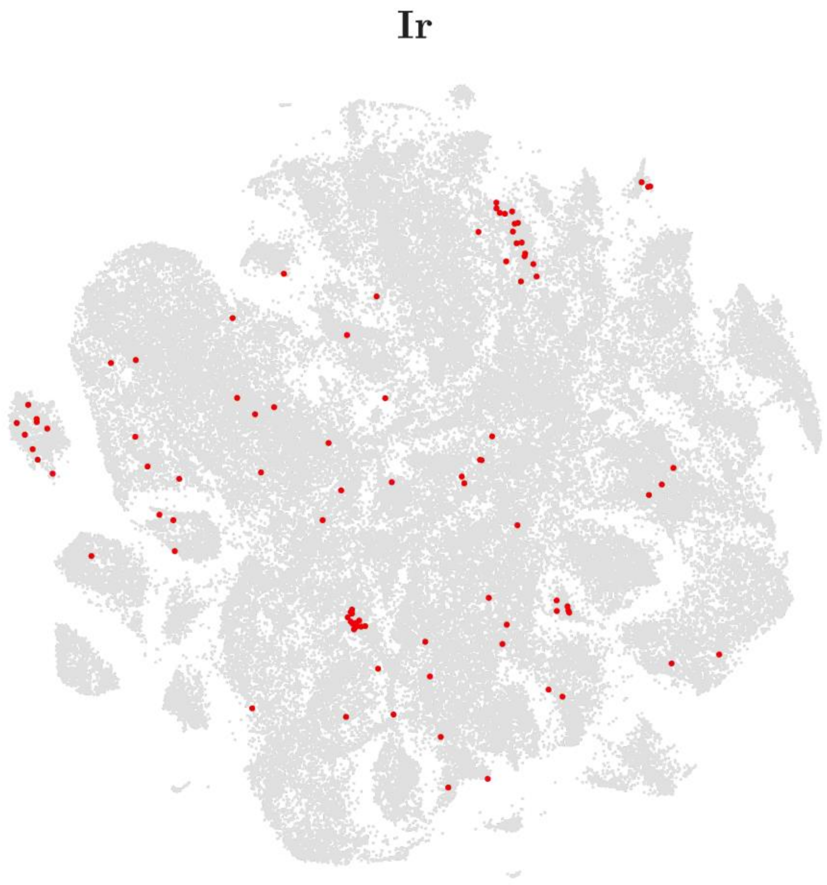

Figure S160. Caption Cluster plot for Platinum.

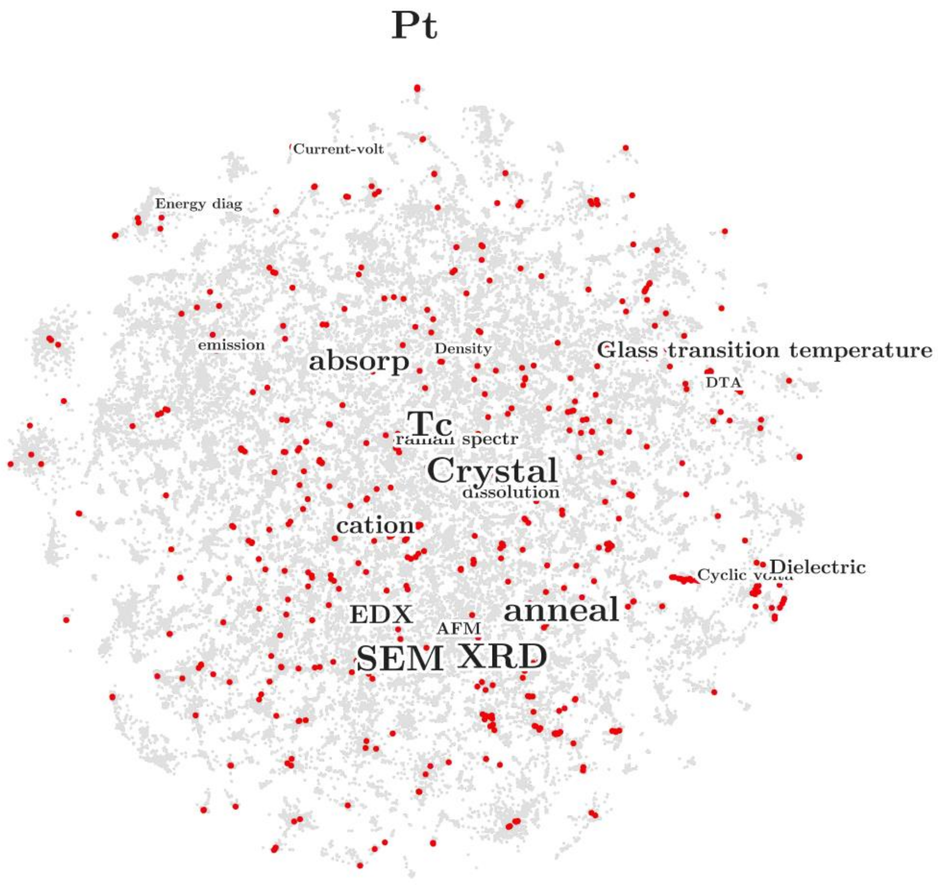

Figure S161. Latent Dirichlet Allocation plot for Platinum.

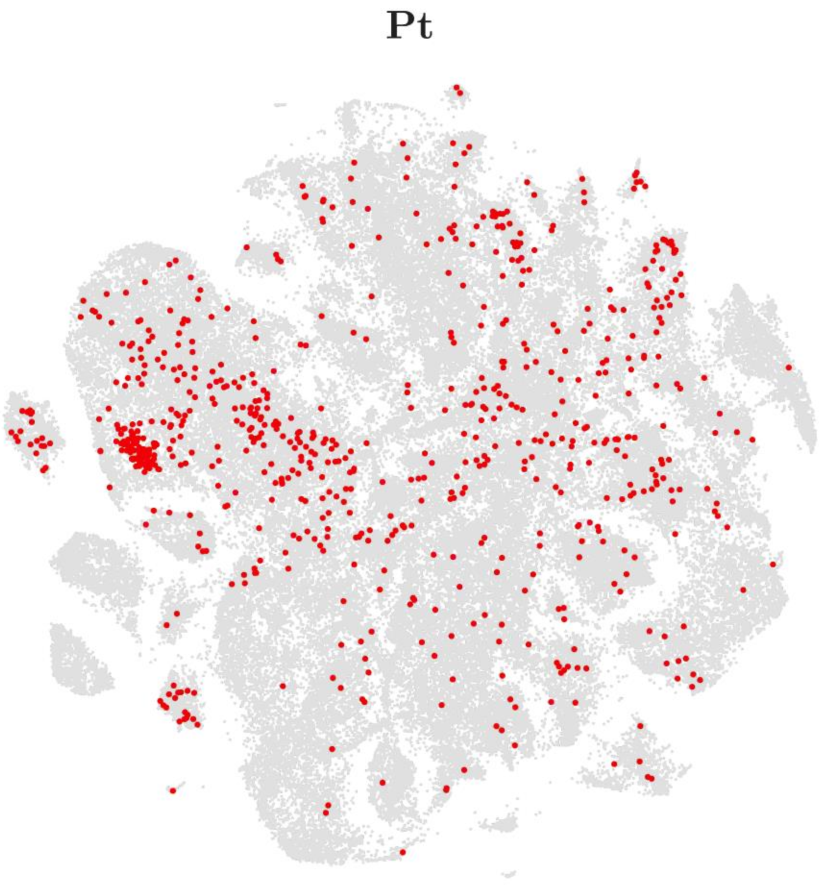

Figure S162. Caption Cluster plot for Gold.

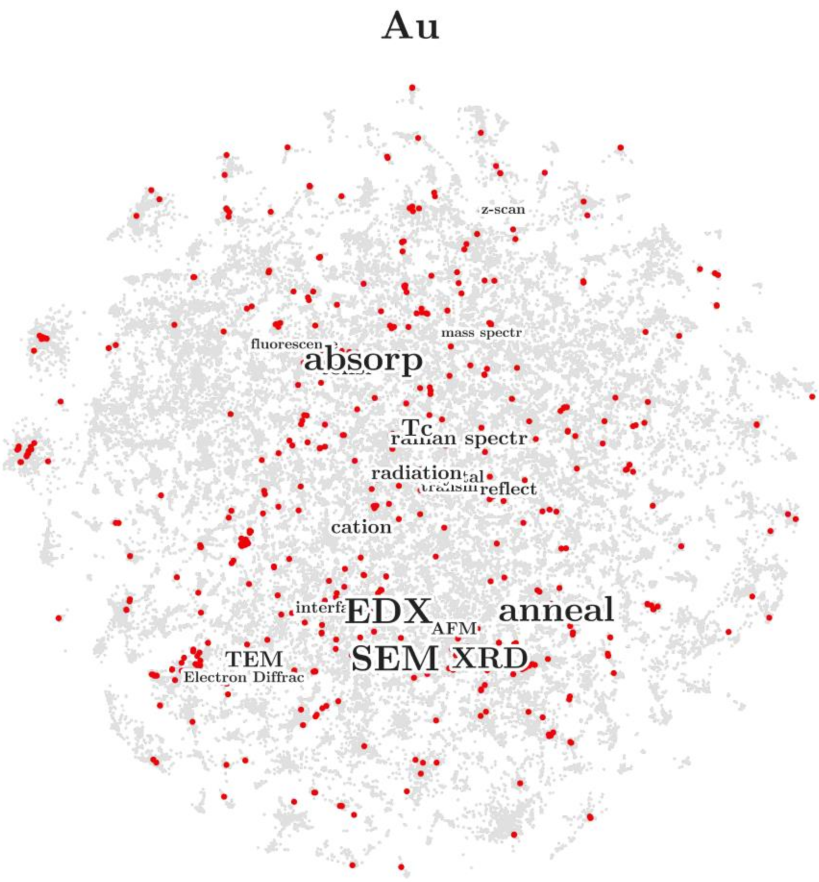

Figure S163. Latent Dirichlet Allocation plot for Gold.

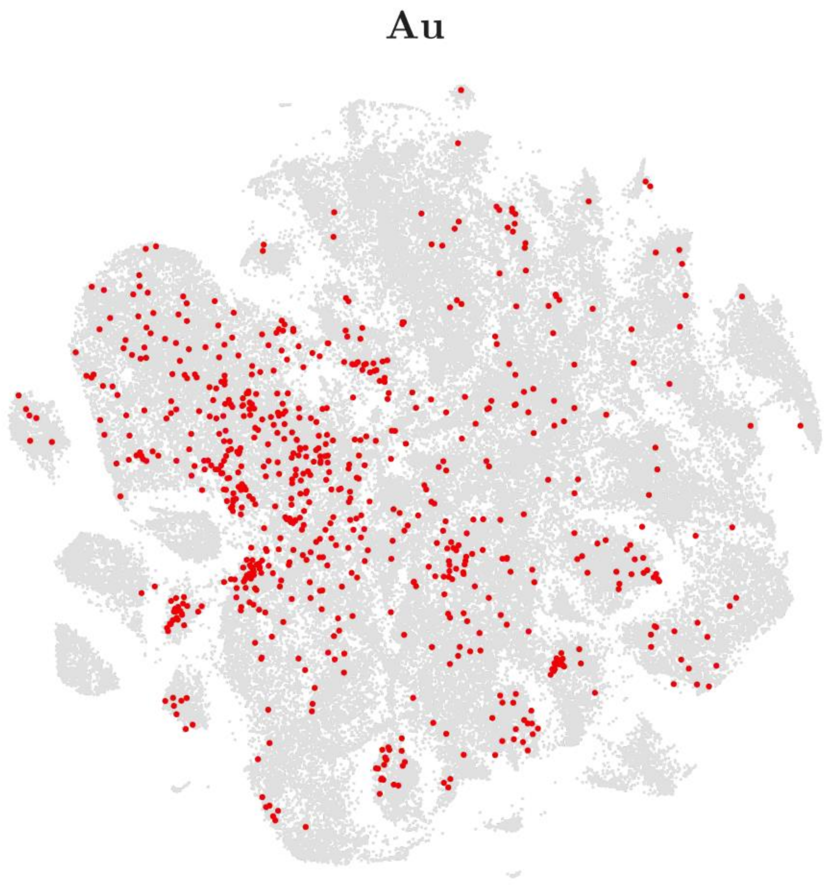

Figure S164. Caption Cluster plot for Mercury.

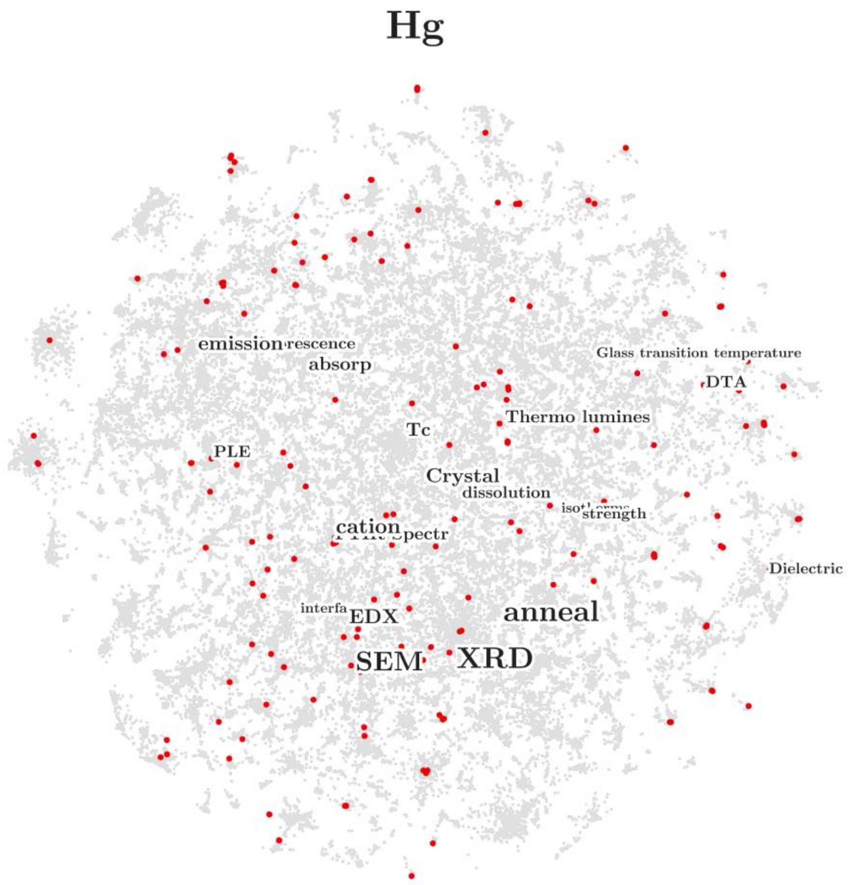

Figure S165. Latent Dirichlet Allocation plot for Mercury.

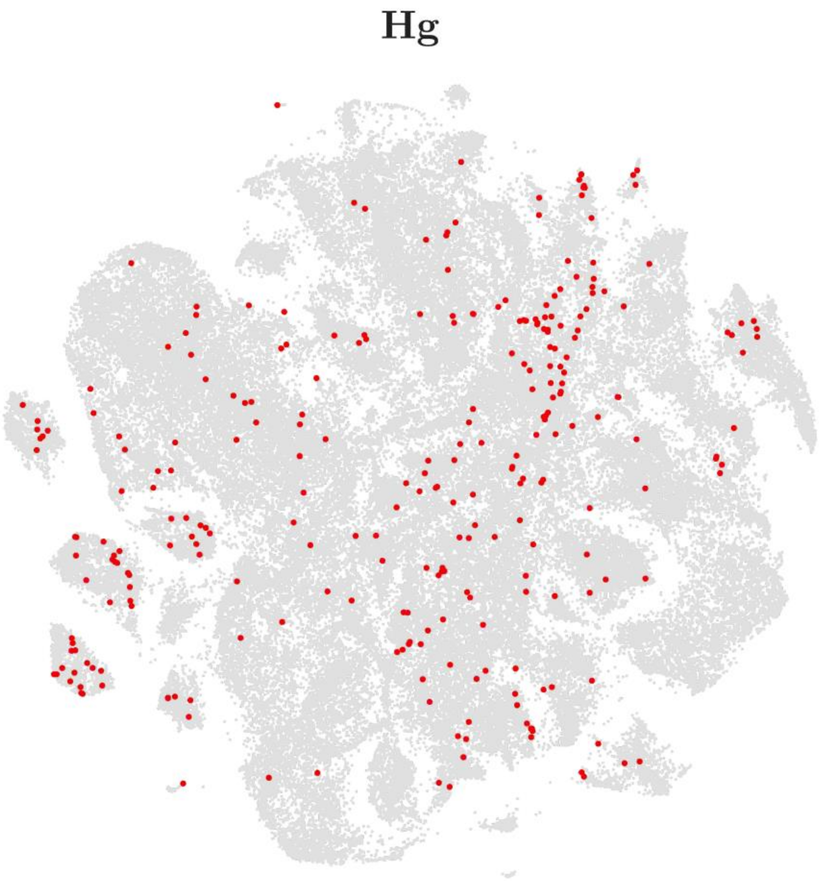

Figure S166. Caption Cluster plot for Thallium.

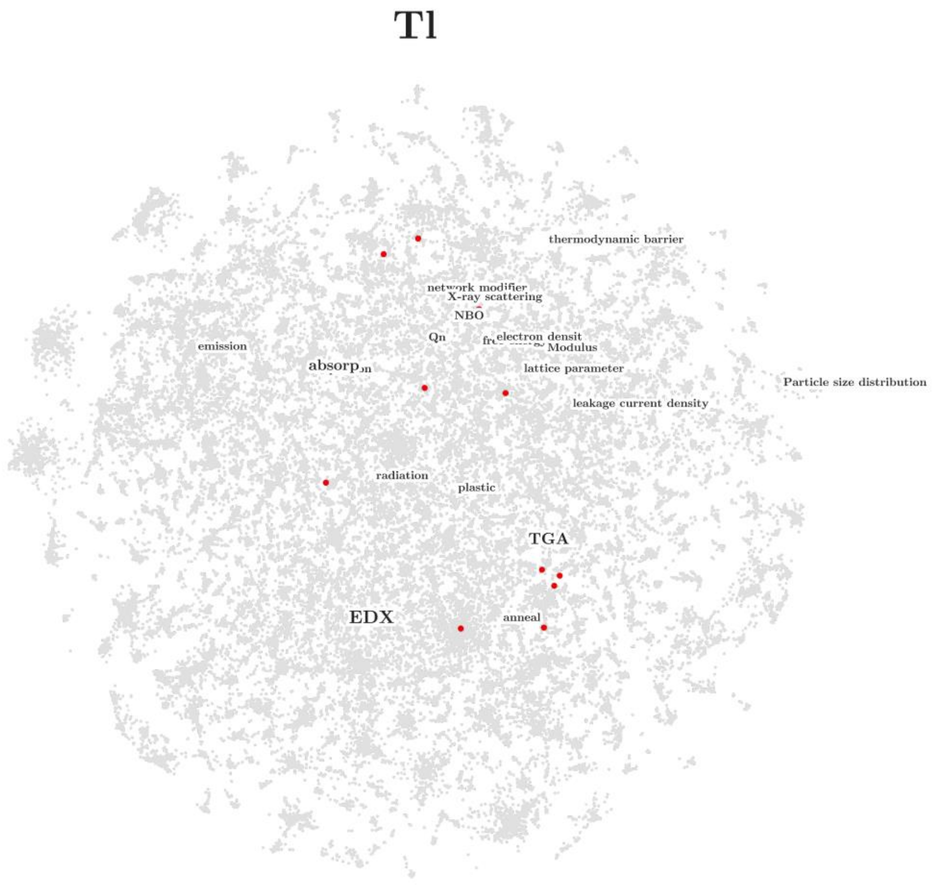

Figure S167. Latent Dirichlet Allocation plot for Thallium.

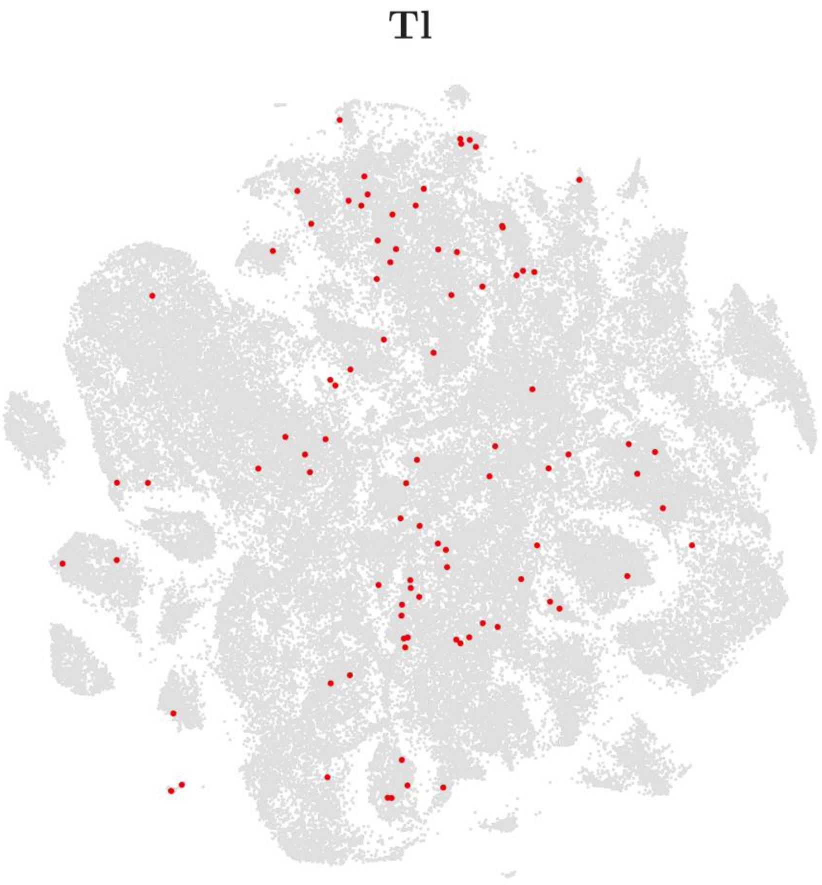

Figure S168. Caption Cluster plot for Lead.

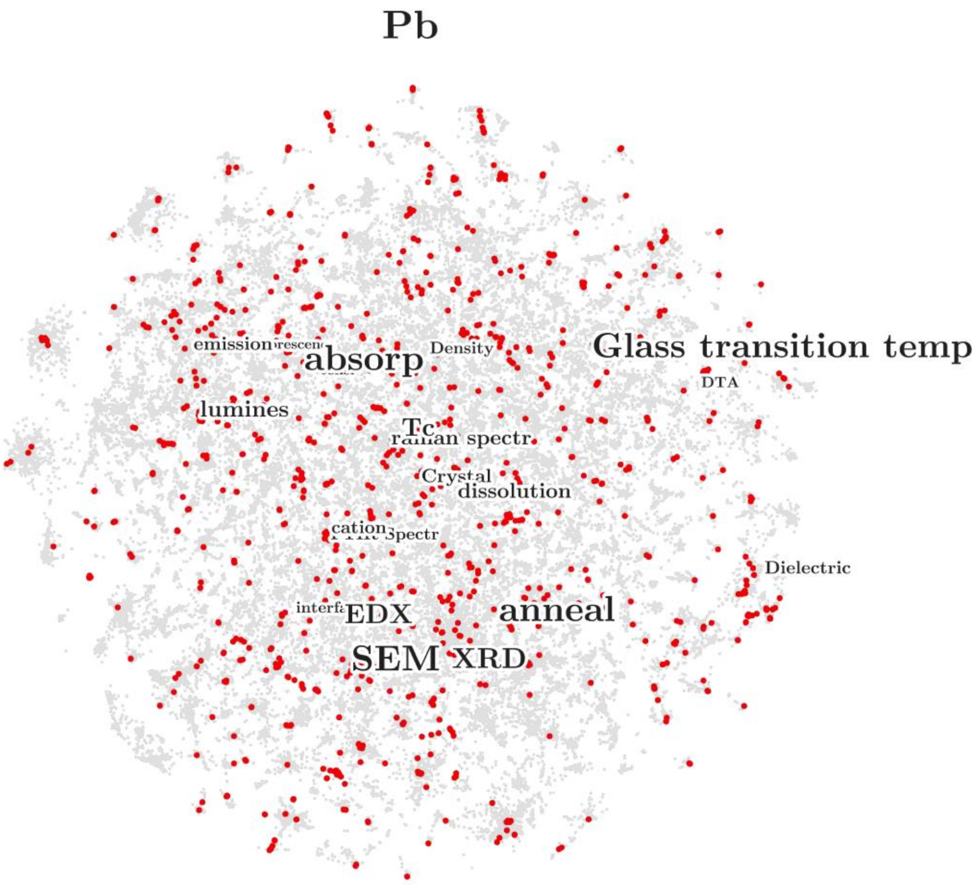

Figure S169. Latent Dirichlet Allocation plot for Lead.

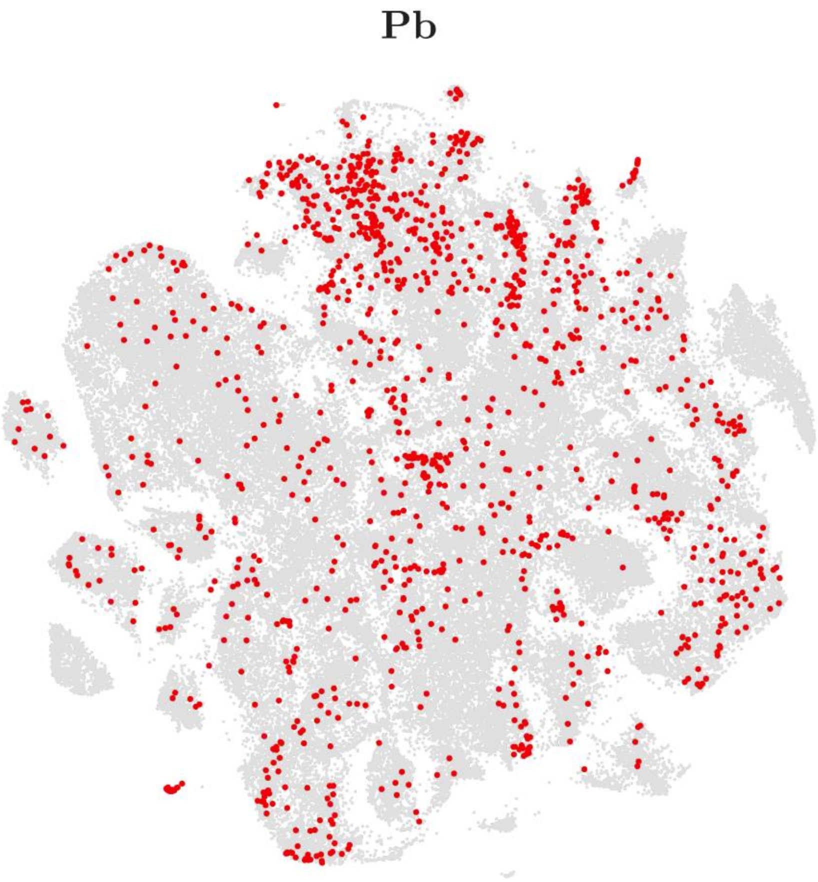

Figure S170. Caption Cluster plot for Bismuth.

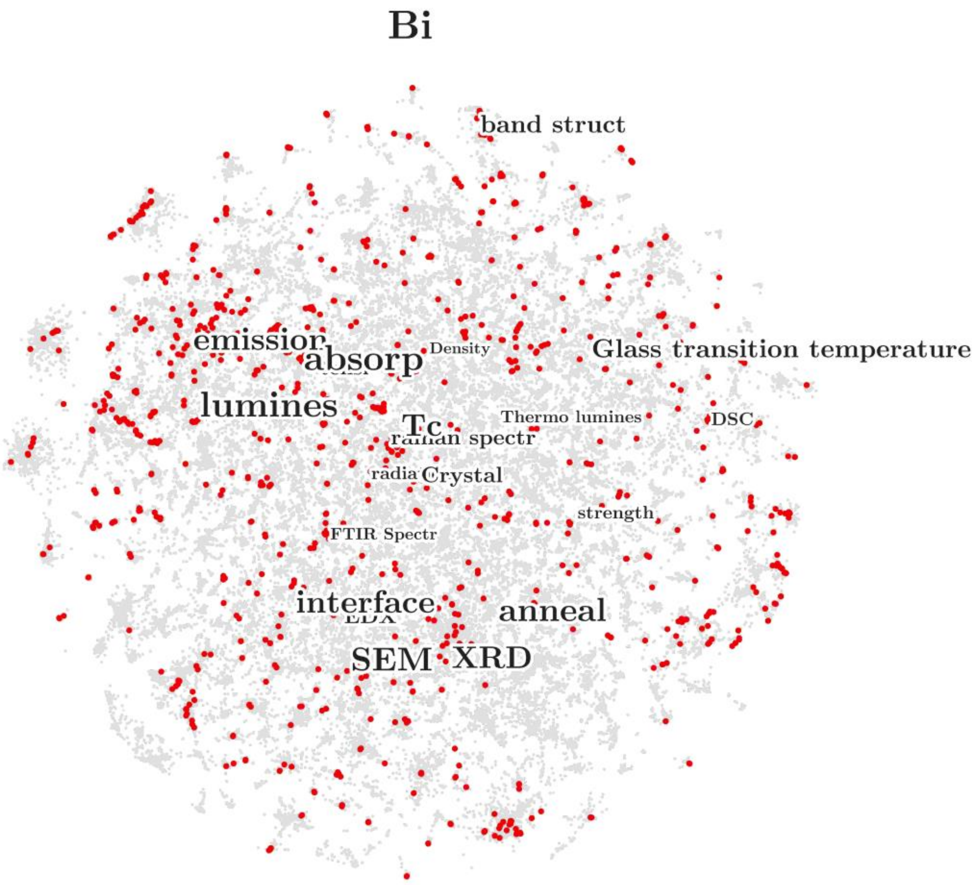

Figure S171. Latent Dirichlet Allocation plot for Bismuth.

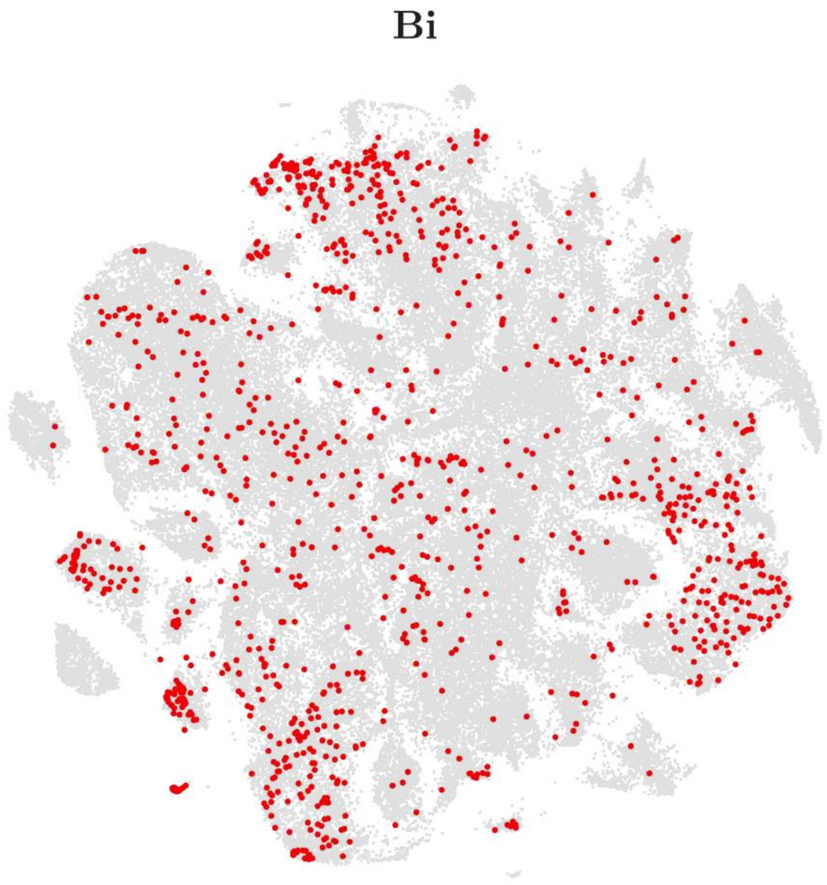

Figure S172. Caption Cluster plot for Polonium.

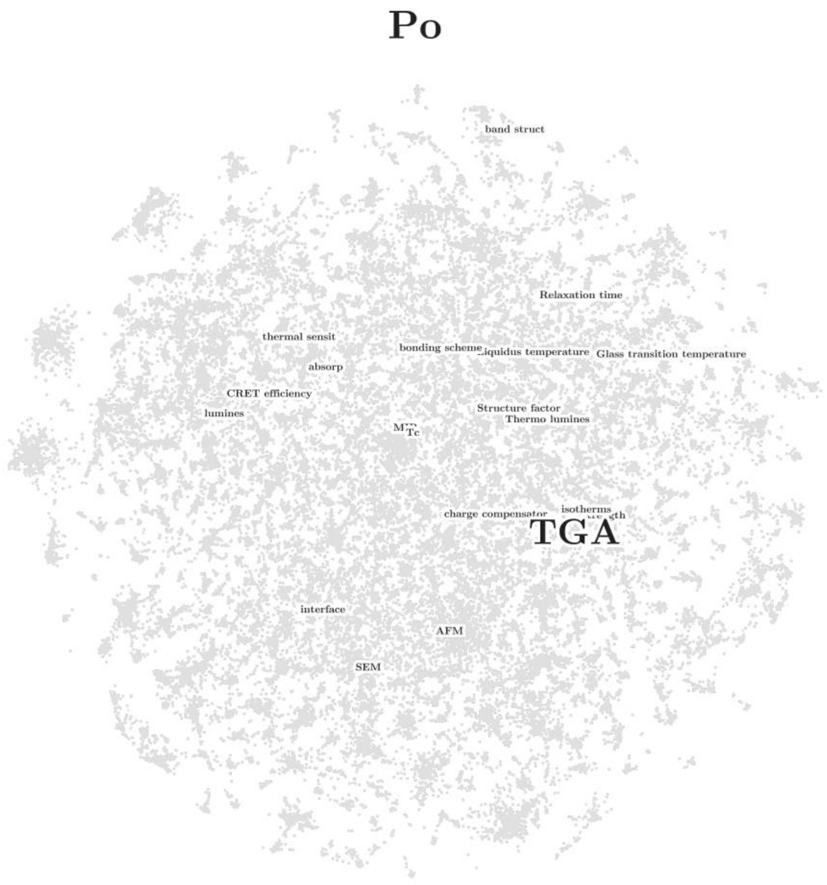

Figure S173. Latent Dirichlet Allocation plot for Polonium.

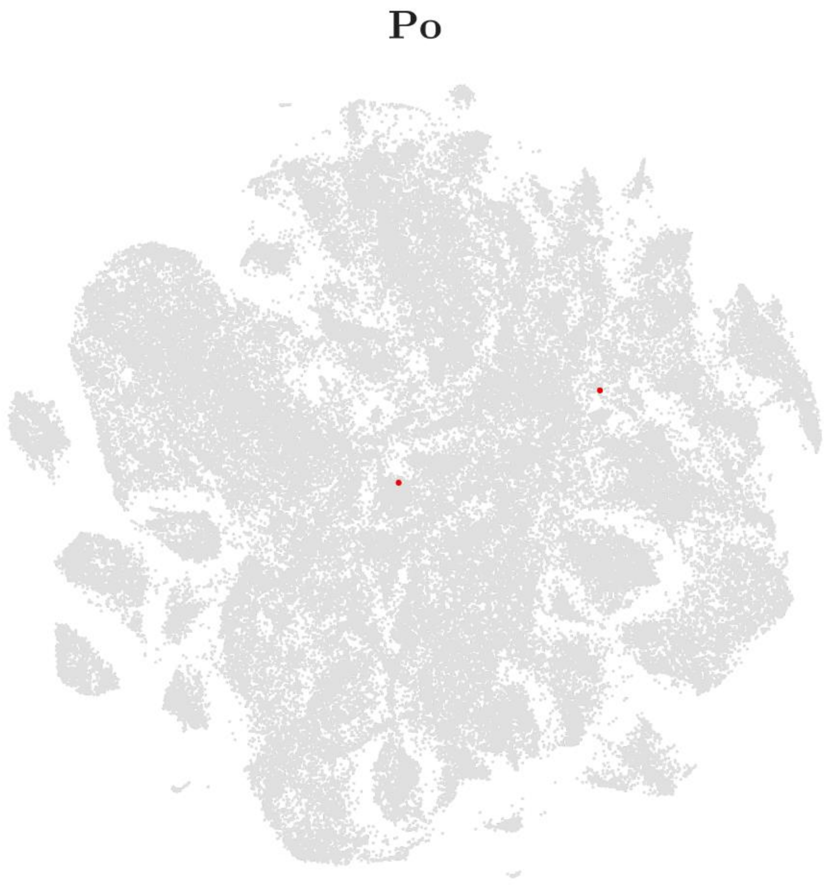

Figure S174. Caption Cluster plot for Astatine.

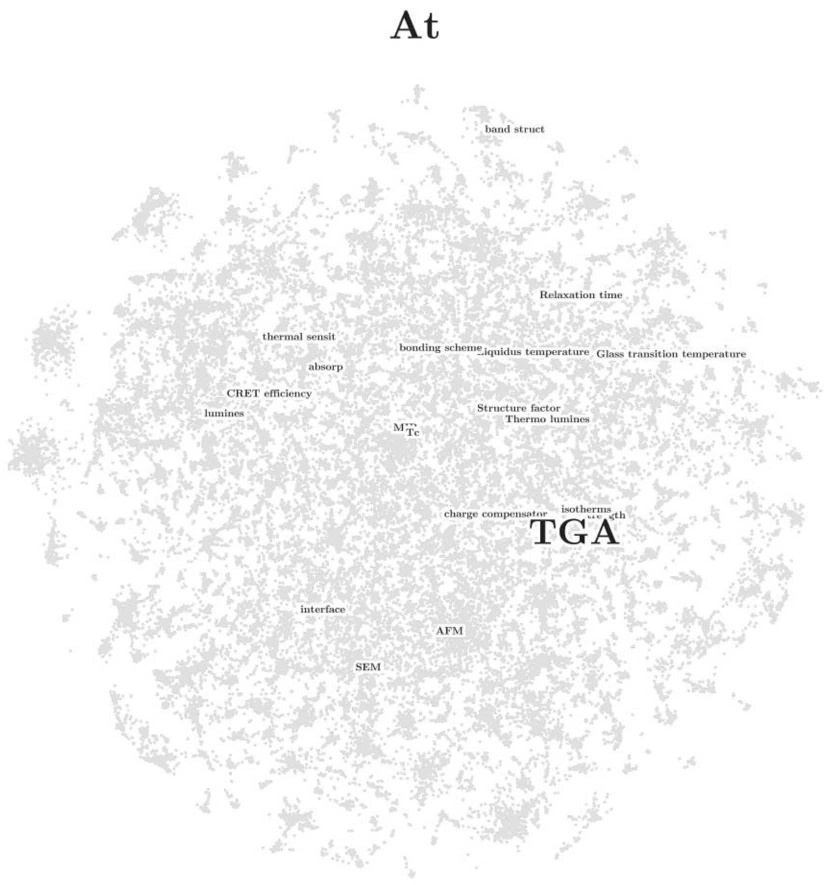

Figure S175. Latent Dirichlet Allocation plot for Astatine.

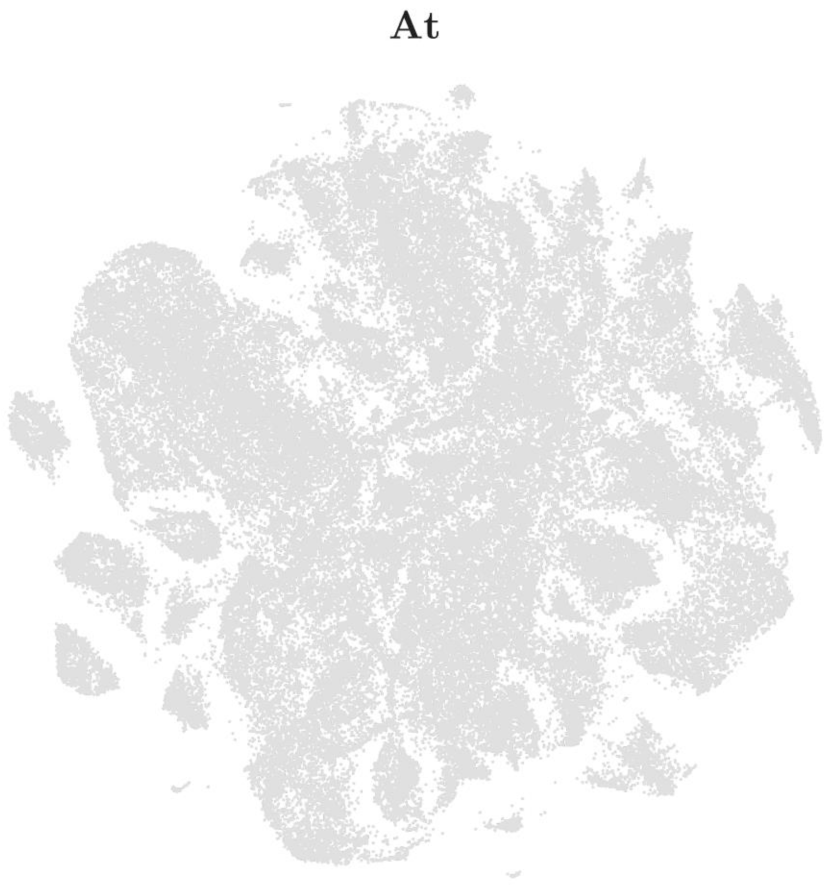

Figure S176. Caption Cluster plot for Radon.

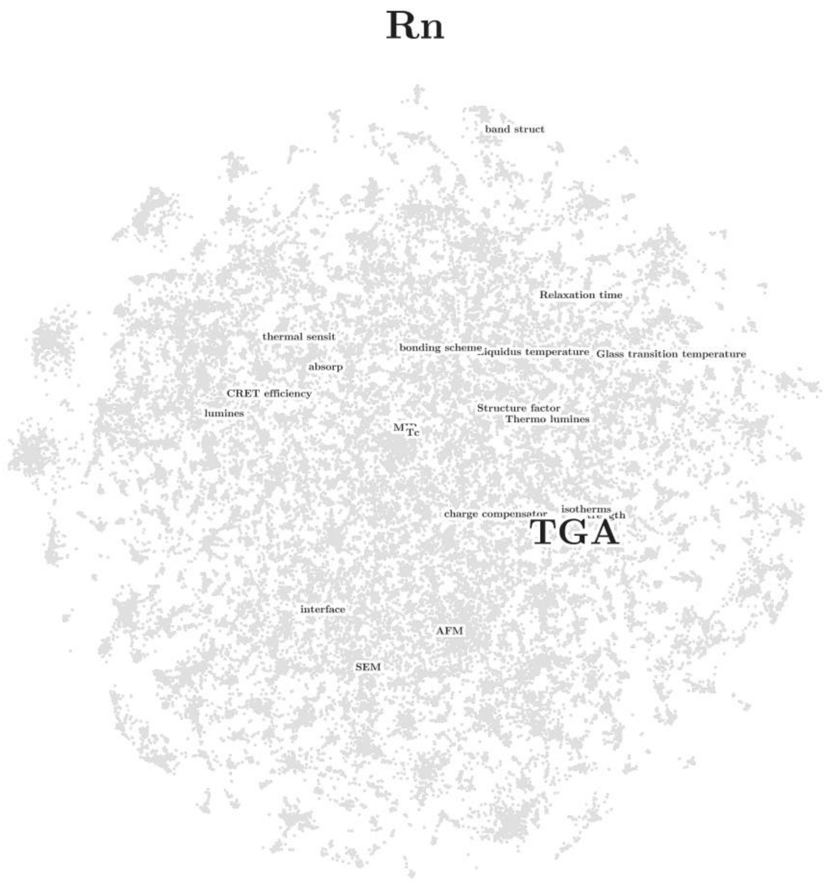

Figure S177. Latent Dirichlet Allocation plot for Radon.

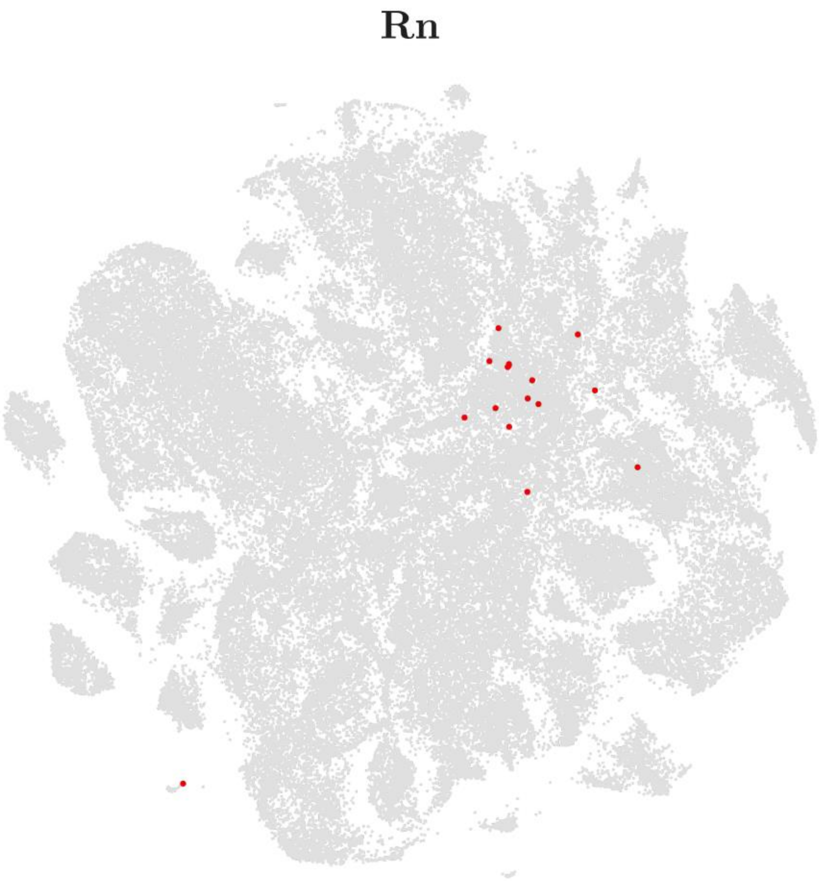

Figure S178. Caption Cluster plot for Francium.

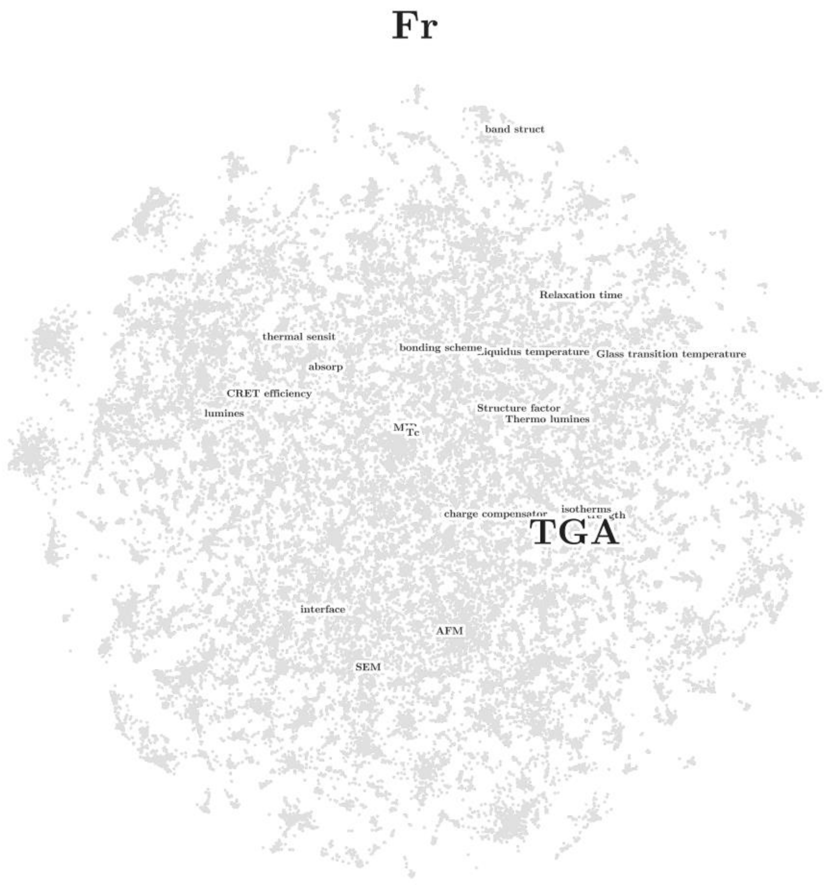

Figure S179. Latent Dirichlet Allocation plot for Francium.

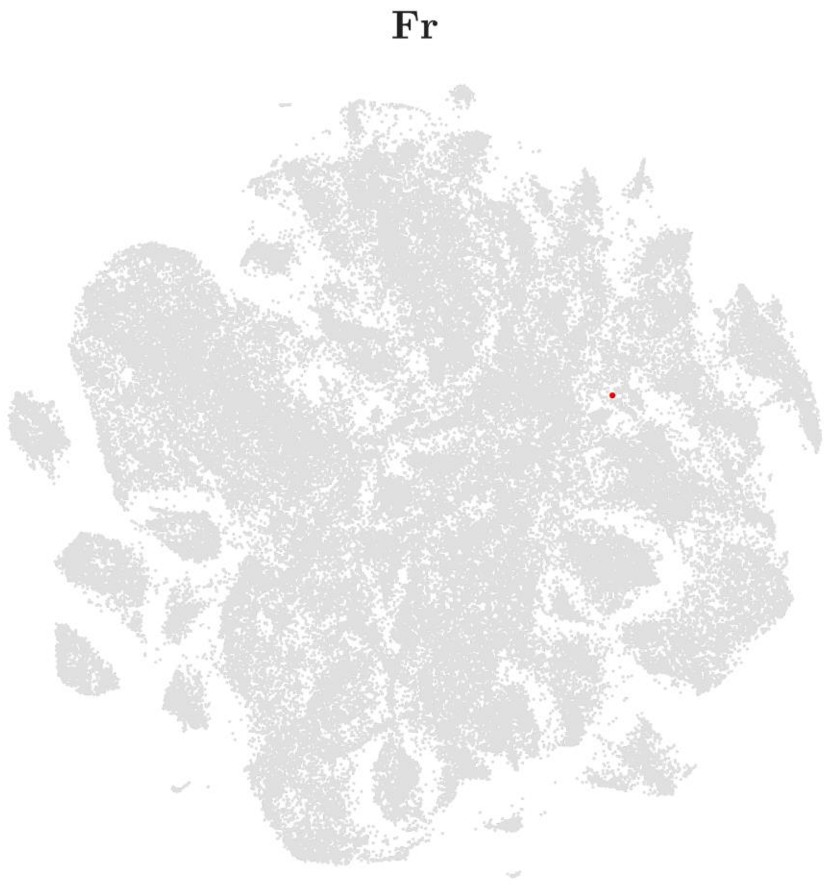

Figure S180. Caption Cluster plot for Radium.

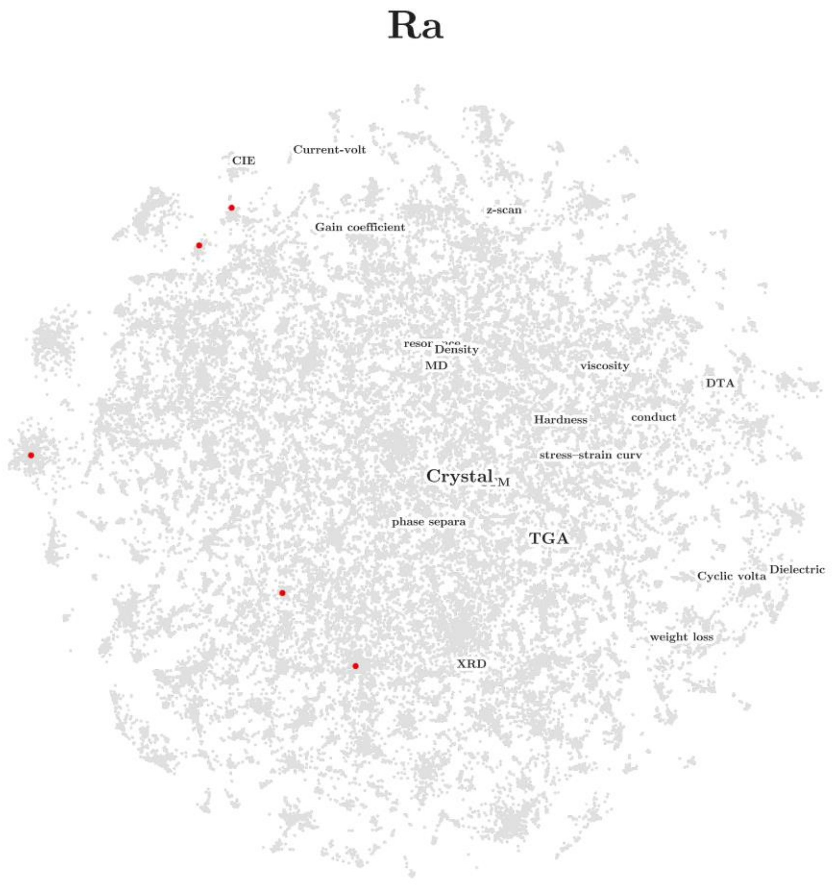

Figure S181. Latent Dirichlet Allocation plot for Radium.

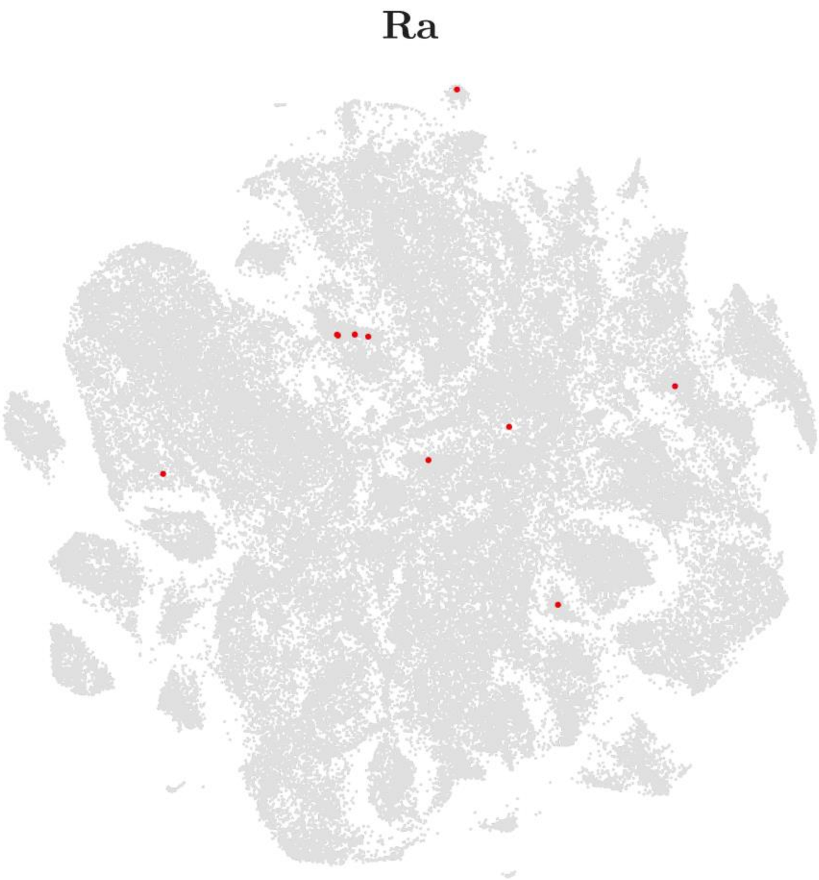

Figure S182. Caption Cluster plot for Actinium.

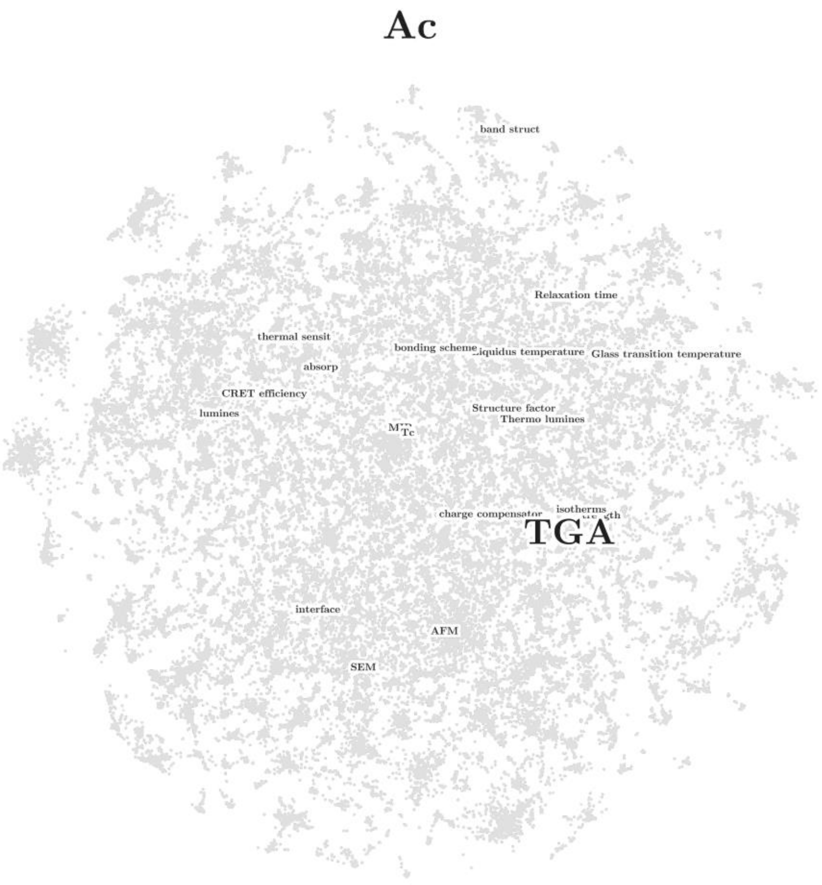

Figure S183. Latent Dirichlet Allocation plot for Actinium.

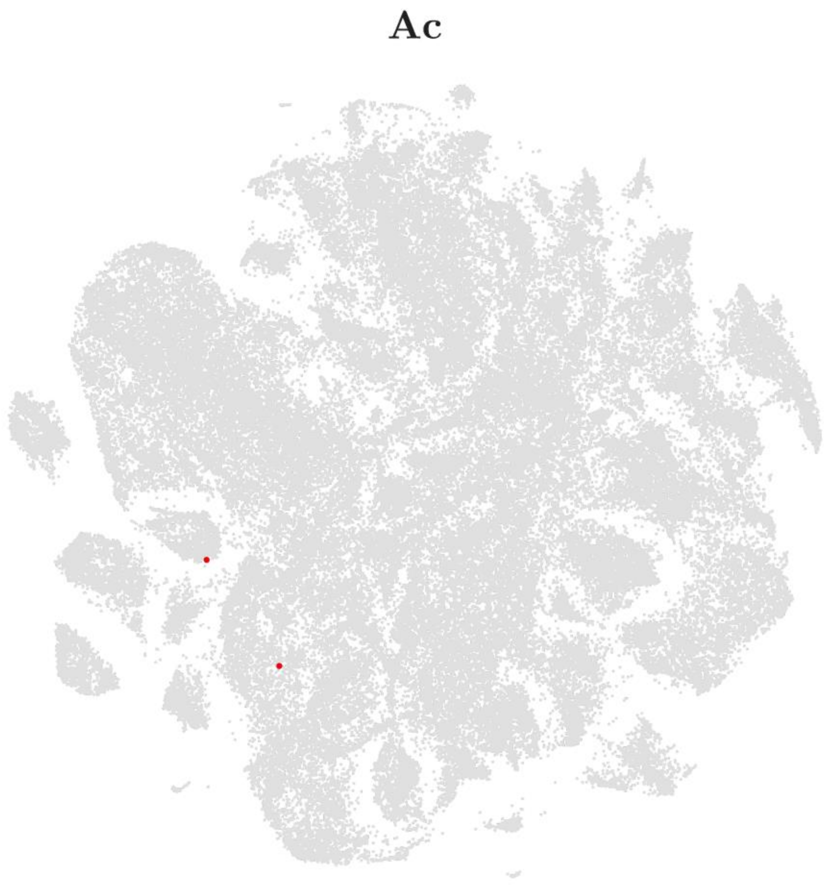

Figure S184. Caption Cluster plot for Thorium.

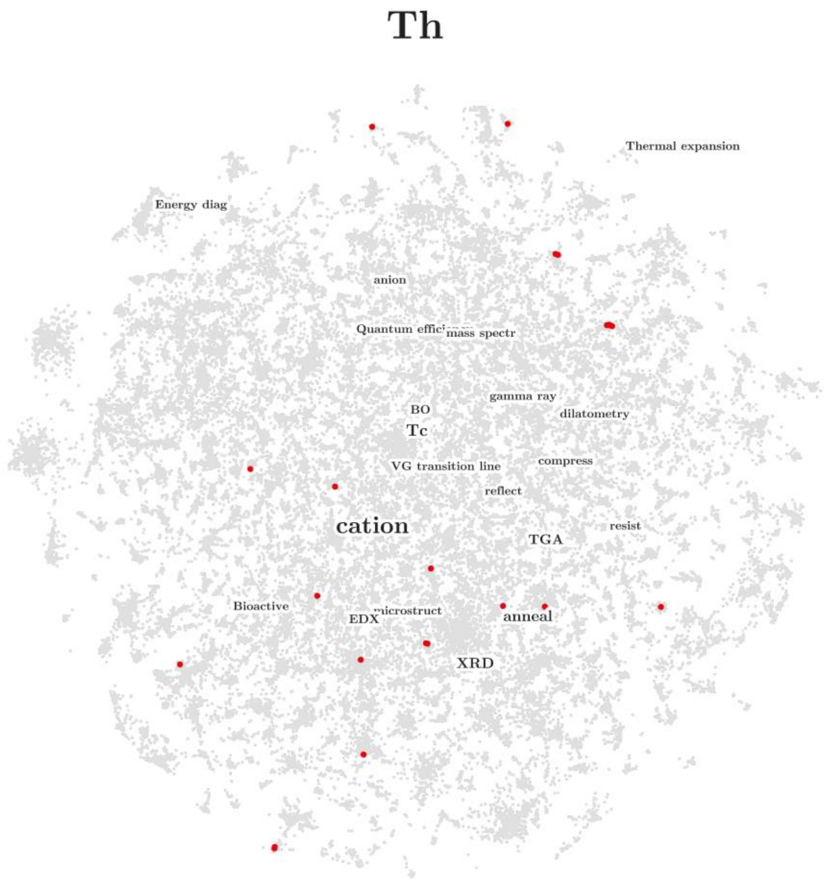

Figure S185. Latent Dirichlet Allocation plot for Thorium.

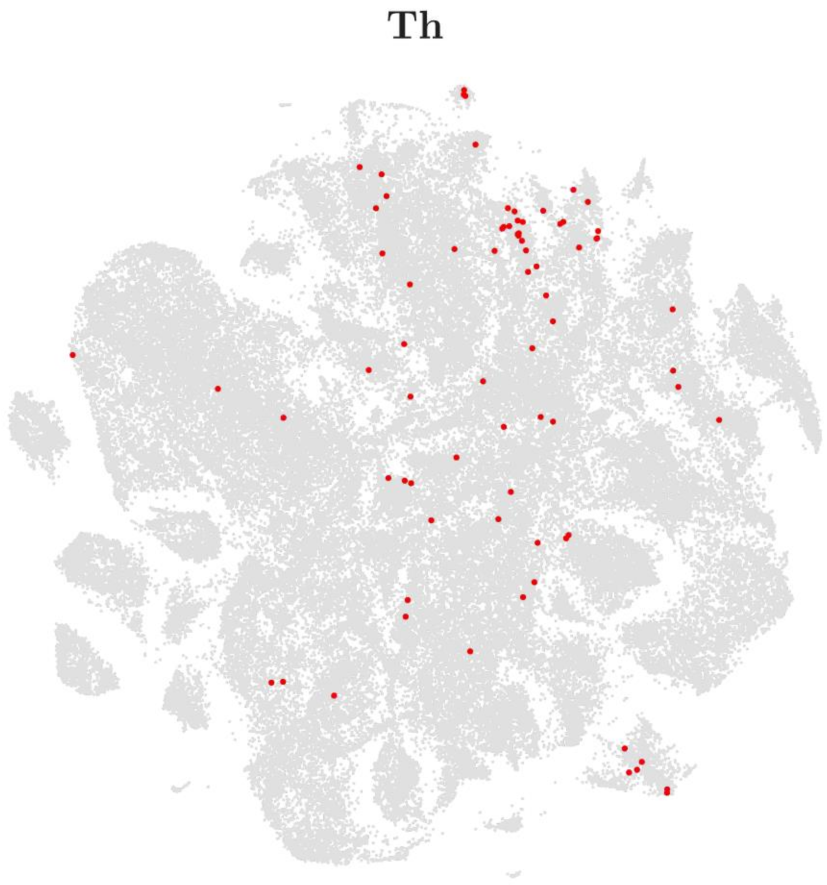

Figure S186. Caption Cluster plot for Protactinium.

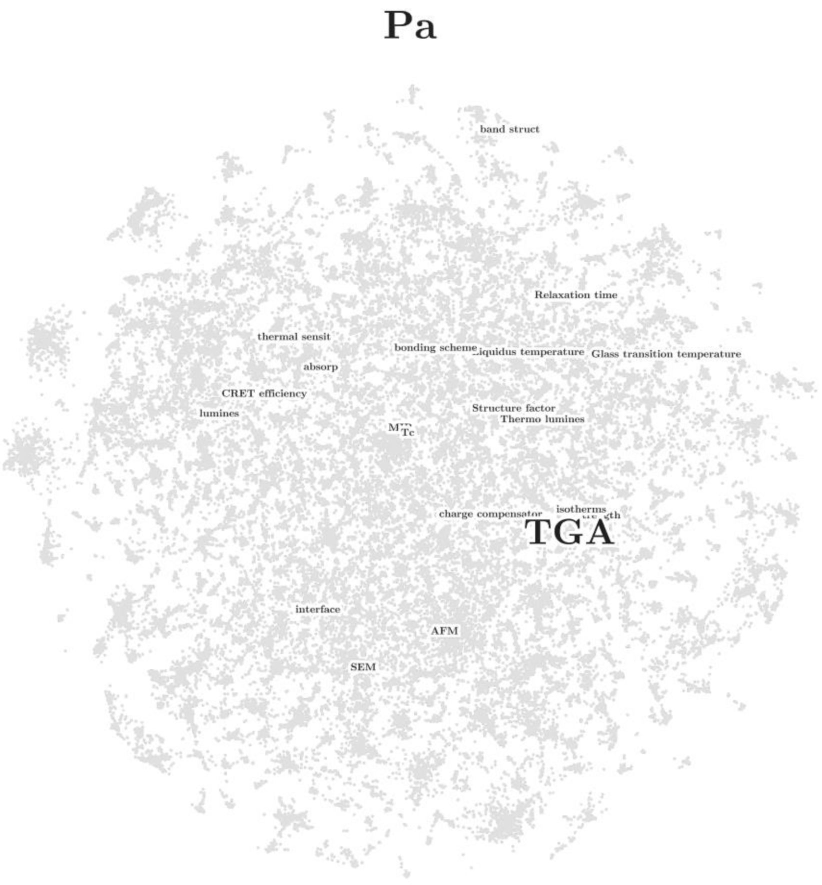

Figure S187. Latent Dirichlet Allocation plot for Protactinium.

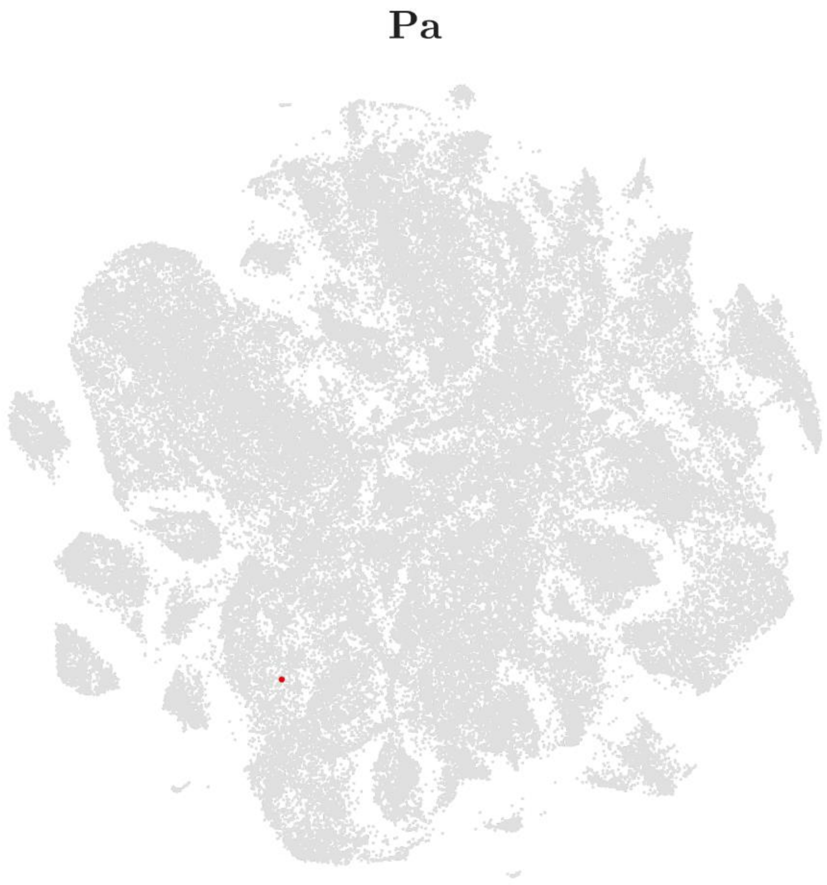

Figure S188. Caption Cluster plot for Uranium.

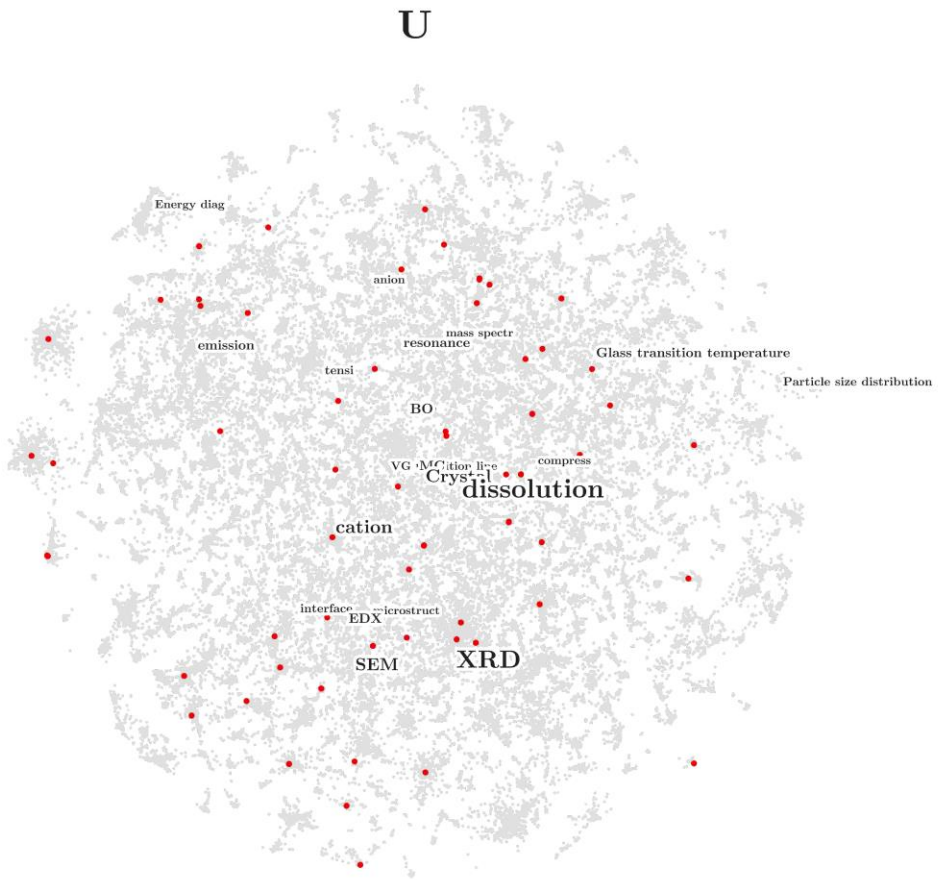

Figure S189. Latent Dirichlet Allocation plot for Uranium.

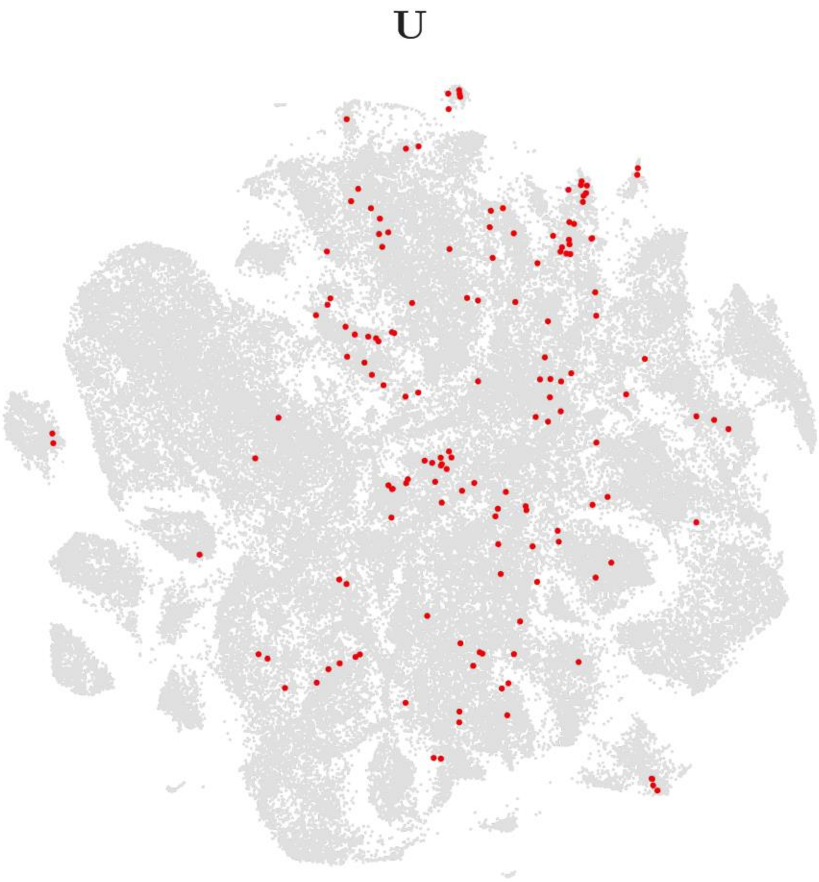

Figure S190. Caption Cluster plot for Neptunium.

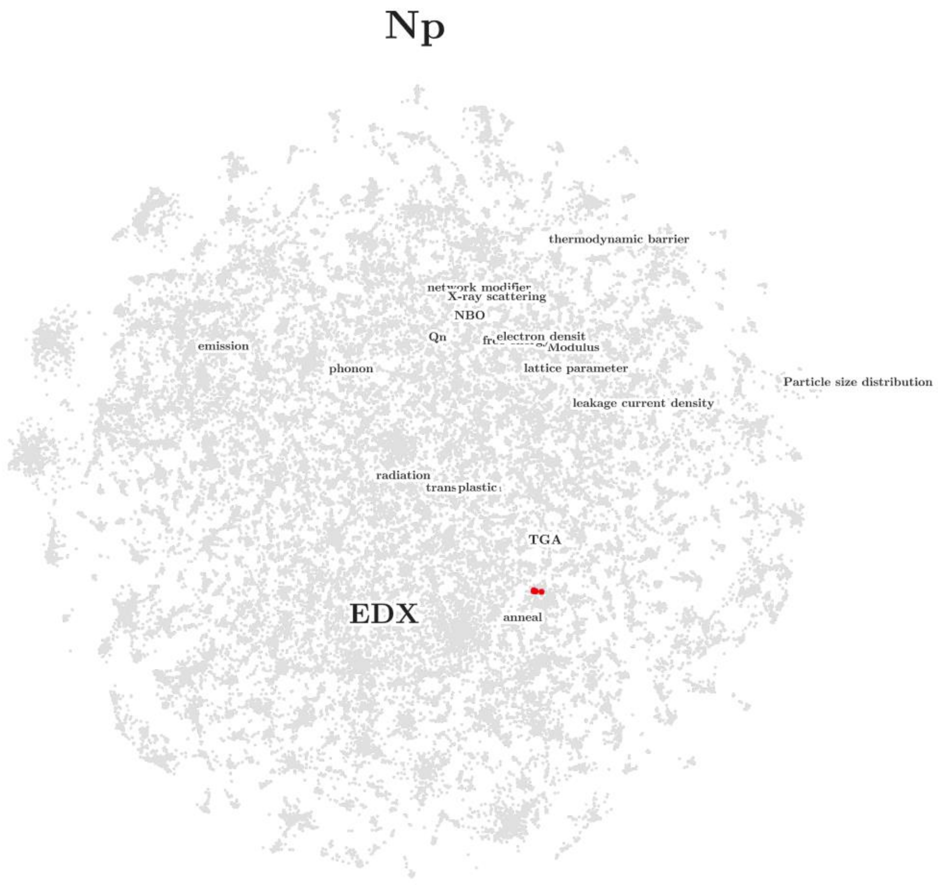

Figure S191. Latent Dirichlet Allocation plot for Neptunium.

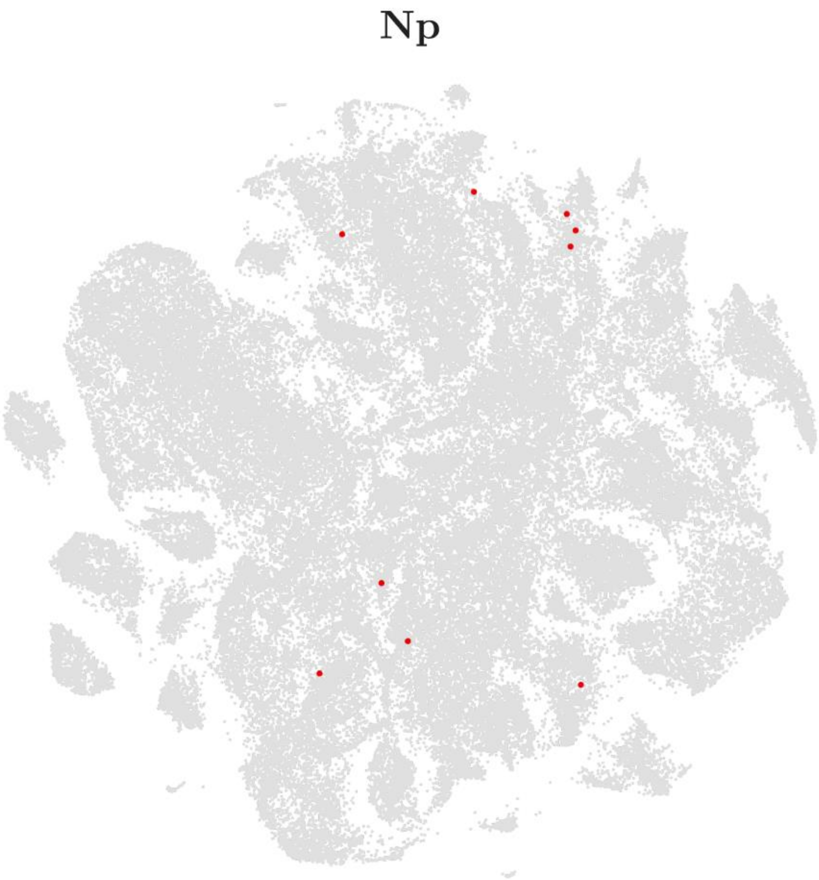

Figure S192. Caption Cluster plot for Plutonium.

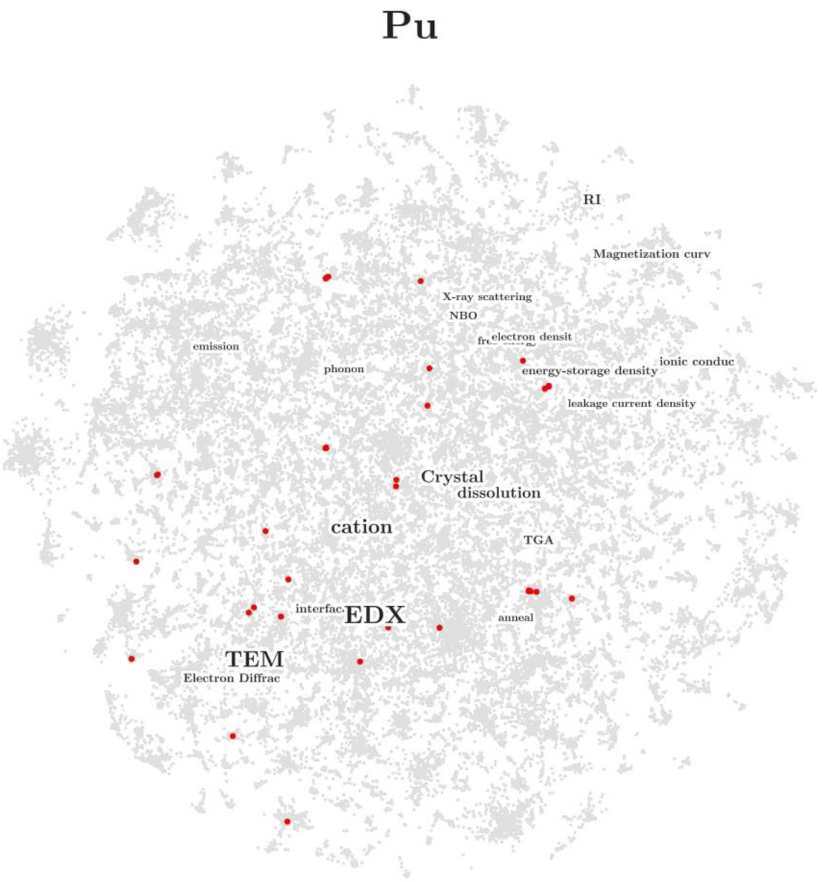

Figure S193. Latent Dirichlet Allocation plot for Plutonium.

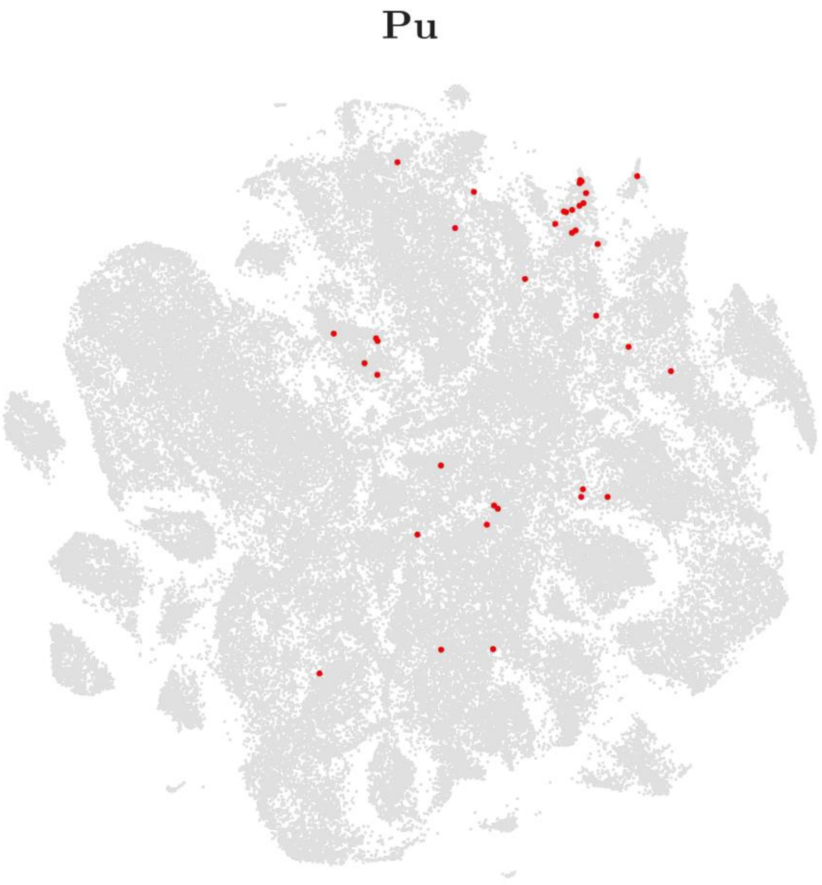

Figure S194. Caption Cluster plot for Americium.

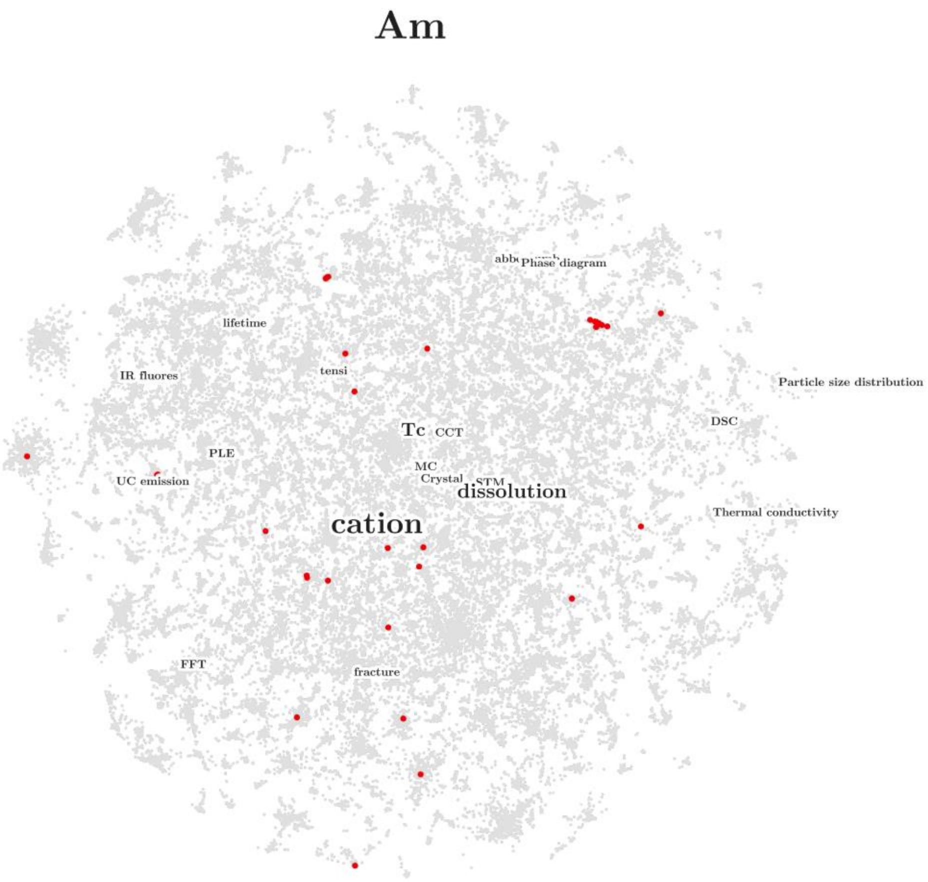

Figure S195. Latent Dirichlet Allocation plot for Americium.

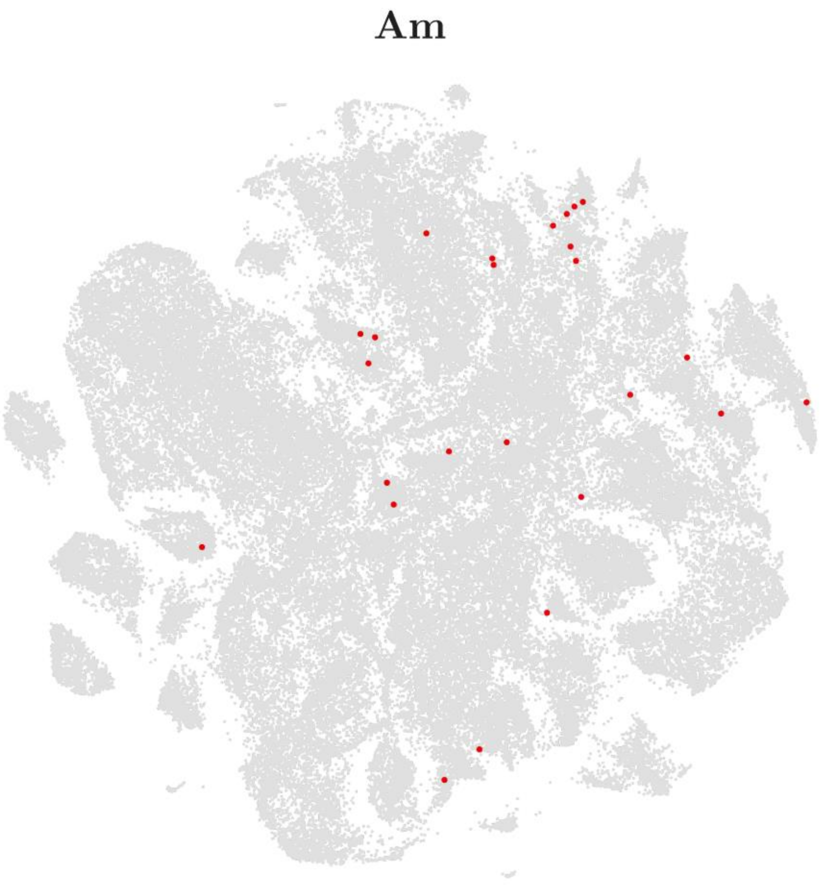

Figure S196. Caption Cluster plot for Curium.

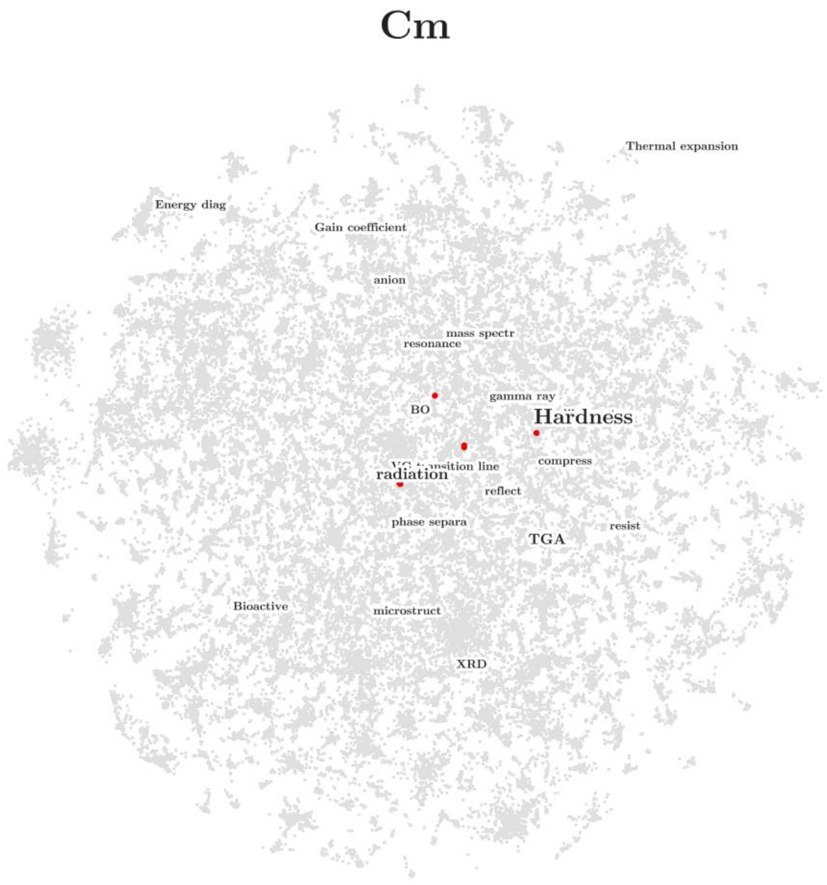

Figure S197. Latent Dirichlet Allocation plot for Curium.

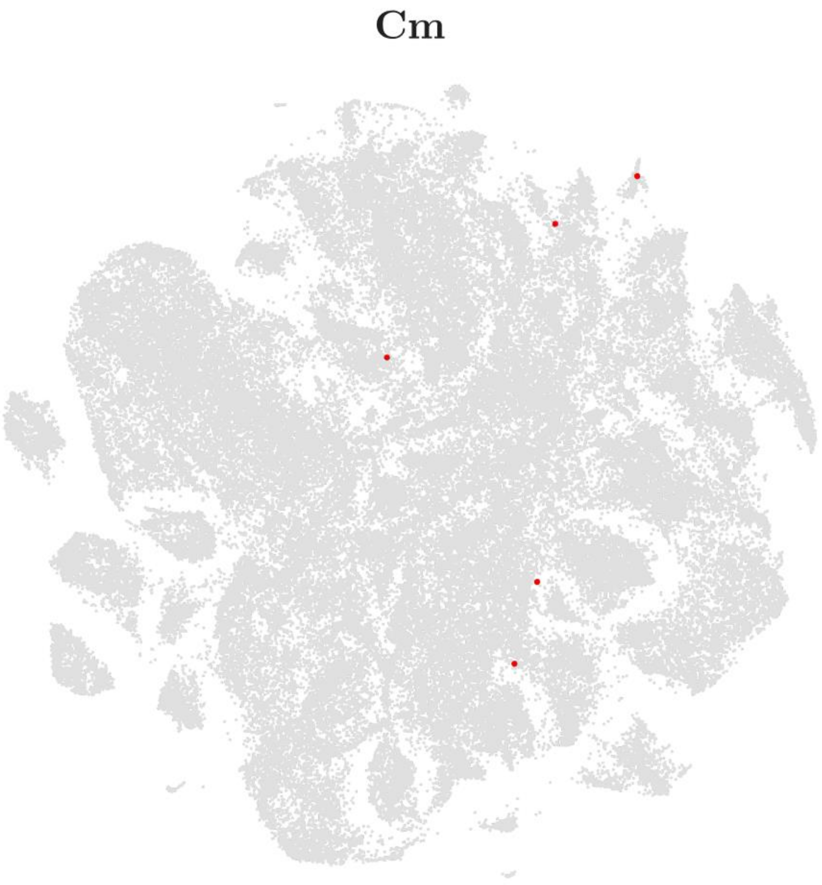

Figure S198. Caption Cluster plot for Berkelium.

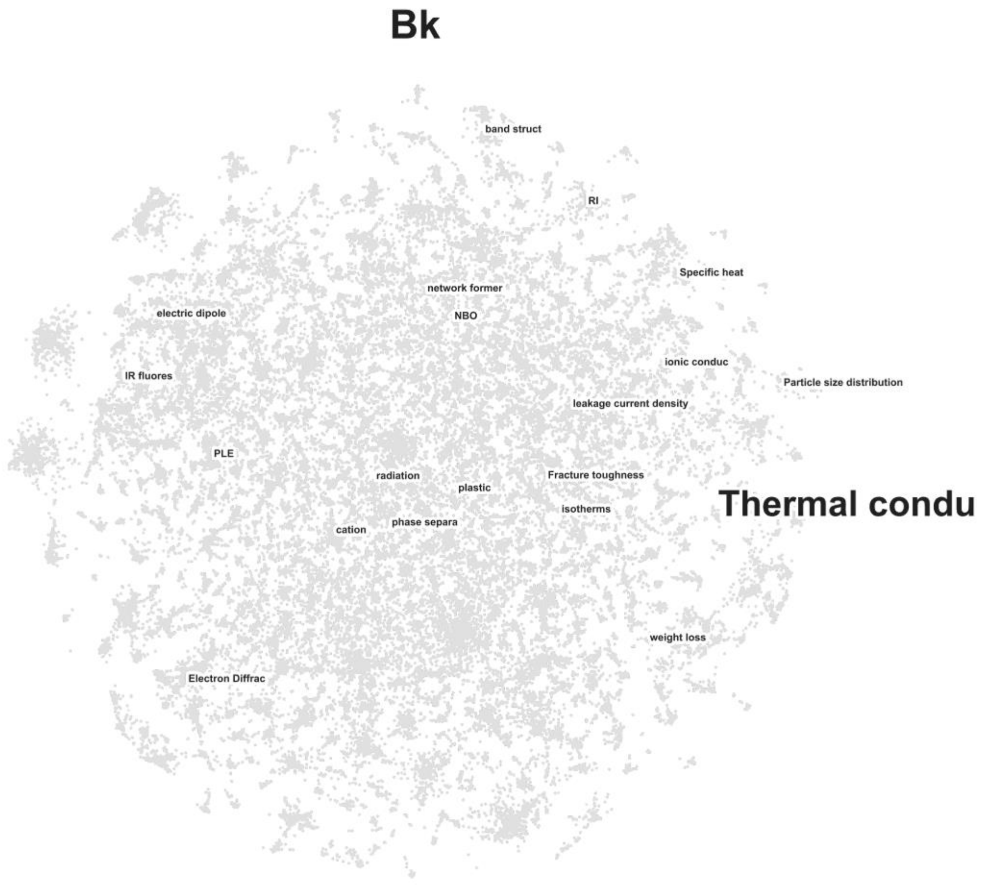

Figure S199. Latent Dirichlet Allocation plot for Berkelium.

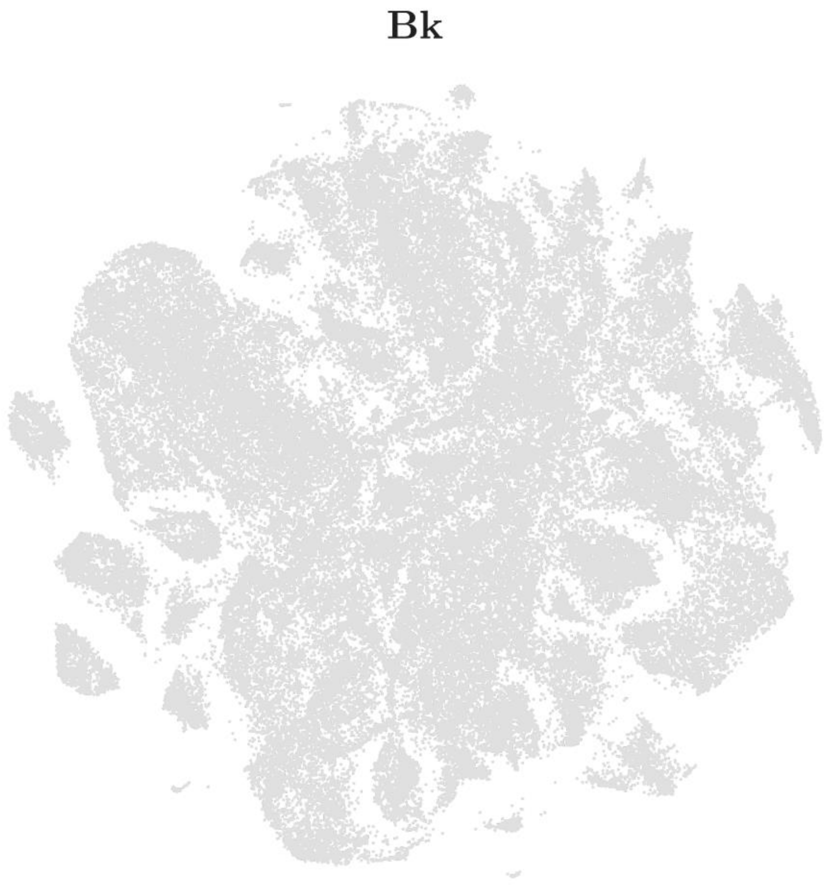

Figure S200. Caption Cluster plot for Californium.

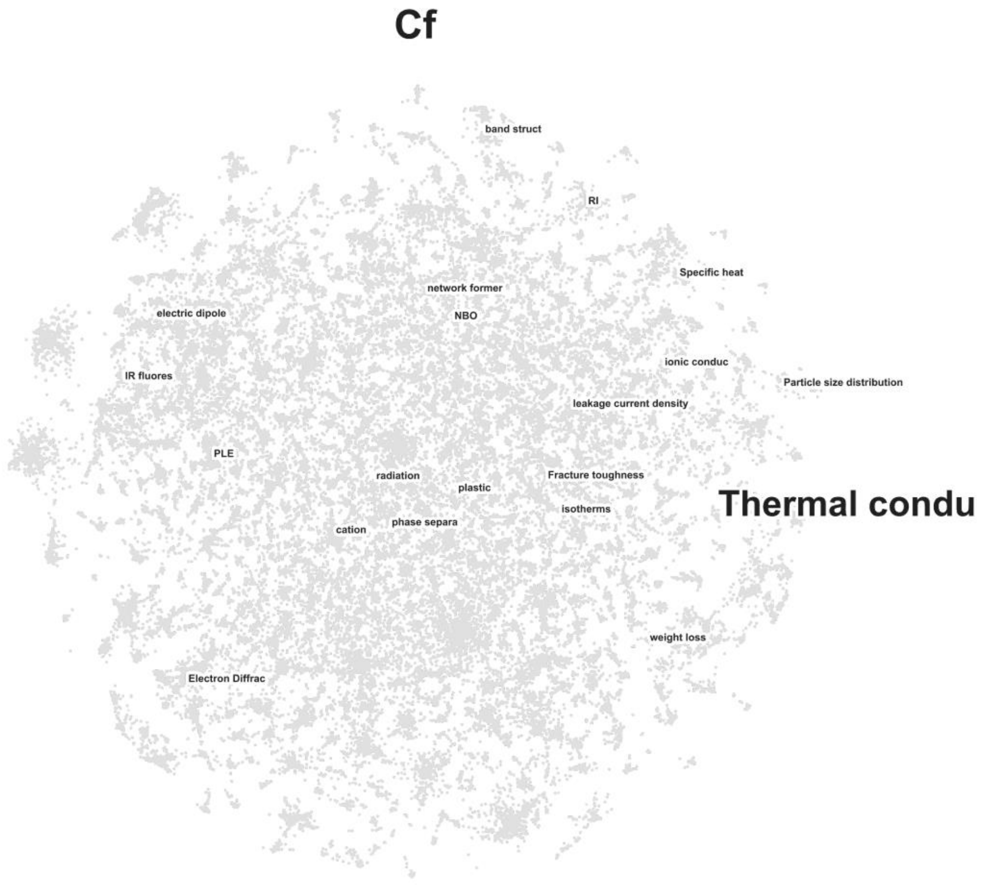

Figure S201. Latent Dirichlet Allocation plot for Californium.

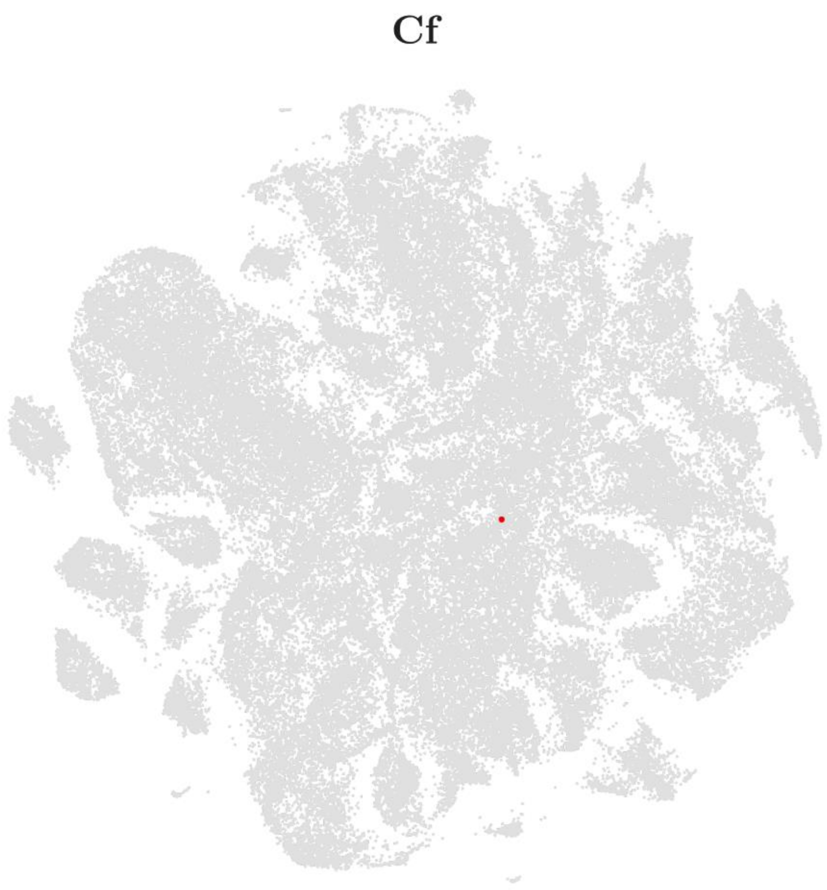

Figure S202. Caption Cluster plot for Einsteinium.

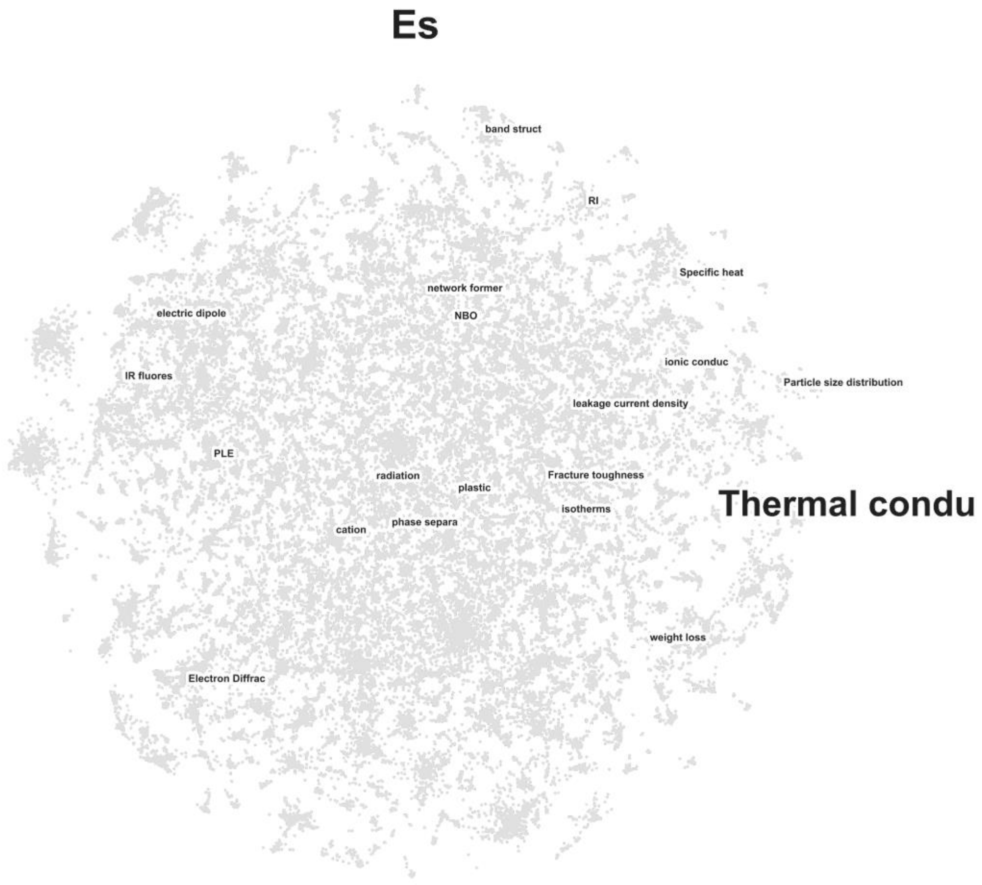

Figure S203. Latent Dirichlet Allocation plot for Einsteinium.

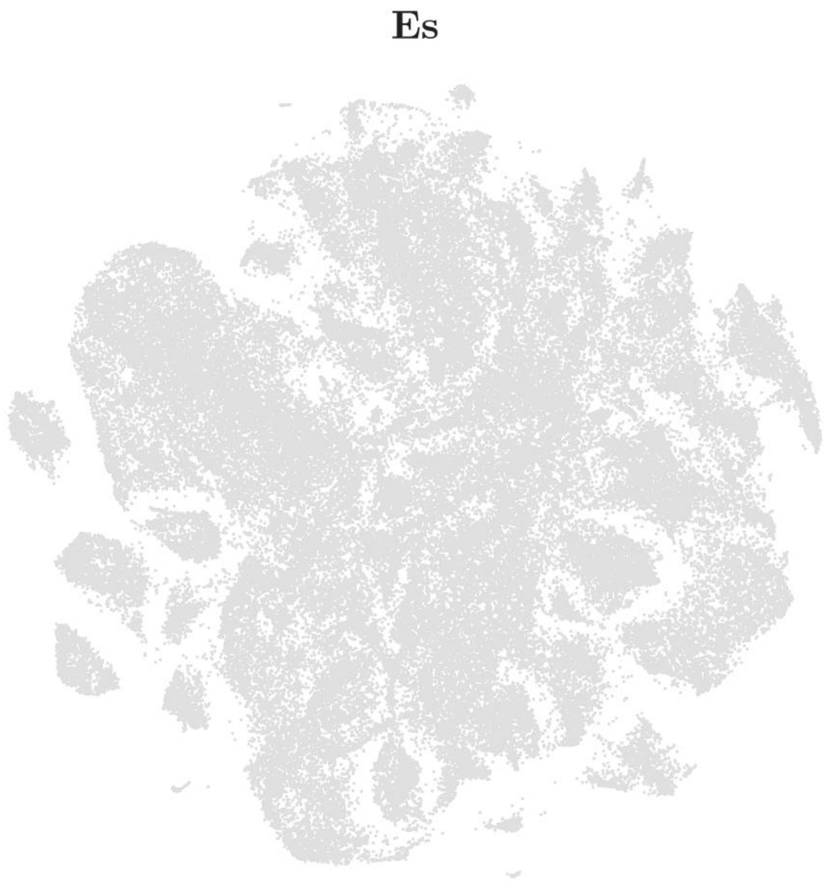

Figure S204. Caption Cluster plot for Fermium.

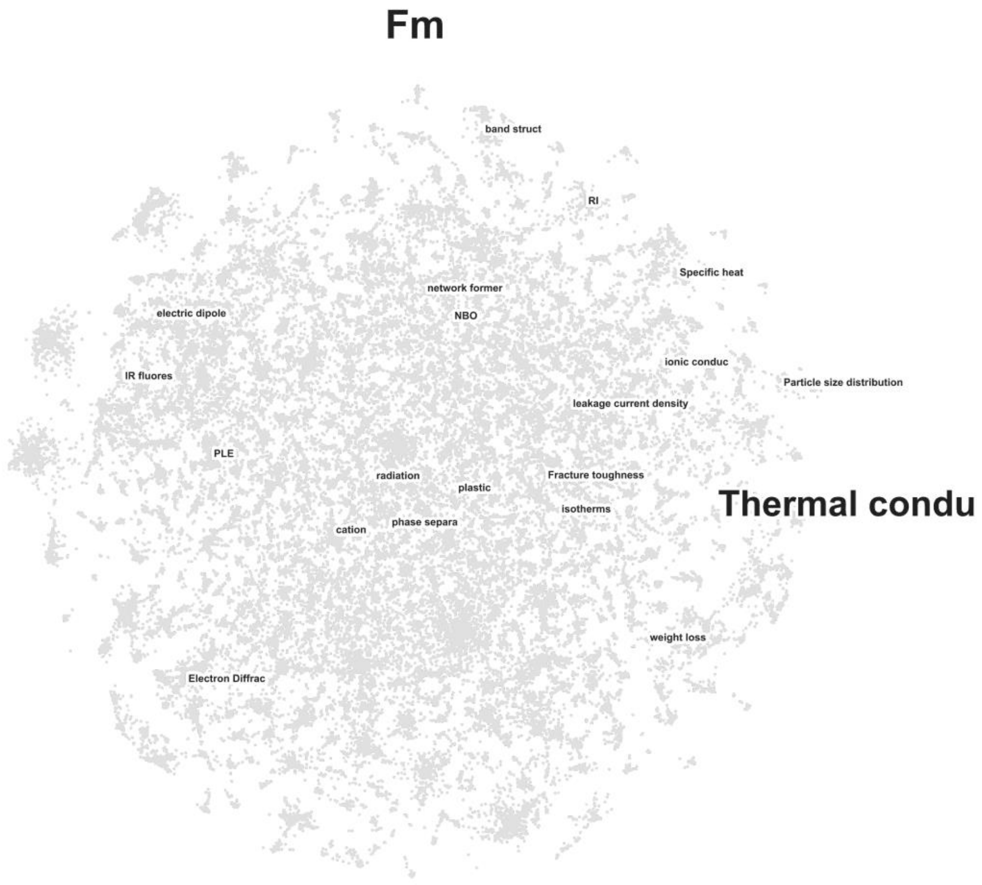

Figure S205. Latent Dirichlet Allocation plot for Fermium.

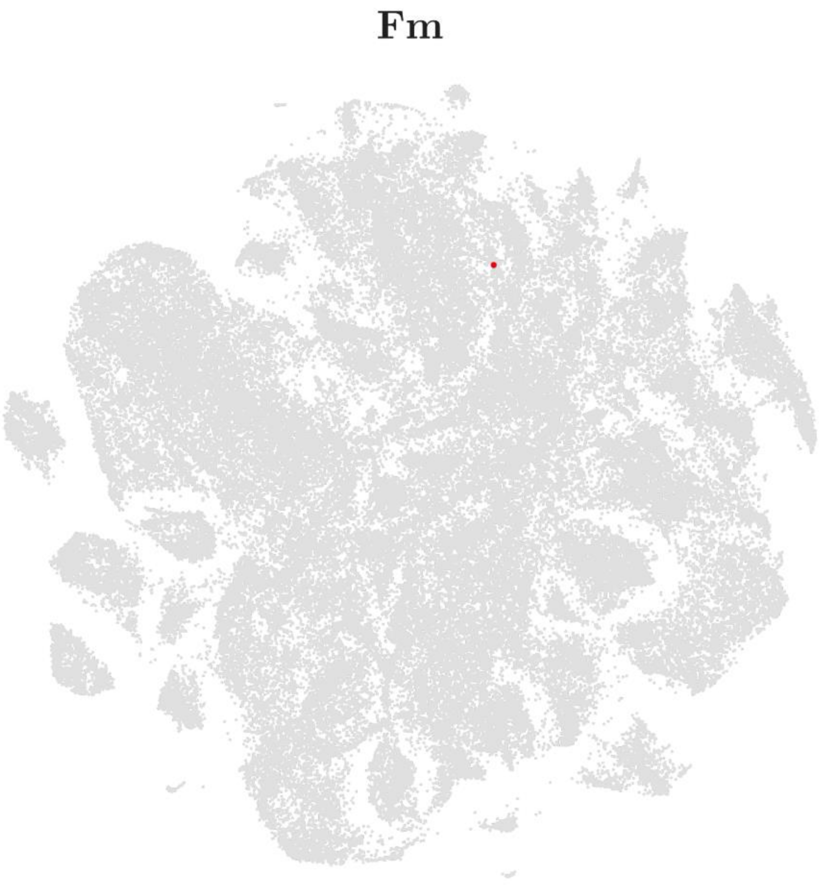

Figure S206. Caption Cluster plot for Mendelevium.

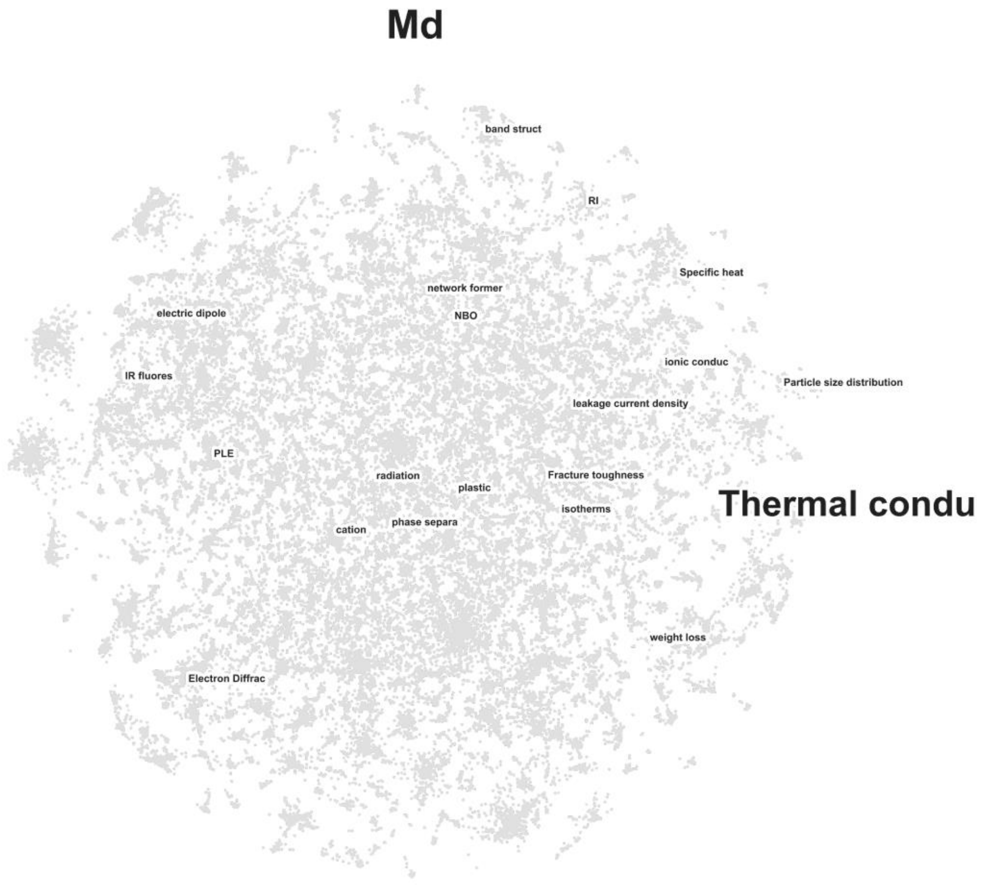

Figure S207. Latent Dirichlet Allocation plot for Mendelevium.

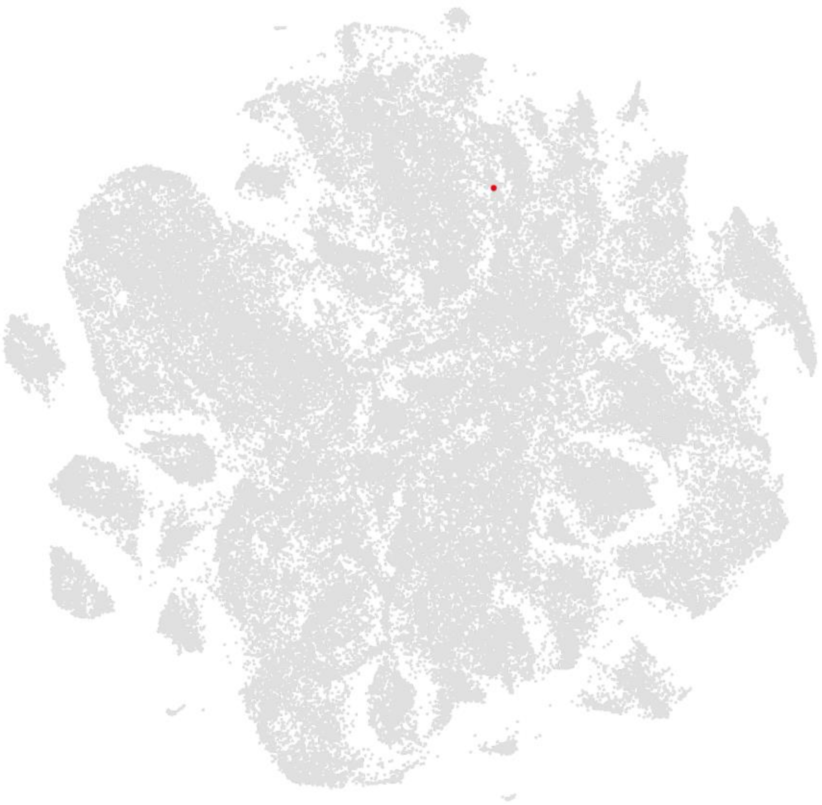

Figure S208. Latent Dirichlet Allocation plot for Nobelium.

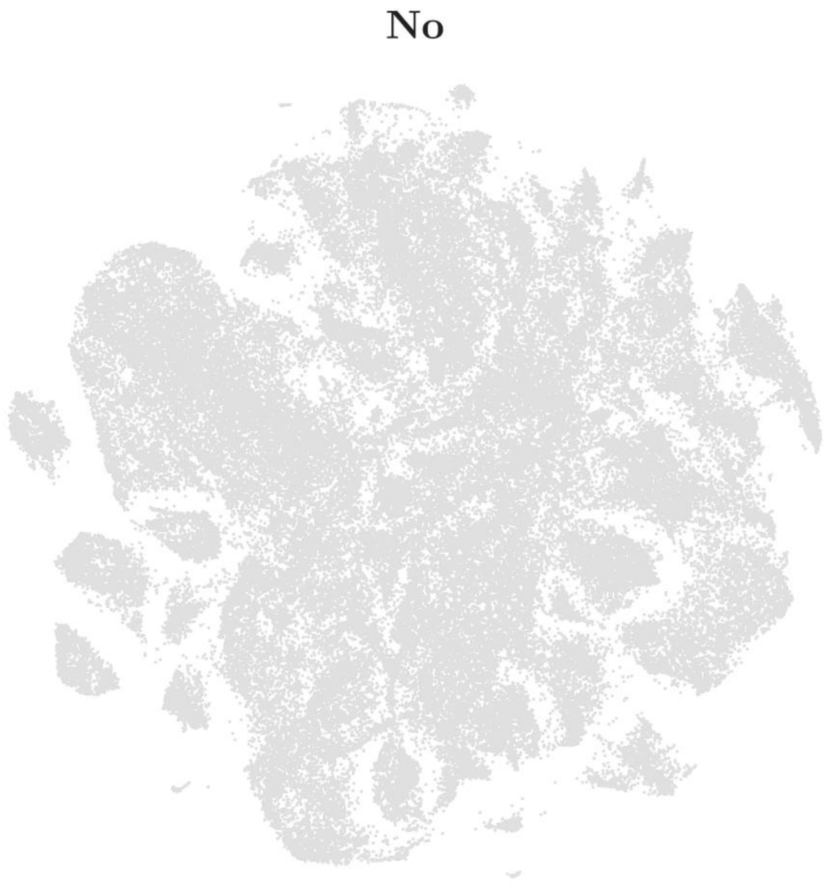

Figure S209. Latent Dirichlet Allocation plot for Lawrencium.

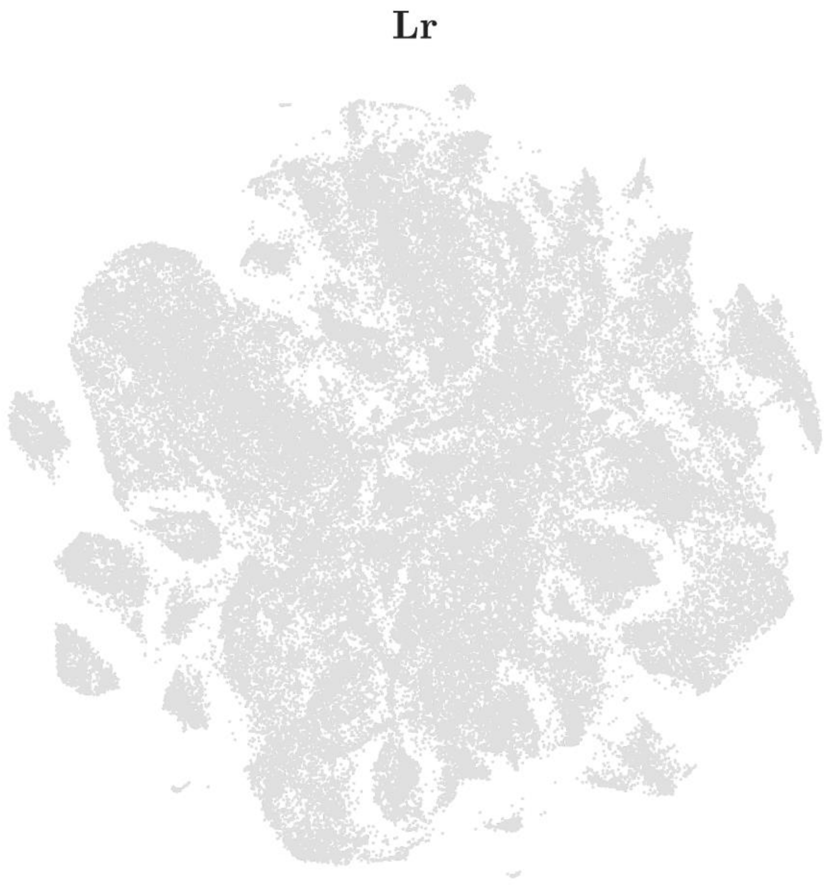

Figure S210. Latent Dirichlet Allocation plot for Rutherfordium.

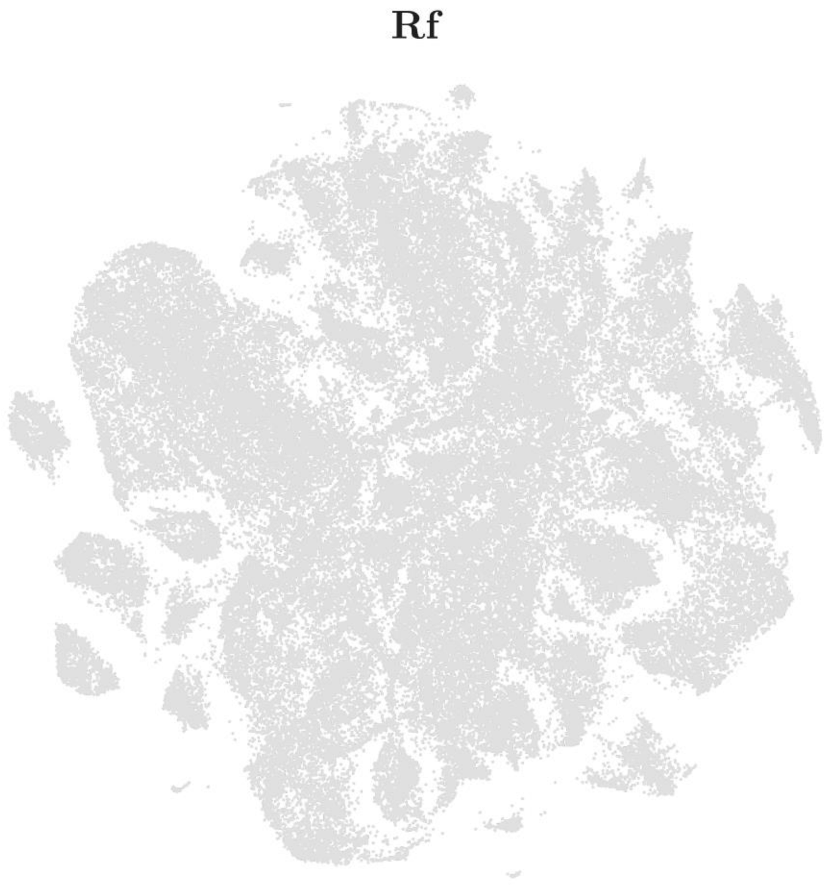

Figure S211. Latent Dirichlet Allocation plot for Dubnium.

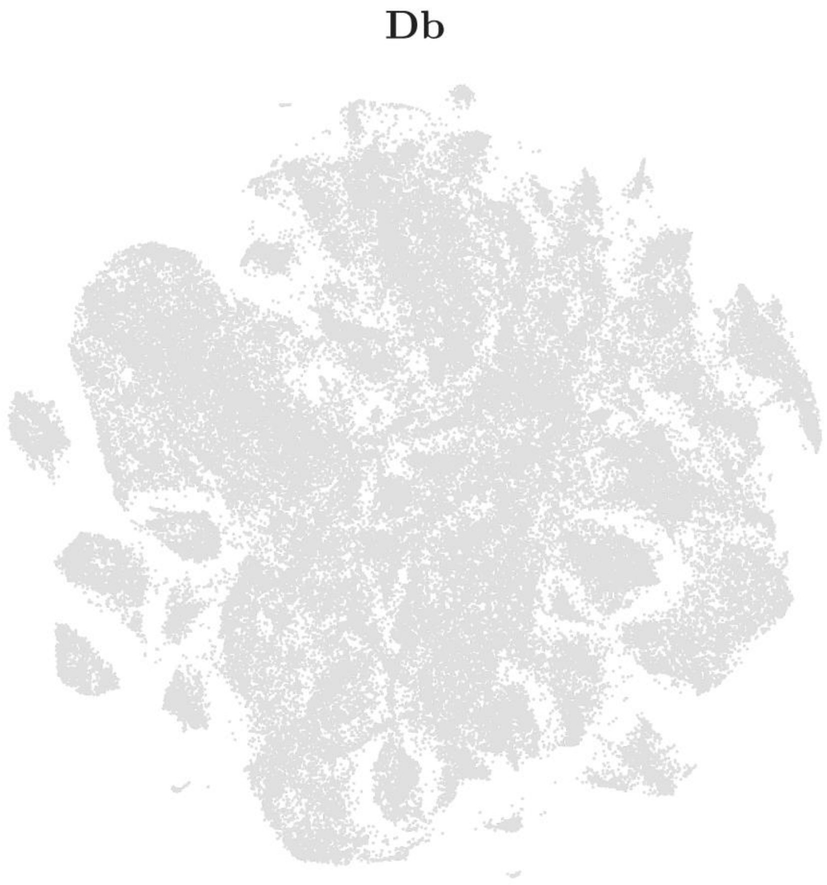

Figure S212. Latent Dirichlet Allocation plot for Seaborgium.

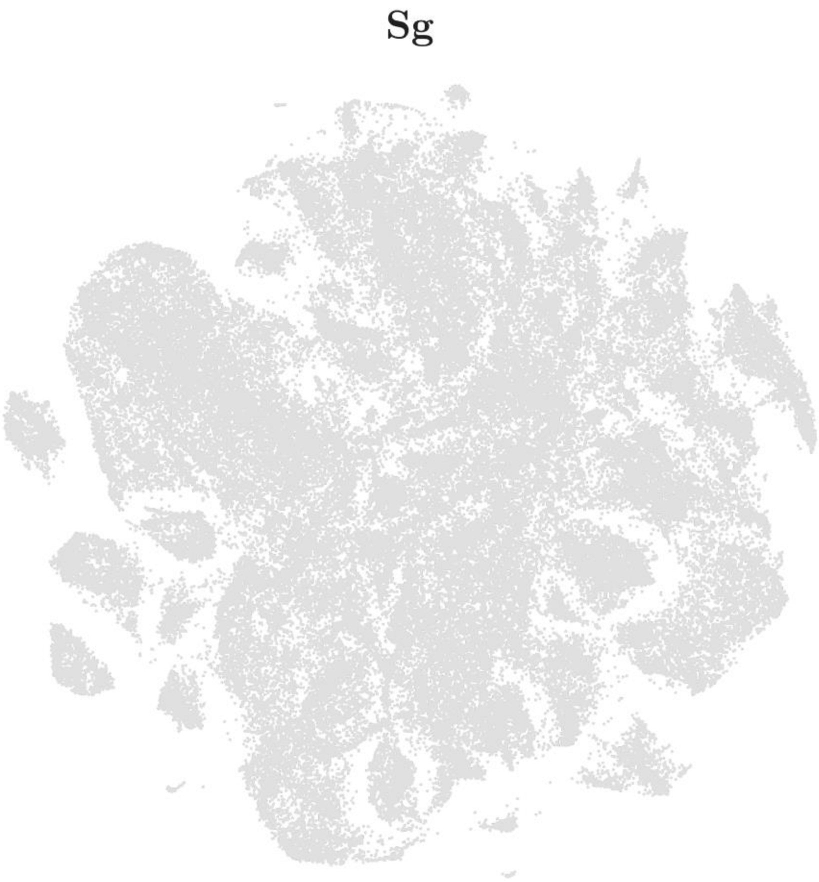

Figure S213. Latent Dirichlet Allocation plot for Bohrium.

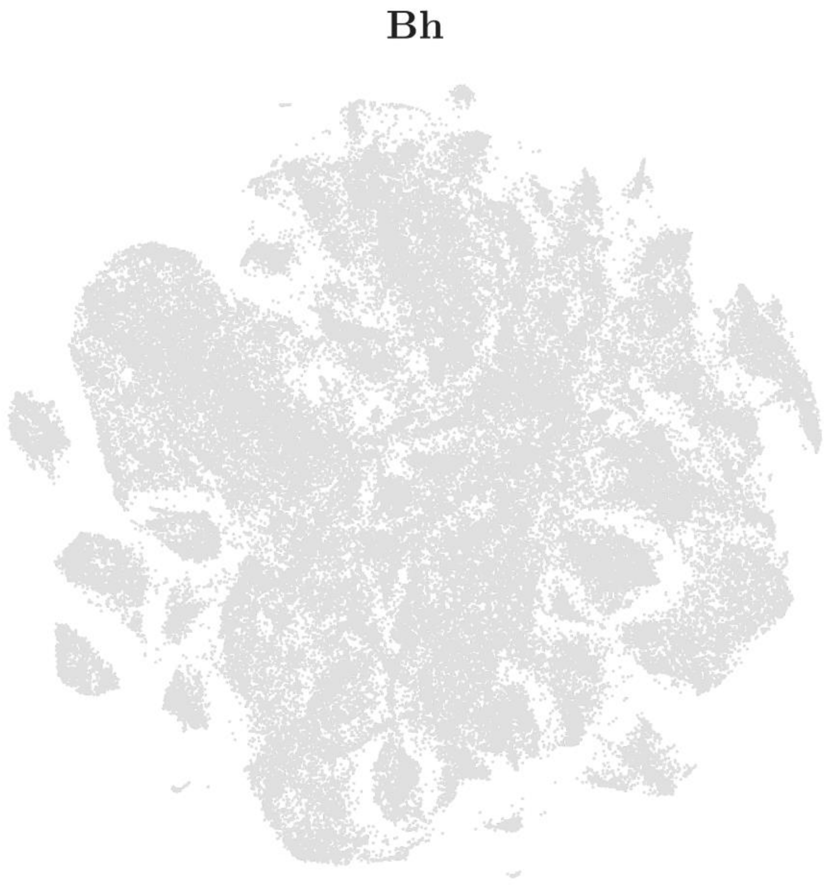

Figure S214. Latent Dirichlet Allocation plot for Hassium.

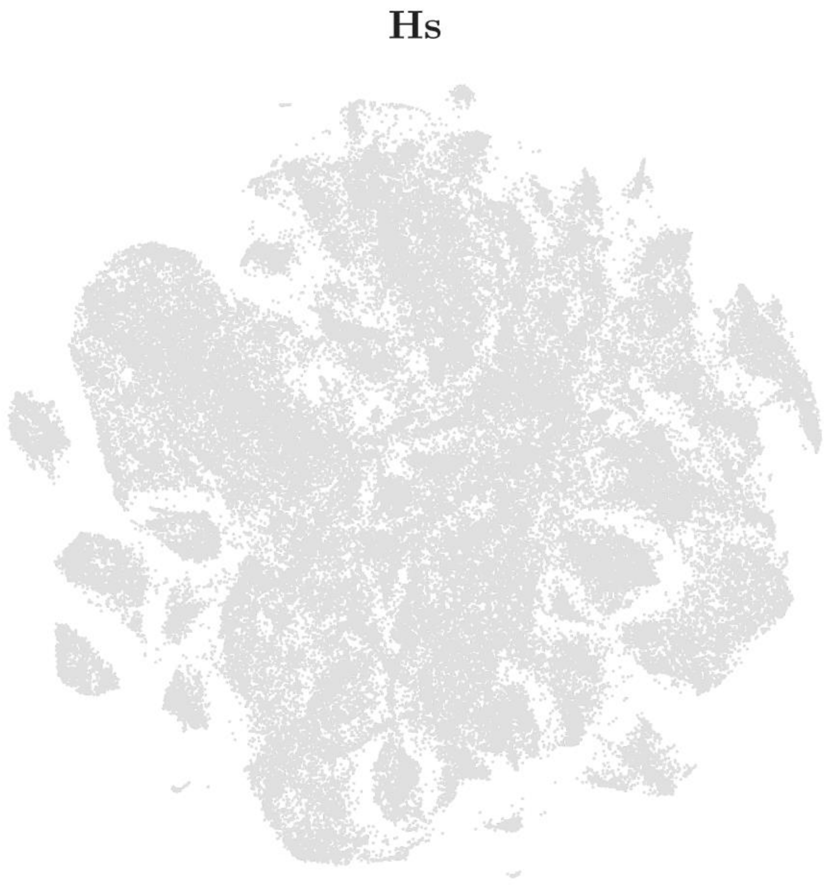

Figure S215. Latent Dirichlet Allocation plot for Meitnerium.

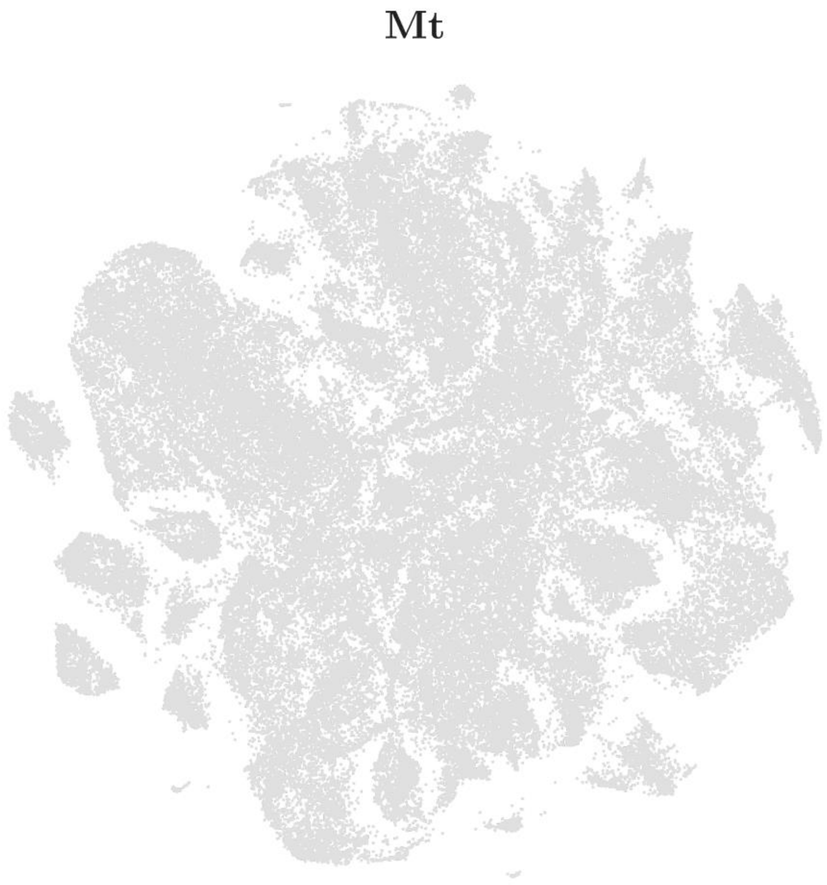

Figure S216. Latent Dirichlet Allocation plot for Darmstadtium.

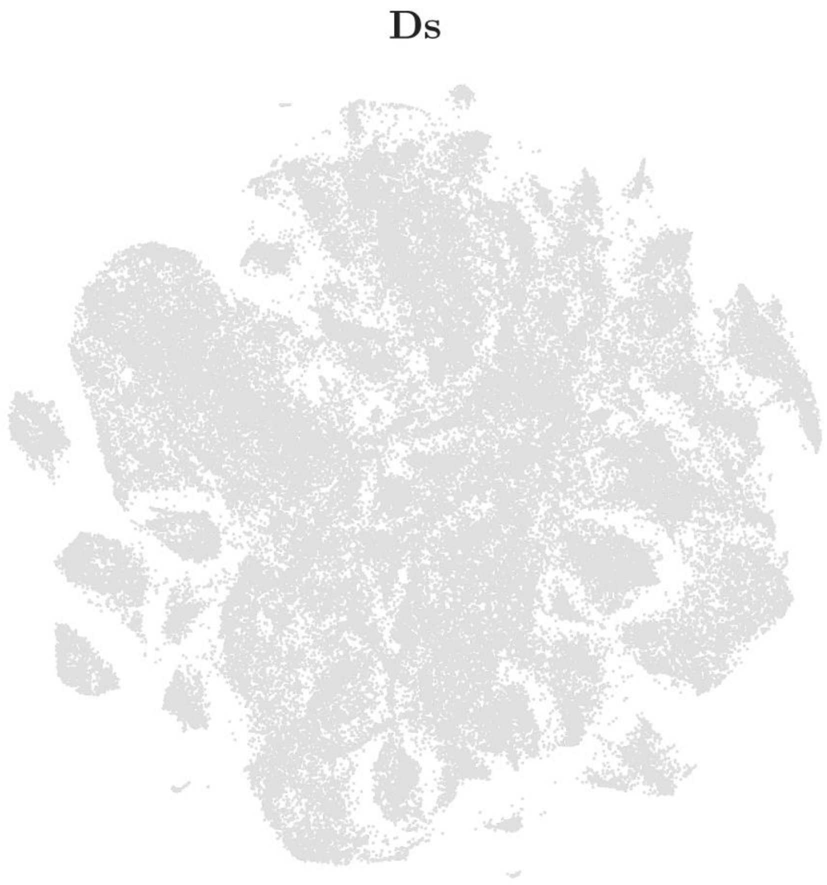

Figure S217. Latent Dirichlet Allocation plot for Roentgenium.

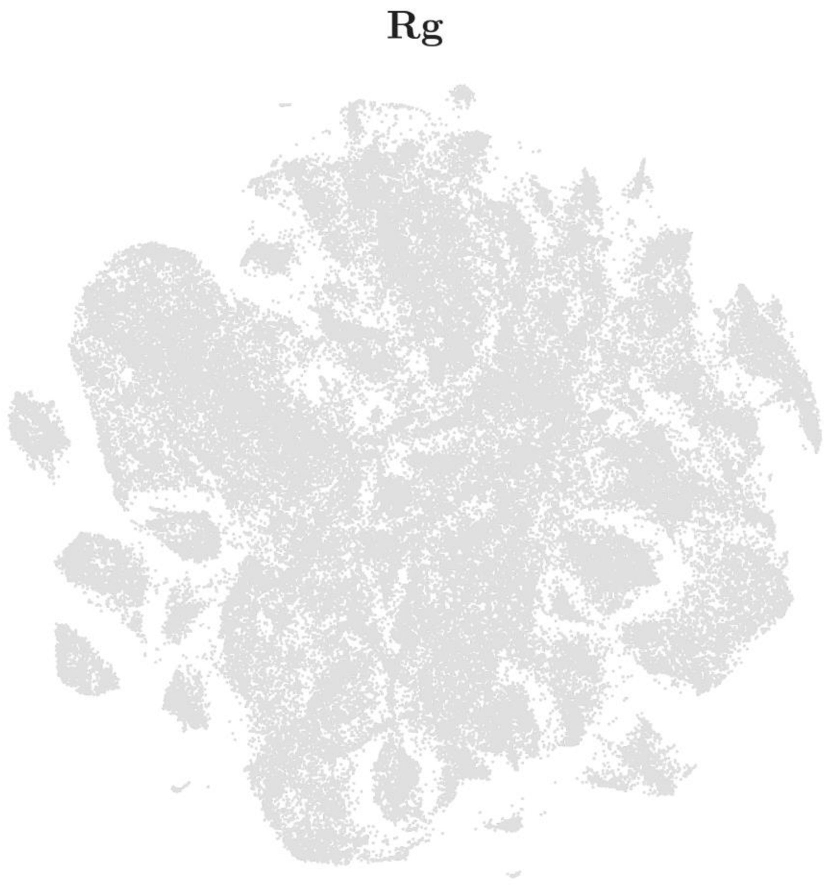

Figure S218. Latent Dirichlet Allocation plot for Copernicium.

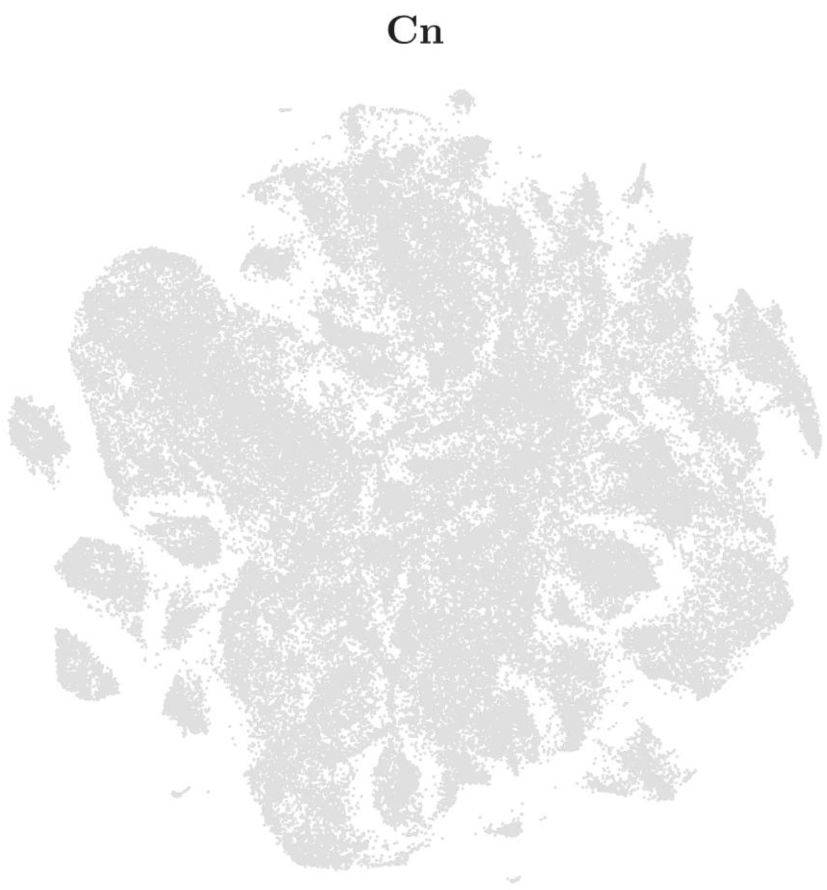

Figure S219. Latent Dirichlet Allocation plot for Nihonium.

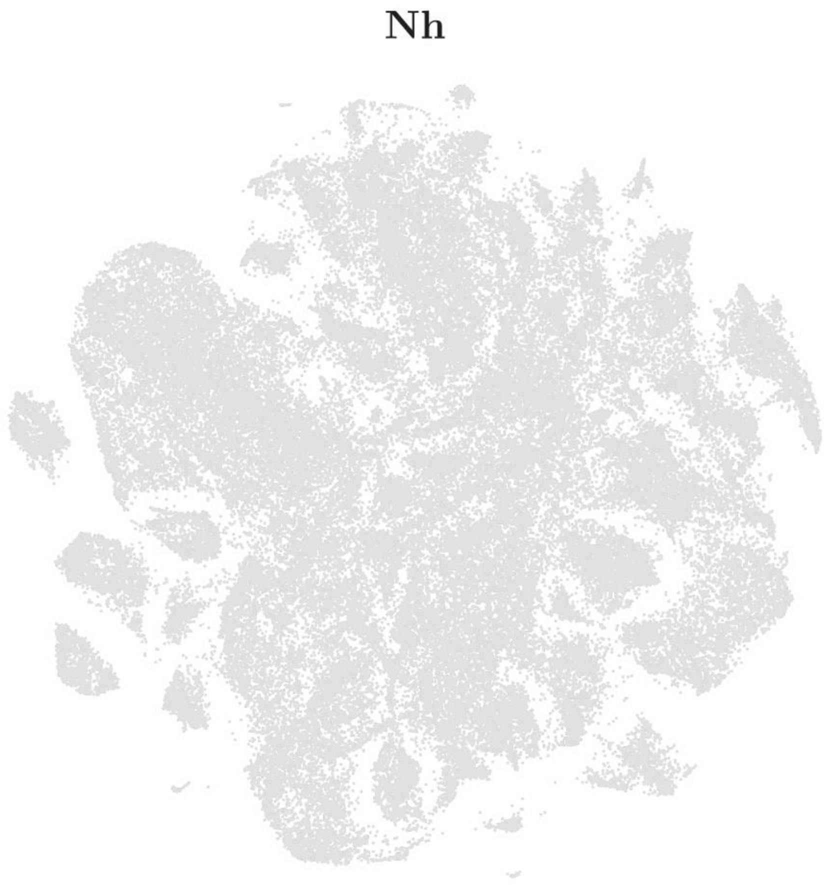

Figure S220. Latent Dirichlet Allocation plot for Flerovium.

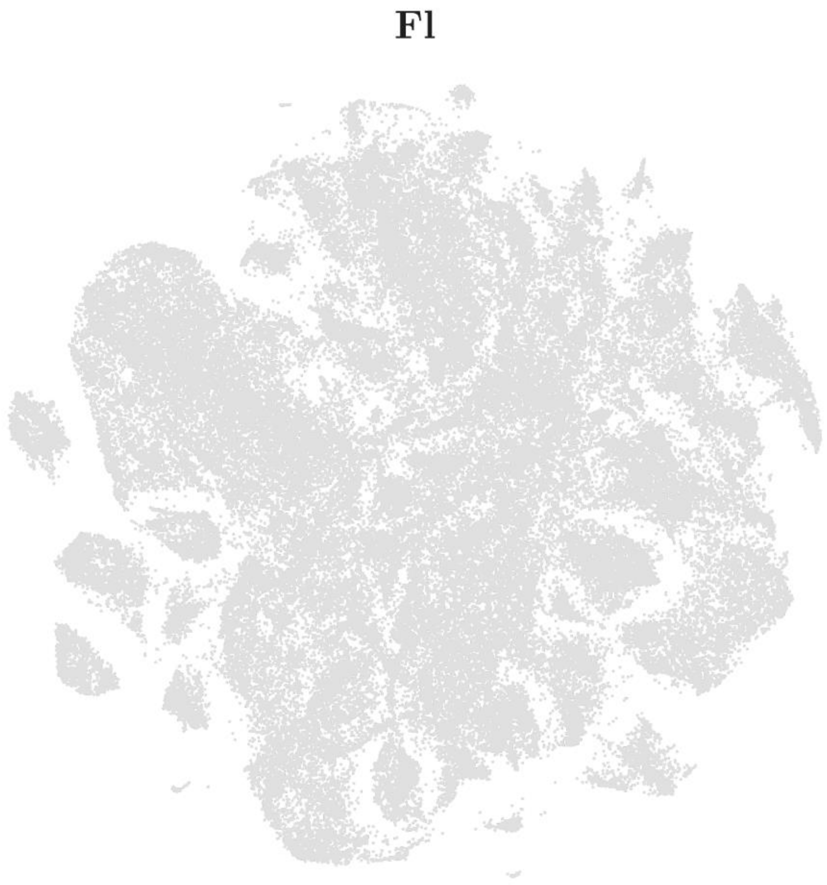

Figure S221. Latent Dirichlet Allocation plot for Moscovium.

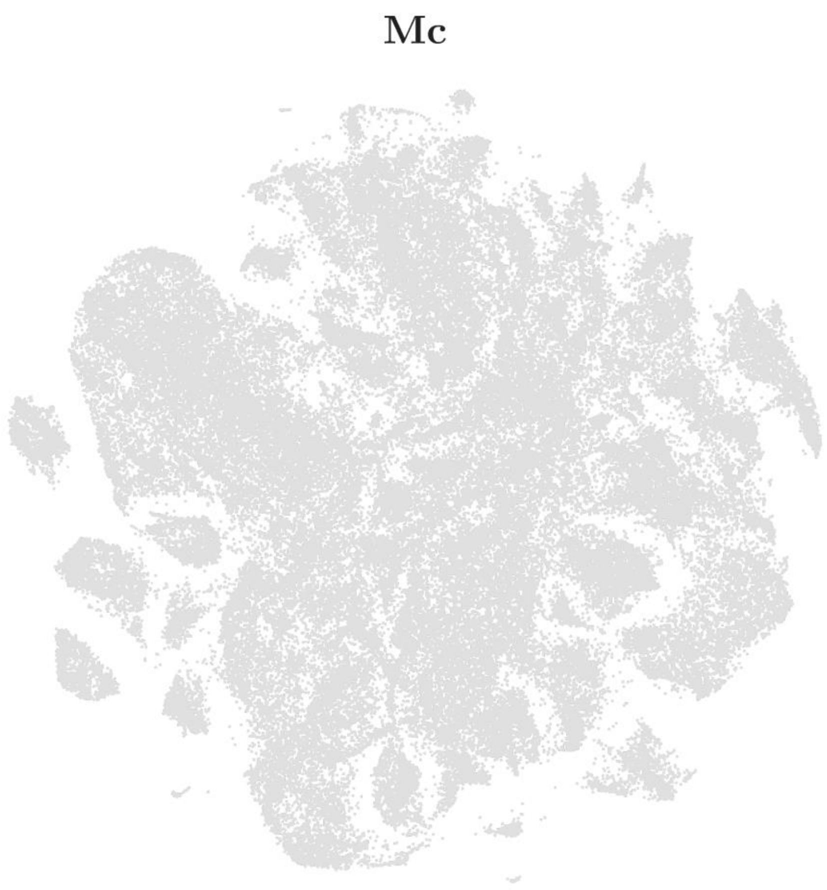

Figure S222. Latent Dirichlet Allocation plot for Livermorium.

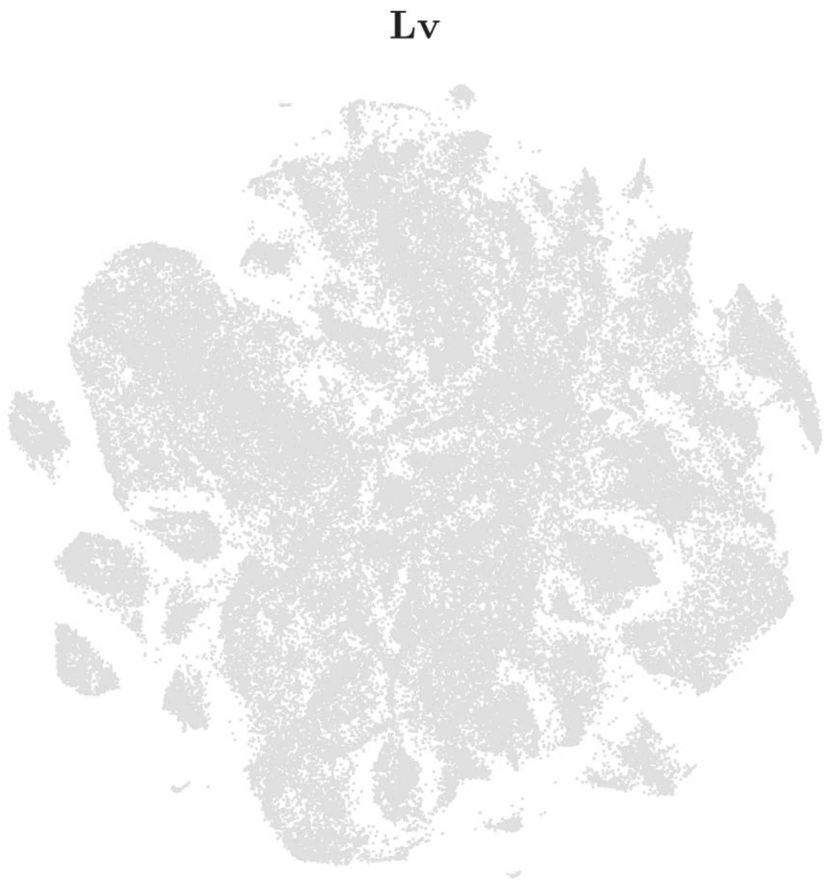

Figure S223. Latent Dirichlet Allocation plot for Tennessine.

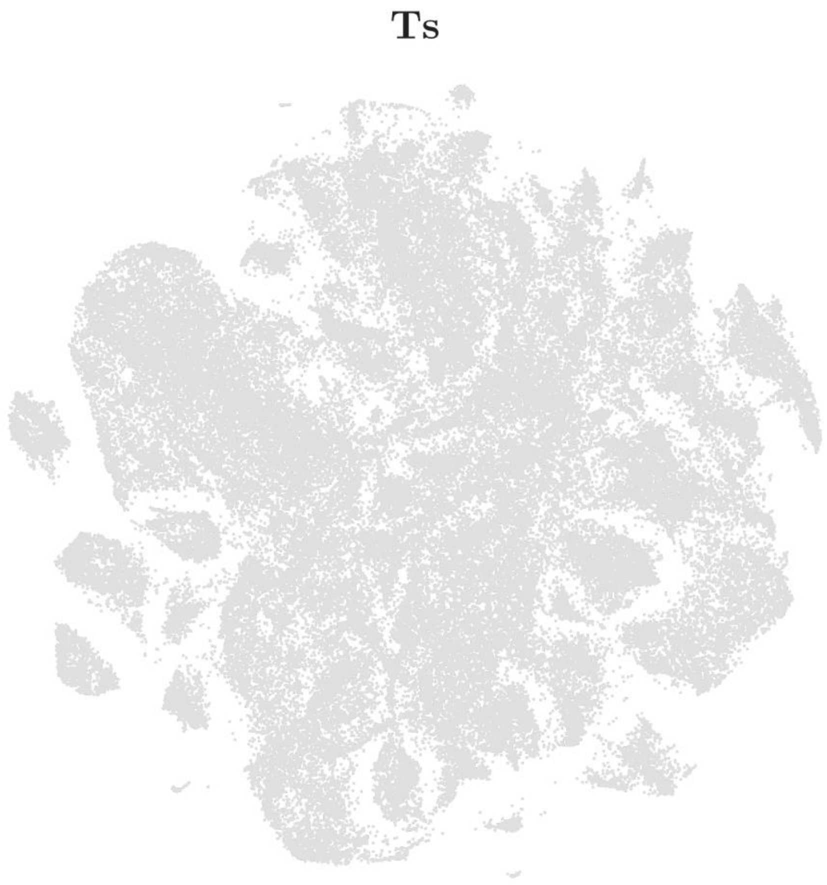

Figure S224. Latent Dirichlet Allocation plot for Oganesson.

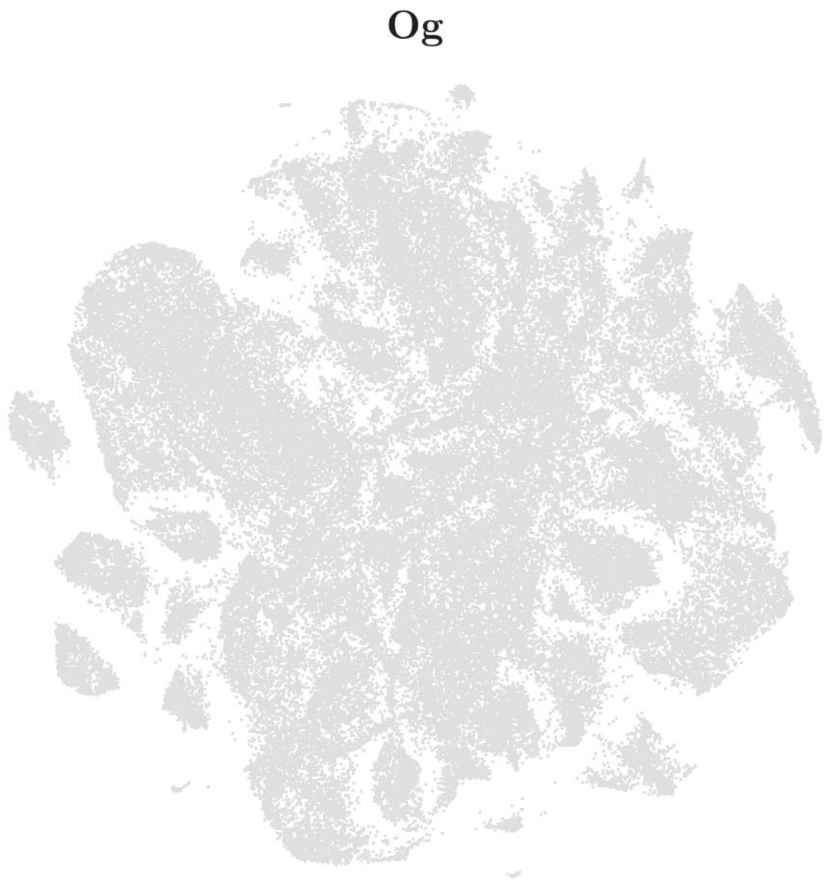

Figure S225. Latent Dirichlet Allocation plot for Ununennium.

Uue
